# The association of community mobility with the time-varying reproduction number (*R*) of SARS-CoV-2: a modelling study across 330 local UK authorities

**DOI:** 10.1016/S2589-7500(21)00144-8

**Published:** 2021-08-31

**Authors:** You Li, Xin Wang, Harry Campbell, Harish Nair

**Affiliations:** a Centre for Global Health, Usher Institute, University of Edinburgh, Edinburgh, UK; b School of Public Health, Nanjing Medical University, Nanjing, China

## Abstract

**Background:**

Community mobility data have been used to assess adherence to non-pharmaceutical interventions and its impact on SARS-CoV-2 transmission. We assessed the association between location-specific community mobility and the reproduction number (*R*) of SARS-CoV-2 across UK local authorities.

**Methods:**

In this modelling study, we linked data on community mobility from Google with data on *R* from 330 UK local authorities, for the period June 1, 2020, to Feb 13, 2021. Six mobility metrics are available in the Google community mobility dataset: visits to retail and recreation places, visits to grocery and pharmacy stores, visits to transit stations, visits to parks, visits to workplaces, and length of stay in residential places. For each local authority, we modelled the weekly change in *R* (the *R* ratio) per a rescaled weekly percentage change in each location-specific mobility metric relative to a pre-pandemic baseline period (Jan 3–Feb 6, 2020), with results synthesised across local authorities using a random-effects meta-analysis.

**Findings:**

On a weekly basis, increased visits to retail and recreation places were associated with a substantial increase in *R* (*R* ratio 1·053 [99·2% CI 1·041–1·065] per 15% weekly increase compared with baseline visits) as were increased visits to workplaces (*R* ratio 1·060 [1·046–1·074] per 10% increase compared with baseline visits). By comparison, increased visits to grocery and pharmacy stores were associated with a small but still statistically significant increase in *R* (*R* ratio 1·011 [1·005–1·017] per 5% weekly increase compared with baseline visits). Increased visits to parks were associated with a decreased *R* (*R* ratio 0·972 [0·965–0·980]), as were longer stays at residential areas (*R* ratio 0·952 [0·928–0·976]). Increased visits to transit stations were not associated with *R* nationally, but were associated with a substantial increase in *R* in cities. An increasing trend was observed for the first 6 weeks of 2021 in the effect of visits to retail and recreation places and workplaces on *R*.

**Interpretation:**

Increased visits to retail and recreation places, workplaces, and transit stations in cities are important drivers of increased SARS-CoV-2 transmission; the increasing trend in the effects of these drivers in the first 6 weeks of 2021 was possibly associated with the emerging alpha (B.1.1.7) variant. These findings provide important evidence for the management of current and future mobility restrictions.

**Funding:**

Wellcome Trust and Data-Driven Innovation initiative.

## Introduction

The first national lockdown in the UK was implemented on March 23, 2020, to reduce the transmission of the novel SARS-CoV-2 coronavirus. This lockdown led to an estimated 74% reduction in the daily number of contacts between individuals;^1^ compared with the pre-pandemic period, population mobility had reduced by about 60% nationwide at the end of March, 2020.^2^, ^3^ Following the gradual relaxation of the lockdown in May, 2020, a resurgence of COVID-19 cases began in the late summer, with the epidemic trajectories beginning to differentiate in different parts of the UK. This resurgence led to the introduction of regionally differentiated non-pharmaceutical interventions, such as the three-tiered approach for England and the five-tiered approach for Scotland, in October, 2020.

Mobility data have been proposed as a method of monitoring adherence to non-pharmaceutical interventions and the effect of these interventions on transmission dynamics;^2^, ^4^, ^5^, ^6^ mobility data have also been used to identify locations or venues associated with an increased probability of super-spreading events.^7^, ^8^ Existing studies suggest that changes in local mobility are strongly correlated with transmission of SARS-CoV-2 and that the correlation might vary over time (appendix pp 3–5). Several studies from the USA and China have highlighted the effect of population mobility on SARS-CoV-2 transmission at the local level. However, few data are available that compare and quantify the association between different location-specific mobility metrics at the local level and transmission of SARS-CoV-2. Moreover, since the epidemic trajectories differed by region in the UK, it became important to understand the differences in mobility, socioeconomic factors, and transmissibility of SARS-CoV-2.

**Research in context**

**Evidence before this study**

We searched PubMed for studies that reported the association between local community mobility and the time-varying reproduction number (*R*), published between Jan 1, 2020, and June 18, 2021, using the search terms “COVID-19”, “SARS-CoV-2”, “mobility”, and “transmission”. No language restrictions were applied. Existing data suggest that changes in local mobility are well correlated with transmission of SARS-CoV-2 and that the correlation might vary over time. However, there is a scarcity of data that compare and quantify the association between different location-specific mobility metrics at the local level and transmission of SARS-CoV-2.

**Added value of this study**

Our findings provide new insights into the heterogeneities in mobility, socioeconomic factors, and transmissibility of SARS-CoV-2 in the UK. We highlight visits to retail and recreation places as an important mobility metric associated with a substantial increase in *R* of SARS-CoV-2; by contrast, increased visits to grocery and pharmacy stores were associated with a small increase in *R*. We report a smaller effect size in the north than in the south of the UK in the increase of *R* associated with visits to workplaces. We report that increased visits to parks and increased time spent in residential areas were associated with a decreased *R*. Increased visits to transit stations were not found to be associated with an increased *R* at the national level; however, two independent sensitivity analyses suggested that increased visits to transit stations were associated with a substantial increase in *R* in UK cities. Furthermore, we highlight an increasing trend for the first 6 weeks of 2021 in the effect size for visits to retail and recreation places and visits to workplaces, possibly associated with the emerging alpha (B.1.1.7) variant.

**Implications of all the available evidence**

Increased visits to retail and recreation places, workplaces, and transit stations in cities are important drivers of SARS-CoV-2 transmission, and the emergence of new variants of concern might further amplify the effects of these drivers. These findings provide important evidence for the management of current and future mobility restrictions.

In this modelling study, we aimed to assess the association between various location-specific community mobility metrics and the time-varying reproduction number (*R*) across local UK authorities. We also aimed to explore whether any such association changed over time, and whether this association differed by *R* range, or according to characteristics of the local authority.

## Methods

### Data sources

For this modelling study, we included a community mobility dataset from the Google COVID-19 Community Mobility Reports, which has been published as a preprint online.^9^ The Google community mobility dataset consists of daily aggregated data on changes in number of visits and length of stay at different location categories compared with a baseline period (Jan 3–Feb 6, 2020). This dataset is based on data from users who have opted in to provide their so-called location history from their Google accounts. Six mobility metrics are available in the Google community mobility dataset: visits to retail and recreation places (eg, restaurants, cafes, shopping centres, theme parks, museums, libraries, and cinemas), visits to grocery and pharmacy stores (eg, grocery markets, food warehouses, farmers' markets, specialty food shops, and pharmacies), visits to transit stations (eg, bus, underground, and train stations), visits to parks, visits to workplaces, and length of stay in residential places. Data for each mobility metric in a UK local authority were available as the percentage change compared with the baseline period. All reported mobility metrics were based on Google mobility metrics unless otherwise specified. For sensitivity analysis, we collected data on public transit requests for 16 major cities in the UK from the Apple Maps Mobility Trends Reports. We included data on R estimates for each local authority published by Imperial College London (London, UK). The *R* for each local authority was estimated on the basis of reported COVID-19 cases and deaths using semi-mechanistic Bayesian models. The time lags between infection and reporting of cases, and between infection and reporting of deaths, were accounted for in the model, thus the *R* estimate was expected to reflect the instantaneous transmissibility of SARS-CoV-2. For each local authority, we also obtained latest data on population density, socioeconomic deprivation, the proportion of the population who were Black, Asian, or from minority ethnic groups, latitude, and the proportion of large households (defined as a household with six people or more) since these characteristics might be associated with change in *R*. We followed the STROBE guidelines for the reporting of our study (appendix pp 148–149). Data sources are summarised in the appendix (p 6).

### Data processing

To incorporate the statistical uncertainty of the *R* estimates, we resampled 100 times for each *R* estimate on the basis of estimated credible intervals (appendix p 2), which yielded 100 individual datasets of *R*. Both the *R* dataset and the community mobility dataset were aggregated by week before they were linked by local authority. The linked dataset was the working dataset of our analysis, which consisted of weekly data on community mobility metrics and *R* (100 individual estimates) for each local authority, between June 1, 2020, and Feb 13, 2021 (ie, the end date of our data collection). We excluded the 3 weeks of the Christmas and New Year festive period (Dec 21, 2020, to Jan 10, 2021), considering the possible data artifacts during the festive period.

### Data analysis

**Figure 1:**
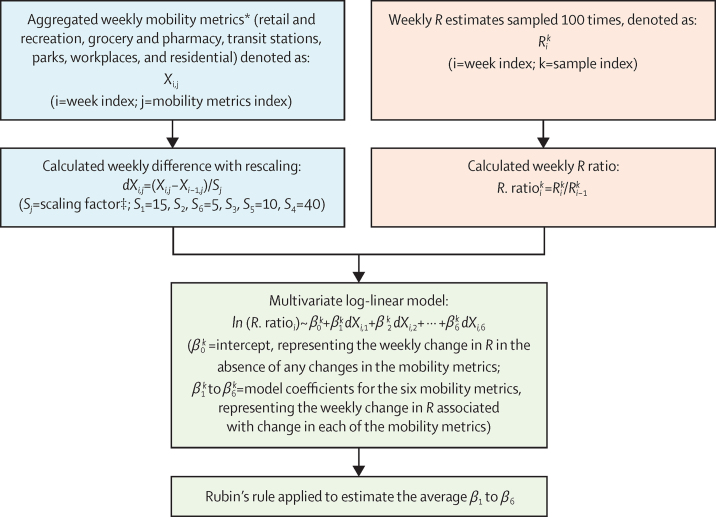
Modelling framework for each local authority For Rubin's rule see Barnard and Rubin.^10^*R*=reproduction number. *Mobility metrics were available as the percentage change compared with the baseline period (Jan 3–Feb 6, 2020).

For each local authority, we used a multivariate log-linear regression to estimate the association between the weekly change in the community mobility metrics and the corresponding weekly change in *R* (as the *R* ratio; figure 1). An *R* ratio of more than 1 indicated increased transmission compared with the previous week—ie, one infected case could infect more susceptible individuals than they could in the previous week. An *R* ratio of less than 1 indicated decreased transmission. Since several time-varying factors (eg, testing practice and personal hygiene) did not change substantially per week, we chose to model the incremental weekly change in *R* with the incremental weekly change in mobility metrics (rather than using the absolute *R* and mobility metrics), which would cancel out the effects of these factors on *R*.

Because the scale of change in the six community mobility metrics was different (eg, the change in the visits to parks has been much greater than the change in the visits to grocery and pharmacy stores since the start of the COVID-19 pandemic), we decided to rescale these community mobility metrics in the regression model to facilitate the interpretation of the model coefficients and to reflect their potential in driving *R*. These rescaling factors were determined, before running the model, by comparing the 90th percentile of the weekly change in these metrics with the baseline period (ie, Jan 3–Feb 6, 2020) and rounding to the nearest 5. As a result, the model coefficient could be interpreted as the associated *R* ratio per rescaled unit change in the mobility metric (figure 1). The regression model was fitted with each of the 100 sampled *R* estimates separately, yielding 100 sets of coefficients, which were then averaged by applying Rubin's rules.^10^ Collinearity was assessed by calculating covariance-inflation factors.

In our working dataset, 330 (86%) of 382 local authorities had sufficient data (defined as complete data for >70% of the weeks in the study period) on all the six community mobility metrics (appendix pp 17–143). Thus, these 330 local authorities were included in the main analysis. We then did a random-effects meta-analysis of the *R* ratios across the local authorities using the DerSimonian-Laird estimator^11^ for each community mobility metric. The pooled *R* ratios for the six community mobility metrics from the meta-analysis were regarded as the primary measures in our analysis. To adjust for multiple testing (ie, six tests for six metrics), we applied the Bonferroni correction for the significance level and reported the 99·2% CIs for the primary measures, in addition to the conventional 95% CIs.

We did several sets of prespecified secondary analyses to gain more insights into the results (appendix p 7). First, we limited the analysis to only the 16 largest UK cities (based on population size); additionally, we did a sensitivity analysis by replacing the Google metric of visits to public transit stations with the Apple Map metric of public transit requests for further validation. Second, we excluded the metric of residential stay, since this metric was measured by duration rather than number of visits (as in the other five mobility metrics). Third, we replaced the error term in the main model with a time-varying autoregressive error term to account for autocorrelation. Fourth, we applied a time lag of 1 week to the effect of mobility metrics in the main model to account for possible lagged effect. Fifth, we did a meta-analysis within each region of the UK to understand possible regional differences. Sixth, we did a stratified analysis by *R* range for the week—ie, 0·5–1·0 and 1·0–1·5—to explore whether the association differed by *R* range (we were unable to include other ranges of *R* due to an absence of data). Seventh, we explored whether the association changed over time by repeating the main analysis using data from a 13-week moving time window. Eighth, we did a meta-regression for each community mobility metric using characteristics at the level of local authority as predictors, including latitude, population density, prosperity score, and proportion of the population who were BAME; for the metric of length of time spent in residential areas, proportion of large households was included as one additional predictor. Considering the exploratory nature of the secondary analyses, we reported only the 95% CIs, without adjustment for multiple testing. All data analyses and data visualisations were done with *R* software (version 3.6.2).

### Role of the funding source

The funders of the study had no role in study design, data collection, data analysis, data interpretation, writing of the manuscript, or the decision to submit for publication.

## Results

**Figure 2:**
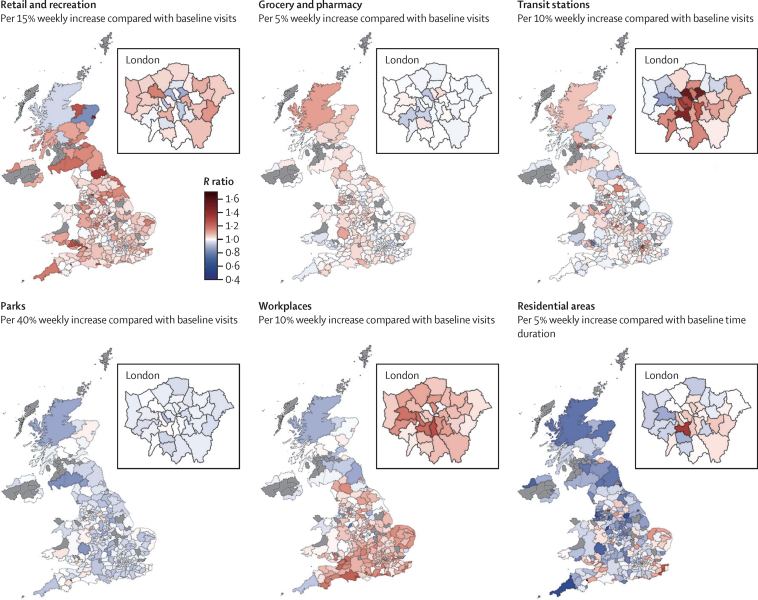
Association between six Google community mobility metrics and *R* for individual local authorities The association is shown as weekly change in *R* (*R* ratio) associated with weekly difference in mobility metrics relative to the baseline level (baseline period Jan 3– Feb 6, 2020). Only point estimates are presented; full estimates with CIs are in the appendix (pp 8–15). *R*=reproduction number.

Our main findings were informed by the meta-analysis across 330 local authorities. The results for individual local authorities are available in figure 2 and the appendix (pp 8–15). The main model accounted for a median of 42% (IQR 34–48) of the variations in *R* ratios across all local authorities. Increased visits to retail and recreation places were associated with a statistically significant increase in *R* (*R* ratio 1·053 [99·2% CI 1·041–1·065] per 15% weekly increase compared with baseline visits; table 1). By comparison, increased visits to grocery and pharmacy stores were associated with a small but statistically significant increase in *R* (1·011 [1·005–1·017] per 5% weekly increase compared with baseline visits). Increased visits to workplaces were associated with a substantial increase in *R* (1·060 [1·046–1·074] per 10% weekly increase compared with baseline visits); a smaller effect was seen in the *R* ratio in northern than in southern parts of the UK (Pearson's correlation coefficient [*r*]=–0·518, p<0·0001). Increased visits to parks and increased time spent in residential areas were both associated with decreases in *R* (0·972 [0·965–0·980] per 10% weekly increase compared with baseline visits; 0·952 [0·928–0·976] per 5% weekly increase per duration); for increased time spent in residential areas, a smaller effect was seen in the *R* ratio in northern than in southern parts of the UK (*r*=–0·328, p<0·0001). The UK-wide meta-analysis results indicated that increased visits to transit stations were not associated with increases in *R*; however, when limited to the 16 major cities, increased visits to transit stations were associated with a substantial increase in *R* (1·072 [95% CI 1·024–1·122] per 10% weekly increase compared with baseline visits) and a similar result was observed when using the Apple Map transit requests (1·055 [1·035–1·075] per 10% weekly increase compared with baseline requests). The results of sensitivity analyses that excluded the mobility metric of residential stay, included an autoregressive error term, or applied a 1-week time lag were similar to those of the main analysis (appendix p 144).

**Table 1:** Association between Google community mobility metrics and *R* across 330 local authorities in the UK

		***R* ratio**	**95% CI**	**99·2% CI**
Retail and recreation (per 15% weekly increase compared with baseline visits)		1·053	1·044–1·062	1·041–1·065
Grocery and pharmacy (per 5% weekly increase compared with baseline visits)		1·011	1·006–1·016	1·005–1·017
Transit stations (per 10% weekly increase compared with baseline visits)		1·000	0·994–1·006	0·991–1·009
	In 16 major cities*	1·072	1·024–1·122	..
	In 16 major cities using Apple transit requests	1·055	1·035–1·075	..
	In rest of the UK	0·999	0·992–1·005	..
Parks (per 40% weekly increase compared with baseline visits)		0·972	0·967–0·978	0·965–0·980
Workplaces (per 10% weekly increase compared with baseline visits)		1·060	1·049–1·070	1·046–1·074
Residential (per 5% weekly increase compared with baseline time length)		0·952	0·934–0·970	0·928–0·976

Association is shown as weekly change in *R* (*R* ratio) associated with weekly difference in mobility metrics relative to the baseline level (baseline period Jan 3–Feb 6, 2020). *R*=reproduction number.

* Belfast, Birmingham, Bradford, Bristol, Cardiff, Edinburgh, Glasgow, Leeds, Liverpool, London, Manchester, Newcastle upon Tyne, Nottingham, Portsmouth, Reading, and Sheffield.

Some regional variations in the associations between mobility metrics and *R* were also identified (table 2). The effect size of increased visits to retail and recreation places was greatest in northeast England (*R* ratio 1·113 [95% CI 1·056–1·173] per 15% weekly increase compared with baseline visits). The overall effect size of increased visits to grocery and pharmacy stores was relatively small, and largest in northwest England (*R* ratio 1·037 [1·022–1·052] per 5% weekly increase compared with baseline visits). The effect size for increased visits to transit stations was highest in London (1·084 [1·027–1·144] per 10% weekly increase compared with baseline visits). The largest effect size for increased visits to workplaces was observed in southwest England (1·101 [1·063–1·141] per 10% weekly increase compared with baseline visits).

**Table 2:** Association between six Google mobility metrics and *R* by UK region

	**Retail and recreation (per weekly increase of 15% of the baseline visits)**	**Grocery and pharmacy (per weekly increase of 5% of the baseline visits)**	**Transit stations (per weekly increase of 10% of the baseline visits)**	**Parks (per weekly increase of 40% of the baseline visits)**	**Workplaces (per weekly increase of 10% of the baseline visits)**	**Residential (per weekly increase of 5% of the baseline time length)**
Southeast England (65)	1·048 (1·030–1·067)	1·009 (0·999–1·019)	1·000 (0·985–1·016)	0·976 (0·964–0·987)	1·095 (1·073–1·119)	0·995 (0·958–1·032)
London (32)	1·036 (1·004–1·069)	0·989 (0·971–1·008)	1·084 (1·027–1·144)	0·972 (0·956–0·988)	1·084 (1·047–1·122)	0·996 (0·939–1·058)
Northwest England (34)	1·039 (1·011–1·067)	1·037 (1·022–1·052)	1·007 (0·987–1·028)	0·974 (0·957–0·990)	1·029 (0·994–1·065)	0·857 (0·799–0·920)
East England (40)	1·042 (1·018–1·067)	0·998 (0·985–1·011)	0·988 (0·970–1·006)	0·974 (0·960–0·988)	1·095 (1·064–1·127)	0·987 (0·938–1·039)
West Midlands (28)	1·062 (1·033–1·092)	1·022 (1·007–1·038)	1·021 (1·001–1·040)	0·964 (0·944–0·985)	1·049 (1·014–1·086)	0·930 (0·866–0·999)
Southwest England (29)	1·058 (1·030–1·087)	1·016 (1·000–1·031)	0·991 (0·975–1·006)	0·981 (0·963–1·000)	1·101 (1·063–1·141)	0·936 (0·876–1·001)
Yorkshire and the Humber (21)	1·084 (1·047–1·122)	1·009 (0·989–1·029)	1·008 (0·982–1·034)	0·944 (0·917–0·972)	1·039 (0·995–1·085)	0·920 (0·844–1·002)
East Midlands (32)	1·044 (1·021–1·069)	1·017 (1·005–1·029)	0·993 (0·978–1·008)	0·964 (0·949–0·979)	1·040 (1·013–1·068)	0·890 (0·841–0·941)
Northeast England (11)	1·113 (1·056–1·173)	0·997 (0·970–1·026)	1·006 (0·961–1·055)	0·961 (0·935–0·988)	0·983 (0·928–1·042)	0·885 (0·790–0·991)
Wales (16)	1·053 (1·013–1·094)	0·987 (0·966–1·008)	0·985 (0·951–1·020)	0·990 (0·966–1·013)	1·028 (0·989–1·069)	1·005 (0·935–1·080)
Scotland (17)	1·111 (1·043–1·183)	1·016 (0·989–1·044)	1·008 (0·964–1·053)	0·980 (0·954–1·007)	0·963 (0·921–1·007)	0·894 (0·810–0·987)
Northern Ireland (5)	1·090 (0·990–1·200)	1·011 (0·975–1·048)	0·979 (0·918–1·044)	0·986 (0·924–1·052)	0·987 (0·863–1·128)	0·836 (0·648–1·078)

Association (95% CI) is shown as weekly change in *R* (*R* ratio) associated with weekly difference in mobility metrics relative to the baseline level (baseline period Jan 3–Feb 6, 2020). *R*=reproduction number.

**Figure 3:**
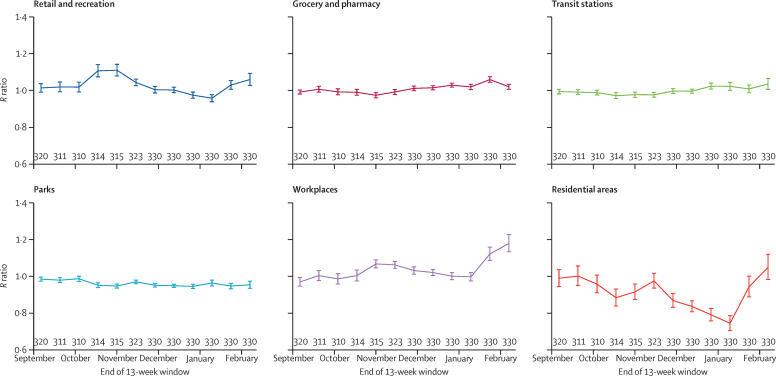
Change over time (13-week moving time window) in the association between six Google community mobility metrics and *R* The association is shown as weekly change in *R* (*R* ratio) associated with weekly difference in mobility metrics relative to the baseline level (baseline period Jan 3–Feb 6, 2020). X-axis numbers denote the local authorities included in the analysis. The 3 weeks of the Christmas and New Year festive period (Dec 21, 2020, to Jan 10, 2021) were excluded from the analysis. *R*=reproduction number.

The subgroup analysis stratified by *R* range showed that increased length of time spent at residential places could reduce *R* when *R* was 1·0–1·5, but not when *R* was 0·5–1·0, and that increased visits to grocery and pharmacy stores could increase *R* when *R* was 1·0–1·5, but not when *R* was 0·5–1·0 (table 3). The sensitivity analysis using the 13-week moving time window showed that for the first 6 weeks of 2021, there was an increasing trend in the effect on *R* of visits to retail and recreation places and workplaces, and time spent at residential areas (figure 3); our ad-hoc analyses stratified by region showed that this increasing trend was most pronounced in London, and the east and southeast of England (appendix pp 145–147).

**Table 3:** Association between six Google community mobility metrics and *R* across 330 local authorities in the UK, stratified by *R* range

	***R* ratio (95% CI)**
**Retail and recreation (per weekly increase of 15% of the baseline visits)**	
1·0–1·5	1·030 (1·007–1·053)
0·5–1·0	1·080 (1·065–1·095)
**Grocery and pharmacy (per weekly increase of 5% of the baseline visits)**	
1·0–1·5	1·024 (1·010–1·038)
0·5–1·0	1·000 (0·994–1·007)
**Transit stations (per weekly increase of 10% of the baseline visits)**	
1·0–1·5	0·998 (0·982–1·015)
0·5–1·0	1·001 (0·991–1·011)
**Parks (per weekly increase of 40% of the baseline visits)**	
1·0–1·5	0·963 (0·951–0·975)
0·5–1·0	0·978 (0·970–0·987)
**Workplaces (per weekly increase of 10% of the baseline visits)**	
1·0–1·5	1·041 (1·018–1·066)
0·5–1·0	1·025 (1·006–1·045)
**Residential (per weekly increase of 5% of the baseline time length)**	
1·0–1·5	0·877 (0·831–0·926)
0·5–1·0	0·995 (0·968–1·023)

Association is shown as weekly change in *R* (*R* ratio) associated with weekly difference in mobility metrics relative to the baseline level (baseline period Jan 3–Feb 6, 2020). *R*=reproduction number.

Meta-regression results that accounted for multiple characteristics of the local authorities confirmed the observed latitudinal gradients for visits to workplaces and time spent at residential areas (appendix p 16). Less deprived local authorities (ie, those with a higher prosperity score) had smaller increases in *R* following increased visits to retail and recreation places than did more deprived local authorities. A higher population density was associated with smaller effects on the association of *R* of increased visits to retail and recreation places, but larger effects of increased visits to transit stations.

## Discussion

In this study, we highlight visits to retail and recreation places as an important mobility metric associated with a substantially increased *R* of SARS-CoV-2; by contrast, increased visits to grocery and pharmacy stores were only associated with a small increase in *R*. We reported a smaller effect in the north than in the south of the UK in the increase of *R* associated with visits to workplaces. We reported that increased visits to parks and increased time spent at residential areas were found to decrease *R*. Increased visits to transit stations were not associated with an increased *R* at the national level; however, two independent sensitivity analyses both suggested that increased visits to transit stations were associated with a substantial increase in *R* in UK cities. Furthermore, we highlighted an increasing trend for the first 6 weeks of 2021, in the effect size for visits to retail and recreation places and visits to workplaces.

The overall findings of our study were qualitatively similar to previous studies done at the local level, as summarised in the appendix (pp 3–5), although most of these studies did not provide any effect sizes associated with their mobility metrics that allow for direct comparison. The mobility data in our model could account for a median of 42% (IQR 34–48) of the variations in transmission, which is similar to a global study^12^ that used country-level mobility data (median 48% [27–77]). Our findings suggest that increased visits to retail and recreation places, such as non-essentials shops, restaurants, and cinemas, might have contributed to the increased SARS-CoV-2 transmission nationwide; by comparison, essential visits to grocery and pharmacy stores were only associated with a small increase in transmission. Similar findings were reported by Deforche and colleagues,^13^ who showed that decreased retail and recreation mobility contributed most to reduced COVID-19 epidemics in 35 high-income countries. These findings might justify the need to focus on non-essential recreation activities to reduce transmission.

Several studies^5^, ^6^, ^14^, ^15^, ^16^, ^17^, ^18^ have highlighted the heterogeneities in mobility among various socioeconomic groups, including population density and income. One study in England^19^ found that population density could increase the effect of a composite mobility metric in the transmission of SARS-CoV-2. In the present study, higher population density was found to be associated with smaller effects of increased visits to retail and recreation places but larger effects of increased visits to transit stations. We showed that increased visits to public transit stations, such as bus, railway, and underground stations, increased *R* substantially in large cities, but not in the rest of the UK. This finding highlights the importance of public transportation in reducing transmission in cities. Additionally, the increase in *R* associated with visits to workplaces was smaller in northern regions of the UK than southern regions. We also found that the increase in *R* associated with increased visits to retail and recreation places was smaller in less deprived local authorities.

An increasing trend was observed in the first 6 weeks of 2021, with regard to the increase in *R* associated with visits to retail and recreation places and visits to workplaces. This trend was most pronounced in London and the east and southeast of England, where the incidence of the alpha variant was highest;^20^ the alpha variant was reported to have increased transmissibility^20^ and increased viral load.^21^ These characteristics suggest possible involvement of the alpha variant in the observed increasing trend. From April, 2021, the UK observed a rapid increase in the prevalence of the delta (B.1.617.2) variant, which was reported to have even higher transmissibility than the alpha variant according to Public Health England.^22^ Considering the findings of our present study, the effects of various increases in mobility might be further amplified by the predominance of the delta variant.

Our study had several strengths. First, we assessed six different mobility metrics rather than a single composite mobility metric. This approach could help refine the components of non-pharmaceutical interventions by restricting certain activities that are shown to increase *R* substantially or relaxing activities that have a smaller effect on transmission. Second, by pooling data from 330 local authorities across a period of more than 8 months, we were able to assess the association between community mobility and *R* with substantial statistical power, which allowed us to further explore the spatiotemporal heterogeneity in the association across the UK. Third, we incorporated a range of plausible *R* estimates in our model to reflect the statistical uncertainty around these estimates, rather than using the single point estimate.

However, our study also had several weaknesses. First, we acknowledge limitations of *R* as a measure of transmission of SARS-CoV-2, which have been discussed in detail previously.^23^ Specific to our study, the *R* estimate for an individual local authority was partly informed by the *R* of its corresponding region; this dependency could lead to smaller within-region heterogeneity that might reduce the variance of our region-specific meta-estimates, but this was unlikely to affect the overall findings. Second, our model captured a median of 42% of the total variations and thus might only capture a small component of the behavioural interventions. We could not capture use of face coverings, physical distancing, or hand washing, and we could not account for the number of peak visits to each location category since crowding could influence the transmission trajectory to a great extent.^17^ Third, we did not explicitly account for several time-variant factors that might have confounded our estimates, including seasonal factors (eg, temperature and humidity^17^, ^24^), testing practice, vaccination (rolled out in December, 2020, in the UK), and the emergence of the Alpha variant, which was reported to have spread quickly since November, 2020, to become the dominant variant in the UK, with increased transmissibility compared with other variants.^20^ Some of these factors, particularly the emergence of the alpha variant, might explain the observed change over time in the effect size of various mobility metrics in our analysis; unfortunately, we were limited by the scarcity of weekly data and data at the level of local authority on the alpha variant. Fourth, we were unable to account for cases moving between local authorities in our analysis, which could influence our estimates at the level of individual local authorities but probably not the overall meta-estimates. Fifth, the mobility metrics were based on Google services users who enabled their location history, so do not represent the whole population. However, a validation analysis for Google mobility data using contact surveys data in the UK indicated that these Google mobility metrics had been good predictors of contacts.^4^ Sixth, we were limited by the mobility data (eg, the absence of data that had origin–destination matrices) and were, therefore, unable to construct a mechanistic model that could help gain more insights into the interaction between mobility and *R*. Seventh, as this was a population-level study, our findings should not be interpreted as individual risks of transmission or infection.

To date, little is known about SARS-CoV-2 variants, interaction between SARS-CoV-2 and other seasonal respiratory viruses, and the role of COVID-19 vaccination in the interplay between mobility and transmission. Modelling studies from China^25^, ^26^ suggest that a vaccination programme (with a gradual increase in coverage) alone could not fully contain resurgence, highlighting the important role of mobility restrictions and physical distancing. As the pandemic continues to evolve, it will be necessary to repeat our analysis by including data from genomic surveillance of SARS-CoV-2 and broader respiratory viral surveillance to understand the effect of mobility on virus transmission after vaccination.

## Data sharing

All the data used in this study are publicly available and properly cited.

## Declaration of interests

YL reports grants from the Wellcome Trust, during the conduct of the study, and grants from WHO, outside the submitted work. HC reports grants from the Innovative Medicines Initiative, UK National Institute for Health Research, and Bill & Melinda Gates Foundation, and grants and personal fees from WHO and Sanofi, outside the submitted work. HN reports grants from the Innovative Medicines Initiative, WHO, the National Institute for Health Research, Sanofi, and the Foundation for Influenza Epidemiology; and personal fees from the Bill & Melinda Gates Foundation, Janssen, ReViral, AbbVie, Sanofi, and the Foundation for Influenza Epidemiology, outside the submitted work. All authors are members of the Respiratory Syncytial Virus Consortium in Europe, which has received funding from the Innovative Medicines Initiative 2 Joint Undertaking, under grant agreement number 116019. This Joint Undertaking receives support from the EU's Horizon 2020 Research and Innovation programme and the European Federation of Pharmaceutical Industries and Associations.
