# Supplementary material for: The association of community mobility with the time-varying reproduction number (R) of SARS-CoV-2: a modelling study across 330 local UK authorities
Source: Lancet Digit Health. 2021 Aug 31;3(10):e676–83. doi: 10.1016/S2589-7500(21)00144-8 (PMC8452268; doi:10.1016/S2589-7500(21)00144-8)
Supplement: Supplementary appendix [file mmc1.pdf]

THE LANCET
Digital Health

Supplementary appendix

This appendix formed part of the original submission and has been peer reviewed. We post it as supplied by the authors.

Supplement to: Li Y, Wang X, Campbell H, et al. The association of community mobility with the time-varying reproduction number (R) of SARS-CoV-2: a modelling study across 330 local UK authorities. *Lancet Digit Health* 2021; published online Aug 31. https://doi.org/10.1016/S2589-7500(21)00144-8.

Table of Contents

Text S1. Details of sampling R based on credible intervals 2

Table S1. Summary of publications on the association between community mobility and transmission of SARS-CoV-2 3

Table S2. Summary of datasets included in the analysis 6

Table S3. Summary of secondary analyses in comparison with the main analysis 7

Table S4. Association between six Google mobility metrics and R by individual local authority, as R ratio (95% CI) 8

Table S5. Meta-regression findings showing the modifiers of the association between six Google community mobility metrics and R, as the ratio of R ratios (95% CI) 16

Figure S1. Change over time (by week) in the mobility metrics and R on the local-authority level 17

Figure S2. Comparisons of findings from the main and sensitivity analyses 144

Figure S3. Change over time in the association between visits to retail and recreation places and R by region 145

Figure S4. Change over time in the association between visits to workplaces and R by region 146

Figure S5. Change over time in the association between time length spent at residential areas and R by region 147

STROBE checklist 148

References 150

Text S1. Details of sampling R based on credible intervals

Model estimates of R were reported originally as 30%, 60% and 90% credible intervals (CrI). To incorporate the uncertainty of R, we assumed that within each non-overlapping CrI, R followed a simple continuous uniform distribution. The joint probability density function is as follows:

$$f(R) = \begin{cases} \frac{1}{6} \times \frac{1}{CrI_{60.lower} - CrI_{90.lower}} & for\ R \in [CrI_{90.lower}, CrI_{60.lower}] \\ \frac{1}{6} \times \frac{1}{CrI_{30.lower} - CrI_{60.lower}} & for\ R \in [CrI_{60.lower}, CrI_{30.lower}] \\ \frac{2}{6} \times \frac{1}{CrI_{30.upper} - CrI_{30.lower}} & for\ R \in [CrI_{30.lower}, CrI_{30.upper}] \\ \frac{1}{6} \times \frac{1}{CrI_{60.upper} - CrI_{30.upper}} & for\ R \in [CrI_{30.upper}, CrI_{60.upper}] \\ \frac{1}{6} \times \frac{1}{CrI_{90.upper} - CrI_{60.upper}} & for\ R \in [CrI_{60.upper}, CrI_{90.upper}] \\ 0 & otherwise \end{cases}$$

Based on above, we used a two-step sampling method to generate 100 samples of R. As the first step, we randomly selected one interval out of the five intervals (with the interval between 30% lower CrI and 30% upper CrI having twice the probability of being selected than the rest), practically done by a simple random sampling among the numbers 1 to 6 (with the numbers 3 and 4 both representing the interval between 30% lower CrI and 30% upper CrI). Then we sampled 100 times from the selected interval based on uniform distribution. An example of the sampled R (Aberdeen city) is shown below,

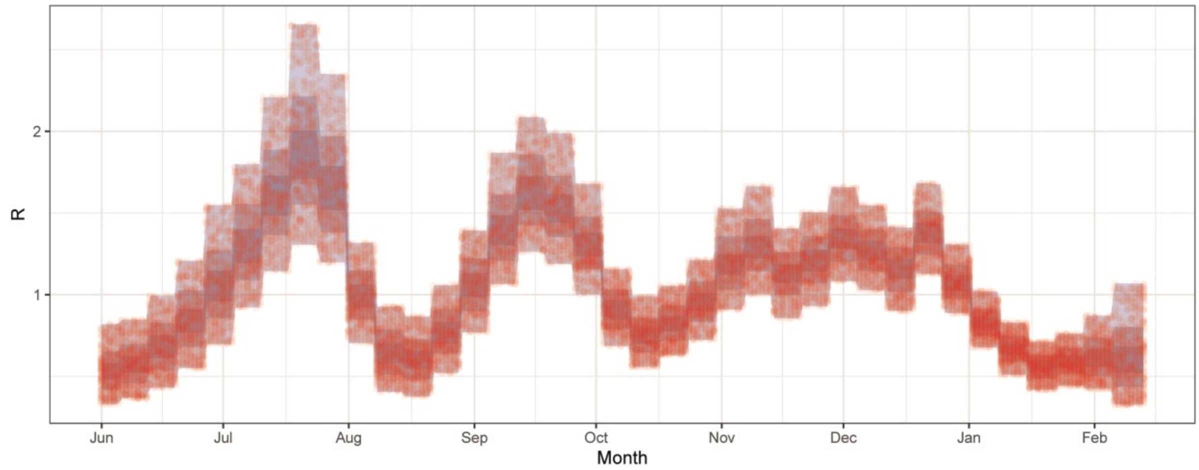

where shaded areas denote 30%, 60% and 90% CrIs and red points denote sampled R.

Table S1. Summary of publications on the association between community mobility and transmission of SARS-CoV-2

Index	Reference	Location and country	Mobility metric(s)	Main outcome measure	Main findings on the association between mobility and transmission
**1**	Badr et al. 2020[1]	Multiple counties, USA	Mobility ratio relative to a pre-COVID-19 baseline, derived from aggregated and anonymised cell phone data	Growth rate of COVID-19 cases	• Mobility patterns are strongly correlated with decreased COVID-19 case growth rates for the most affected counties in the USA, with Pearson correlation coefficients above 0.7 for 20 of the 25 counties evaluated.<br>• The effect of changes in mobility patterns on COVID-19 cases are not likely to be perceptible for 9–12 days, and potentially up to 3 weeks.
**2**	Brown et al. 2021[2]	Multiple provinces, Canada	Out-of-home mobility, derived from Google community mobility reports	Growth rate of COVID-19 cases	• **Each 10% increase in mobility is associated with 25% increase in growth rate of COVID-19 cases.**
**3**	Carlitz et al. 2021[3]	Multiple provinces, South Africa	Multiple Google community mobility metrics	Growth rate of COVID-19 cases	• Mobility reductions are significantly and negatively associated with COVID-19 growth rates two weeks later.
**4**	Cazelles et al. 2021[4]	Multiple regions in France, and Ireland	Multiple Google community mobility metrics	Reproduction number	• High positive correlation between trends in the transmission of SARS-CoV-2 and mobility.
**5**	Chen et al. 2021[5]	Wuhan, China	Intra-city mobility and inter-city mobility	COVID-19 cases	• Intra-city mobility is weakly negatively correlated with COVID-19 cases whereas inter-city mobility is positively correlated with COVID-19 cases
**6**	Da Silva et al. 2021[6]	Multiple cities, Brazil	Multiple Google community mobility metrics	COVID-19 cases	• Inconsistent correlation results between mobility metrics and COVID-19 cases are observed among different cities.
**7**	Díaz-Castro et al. 2021[7]	Multiple states, Mexico	Multiple Google community mobility metrics	COVID-19 cases doubling time	• The mobility in parks ($p < 0.01$) and the residential mobility ($p < 0.01$) are associated with doubling time of COVID-19 cases.
**8**	Fan et al. 2021[8]	Multiple counties, USA	Facebook co-location maps	Growth rate of COVID-19 cases	• The mitigation effects of co-location reduction appear in the growth of weekly new confirmed cases with one week of delay.
**9**	Gao et al. 2020[9]	Multiple states, USA	Daily travel distance and home dwell time derived from anonymous mobile phone location data	Growth rate of COVID-19 cases	• The correlation between the COVID-19 increase rate and travel distance decrease rate is –0.586 and that between COVID-19 increase rate and home dwell time increase rate was 0.526.
**10**	Gatalo et al. 2020[10]	Multiple counties, USA	Social distancing metrics created from phone mobility data provided by mobile phone service provider	Growth rate of COVID-19 cases	• Strong correlation results are observed between decreased mobility and reduced COVID-19 case growth between March 27 and April 20, 2020, but only weak correlation results are observed in later months, between April and July, 2020.
**11**	Glaeser et al. 2020[11]	Multiple cities, USA	Weekly number of visitors to a point of interest based on cell phone location data	Total / new cases per capita per zip code	• **Total cases per capita decrease by 20% for every ten percentage point fall in mobility.**
**12**	Iacus et al. 2020[12]	Multiple regions, France and Italy	Connectivity metric derived from mobile network operators	Excess COVID-19 deaths	• Mobility alone can explain up to 92% of the initial spread.<br>• Internal mobility is more important than mobility across provinces in explaining the spread.<br>• The time lag between changes in mobility and excess COVID-19 deaths is 14–20 days.
**13**	Jamshidi et al. 2020[13]	Multiple counties, USA	Mobility index and home index derived from anonymous cell phone location data	Growth rate of COVID-19 cases	• The mobility index (with the relative importance of 34.32%) is the highest contributing factor to the COVID-19 pandemic growth, followed by homestay (26.14%), population (23.86%), and urban density (13.03%).
**14**	Kajitani et al. 2021[14]	Multiple prefectures, Japan	Mobility metric derived from mobile phone service provider	Reproduction number	• **A 20%–35% reduction in weekly mobility is required to reduce R down to below 1.**

Index	Reference	Location and country	Mobility metric(s)	Main outcome measure	Main findings on the association between mobility and transmission
**15**	Kissler et al. 2020[15]	New York City, USA	The percent change in commuting-style movements between boroughs using data provided by Facebook's Data for Good program	Prevalence of COVID-19 among pregnant women	• Prevalence of COVID-19 is lowest in boroughs with the greatest reductions in morning movements out of and evening movements into the borough (Pearson R = −0.88 [−0.52, −0.99])
**16**	Kraemer et al. 2020[16]	Multiple provinces, China	Real-time travel history data between Wuhan and other provinces from internet services company, Baidu	COVID-19 cases	• The spatial distribution of COVID-19 cases in China is explained well by mobility data early on but this correlation drops after the implementation of control measures.
**17**	Lamb et al. 2020[17]	New York City, USA	Anonymized location data from cell phone visits to businesses obtained via SafeGraph	COVID-19 case positivity	• Increases in mobility are independently associated with decreased case positivity.
**18**	Leung et al. 2021[18]	Hong Kong, China	Number of transactions on Octopus cards (used for transportation and small retail payments)	Reproduction number	• R estimates are highly correlated with the number of Octopus transactions for transport: the Pearson's correlation coefficients are 0.62, 0.68, 0.80, and 0.76 for children, students, adults, and the elderly, respectively.
**19**	Li et al. 2020[19]	Multiple counties, USA	Multiple Google community mobility metrics	Growth rate of COVID-19 cases	• Mobility metrics have significant correlations with growth rate of COVID-19 cases 11 days later.
**20**	Monod et al. 2021[20]	Multiple states, USA	Age-specific human mobility trends derived from the Foursquare Labs Inc.	Reproduction number	• The high reproduction numbers from adults are linked both to rebounding mobility over the summer and elevated transmission risks per venue visit among adults aged 20 to 49.
**21**	Nakanishi et al. 2021[21]	Tokyo, Japan	Population volumes at 10 PM to midnight estimated from phone location data by LocationMind xPop.	Reproduction number	• R significantly increased 3 weeks after the night-time population volume increased.
**22**	Sartorius et al. 2021[22]	Multiple Middle Layer Super Output Areas (MSOA), England	The population movement provided by Cuebiq, which is a location intelligence and measurement platform	COVID-19 case	• A decrease in mobility at MSOA scale has had a significant reduction on caseloads.<br>• The change in mobility significantly interacts with population density within a MSOA, i.e. the impact of higher mobility is amplified by increasing population density.
**23**	Sehra et al. 2020[23]	Multiple counties, USA	Google workplace mobility metric	The slope of change in the 7-day rolling average for new COVID-19 case	• Counties with the lowest workplace activity had an average slope of change in new cases of 5.64 (4.60, 6.68) per 100 000 population as compared to 7.88 (6.92, 8.84) in the counties with the highest workplace activity (P<0.001).
**24**	Steiger et al. 2021[24]	Multiple districts, Germany	Multiple Google community mobility metrics	COVID-19 case	• **Daily reported case number increases by 0.11% for every 1% increase in retail/recreation mobility, by 0.33% for every 1% increase in workplace mobility, and by 0.26% for every 1% increase in transit stations mobility.**<br>• **Daily reported case number decreases by 0.23% for every 1% increase in grocery/pharmacy mobility, by 0.03% for every 1% increase in parks mobility, and by 0.97% for every 1% increase in residential stay.**
**25**	Unwin et al. 2020[25]	Multiple states, USA	Average mobility metrics and residential mobility derived from Google community mobility reports	Reproduction number	• **62% reduction in average mobility could reduce R by 37%**<br>• **33% increase in residential stay could reduce R by 5%**
**26**	Wang et al. 2020[26]	Multiple cities, China	Mobility scale index (MSI) from internet services company, Baidu	COVID-19 case	• Each 1 unit increase in daily MSI is significantly positively associated with daily confirmed cases of COVID-19 in all lag days and the correlation is strongest at the lag of 14 days.

Index	Reference	Location and country	Mobility metric(s)	Main outcome measure	Main findings on the association between mobility and transmission
**27**	Wang et al. 2020[27]	Multiple states, Australia	Multiple Google community mobility metrics	Growth rate of COVID-19 cases	• Inconsistent relationship between mobility and COVID-19 spread, which varies by date and by mobility type.
**28**	Xiong et al. 2020[28]	Multiple counties, USA	County-level daily mobility inflow	COVID-19 case	• **Every 10% increase in mobility inflow is associated with 2.34% increase in COVID-19 case one week later**
**29**	Yang et al. 2020[29]	Multiple cities, China	Intra-city travel intensity from internet services company, Baidu	Growth rate of COVID-19 cases	• Correlation coefficient between the average intracity travel intensity and the average growth rate of COVID-19 cases five days after is 0.86.
**30**	Younis et al. 2020[30]	Multiple counties, USA	Multiple Google community mobility metrics	Reproduction number	• Correlation coefficient between various mobility metrics and R ranges 0.66 to 0.89.
**31**	Zheng et al. 2021[31]	Multiple states, USA	Multiple Google community mobility metrics	COVID-19 case	• Correlation coefficient between various mobility metrics and R ranges 0.22 to 0.48.
**32**	Zhou et al. 2020[32]	Shenzhen, China	Anonymized location data from cell phone provided by cell phone service provider	Reproduction number	• **A 20% reduction in mobility is associated with a 25% reduction in transmissibility.**
**33**	Zhu et al. 2020[33]	Multiple cities, China	Intra-city travel intensity from internet services company, Baidu	COVID-19 case	• A unit increase in human mobility index is associated with a 6.45% increase in daily COVID-19 cases.

Findings that presented interpretable effect size of community mobility metrics on transmission of SARS-CoV-2 are highlighted **in bold**.

Studies were identified through a rapid literature search in PubMed using the combination of the following search terms: COVID-19 or SARS-CoV-2, mobility and transmission. The following criteria are applied for selection of the publications: 1) peer-reviewed studies (i.e. excluding pre-prints) published between 1-Jan-2020 and 18-Jun-2021 that assessed the association between real-world community mobility metrics and transmission of SARS-CoV-2 at local level; 2) studies are not included if they used mobility data only for mechanistic/statistical model fitting without assessing their specific effects on / association with transmission of SARS-CoV-2; 3) studies are not included if they only assessed the association between mobility restrictions and real-world mobility metrics (i.e. treating mobility metrics as the outcome; 4) studies are not included if they focused on a special group of population (e.g. health-care workers).

Table S2. Summary of datasets included in the analysis

Index	Name	Description
**1**	Google COVID-19 Community Mobility Reports[34,35]	The dataset consists of daily aggregated data on changes in visits and length of stay at different location categories compared to the baseline period, defined as 3rd Jan to 6th Feb, 2020, based on data from users who have opted-in to "location history" for their Google accounts.<br>Six mobility metrics are available in the Google community mobility dataset: visits to retail and recreation places (e.g. restaurants, cafes, shopping centres, theme parks, museums, libraries, and movie theatres), visits to grocery and pharmacy (e.g. grocery markets, food warehouses, farmers markets, specialty food shops, and pharmacies), visits to transit stations (e.g. bus, underground and train stations), visits to parks, visits to workplaces, and length of stay in residential places.
**2**	Apple Maps Mobility Trends Reports[36]	The dataset reports the relative volume of daily directions requests (categorised into walking, driving and public transit), compared to a pre-pandemic baseline volume on 13th Jan 2020 for 16 major cities of the UK: Belfast, Birmingham, Bradford, Bristol, Cardiff, Edinburgh, Glasgow, Leeds, Liverpool, London, Manchester, Newcastle upon Tyne, Nottingham, Portsmouth, Reading, and Sheffield.
**3**	COVID-19 Local, Imperial College London[37,38]	The dataset consists of daily Rt estimates for local authorities of the UK. The Rt for each local authority was estimated based on the reported COVID-19 cases and deaths by semi-mechanistic Bayesian models.[38] Rt was estimated based on projected infections that were back-calculated from the reported cases and deaths, through the two parameters infection ascertainment rate and infection fatality ratio, respectively. The time lags between infection and reporting of cases, and between infection and reporting of deaths were accounted for in the model so the R estimate was expected to reflect the instantaneous transmissibility of SARS-CoV-2. The local authority R data are published as 30%, 60% and 90% credible intervals and are available from 1st Jun 2020.
**4**	UK population density[39,40]	The dataset consists of population density data for the year 2019.
**5**	The Legatum Institute UK Prosperity Index (2016)[41]	The dataset consists of local-authority-specific prosperity index for the year 2016. The prosperity index, ranging between 0 and 1, defines and measures prosperity through seven pillars—economic quality, business environment, education, health, safety & security, social capital, and natural environment.[42] A higher prosperity index indicates less deprivation.
**6**	2011 UK Census data[43]	The dataset consists of all 2011 UK Census data. For the current analysis, we extracted the number and proportion of households with six or more people, and the number and proportion of BAME population per local authority.

BAME = Black, Asian and minority ethnic.

Table S3. Summary of secondary analyses in comparison with the main analysis

Index	Objective(s)	Individual-local-authority level analysis (Figure 1)	Meta-analysis across local authorities	Results available at
**1**	Assess whether use of public transportation was associated with increased R in cities	Two separate analyses, both limiting data to 16 major cities* of the UK:<br>1. same as the main analysis<br>2. using the Apple public transit requests in replace of the Google visits to transit stations	Same as the main analysis	Table 1
**2**	Exclude the mobility metric of residential areas since it had a different measure (i.e. time length) from other metrics (i.e. number of visits)	The independent variable residential stay removed from the main analysis	Same as the main analysis	Figure S2
**3**	Account for autocorrelation	The error term in the main analysis replaced with an autoregressive error term AR(1)	Same as the main analysis	Figure S2
**4**	Explore lagged effects	The R ratio of time i replaced with the R ratio of time i+1 while independent variables being the same as the main analysis	Same as the main analysis	Figure S2
**5**	Obtain results by region	Same as the main analysis	Separately by region	Table 2
**6**	Explore whether the observed association differed by R level	Stratified by R level (0.5–1.0 and 1.0–1.5)	Separately by R level	Table 3
**7**	Explore whether the observed association changed over time	Among 13-week moving time windows with the step of two weeks	Separately by time window	Figure 3
**8**	Explore whether the association was modified by population density, deprivation, proportion of BAME population, latitude and proportion of large household†(for the metric of time spent in residential areas only) of local authority	Same as the main analysis	Meta-regression using population density, prosperity score, proportion of BAME population, latitude and proportion of large household (for the metric of time length at residential areas only) as covariates.	Table S4

BAME = Black, Asian and minority ethnic.
*Belfast, Birmingham, Bradford, Bristol, Cardiff, Edinburgh, Glasgow, Leeds, Liverpool, London, Manchester, Newcastle upon Tyne, Nottingham, Portsmouth, Reading, and Sheffield.
†Defined as households with six or more members.

Table S4. Association between six Google mobility metrics and R by individual local authority, as R ratio (95% CI)

Local authority	Retail & recreation (per weekly increase of 15% of the baseline visits)	Grocery & pharmacy (per weekly increase of 5% of the baseline visits)	Transit stations (per weekly increase of 10% of the baseline visits)	Parks (per weekly increase of 40% of the baseline visits)	Workplaces (per weekly increase of 10% of the baseline visits)	Residential (per weekly increase of 5% of the baseline time length))
**Aberdeen City**	1·537 (0·889–2·659)	0·986 (0·826–1·177)	1·332 (0·879–2·017)	0·951 (0·788–1·148)	1·053 (0·698–1·590)	1·074 (0·595–1·939)
**Aberdeenshire**	0·800 (0·486–1·315)	1·084 (0·899–1·307)	0·942 (0·722–1·228)	1·022 (0·743–1·407)	0·986 (0·727–1·336)	0·737 (0·386–1·408)
**Allerdale**	1·007 (0·811–1·250)	1·064 (0·936–1·208)	0·965 (0·805–1·157)	0·965 (0·857–1·087)	1·115 (0·845–1·471)	0·866 (0·478–1·569)
**Amber Valley**	1·041 (0·862–1·257)	1·051 (0·943–1·171)	0·963 (0·842–1·102)	0·929 (0·813–1·061)	1·029 (0·891–1·190)	0·753 (0·511–1·111)
**Angus**	1·114 (0·828–1·499)	1·067 (0·927–1·227)	0·982 (0·858–1·123)	0·975 (0·895–1·062)	1·025 (0·878–1·195)	0·938 (0·625–1·407)
**Ards & North Down**	1·102 (0·910–1·334)	1·004 (0·946–1·067)	0·982 (0·904–1·068)	0·965 (0·797–1·168)	0·970 (0·754–1·248)	0·916 (0·561–1·498)
**Argyll & Bute**	1·115 (0·947–1·313)	0·997 (0·934–1·064)	0·986 (0·906–1·072)	0·972 (0·895–1·057)	1·009 (0·898–1·135)	0·930 (0·709–1·220)
**Arun**	1·102 (0·921–1·317)	1·012 (0·906–1·130)	1·041 (0·894–1·213)	0·984 (0·931–1·040)	1·174 (0·978–1·410)	1·090 (0·731–1·624)
**Ashfield**	1·060 (0·869–1·293)	1·011 (0·934–1·093)	0·988 (0·910–1·073)	0·938 (0·805–1·094)	1·051 (0·892–1·238)	0·807 (0·550–1·183)
**Ashford**	1·015 (0·884–1·165)	0·993 (0·883–1·117)	1·043 (0·850–1·279)	0·922 (0·750–1·134)	1·110 (0·889–1·386)	1·079 (0·692–1·684)
**Aylesbury Vale**	1·129 (0·921–1·385)	0·963 (0·873–1·062)	1·019 (0·888–1·170)	0·971 (0·849–1·110)	1·098 (0·936–1·287)	1·036 (0·647–1·659)
**Babergh**	1·090 (0·943–1·259)	1·040 (0·954–1·134)	0·946 (0·862–1·037)	0·953 (0·818–1·111)	1·162 (0·957–1·410)	0·978 (0·753–1·271)
**Barking & Dagenham**	1·075 (0·909–1·271)	0·982 (0·885–1·090)	1·063 (0·834–1·355)	0·964 (0·883–1·053)	1·082 (0·932–1·257)	1·026 (0·717–1·470)
**Barnet**	1·056 (0·887–1·257)	0·980 (0·871–1·102)	1·049 (0·742–1·484)	0·962 (0·878–1·054)	1·120 (0·920–1·365)	0·997 (0·670–1·484)
**Barnsley**	1·169 (0·912–1·500)	0·960 (0·839–1·099)	1·022 (0·868–1·204)	0·919 (0·776–1·089)	1·004 (0·760–1·328)	0·968 (0·626–1·494)
**Basildon**	1·042 (0·876–1·239)	0·947 (0·863–1·039)	1·006 (0·821–1·233)	0·955 (0·796–1·146)	1·011 (0·817–1·251)	1·010 (0·718–1·421)
**Basingstoke & Deane**	1·061 (0·803–1·403)	1·026 (0·933–1·128)	1·008 (0·825–1·232)	0·967 (0·776–1·204)	1·115 (0·900–1·381)	0·973 (0·528–1·793)
**Bassetlaw**	1·030 (0·912–1·164)	0·995 (0·949–1·043)	0·976 (0·905–1·053)	0·853 (0·755–0·963)	0·956 (0·832–1·099)	0·717 (0·507–1·015)
**Bath & North East Somerset**	1·083 (0·933–1·258)	1·015 (0·887–1·162)	0·937 (0·721–1·218)	0·977 (0·840–1·137)	1·172 (0·921–1·490)	0·939 (0·627–1·406)
**Bedford**	1·083 (0·915–1·282)	0·978 (0·911–1·050)	1·065 (0·882–1·286)	0·970 (0·877–1·073)	1·114 (0·962–1·291)	1·130 (0·826–1·546)
**Belfast**	1·028 (0·706–1·496)	1·015 (0·906–1·138)	0·981 (0·703–1·370)	0·922 (0·674–1·260)	1·029 (0·734–1·444)	0·762 (0·426–1·361)
**Bexley**	1·146 (0·960–1·367)	0·995 (0·892–1·109)	1·076 (0·833–1·390)	0·944 (0·833–1·069)	1·035 (0·805–1·331)	1·055 (0·723–1·537)
**Birmingham**	1·193 (1·009–1·410)	1·016 (0·926–1·114)	1·030 (0·798–1·331)	0·978 (0·857–1·115)	1·047 (0·880–1·245)	1·023 (0·671–1·559)
**Blaby**	1·082 (0·994–1·178)	1·012 (0·968–1·058)	0·975 (0·841–1·130)	0·990 (0·946–1·036)	0·983 (0·901–1·073)	1·002 (0·777–1·293)
**Blackpool**	1·008 (0·936–1·085)	1·040 (0·962–1·123)	0·962 (0·881–1·052)	1·001 (0·950–1·055)	1·059 (0·815–1·377)	0·783 (0·468–1·308)
**Bolsover**	1·031 (0·946–1·123)	0·999 (0·917–1·088)	0·996 (0·930–1·067)	0·974 (0·895–1·060)	1·060 (0·895–1·255)	0·831 (0·587–1·178)
**Bolton**	1·178 (0·963–1·442)	1·016 (0·917–1·125)	0·987 (0·789–1·235)	1·036 (0·881–1·219)	1·180 (0·954–1·458)	1·068 (0·662–1·722)
**Bournemouth, Christchurch & Poole**	1·270 (1·000–1·612)	1·030 (0·886–1·197)	0·889 (0·659–1·200)	1·027 (0·943–1·118)	1·282 (0·968–1·697)	1·123 (0·661–1·910)
**Bracknell Forest**	1·023 (0·888–1·178)	0·988 (0·875–1·115)	1·000 (0·894–1·119)	0·990 (0·923–1·063)	1·103 (0·951–1·279)	0·986 (0·790–1·230)
**Bradford**	1·098 (0·891–1·353)	1·004 (0·919–1·096)	1·108 (0·919–1·337)	0·961 (0·860–1·073)	0·986 (0·841–1·157)	1·032 (0·670–1·590)
**Breckland**	1·055 (0·849–1·310)	1·029 (0·933–1·136)	1·023 (0·893–1·172)	0·908 (0·790–1·044)	1·113 (0·881–1·407)	0·974 (0·585–1·622)
**Brent**	1·180 (1·009–1·379)	0·998 (0·901–1·106)	0·896 (0·652–1·231)	1·005 (0·941–1·072)	1·090 (0·883–1·347)	1·002 (0·707–1·422)
**Brentwood**	1·052 (0·900–1·229)	0·958 (0·889–1·033)	1·027 (0·899–1·172)	0·985 (0·844–1·150)	1·181 (0·965–1·446)	1·070 (0·832–1·376)
**Bridgend**	1·274 (0·952–1·705)	0·989 (0·875–1·118)	0·886 (0·706–1·112)	1·002 (0·932–1·078)	1·039 (0·759–1·421)	1·083 (0·603–1·944)
**Brighton & Hove**	1·106 (0·843–1·449)	1·037 (0·861–1·248)	0·964 (0·676–1·376)	0·988 (0·855–1·142)	1·238 (0·903–1·697)	1·084 (0·574–2·047)
**Bristol, City of**	1·334 (0·835–2·129)	1·055 (0·845–1·318)	0·999 (0·572–1·745)	1·005 (0·810–1·247)	1·283 (0·852–1·932)	1·188 (0·564–2·502)
**Broadland**	1·080 (0·964–1·209)	1·025 (0·954–1·100)	0·967 (0·882–1·061)	0·954 (0·832–1·094)	1·108 (0·929–1·320)	1·034 (0·801–1·334)
**Bromley**	1·082 (0·937–1·249)	0·976 (0·907–1·050)	1·033 (0·844–1·264)	0·966 (0·846–1·103)	1·095 (0·904–1·326)	1·042 (0·750–1·446)
**Bromsgrove**	1·118 (1·004–1·245)	1·007 (0·950–1·068)	1·046 (0·975–1·122)	0·949 (0·871–1·034)	1·111 (0·963–1·282)	1·138 (0·816–1·587)

Local authority	Retail & recreation (per weekly increase of 15% of the baseline visits)	Grocery & pharmacy (per weekly increase of 5% of the baseline visits)	Transit stations (per weekly increase of 10% of the baseline visits)	Parks (per weekly increase of 40% of the baseline visits)	Workplaces (per weekly increase of 10% of the baseline visits)	Residential (per weekly increase of 5% of the baseline time length))
**Broxbourne**	1·033 (0·897–1·190)	0·981 (0·914–1·051)	1·017 (0·902–1·147)	0·964 (0·846–1·099)	1·059 (0·905–1·241)	0·974 (0·747–1·269)
**Broxtowe**	0·992 (0·834–1·180)	1·039 (0·935–1·155)	1·059 (0·878–1·277)	0·982 (0·777–1·241)	1·075 (0·780–1·481)	0·890 (0·547–1·447)
**Burnley**	1·299 (1·036–1·628)	0·980 (0·908–1·057)	1·204 (0·948–1·529)	0·984 (0·881–1·099)	0·896 (0·699–1·149)	1·367 (0·894–2·091)
**Bury**	1·025 (0·864–1·215)	1·045 (0·966–1·130)	1·020 (0·915–1·136)	0·982 (0·900–1·072)	1·004 (0·849–1·188)	0·846 (0·598–1·197)
**Caerphilly**	1·300 (0·912–1·853)	0·977 (0·812–1·177)	0·742 (0·500–1·099)	1·028 (0·890–1·188)	1·151 (0·758–1·746)	0·906 (0·474–1·729)
**Calderdale**	1·056 (0·894–1·247)	1·026 (0·949–1·110)	1·008 (0·837–1·214)	0·918 (0·746–1·128)	1·032 (0·865–1·231)	0·933 (0·590–1·474)
**Cambridge**	0·837 (0·614–1·142)	1·100 (0·983–1·231)	1·127 (0·734–1·732)	0·834 (0·696–0·999)	1·012 (0·792–1·293)	0·687 (0·400–1·181)
**Camden**	0·882 (0·596–1·307)	0·908 (0·769–1·072)	1·561 (0·911–2·676)	0·976 (0·822–1·159)	1·006 (0·847–1·195)	1·018 (0·724–1·431)
**Cannock Chase**	1·051 (0·862–1·280)	1·021 (0·946–1·103)	1·044 (0·974–1·120)	0·951 (0·849–1·066)	1·120 (0·960–1·308)	0·992 (0·718–1·372)
**Canterbury**	1·161 (0·988–1·365)	0·945 (0·854–1·046)	0·992 (0·854–1·152)	1·019 (0·955–1·087)	1·142 (0·949–1·375)	1·267 (0·905–1·775)
**Cardiff**	1·146 (0·787–1·668)	0·906 (0·771–1·063)	1·105 (0·689–1·772)	1·078 (0·810–1·434)	1·184 (0·868–1·613)	1·174 (0·660–2·088)
**Carlisle**	0·959 (0·765–1·202)	1·130 (0·980–1·304)	0·987 (0·785–1·241)	0·829 (0·606–1·134)	1·054 (0·707–1·570)	0·730 (0·398–1·341)
**Carmarthenshire**	1·121 (0·915–1·374)	0·953 (0·880–1·033)	0·933 (0·808–1·077)	1·037 (0·916–1·174)	1·016 (0·847–1·220)	0·900 (0·602–1·345)
**Causeway Coast & Glens**	1·041 (0·858–1·264)	1·035 (0·963–1·112)	0·961 (0·816–1·131)	0·989 (0·903–1·083)	1·012 (0·777–1·318)	0·861 (0·499–1·485)
**Central Bedfordshire**	1·059 (0·851–1·318)	0·966 (0·871–1·072)	0·996 (0·826–1·200)	0·950 (0·851–1·061)	1·057 (0·873–1·279)	0·948 (0·593–1·516)
**Charnwood**	0·945 (0·728–1·227)	1·070 (0·962–1·189)	0·976 (0·818–1·164)	0·941 (0·845–1·049)	0·975 (0·809–1·176)	0·739 (0·462–1·180)
**Chelmsford**	1·058 (0·884–1·267)	0·968 (0·862–1·087)	0·992 (0·872–1·129)	0·965 (0·847–1·099)	1·087 (0·894–1·323)	0·981 (0·638–1·507)
**Cheltenham**	1·070 (0·951–1·204)	1·003 (0·943–1·067)	1·001 (0·948–1·058)	0·989 (0·912–1·072)	1·062 (0·916–1·230)	0·959 (0·744–1·237)
**Cherwell**	0·953 (0·773–1·175)	1·036 (0·935–1·148)	1·024 (0·849–1·234)	0·839 (0·619–1·136)	1·138 (0·901–1·436)	0·778 (0·434–1·392)
**Cheshire East**	1·049 (0·911–1·208)	1·006 (0·929–1·089)	1·115 (0·955–1·302)	0·973 (0·869–1·089)	1·012 (0·859–1·192)	0·927 (0·635–1·352)
**Cheshire West & Chester**	1·152 (0·972–1·366)	1·024 (0·925–1·133)	1·001 (0·873–1·147)	1·017 (0·820–1·262)	1·073 (0·813–1·416)	1·037 (0·625–1·718)
**Chesterfield**	1·005 (0·860–1·175)	1·035 (0·953–1·123)	1·077 (0·908–1·278)	0·932 (0·852–1·020)	1·104 (0·936–1·303)	0·872 (0·613–1·239)
**Chichester**	1·030 (0·878–1·208)	1·042 (0·956–1·135)	1·003 (0·870–1·156)	0·931 (0·778–1·113)	1·161 (0·943–1·430)	0·979 (0·630–1·523)
**Chiltern**	1·069 (0·946–1·208)	0·990 (0·919–1·067)	1·022 (0·884–1·182)	0·955 (0·902–1·012)	1·092 (0·964–1·238)	0·984 (0·785–1·233)
**Chorley**	0·998 (0·892–1·118)	1·030 (0·965–1·100)	1·022 (0·960–1·088)	0·971 (0·911–1·036)	1·039 (0·886–1·218)	0·832 (0·631–1·098)
**City of Edinburgh**	1·000 (0·643–1·555)	0·963 (0·820–1·132)	1·125 (0·713–1·776)	0·993 (0·846–1·166)	0·891 (0·677–1·172)	0·774 (0·502–1·193)
**Colchester**	0·976 (0·815–1·169)	1·047 (0·935–1·171)	1·024 (0·787–1·332)	0·930 (0·795–1·088)	1·065 (0·769–1·474)	0·896 (0·554–1·449)
**Conwy**	1·032 (0·899–1·186)	1·014 (0·948–1·084)	0·977 (0·905–1·055)	1·002 (0·939–1·069)	1·006 (0·872–1·162)	0·949 (0·711–1·266)
**Copeland**	1·058 (0·803–1·393)	1·039 (0·926–1·164)	1·058 (0·967–1·158)	0·943 (0·826–1·077)	0·997 (0·724–1·373)	0·790 (0·445–1·405)
**Cornwall**	1·187 (0·937–1·505)	0·969 (0·786–1·194)	1·000 (0·739–1·352)	0·906 (0·796–1·031)	0·910 (0·673–1·232)	0·586 (0·301–1·142)
**Cotswold**	1·003 (0·870–1·156)	1·064 (0·958–1·181)	0·977 (0·865–1·103)	0·911 (0·756–1·099)	1·143 (0·954–1·369)	0·854 (0·564–1·295)
**County Durham**	1·378 (1·006–1·888)	1·015 (0·850–1·212)	0·916 (0·653–1·284)	0·951 (0·710–1·273)	0·969 (0·665–1·412)	0·935 (0·411–2·131)
**Coventry**	1·152 (0·942–1·408)	1·046 (0·949–1·152)	1·012 (0·791–1·294)	0·982 (0·878–1·100)	1·057 (0·879–1·271)	0·960 (0·624–1·476)
**Craven**	1·075 (0·973–1·187)	1·024 (0·940–1·115)	1·006 (0·936–1·081)	0·947 (0·849–1·056)	0·996 (0·846–1·172)	0·964 (0·712–1·305)
**Crawley**	1·034 (0·826–1·294)	1·046 (0·920–1·189)	1·041 (0·847–1·279)	0·997 (0·928–1·072)	1·246 (0·970–1·602)	0·991 (0·657–1·495)
**Croydon**	1·010 (0·858–1·188)	0·994 (0·910–1·085)	1·162 (0·776–1·740)	0·960 (0·870–1·060)	1·087 (0·892–1·325)	1·011 (0·746–1·372)
**Dacorum**	1·022 (0·870–1·199)	0·994 (0·927–1·066)	0·978 (0·890–1·075)	0·956 (0·843–1·085)	1·071 (0·936–1·226)	0·957 (0·691–1·325)
**Darlington**	1·036 (0·923–1·162)	0·967 (0·894–1·046)	1·161 (0·968–1·392)	0·981 (0·931–1·034)	0·991 (0·880–1·116)	0·965 (0·767–1·215)
**Dartford**	1·056 (0·940–1·187)	1·003 (0·957–1·052)	0·999 (0·854–1·169)	1·000 (0·954–1·049)	1·161 (0·947–1·425)	1·065 (0·833–1·362)
**Daventry**	1·059 (0·920–1·220)	1·012 (0·929–1·102)	1·055 (0·940–1·185)	0·883 (0·763–1·021)	1·077 (0·919–1·263)	0·953 (0·707–1·285)
**Denbighshire**	1·016 (0·859–1·202)	1·009 (0·924–1·102)	1·104 (0·900–1·354)	0·945 (0·860–1·039)	0·968 (0·834–1·124)	1·024 (0·783–1·339)
**Derby**	1·084 (0·879–1·337)	1·049 (0·939–1·172)	1·096 (0·847–1·417)	0·982 (0·863–1·118)	1·074 (0·855–1·350)	0·970 (0·638–1·473)

Local authority	Retail & recreation (per weekly increase of 15% of the baseline visits)	Grocery & pharmacy (per weekly increase of 5% of the baseline visits)	Transit stations (per weekly increase of 10% of the baseline visits)	Parks (per weekly increase of 40% of the baseline visits)	Workplaces (per weekly increase of 10% of the baseline visits)	Residential (per weekly increase of 5% of the baseline time length))
**Derbyshire Dales**	1·031 (0·966–1·101)	1·016 (0·972–1·061)	0·990 (0·948–1·033)	0·885 (0·756–1·035)	1·033 (0·915–1·166)	0·818 (0·662–1·010)
**Derry City & Strabane**	1·161 (0·883–1·527)	0·981 (0·867–1·110)	0·962 (0·795–1·163)	0·939 (0·606–1·455)	0·981 (0·653–1·473)	0·685 (0·345–1·359)
**Doncaster**	1·004 (0·819–1·231)	1·037 (0·918–1·171)	0·931 (0·719–1·206)	0·885 (0·740–1·059)	0·868 (0·633–1·191)	0·589 (0·329–1·052)
**Dorset**	1·055 (0·896–1·243)	1·030 (0·920–1·154)	0·974 (0·872–1·088)	0·988 (0·870–1·121)	1·246 (0·891–1·743)	0·985 (0·542–1·789)
**Dover**	1·041 (0·947–1·144)	1·011 (0·940–1·086)	0·923 (0·865–0·984)	0·992 (0·920–1·069)	1·048 (0·917–1·197)	1·025 (0·768–1·368)
**Dudley**	1·128 (0·898–1·417)	1·032 (0·946–1·125)	0·995 (0·917–1·079)	0·969 (0·723–1·298)	1·107 (0·875–1·400)	0·924 (0·544–1·569)
**Dumfries & Galloway**	1·215 (0·680–2·173)	1·024 (0·865–1·212)	1·067 (0·857–1·327)	0·803 (0·581–1·111)	0·976 (0·661–1·440)	0·865 (0·369–2·027)
**Dundee City**	1·252 (0·978–1·603)	0·964 (0·865–1·075)	1·044 (0·865–1·259)	1·003 (0·898–1·120)	0·978 (0·809–1·182)	0·958 (0·692–1·325)
**Ealing**	1·067 (0·882–1·291)	1·024 (0·913–1·149)	0·844 (0·544–1·311)	0·964 (0·884–1·051)	1·204 (0·953–1·521)	0·856 (0·555–1·321)
**East Cambridgeshire**	1·055 (0·943–1·180)	0·993 (0·944–1·045)	0·985 (0·883–1·099)	0·949 (0·851–1·058)	1·068 (0·936–1·219)	0·999 (0·783–1·274)
**East Devon**	1·020 (0·922–1·129)	1·018 (0·962–1·076)	0·992 (0·922–1·067)	1·007 (0·930–1·089)	1·171 (0·976–1·406)	1·111 (0·786–1·570)
**East Hampshire**	1·048 (0·905–1·215)	1·031 (0·932–1·140)	1·014 (0·917–1·120)	0·902 (0·790–1·030)	1·010 (0·876–1·164)	0·837 (0·642–1·092)
**East Hertfordshire**	1·065 (0·936–1·211)	1·033 (0·955–1·117)	1·028 (0·965–1·094)	0·951 (0·851–1·064)	1·048 (0·904–1·215)	1·021 (0·780–1·336)
**East Lindsey**	1·040 (0·846–1·278)	0·993 (0·931–1·059)	0·969 (0·855–1·098)	0·989 (0·851–1·148)	1·089 (0·922–1·286)	0·834 (0·515–1·351)
**East Lothian**	1·213 (0·951–1·547)	0·975 (0·878–1·083)	1·024 (0·845–1·242)	0·991 (0·925–1·062)	1·029 (0·901–1·175)	1·077 (0·709–1·634)
**East Northamptonshire**	1·070 (0·967–1·184)	0·995 (0·946–1·047)	0·991 (0·951–1·033)	0·958 (0·879–1·044)	1·121 (0·985–1·276)	1·045 (0·820–1·333)
**East Riding of Yorkshire**	1·163 (1·018–1·327)	1·023 (0·943–1·110)	0·981 (0·903–1·067)	0·936 (0·866–1·012)	0·991 (0·831–1·181)	0·872 (0·600–1·269)
**East Staffordshire**	1·102 (0·935–1·298)	1·026 (0·957–1·100)	1·018 (0·903–1·148)	0·973 (0·905–1·046)	1·061 (0·917–1·228)	1·009 (0·706–1·441)
**East Suffolk**	1·040 (0·914–1·183)	1·050 (0·974–1·133)	0·945 (0·862–1·036)	1·014 (0·912–1·128)	1·146 (0·974–1·348)	1·024 (0·748–1·403)
**Eastbourne**	1·014 (0·790–1·302)	1·046 (0·929–1·177)	1·008 (0·808–1·257)	0·991 (0·901–1·091)	1·231 (0·970–1·561)	1·178 (0·623–2·226)
**Eastleigh**	1·062 (0·806–1·398)	1·054 (0·924–1·202)	0·991 (0·813–1·209)	0·990 (0·815–1·201)	1·249 (0·999–1·562)	0·950 (0·539–1·673)
**Eden**	1·002 (0·834–1·204)	1·060 (0·996–1·128)	0·917 (0·811–1·036)	0·990 (0·858–1·142)	1·161 (0·956–1·409)	0·936 (0·694–1·261)
**Elmbridge**	1·046 (0·889–1·230)	1·001 (0·929–1·079)	1·055 (0·933–1·192)	0·958 (0·830–1·104)	1·110 (0·915–1·347)	1·046 (0·757–1·445)
**Enfield**	1·053 (0·859–1·290)	0·975 (0·868–1·095)	0·990 (0·689–1·421)	0·942 (0·829–1·070)	1·069 (0·839–1·363)	0·914 (0·591–1·416)
**Epping Forest**	1·091 (0·906–1·313)	0·921 (0·844–1·004)	1·121 (0·916–1·372)	0·985 (0·922–1·052)	1·116 (0·946–1·317)	1·115 (0·771–1·613)
**Epsom & Ewell**	1·026 (0·913–1·154)	1·008 (0·957–1·062)	1·004 (0·906–1·113)	0·961 (0·881–1·048)	1·090 (0·981–1·212)	0·968 (0·823–1·138)
**Erewash**	1·034 (0·878–1·218)	1·063 (0·988–1·143)	0·983 (0·905–1·067)	0·929 (0·831–1·038)	1·082 (0·932–1·256)	0·804 (0·563–1·147)
**Exeter**	1·236 (0·767–1·993)	1·095 (0·823–1·456)	1·111 (0·786–1·569)	1·339 (0·874–2·050)	0·953 (0·522–1·741)	1·668 (0·545–5·106)
**Falkirk**	1·212 (0·976–1·506)	1·008 (0·925–1·100)	1·017 (0·873–1·185)	0·982 (0·920–1·049)	0·874 (0·749–1·019)	0·798 (0·581–1·097)
**Fareham**	1·036 (0·857–1·251)	1·037 (0·950–1·132)	1·067 (0·888–1·282)	0·957 (0·870–1·052)	1·127 (0·923–1·375)	0·995 (0·654–1·513)
**Fenland**	1·013 (0·894–1·148)	1·007 (0·957–1·060)	0·938 (0·887–0·990)	0·975 (0·853–1·114)	1·134 (0·984–1·307)	0·872 (0·663–1·148)
**Fife**	1·159 (0·905–1·484)	1·063 (0·933–1·212)	1·012 (0·806–1·269)	0·969 (0·850–1·104)	0·902 (0·755–1·078)	0·823 (0·555–1·220)
**Folkestone & Hythe**	1·050 (0·951–1·160)	1·011 (0·940–1·087)	0·959 (0·885–1·039)	1·011 (0·963–1·060)	1·185 (1·014–1·386)	1·288 (0·972–1·708)
**Forest of Dean**	1·049 (0·942–1·170)	1·018 (0·951–1·089)	0·973 (0·889–1·066)	0·989 (0·858–1·139)	1·019 (0·853–1·217)	0·877 (0·642–1·198)
**Fylde**	1·044 (0·960–1·135)	1·017 (0·966–1·070)	0·998 (0·938–1·062)	0·971 (0·915–1·030)	1·055 (0·911–1·221)	0·867 (0·668–1·125)
**Gateshead**	1·175 (1·010–1·368)	0·993 (0·874–1·129)	0·991 (0·816–1·205)	0·944 (0·809–1·101)	1·022 (0·736–1·419)	0·826 (0·543–1·256)
**Gedling**	1·127 (0·949–1·340)	1·006 (0·929–1·088)	1·059 (0·940–1·194)	0·963 (0·918–1·011)	1·154 (0·940–1·416)	0·992 (0·664–1·480)
**Glasgow City**	0·950 (0·716–1·260)	0·966 (0·855–1·092)	1·270 (0·785–2·052)	0·996 (0·885–1·122)	0·880 (0·734–1·056)	0·870 (0·567–1·334)
**Gloucester**	1·065 (0·934–1·215)	0·984 (0·896–1·080)	0·957 (0·840–1·090)	0·979 (0·910–1·053)	1·065 (0·881–1·286)	0·885 (0·642–1·219)
**Gosport**	1·000 (0·869–1·152)	1·044 (0·963–1·131)	0·980 (0·863–1·112)	0·972 (0·901–1·048)	1·103 (0·952–1·277)	0·887 (0·634–1·241)
**Gravesham**	1·096 (0·967–1·242)	1·012 (0·941–1·089)	1·041 (0·917–1·182)	0·907 (0·772–1·065)	1·027 (0·890–1·186)	1·068 (0·831–1·372)
**Great Yarmouth**	0·996 (0·834–1·190)	1·024 (0·918–1·143)	1·004 (0·921–1·096)	1·003 (0·908–1·109)	1·163 (0·908–1·490)	1·069 (0·731–1·564)

Local authority	Retail & recreation (per weekly increase of 15% of the baseline visits)	Grocery & pharmacy (per weekly increase of 5% of the baseline visits)	Transit stations (per weekly increase of 10% of the baseline visits)	Parks (per weekly increase of 40% of the baseline visits)	Workplaces (per weekly increase of 10% of the baseline visits)	Residential (per weekly increase of 5% of the baseline time length))
**Greenwich**	1·079 (0·949–1·226)	0·976 (0·879–1·084)	1·128 (0·806–1·578)	0·965 (0·852–1·094)	1·060 (0·855–1·314)	1·072 (0·743–1·546)
**Guildford**	1·025 (0·916–1·146)	0·989 (0·943–1·038)	1·058 (0·932–1·201)	0·961 (0·830–1·113)	1·036 (0·918–1·169)	0·966 (0·776–1·202)
**Gwynedd**	1·022 (0·907–1·153)	1·031 (0·947–1·122)	1·038 (0·892–1·209)	0·937 (0·791–1·111)	0·950 (0·776–1·163)	0·933 (0·687–1·268)
**Hackney**	0·861 (0·575–1·290)	1·046 (0·886–1·235)	1·545 (0·889–2·683)	0·967 (0·834–1·122)	0·975 (0·671–1·416)	1·040 (0·590–1·831)
**Halton**	1·091 (0·835–1·427)	1·039 (0·905–1·193)	1·139 (0·923–1·405)	1·003 (0·931–1·081)	0·860 (0·644–1·149)	0·826 (0·500–1·365)
**Hambleton**	1·038 (0·860–1·254)	1·063 (0·964–1·172)	0·974 (0·807–1·177)	0·892 (0·755–1·054)	1·112 (0·915–1·351)	0·763 (0·511–1·138)
**Hammersmith & Fulham**	1·016 (0·825–1·251)	0·960 (0·837–1·099)	1·177 (0·812–1·707)	1·016 (0·880–1·173)	1·207 (0·917–1·590)	1·079 (0·763–1·525)
**Harborough**	1·042 (0·927–1·172)	1·023 (0·960–1·091)	1·002 (0·897–1·120)	0·981 (0·918–1·050)	0·993 (0·904–1·091)	0·920 (0·736–1·150)
**Haringey**	0·955 (0·769–1·186)	0·978 (0·848–1·129)	1·154 (0·750–1·777)	0·981 (0·886–1·086)	1·138 (0·910–1·423)	1·038 (0·722–1·493)
**Harlow**	1·024 (0·868–1·208)	1·004 (0·924–1·092)	0·967 (0·868–1·078)	0·979 (0·933–1·028)	1·057 (0·922–1·212)	0·901 (0·697–1·165)
**Harrogate**	1·052 (0·870–1·272)	0·991 (0·897–1·095)	1·180 (0·967–1·439)	0·905 (0·721–1·134)	1·126 (0·868–1·460)	1·065 (0·625–1·817)
**Harrow**	1·057 (0·938–1·191)	0·988 (0·916–1·065)	0·939 (0·712–1·238)	0·987 (0·935–1·041)	1·124 (0·942–1·340)	0·950 (0·707–1·277)
**Hart**	1·007 (0·871–1·164)	1·005 (0·956–1·056)	1·050 (0·956–1·153)	0·960 (0·835–1·103)	1·156 (0·976–1·368)	1·045 (0·760–1·438)
**Hastings**	1·145 (0·890–1·473)	0·924 (0·793–1·075)	0·985 (0·642–1·511)	1·000 (0·888–1·126)	1·110 (0·833–1·480)	1·141 (0·656–1·983)
**Havant**	1·129 (0·908–1·403)	1·026 (0·937–1·123)	1·007 (0·847–1·197)	0·984 (0·901–1·076)	1·172 (0·980–1·403)	1·026 (0·579–1·817)
**Havering**	1·067 (0·877–1·298)	0·981 (0·870–1·106)	1·108 (0·822–1·494)	0·948 (0·816–1·101)	1·041 (0·832–1·302)	1·009 (0·687–1·483)
**Herefordshire, County of**	0·985 (0·779–1·245)	1·126 (0·980–1·293)	0·966 (0·835–1·118)	0·818 (0·633–1·057)	0·991 (0·750–1·309)	0·684 (0·379–1·233)
**Hertsmere**	0·957 (0·769–1·191)	0·981 (0·918–1·049)	0·979 (0·818–1·172)	0·935 (0·831–1·052)	1·053 (0·866–1·282)	0·837 (0·590–1·187)
**High Peak**	1·040 (0·920–1·174)	1·046 (0·974–1·123)	0·970 (0·882–1·068)	0·986 (0·950–1·023)	1·102 (0·922–1·317)	0·908 (0·646–1·275)
**Highland**	0·935 (0·653–1·339)	1·132 (0·978–1·311)	1·079 (0·761–1·529)	0·848 (0·668–1·075)	0·865 (0·673–1·111)	0·731 (0·407–1·313)
**Hillingdon**	1·080 (0·868–1·344)	1·001 (0·912–1·099)	0·982 (0·783–1·232)	0·964 (0·873–1·065)	1·066 (0·899–1·264)	0·923 (0·589–1·446)
**Hinckley & Bosworth**	1·044 (0·871–1·251)	1·029 (0·956–1·108)	0·968 (0·850–1·103)	0·971 (0·875–1·078)	0·978 (0·867–1·103)	0·845 (0·630–1·132)
**Horsham**	1·086 (0·921–1·280)	1·005 (0·915–1·105)	1·039 (0·871–1·239)	0·932 (0·801–1·085)	1·017 (0·861–1·200)	0·943 (0·670–1·326)
**Hounslow**	1·049 (0·848–1·297)	1·015 (0·921–1·118)	0·983 (0·690–1·401)	0·947 (0·858–1·045)	1·148 (0·929–1·418)	0·942 (0·660–1·344)
**Huntingdonshire**	0·990 (0·781–1·253)	1·020 (0·939–1·108)	1·139 (0·922–1·407)	0·937 (0·836–1·051)	1·053 (0·868–1·278)	0·881 (0·507–1·531)
**Ipswich**	0·979 (0·776–1·233)	0·996 (0·878–1·130)	1·025 (0·869–1·209)	0·954 (0·808–1·126)	1·057 (0·846–1·319)	0·959 (0·609–1·511)
**Isle of Wight**	1·106 (0·862–1·421)	1·064 (0·886–1·278)	0·978 (0·805–1·188)	0·897 (0·728–1·105)	1·021 (0·686–1·520)	0·755 (0·416–1·371)
**Isles of Scilly**	1·190 (0·944–1·500)	0·970 (0·789–1·192)	0·989 (0·729–1·341)	0·909 (0·795–1·040)	0·904 (0·664–1·232)	0·573 (0·288–1·139)
**Islington**	1·010 (0·630–1·621)	1·034 (0·878–1·218)	1·191 (0·848–1·673)	0·973 (0·835–1·133)	1·150 (0·886–1·493)	1·065 (0·738–1·537)
**Kensington & Chelsea**	0·907 (0·754–1·091)	1·009 (0·919–1·107)	1·337 (1·002–1·785)	0·896 (0·757–1·061)	1·065 (0·832–1·363)	1·013 (0·807–1·272)
**King's Lynn & West Norfolk**	1·076 (0·958–1·209)	0·990 (0·912–1·074)	0·993 (0·843–1·169)	1·014 (0·963–1·068)	1·159 (0·968–1·388)	1·144 (0·801–1·635)
**Kingston upon Hull, City of**	1·091 (0·784–1·517)	1·038 (0·911–1·182)	0·903 (0·691–1·180)	0·875 (0·726–1·055)	1·023 (0·764–1·370)	0·621 (0·356–1·086)
**Kingston upon Thames**	0·998 (0·921–1·081)	0·972 (0·912–1·037)	1·166 (0·979–1·388)	0·987 (0·951–1·024)	1·031 (0·890–1·195)	1·031 (0·854–1·245)
**Kirklees**	1·196 (0·968–1·478)	0·984 (0·899–1·078)	0·956 (0·768–1·189)	1·052 (0·907–1·220)	1·124 (0·919–1·374)	1·131 (0·652–1·963)
**Knowsley**	1·092 (0·789–1·512)	1·076 (0·982–1·179)	1·170 (0·982–1·394)	0·876 (0·682–1·124)	0·884 (0·658–1·188)	0·708 (0·443–1·132)
**Lambeth**	1·002 (0·719–1·396)	0·982 (0·847–1·138)	1·126 (0·769–1·649)	1·001 (0·841–1·190)	1·291 (0·967–1·722)	1·071 (0·692–1·657)
**Lancaster**	0·998 (0·756–1·318)	0·998 (0·894–1·114)	1·078 (0·927–1·253)	0·954 (0·766–1·187)	1·048 (0·754–1·459)	0·888 (0·455–1·731)
**Leeds**	1·158 (0·840–1·598)	0·962 (0·822–1·127)	1·010 (0·677–1·508)	0·998 (0·847–1·175)	1·023 (0·746–1·403)	0·890 (0·487–1·626)
**Leicester**	1·183 (0·912–1·534)	1·062 (0·967–1·166)	0·997 (0·767–1·297)	0·937 (0·841–1·043)	1·027 (0·836–1·261)	1·097 (0·706–1·706)
**Lewes**	1·091 (0·939–1·269)	1·019 (0·946–1·097)	0·990 (0·877–1·117)	0·974 (0·923–1·028)	1·176 (0·991–1·395)	1·088 (0·796–1·487)
**Lewisham**	0·967 (0·755–1·240)	1·012 (0·892–1·148)	1·060 (0·766–1·466)	0·961 (0·878–1·053)	1·132 (0·884–1·450)	0·933 (0·634–1·372)
**Lichfield**	1·078 (0·951–1·223)	1·031 (0·951–1·117)	1·050 (0·912–1·209)	0·904 (0·782–1·044)	1·010 (0·808–1·262)	0·891 (0·594–1·336)

Local authority	Retail & recreation (per weekly increase of 15% of the baseline visits)	Grocery & pharmacy (per weekly increase of 5% of the baseline visits)	Transit stations (per weekly increase of 10% of the baseline visits)	Parks (per weekly increase of 40% of the baseline visits)	Workplaces (per weekly increase of 10% of the baseline visits)	Residential (per weekly increase of 5% of the baseline time length))
**Lincoln**	0·982 (0·861–1·120)	1·004 (0·945–1·067)	0·983 (0·883–1·095)	0·971 (0·924–1·020)	1·040 (0·917–1·180)	0·880 (0·690–1·123)
**Liverpool**	1·186 (0·878–1·602)	1·113 (0·949–1·304)	0·893 (0·483–1·650)	0·979 (0·715–1·340)	0·961 (0·668–1·383)	0·739 (0·335–1·631)
**Maidstone**	1·038 (0·968–1·114)	0·986 (0·920–1·056)	1·012 (0·913–1·121)	0·949 (0·861–1·045)	1·039 (0·930–1·161)	1·037 (0·828–1·298)
**Maldon**	1·046 (0·927–1·180)	1·002 (0·902–1·112)	1·016 (0·870–1·187)	0·978 (0·925–1·035)	1·100 (0·916–1·322)	1·047 (0·756–1·449)
**Malvern Hills**	1·055 (0·920–1·209)	1·023 (0·944–1·108)	1·042 (0·907–1·197)	0·964 (0·880–1·055)	1·047 (0·911–1·202)	0·951 (0·706–1·280)
**Manchester**	0·922 (0·532–1·598)	1·036 (0·834–1·288)	1·059 (0·473–2·372)	1·006 (0·749–1·351)	0·919 (0·566–1·492)	0·631 (0·254–1·565)
**Mansfield**	1·050 (0·900–1·225)	1·044 (0·959–1·137)	0·950 (0·799–1·128)	0·883 (0·726–1·073)	1·082 (0·894–1·311)	0·769 (0·539–1·096)
**Medway**	1·089 (0·969–1·223)	0·965 (0·890–1·046)	1·023 (0·879–1·190)	0·987 (0·875–1·113)	1·106 (0·931–1·313)	1·128 (0·836–1·520)
**Mendip**	1·069 (0·925–1·236)	1·017 (0·946–1·093)	0·981 (0·903–1·066)	1·032 (0·901–1·181)	1·163 (0·970–1·393)	1·123 (0·797–1·583)
**Merton**	0·949 (0·814–1·105)	1·038 (0·957–1·125)	1·009 (0·785–1·298)	0·930 (0·839–1·030)	1·079 (0·889–1·310)	0·867 (0·667–1·128)
**Mid Devon**	1·025 (0·913–1·151)	0·987 (0·911–1·070)	1·026 (0·951–1·106)	0·941 (0·874–1·013)	1·115 (0·979–1·269)	0·957 (0·784–1·169)
**Mid Suffolk**	1·087 (0·931–1·271)	0·992 (0·912–1·079)	1·017 (0·867–1·192)	0·936 (0·818–1·072)	1·083 (0·927–1·266)	1·058 (0·788–1·422)
**Mid Sussex**	1·034 (0·882–1·212)	1·043 (0·941–1·156)	1·021 (0·906–1·150)	0·937 (0·830–1·059)	1·067 (0·916–1·243)	0·916 (0·659–1·273)
**Middlesbrough**	1·159 (1·008–1·333)	0·975 (0·911–1·044)	1·016 (0·893–1·156)	0·981 (0·917–1·049)	1·028 (0·855–1·236)	0·950 (0·718–1·258)
**Milton Keynes**	1·029 (0·867–1·221)	0·987 (0·882–1·104)	1·076 (0·831–1·393)	0·983 (0·852–1·135)	1·162 (0·901–1·498)	1·035 (0·588–1·824)
**Mole Valley**	0·973 (0·844–1·122)	1·054 (0·967–1·150)	1·005 (0·893–1·130)	0·927 (0·733–1·171)	1·187 (1·011–1·394)	0·924 (0·718–1·189)
**Monmouthshire**	1·085 (0·970–1·214)	1·000 (0·938–1·066)	0·962 (0·877–1·056)	1·012 (0·909–1·126)	1·040 (0·920–1·176)	1·058 (0·924–1·211)
**Moray**	1·284 (0·888–1·857)	1·001 (0·878–1·142)	1·014 (0·762–1·350)	0·948 (0·784–1·145)	0·935 (0·662–1·320)	0·872 (0·452–1·682)
**Neath Port Talbot**	1·144 (0·895–1·463)	0·970 (0·896–1·049)	1·050 (0·894–1·232)	0·961 (0·892–1·036)	1·082 (0·884–1·325)	1·055 (0·735–1·514)
**New Forest**	1·036 (0·923–1·162)	1·099 (0·981–1·232)	0·995 (0·888–1·115)	0·906 (0·764–1·076)	1·062 (0·827–1·363)	0·887 (0·536–1·469)
**Newark & Sherwood**	1·022 (0·932–1·122)	1·037 (0·982–1·095)	0·985 (0·913–1·062)	0·979 (0·875–1·094)	1·040 (0·896–1·207)	0·866 (0·673–1·115)
**Newcastle-under-Lyme**	1·247 (0·837–1·859)	1·116 (0·891–1·396)	0·798 (0·546–1·167)	1·122 (0·950–1·327)	0·758 (0·476–1·206)	0·570 (0·238–1·362)
**Newcastle upon Tyne**	1·071 (0·832–1·379)	0·982 (0·903–1·067)	1·218 (0·889–1·669)	0·982 (0·901–1·072)	1·139 (0·940–1·380)	1·193 (0·827–1·722)
**Newham**	0·994 (0·843–1·173)	0·989 (0·895–1·092)	1·052 (0·793–1·396)	0·971 (0·921–1·023)	1·088 (0·897–1·320)	0·962 (0·709–1·307)
**Newport**	1·291 (0·930–1·791)	0·963 (0·865–1·072)	0·909 (0·791–1·045)	1·036 (0·871–1·233)	1·011 (0·787–1·300)	0·992 (0·657–1·498)
**Newry, Mourne & Down**	1·106 (0·931–1·314)	1·001 (0·922–1·087)	1·003 (0·820–1·226)	1·002 (0·889–1·129)	0·951 (0·704–1·285)	0·903 (0·499–1·635)
**North Devon**	1·033 (0·898–1·190)	1·011 (0·928–1·102)	0·986 (0·868–1·120)	0·996 (0·946–1·048)	1·089 (0·905–1·310)	0·948 (0·668–1·345)
**North East Derbyshire**	0·997 (0·877–1·132)	1·043 (0·970–1·121)	0·989 (0·932–1·049)	0·955 (0·798–1·144)	0·984 (0·804–1·206)	0·762 (0·497–1·168)
**North East Lincolnshire**	1·053 (0·908–1·222)	1·019 (0·943–1·101)	0·969 (0·885–1·061)	0·967 (0·893–1·046)	1·109 (0·904–1·361)	0·802 (0·553–1·164)
**North Hertfordshire**	1·059 (0·886–1·266)	1·010 (0·930–1·098)	1·006 (0·875–1·158)	0·952 (0·846–1·071)	1·076 (0·889–1·301)	0·974 (0·673–1·410)
**North Kesteven**	1·048 (0·956–1·148)	1·004 (0·965–1·046)	0·986 (0·948–1·025)	0·928 (0·813–1·060)	1·072 (0·977–1·177)	0·947 (0·776–1·155)
**North Lincolnshire**	1·123 (0·951–1·325)	0·988 (0·937–1·043)	1·001 (0·933–1·074)	0·950 (0·825–1·093)	1·000 (0·894–1·118)	0·863 (0·656–1·136)
**North Norfolk**	1·014 (0·928–1·106)	1·019 (0·955–1·087)	0·968 (0·914–1·025)	1·004 (0·943–1·068)	1·127 (0·933–1·361)	1·123 (0·803–1·569)
**North Somerset**	1·122 (0·946–1·332)	1·013 (0·922–1·113)	1·066 (0·940–1·209)	0·954 (0·872–1·043)	1·182 (0·973–1·436)	1·047 (0·739–1·483)
**North Tyneside**	1·098 (0·912–1·322)	1·001 (0·905–1·107)	0·991 (0·816–1·202)	0·919 (0·793–1·066)	0·929 (0·763–1·130)	0·723 (0·456–1·144)
**North Warwickshire**	1·034 (0·921–1·162)	0·981 (0·929–1·036)	1·051 (0·974–1·134)	0·926 (0·812–1·056)	1·134 (0·957–1·343)	0·905 (0·698–1·173)
**North West Leicestershire**	1·037 (0·854–1·260)	1·060 (0·968–1·161)	1·023 (0·851–1·230)	0·900 (0·748–1·083)	0·938 (0·814–1·080)	0·770 (0·554–1·071)
**Northampton**	1·032 (0·836–1·274)	1·083 (0·953–1·230)	1·036 (0·778–1·380)	0·951 (0·777–1·164)	1·129 (0·902–1·412)	0·991 (0·694–1·415)
**Northumberland**	1·131 (0·961–1·332)	1·000 (0·915–1·092)	1·026 (0·894–1·177)	0·937 (0·856–1·026)	0·853 (0·673–1·081)	0·739 (0·452–1·209)
**Norwich**	1·052 (0·872–1·269)	0·984 (0·902–1·075)	0·997 (0·773–1·286)	0·988 (0·898–1·087)	1·115 (0·914–1·360)	1·060 (0·777–1·446)
**Nottingham**	1·479 (0·754–2·904)	1·165 (0·777–1·747)	0·713 (0·232–2·189)	1·107 (0·774–1·582)	0·971 (0·448–2·104)	1·119 (0·245–5·105)
**Oldham**	0·998 (0·773–1·289)	1·092 (0·961–1·241)	1·053 (0·841–1·317)	0·922 (0·834–1·020)	0·993 (0·785–1·258)	0·754 (0·436–1·305)

Local authority	Retail & recreation (per weekly increase of 15% of the baseline visits)	Grocery & pharmacy (per weekly increase of 5% of the baseline visits)	Transit stations (per weekly increase of 10% of the baseline visits)	Parks (per weekly increase of 40% of the baseline visits)	Workplaces (per weekly increase of 10% of the baseline visits)	Residential (per weekly increase of 5% of the baseline time length))
**Oxford**	1·108 (0·891–1·377)	0·978 (0·893–1·071)	1·068 (0·758–1·506)	1·050 (0·892–1·235)	1·087 (0·920–1·286)	1·152 (0·812–1·635)
**Pembrokeshire**	1·040 (0·862–1·255)	0·976 (0·903–1·055)	1·005 (0·904–1·117)	1·007 (0·917–1·105)	1·032 (0·945–1·127)	1·013 (0·720–1·427)
**Pendle**	1·036 (0·897–1·196)	1·072 (0·992–1·158)	0·967 (0·914–1·022)	0·962 (0·898–1·029)	1·046 (0·903–1·211)	0·898 (0·648–1·244)
**Perth & Kinross**	1·124 (0·858–1·472)	1·060 (0·920–1·222)	0·942 (0·807–1·099)	0·995 (0·835–1·186)	1·003 (0·824–1·221)	0·925 (0·609–1·404)
**Plymouth**	1·164 (0·877–1·547)	1·115 (0·962–1·291)	0·839 (0·671–1·049)	1·039 (0·843–1·280)	1·106 (0·876–1·396)	0·779 (0·415–1·462)
**Portsmouth**	1·205 (0·960–1·513)	1·019 (0·911–1·141)	0·889 (0·698–1·131)	1·016 (0·896–1·152)	1·193 (0·904–1·575)	1·018 (0·550–1·884)
**Powys**	0·993 (0·929–1·063)	1·010 (0·962–1·060)	0·994 (0·912–1·083)	0·984 (0·895–1·082)	1·020 (0·941–1·106)	0·934 (0·791–1·102)
**Preston**	0·992 (0·874–1·126)	1·028 (0·964–1·097)	1·031 (0·845–1·257)	0·972 (0·901–1·048)	1·031 (0·896–1·186)	0·795 (0·553–1·143)
**Reading**	0·952 (0·775–1·170)	1·019 (0·935–1·111)	1·088 (0·759–1·559)	0·979 (0·923–1·037)	1·127 (0·919–1·382)	1·002 (0·734–1·367)
**Redbridge**	1·106 (0·942–1·298)	0·986 (0·900–1·081)	0·933 (0·647–1·346)	0·973 (0·892–1·061)	1·090 (0·932–1·276)	0·959 (0·650–1·417)
**Redcar & Cleveland**	1·134 (0·946–1·358)	1·073 (0·961–1·198)	0·906 (0·759–1·082)	0·992 (0·899–1·094)	1·054 (0·868–1·279)	0·785 (0·492–1·254)
**Redditch**	1·097 (0·946–1·272)	1·021 (0·919–1·134)	1·105 (0·912–1·339)	0·968 (0·897–1·045)	1·012 (0·824–1·244)	0·879 (0·633–1·221)
**Reigate & Banstead**	1·056 (0·864–1·292)	0·947 (0·862–1·041)	1·133 (0·870–1·477)	0·903 (0·798–1·021)	1·076 (0·901–1·286)	1·002 (0·692–1·450)
**Rhondda Cynon Taf**	1·280 (0·967–1·695)	0·926 (0·783–1·096)	1·100 (0·673–1·799)	0·978 (0·823–1·162)	0·989 (0·701–1·397)	1·017 (0·545–1·896)
**Ribble Valley**	1·066 (0·911–1·249)	1·020 (0·959–1·085)	1·036 (0·892–1·204)	0·978 (0·907–1·053)	1·048 (0·899–1·222)	0·984 (0·723–1·339)
**Richmond upon Thames**	0·982 (0·803–1·200)	0·918 (0·830–1·015)	1·155 (0·897–1·487)	0·967 (0·867–1·078)	1·031 (0·935–1·137)	0·928 (0·642–1·340)
**Richmondshire**	1·087 (0·976–1·211)	1·019 (0·937–1·108)	1·025 (0·961–1·094)	0·930 (0·826–1·047)	1·041 (0·894–1·211)	0·955 (0·763–1·195)
**Rochdale**	1·065 (0·897–1·266)	1·045 (0·975–1·121)	0·996 (0·871–1·139)	0·968 (0·898–1·044)	0·999 (0·892–1·119)	0·845 (0·587–1·216)
**Rother**	1·050 (0·971–1·134)	0·978 (0·920–1·039)	0·992 (0·881–1·117)	1·005 (0·880–1·147)	1·097 (0·966–1·247)	1·108 (0·870–1·411)
**Rotherham**	1·017 (0·871–1·188)	1·012 (0·938–1·091)	1·022 (0·902–1·159)	0·906 (0·781–1·052)	0·982 (0·799–1·207)	0·859 (0·584–1·263)
**Rugby**	1·106 (0·947–1·292)	0·993 (0·920–1·072)	1·072 (0·925–1·241)	0·962 (0·878–1·054)	1·008 (0·892–1·139)	0·957 (0·707–1·296)
**Runnymede**	1·023 (0·938–1·116)	1·023 (0·967–1·081)	0·994 (0·922–1·072)	0·992 (0·947–1·040)	1·082 (0·950–1·232)	0·987 (0·867–1·125)
**Rushcliffe**	1·013 (0·868–1·182)	1·023 (0·947–1·106)	0·960 (0·881–1·046)	0·943 (0·843–1·055)	1·131 (0·918–1·394)	0·796 (0·547–1·159)
**Rushmoor**	0·935 (0·673–1·298)	1·066 (0·923–1·232)	1·123 (0·940–1·340)	0·971 (0·900–1·046)	1·187 (0·946–1·490)	0·977 (0·652–1·465)
**Ryedale**	1·044 (0·962–1·133)	1·013 (0·931–1·102)	1·010 (0·906–1·126)	0·943 (0·802–1·108)	1·093 (0·903–1·324)	0·938 (0·678–1·298)
**Salford**	1·116 (0·870–1·431)	1·040 (0·953–1·135)	0·980 (0·843–1·139)	0·984 (0·873–1·108)	1·175 (0·923–1·495)	0·888 (0·626–1·259)
**Sandwell**	1·197 (0·961–1·491)	1·030 (0·941–1·129)	1·180 (0·901–1·546)	0·969 (0·844–1·114)	1·091 (0·908–1·311)	1·107 (0·644–1·900)
**Scarborough**	1·212 (1·000–1·468)	1·096 (0·953–1·261)	0·905 (0·761–1·075)	0·917 (0·827–1·017)	0·987 (0·723–1·348)	0·882 (0·460–1·693)
**Scottish Borders**	1·167 (0·896–1·521)	1·043 (0·912–1·192)	0·986 (0·815–1·192)	0·933 (0·711–1·224)	0·916 (0·757–1·108)	0·812 (0·521–1·267)
**Sedgemoor**	1·126 (0·980–1·295)	1·019 (0·939–1·106)	0·990 (0·932–1·052)	0·974 (0·902–1·052)	1·233 (0·973–1·561)	1·073 (0·749–1·539)
**Sefton**	1·080 (0·821–1·422)	1·102 (0·930–1·307)	0·875 (0·647–1·184)	0·961 (0·844–1·094)	0·852 (0·603–1·205)	0·548 (0·243–1·239)
**Selby**	1·186 (0·968–1·453)	1·022 (0·955–1·093)	0·992 (0·913–1·077)	0·897 (0·724–1·112)	1·072 (0·850–1·352)	1·049 (0·673–1·635)
**Sevenoaks**	1·089 (0·950–1·248)	0·979 (0·902–1·062)	1·078 (0·944–1·232)	0·895 (0·795–1·008)	1·036 (0·878–1·223)	1·021 (0·813–1·281)
**Sheffield**	1·179 (0·905–1·535)	0·932 (0·806–1·078)	0·979 (0·757–1·267)	1·041 (0·871–1·244)	1·149 (0·828–1·594)	1·080 (0·589–1·980)
**Shropshire**	1·116 (0·857–1·453)	1·051 (0·920–1·201)	1·045 (0·873–1·252)	1·023 (0·802–1·305)	1·074 (0·765–1·508)	1·041 (0·506–2·144)
**Slough**	1·112 (0·949–1·303)	1·033 (0·950–1·124)	0·988 (0·847–1·154)	0·974 (0·903–1·050)	1·055 (0·917–1·213)	0·958 (0·739–1·243)
**Solihull**	1·028 (0·885–1·196)	1·019 (0·954–1·089)	1·226 (0·948–1·584)	0·965 (0·886–1·050)	1·018 (0·880–1·177)	0·968 (0·680–1·378)
**Somerset West & Taunton**	1·055 (0·885–1·257)	0·992 (0·893–1·101)	1·001 (0·940–1·065)	0·964 (0·841–1·105)	1·063 (0·867–1·302)	0·889 (0·576–1·370)
**South Bucks**	1·053 (0·917–1·208)	0·982 (0·927–1·040)	1·003 (0·878–1·147)	0·858 (0·717–1·027)	1·039 (0·882–1·222)	0·865 (0·677–1·103)
**South Cambridgeshire**	1·016 (0·883–1·169)	0·985 (0·912–1·063)	1·050 (0·933–1·182)	0·908 (0·791–1·043)	1·050 (0·892–1·237)	0·938 (0·666–1·322)
**South Derbyshire**	1·103 (0·932–1·306)	1·051 (0·966–1·143)	1·037 (0·938–1·146)	0·875 (0·741–1·032)	1·093 (0·877–1·363)	0·902 (0·580–1·404)
**South Gloucestershire**	1·058 (0·855–1·309)	1·048 (0·967–1·135)	0·954 (0·857–1·061)	0·978 (0·868–1·101)	1·210 (1·006–1·455)	0·849 (0·590–1·222)

Local authority	Retail & recreation (per weekly increase of 15% of the baseline visits)	Grocery & pharmacy (per weekly increase of 5% of the baseline visits)	Transit stations (per weekly increase of 10% of the baseline visits)	Parks (per weekly increase of 40% of the baseline visits)	Workplaces (per weekly increase of 10% of the baseline visits)	Residential (per weekly increase of 5% of the baseline time length))
**South Hams**	1·026 (0·912–1·155)	1·049 (0·964–1·141)	0·970 (0·884–1·066)	1·000 (0·881–1·136)	1·195 (0·939–1·522)	0·965 (0·644–1·446)
**South Holland**	1·083 (0·960–1·222)	0·985 (0·924–1·051)	1·005 (0·916–1·102)	0·954 (0·819–1·111)	1·048 (0·903–1·216)	0·962 (0·718–1·290)
**South Kesteven**	1·084 (0·942–1·247)	1·002 (0·937–1·071)	1·003 (0·944–1·064)	0·990 (0·926–1·059)	1·071 (0·931–1·232)	1·039 (0·756–1·427)
**South Lakeland**	1·050 (0·959–1·150)	1·004 (0·951–1·061)	1·043 (0·965–1·127)	0·919 (0·798–1·057)	1·041 (0·875–1·239)	1·011 (0·759–1·349)
**South Lanarkshire**	1·133 (0·948–1·354)	1·011 (0·897–1·139)	0·983 (0·796–1·215)	0·971 (0·883–1·069)	0·900 (0·751–1·079)	0·745 (0·458–1·210)
**South Norfolk**	1·183 (0·998–1·401)	1·013 (0·940–1·091)	1·008 (0·918–1·107)	0·919 (0·769–1·098)	1·112 (0·878–1·409)	1·092 (0·714–1·672)
**South Northamptonshire**	1·065 (0·924–1·227)	0·975 (0·917–1·036)	1·040 (0·931–1·162)	0·917 (0·814–1·033)	1·092 (0·923–1·291)	0·882 (0·648–1·200)
**South Oxfordshire**	0·944 (0·755–1·180)	1·055 (0·929–1·199)	1·054 (0·803–1·383)	0·908 (0·788–1·047)	1·048 (0·842–1·303)	0·767 (0·410–1·435)
**South Ribble**	0·944 (0·813–1·096)	1·057 (0·989–1·130)	0·951 (0·835–1·082)	0·985 (0·916–1·060)	0·994 (0·855–1·155)	0·706 (0·489–1·020)
**South Somerset**	1·123 (0·933–1·353)	0·992 (0·911–1·081)	0·968 (0·883–1·062)	1·032 (0·894–1·192)	1·104 (0·927–1·316)	1·036 (0·653–1·645)
**South Staffordshire**	1·084 (0·954–1·232)	1·022 (0·945–1·106)	1·047 (0·949–1·156)	0·927 (0·817–1·051)	1·062 (0·888–1·272)	0·917 (0·605–1·392)
**South Tyneside**	1·132 (0·866–1·478)	1·004 (0·890–1·131)	1·011 (0·870–1·175)	0·924 (0·846–1·010)	0·895 (0·728–1·101)	0·760 (0·421–1·370)
**Southampton**	1·097 (0·882–1·365)	1·021 (0·908–1·148)	0·964 (0·718–1·295)	0·972 (0·775–1·219)	1·182 (0·923–1·514)	0·824 (0·489–1·388)
**Southend-on-Sea**	1·048 (0·906–1·212)	0·978 (0·882–1·085)	1·054 (0·871–1·275)	0·975 (0·936–1·015)	1·150 (0·963–1·373)	1·096 (0·794–1·513)
**Southwark**	0·916 (0·633–1·325)	0·978 (0·836–1·144)	1·266 (0·854–1·877)	0·983 (0·772–1·253)	1·146 (0·875–1·500)	1·045 (0·676–1·614)
**Spelthorne**	1·047 (0·914–1·199)	1·002 (0·917–1·095)	1·019 (0·874–1·188)	0·968 (0·894–1·047)	1·088 (0·904–1·310)	0·938 (0·705–1·249)
**St Albans**	1·114 (0·891–1·393)	0·988 (0·909–1·074)	0·883 (0·694–1·123)	0·974 (0·873–1·086)	1·151 (0·941–1·408)	1·008 (0·725–1·402)
**Stafford**	1·017 (0·894–1·157)	1·043 (0·974–1·118)	0·984 (0·881–1·098)	0·926 (0·807–1·063)	0·979 (0·815–1·175)	0·777 (0·518–1·165)
**Staffordshire Moorlands**	1·002 (0·936–1·074)	1·029 (0·967–1·096)	0·990 (0·952–1·029)	1·011 (0·944–1·084)	0·992 (0·854–1·151)	0·855 (0·662–1·105)
**Stevenage**	1·006 (0·880–1·150)	1·007 (0·931–1·089)	1·015 (0·852–1·209)	0·970 (0·921–1·021)	1·169 (0·986–1·387)	0·930 (0·721–1·200)
**Stirling**	0·930 (0·769–1·126)	1·040 (0·974–1·111)	1·090 (0·918–1·295)	1·004 (0·919–1·096)	1·019 (0·888–1·170)	1·013 (0·775–1·324)
**Stockport**	0·999 (0·849–1·176)	1·086 (1·007–1·172)	1·006 (0·814–1·244)	0·926 (0·806–1·064)	1·033 (0·855–1·247)	0·761 (0·506–1·145)
**Stockton-on-Tees**	1·104 (0·942–1·295)	0·994 (0·929–1·065)	1·013 (0·928–1·106)	0·919 (0·824–1·024)	0·977 (0·838–1·138)	0·866 (0·649–1·154)
**Stoke-on-Trent**	1·067 (0·929–1·224)	0·987 (0·912–1·067)	1·032 (0·906–1·176)	0·951 (0·875–1·032)	1·051 (0·888–1·243)	0·945 (0·697–1·282)
**Stratford-on-Avon**	1·043 (0·924–1·177)	1·009 (0·942–1·080)	1·059 (0·945–1·187)	0·952 (0·837–1·082)	1·109 (0·924–1·332)	1·013 (0·739–1·389)
**Stroud**	1·071 (0·874–1·312)	0·989 (0·939–1·041)	1·026 (0·956–1·100)	0·912 (0·793–1·050)	1·074 (0·940–1·228)	0·904 (0·650–1·257)
**Sunderland**	1·005 (0·761–1·327)	1·080 (0·957–1·219)	0·908 (0·771–1·069)	0·905 (0·792–1·034)	0·921 (0·743–1·142)	0·577 (0·337–0·987)
**Sutton**	1·043 (0·909–1·197)	1·009 (0·920–1·106)	1·014 (0·766–1·343)	0·939 (0·853–1·033)	1·059 (0·870–1·289)	0·933 (0·666–1·308)
**Swale**	1·104 (0·988–1·233)	1·005 (0·946–1·068)	0·995 (0·898–1·101)	0·971 (0·877–1·075)	1·038 (0·900–1·197)	1·052 (0·801–1·383)
**Swansea**	1·163 (0·872–1·552)	0·948 (0·840–1·070)	0·889 (0·669–1·182)	0·998 (0·912–1·091)	1·097 (0·859–1·401)	0·774 (0·449–1·334)
**Tameside**	1·057 (0·840–1·330)	1·058 (0·947–1·182)	0·984 (0·820–1·181)	0·942 (0·817–1·087)	1·045 (0·853–1·281)	0·753 (0·478–1·188)
**Tandridge**	1·038 (0·906–1·190)	1·013 (0·942–1·089)	1·028 (0·892–1·184)	0·962 (0·845–1·095)	1·089 (0·966–1·228)	1·022 (0·839–1·245)
**Teignbridge**	1·011 (0·868–1·177)	1·043 (0·957–1·137)	0·957 (0·860–1·066)	1·041 (0·909–1·192)	1·183 (0·956–1·466)	0·999 (0·676–1·477)
**Telford & Wrekin**	1·113 (0·912–1·358)	1·026 (0·941–1·119)	1·028 (0·959–1·103)	1·024 (0·893–1·174)	1·067 (0·847–1·344)	1·030 (0·605–1·753)
**Tendring**	1·033 (0·784–1·361)	1·088 (0·961–1·231)	0·959 (0·833–1·105)	0·918 (0·760–1·110)	0·942 (0·644–1·378)	0·803 (0·386–1·673)
**Test Valley**	1·064 (0·826–1·371)	1·006 (0·886–1·141)	1·003 (0·916–1·098)	0·957 (0·786–1·166)	1·076 (0·867–1·335)	0·921 (0·533–1·594)
**Tewkesbury**	1·075 (0·936–1·235)	1·018 (0·943–1·098)	0·998 (0·927–1·075)	0·984 (0·928–1·043)	1·071 (0·918–1·250)	0·940 (0·724–1·221)
**Thanet**	1·135 (0·982–1·310)	1·035 (0·952–1·126)	0·968 (0·879–1·065)	0·986 (0·935–1·039)	1·180 (0·974–1·431)	1·225 (0·827–1·815)
**Three Rivers**	1·025 (0·924–1·138)	0·965 (0·911–1·022)	0·988 (0·857–1·140)	1·048 (0·776–1·415)	1·077 (0·916–1·265)	0·930 (0·753–1·150)
**Thurrock**	1·075 (0·926–1·248)	0·935 (0·779–1·123)	0·974 (0·783–1·212)	1·019 (0·884–1·176)	1·214 (0·921–1·601)	1·004 (0·650–1·552)
**Tonbridge & Malling**	1·065 (0·948–1·196)	0·983 (0·915–1·055)	1·024 (0·911–1·150)	0·928 (0·810–1·063)	1·085 (0·936–1·257)	1·048 (0·819–1·342)
**Torbay**	1·039 (0·905–1·193)	1·058 (0·955–1·172)	0·983 (0·881–1·098)	0·927 (0·813–1·057)	1·117 (0·907–1·376)	0·806 (0·513–1·267)

Local authority	Retail & recreation (per weekly increase of 15% of the baseline visits)	Grocery & pharmacy (per weekly increase of 5% of the baseline visits)	Transit stations (per weekly increase of 10% of the baseline visits)	Parks (per weekly increase of 40% of the baseline visits)	Workplaces (per weekly increase of 10% of the baseline visits)	Residential (per weekly increase of 5% of the baseline time length))
**Torridge**	1·053 (0·969–1·145)	0·990 (0·929–1·056)	0·995 (0·963–1·028)	0·977 (0·873–1·094)	1·024 (0·868–1·209)	0·859 (0·649–1·138)
**Tower Hamlets**	1·108 (0·836–1·469)	1·001 (0·882–1·135)	1·102 (0·848–1·431)	0·945 (0·779–1·145)	1·101 (0·872–1·389)	1·020 (0·731–1·424)
**Trafford**	1·089 (0·966–1·228)	1·048 (0·943–1·165)	1·017 (0·824–1·256)	0·965 (0·818–1·139)	1·066 (0·855–1·329)	0·841 (0·542–1·303)
**Tunbridge Wells**	1·037 (0·920–1·169)	0·998 (0·952–1·047)	1·007 (0·893–1·135)	0·937 (0·810–1·084)	1·019 (0·872–1·190)	0·992 (0·740–1·331)
**Uttlesford**	1·088 (0·899–1·317)	1·008 (0·908–1·120)	0·962 (0·863–1·072)	0·961 (0·801–1·152)	1·075 (0·894–1·292)	0·922 (0·690–1·231)
**Vale of Glamorgan**	1·134 (0·977–1·315)	0·951 (0·888–1·018)	1·040 (0·894–1·211)	0·984 (0·931–1·039)	1·043 (0·907–1·200)	1·102 (0·851–1·427)
**Vale of White Horse**	1·027 (0·866–1·219)	1·050 (0·956–1·153)	0·984 (0·842–1·151)	0·978 (0·869–1·100)	1·134 (0·955–1·348)	0·952 (0·686–1·321)
**Wakefield**	1·051 (0·898–1·230)	0·981 (0·908–1·060)	1·114 (0·996–1·245)	0·958 (0·842–1·090)	1·123 (0·961–1·312)	1·074 (0·777–1·485)
**Walsall**	1·081 (0·896–1·303)	1·029 (0·919–1·152)	1·049 (0·897–1·227)	0·960 (0·813–1·135)	0·999 (0·812–1·229)	0·882 (0·533–1·461)
**Waltham Forest**	1·034 (0·878–1·218)	1·010 (0·919–1·109)	0·966 (0·703–1·327)	0·955 (0·873–1·045)	1·088 (0·901–1·313)	0·957 (0·720–1·274)
**Wandsworth**	1·101 (0·762–1·592)	0·927 (0·760–1·131)	1·481 (0·988–2·221)	1·013 (0·829–1·237)	1·223 (0·921–1·624)	1·361 (0·748–2·475)
**Warrington**	1·005 (0·826–1·224)	0·987 (0·903–1·077)	1·089 (0·945–1·256)	0·962 (0·901–1·027)	1·011 (0·811–1·261)	0·804 (0·557–1·161)
**Warwick**	1·001 (0·869–1·154)	1·028 (0·929–1·139)	0·959 (0·733–1·256)	0·941 (0·824–1·076)	1·078 (0·823–1·411)	0·825 (0·544–1·253)
**Watford**	1·005 (0·905–1·116)	0·992 (0·918–1·072)	0·957 (0·778–1·176)	0·968 (0·891–1·052)	1·083 (0·922–1·271)	0·914 (0·677–1·233)
**Waverley**	1·016 (0·880–1·172)	1·042 (0·952–1·140)	1·003 (0·870–1·156)	0·949 (0·838–1·074)	1·111 (0·946–1·306)	0·933 (0·695–1·252)
**Wealden**	1·025 (0·912–1·153)	1·057 (0·978–1·142)	0·969 (0·903–1·040)	0·929 (0·820–1·052)	1·023 (0·841–1·245)	0·919 (0·625–1·352)
**Welwyn Hatfield**	1·033 (0·880–1·213)	0·987 (0·869–1·122)	1·032 (0·928–1·146)	0·974 (0·903–1·050)	1·077 (0·881–1·317)	0·961 (0·676–1·367)
**West Berkshire**	1·041 (0·895–1·211)	1·016 (0·929–1·112)	1·001 (0·884–1·134)	0·979 (0·835–1·148)	1·136 (0·982–1·314)	0·995 (0·781–1·269)
**West Devon**	0·997 (0·900–1·105)	1·020 (0·974–1·068)	0·961 (0·898–1·029)	1·007 (0·915–1·109)	1·040 (0·930–1·163)	0·874 (0·700–1·092)
**West Lancashire**	0·995 (0·781–1·267)	1·082 (0·988–1·185)	0·956 (0·817–1·117)	0·951 (0·792–1·141)	0·964 (0·743–1·251)	0·692 (0·424–1·128)
**West Oxfordshire**	0·985 (0·751–1·293)	1·045 (0·936–1·166)	0·964 (0·849–1·093)	0·988 (0·866–1·127)	1·177 (0·903–1·533)	0·868 (0·498–1·514)
**West Suffolk**	1·074 (0·892–1·293)	0·981 (0·888–1·083)	0·976 (0·889–1·072)	0·972 (0·831–1·137)	1·134 (0·891–1·444)	1·036 (0·694–1·546)
**Westminster**	1·022 (0·631–1·655)	0·975 (0·885–1·075)	1·310 (0·765–2·245)	0·961 (0·661–1·397)	1·070 (0·866–1·323)	1·072 (0·761–1·511)
**Wigan**	1·016 (0·832–1·241)	1·072 (0·986–1·166)	0·975 (0·834–1·139)	1·012 (0·916–1·119)	1·041 (0·858–1·263)	0·755 (0·504–1·131)
**Wiltshire**	1·080 (0·898–1·299)	1·052 (0·935–1·184)	1·014 (0·839–1·226)	0·907 (0·757–1·087)	1·101 (0·884–1·371)	0·939 (0·576–1·533)
**Winchester**	1·075 (0·897–1·289)	1·025 (0·940–1·116)	1·032 (0·903–1·179)	0·915 (0·693–1·208)	1·121 (0·910–1·380)	0·953 (0·628–1·447)
**Windsor & Maidenhead**	1·081 (0·948–1·231)	1·022 (0·935–1·117)	1·000 (0·927–1·079)	0·937 (0·831–1·055)	1·070 (0·883–1·296)	0·907 (0·706–1·164)
**Wirral**	1·111 (0·859–1·437)	1·061 (0·908–1·240)	1·015 (0·781–1·318)	0·958 (0·829–1·109)	0·927 (0·679–1·266)	0·728 (0·386–1·374)
**Woking**	1·014 (0·915–1·123)	1·017 (0·963–1·074)	1·002 (0·901–1·115)	0·972 (0·859–1·101)	1·052 (0·943–1·174)	0·965 (0·799–1·166)
**Wokingham**	1·025 (0·898–1·168)	0·995 (0·926–1·070)	1·002 (0·915–1·096)	0·984 (0·934–1·037)	1·094 (0·963–1·243)	0·979 (0·773–1·240)
**Wolverhampton**	1·040 (0·863–1·253)	1·118 (1·008–1·241)	0·911 (0·731–1·137)	0·904 (0·760–1·075)	1·072 (0·862–1·334)	0·834 (0·528–1·319)
**Worcester**	1·087 (0·809–1·460)	1·047 (0·947–1·158)	1·029 (0·919–1·152)	0·977 (0·793–1·203)	1·052 (0·748–1·479)	0·921 (0·524–1·619)
**Worthing**	0·937 (0·714–1·230)	1·026 (0·929–1·133)	1·008 (0·736–1·380)	0·937 (0·842–1·044)	1·180 (0·902–1·545)	0·728 (0·354–1·498)
**Wrexham**	1·007 (0·849–1·196)	0·999 (0·896–1·113)	1·090 (0·870–1·366)	0·954 (0·842–1·081)	1·092 (0·872–1·367)	1·058 (0·743–1·506)
**Wychavon**	1·015 (0·878–1·174)	1·044 (0·968–1·126)	1·001 (0·940–1·065)	0·940 (0·802–1·103)	1·072 (0·892–1·288)	0·845 (0·531–1·343)
**Wycombe**	1·041 (0·872–1·242)	1·005 (0·928–1·088)	0·939 (0·800–1·103)	0·964 (0·839–1·109)	1·115 (0·940–1·324)	0·923 (0·640–1·331)
**Wyre**	1·040 (0·912–1·186)	1·012 (0·944–1·084)	1·008 (0·956–1·062)	0·971 (0·853–1·105)	1·095 (0·870–1·379)	0·875 (0·557–1·373)
**Wyre Forest**	1·133 (0·969–1·324)	0·997 (0·916–1·085)	1·052 (0·949–1·166)	0·948 (0·851–1·056)	1·018 (0·864–1·201)	0·953 (0·687–1·321)
**York**	1·127 (0·818–1·552)	0·952 (0·791–1·145)	1·069 (0·819–1·396)	0·987 (0·742–1·314)	1·057 (0·745–1·498)	1·038 (0·616–1·749)

*Baseline period: 3rd Jan 2020–6th Feb 2020.

Table S5. Meta-regression findings showing the modifiers of the association between six Google community mobility metrics and R, as the ratio of R ratios (95% CI)

Mobility metrics	Population density (per 1000 persons/km^2 increase)	Prosperity score* (per 0.1 points increase)	% BAME population (per 10% increase)	Latitude (per 2 degrees increase)	% Households with 6+ members (per 1% increase)
**Retail & recreation**	0·991 (0·984–0·999)	0·976 (0·955–0·997)	1·011 (0·998–1·023)	1·001 (0·985–1·018)	—
**Grocery & pharmacy**	0·997 (0·997–1·005)	1·003 (0·991–1·015)	0·997 (0·991–1·004)	1·009 (1·000–1·018)	—
**Transit stations**	1·010 (1·002–1·017)	1·015 (0·998–1·033)	1·002 (0·988–1·015)	1·010 (0·998–1·022)	—
**Parks**	1·000 (0·996–1·005)	0·995 (0·981–1·010)	0·999 (0·992–1·005)	0·994 (0·984–1·004)	—
**Workplaces**	1·002 (0·995–1·010)	1·006 (0·981–1·032)	1·000 (0·986–1·013)	0·955 (0·938–0·973)	—
**Residential**	1·002 (0·988–1·016)	1·012 (0·960–1·066)	1·003 (0·958–1·050)	0·948 (0·913–0·984)	1·002 (0·997–1·012)

BAME = Black, Asian and minority ethnic. R ratio indicated the weekly change in R associated with weekly difference in mobility metrics relative to the baseline level (baseline period: 3rd Jan 2020–6th Feb 2020). The ratio of R ratios quantified the modification per unit increase in the potential modifiers. Interpretation of the direction of modification is dependent on the R ratio of the corresponding mobility metric (shown in **Table 1**).

*A higher prosperity index indicates less deprivation.

Figure S1. Change over time (by week) in the mobility metrics and R on the local-authority level

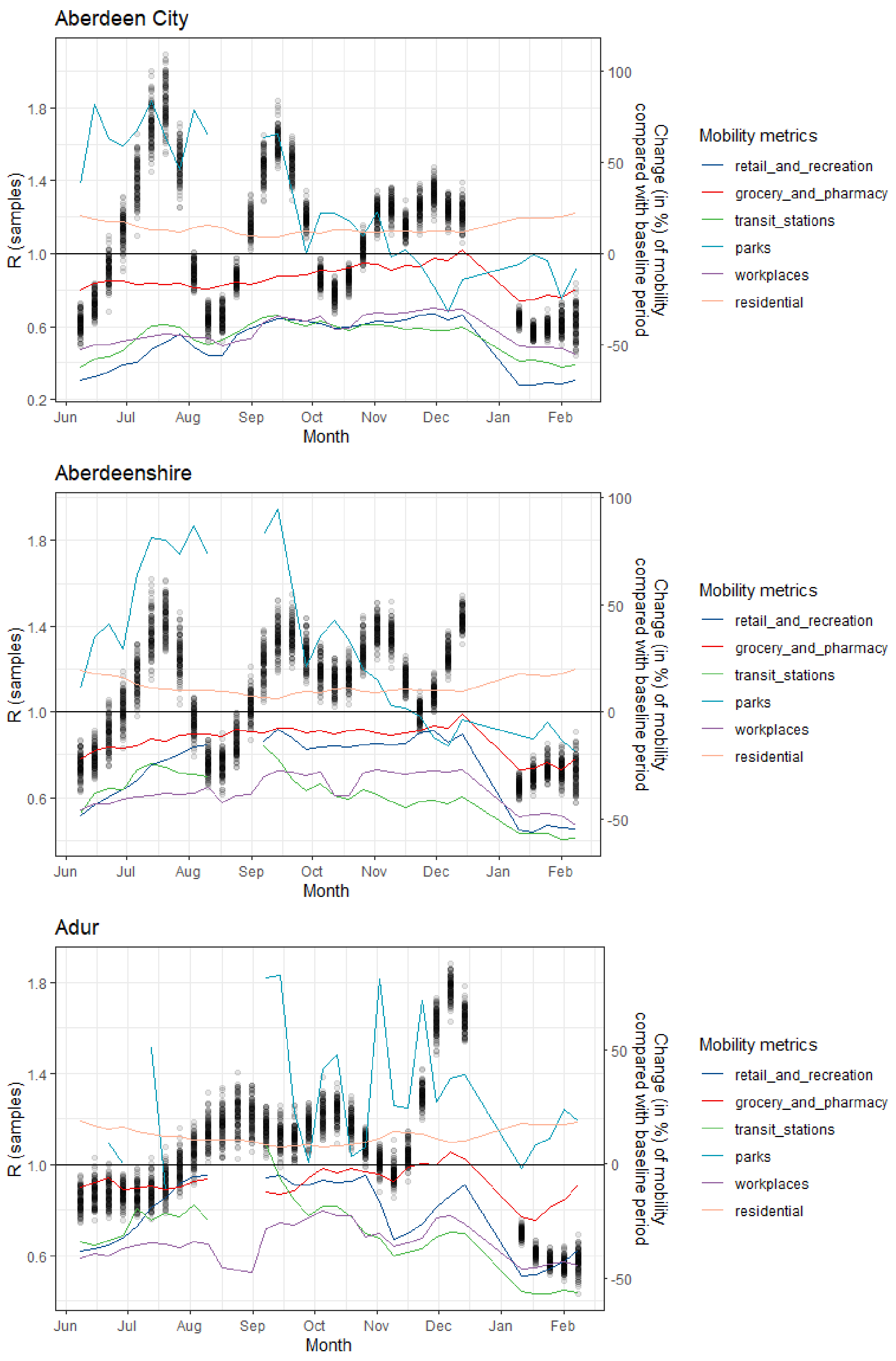

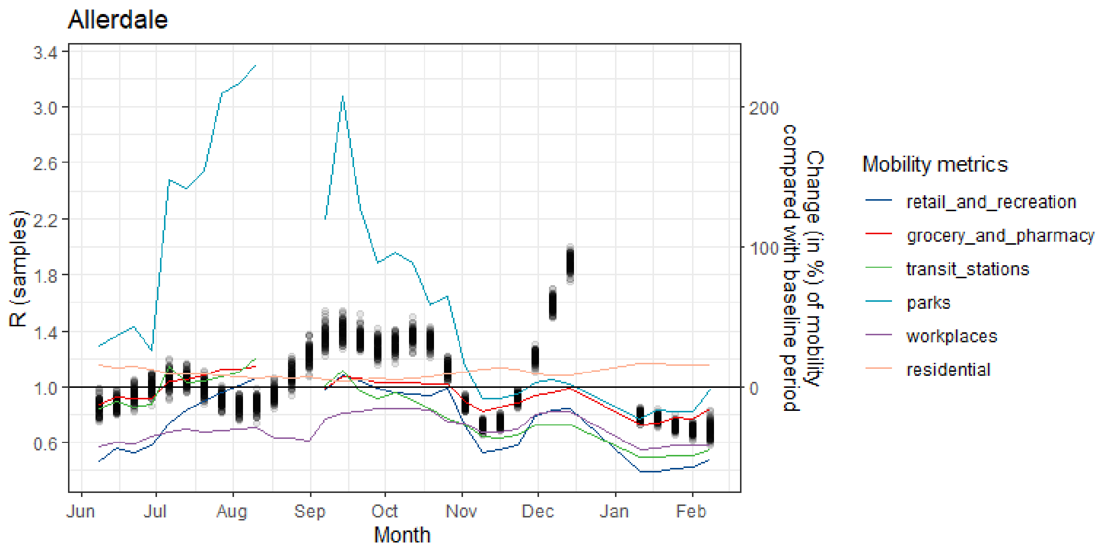
Allerdale
R (samples)
Change (in %) of mobility compared with baseline period
Month
Jun Jul Aug Sep Oct Nov Dec Jan Feb
Mobility metrics
retail_and_recreation
grocery_and_pharmacy
transit_stations
parks
workplaces
residential

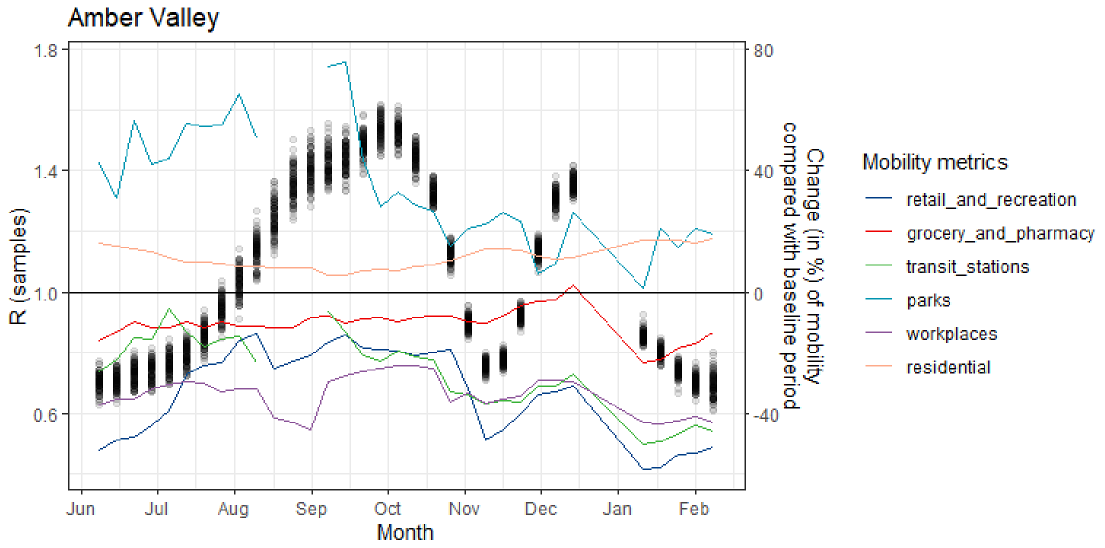
Amber Valley
R (samples)
Change (in %) of mobility compared with baseline period
Month
Jun Jul Aug Sep Oct Nov Dec Jan Feb
Mobility metrics
retail_and_recreation
grocery_and_pharmacy
transit_stations
parks
workplaces
residential

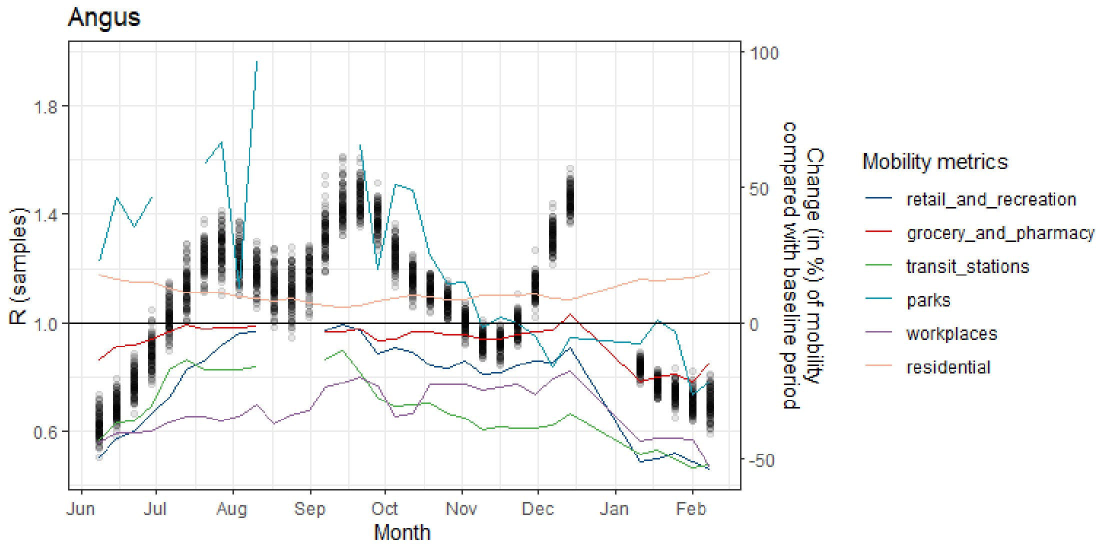
Angus
R (samples)
Change (in %) of mobility compared with baseline period
Month
Jun Jul Aug Sep Oct Nov Dec Jan Feb
Mobility metrics
retail_and_recreation
grocery_and_pharmacy
transit_stations
parks
workplaces
residential

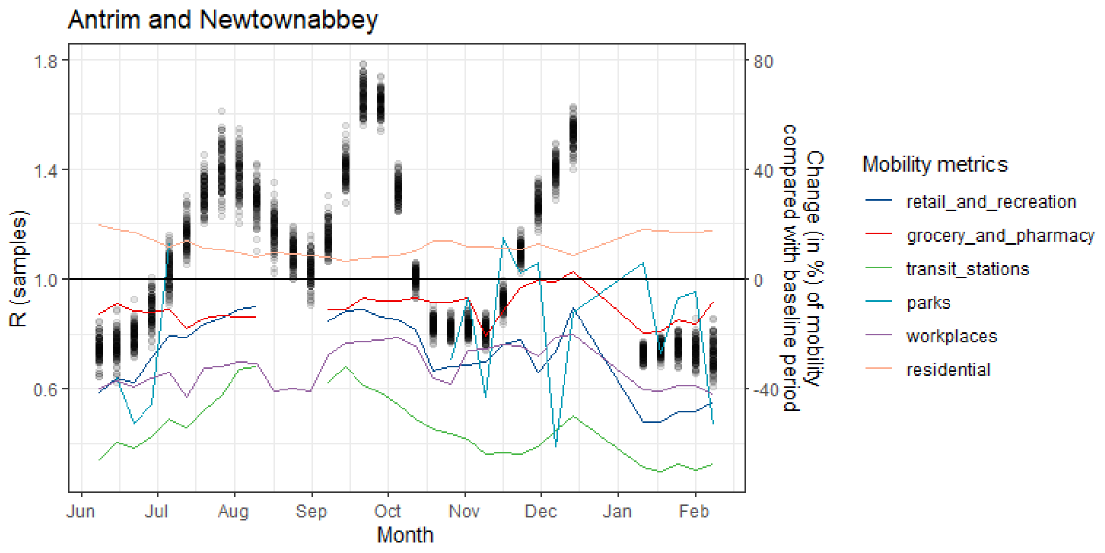
Antrim and Newtownabbey
R (samples)
1.8
1.4
1.0
0.6
Change (in %) of mobility compared with baseline period
80
40
0
-40
Jun
Jul
Aug
Sep
Oct
Nov
Dec
Jan
Feb
Month
Mobility metrics
retail_and_recreation
grocery_and_pharmacy
transit_stations
parks
workplaces
residential

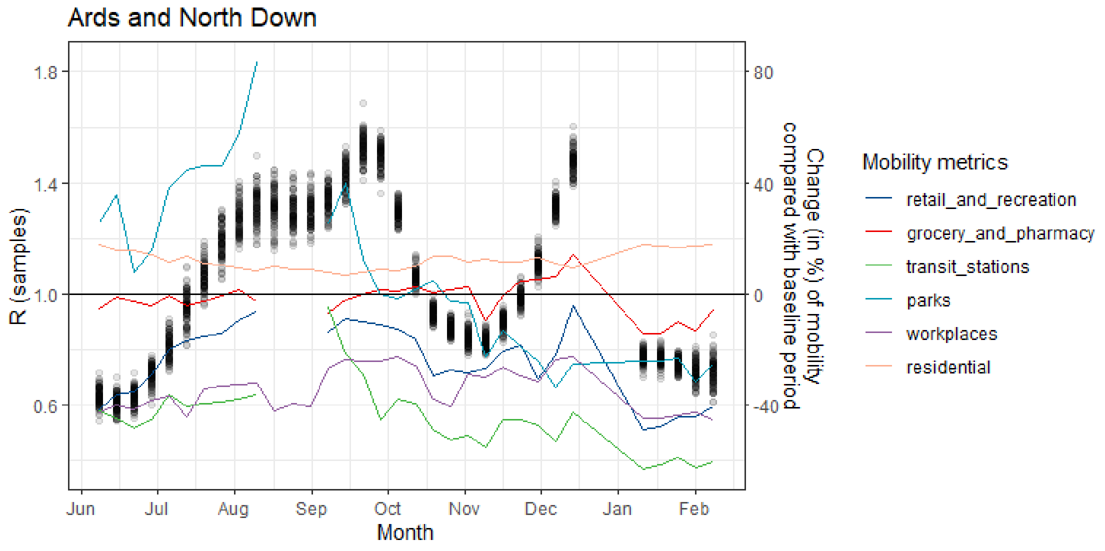
Ards and North Down
R (samples)
1.8
1.4
1.0
0.6
Change (in %) of mobility compared with baseline period
80
40
0
-40
Jun
Jul
Aug
Sep
Oct
Nov
Dec
Jan
Feb
Month
Mobility metrics
retail_and_recreation
grocery_and_pharmacy
transit_stations
parks
workplaces
residential

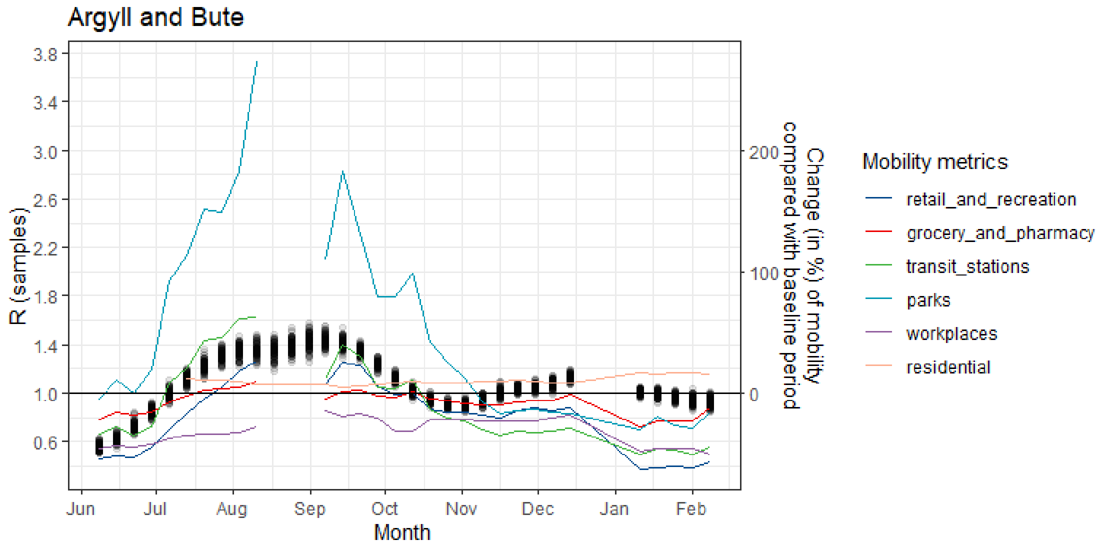
Argyll and Bute
R (samples)
3.8
3.4
3.0
2.6
2.2
1.8
1.4
1.0
0.6
Change (in %) of mobility compared with baseline period
200
100
0
Jun
Jul
Aug
Sep
Oct
Nov
Dec
Jan
Feb
Month
Mobility metrics
retail_and_recreation
grocery_and_pharmacy
transit_stations
parks
workplaces
residential

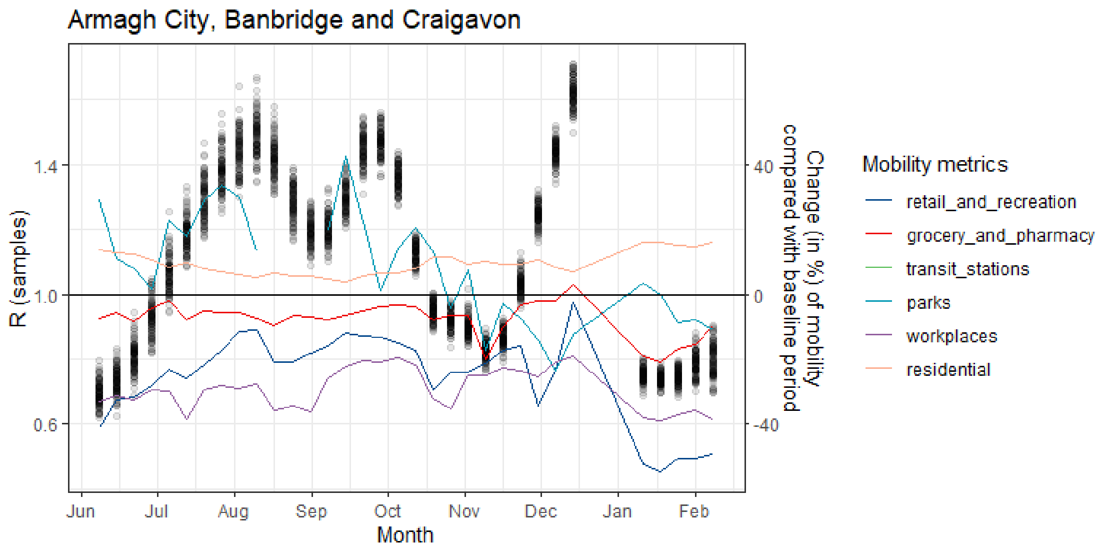

Armagh City, Banbridge and Craigavon
R (samples)
1.4
1.0
0.6
Jun
Jul
Aug
Sep
Oct
Nov
Dec
Jan
Feb
Month
40
0
-40
Change (in %) of mobility compared with baseline period
Mobility metrics
retail_and_recreation
grocery_and_pharmacy
transit_stations
parks
workplaces
residential

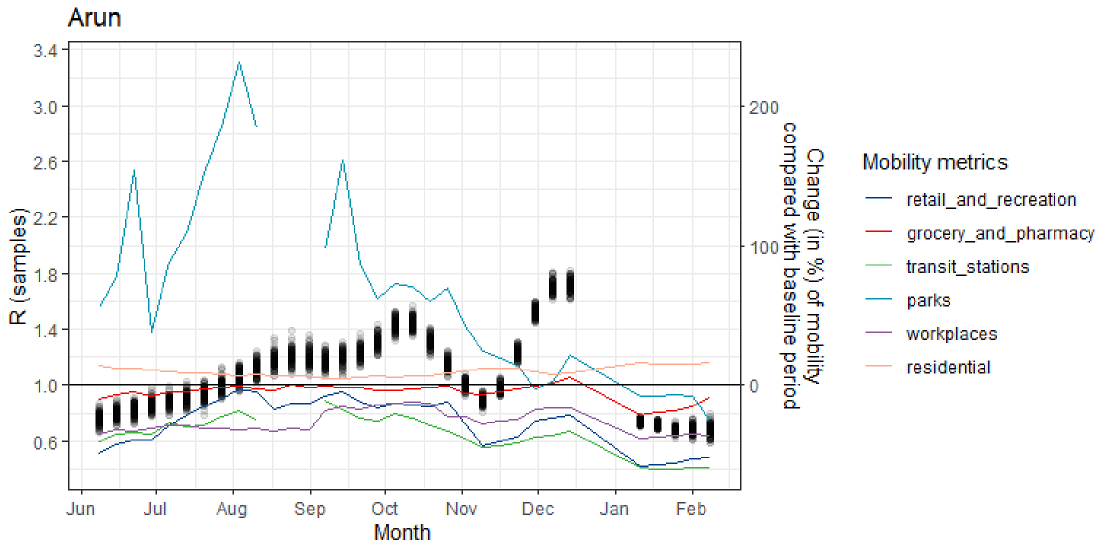

Arun
R (samples)
3.4
3.0
2.6
2.2
1.8
1.4
1.0
0.6
Jun
Jul
Aug
Sep
Oct
Nov
Dec
Jan
Feb
Month
200
100
0
Change (in %) of mobility compared with baseline period
Mobility metrics
retail_and_recreation
grocery_and_pharmacy
transit_stations
parks
workplaces
residential

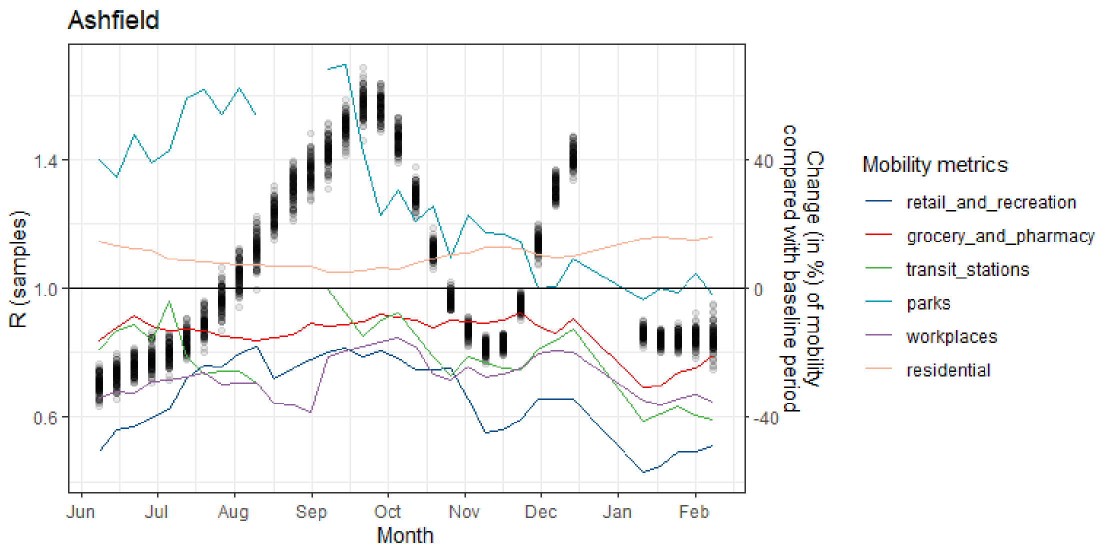

Ashfield
R (samples)
1.4
1.0
0.6
Jun
Jul
Aug
Sep
Oct
Nov
Dec
Jan
Feb
Month
40
0
-40
Change (in %) of mobility compared with baseline period
Mobility metrics
retail_and_recreation
grocery_and_pharmacy
transit_stations
parks
workplaces
residential

Ashford

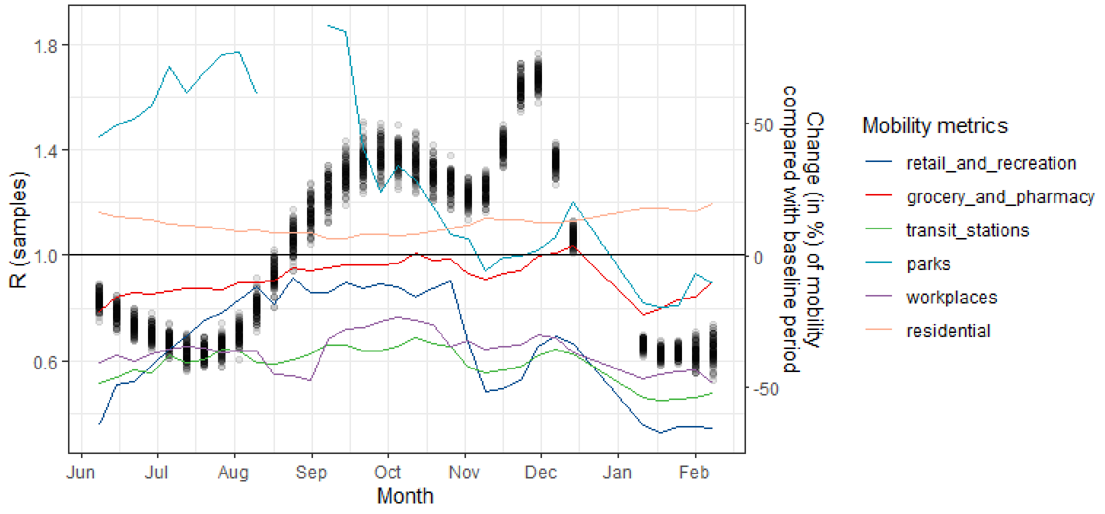

Aylesbury Vale

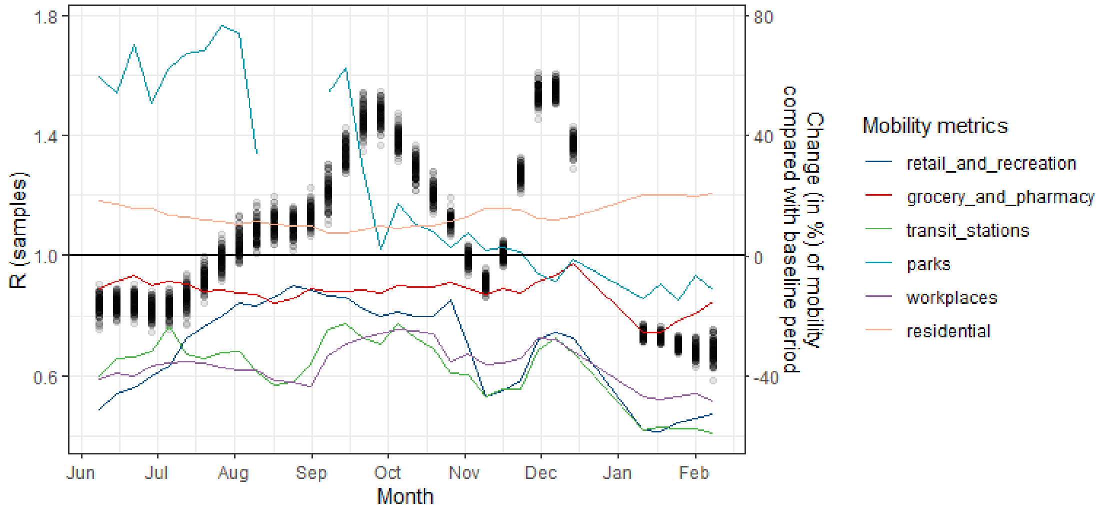

Babergh

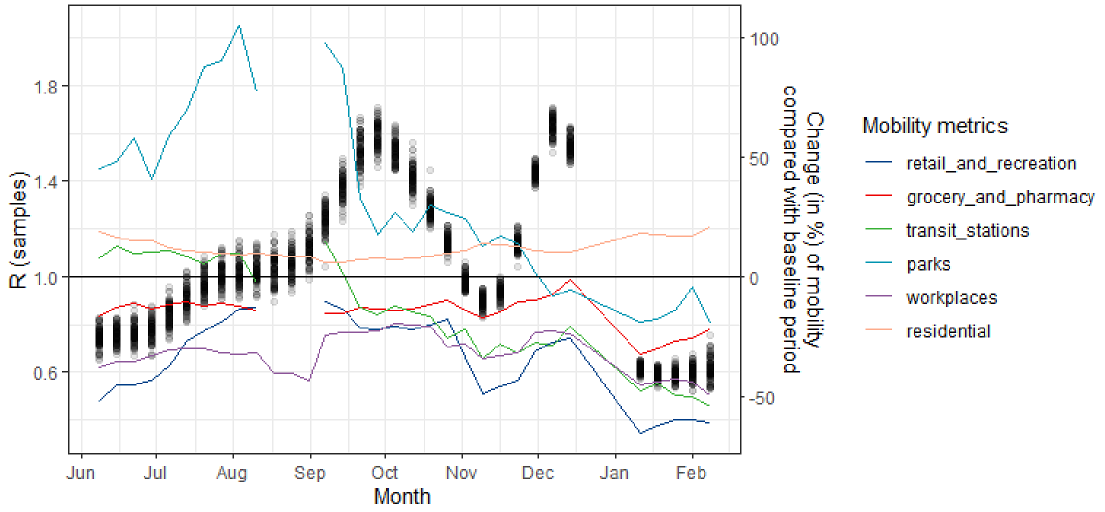

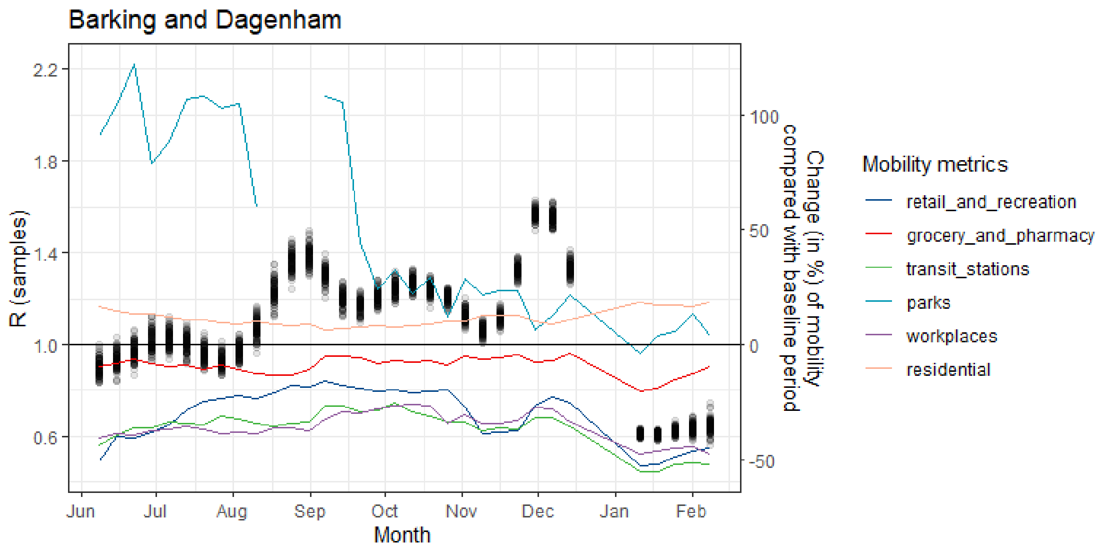
Barking and Dagenham
R (samples)
2.2
1.8
1.4
1.0
0.6
Jun Jul Aug Sep Oct Nov Dec Jan Feb
Month
100
50
0
-50
Change (in %) of mobility compared with baseline period
Mobility metrics
retail_and_recreation
grocery_and_pharmacy
transit_stations
parks
workplaces
residential

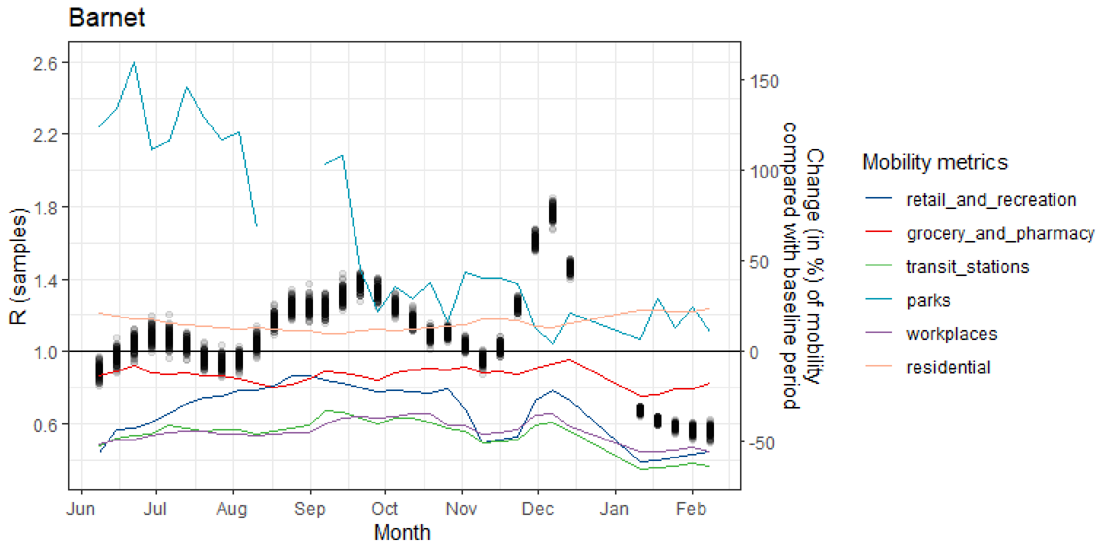
Barnet
R (samples)
2.6
2.2
1.8
1.4
1.0
0.6
Jun Jul Aug Sep Oct Nov Dec Jan Feb
Month
150
100
50
0
-50
Change (in %) of mobility compared with baseline period
Mobility metrics
retail_and_recreation
grocery_and_pharmacy
transit_stations
parks
workplaces
residential

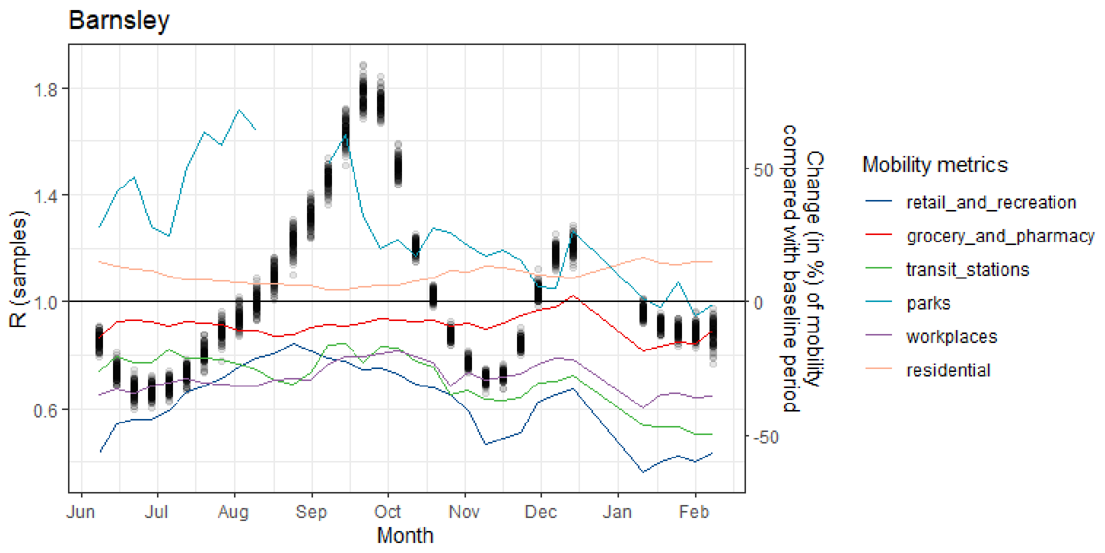
Barnsley
R (samples)
1.8
1.4
1.0
0.6
Jun Jul Aug Sep Oct Nov Dec Jan Feb
Month
50
0
-50
Change (in %) of mobility compared with baseline period
Mobility metrics
retail_and_recreation
grocery_and_pharmacy
transit_stations
parks
workplaces
residential

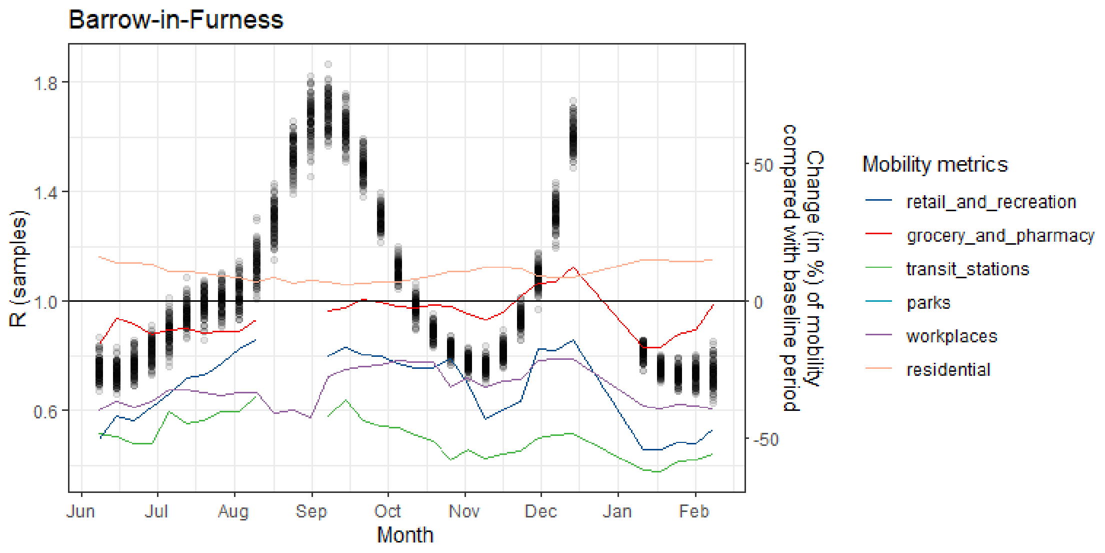
Barrow-in-Furness
R (samples)
1.8
1.4
1.0
0.6
50
0
-50
Change (in %) of mobility compared with baseline period
Jun
Jul
Aug
Sep
Oct
Nov
Dec
Jan
Feb
Month
Mobility metrics
retail_and_recreation
grocery_and_pharmacy
transit_stations
parks
workplaces
residential

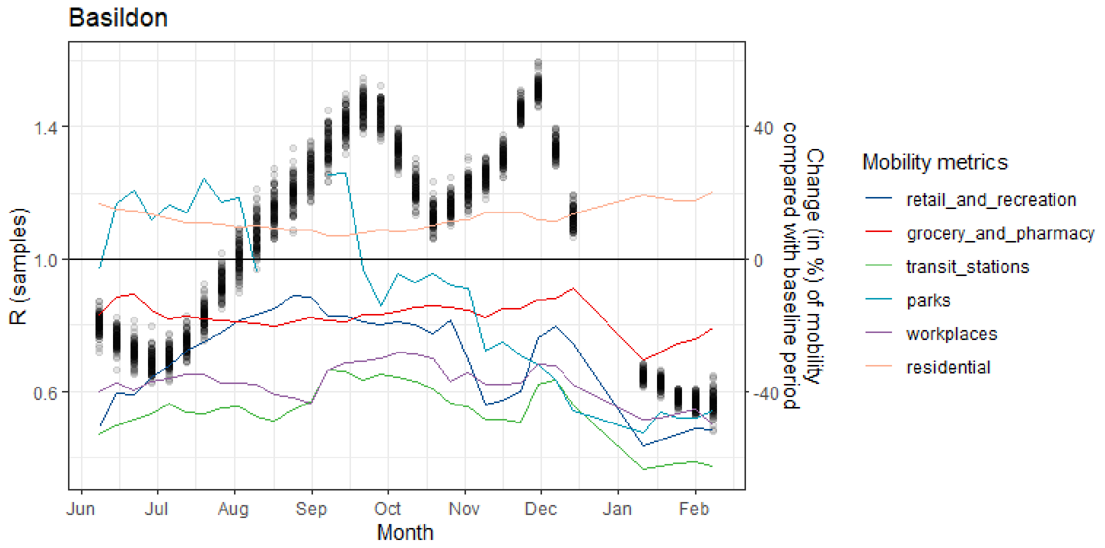
Basildon
R (samples)
1.4
1.0
0.6
40
0
-40
Change (in %) of mobility compared with baseline period
Jun
Jul
Aug
Sep
Oct
Nov
Dec
Jan
Feb
Month
Mobility metrics
retail_and_recreation
grocery_and_pharmacy
transit_stations
parks
workplaces
residential

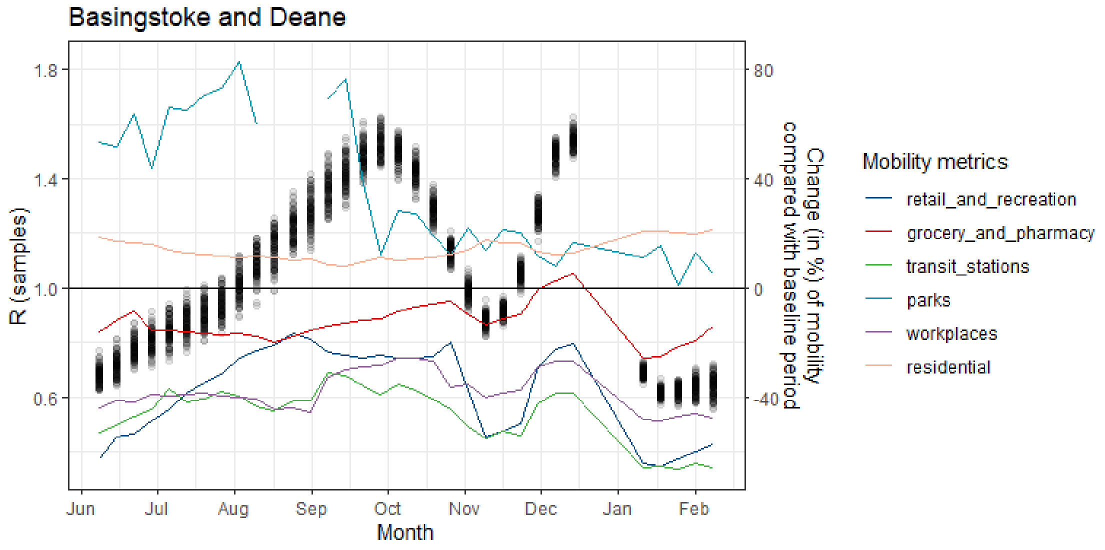
Basingstoke and Deane
R (samples)
1.8
1.4
1.0
0.6
80
40
0
-40
Change (in %) of mobility compared with baseline period
Jun
Jul
Aug
Sep
Oct
Nov
Dec
Jan
Feb
Month
Mobility metrics
retail_and_recreation
grocery_and_pharmacy
transit_stations
parks
workplaces
residential

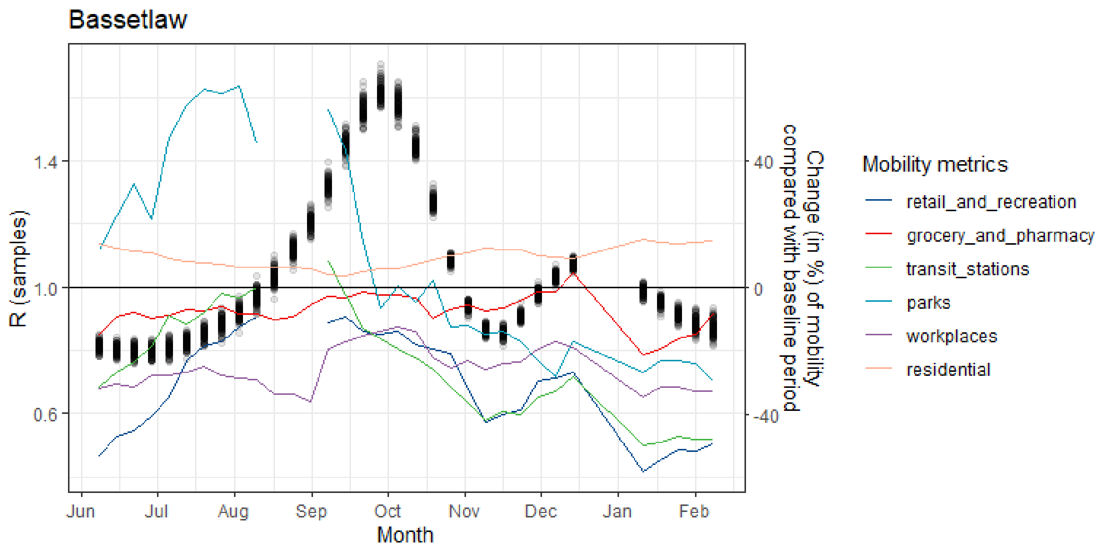
Bassetlaw
R (samples)
1.4
1.0
0.6
Jun Jul Aug Sep Oct Nov Dec Jan Feb
Month
40
0
-40
Change (in %) of mobility compared with baseline period
Mobility metrics
retail_and_recreation
grocery_and_pharmacy
transit_stations
parks
workplaces
residential

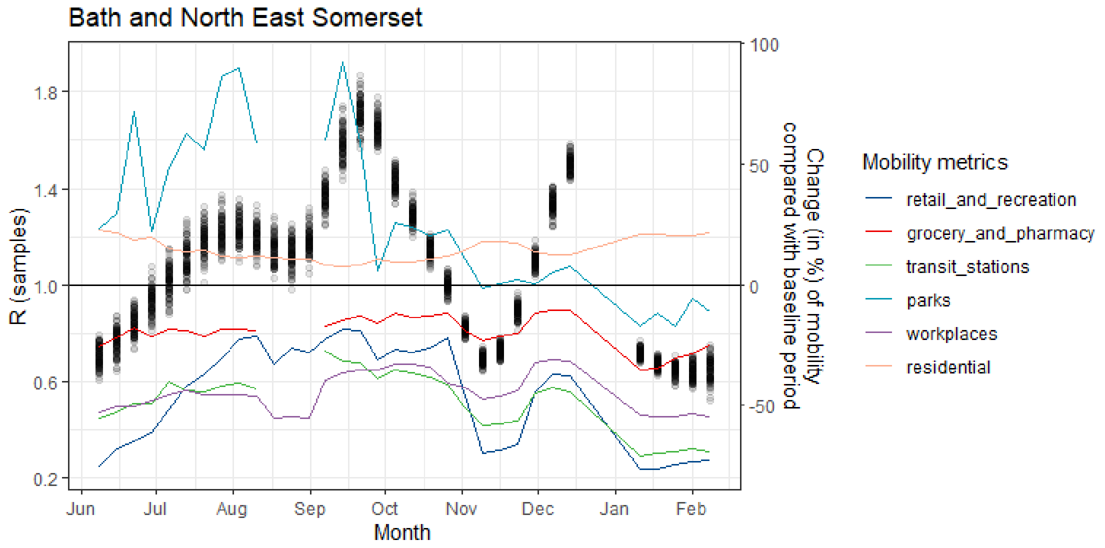
Bath and North East Somerset
R (samples)
1.8
1.4
1.0
0.6
0.2
Jun Jul Aug Sep Oct Nov Dec Jan Feb
Month
100
50
0
-50
Change (in %) of mobility compared with baseline period
Mobility metrics
retail_and_recreation
grocery_and_pharmacy
transit_stations
parks
workplaces
residential

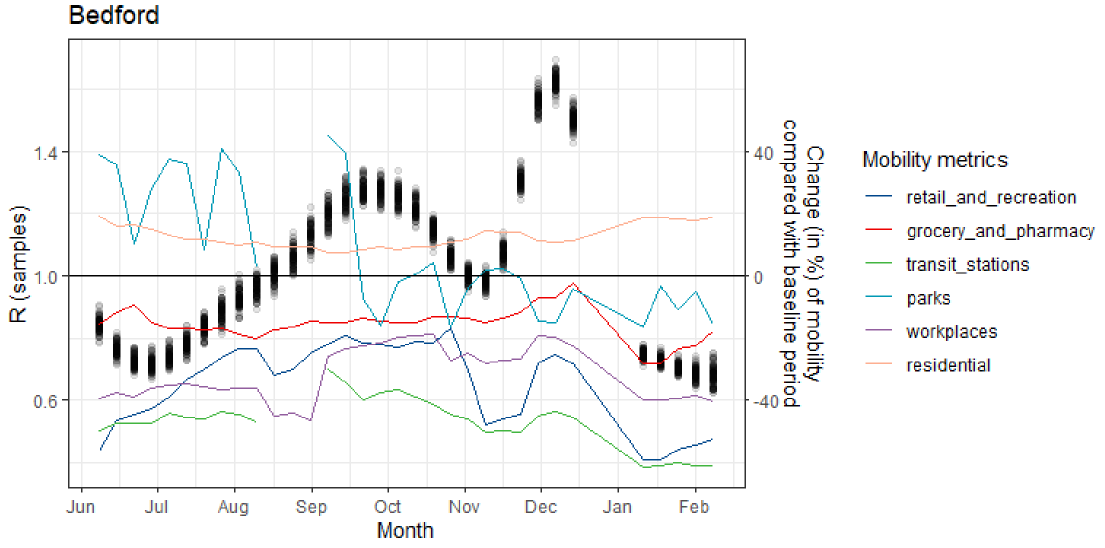
Bedford
R (samples)
1.4
1.0
0.6
Jun Jul Aug Sep Oct Nov Dec Jan Feb
Month
40
0
-40
Change (in %) of mobility compared with baseline period
Mobility metrics
retail_and_recreation
grocery_and_pharmacy
transit_stations
parks
workplaces
residential

Belfast

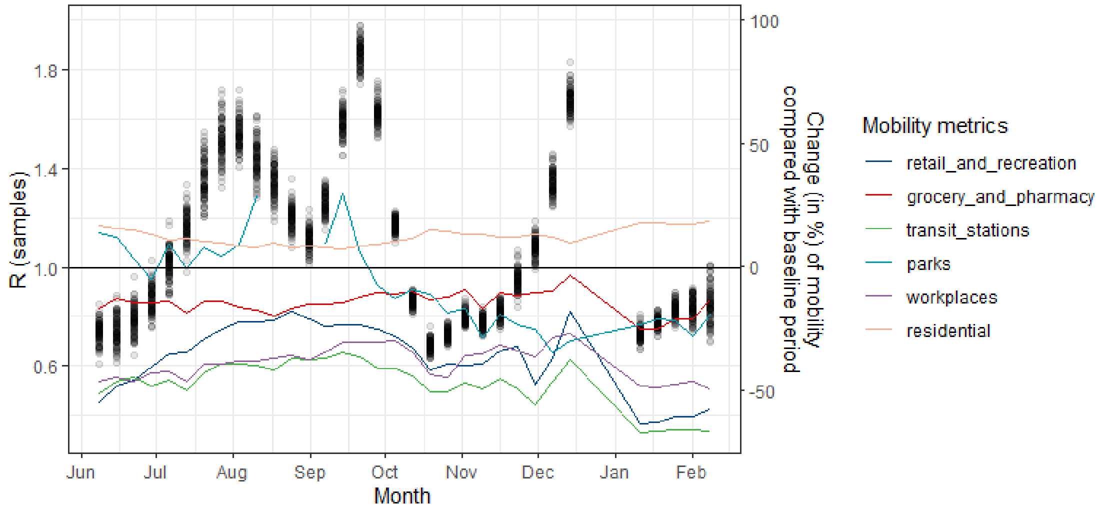

Bexley

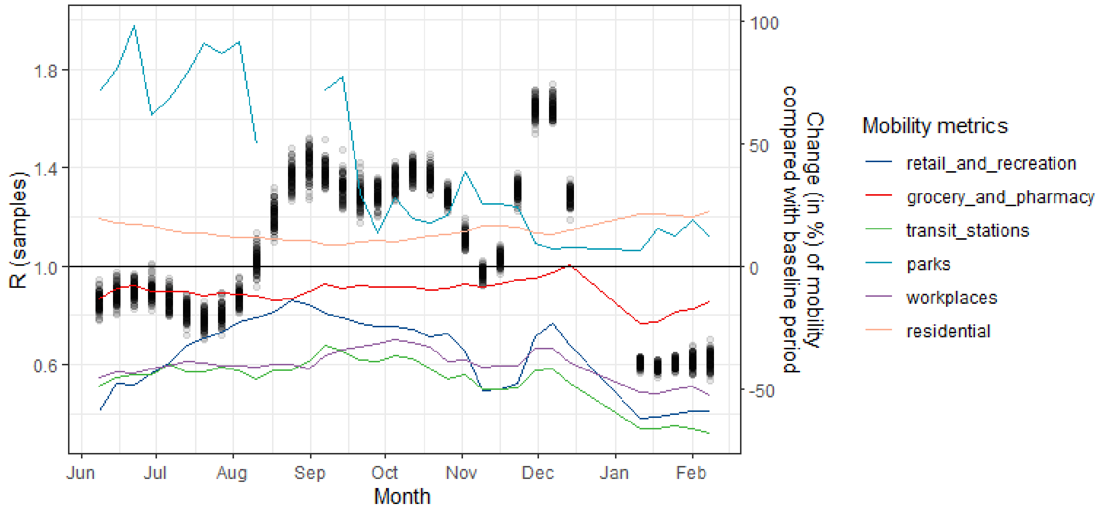

Birmingham

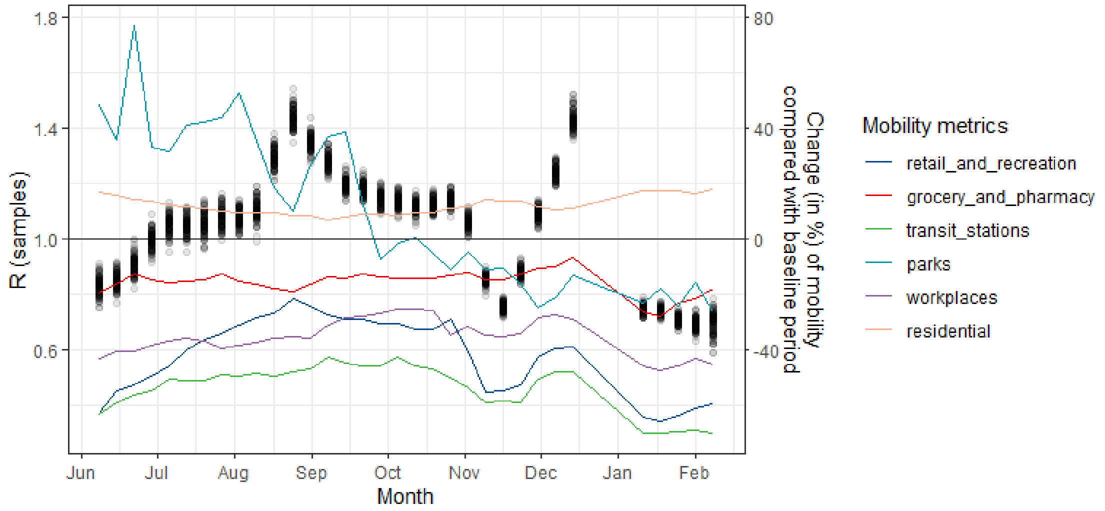

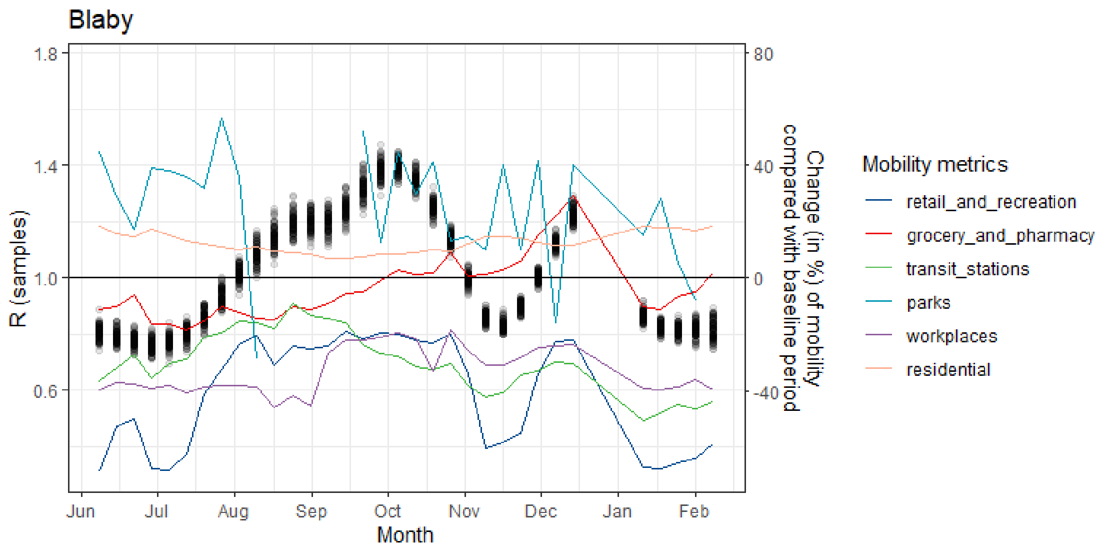

Blaby
1.8
1.4
1.0
0.6
R (samples)
80
40
0
-40
Change (in %) of mobility compared with baseline period
Jun
Jul
Aug
Sep
Oct
Nov
Dec
Jan
Feb
Month
Mobility metrics
retail_and_recreation
grocery_and_pharmacy
transit_stations
parks
workplaces
residential

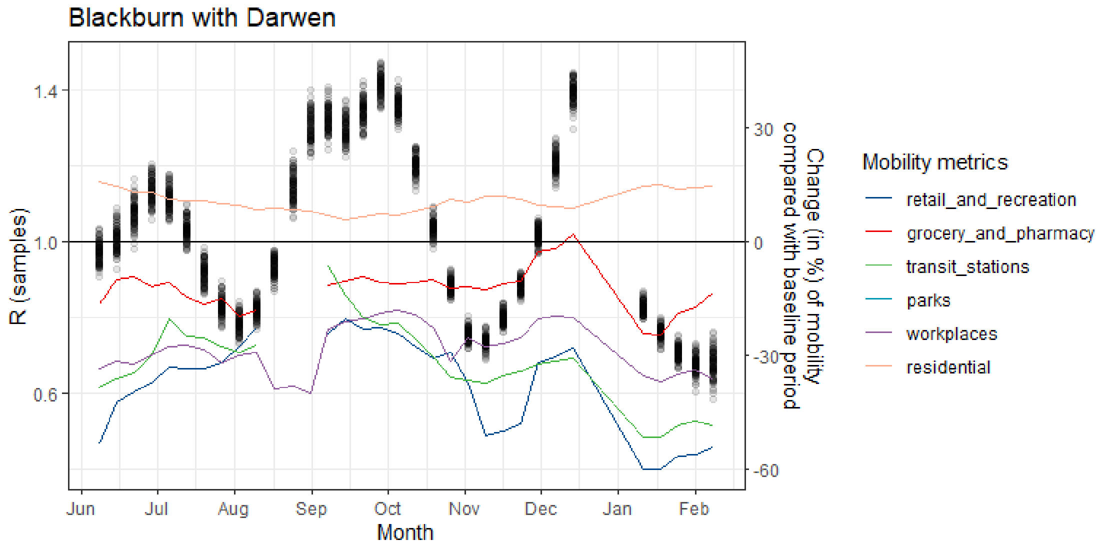

Blackburn with Darwen
1.4
1.0
0.6
R (samples)
30
0
-30
-60
Change (in %) of mobility compared with baseline period
Jun
Jul
Aug
Sep
Oct
Nov
Dec
Jan
Feb
Month
Mobility metrics
retail_and_recreation
grocery_and_pharmacy
transit_stations
parks
workplaces
residential

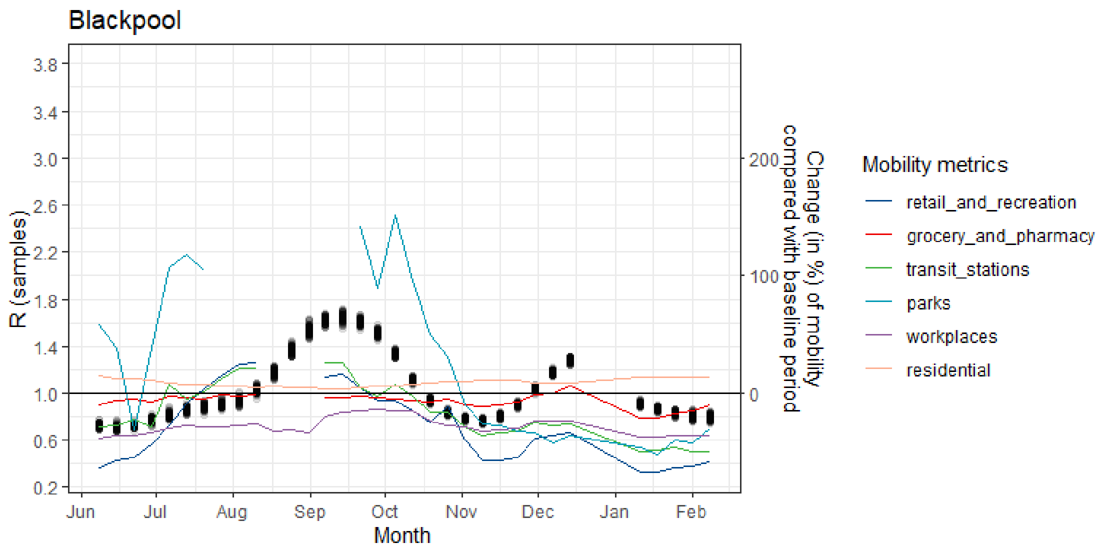

Blackpool
3.8
3.4
3.0
2.6
2.2
1.8
1.4
1.0
0.6
0.2
R (samples)
200
100
0
Change (in %) of mobility compared with baseline period
Jun
Jul
Aug
Sep
Oct
Nov
Dec
Jan
Feb
Month
Mobility metrics
retail_and_recreation
grocery_and_pharmacy
transit_stations
parks
workplaces
residential

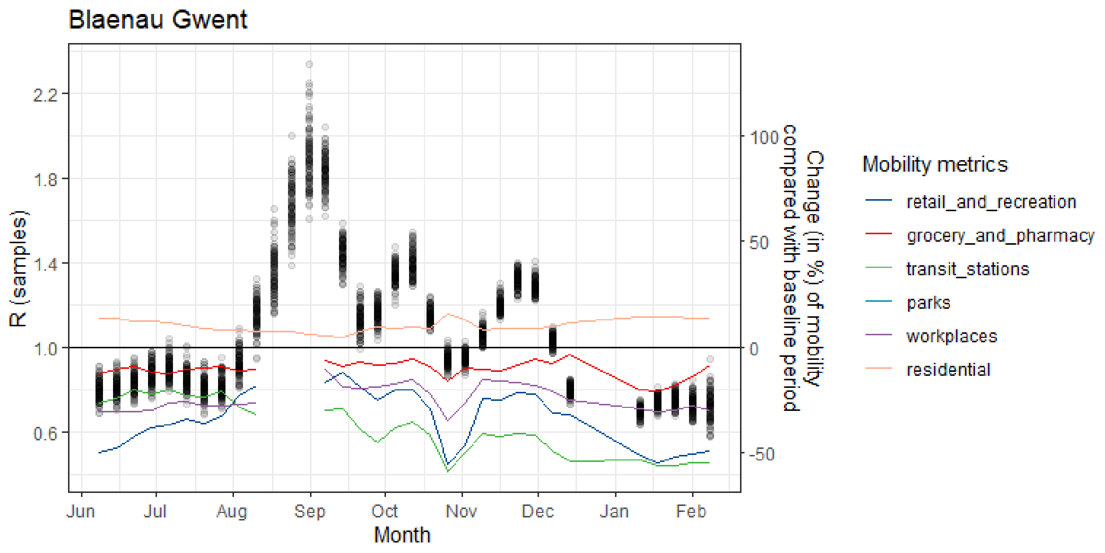
Blaenau Gwent
R (samples)
2.2
1.8
1.4
1.0
0.6
Jun
Jul
Aug
Sep
Oct
Nov
Dec
Jan
Feb
Month
100
50
0
-50
Change (in %) of mobility compared with baseline period
Mobility metrics
retail_and_recreation
grocery_and_pharmacy
transit_stations
parks
workplaces
residential

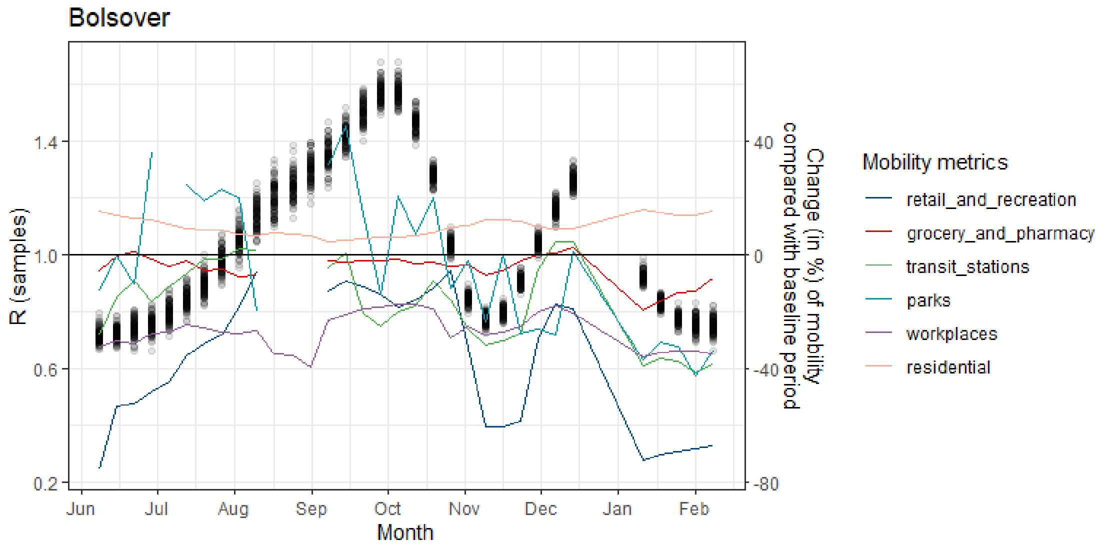
Bolsover
R (samples)
1.4
1.0
0.6
0.2
Jun
Jul
Aug
Sep
Oct
Nov
Dec
Jan
Feb
Month
40
0
-40
-80
Change (in %) of mobility compared with baseline period
Mobility metrics
retail_and_recreation
grocery_and_pharmacy
transit_stations
parks
workplaces
residential

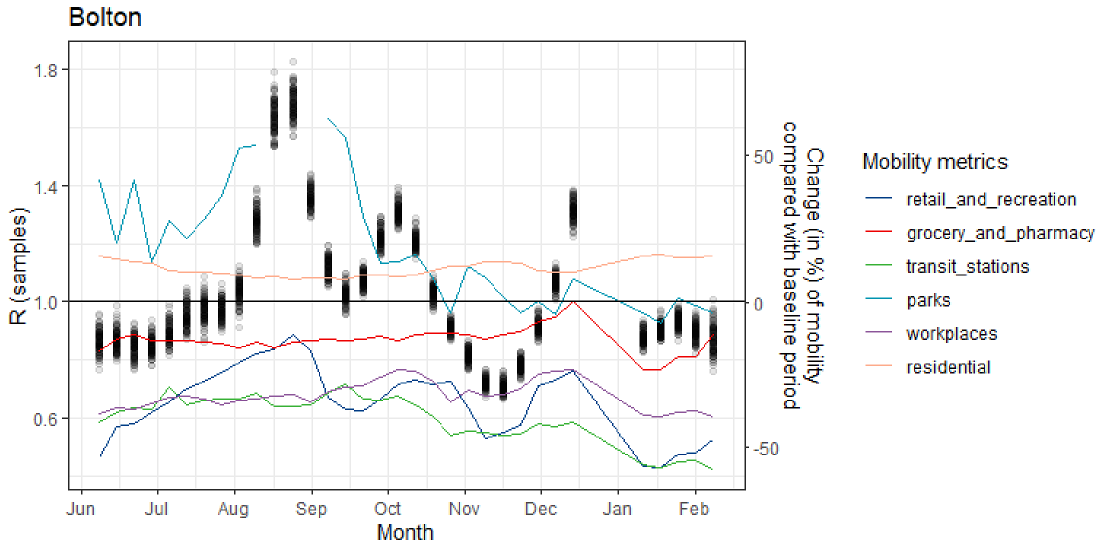
Bolton
R (samples)
1.8
1.4
1.0
0.6
Jun
Jul
Aug
Sep
Oct
Nov
Dec
Jan
Feb
Month
50
0
-50
Change (in %) of mobility compared with baseline period
Mobility metrics
retail_and_recreation
grocery_and_pharmacy
transit_stations
parks
workplaces
residential

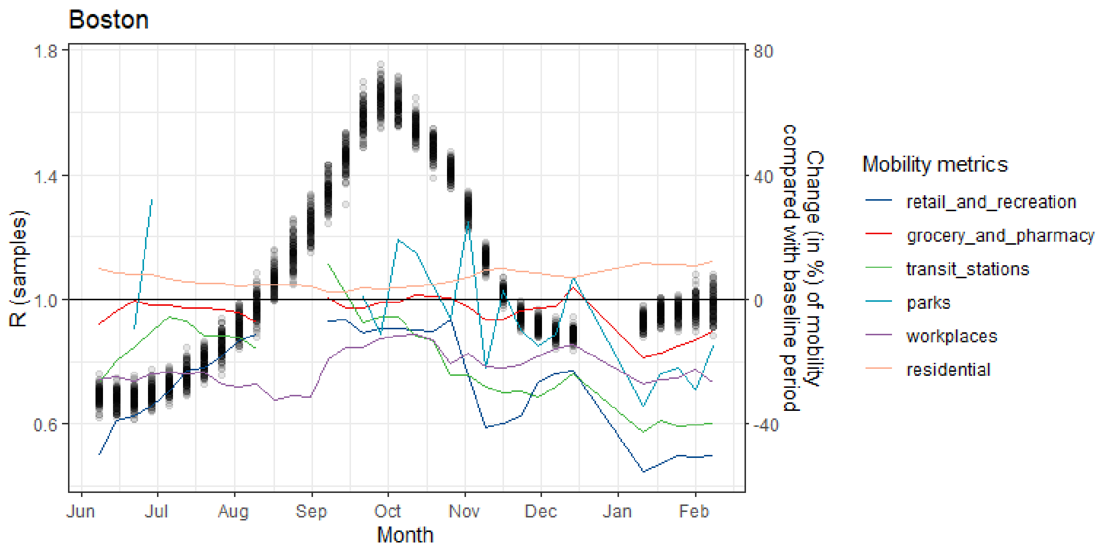
Boston
R (samples)
Change (in %) of mobility compared with baseline period
Month
Jun
Jul
Aug
Sep
Oct
Nov
Dec
Jan
Feb
Mobility metrics
retail_and_recreation
grocery_and_pharmacy
transit_stations
parks
workplaces
residential

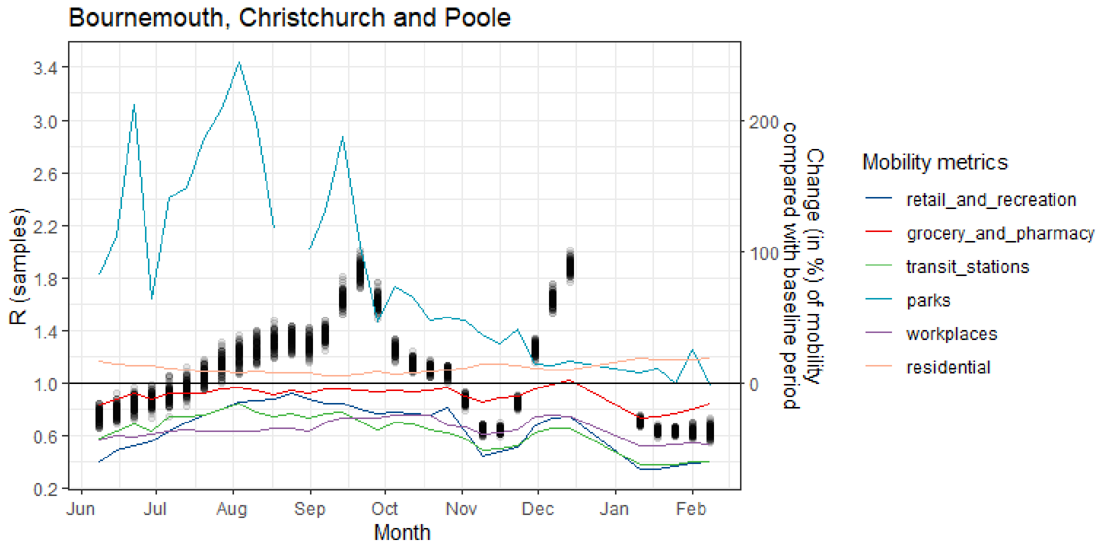
Bournemouth, Christchurch and Poole
R (samples)
Change (in %) of mobility compared with baseline period
Month
Jun
Jul
Aug
Sep
Oct
Nov
Dec
Jan
Feb
Mobility metrics
retail_and_recreation
grocery_and_pharmacy
transit_stations
parks
workplaces
residential

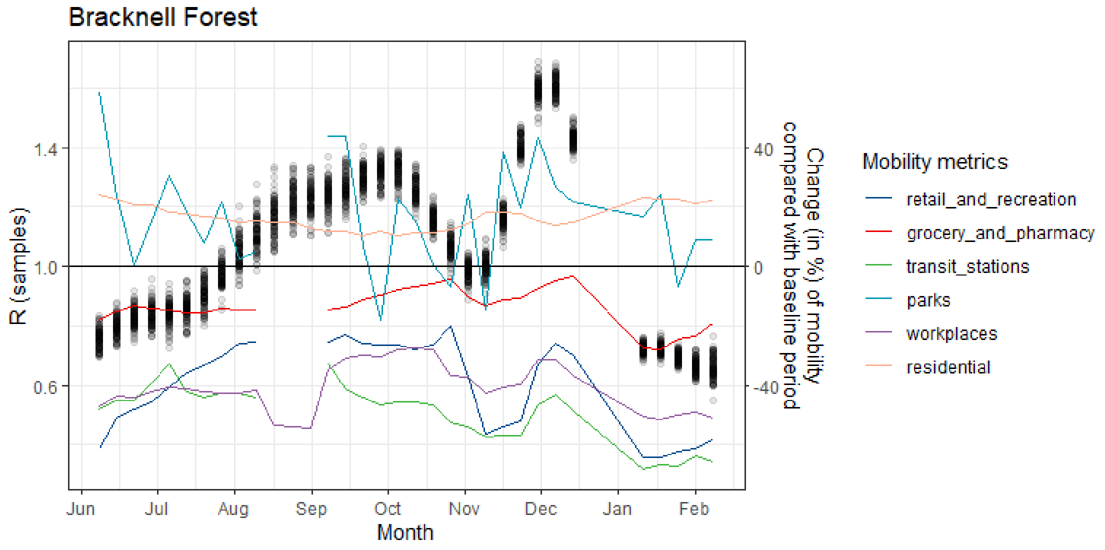
Bracknell Forest
R (samples)
Change (in %) of mobility compared with baseline period
Month
Jun
Jul
Aug
Sep
Oct
Nov
Dec
Jan
Feb
Mobility metrics
retail_and_recreation
grocery_and_pharmacy
transit_stations
parks
workplaces
residential

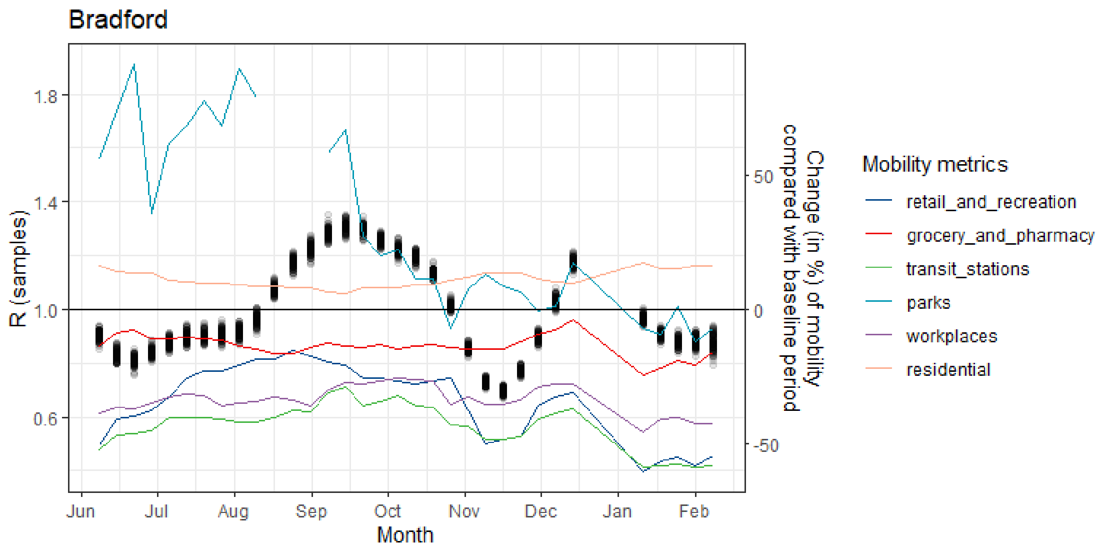

Bradford
1.8
1.4
1.0
0.6
R (samples)
50
0
-50
Change (in %) of mobility compared with baseline period
Jun
Jul
Aug
Sep
Oct
Nov
Dec
Jan
Feb
Month
Mobility metrics
retail_and_recreation
grocery_and_pharmacy
transit_stations
parks
workplaces
residential

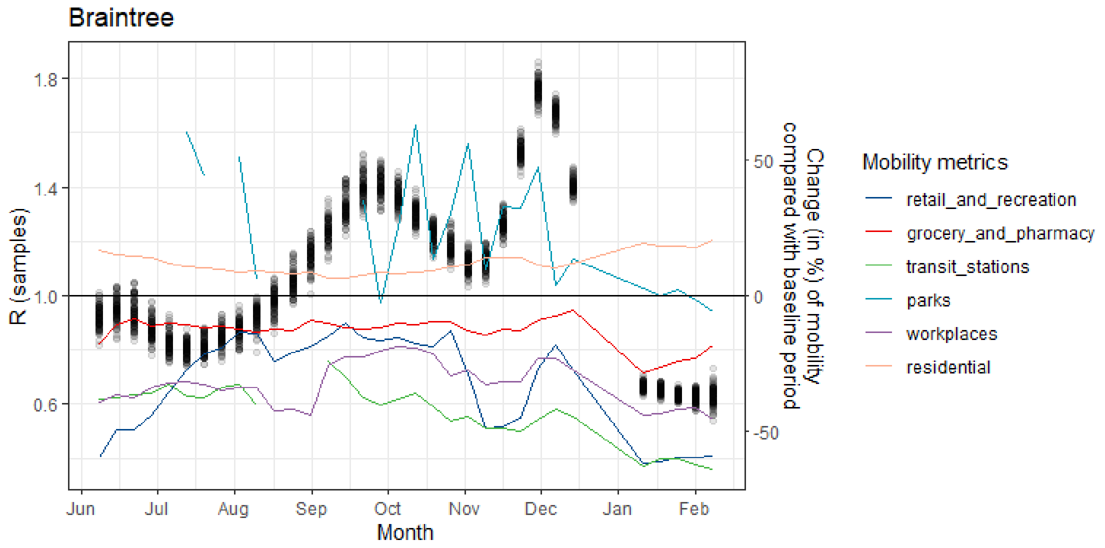

Braintree
1.8
1.4
1.0
0.6
R (samples)
50
0
-50
Change (in %) of mobility compared with baseline period
Jun
Jul
Aug
Sep
Oct
Nov
Dec
Jan
Feb
Month
Mobility metrics
retail_and_recreation
grocery_and_pharmacy
transit_stations
parks
workplaces
residential

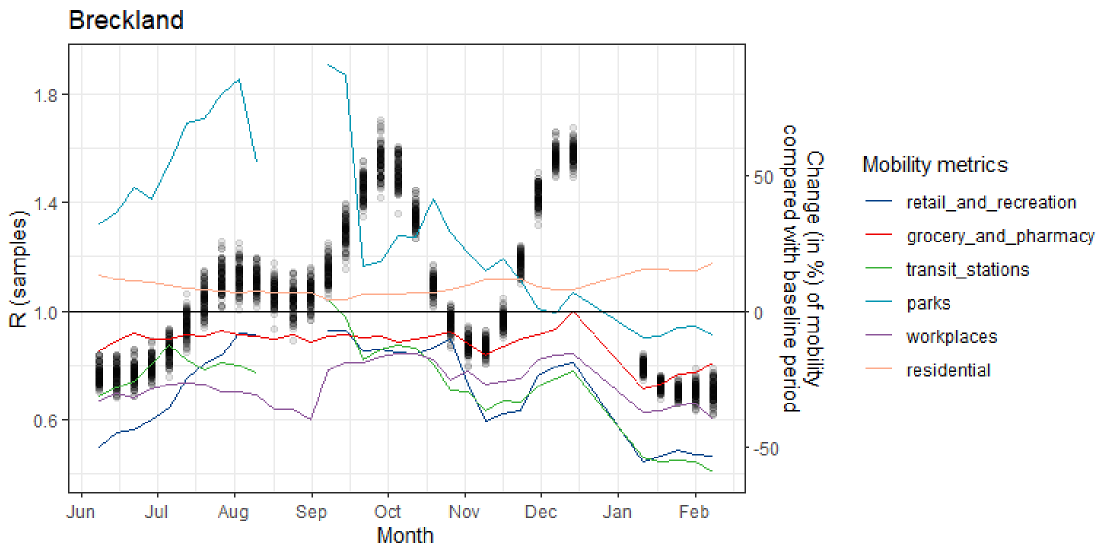

Breckland
1.8
1.4
1.0
0.6
R (samples)
50
0
-50
Change (in %) of mobility compared with baseline period
Jun
Jul
Aug
Sep
Oct
Nov
Dec
Jan
Feb
Month
Mobility metrics
retail_and_recreation
grocery_and_pharmacy
transit_stations
parks
workplaces
residential

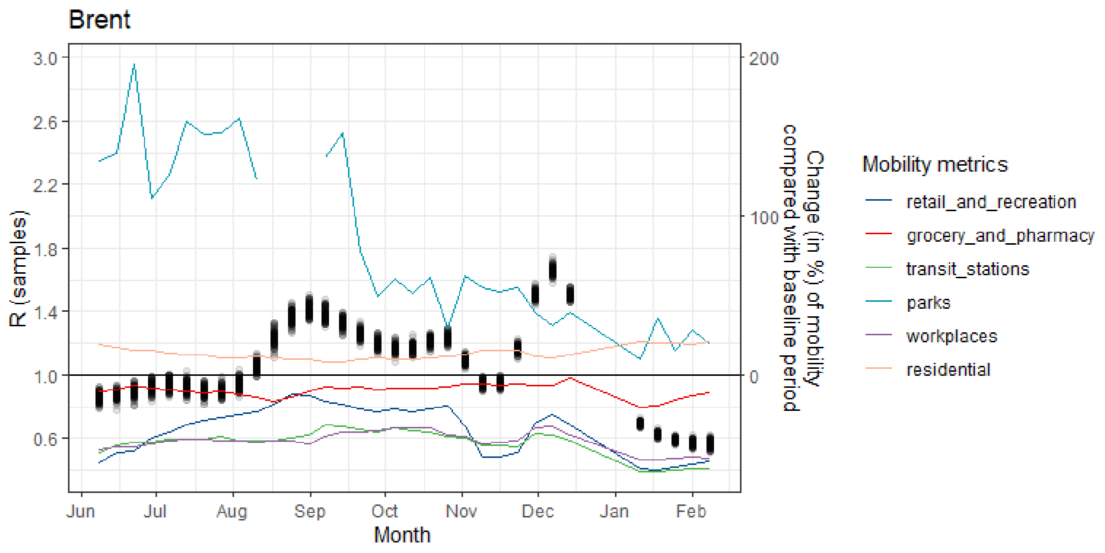
Brent
R (samples)
3.0
2.6
2.2
1.8
1.4
1.0
0.6
Jun
Jul
Aug
Sep
Oct
Nov
Dec
Jan
Feb
Month
200
100
0
Change (in %) of mobility compared with baseline period
Mobility metrics
retail_and_recreation
grocery_and_pharmacy
transit_stations
parks
workplaces
residential

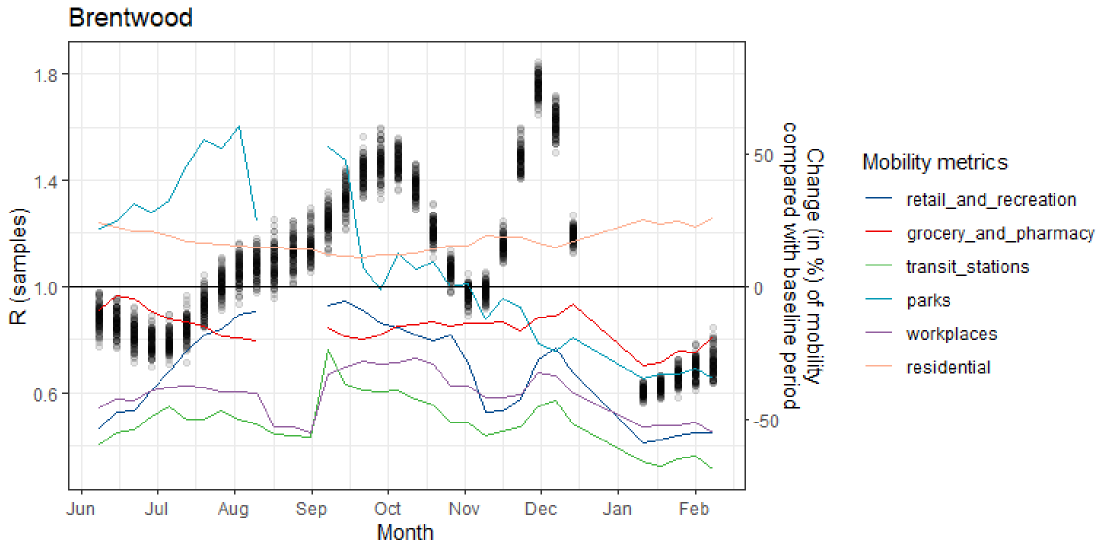
Brentwood
R (samples)
1.8
1.4
1.0
0.6
Jun
Jul
Aug
Sep
Oct
Nov
Dec
Jan
Feb
Month
50
0
-50
Change (in %) of mobility compared with baseline period
Mobility metrics
retail_and_recreation
grocery_and_pharmacy
transit_stations
parks
workplaces
residential

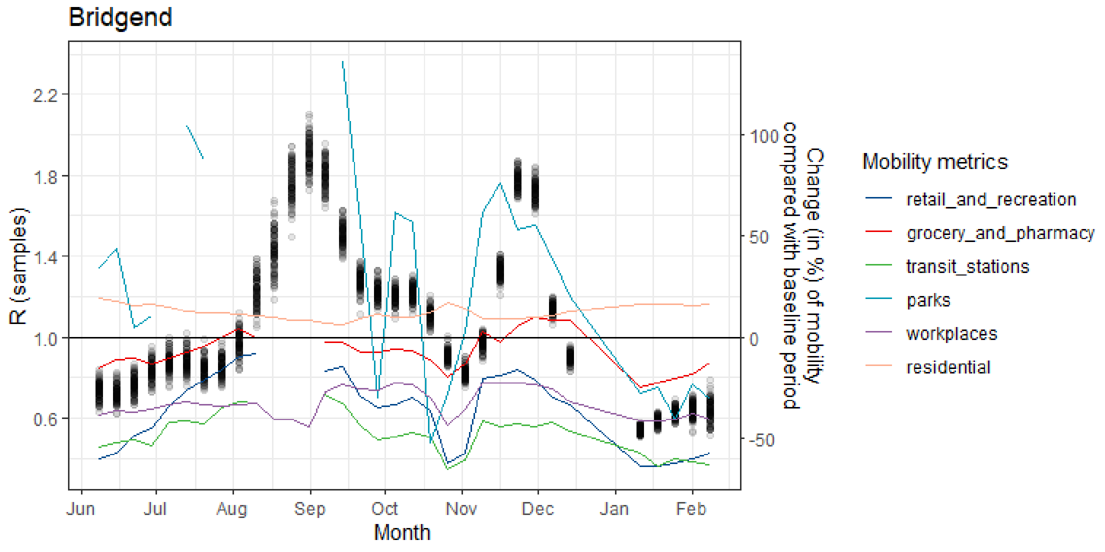
Bridgend
R (samples)
2.2
1.8
1.4
1.0
0.6
Jun
Jul
Aug
Sep
Oct
Nov
Dec
Jan
Feb
Month
100
50
0
-50
Change (in %) of mobility compared with baseline period
Mobility metrics
retail_and_recreation
grocery_and_pharmacy
transit_stations
parks
workplaces
residential

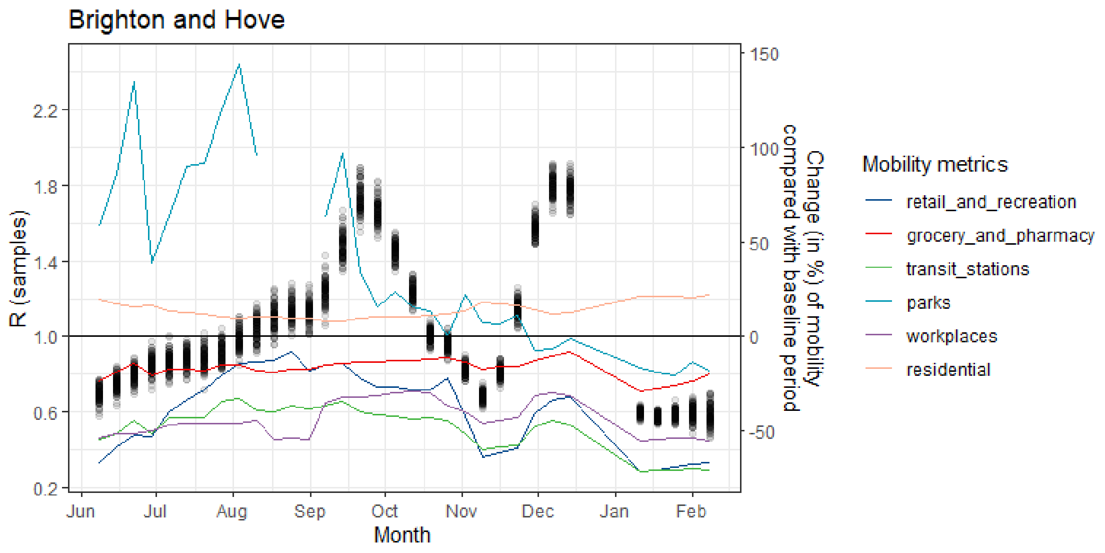
Brighton and Hove
R (samples)
Month
Change (in %) of mobility compared with baseline period
Jun
Jul
Aug
Sep
Oct
Nov
Dec
Jan
Feb
Mobility metrics
retail_and_recreation
grocery_and_pharmacy
transit_stations
parks
workplaces
residential

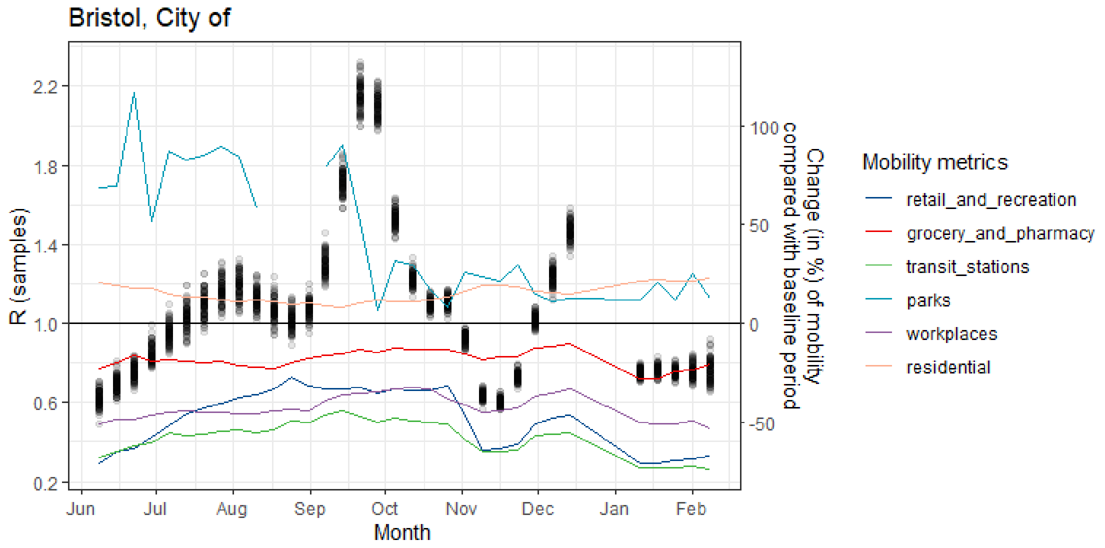
Bristol, City of
R (samples)
Month
Change (in %) of mobility compared with baseline period
Jun
Jul
Aug
Sep
Oct
Nov
Dec
Jan
Feb
Mobility metrics
retail_and_recreation
grocery_and_pharmacy
transit_stations
parks
workplaces
residential

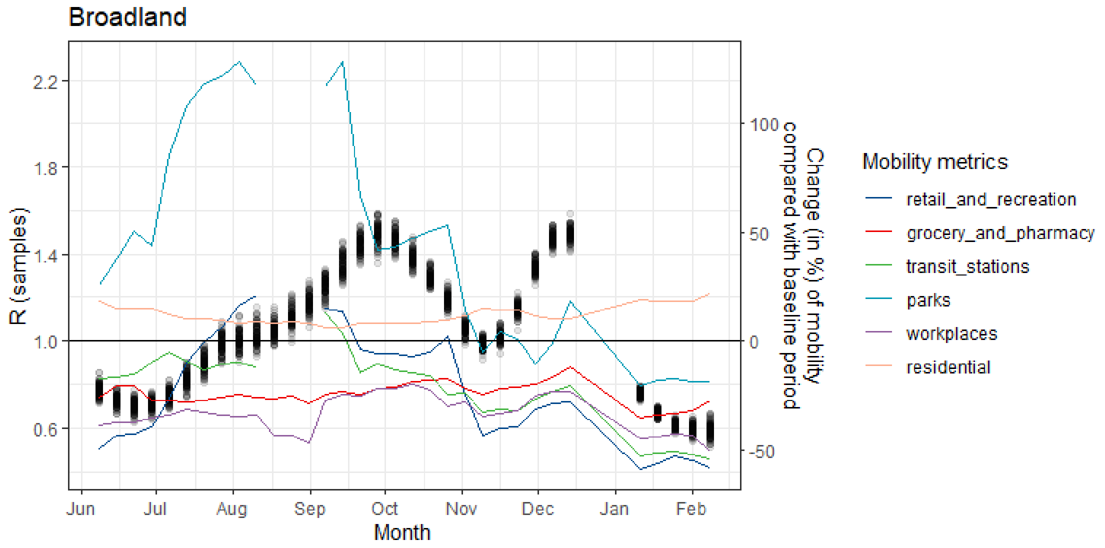
Broadland
R (samples)
Month
Change (in %) of mobility compared with baseline period
Jun
Jul
Aug
Sep
Oct
Nov
Dec
Jan
Feb
Mobility metrics
retail_and_recreation
grocery_and_pharmacy
transit_stations
parks
workplaces
residential

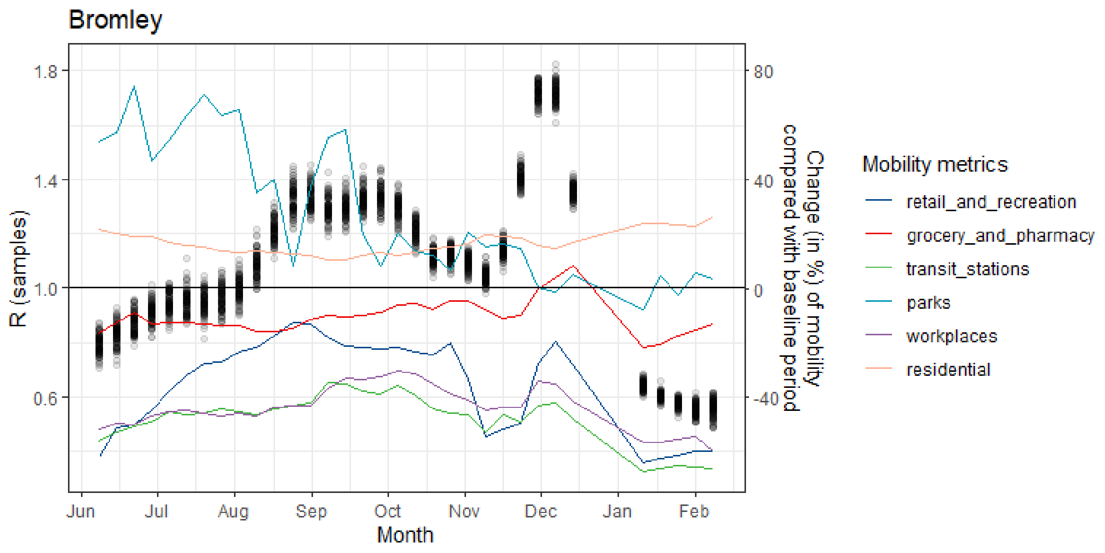
Bromley
R (samples)
1.8
1.4
1.0
0.6
80
40
0
-40
Change (in %) of mobility compared with baseline period
Jun Jul Aug Sep Oct Nov Dec Jan Feb
Month
Mobility metrics
retail_and_recreation
grocery_and_pharmacy
transit_stations
parks
workplaces
residential

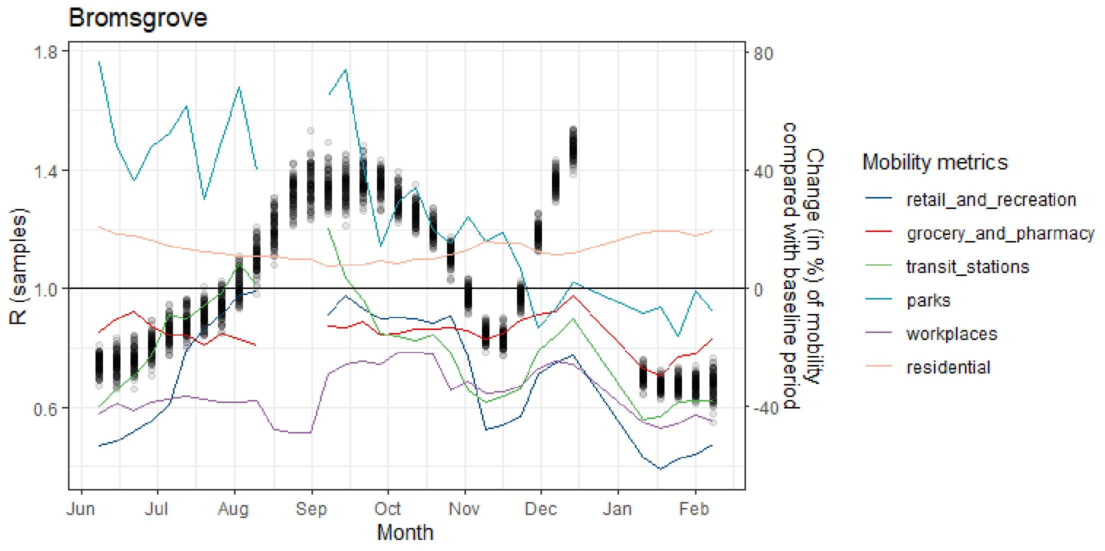
Bromsgrove
R (samples)
1.8
1.4
1.0
0.6
80
40
0
-40
Change (in %) of mobility compared with baseline period
Jun Jul Aug Sep Oct Nov Dec Jan Feb
Month
Mobility metrics
retail_and_recreation
grocery_and_pharmacy
transit_stations
parks
workplaces
residential

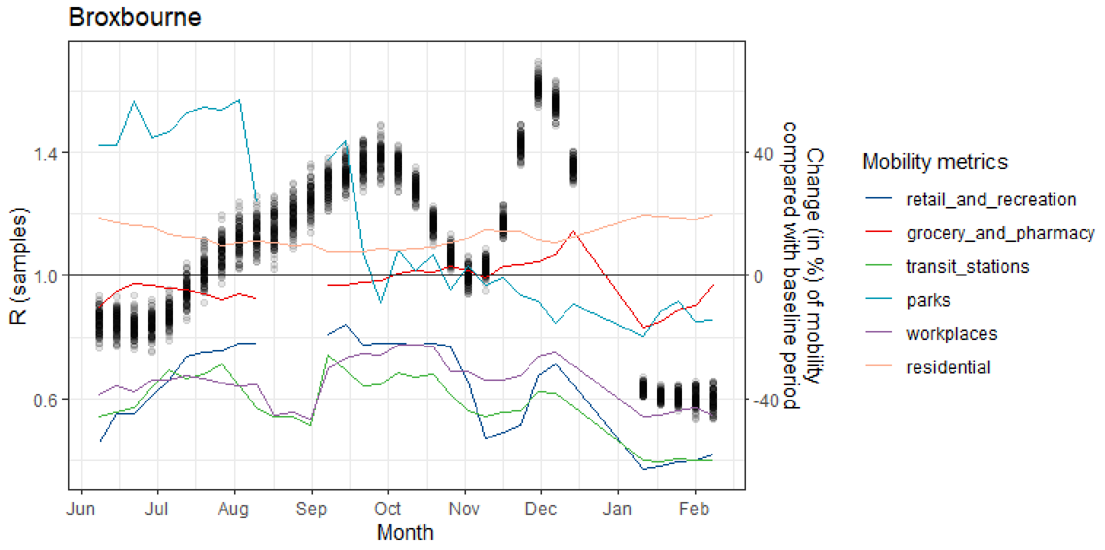
Broxbourne
R (samples)
1.4
1.0
0.6
40
0
-40
Change (in %) of mobility compared with baseline period
Jun Jul Aug Sep Oct Nov Dec Jan Feb
Month
Mobility metrics
retail_and_recreation
grocery_and_pharmacy
transit_stations
parks
workplaces
residential

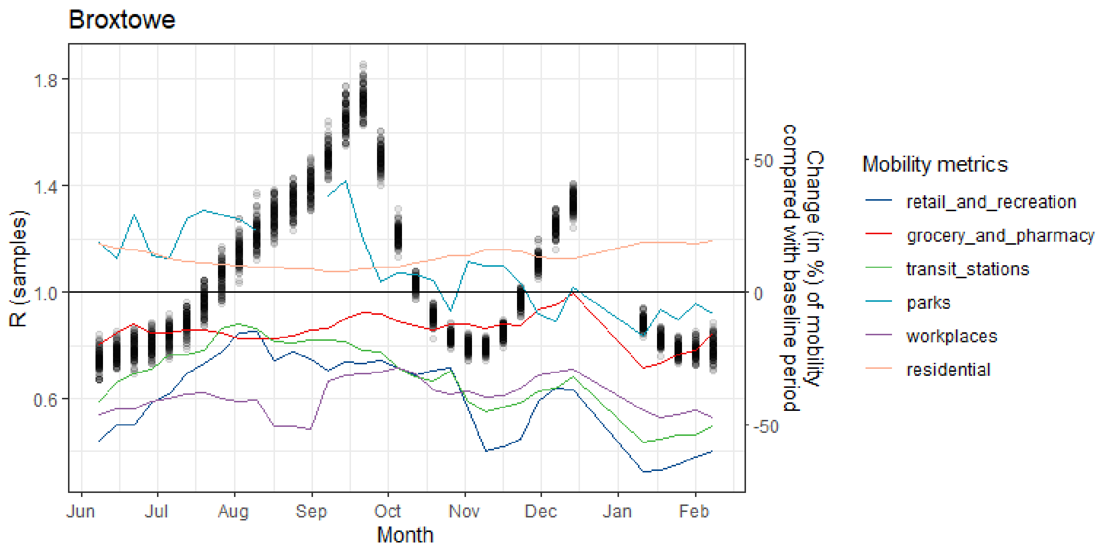
Broxtowe
R (samples)
Change (in %) of mobility compared with baseline period
Month
Mobility metrics
retail_and_recreation
grocery_and_pharmacy
transit_stations
parks
workplaces
residential

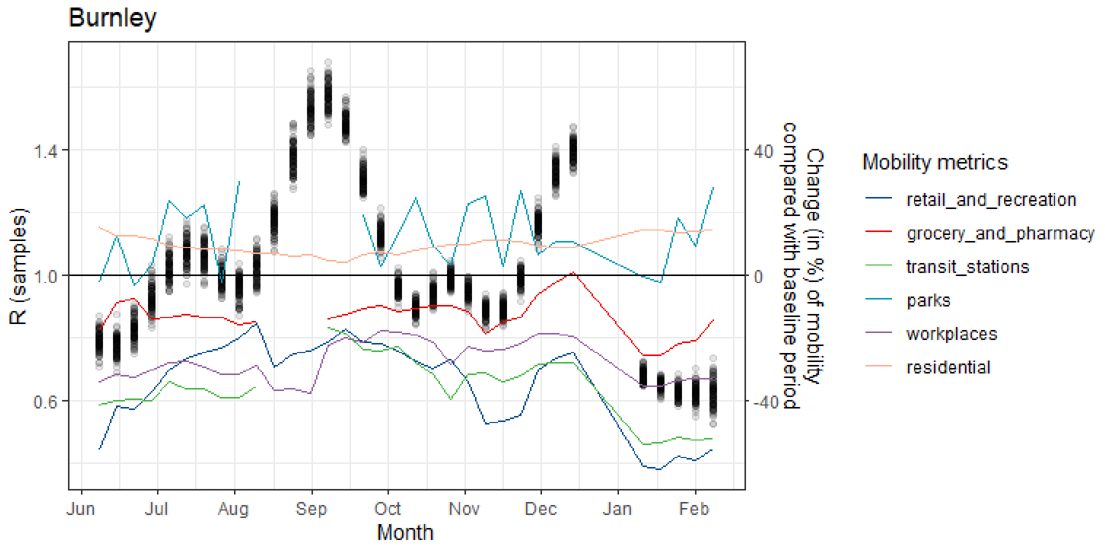
Burnley
R (samples)
Change (in %) of mobility compared with baseline period
Month
Mobility metrics
retail_and_recreation
grocery_and_pharmacy
transit_stations
parks
workplaces
residential

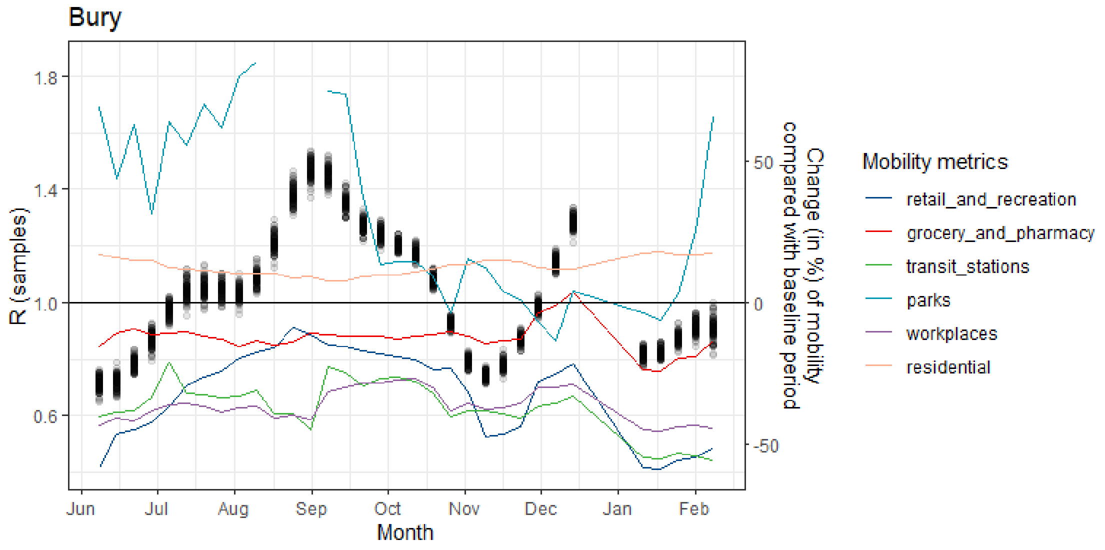
Bury
R (samples)
Change (in %) of mobility compared with baseline period
Month
Mobility metrics
retail_and_recreation
grocery_and_pharmacy
transit_stations
parks
workplaces
residential

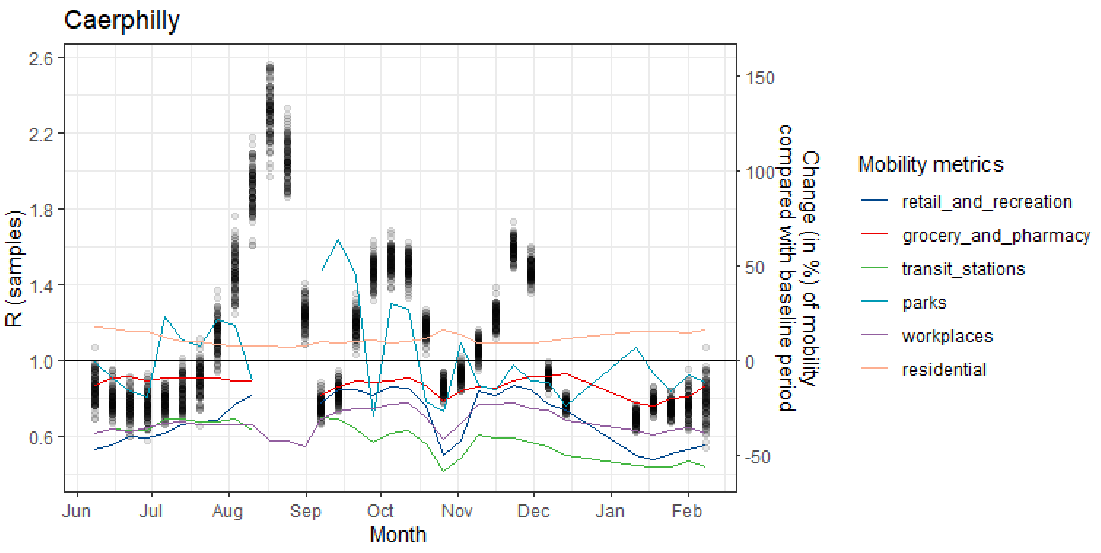
Caerphilly
R (samples)
2.6
2.2
1.8
1.4
1.0
0.6
150
100
50
0
-50
Change (in %) of mobility compared with baseline period
Jun
Jul
Aug
Sep
Oct
Nov
Dec
Jan
Feb
Month
Mobility metrics
retail_and_recreation
grocery_and_pharmacy
transit_stations
parks
workplaces
residential

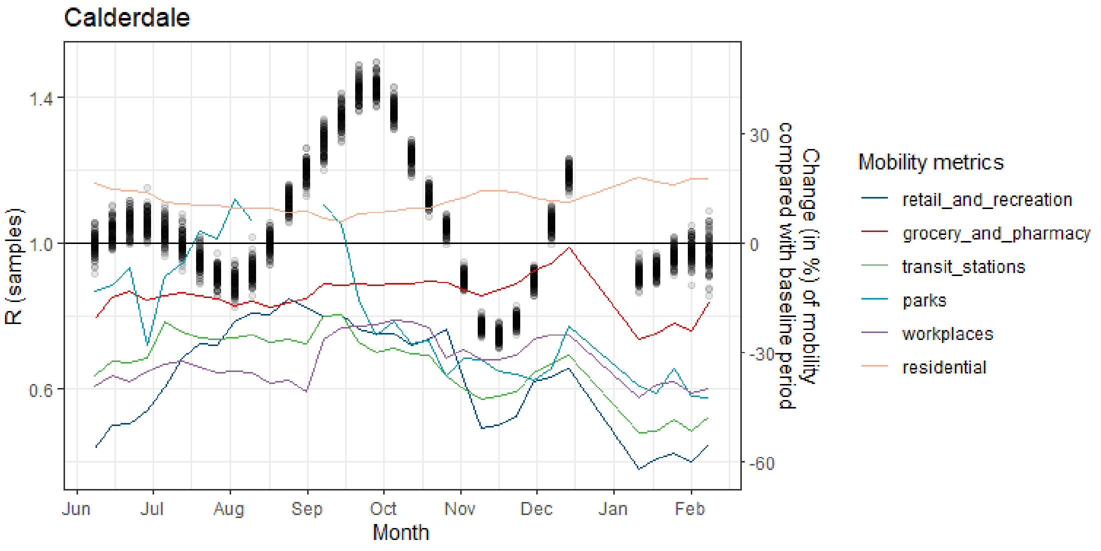
Calderdale
R (samples)
1.4
1.0
0.6
30
0
-30
-60
Change (in %) of mobility compared with baseline period
Jun
Jul
Aug
Sep
Oct
Nov
Dec
Jan
Feb
Month
Mobility metrics
retail_and_recreation
grocery_and_pharmacy
transit_stations
parks
workplaces
residential

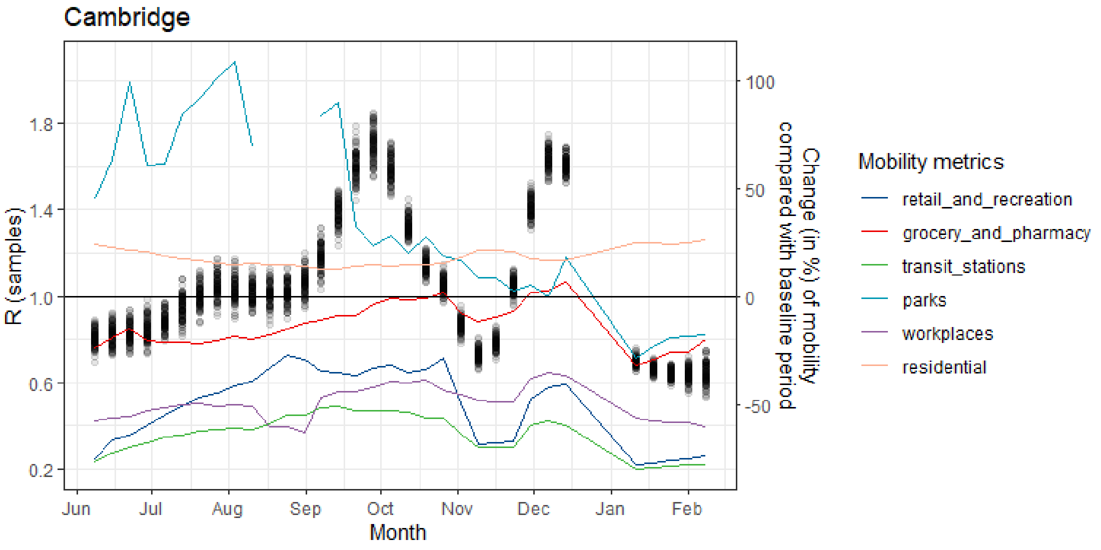
Cambridge
R (samples)
1.8
1.4
1.0
0.6
0.2
100
50
0
-50
Change (in %) of mobility compared with baseline period
Jun
Jul
Aug
Sep
Oct
Nov
Dec
Jan
Feb
Month
Mobility metrics
retail_and_recreation
grocery_and_pharmacy
transit_stations
parks
workplaces
residential

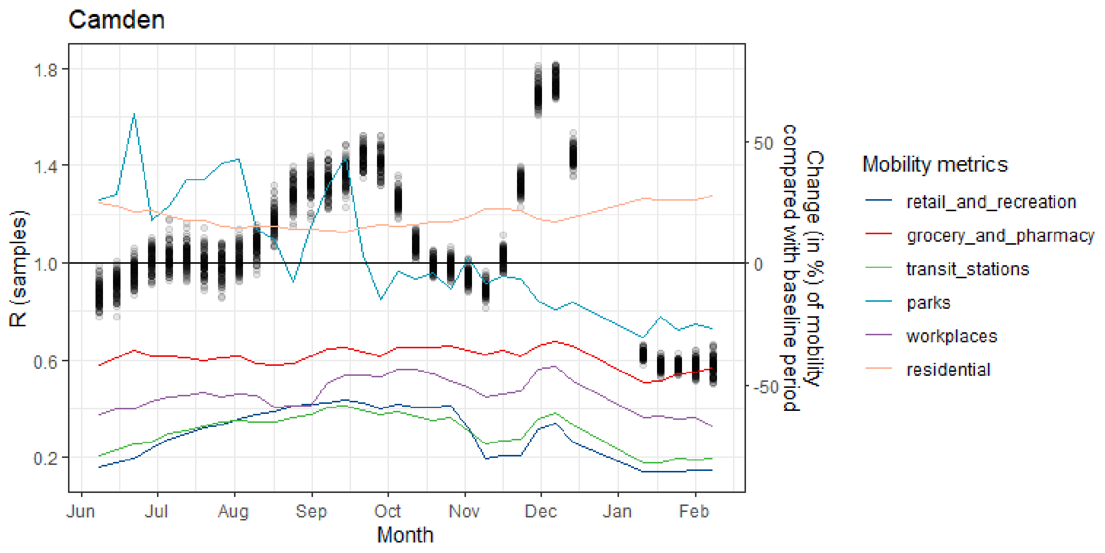
Camden
R (samples)
1.8
1.4
1.0
0.6
0.2
50
0
-50
Change (in %) of mobility compared with baseline period
Jun
Jul
Aug
Sep
Oct
Nov
Dec
Jan
Feb
Month
Mobility metrics
retail_and_recreation
grocery_and_pharmacy
transit_stations
parks
workplaces
residential

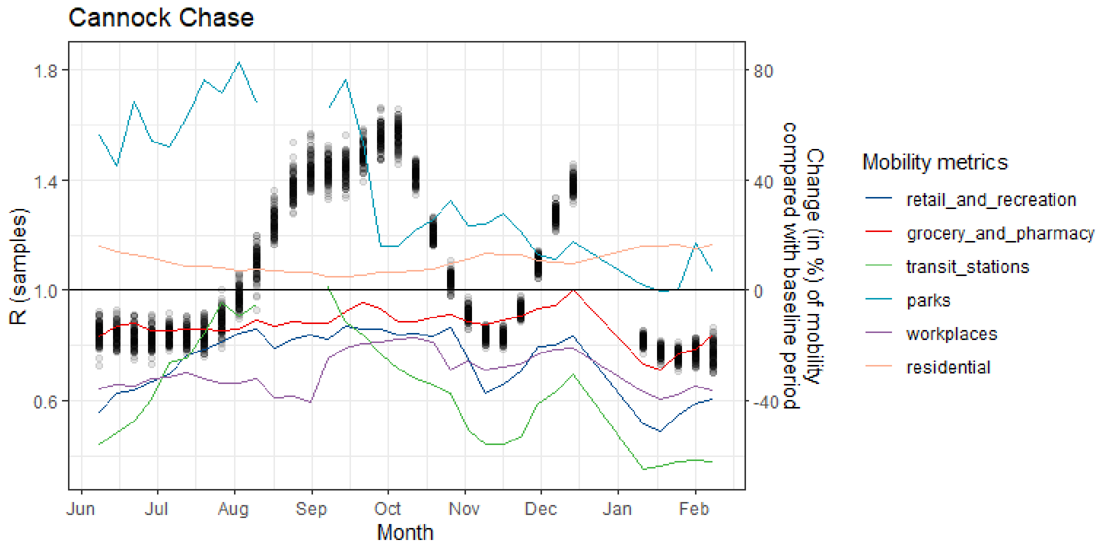
Cannock Chase
R (samples)
1.8
1.4
1.0
0.6
80
40
0
-40
Change (in %) of mobility compared with baseline period
Jun
Jul
Aug
Sep
Oct
Nov
Dec
Jan
Feb
Month
Mobility metrics
retail_and_recreation
grocery_and_pharmacy
transit_stations
parks
workplaces
residential

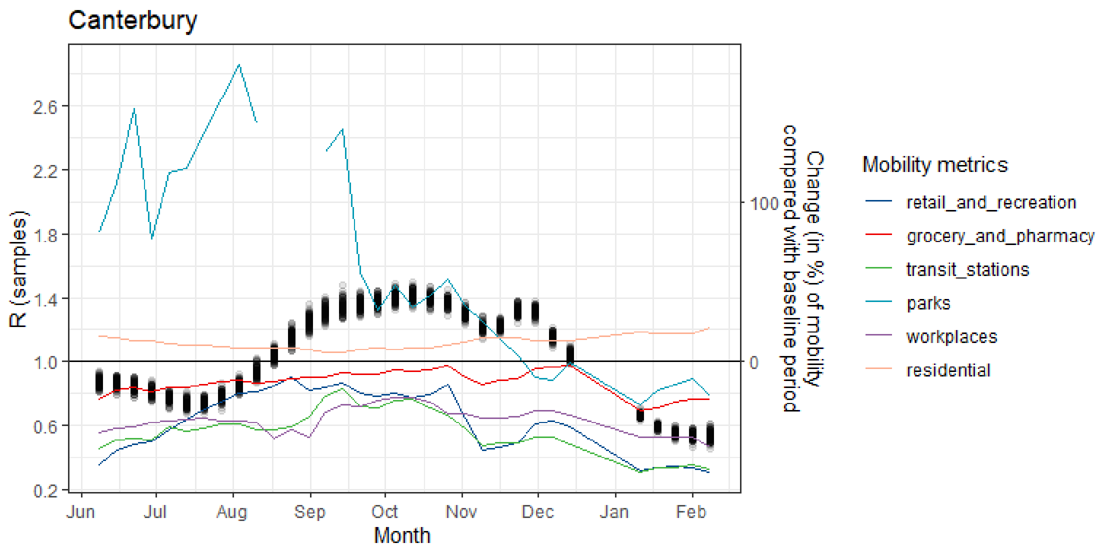
Canterbury
R (samples)
2.6
2.2
1.8
1.4
1.0
0.6
0.2
100
0
Change (in %) of mobility compared with baseline period
Jun
Jul
Aug
Sep
Oct
Nov
Dec
Jan
Feb
Month
Mobility metrics
retail_and_recreation
grocery_and_pharmacy
transit_stations
parks
workplaces
residential

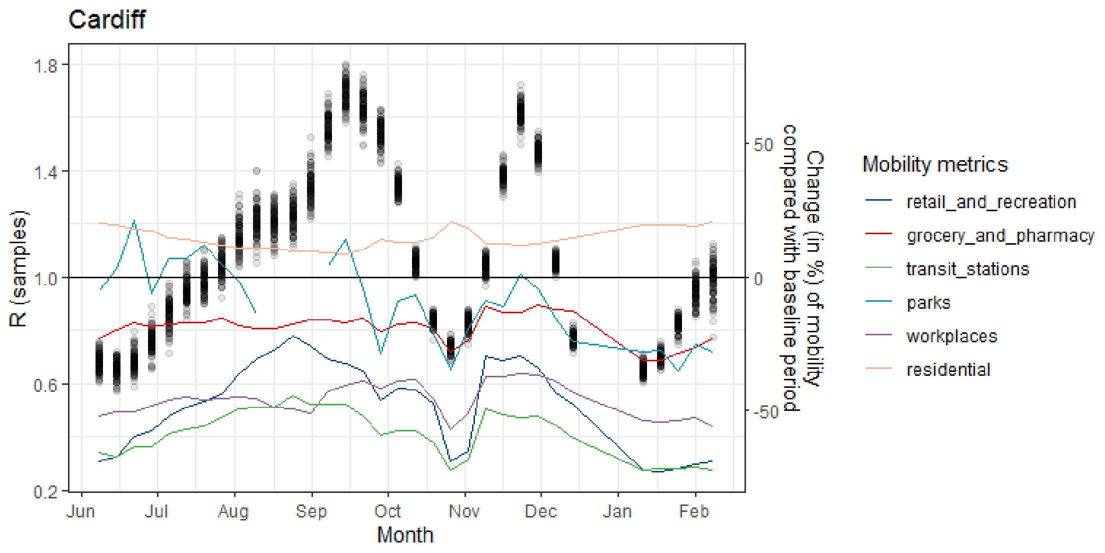

Cardiff
R (samples)
1.8
1.4
1.0
0.6
0.2
Jun
Jul
Aug
Sep
Oct
Nov
Dec
Jan
Feb
Month
50
0
-50
Change (in %) of mobility compared with baseline period
Mobility metrics
retail_and_recreation
grocery_and_pharmacy
transit_stations
parks
workplaces
residential

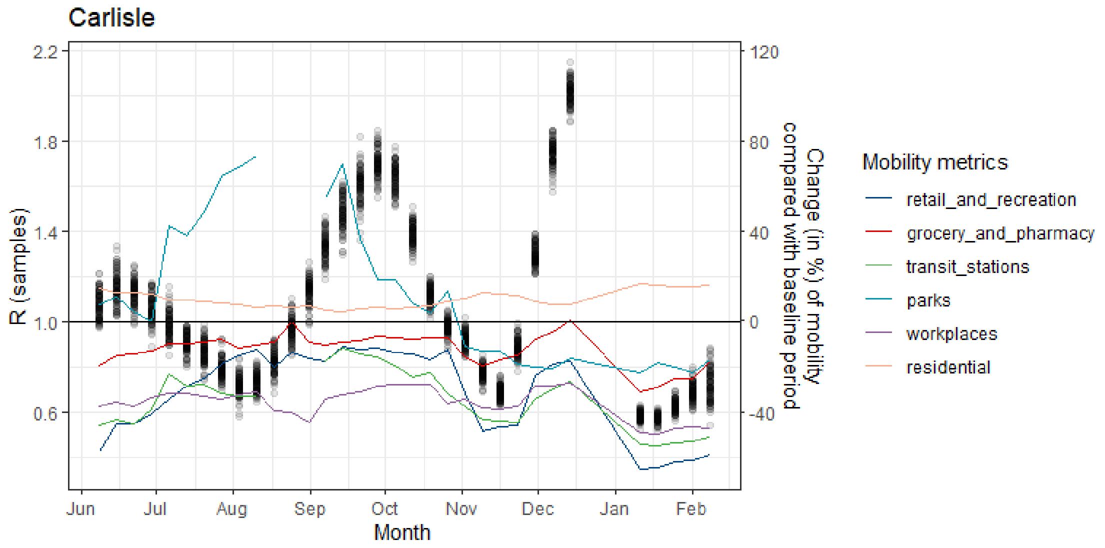

Carlisle
R (samples)
2.2
1.8
1.4
1.0
0.6
Jun
Jul
Aug
Sep
Oct
Nov
Dec
Jan
Feb
Month
120
80
40
0
-40
Change (in %) of mobility compared with baseline period
Mobility metrics
retail_and_recreation
grocery_and_pharmacy
transit_stations
parks
workplaces
residential

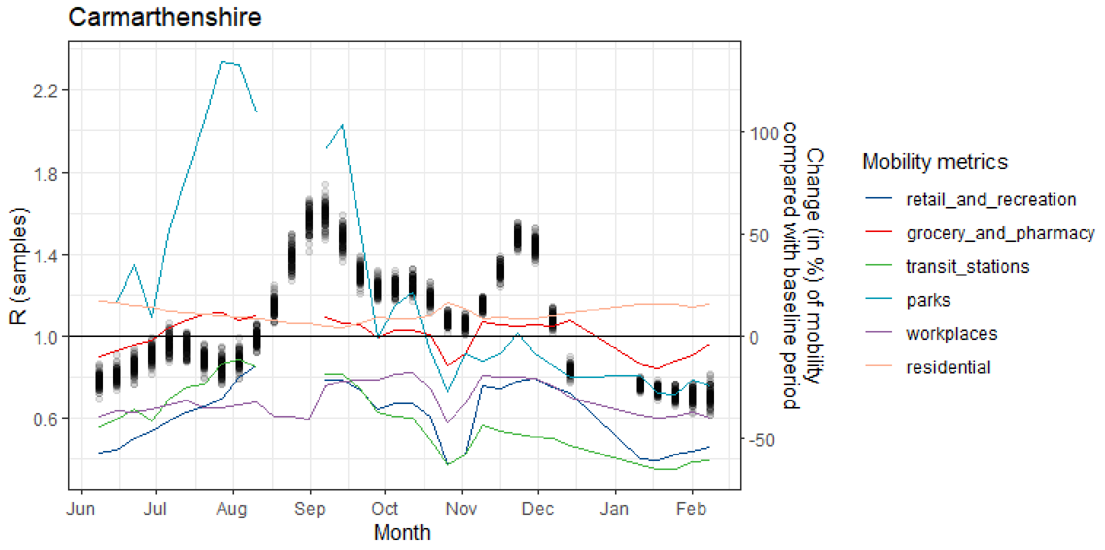

Carmarthenshire
R (samples)
2.2
1.8
1.4
1.0
0.6
Jun
Jul
Aug
Sep
Oct
Nov
Dec
Jan
Feb
Month
100
50
0
-50
Change (in %) of mobility compared with baseline period
Mobility metrics
retail_and_recreation
grocery_and_pharmacy
transit_stations
parks
workplaces
residential

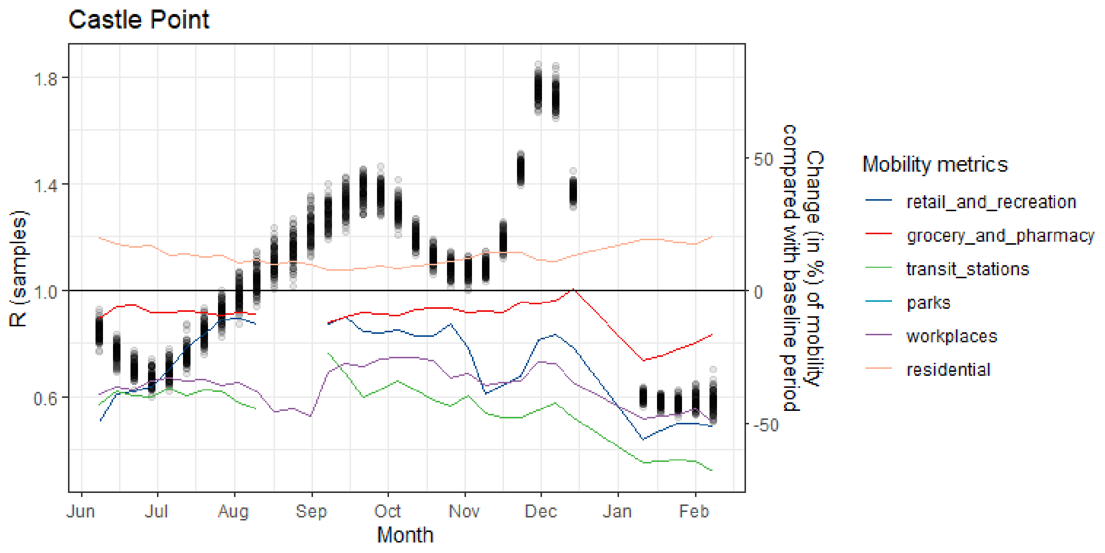
Castle Point
R (samples)
1.8
1.4
1.0
0.6
Jun
Jul
Aug
Sep
Oct
Nov
Dec
Jan
Feb
Month
50
0
-50
Change (in %) of mobility compared with baseline period
Mobility metrics
retail_and_recreation
grocery_and_pharmacy
transit_stations
parks
workplaces
residential

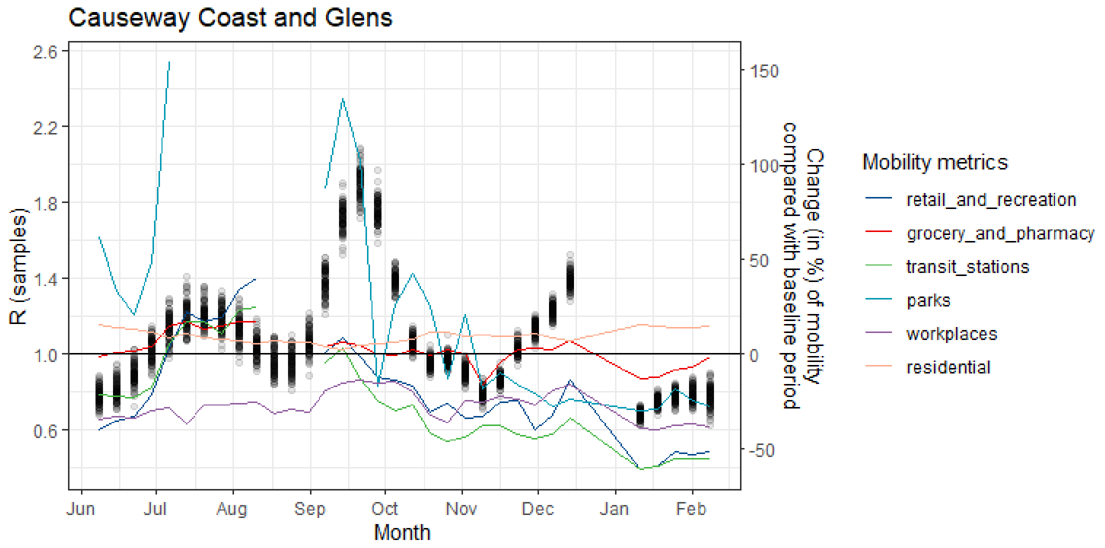
Causeway Coast and Glens
R (samples)
2.6
2.2
1.8
1.4
1.0
0.6
Jun
Jul
Aug
Sep
Oct
Nov
Dec
Jan
Feb
Month
150
100
50
0
-50
Change (in %) of mobility compared with baseline period
Mobility metrics
retail_and_recreation
grocery_and_pharmacy
transit_stations
parks
workplaces
residential

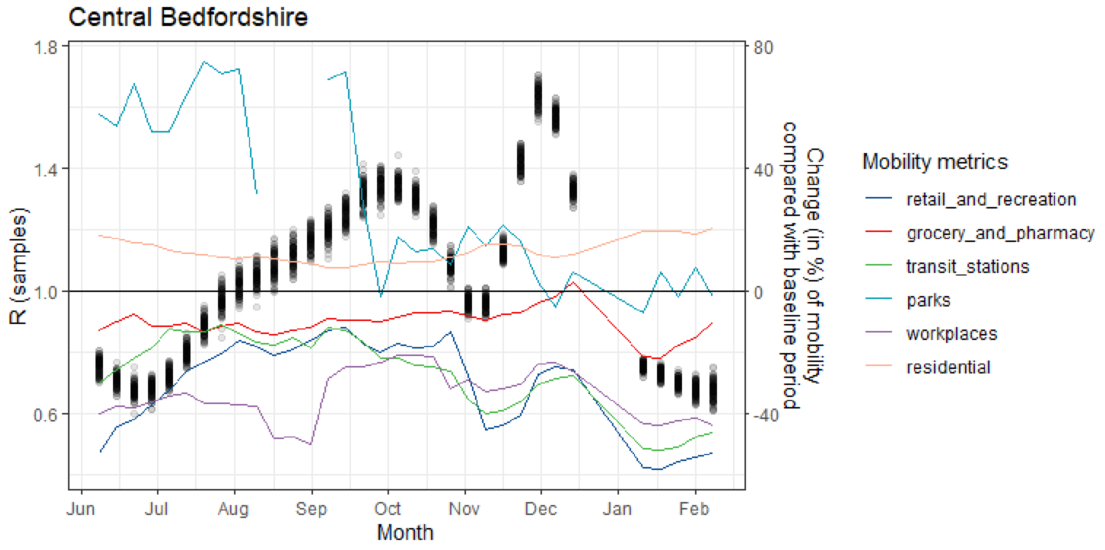
Central Bedfordshire
R (samples)
1.8
1.4
1.0
0.6
Jun
Jul
Aug
Sep
Oct
Nov
Dec
Jan
Feb
Month
80
40
0
-40
Change (in %) of mobility compared with baseline period
Mobility metrics
retail_and_recreation
grocery_and_pharmacy
transit_stations
parks
workplaces
residential

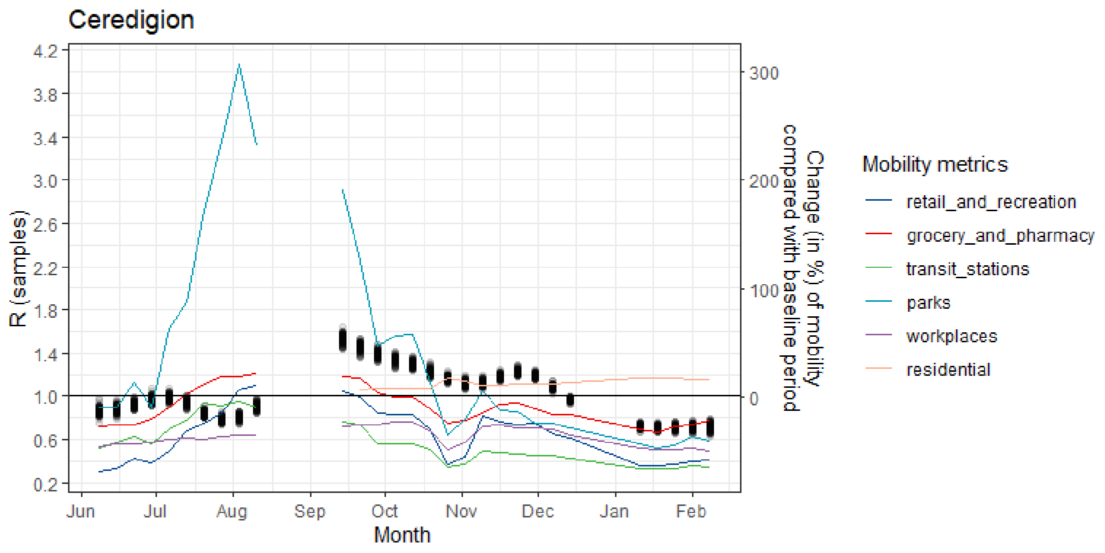
Ceredigion
4.2
3.8
3.4
3.0
2.6
2.2
1.8
1.4
1.0
0.6
0.2
R (samples)
300
200
100
0
Change (in %) of mobility compared with baseline period
Jun
Jul
Aug
Sep
Oct
Nov
Dec
Jan
Feb
Month
Mobility metrics
retail_and_recreation
grocery_and_pharmacy
transit_stations
parks
workplaces
residential

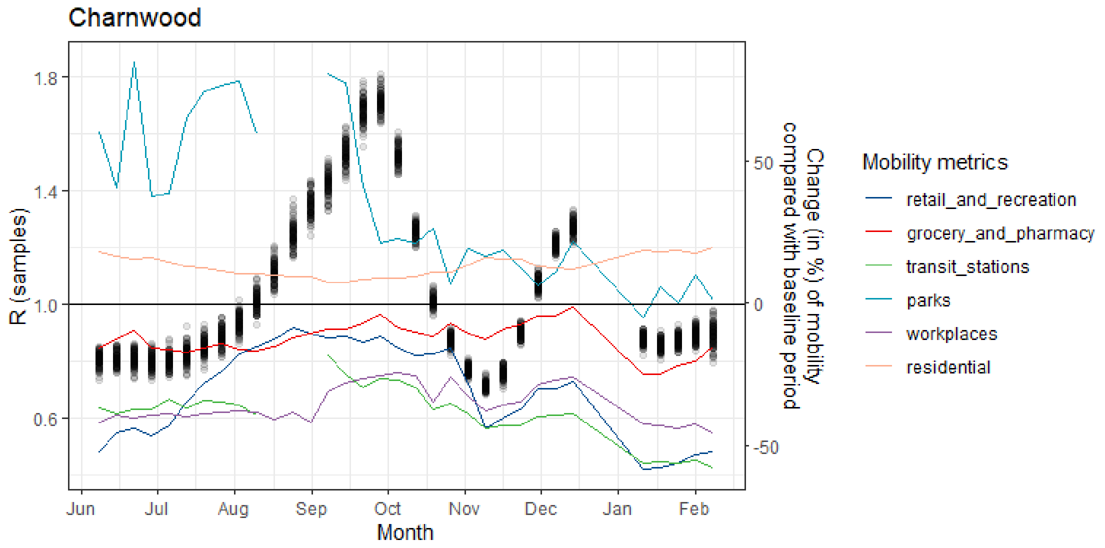
Charnwood
1.8
1.4
1.0
0.6
R (samples)
50
0
-50
Change (in %) of mobility compared with baseline period
Jun
Jul
Aug
Sep
Oct
Nov
Dec
Jan
Feb
Month
Mobility metrics
retail_and_recreation
grocery_and_pharmacy
transit_stations
parks
workplaces
residential

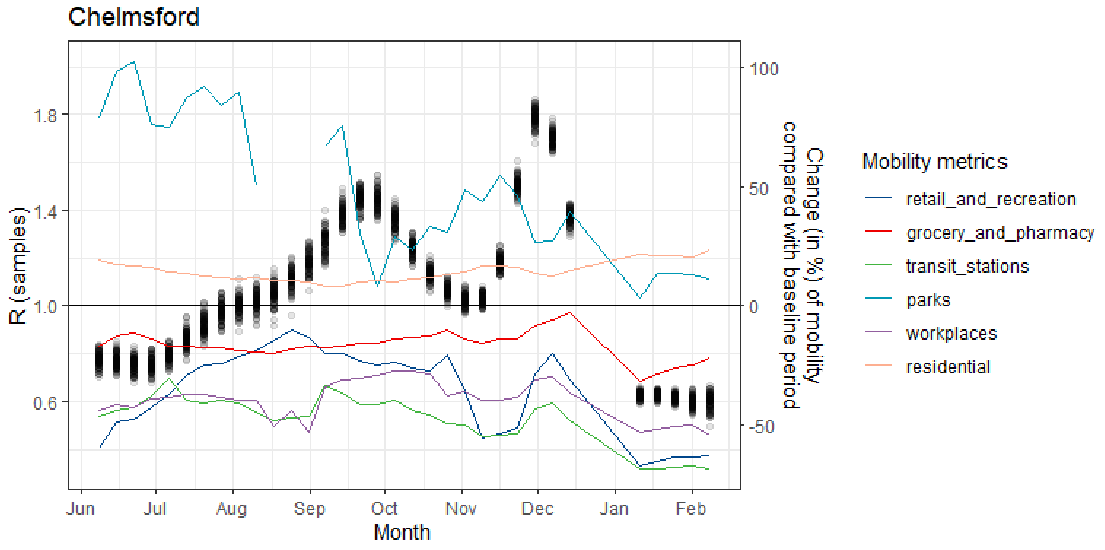
Chelmsford
1.8
1.4
1.0
0.6
R (samples)
100
50
0
-50
Change (in %) of mobility compared with baseline period
Jun
Jul
Aug
Sep
Oct
Nov
Dec
Jan
Feb
Month
Mobility metrics
retail_and_recreation
grocery_and_pharmacy
transit_stations
parks
workplaces
residential

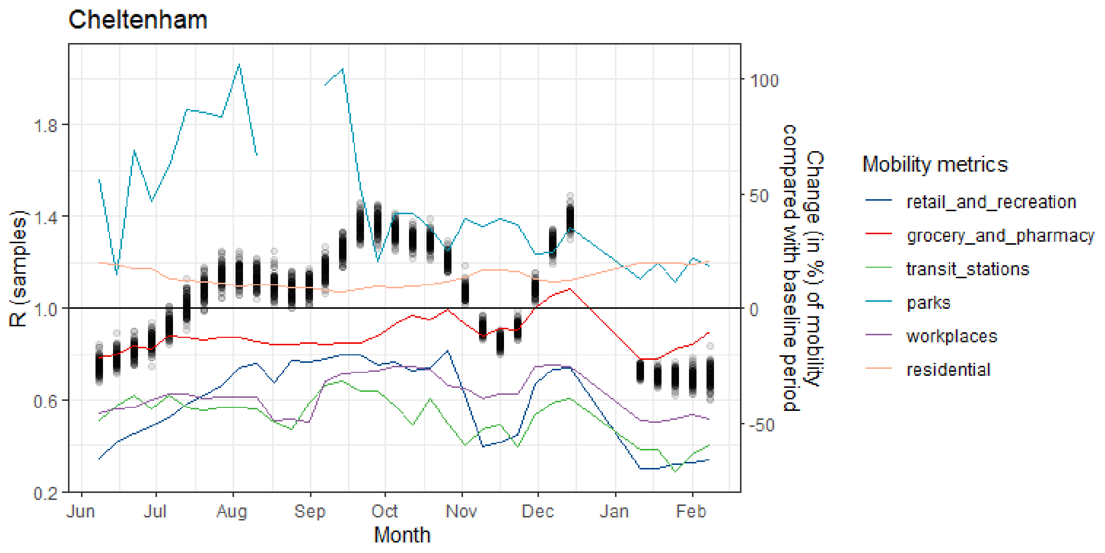
Cheltenham
R (samples)
1.8
1.4
1.0
0.6
0.2
100
50
0
-50
Change (in %) of mobility compared with baseline period
Jun
Jul
Aug
Sep
Oct
Nov
Dec
Jan
Feb
Month
Mobility metrics
retail_and_recreation
grocery_and_pharmacy
transit_stations
parks
workplaces
residential

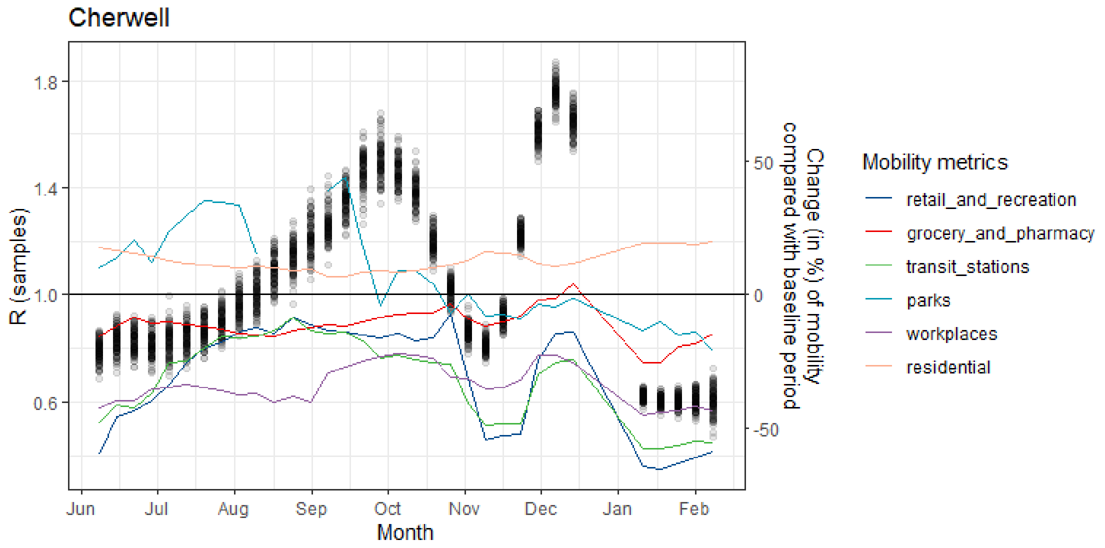
Cherwell
R (samples)
1.8
1.4
1.0
0.6
50
0
-50
Change (in %) of mobility compared with baseline period
Jun
Jul
Aug
Sep
Oct
Nov
Dec
Jan
Feb
Month
Mobility metrics
retail_and_recreation
grocery_and_pharmacy
transit_stations
parks
workplaces
residential

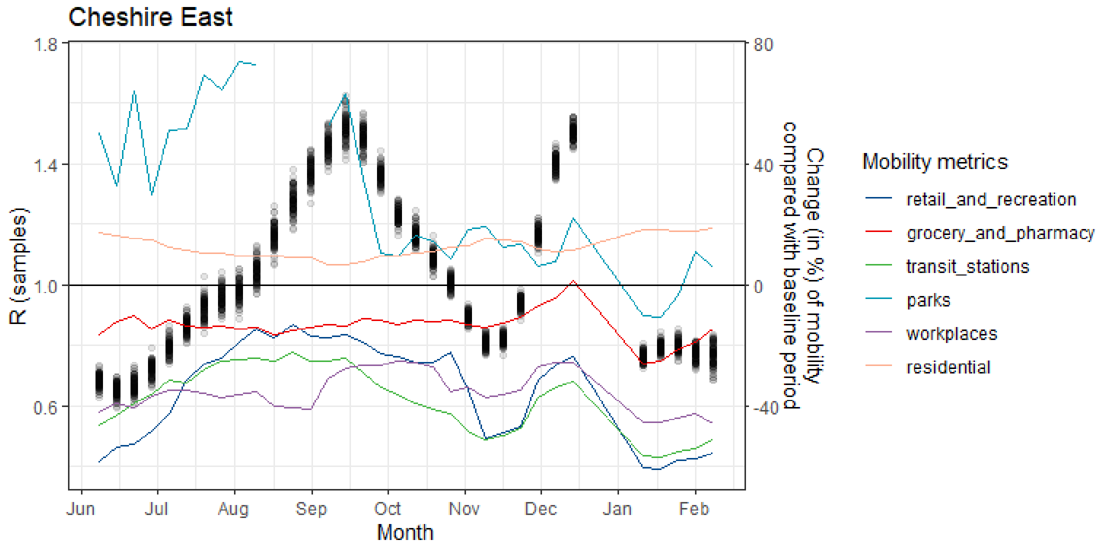
Cheshire East
R (samples)
1.8
1.4
1.0
0.6
80
40
0
-40
Change (in %) of mobility compared with baseline period
Jun
Jul
Aug
Sep
Oct
Nov
Dec
Jan
Feb
Month
Mobility metrics
retail_and_recreation
grocery_and_pharmacy
transit_stations
parks
workplaces
residential

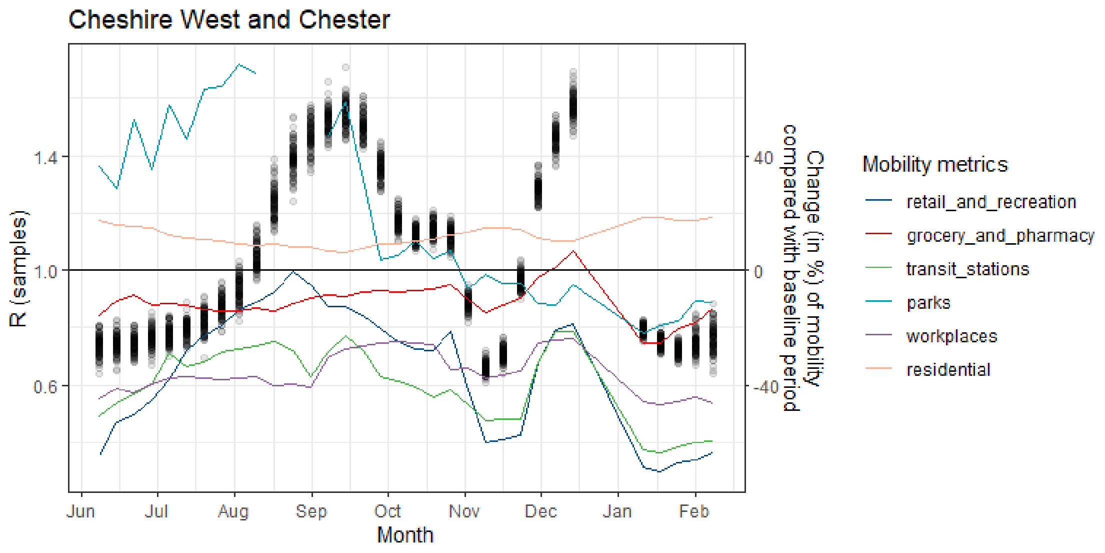
Cheshire West and Chester
R (samples)
1.4
1.0
0.6
40
0
-40
Change (in %) of mobility compared with baseline period
Jun Jul Aug Sep Oct Nov Dec Jan Feb
Month
Mobility metrics
retail_and_recreation
grocery_and_pharmacy
transit_stations
parks
workplaces
residential

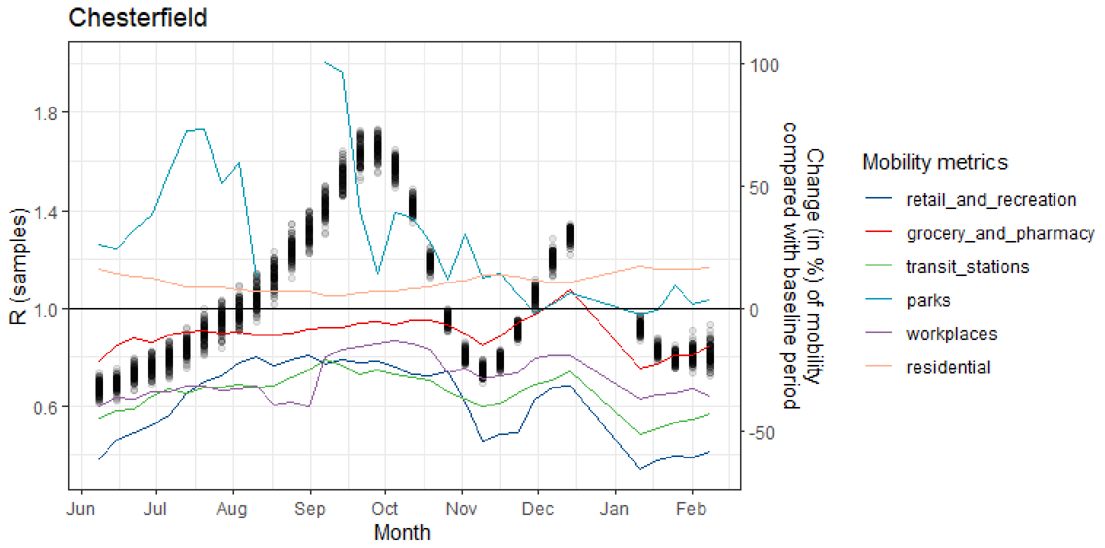
Chesterfield
R (samples)
1.8
1.4
1.0
0.6
100
50
0
-50
Change (in %) of mobility compared with baseline period
Jun Jul Aug Sep Oct Nov Dec Jan Feb
Month
Mobility metrics
retail_and_recreation
grocery_and_pharmacy
transit_stations
parks
workplaces
residential

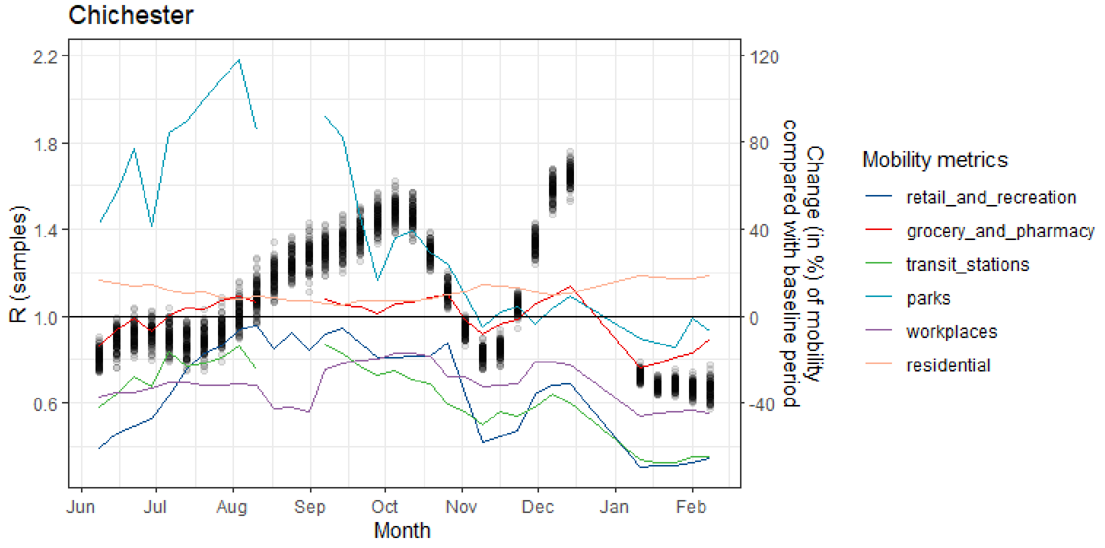
Chichester
R (samples)
2.2
1.8
1.4
1.0
0.6
120
80
40
0
-40
Change (in %) of mobility compared with baseline period
Jun Jul Aug Sep Oct Nov Dec Jan Feb
Month
Mobility metrics
retail_and_recreation
grocery_and_pharmacy
transit_stations
parks
workplaces
residential

Chiltern

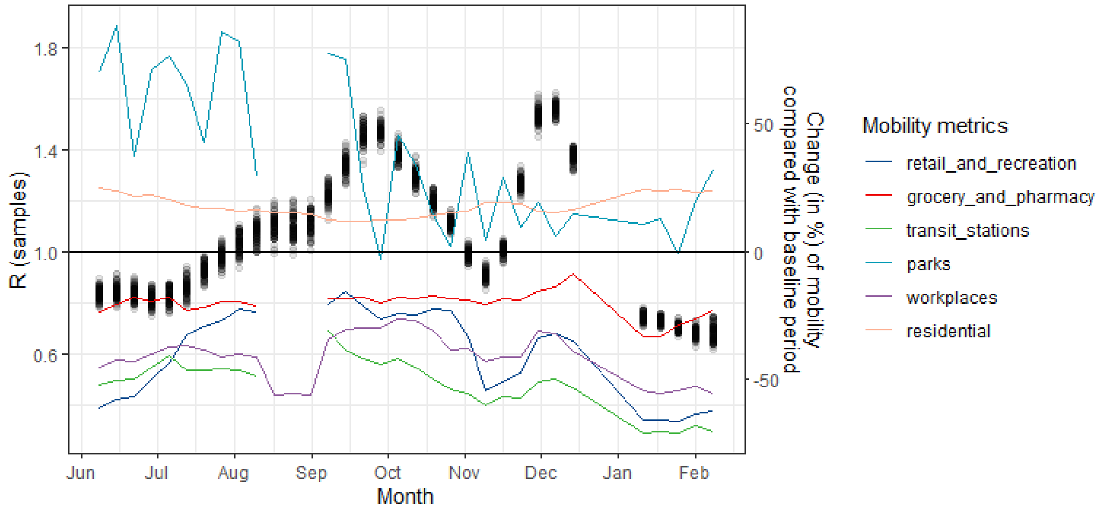

Chorley

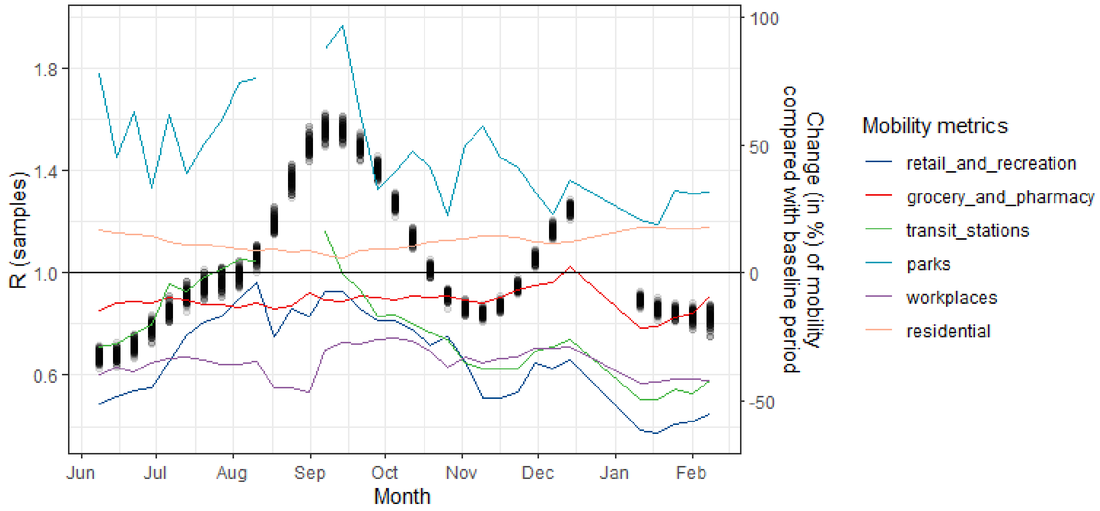

City of Edinburgh

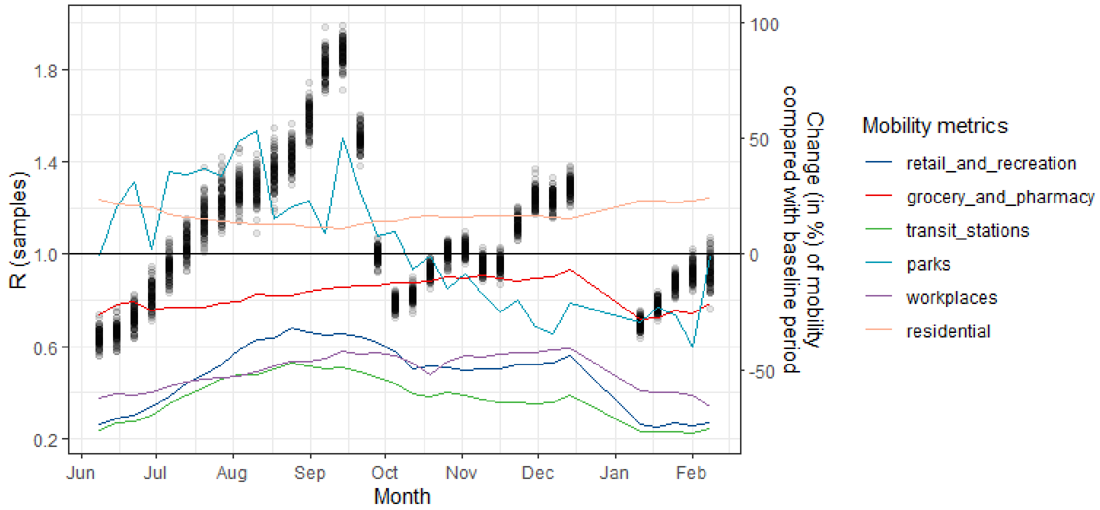

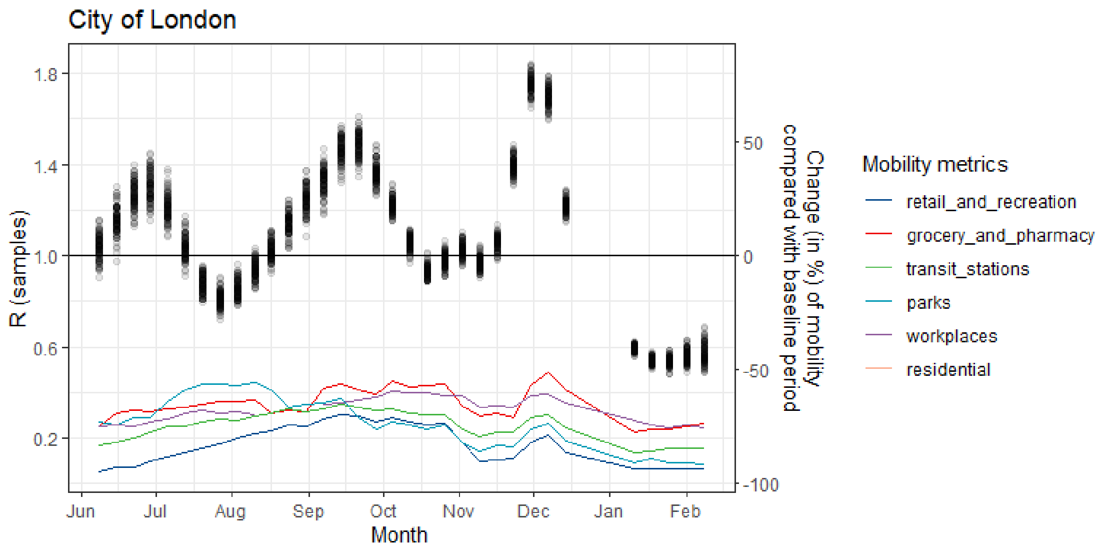
City of London
R (samples)
1.8
1.4
1.0
0.6
0.2
50
0
-50
-100
Change (in %) of mobility compared with baseline period
Jun
Jul
Aug
Sep
Oct
Nov
Dec
Jan
Feb
Month
Mobility metrics
retail_and_recreation
grocery_and_pharmacy
transit_stations
parks
workplaces
residential

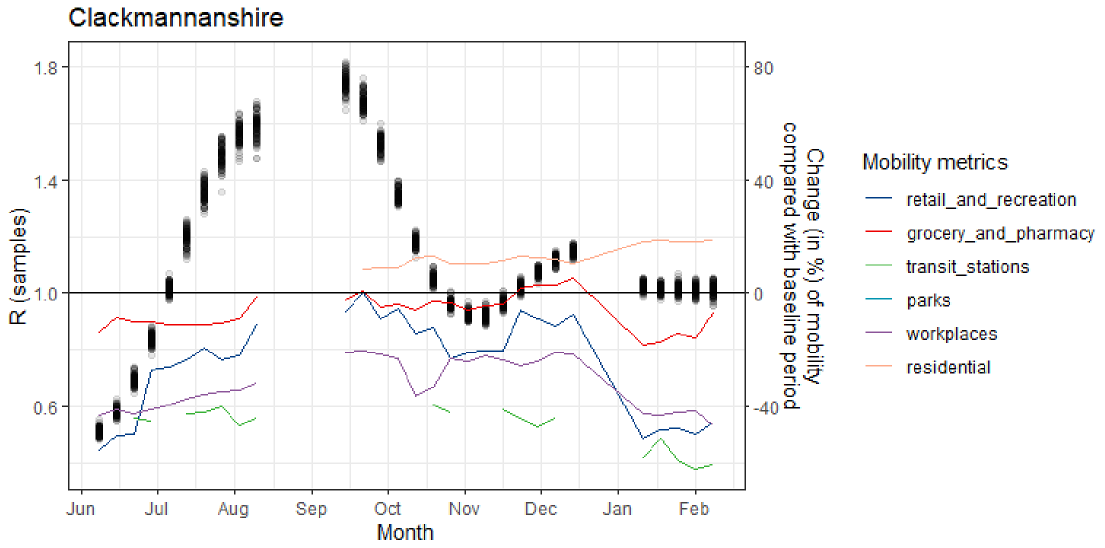
Clackmannanshire
R (samples)
1.8
1.4
1.0
0.6
80
40
0
-40
Change (in %) of mobility compared with baseline period
Jun
Jul
Aug
Sep
Oct
Nov
Dec
Jan
Feb
Month
Mobility metrics
retail_and_recreation
grocery_and_pharmacy
transit_stations
parks
workplaces
residential

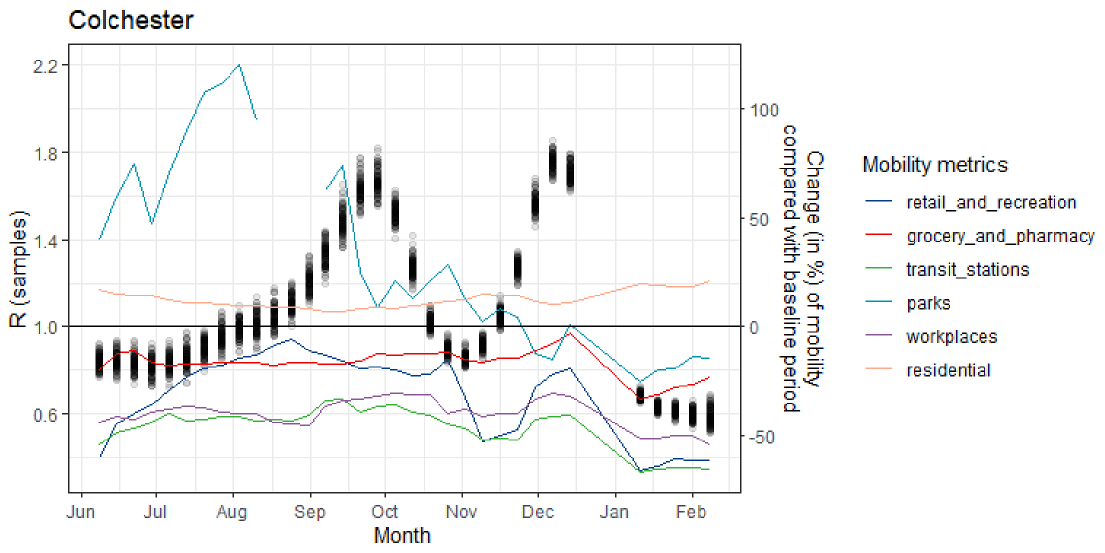
Colchester
R (samples)
2.2
1.8
1.4
1.0
0.6
100
50
0
-50
Change (in %) of mobility compared with baseline period
Jun
Jul
Aug
Sep
Oct
Nov
Dec
Jan
Feb
Month
Mobility metrics
retail_and_recreation
grocery_and_pharmacy
transit_stations
parks
workplaces
residential

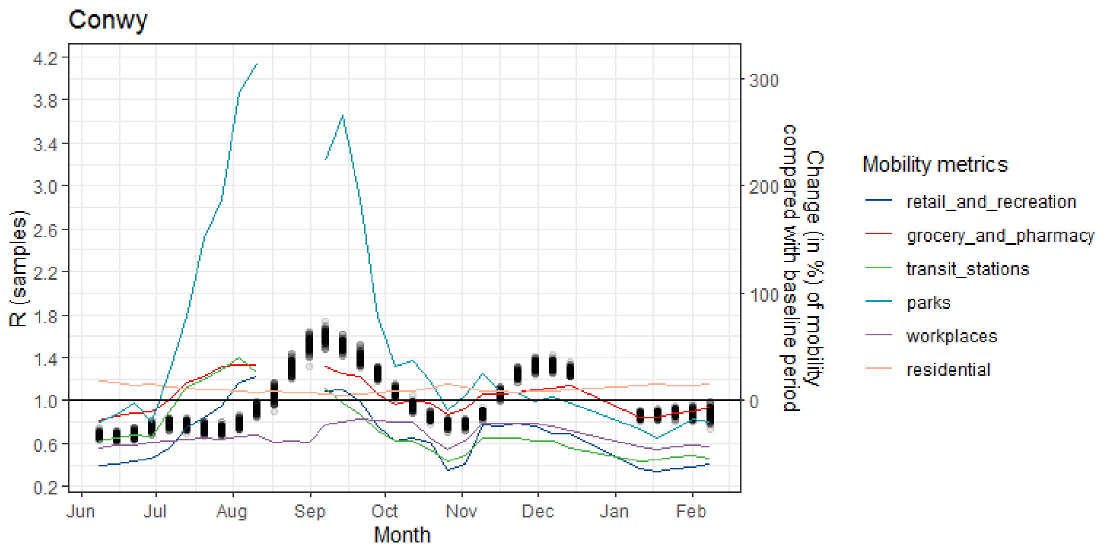
Conwy
R (samples)
4.2
3.8
3.4
3.0
2.6
2.2
1.8
1.4
1.0
0.6
0.2
Jun
Jul
Aug
Sep
Oct
Nov
Dec
Jan
Feb
Month
300
200
100
0
Change (in %) of mobility compared with baseline period
Mobility metrics
retail_and_recreation
grocery_and_pharmacy
transit_stations
parks
workplaces
residential

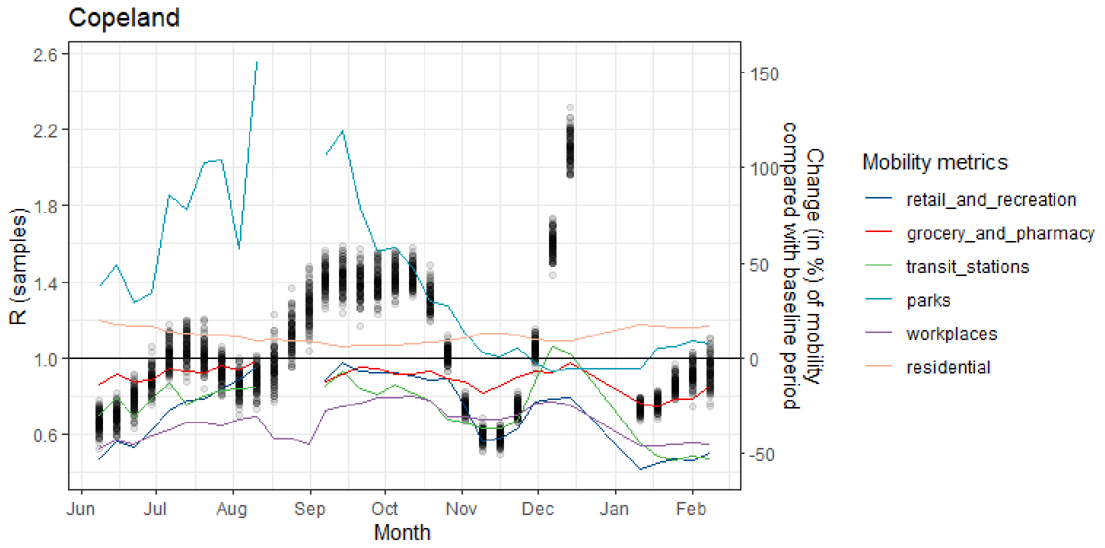
Copeland
R (samples)
2.6
2.2
1.8
1.4
1.0
0.6
Jun
Jul
Aug
Sep
Oct
Nov
Dec
Jan
Feb
Month
150
100
50
0
-50
Change (in %) of mobility compared with baseline period
Mobility metrics
retail_and_recreation
grocery_and_pharmacy
transit_stations
parks
workplaces
residential

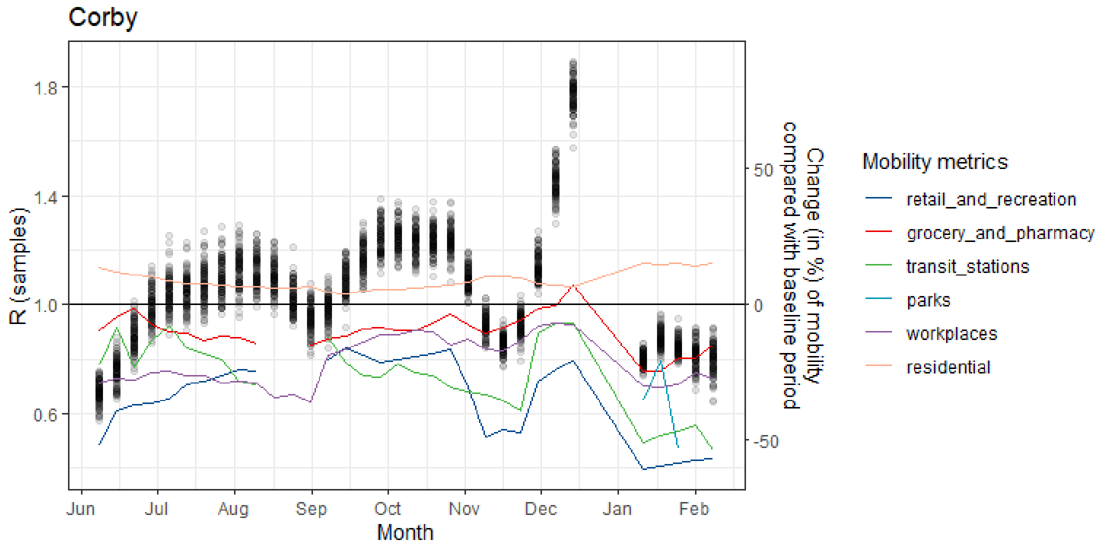
Corby
R (samples)
1.8
1.4
1.0
0.6
Jun
Jul
Aug
Sep
Oct
Nov
Dec
Jan
Feb
Month
50
0
-50
Change (in %) of mobility compared with baseline period
Mobility metrics
retail_and_recreation
grocery_and_pharmacy
transit_stations
parks
workplaces
residential

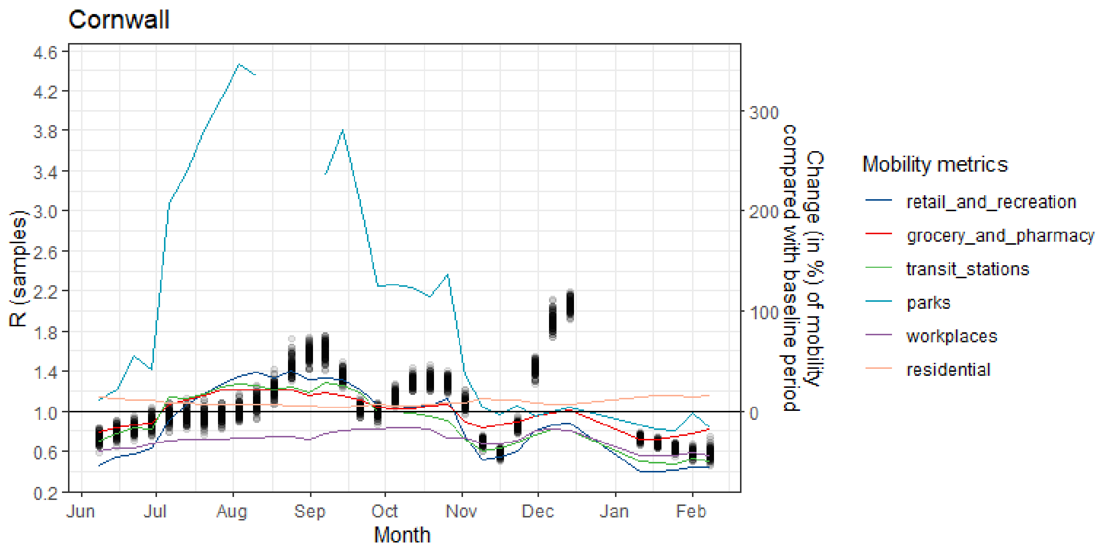
Cornwall
R (samples)
4.6
4.2
3.8
3.4
3.0
2.6
2.2
1.8
1.4
1.0
0.6
0.2
300
200
100
0
Change (in %) of mobility compared with baseline period
Jun Jul Aug Sep Oct Nov Dec Jan Feb
Month
Mobility metrics
retail_and_recreation
grocery_and_pharmacy
transit_stations
parks
workplaces
residential

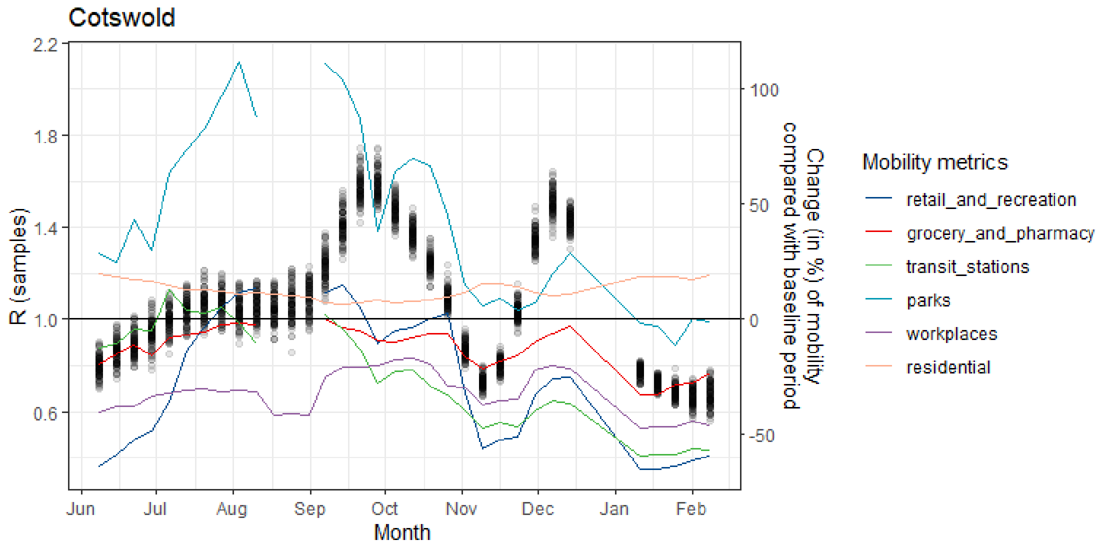
Cotswold
R (samples)
2.2
1.8
1.4
1.0
0.6
100
50
0
-50
Change (in %) of mobility compared with baseline period
Jun Jul Aug Sep Oct Nov Dec Jan Feb
Month
Mobility metrics
retail_and_recreation
grocery_and_pharmacy
transit_stations
parks
workplaces
residential

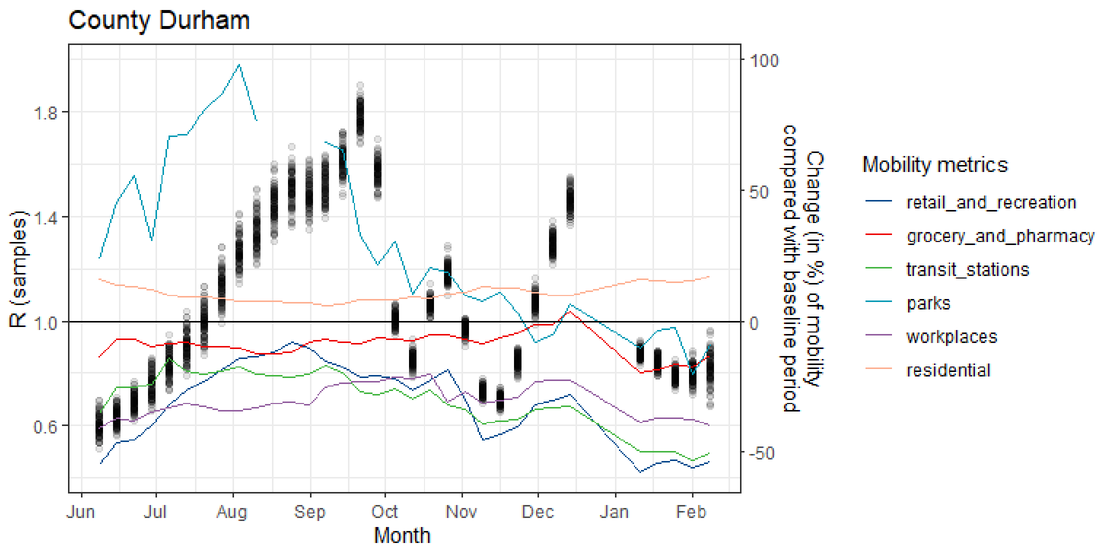
County Durham
R (samples)
1.8
1.4
1.0
0.6
100
50
0
-50
Change (in %) of mobility compared with baseline period
Jun Jul Aug Sep Oct Nov Dec Jan Feb
Month
Mobility metrics
retail_and_recreation
grocery_and_pharmacy
transit_stations
parks
workplaces
residential

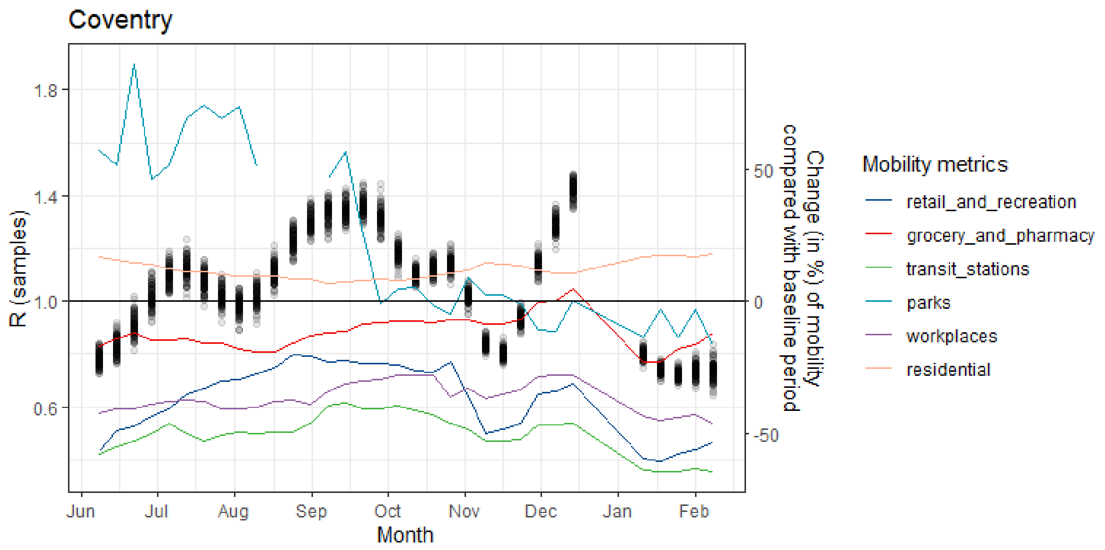
Coventry
R (samples)
1.8
1.4
1.0
0.6
Jun
Jul
Aug
Sep
Oct
Nov
Dec
Jan
Feb
Month
Change (in %) of mobility compared with baseline period
50
0
-50
Mobility metrics
retail_and_recreation
grocery_and_pharmacy
transit_stations
parks
workplaces
residential

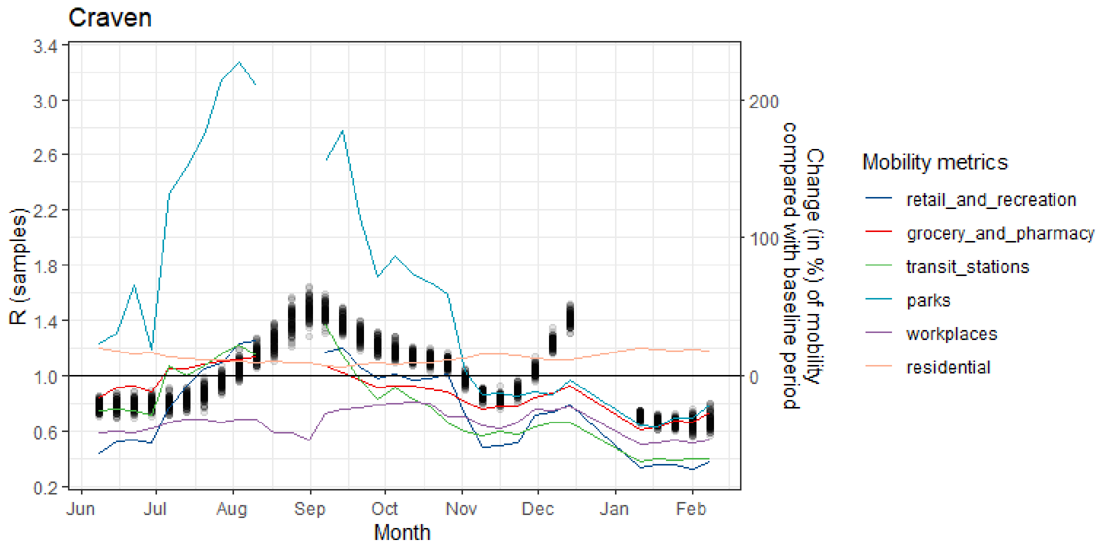
Craven
R (samples)
3.4
3.0
2.6
2.2
1.8
1.4
1.0
0.6
0.2
Jun
Jul
Aug
Sep
Oct
Nov
Dec
Jan
Feb
Month
Change (in %) of mobility compared with baseline period
200
100
0
Mobility metrics
retail_and_recreation
grocery_and_pharmacy
transit_stations
parks
workplaces
residential

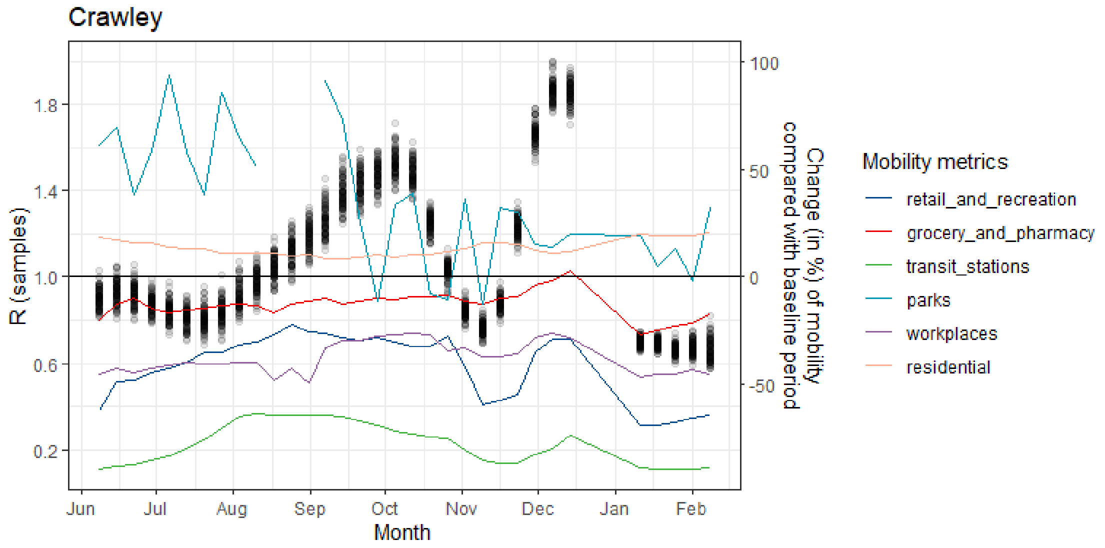
Crawley
R (samples)
1.8
1.4
1.0
0.6
0.2
Jun
Jul
Aug
Sep
Oct
Nov
Dec
Jan
Feb
Month
Change (in %) of mobility compared with baseline period
100
50
0
-50
Mobility metrics
retail_and_recreation
grocery_and_pharmacy
transit_stations
parks
workplaces
residential

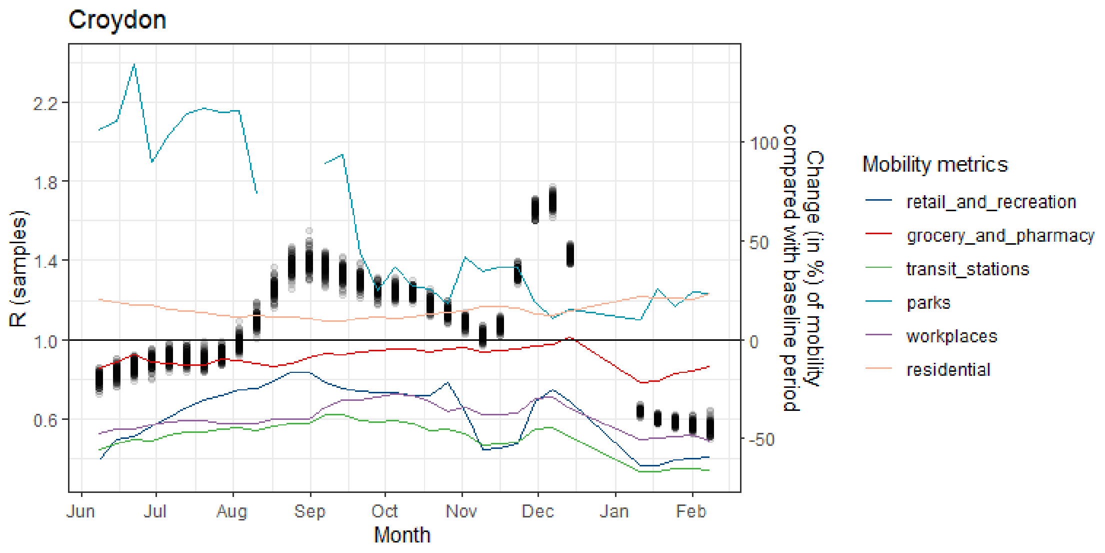
Croydon
R (samples)
2.2
1.8
1.4
1.0
0.6
Jun
Jul
Aug
Sep
Oct
Nov
Dec
Jan
Feb
Month
100
50
0
-50
Change (in %) of mobility compared with baseline period
Mobility metrics
retail_and_recreation
grocery_and_pharmacy
transit_stations
parks
workplaces
residential

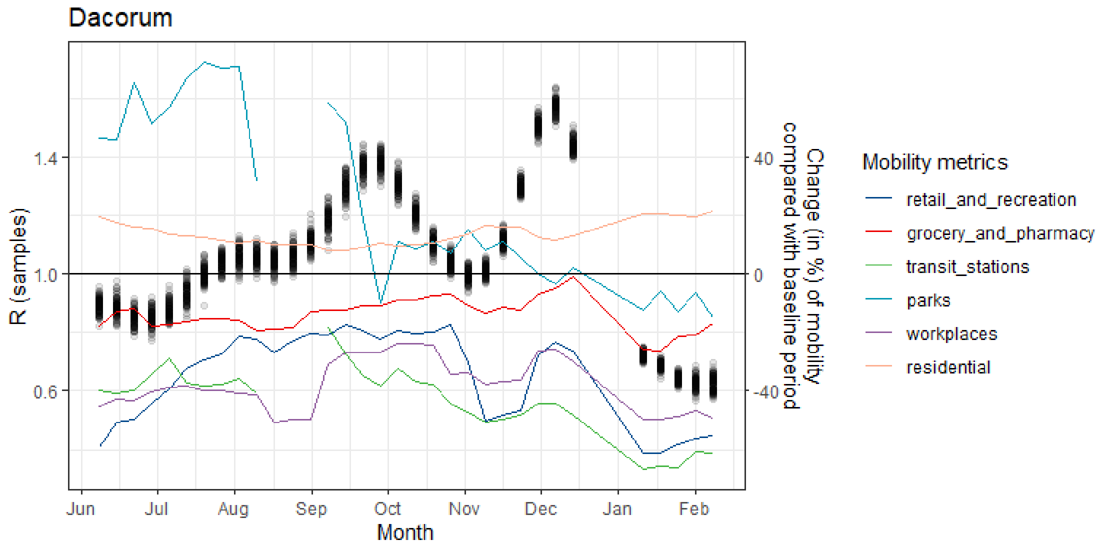
Dacorum
R (samples)
1.4
1.0
0.6
Jun
Jul
Aug
Sep
Oct
Nov
Dec
Jan
Feb
Month
40
0
-40
Change (in %) of mobility compared with baseline period
Mobility metrics
retail_and_recreation
grocery_and_pharmacy
transit_stations
parks
workplaces
residential

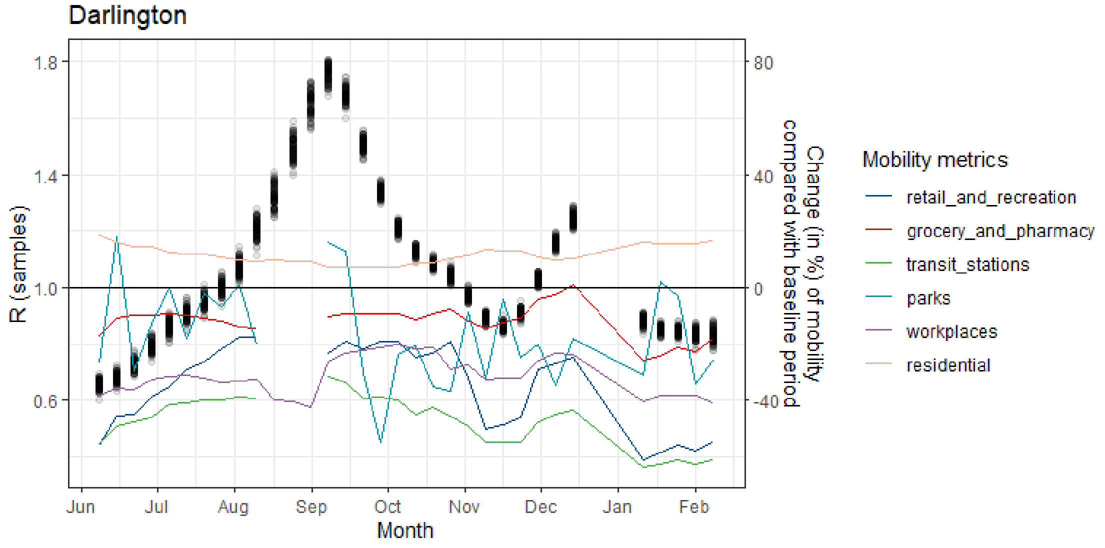
Darlington
R (samples)
1.8
1.4
1.0
0.6
Jun
Jul
Aug
Sep
Oct
Nov
Dec
Jan
Feb
Month
80
40
0
-40
Change (in %) of mobility compared with baseline period
Mobility metrics
retail_and_recreation
grocery_and_pharmacy
transit_stations
parks
workplaces
residential

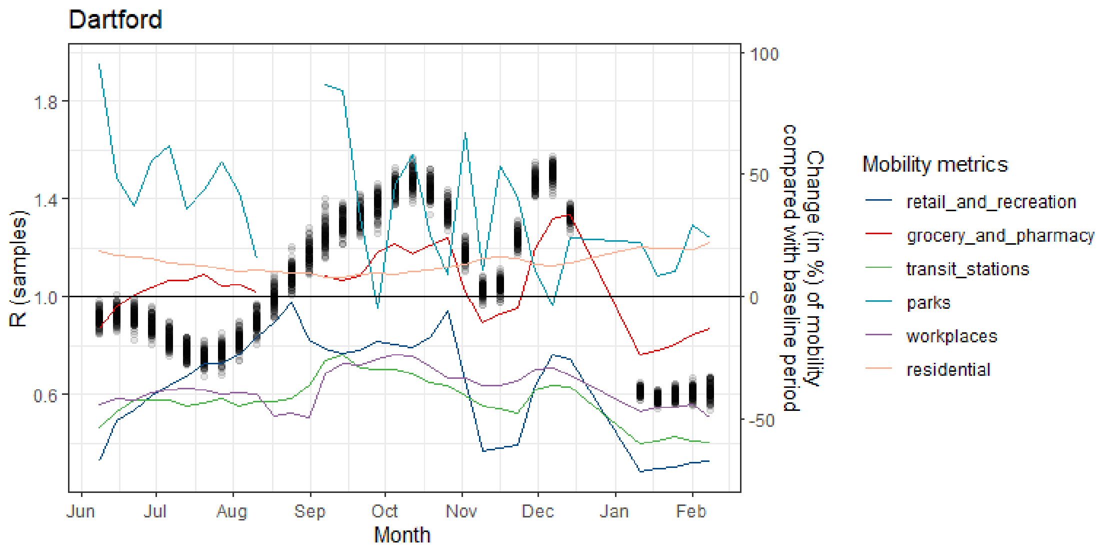
Dartford
R (samples)
1.8
1.4
1.0
0.6
Jun
Jul
Aug
Sep
Oct
Nov
Dec
Jan
Feb
Month
100
50
0
-50
Change (in %) of mobility compared with baseline period
Mobility metrics
retail_and_recreation
grocery_and_pharmacy
transit_stations
parks
workplaces
residential

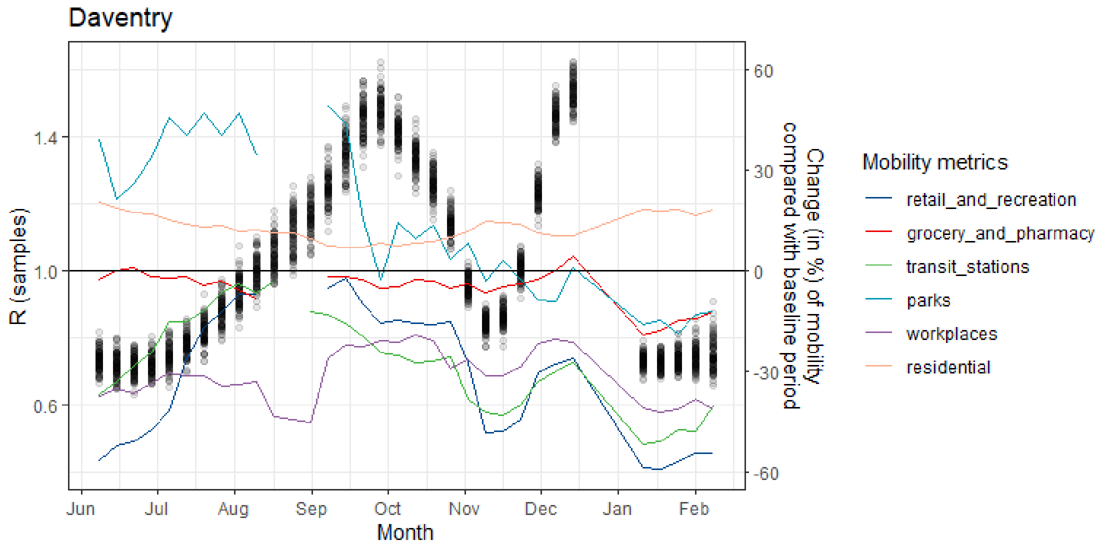
Daventry
R (samples)
1.4
1.0
0.6
Jun
Jul
Aug
Sep
Oct
Nov
Dec
Jan
Feb
Month
60
30
0
-30
-60
Change (in %) of mobility compared with baseline period
Mobility metrics
retail_and_recreation
grocery_and_pharmacy
transit_stations
parks
workplaces
residential

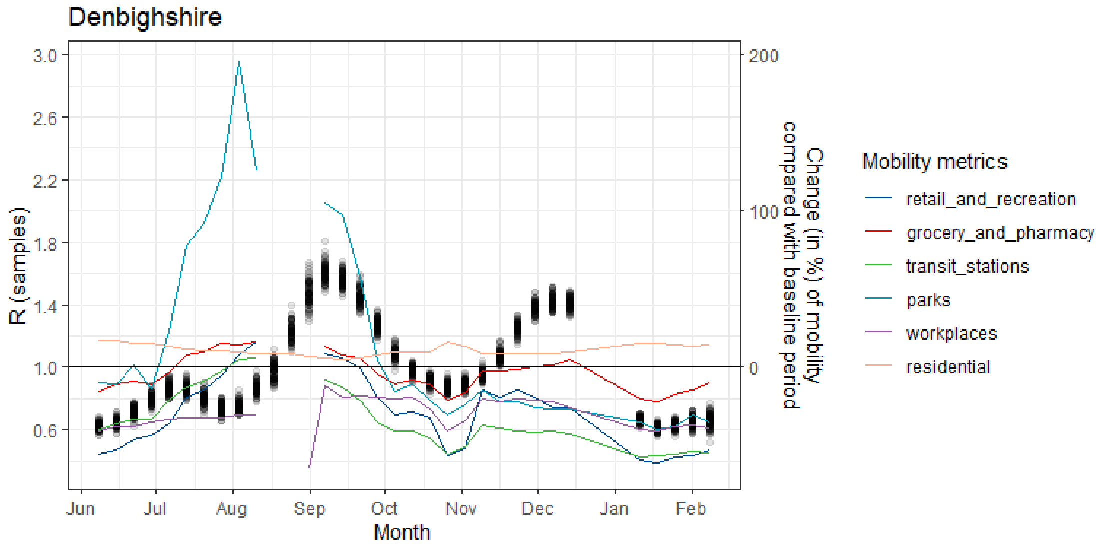
Denbighshire
R (samples)
3.0
2.6
2.2
1.8
1.4
1.0
0.6
Jun
Jul
Aug
Sep
Oct
Nov
Dec
Jan
Feb
Month
200
100
0
Change (in %) of mobility compared with baseline period
Mobility metrics
retail_and_recreation
grocery_and_pharmacy
transit_stations
parks
workplaces
residential

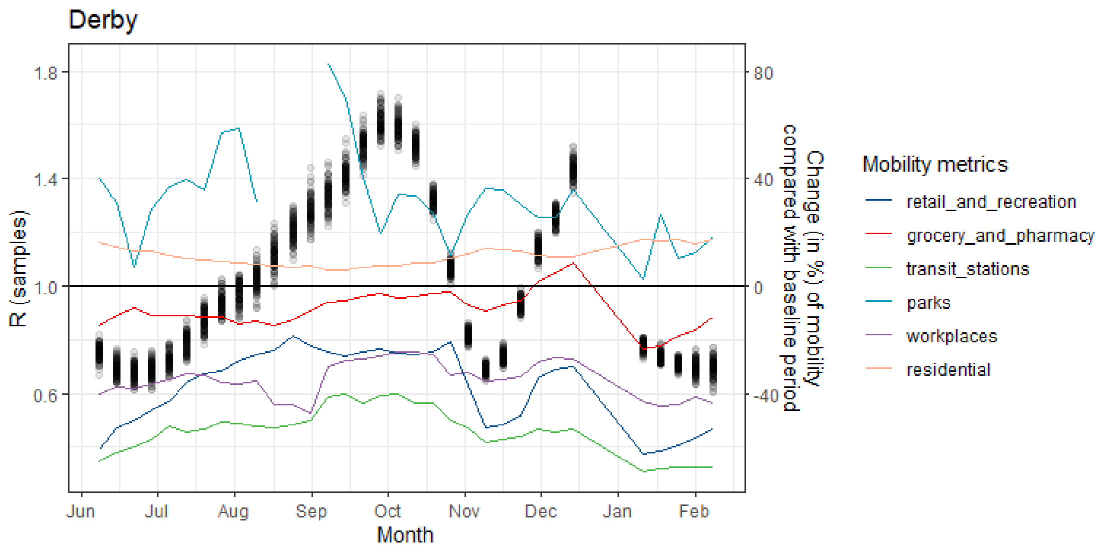
Derby
R (samples)
1.8
1.4
1.0
0.6
Jun
Jul
Aug
Sep
Oct
Nov
Dec
Jan
Feb
Month
80
40
0
-40
Change (in %) of mobility compared with baseline period
Mobility metrics
retail_and_recreation
grocery_and_pharmacy
transit_stations
parks
workplaces
residential

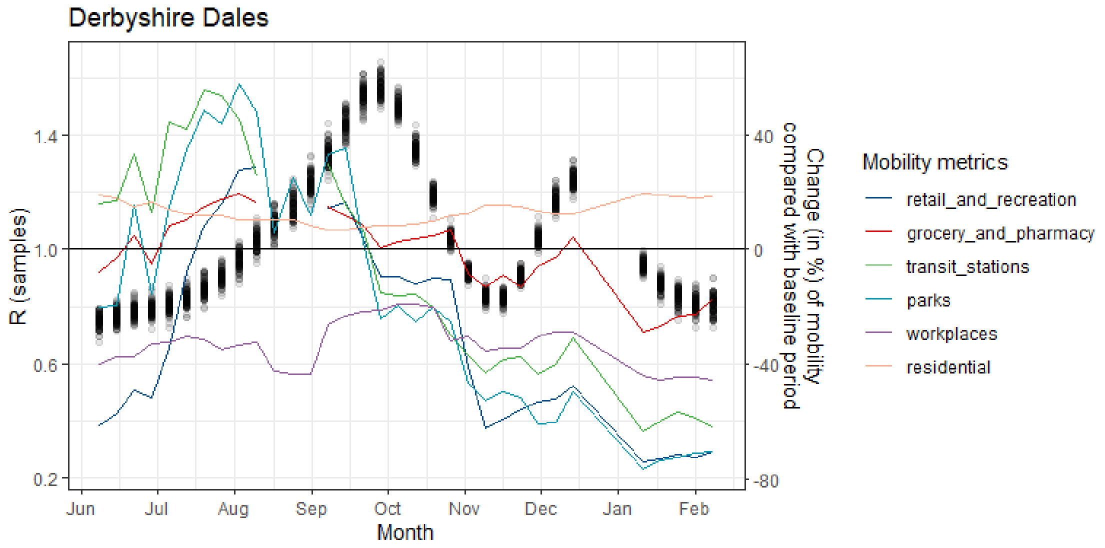
Derbyshire Dales
R (samples)
1.4
1.0
0.6
0.2
Jun
Jul
Aug
Sep
Oct
Nov
Dec
Jan
Feb
Month
40
0
-40
-80
Change (in %) of mobility compared with baseline period
Mobility metrics
retail_and_recreation
grocery_and_pharmacy
transit_stations
parks
workplaces
residential

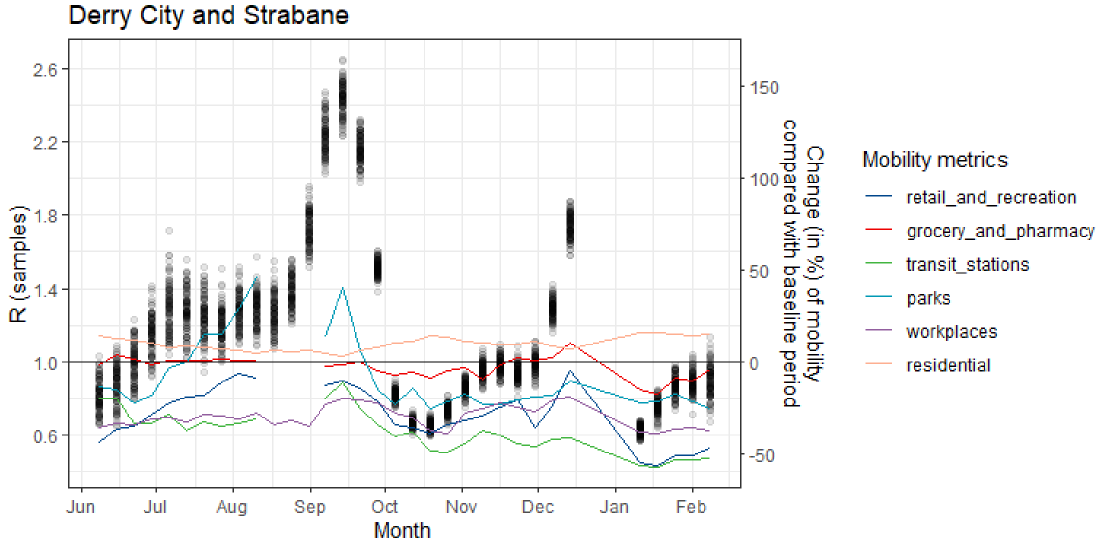
Derry City and Strabane
R (samples)
2.6
2.2
1.8
1.4
1.0
0.6
Jun
Jul
Aug
Sep
Oct
Nov
Dec
Jan
Feb
Month
150
100
50
0
-50
Change (in %) of mobility compared with baseline period
Mobility metrics
retail_and_recreation
grocery_and_pharmacy
transit_stations
parks
workplaces
residential

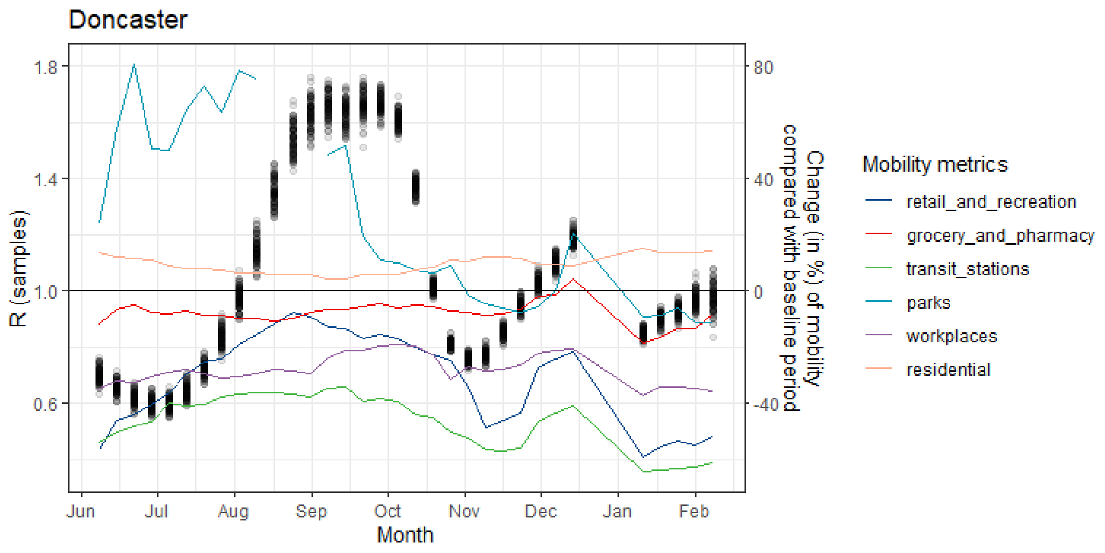
Doncaster
R (samples)
1.8
1.4
1.0
0.6
80
40
0
-40
Change (in %) of mobility compared with baseline period
Jun Jul Aug Sep Oct Nov Dec Jan Feb
Month
Mobility metrics
retail_and_recreation
grocery_and_pharmacy
transit_stations
parks
workplaces
residential

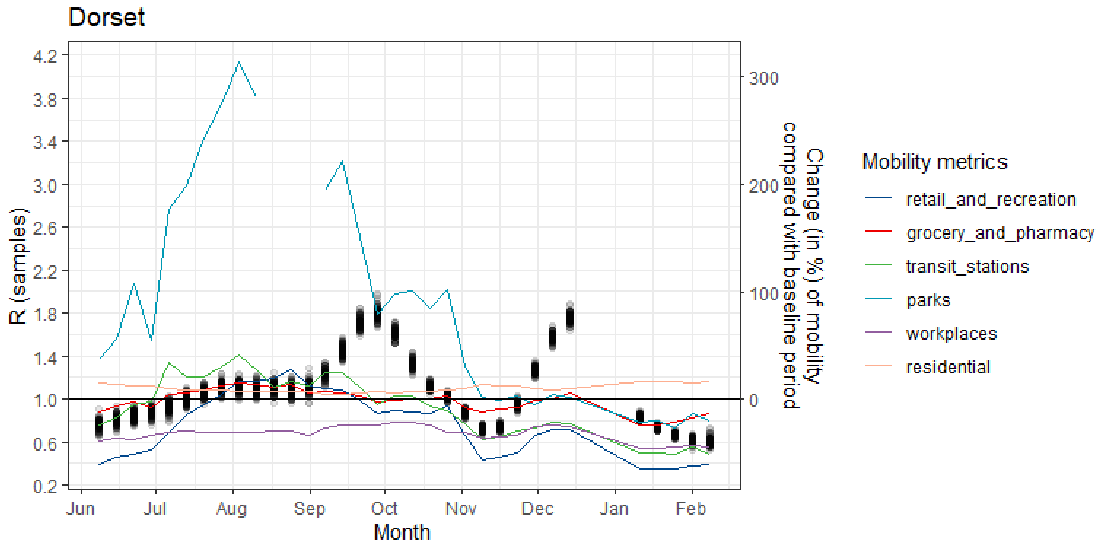
Dorset
R (samples)
4.2
3.8
3.4
3.0
2.6
2.2
1.8
1.4
1.0
0.6
0.2
300
200
100
0
Change (in %) of mobility compared with baseline period
Jun Jul Aug Sep Oct Nov Dec Jan Feb
Month
Mobility metrics
retail_and_recreation
grocery_and_pharmacy
transit_stations
parks
workplaces
residential

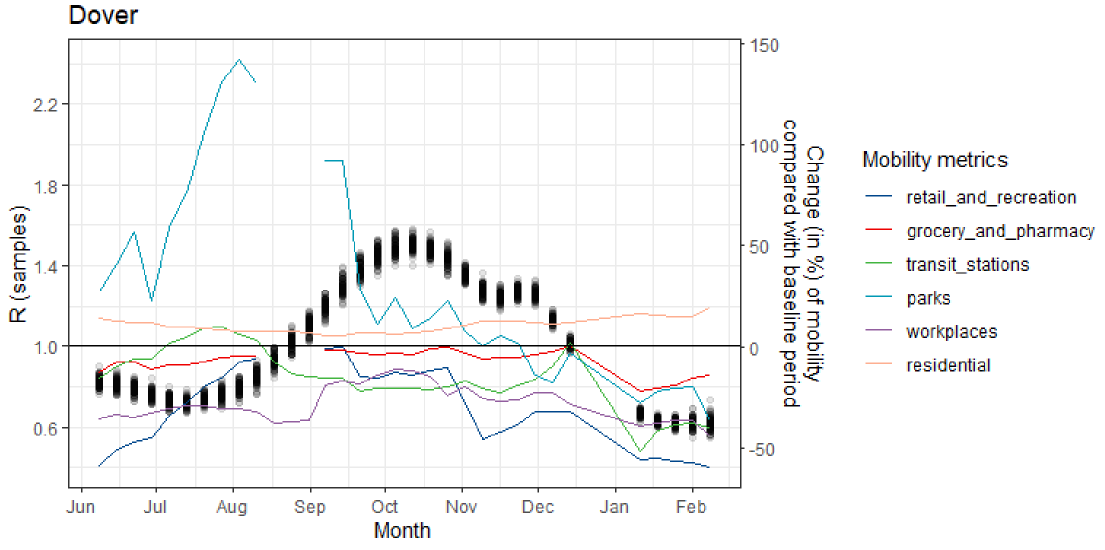
Dover
R (samples)
2.2
1.8
1.4
1.0
0.6
150
100
50
0
-50
Change (in %) of mobility compared with baseline period
Jun Jul Aug Sep Oct Nov Dec Jan Feb
Month
Mobility metrics
retail_and_recreation
grocery_and_pharmacy
transit_stations
parks
workplaces
residential

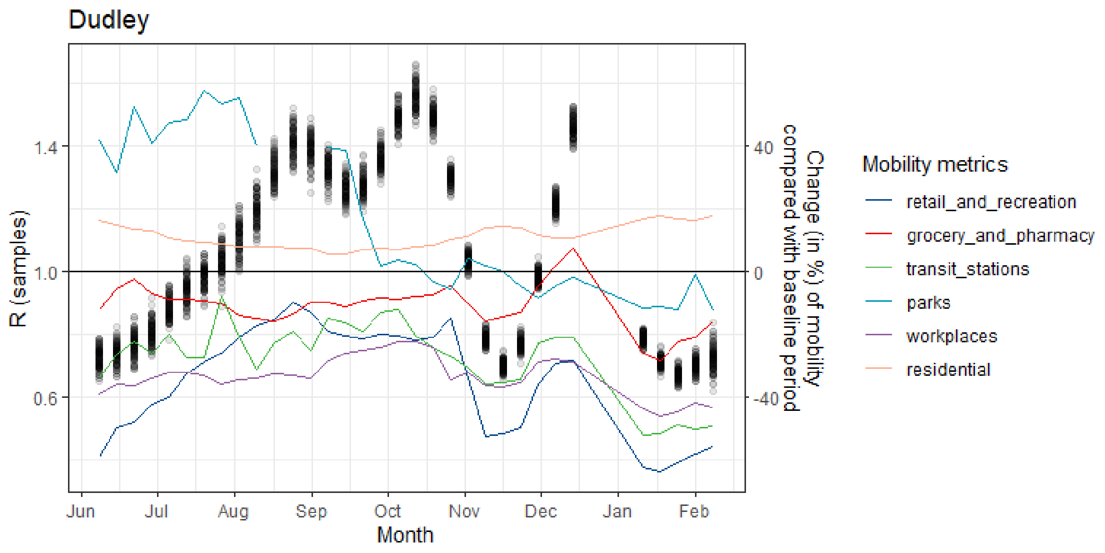
Dudley
R (samples)
1.4
1.0
0.6
Jun
Jul
Aug
Sep
Oct
Nov
Dec
Jan
Feb
Month
40
0
-40
Change (in %) of mobility compared with baseline period
Mobility metrics
retail_and_recreation
grocery_and_pharmacy
transit_stations
parks
workplaces
residential

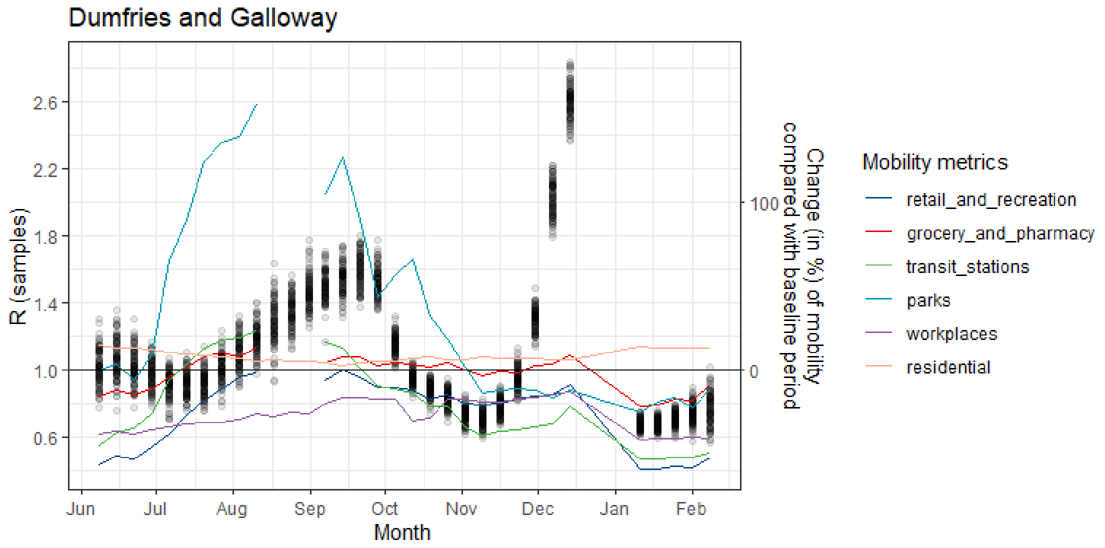
Dumfries and Galloway
R (samples)
2.6
2.2
1.8
1.4
1.0
0.6
Jun
Jul
Aug
Sep
Oct
Nov
Dec
Jan
Feb
Month
100
0
Change (in %) of mobility compared with baseline period
Mobility metrics
retail_and_recreation
grocery_and_pharmacy
transit_stations
parks
workplaces
residential

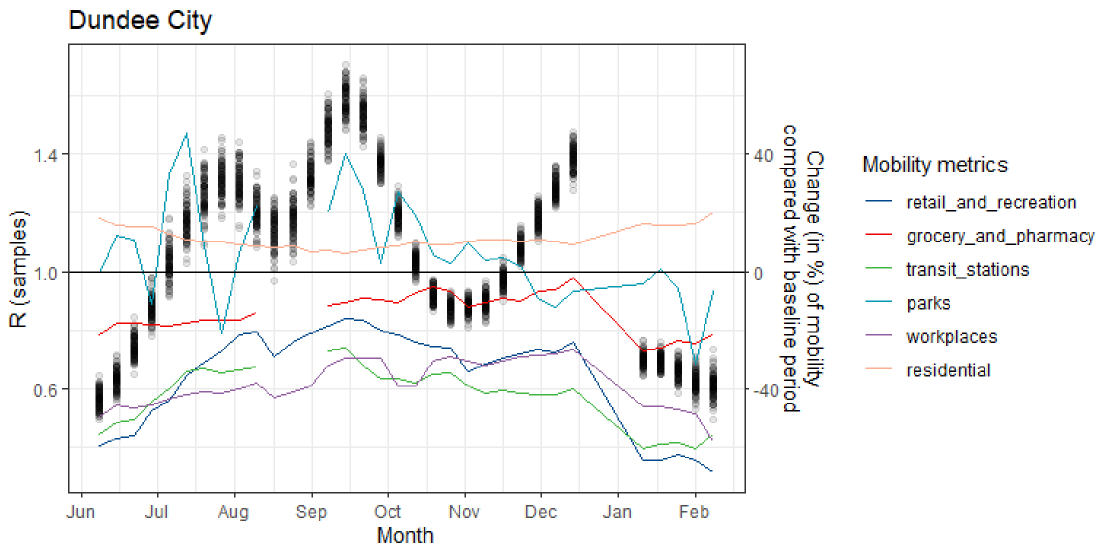
Dundee City
R (samples)
1.4
1.0
0.6
Jun
Jul
Aug
Sep
Oct
Nov
Dec
Jan
Feb
Month
40
0
-40
Change (in %) of mobility compared with baseline period
Mobility metrics
retail_and_recreation
grocery_and_pharmacy
transit_stations
parks
workplaces
residential

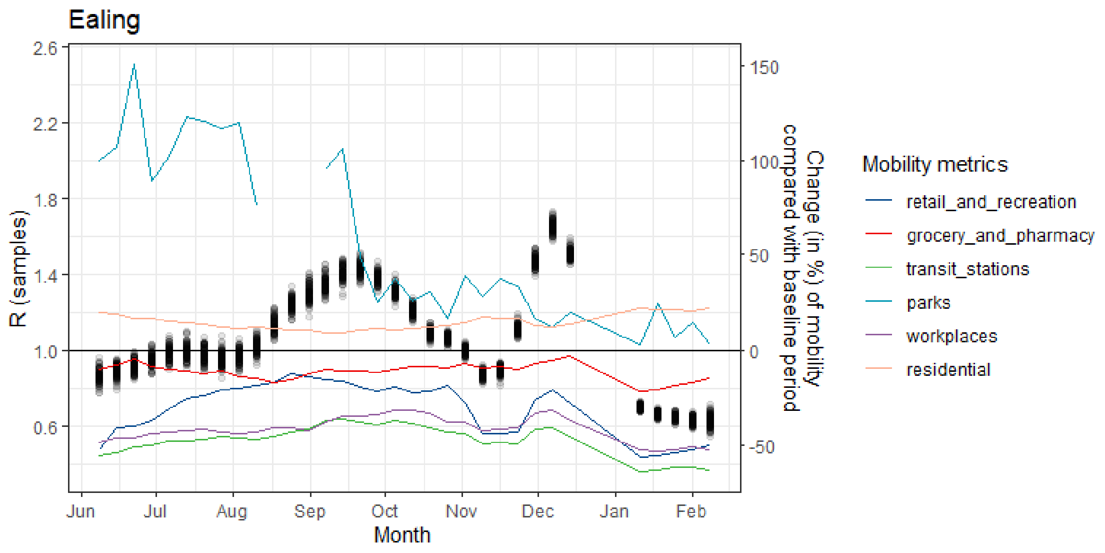
Ealing
R (samples)
Change (in %) of mobility compared with baseline period
Month
Jun
Jul
Aug
Sep
Oct
Nov
Dec
Jan
Feb
2.6
2.2
1.8
1.4
1.0
0.6
150
100
50
0
-50
Mobility metrics
retail_and_recreation
grocery_and_pharmacy
transit_stations
parks
workplaces
residential

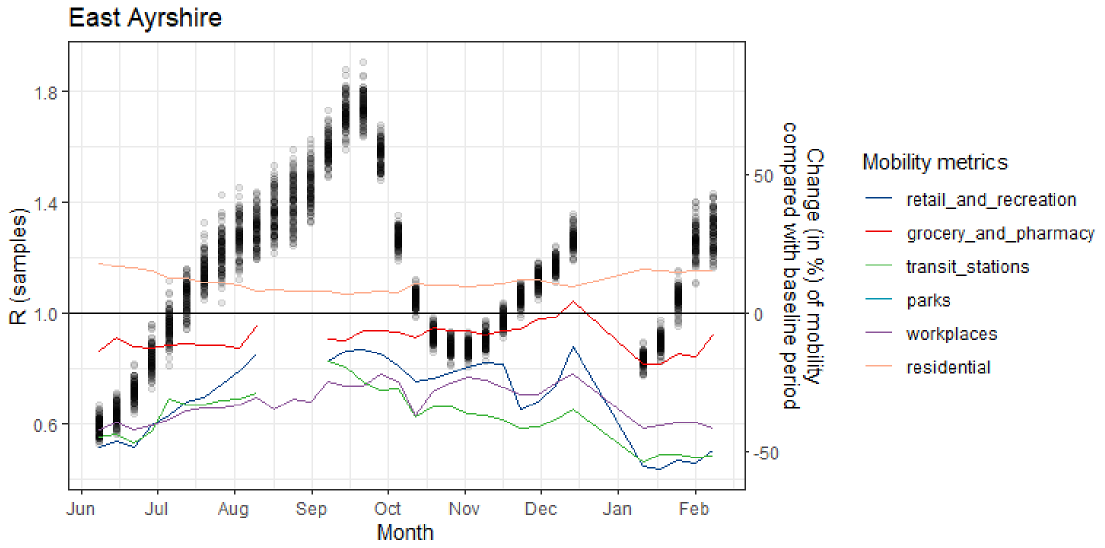
East Ayrshire
R (samples)
Change (in %) of mobility compared with baseline period
Month
Jun
Jul
Aug
Sep
Oct
Nov
Dec
Jan
Feb
1.8
1.4
1.0
0.6
50
0
-50
Mobility metrics
retail_and_recreation
grocery_and_pharmacy
transit_stations
parks
workplaces
residential

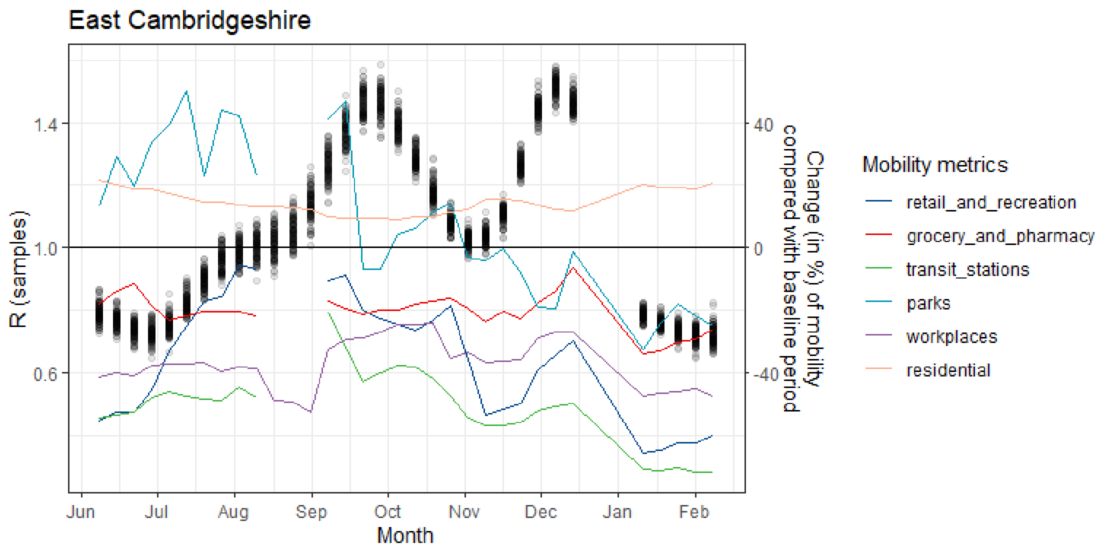
East Cambridgeshire
R (samples)
Change (in %) of mobility compared with baseline period
Month
Jun
Jul
Aug
Sep
Oct
Nov
Dec
Jan
Feb
1.4
1.0
0.6
40
0
-40
Mobility metrics
retail_and_recreation
grocery_and_pharmacy
transit_stations
parks
workplaces
residential

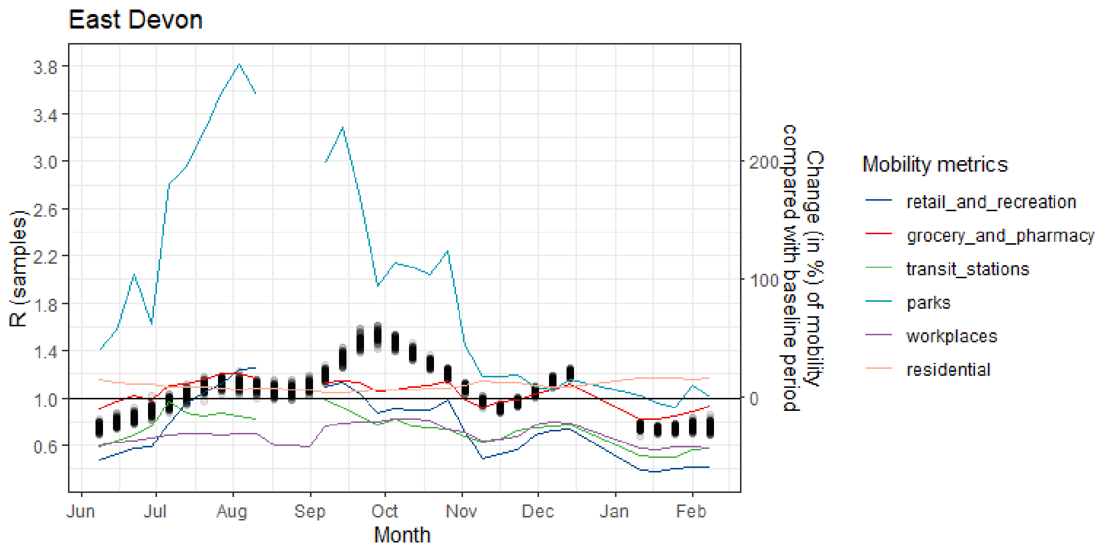
East Devon
R (samples)
3.8
3.4
3.0
2.6
2.2
1.8
1.4
1.0
0.6
200
100
0
Change (in %) of mobility compared with baseline period
Jun Jul Aug Sep Oct Nov Dec Jan Feb
Month
Mobility metrics
retail_and_recreation
grocery_and_pharmacy
transit_stations
parks
workplaces
residential

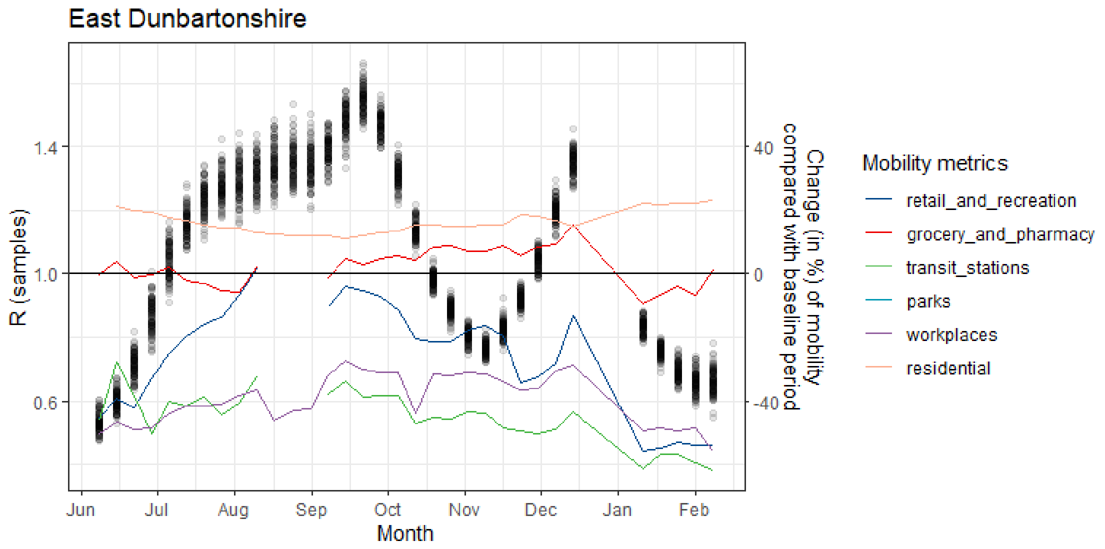
East Dunbartonshire
R (samples)
1.4
1.0
0.6
40
0
-40
Change (in %) of mobility compared with baseline period
Jun Jul Aug Sep Oct Nov Dec Jan Feb
Month
Mobility metrics
retail_and_recreation
grocery_and_pharmacy
transit_stations
parks
workplaces
residential

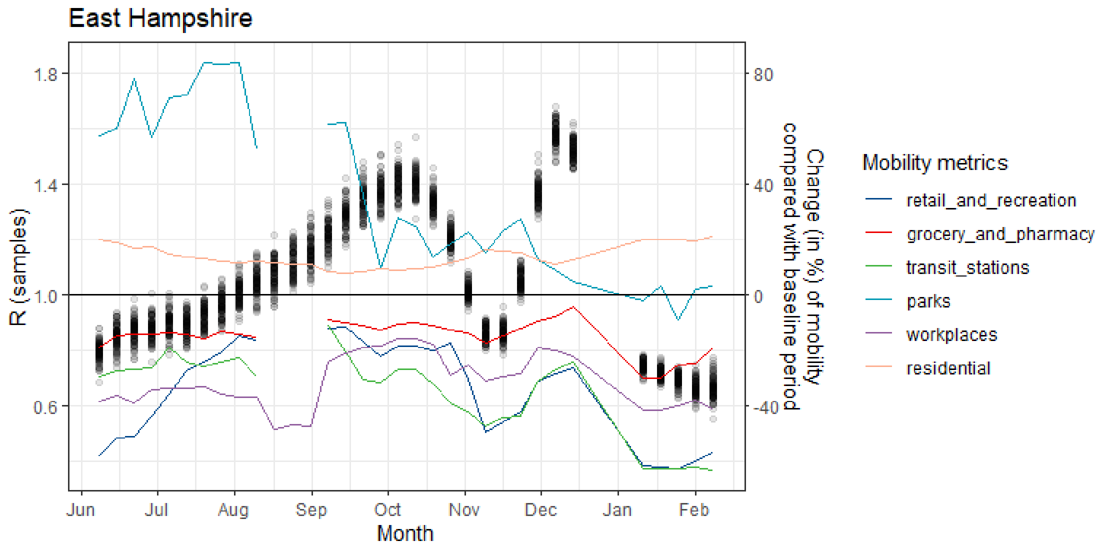
East Hampshire
R (samples)
1.8
1.4
1.0
0.6
80
40
0
-40
Change (in %) of mobility compared with baseline period
Jun Jul Aug Sep Oct Nov Dec Jan Feb
Month
Mobility metrics
retail_and_recreation
grocery_and_pharmacy
transit_stations
parks
workplaces
residential

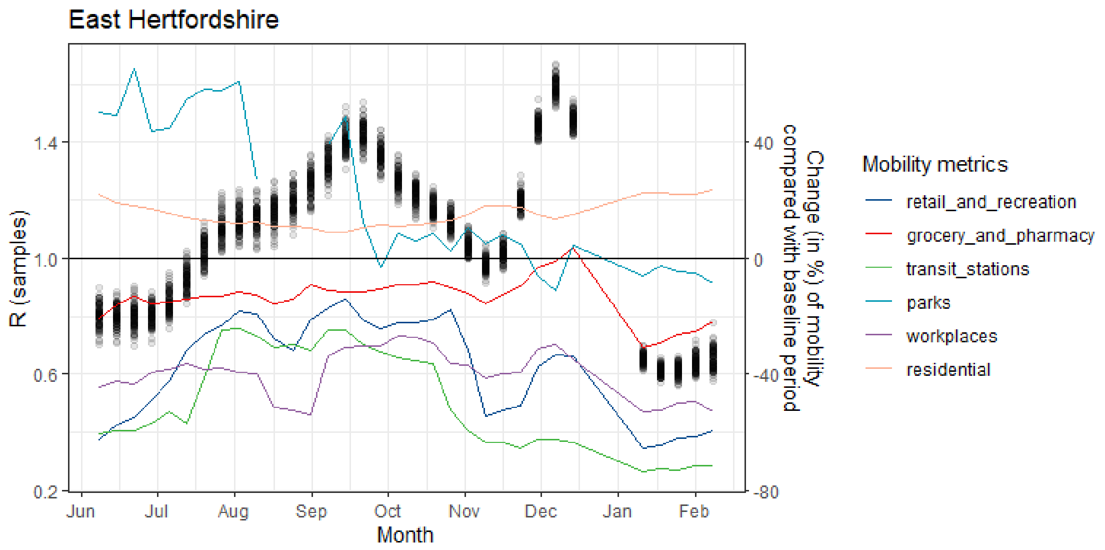
East Hertfordshire
R (samples)
Change (in %) of mobility compared with baseline period
Month
Jun
Jul
Aug
Sep
Oct
Nov
Dec
Jan
Feb
0.2
0.6
1.0
1.4
-80
-40
0
40
Mobility metrics
retail_and_recreation
grocery_and_pharmacy
transit_stations
parks
workplaces
residential

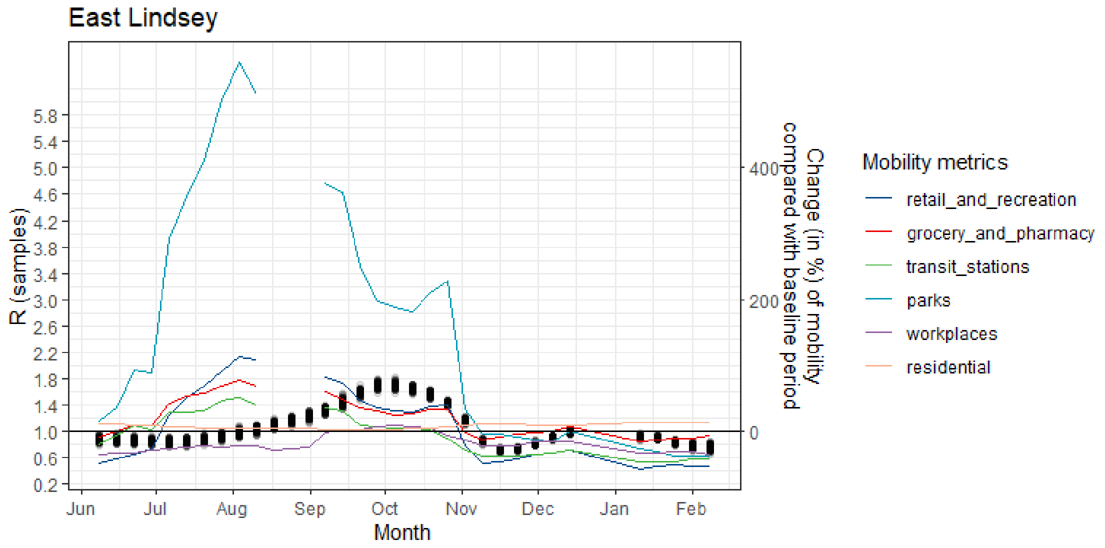
East Lindsey
R (samples)
Change (in %) of mobility compared with baseline period
Month
Jun
Jul
Aug
Sep
Oct
Nov
Dec
Jan
Feb
0.2
0.6
1.0
1.4
1.8
2.2
2.6
3.0
3.4
3.8
4.2
4.6
5.0
5.4
5.8
0
200
400
Mobility metrics
retail_and_recreation
grocery_and_pharmacy
transit_stations
parks
workplaces
residential

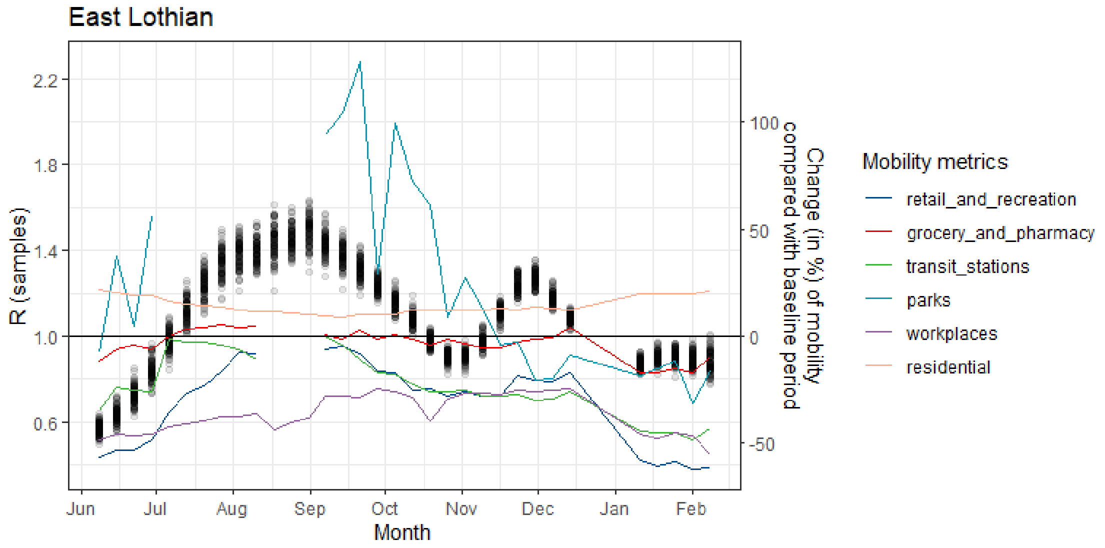
East Lothian
R (samples)
Change (in %) of mobility compared with baseline period
Month
Jun
Jul
Aug
Sep
Oct
Nov
Dec
Jan
Feb
0.6
1.0
1.4
1.8
2.2
-50
0
50
100
Mobility metrics
retail_and_recreation
grocery_and_pharmacy
transit_stations
parks
workplaces
residential

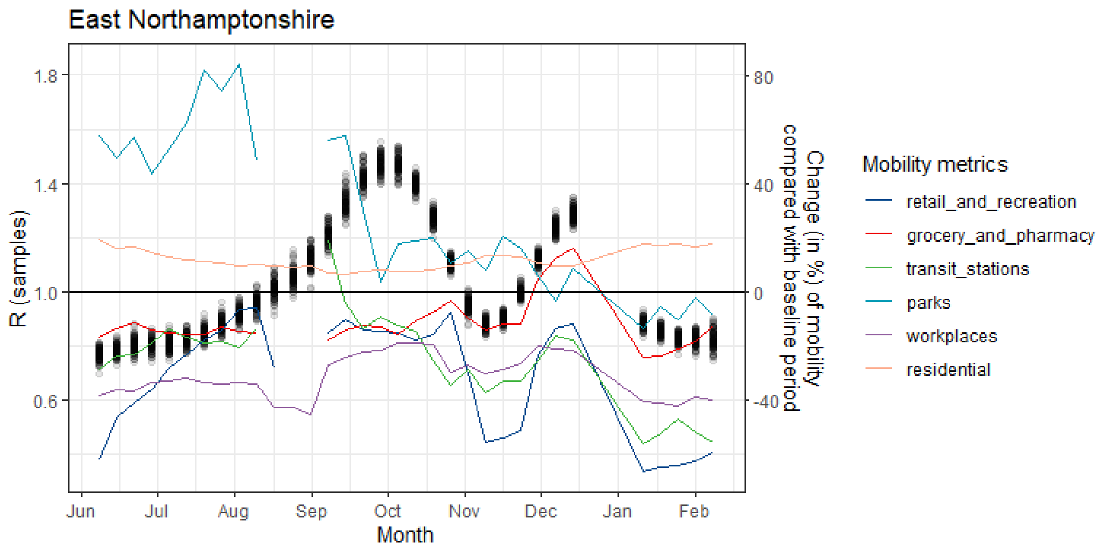
East Northamptonshire
R (samples)
1.8
1.4
1.0
0.6
80
40
0
-40
Change (in %) of mobility compared with baseline period
Jun
Jul
Aug
Sep
Oct
Nov
Dec
Jan
Feb
Month
Mobility metrics
retail_and_recreation
grocery_and_pharmacy
transit_stations
parks
workplaces
residential

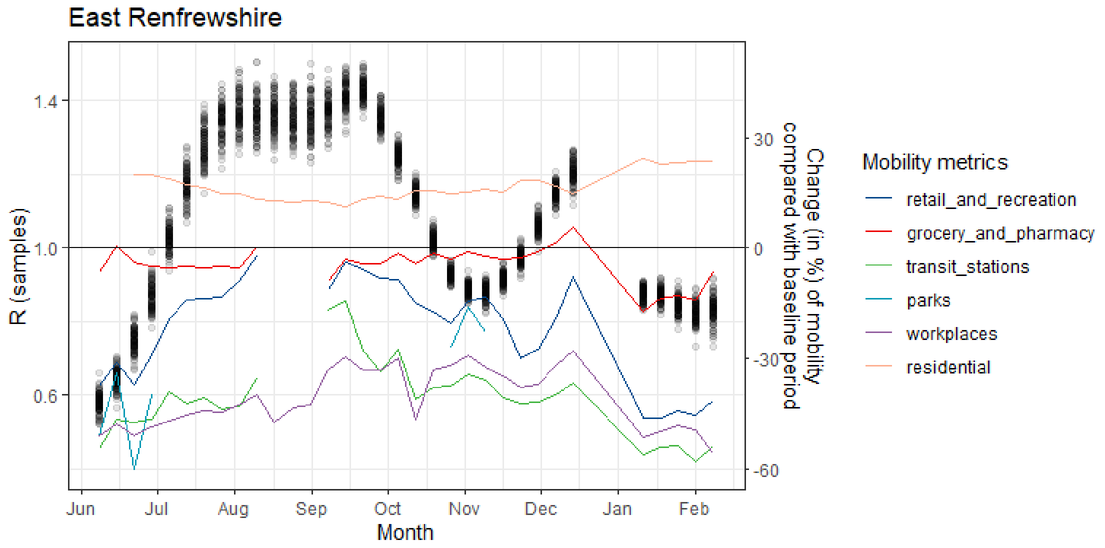
East Renfrewshire
R (samples)
1.4
1.0
0.6
30
0
-30
-60
Change (in %) of mobility compared with baseline period
Jun
Jul
Aug
Sep
Oct
Nov
Dec
Jan
Feb
Month
Mobility metrics
retail_and_recreation
grocery_and_pharmacy
transit_stations
parks
workplaces
residential

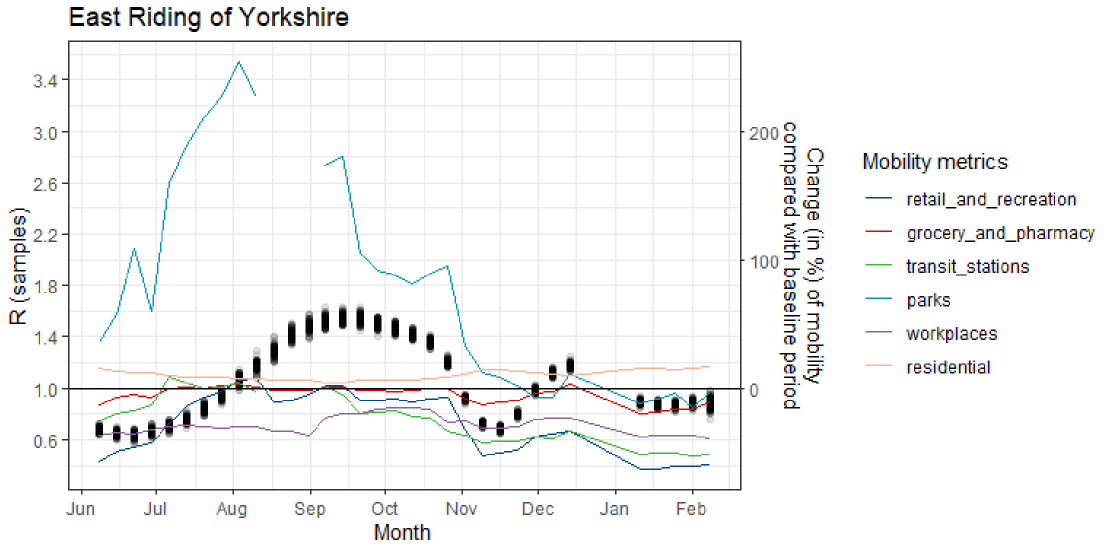
East Riding of Yorkshire
R (samples)
3.4
3.0
2.6
2.2
1.8
1.4
1.0
0.6
200
100
0
Change (in %) of mobility compared with baseline period
Jun
Jul
Aug
Sep
Oct
Nov
Dec
Jan
Feb
Month
Mobility metrics
retail_and_recreation
grocery_and_pharmacy
transit_stations
parks
workplaces
residential

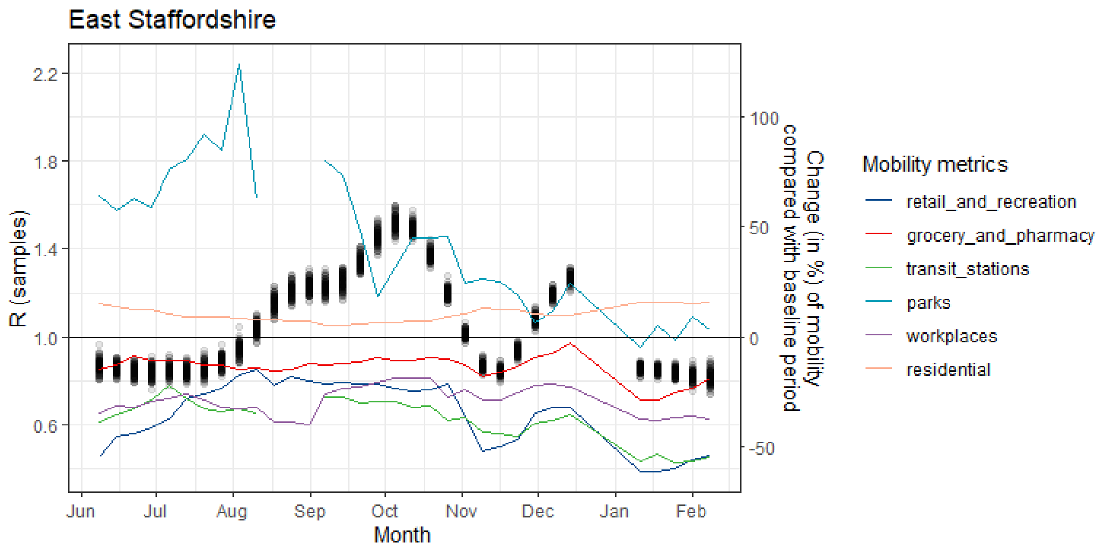
East Staffordshire
R (samples)
Change (in %) of mobility compared with baseline period
Month
Jun
Jul
Aug
Sep
Oct
Nov
Dec
Jan
Feb
Mobility metrics
retail_and_recreation
grocery_and_pharmacy
transit_stations
parks
workplaces
residential

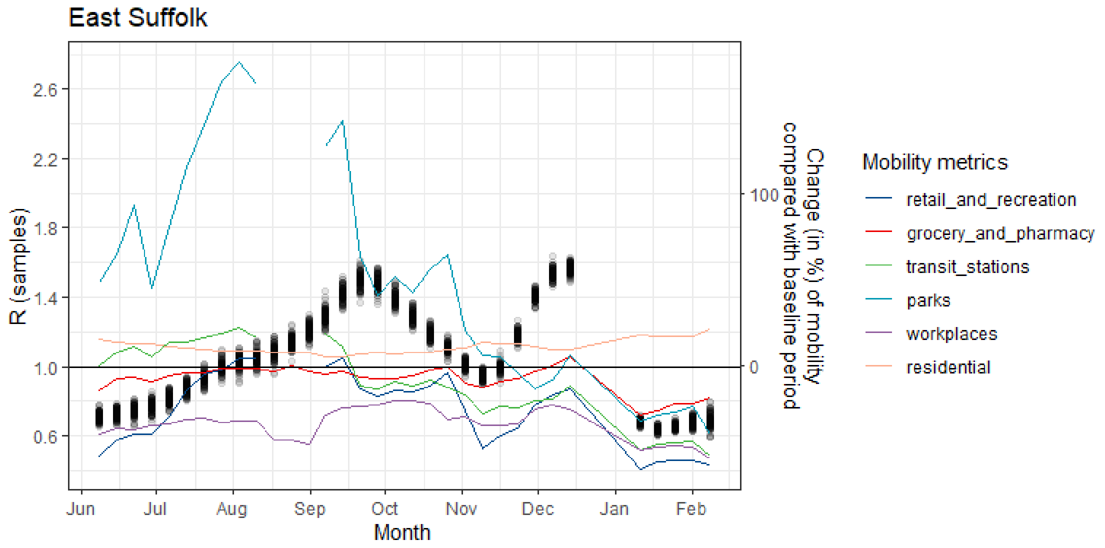
East Suffolk
R (samples)
Change (in %) of mobility compared with baseline period
Month
Jun
Jul
Aug
Sep
Oct
Nov
Dec
Jan
Feb
Mobility metrics
retail_and_recreation
grocery_and_pharmacy
transit_stations
parks
workplaces
residential

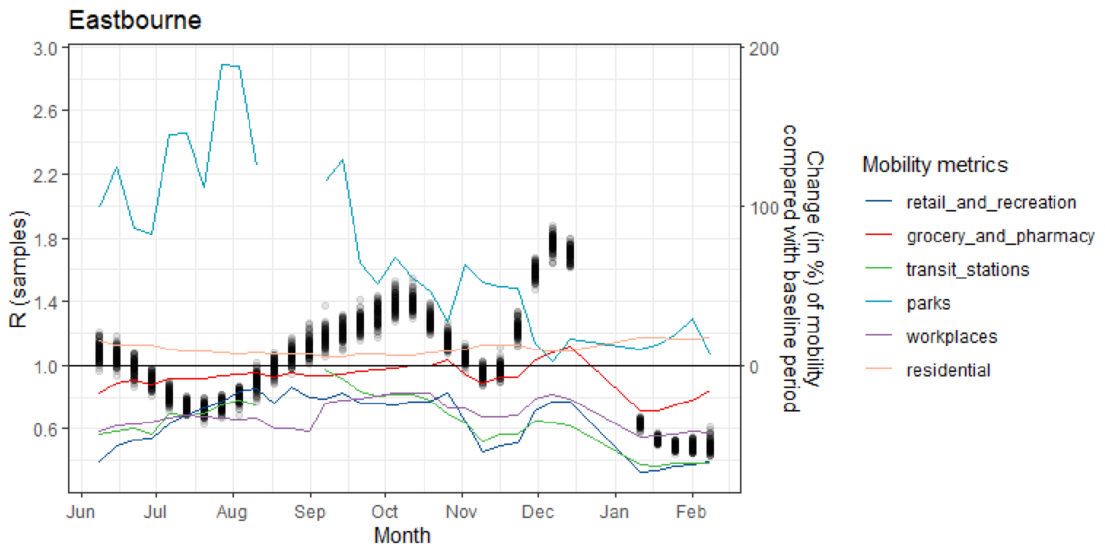
Eastbourne
R (samples)
Change (in %) of mobility compared with baseline period
Month
Jun
Jul
Aug
Sep
Oct
Nov
Dec
Jan
Feb
Mobility metrics
retail_and_recreation
grocery_and_pharmacy
transit_stations
parks
workplaces
residential

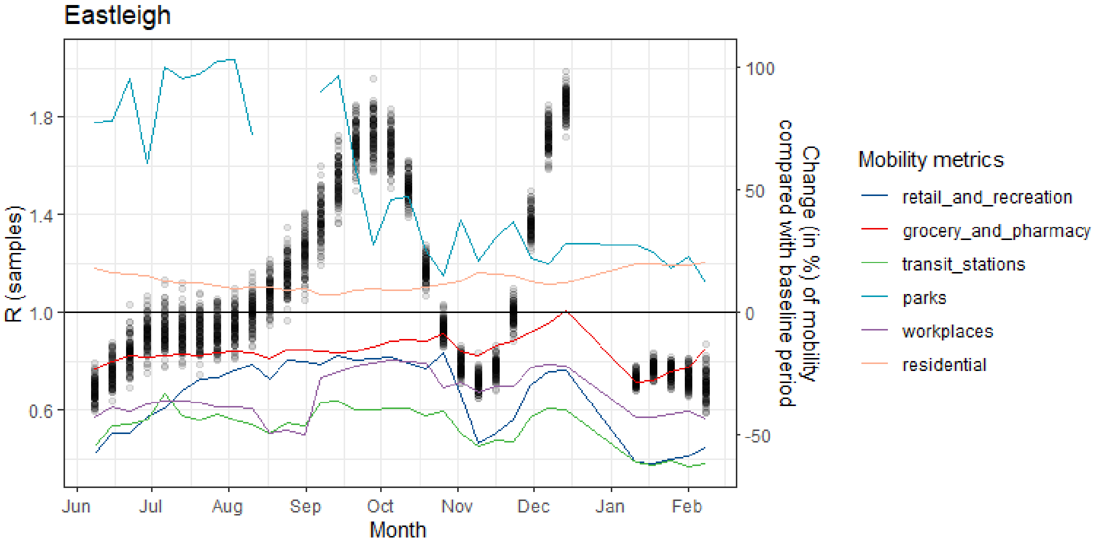
Eastleigh
R (samples)
1.8
1.4
1.0
0.6
Jun
Jul
Aug
Sep
Oct
Nov
Dec
Jan
Feb
Month
100
50
0
-50
Change (in %) of mobility compared with baseline period
Mobility metrics
retail_and_recreation
grocery_and_pharmacy
transit_stations
parks
workplaces
residential

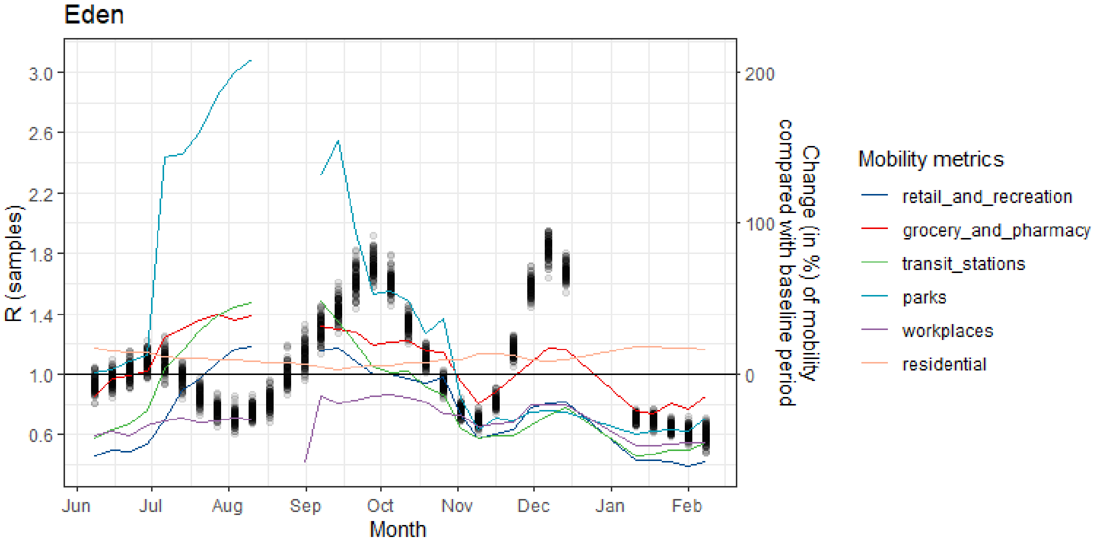
Eden
R (samples)
3.0
2.6
2.2
1.8
1.4
1.0
0.6
Jun
Jul
Aug
Sep
Oct
Nov
Dec
Jan
Feb
Month
200
100
0
Change (in %) of mobility compared with baseline period
Mobility metrics
retail_and_recreation
grocery_and_pharmacy
transit_stations
parks
workplaces
residential

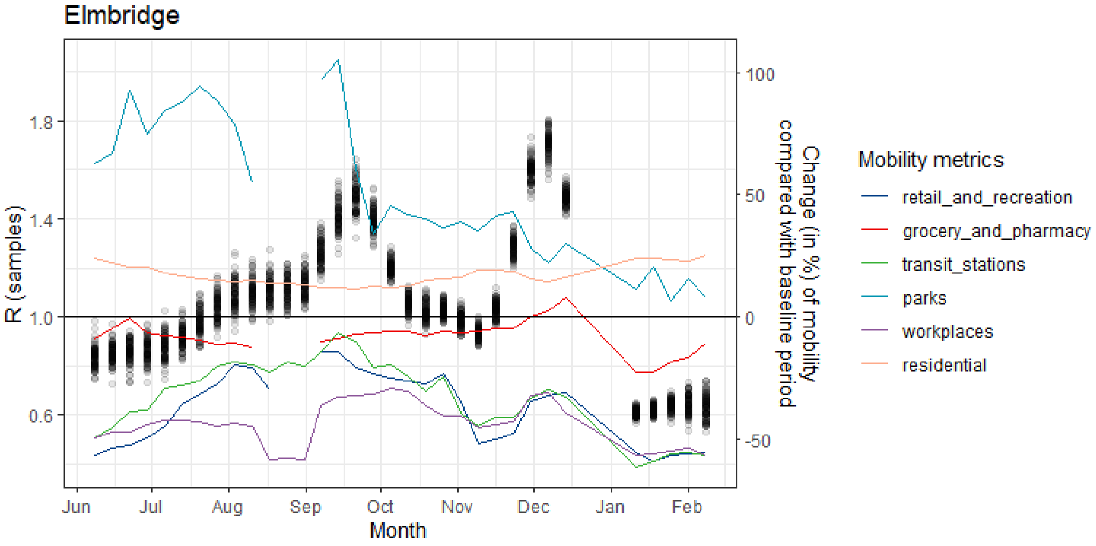
Elmbridge
R (samples)
1.8
1.4
1.0
0.6
Jun
Jul
Aug
Sep
Oct
Nov
Dec
Jan
Feb
Month
100
50
0
-50
Change (in %) of mobility compared with baseline period
Mobility metrics
retail_and_recreation
grocery_and_pharmacy
transit_stations
parks
workplaces
residential

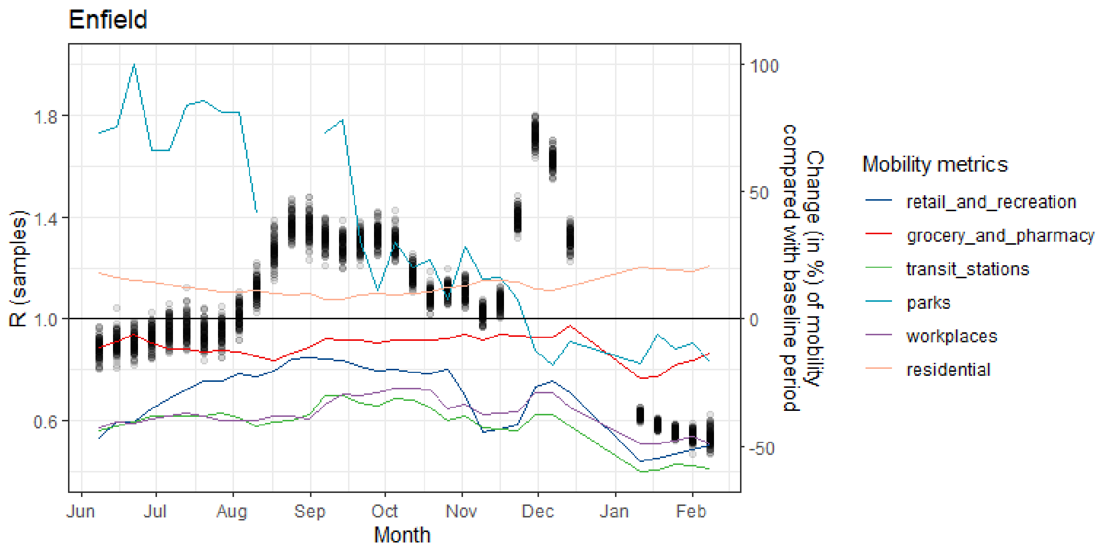
Enfield
R (samples)
1.8
1.4
1.0
0.6
Jun
Jul
Aug
Sep
Oct
Nov
Dec
Jan
Feb
Month
100
50
0
-50
Change (in %) of mobility compared with baseline period
Mobility metrics
retail_and_recreation
grocery_and_pharmacy
transit_stations
parks
workplaces
residential

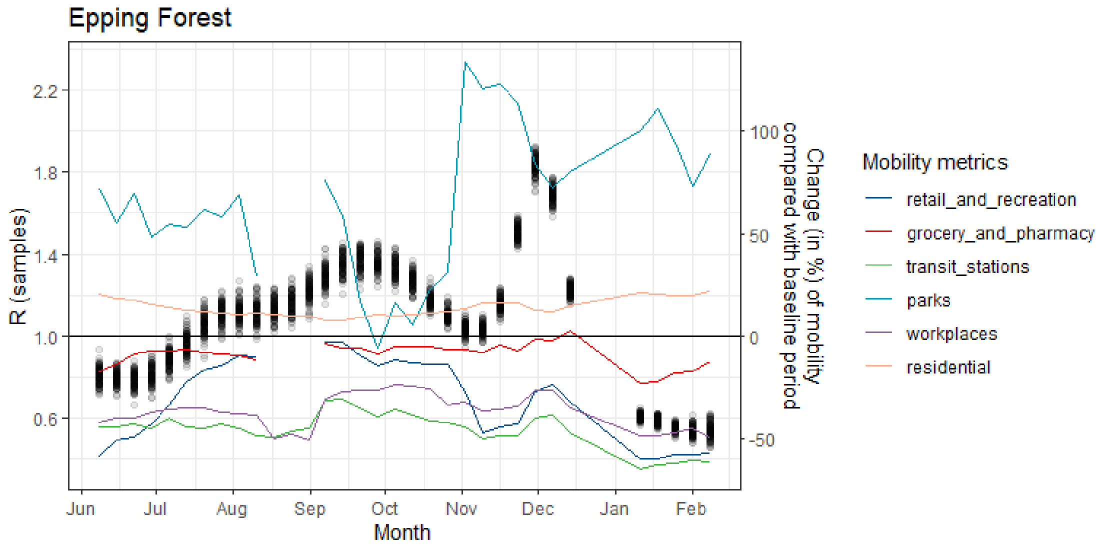
Epping Forest
R (samples)
2.2
1.8
1.4
1.0
0.6
Jun
Jul
Aug
Sep
Oct
Nov
Dec
Jan
Feb
Month
100
50
0
-50
Change (in %) of mobility compared with baseline period
Mobility metrics
retail_and_recreation
grocery_and_pharmacy
transit_stations
parks
workplaces
residential

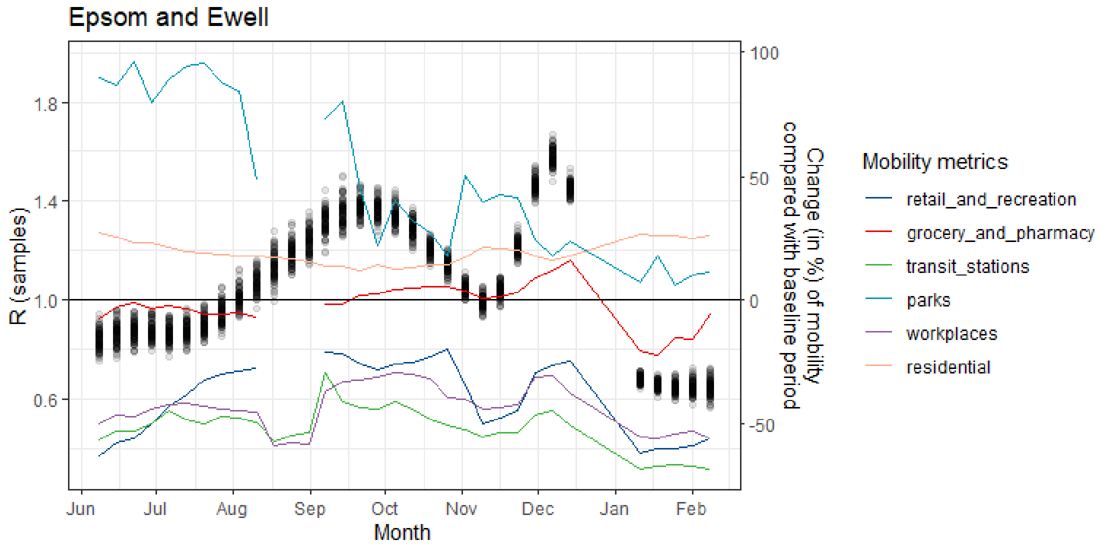
Epsom and Ewell
R (samples)
1.8
1.4
1.0
0.6
Jun
Jul
Aug
Sep
Oct
Nov
Dec
Jan
Feb
Month
100
50
0
-50
Change (in %) of mobility compared with baseline period
Mobility metrics
retail_and_recreation
grocery_and_pharmacy
transit_stations
parks
workplaces
residential

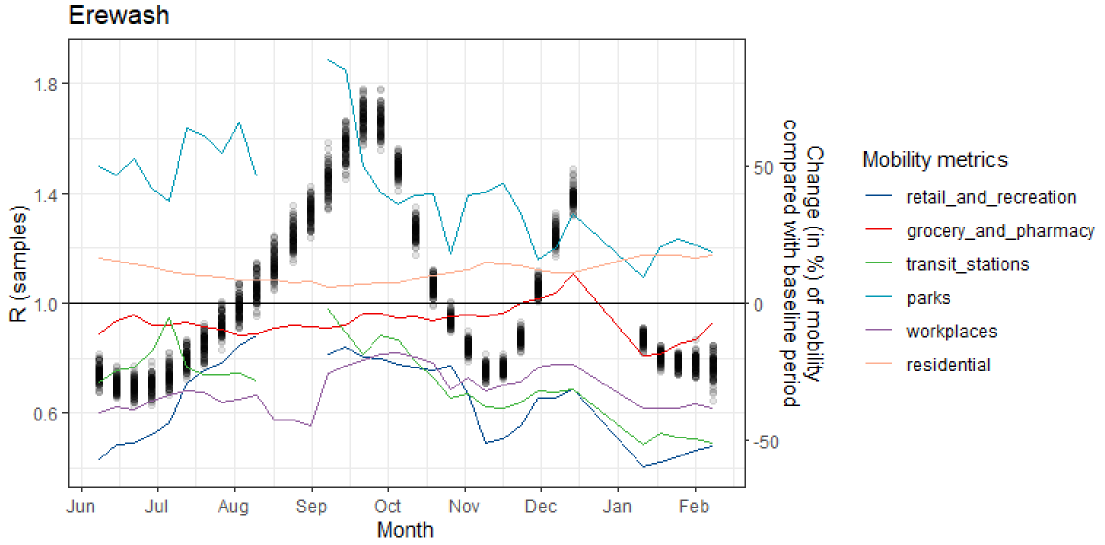
Erewash
R (samples)
1.8
1.4
1.0
0.6
Change (in %) of mobility compared with baseline period
50
0
-50
Jun Jul Aug Sep Oct Nov Dec Jan Feb
Month
Mobility metrics
retail_and_recreation
grocery_and_pharmacy
transit_stations
parks
workplaces
residential

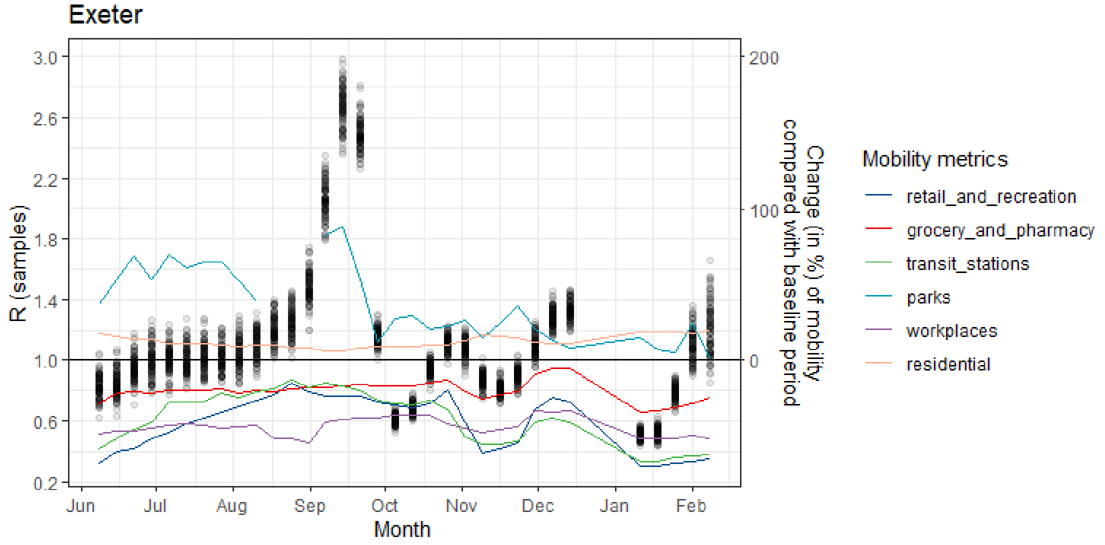
Exeter
R (samples)
3.0
2.6
2.2
1.8
1.4
1.0
0.6
0.2
Change (in %) of mobility compared with baseline period
200
100
0
Jun Jul Aug Sep Oct Nov Dec Jan Feb
Month
Mobility metrics
retail_and_recreation
grocery_and_pharmacy
transit_stations
parks
workplaces
residential

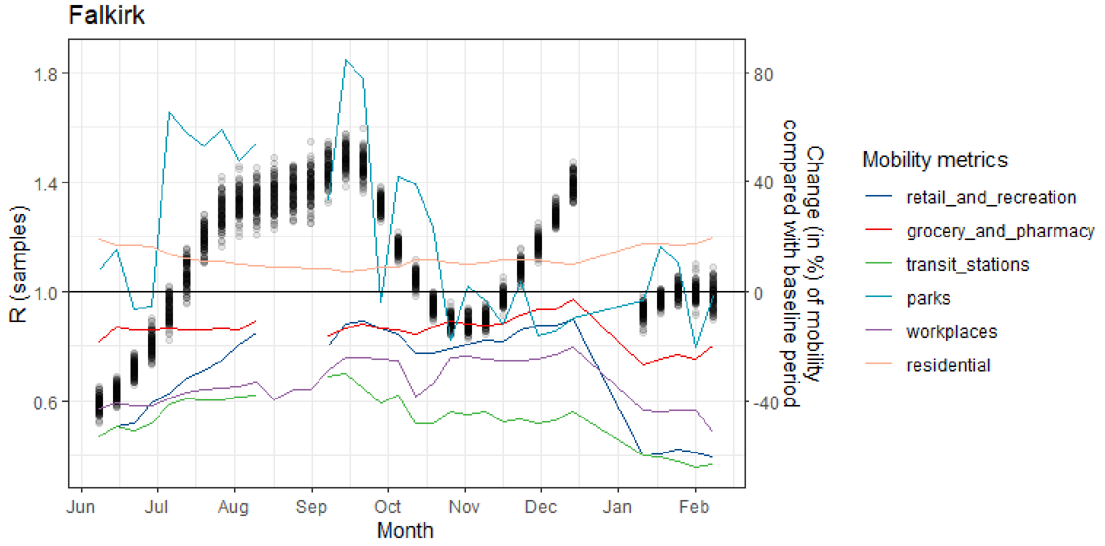
Falkirk
R (samples)
1.8
1.4
1.0
0.6
Change (in %) of mobility compared with baseline period
80
40
0
-40
Jun Jul Aug Sep Oct Nov Dec Jan Feb
Month
Mobility metrics
retail_and_recreation
grocery_and_pharmacy
transit_stations
parks
workplaces
residential

Fareham

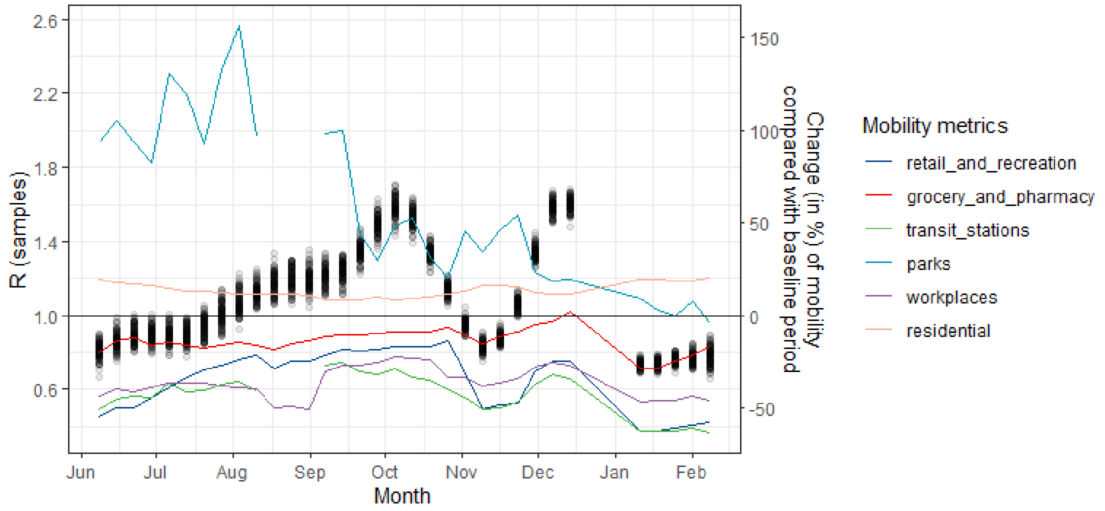

Fenland

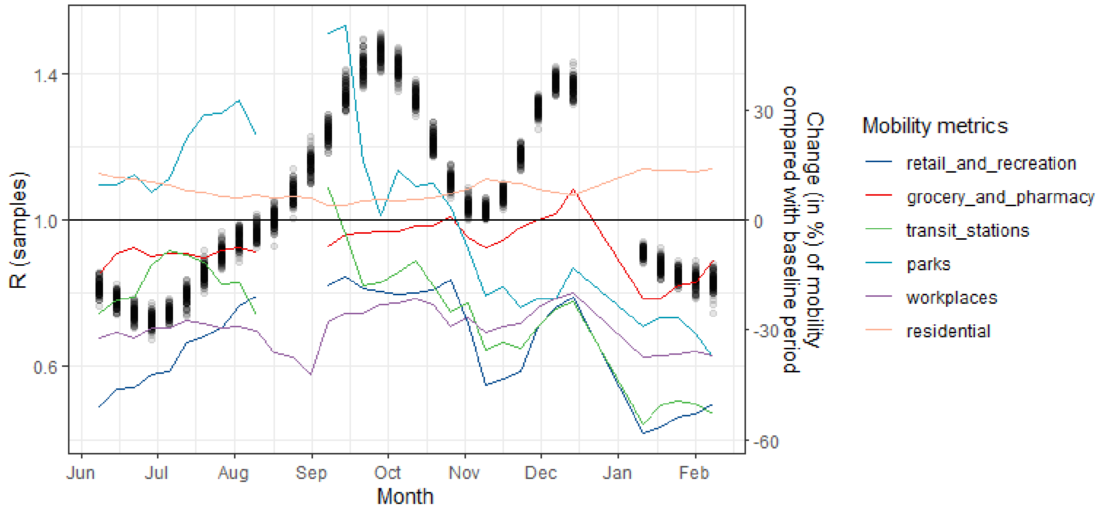

Fermanagh and Omagh

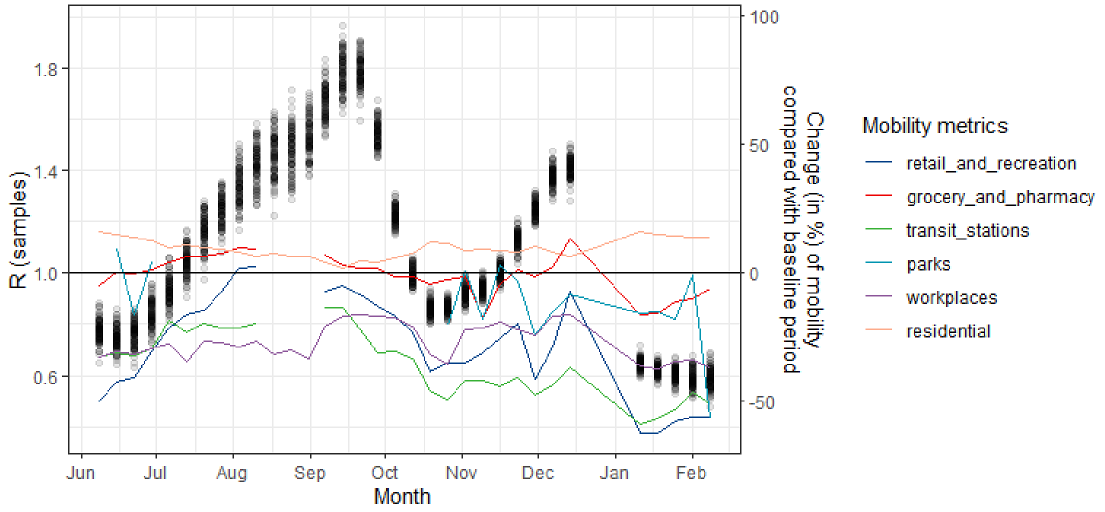

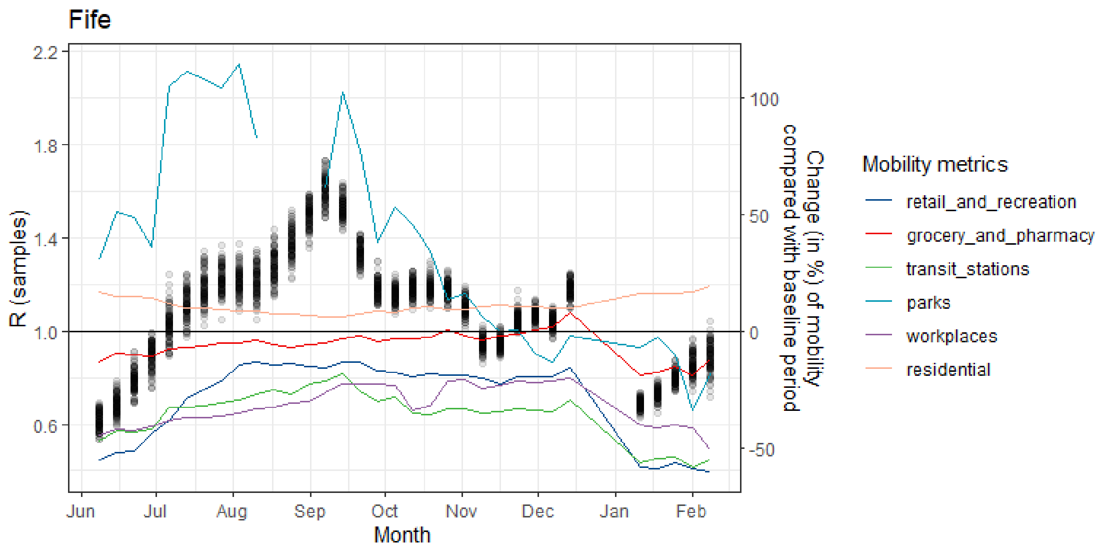
Fife
R (samples)
2.2
1.8
1.4
1.0
0.6
100
50
0
-50
Change (in %) of mobility compared with baseline period
Jun Jul Aug Sep Oct Nov Dec Jan Feb
Month
Mobility metrics
retail_and_recreation
grocery_and_pharmacy
transit_stations
parks
workplaces
residential

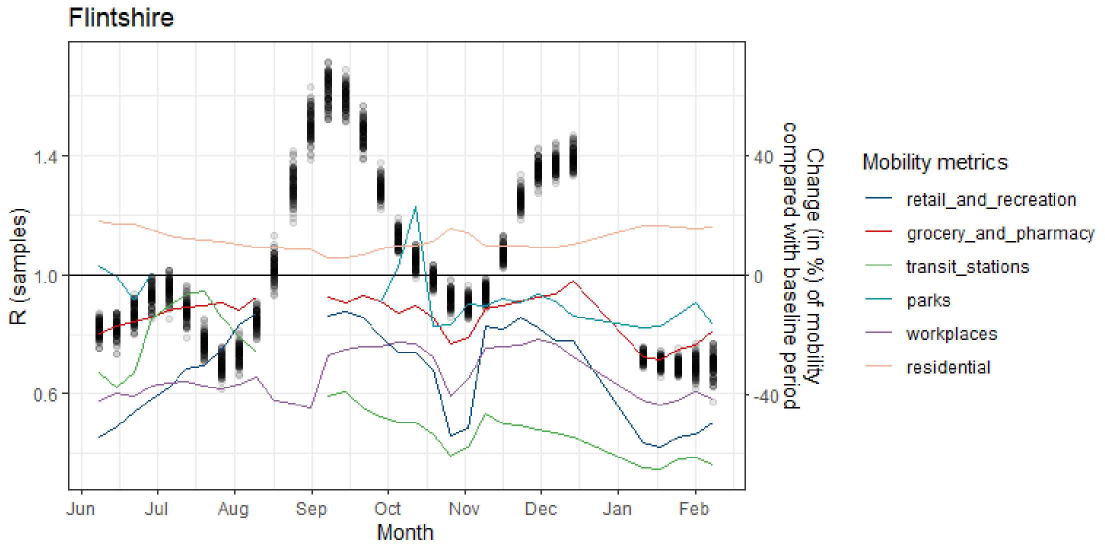
Flintshire
R (samples)
1.4
1.0
0.6
40
0
-40
Change (in %) of mobility compared with baseline period
Jun Jul Aug Sep Oct Nov Dec Jan Feb
Month
Mobility metrics
retail_and_recreation
grocery_and_pharmacy
transit_stations
parks
workplaces
residential

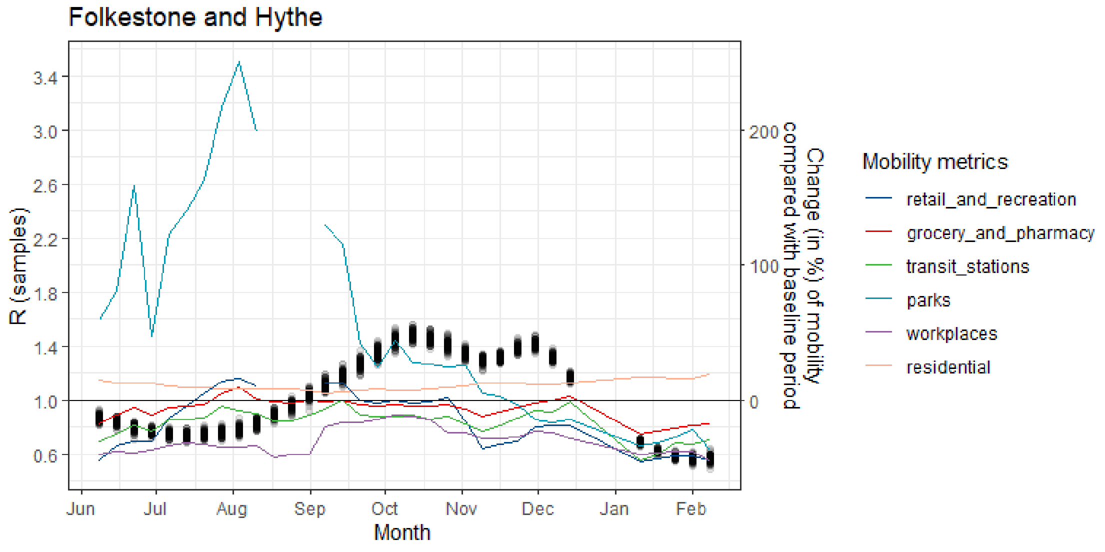
Folkestone and Hythe
R (samples)
3.4
3.0
2.6
2.2
1.8
1.4
1.0
0.6
200
100
0
Change (in %) of mobility compared with baseline period
Jun Jul Aug Sep Oct Nov Dec Jan Feb
Month
Mobility metrics
retail_and_recreation
grocery_and_pharmacy
transit_stations
parks
workplaces
residential

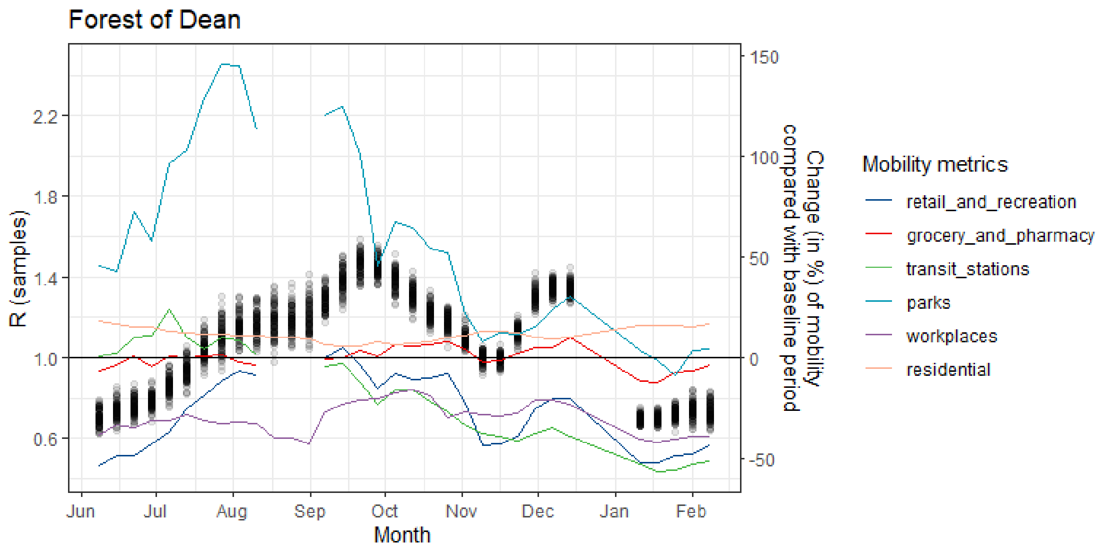
Forest of Dean
R (samples)
2.2
1.8
1.4
1.0
0.6
150
100
50
0
-50
Change (in %) of mobility compared with baseline period
Jun
Jul
Aug
Sep
Oct
Nov
Dec
Jan
Feb
Month
Mobility metrics
retail_and_recreation
grocery_and_pharmacy
transit_stations
parks
workplaces
residential

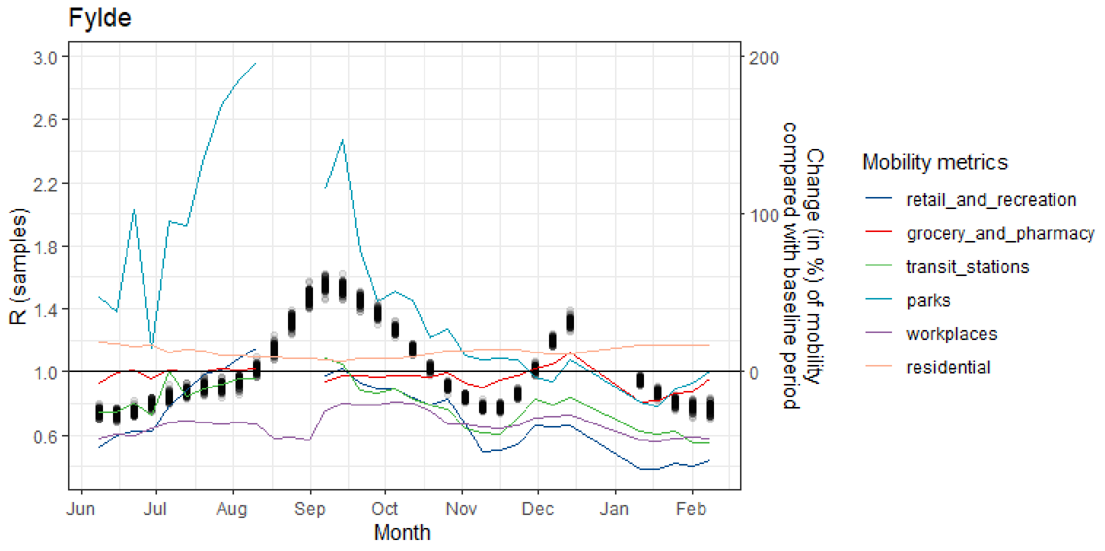
Fylde
R (samples)
3.0
2.6
2.2
1.8
1.4
1.0
0.6
200
100
0
Change (in %) of mobility compared with baseline period
Jun
Jul
Aug
Sep
Oct
Nov
Dec
Jan
Feb
Month
Mobility metrics
retail_and_recreation
grocery_and_pharmacy
transit_stations
parks
workplaces
residential

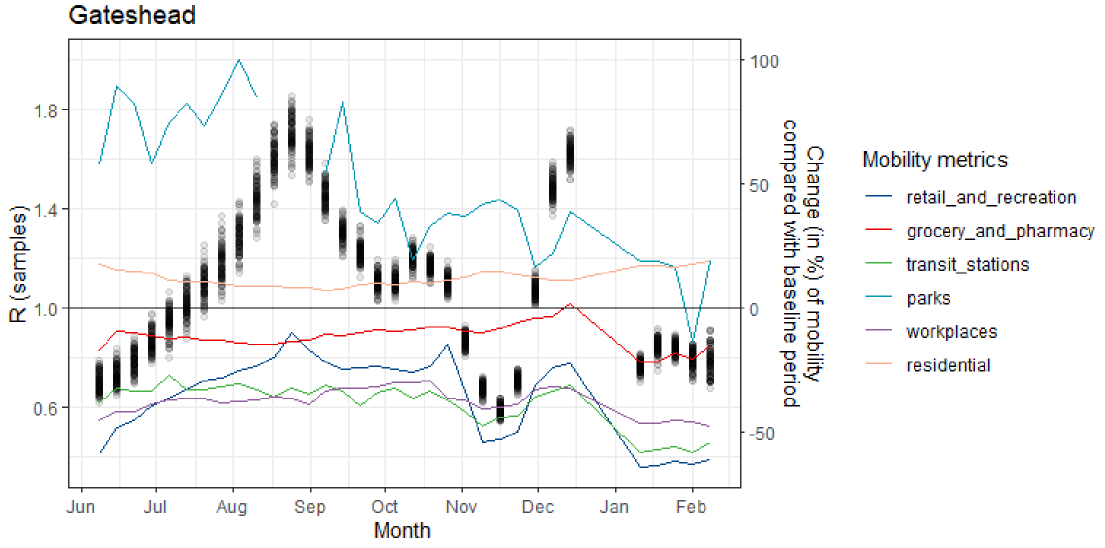
Gateshead
R (samples)
1.8
1.4
1.0
0.6
100
50
0
-50
Change (in %) of mobility compared with baseline period
Jun
Jul
Aug
Sep
Oct
Nov
Dec
Jan
Feb
Month
Mobility metrics
retail_and_recreation
grocery_and_pharmacy
transit_stations
parks
workplaces
residential

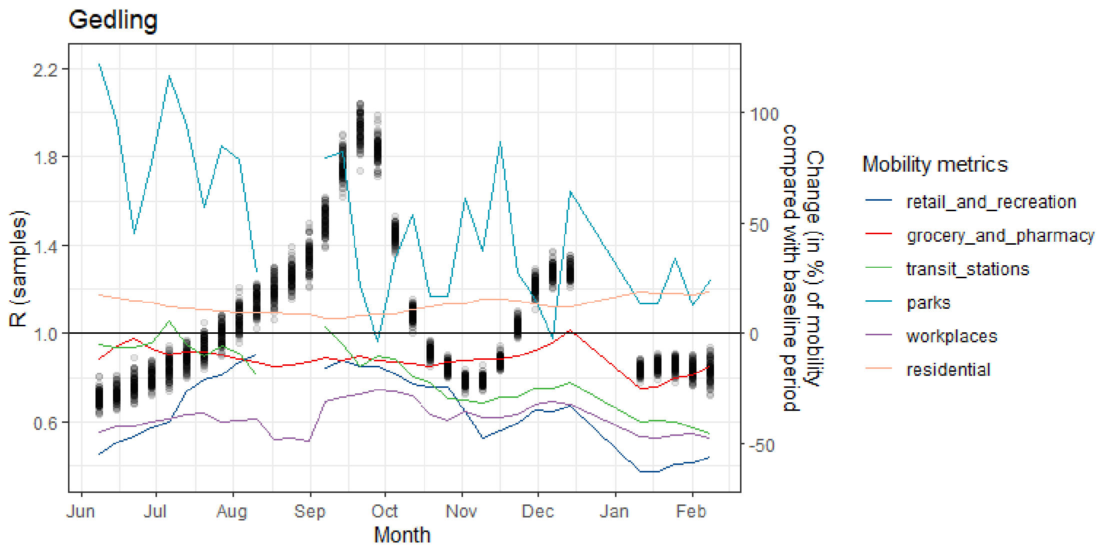
Gedling
R (samples)
2.2
1.8
1.4
1.0
0.6
Jun
Jul
Aug
Sep
Oct
Nov
Dec
Jan
Feb
Month
100
50
0
-50
Change (in %) of mobility compared with baseline period
Mobility metrics
retail_and_recreation
grocery_and_pharmacy
transit_stations
parks
workplaces
residential

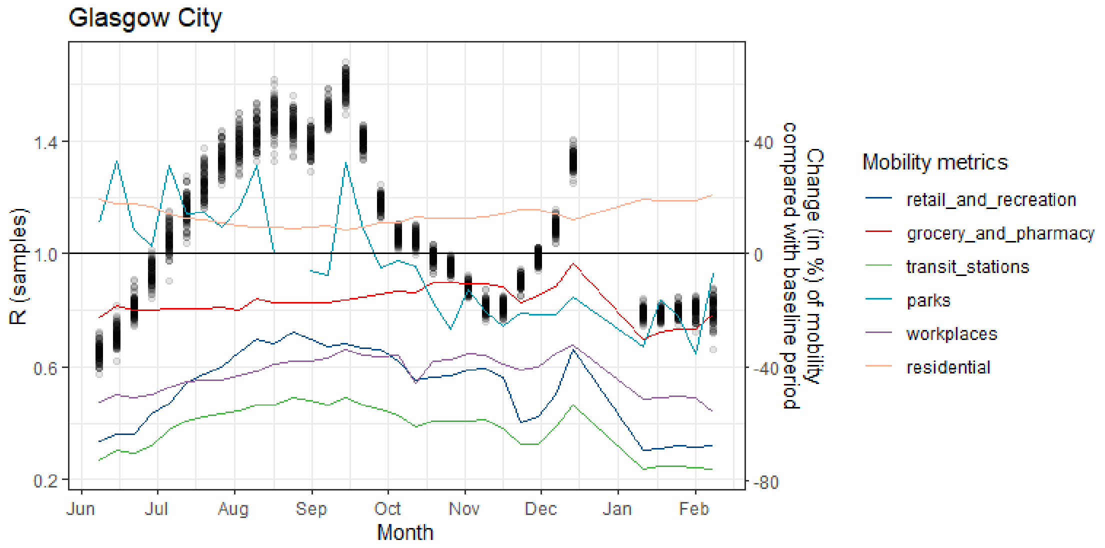
Glasgow City
R (samples)
1.4
1.0
0.6
0.2
Jun
Jul
Aug
Sep
Oct
Nov
Dec
Jan
Feb
Month
40
0
-40
-80
Change (in %) of mobility compared with baseline period
Mobility metrics
retail_and_recreation
grocery_and_pharmacy
transit_stations
parks
workplaces
residential

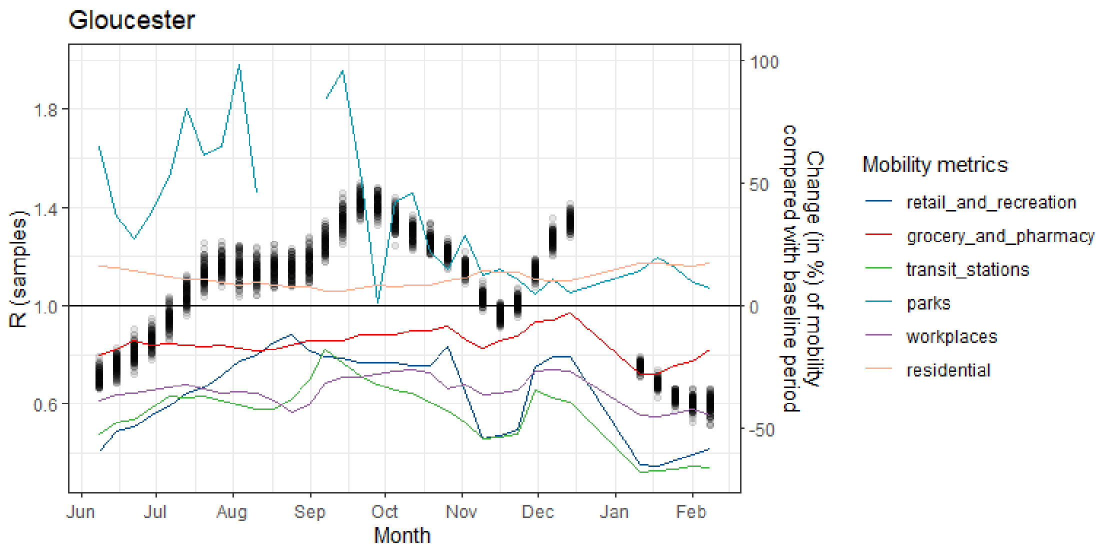
Gloucester
R (samples)
1.8
1.4
1.0
0.6
Jun
Jul
Aug
Sep
Oct
Nov
Dec
Jan
Feb
Month
100
50
0
-50
Change (in %) of mobility compared with baseline period
Mobility metrics
retail_and_recreation
grocery_and_pharmacy
transit_stations
parks
workplaces
residential

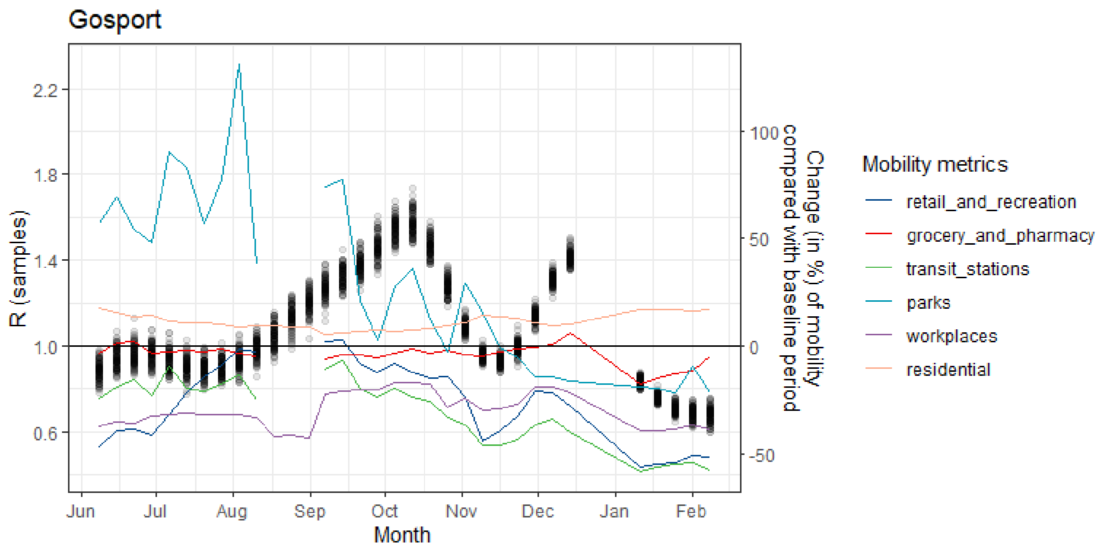
Gosport
R (samples)
Change (in %) of mobility compared with baseline period
Month
Jun
Jul
Aug
Sep
Oct
Nov
Dec
Jan
Feb
Mobility metrics
retail_and_recreation
grocery_and_pharmacy
transit_stations
parks
workplaces
residential

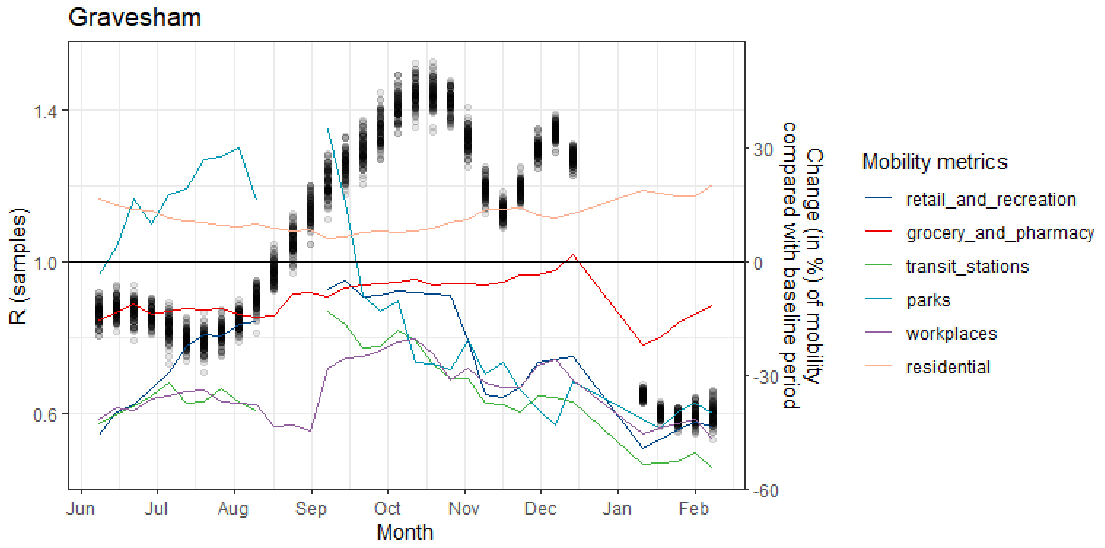
Gravesham
R (samples)
Change (in %) of mobility compared with baseline period
Month
Jun
Jul
Aug
Sep
Oct
Nov
Dec
Jan
Feb
Mobility metrics
retail_and_recreation
grocery_and_pharmacy
transit_stations
parks
workplaces
residential

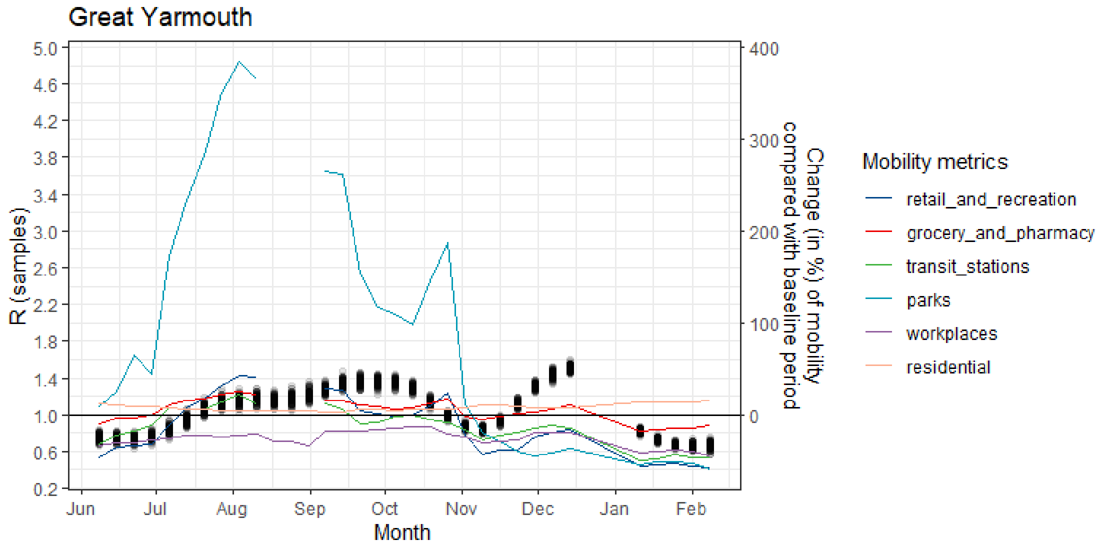
Great Yarmouth
R (samples)
Change (in %) of mobility compared with baseline period
Month
Jun
Jul
Aug
Sep
Oct
Nov
Dec
Jan
Feb
Mobility metrics
retail_and_recreation
grocery_and_pharmacy
transit_stations
parks
workplaces
residential

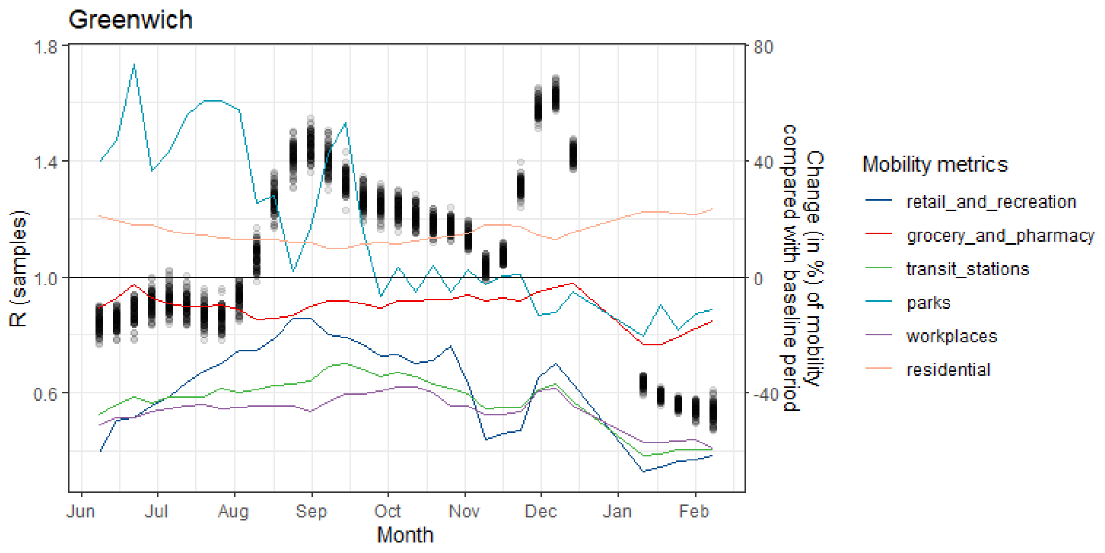
Greenwich
R (samples)
Change (in %) of mobility compared with baseline period
Month
Jun
Jul
Aug
Sep
Oct
Nov
Dec
Jan
Feb
Mobility metrics
retail_and_recreation
grocery_and_pharmacy
transit_stations
parks
workplaces
residential

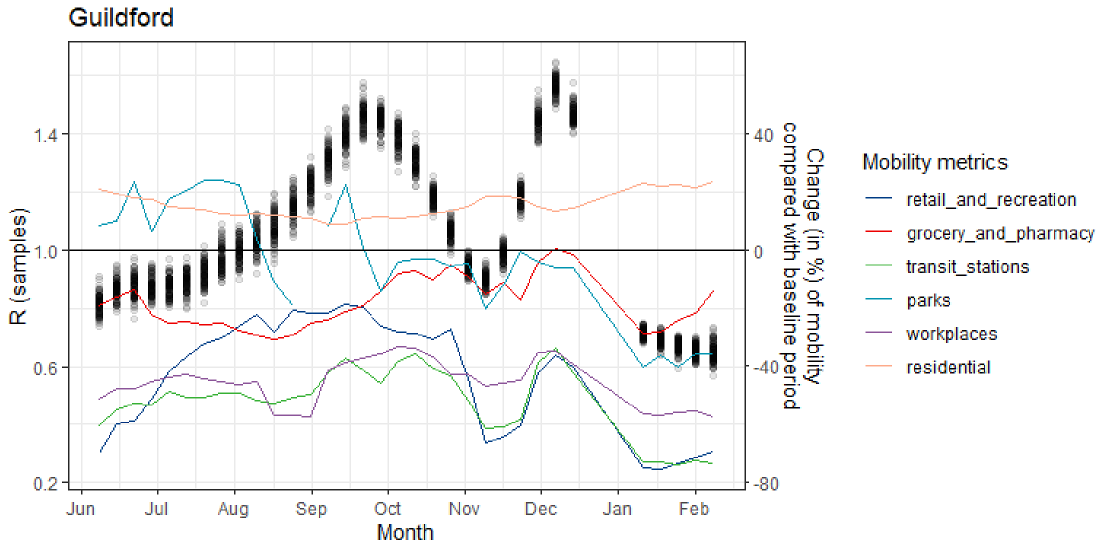
Guildford
R (samples)
Change (in %) of mobility compared with baseline period
Month
Jun
Jul
Aug
Sep
Oct
Nov
Dec
Jan
Feb
Mobility metrics
retail_and_recreation
grocery_and_pharmacy
transit_stations
parks
workplaces
residential

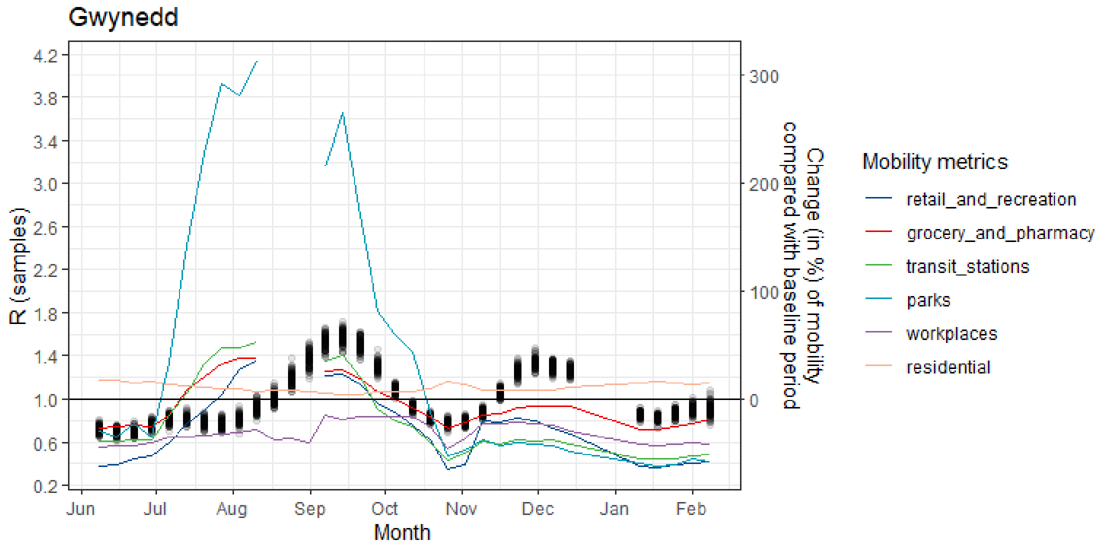
Gwynedd
R (samples)
Change (in %) of mobility compared with baseline period
Month
Jun
Jul
Aug
Sep
Oct
Nov
Dec
Jan
Feb
Mobility metrics
retail_and_recreation
grocery_and_pharmacy
transit_stations
parks
workplaces
residential

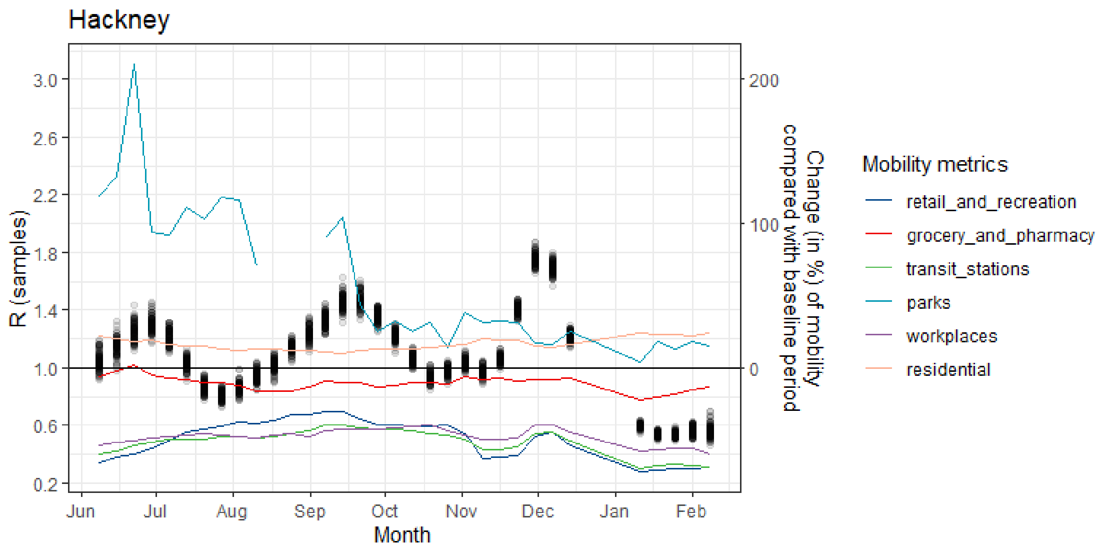
Hackney
R (samples)
3.0
2.6
2.2
1.8
1.4
1.0
0.6
0.2
200
100
0
Change (in %) of mobility compared with baseline period
Jun
Jul
Aug
Sep
Oct
Nov
Dec
Jan
Feb
Month
Mobility metrics
retail_and_recreation
grocery_and_pharmacy
transit_stations
parks
workplaces
residential

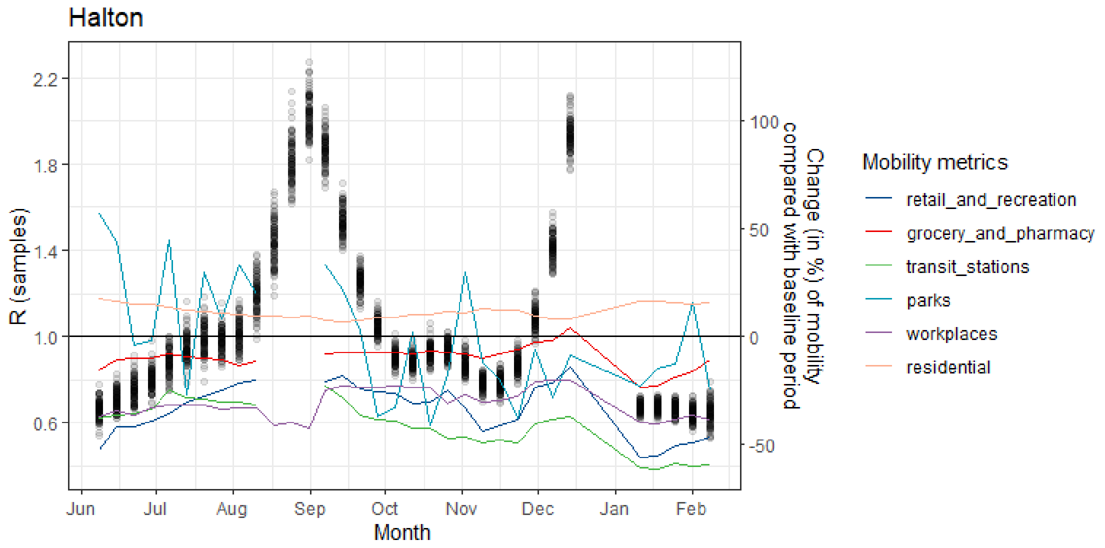
Halton
R (samples)
2.2
1.8
1.4
1.0
0.6
100
50
0
-50
Change (in %) of mobility compared with baseline period
Jun
Jul
Aug
Sep
Oct
Nov
Dec
Jan
Feb
Month
Mobility metrics
retail_and_recreation
grocery_and_pharmacy
transit_stations
parks
workplaces
residential

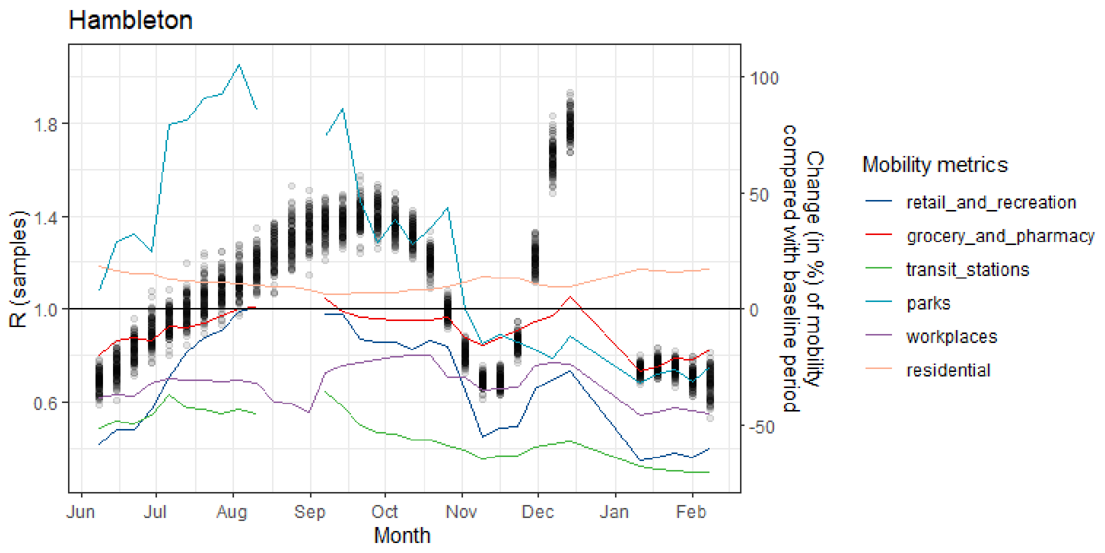
Hambleton
R (samples)
1.8
1.4
1.0
0.6
100
50
0
-50
Change (in %) of mobility compared with baseline period
Jun
Jul
Aug
Sep
Oct
Nov
Dec
Jan
Feb
Month
Mobility metrics
retail_and_recreation
grocery_and_pharmacy
transit_stations
parks
workplaces
residential

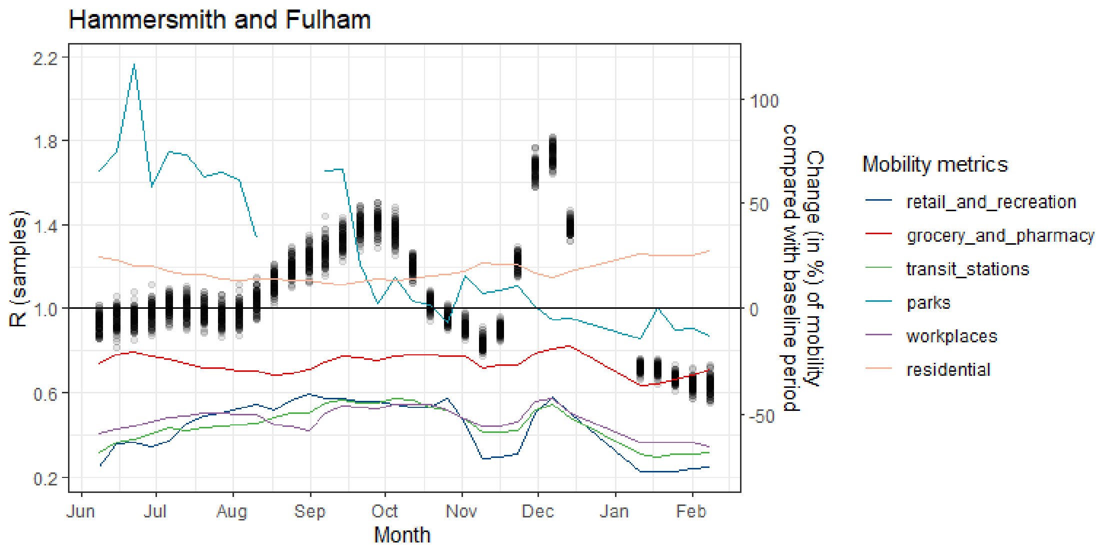
Hammersmith and Fulham
R (samples)
2.2
1.8
1.4
1.0
0.6
0.2
Jun
Jul
Aug
Sep
Oct
Nov
Dec
Jan
Feb
Month
100
50
0
-50
Change (in %) of mobility compared with baseline period
Mobility metrics
retail_and_recreation
grocery_and_pharmacy
transit_stations
parks
workplaces
residential

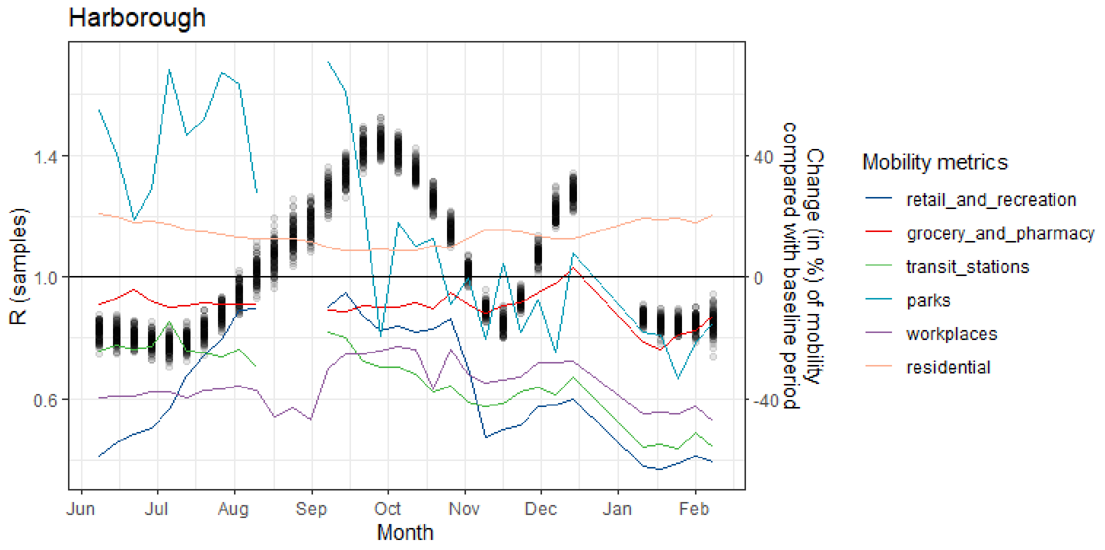
Harborough
R (samples)
1.4
1.0
0.6
Jun
Jul
Aug
Sep
Oct
Nov
Dec
Jan
Feb
Month
40
0
-40
Change (in %) of mobility compared with baseline period
Mobility metrics
retail_and_recreation
grocery_and_pharmacy
transit_stations
parks
workplaces
residential

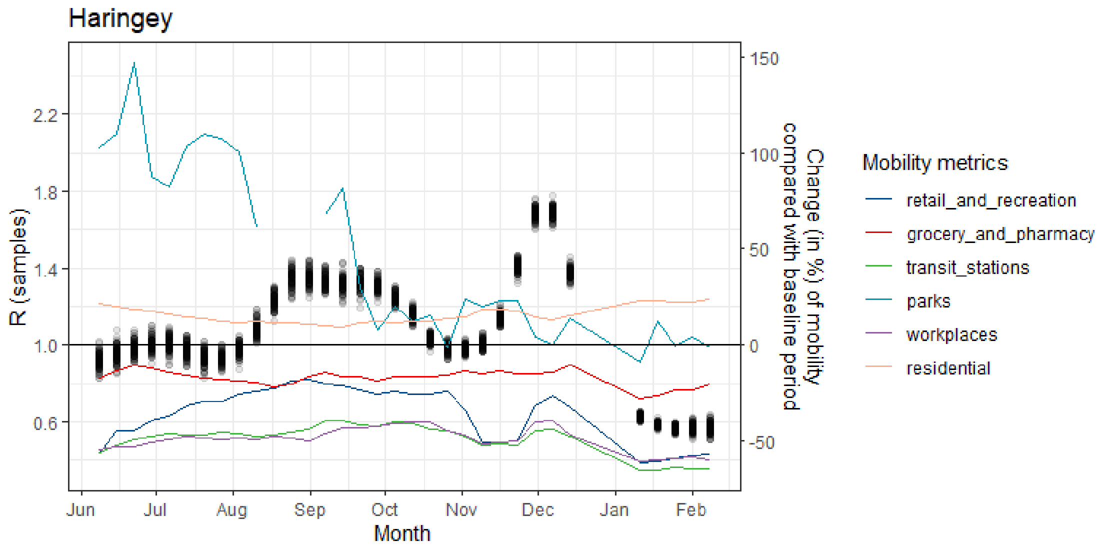
Haringey
R (samples)
2.2
1.8
1.4
1.0
0.6
Jun
Jul
Aug
Sep
Oct
Nov
Dec
Jan
Feb
Month
150
100
50
0
-50
Change (in %) of mobility compared with baseline period
Mobility metrics
retail_and_recreation
grocery_and_pharmacy
transit_stations
parks
workplaces
residential

Harlow

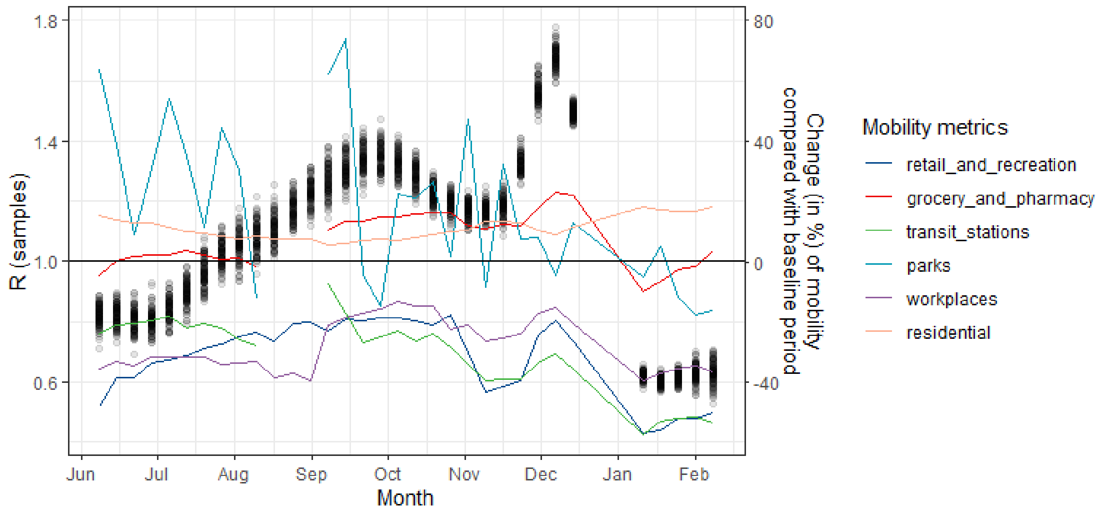

Harrogate

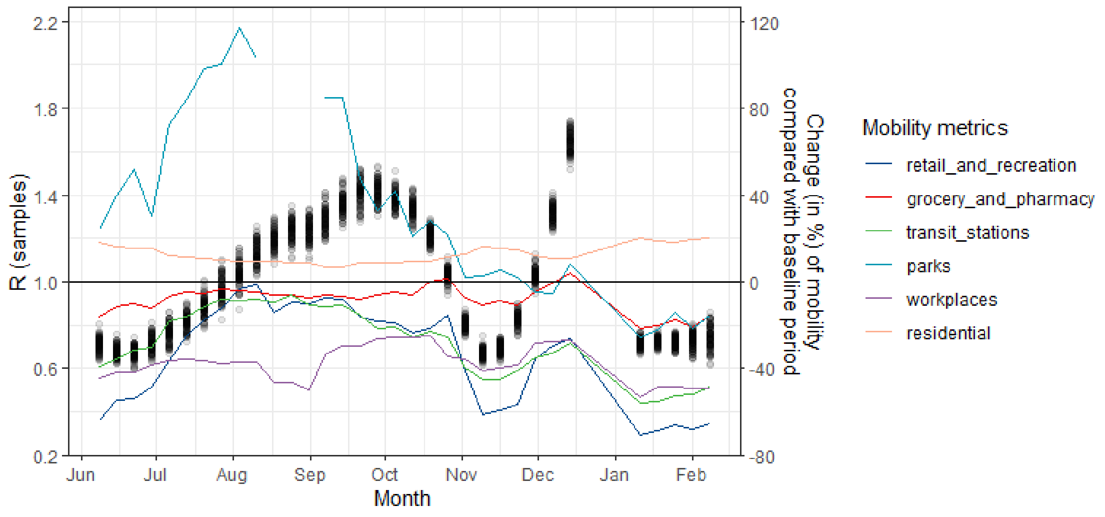

Harrow

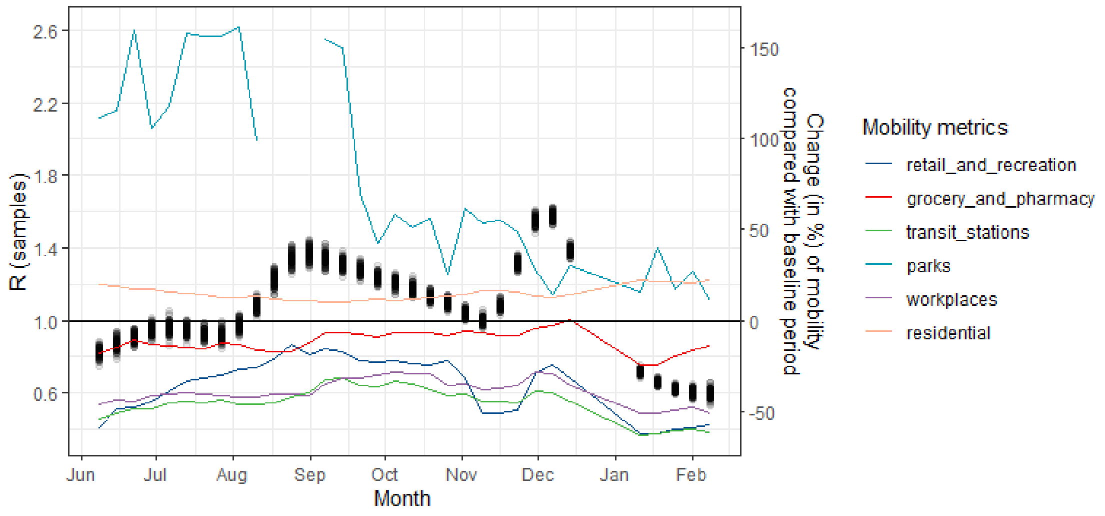

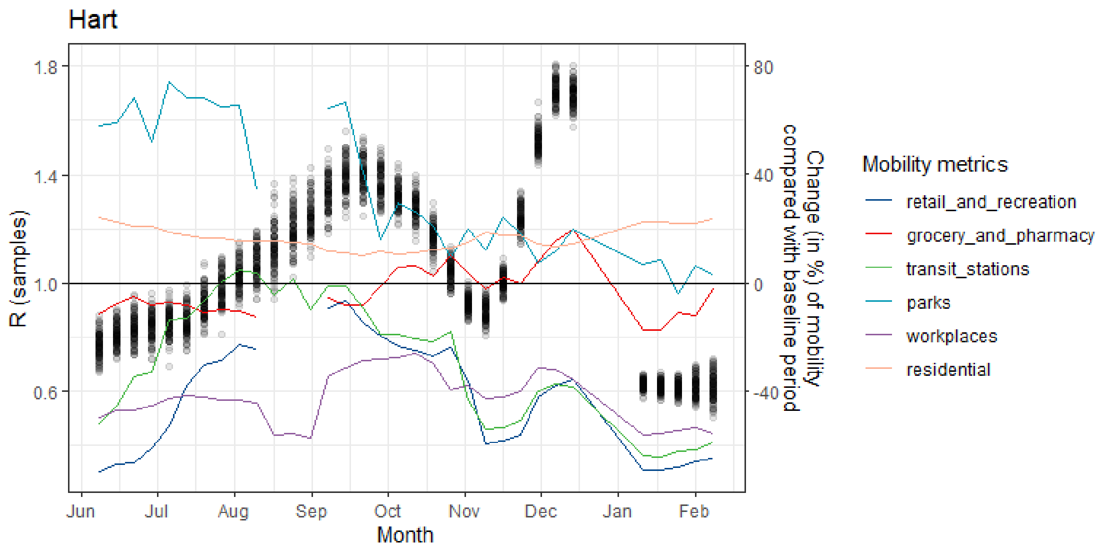
Hart
R (samples)
1.8
1.4
1.0
0.6
80
40
0
-40
Change (in %) of mobility compared with baseline period
Jun
Jul
Aug
Sep
Oct
Nov
Dec
Jan
Feb
Month
Mobility metrics
retail_and_recreation
grocery_and_pharmacy
transit_stations
parks
workplaces
residential

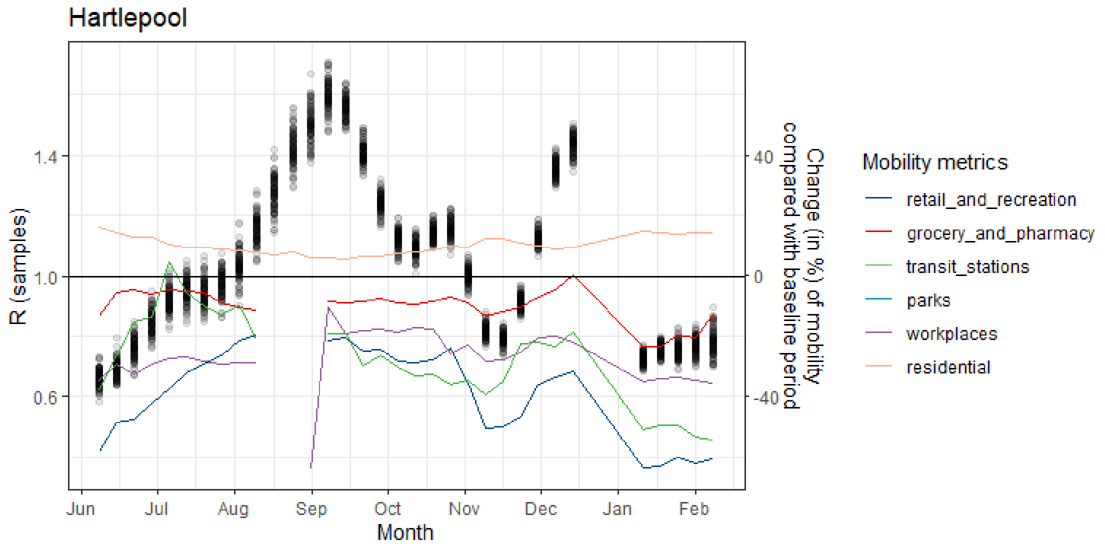
Hartlepool
R (samples)
1.4
1.0
0.6
40
0
-40
Change (in %) of mobility compared with baseline period
Jun
Jul
Aug
Sep
Oct
Nov
Dec
Jan
Feb
Month
Mobility metrics
retail_and_recreation
grocery_and_pharmacy
transit_stations
parks
workplaces
residential

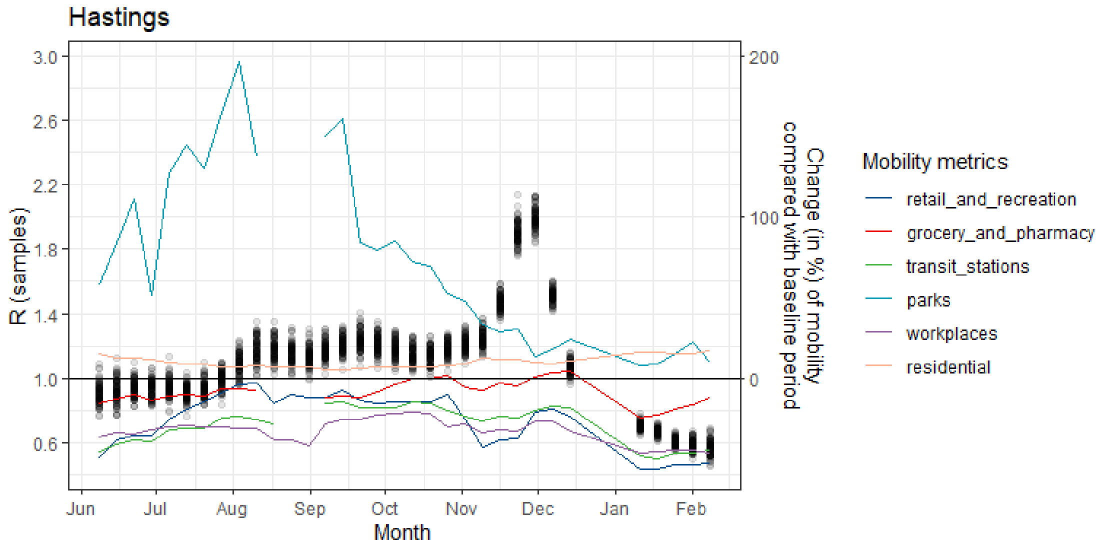
Hastings
R (samples)
3.0
2.6
2.2
1.8
1.4
1.0
0.6
200
100
0
Change (in %) of mobility compared with baseline period
Jun
Jul
Aug
Sep
Oct
Nov
Dec
Jan
Feb
Month
Mobility metrics
retail_and_recreation
grocery_and_pharmacy
transit_stations
parks
workplaces
residential

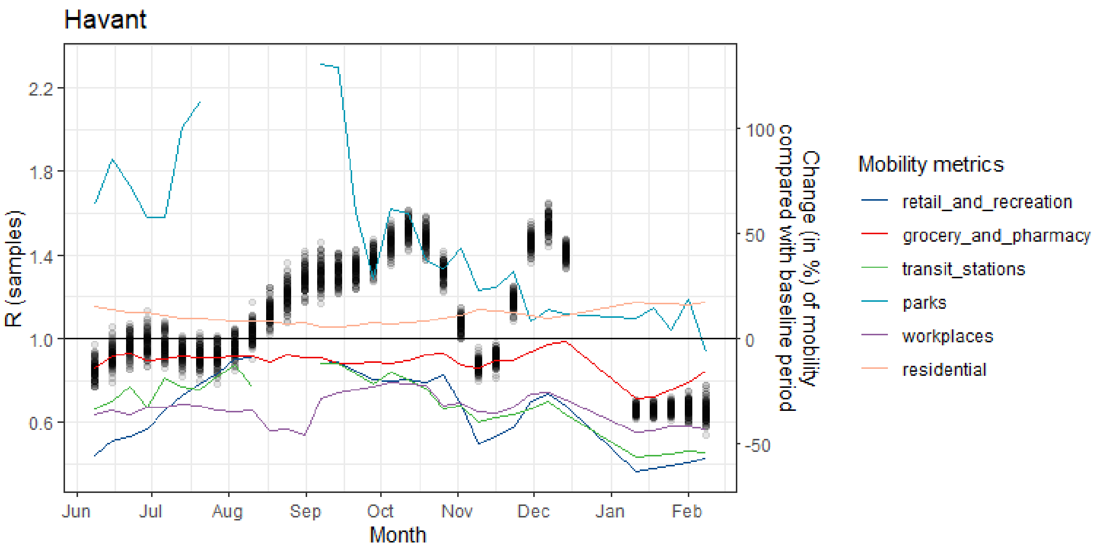
Havant
R (samples)
2.2
1.8
1.4
1.0
0.6
Jun Jul Aug Sep Oct Nov Dec Jan Feb
Month
100
50
0
-50
Change (in %) of mobility compared with baseline period
Mobility metrics
retail_and_recreation
grocery_and_pharmacy
transit_stations
parks
workplaces
residential

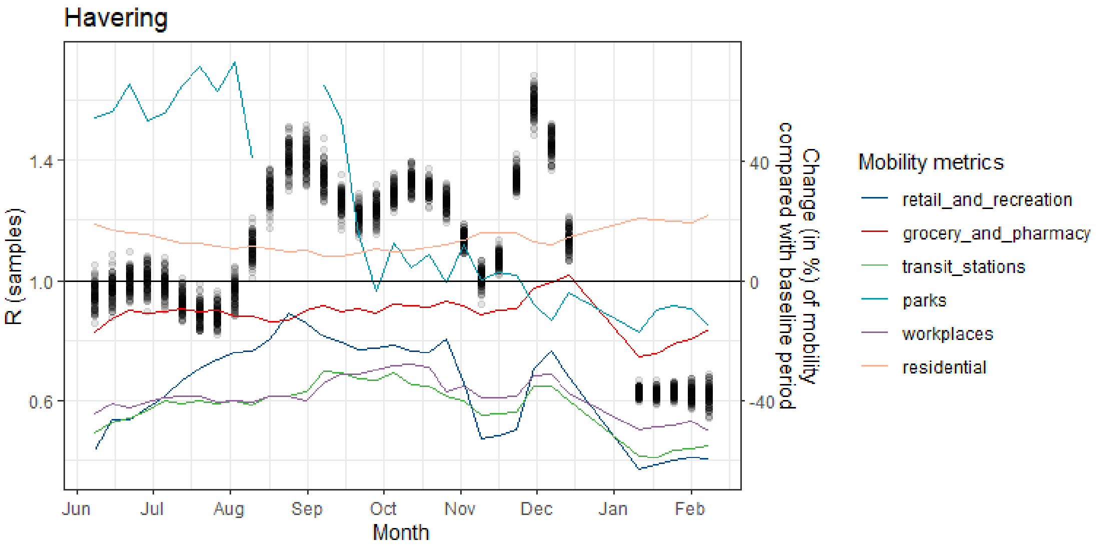
Havering
R (samples)
1.4
1.0
0.6
Jun Jul Aug Sep Oct Nov Dec Jan Feb
Month
40
0
-40
Change (in %) of mobility compared with baseline period
Mobility metrics
retail_and_recreation
grocery_and_pharmacy
transit_stations
parks
workplaces
residential

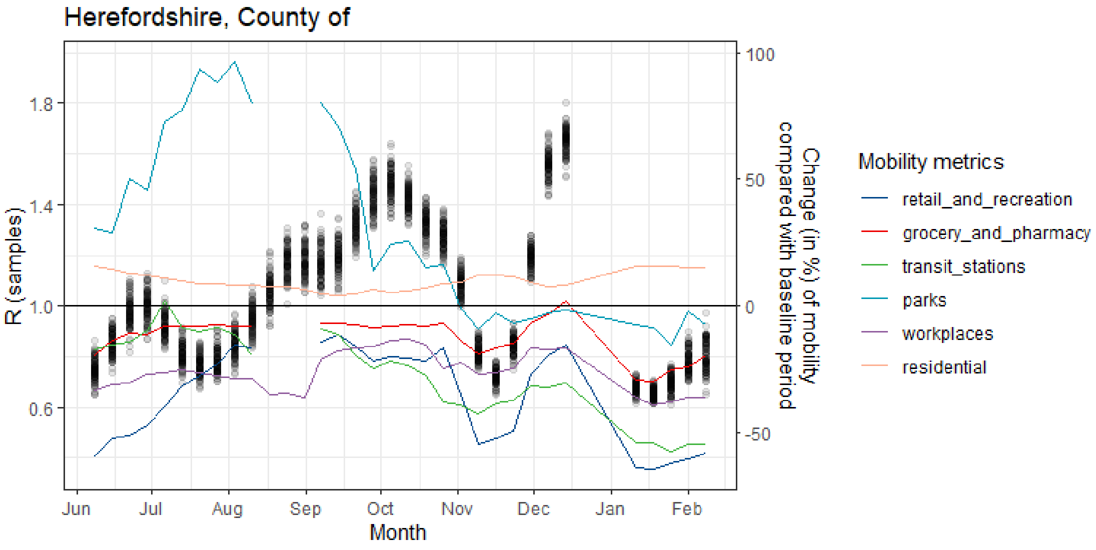
Herefordshire, County of
R (samples)
1.8
1.4
1.0
0.6
Jun Jul Aug Sep Oct Nov Dec Jan Feb
Month
100
50
0
-50
Change (in %) of mobility compared with baseline period
Mobility metrics
retail_and_recreation
grocery_and_pharmacy
transit_stations
parks
workplaces
residential

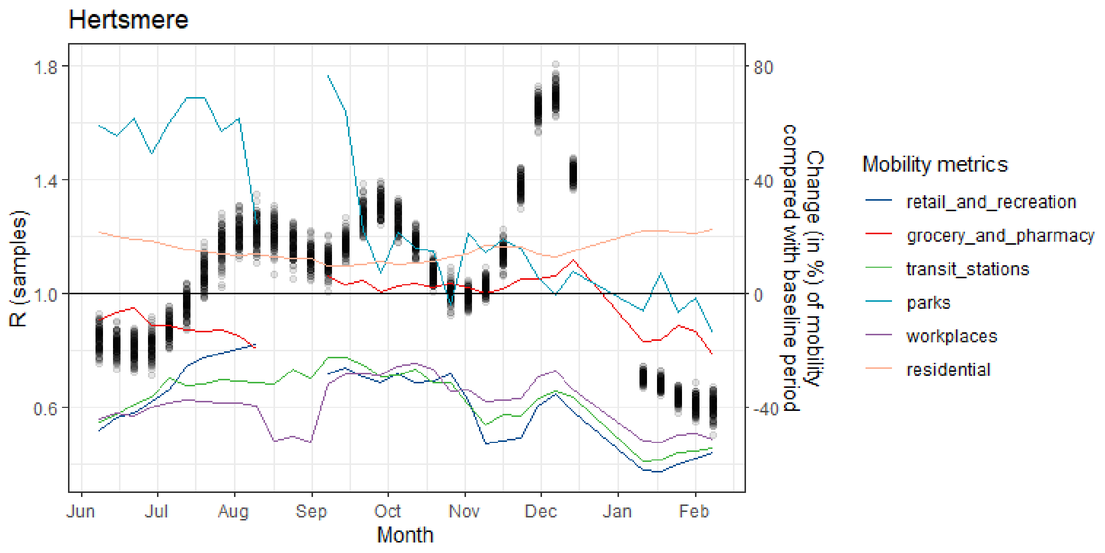
Hertsmere
R (samples)
1.8
1.4
1.0
0.6
80
40
0
-40
Change (in %) of mobility compared with baseline period
Jun
Jul
Aug
Sep
Oct
Nov
Dec
Jan
Feb
Month
Mobility metrics
retail_and_recreation
grocery_and_pharmacy
transit_stations
parks
workplaces
residential

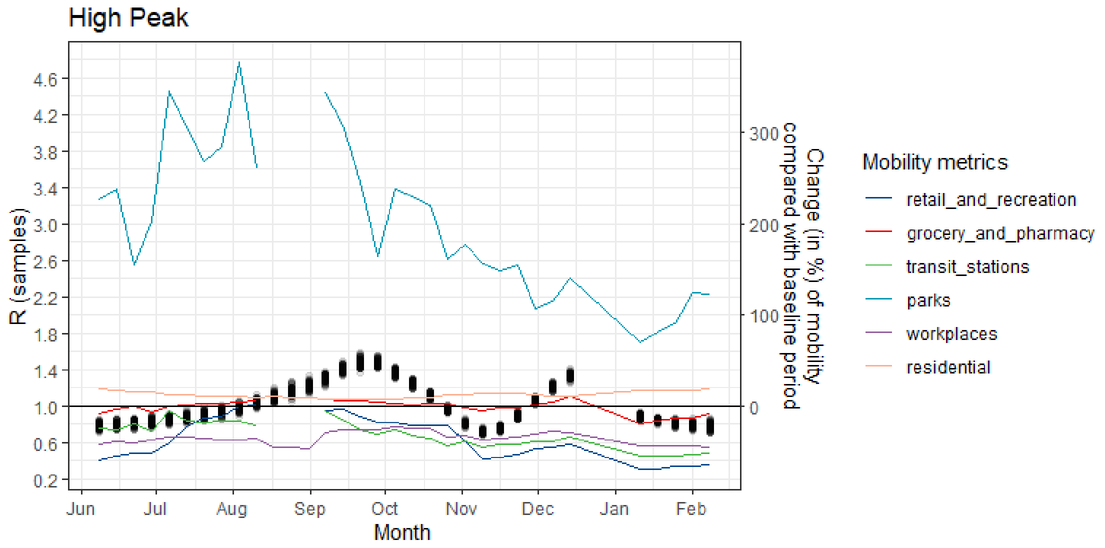
High Peak
R (samples)
4.6
4.2
3.8
3.4
3.0
2.6
2.2
1.8
1.4
1.0
0.6
0.2
300
200
100
0
Change (in %) of mobility compared with baseline period
Jun
Jul
Aug
Sep
Oct
Nov
Dec
Jan
Feb
Month
Mobility metrics
retail_and_recreation
grocery_and_pharmacy
transit_stations
parks
workplaces
residential

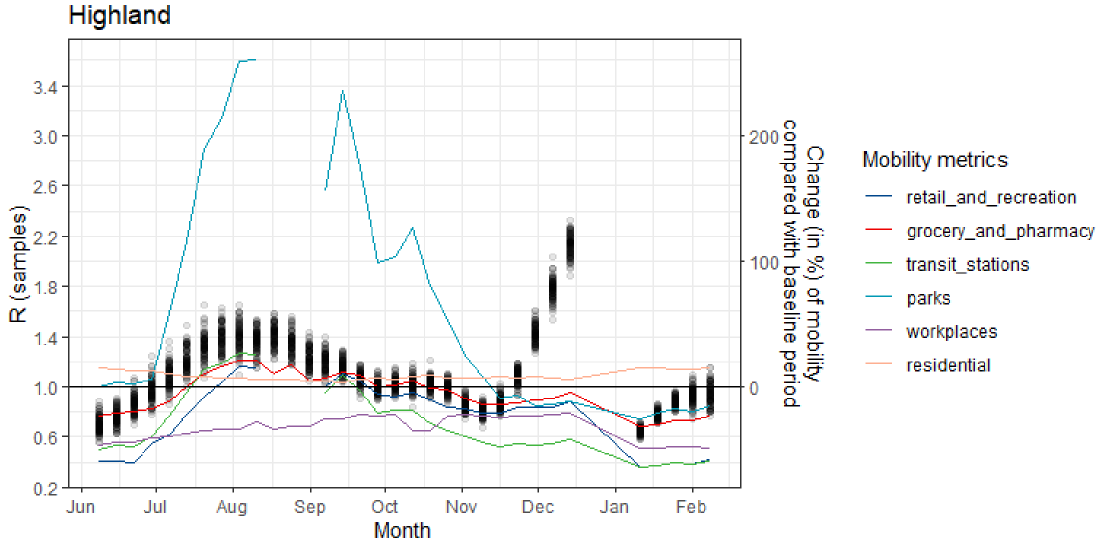
Highland
R (samples)
3.4
3.0
2.6
2.2
1.8
1.4
1.0
0.6
0.2
200
100
0
Change (in %) of mobility compared with baseline period
Jun
Jul
Aug
Sep
Oct
Nov
Dec
Jan
Feb
Month
Mobility metrics
retail_and_recreation
grocery_and_pharmacy
transit_stations
parks
workplaces
residential

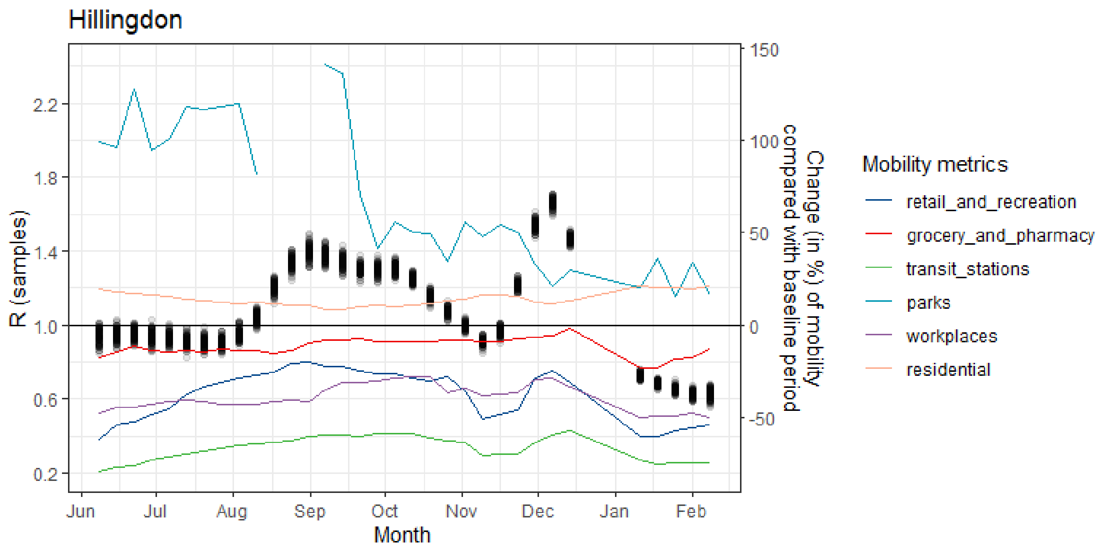
Hillingdon
R (samples)
2.2
1.8
1.4
1.0
0.6
0.2
Jun
Jul
Aug
Sep
Oct
Nov
Dec
Jan
Feb
Month
150
100
50
0
-50
Change (in %) of mobility compared with baseline period
Mobility metrics
retail_and_recreation
grocery_and_pharmacy
transit_stations
parks
workplaces
residential

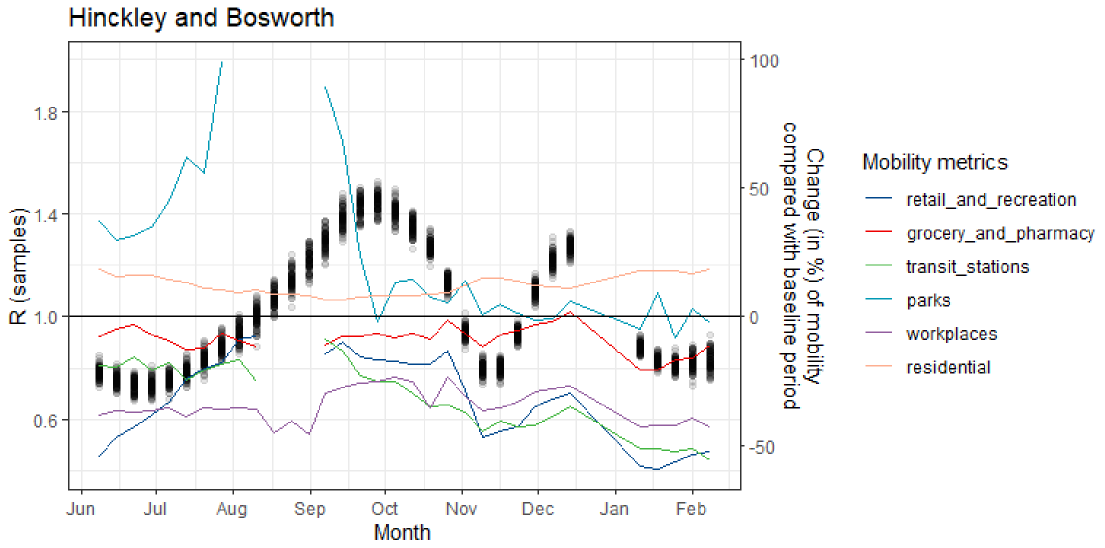
Hinckley and Bosworth
R (samples)
1.8
1.4
1.0
0.6
Jun
Jul
Aug
Sep
Oct
Nov
Dec
Jan
Feb
Month
100
50
0
-50
Change (in %) of mobility compared with baseline period
Mobility metrics
retail_and_recreation
grocery_and_pharmacy
transit_stations
parks
workplaces
residential

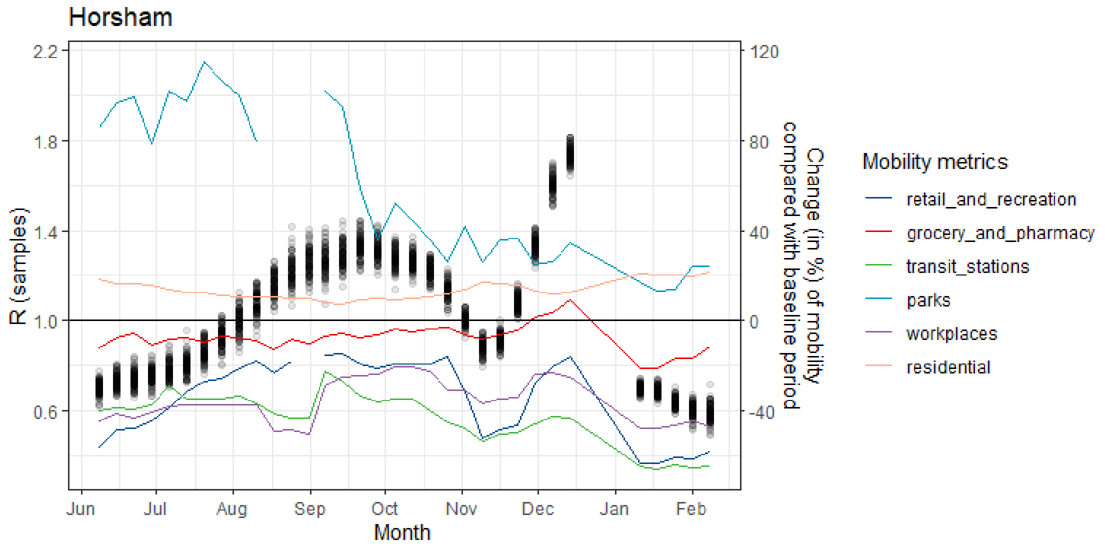
Horsham
R (samples)
2.2
1.8
1.4
1.0
0.6
Jun
Jul
Aug
Sep
Oct
Nov
Dec
Jan
Feb
Month
120
80
40
0
-40
Change (in %) of mobility compared with baseline period
Mobility metrics
retail_and_recreation
grocery_and_pharmacy
transit_stations
parks
workplaces
residential

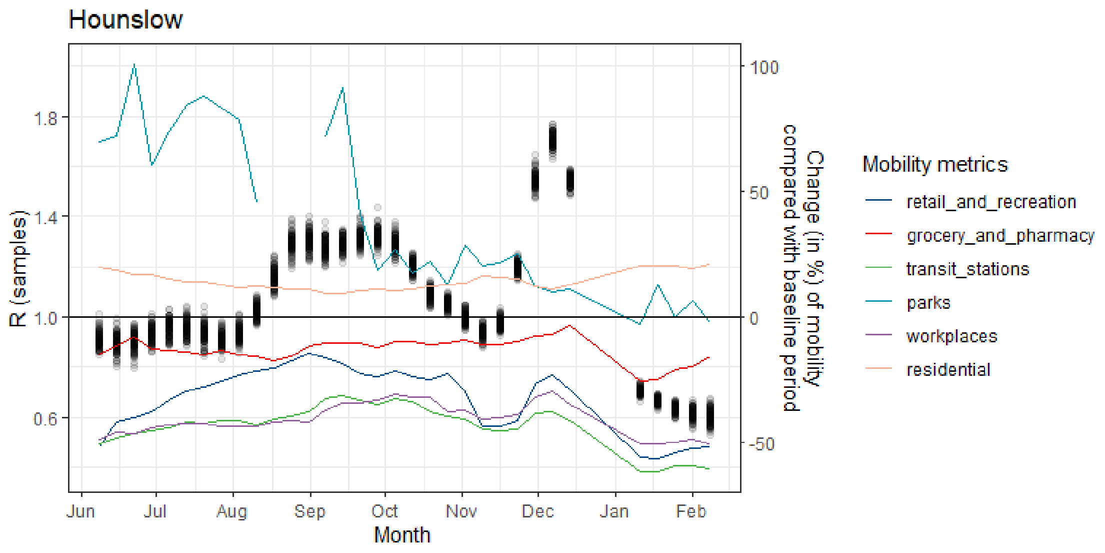
Hounslow
R (samples)
1.8
1.4
1.0
0.6
100
50
0
-50
Change (in %) of mobility compared with baseline period
Jun
Jul
Aug
Sep
Oct
Nov
Dec
Jan
Feb
Month
Mobility metrics
retail_and_recreation
grocery_and_pharmacy
transit_stations
parks
workplaces
residential

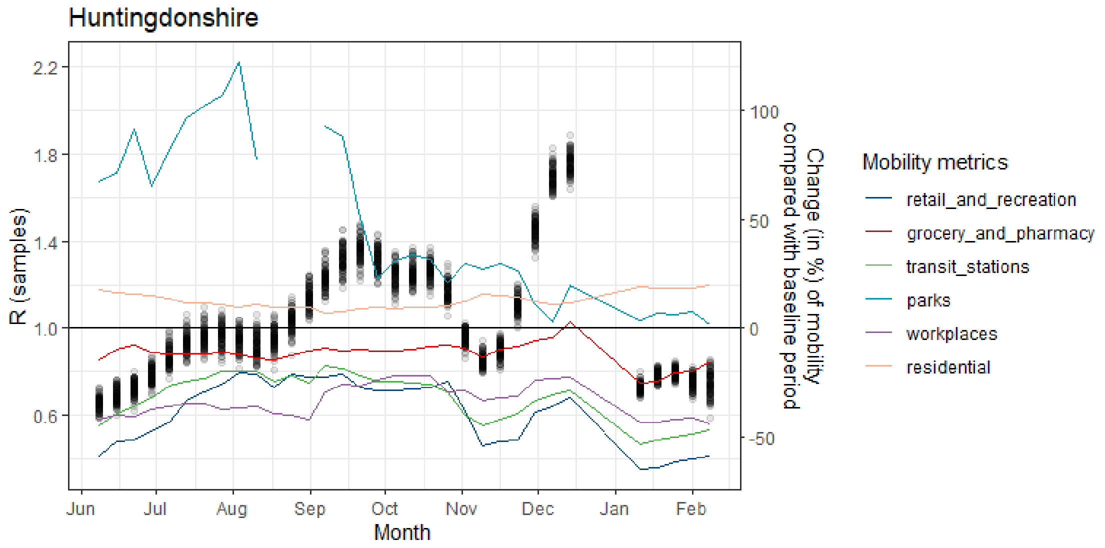
Huntingdonshire
R (samples)
2.2
1.8
1.4
1.0
0.6
100
50
0
-50
Change (in %) of mobility compared with baseline period
Jun
Jul
Aug
Sep
Oct
Nov
Dec
Jan
Feb
Month
Mobility metrics
retail_and_recreation
grocery_and_pharmacy
transit_stations
parks
workplaces
residential

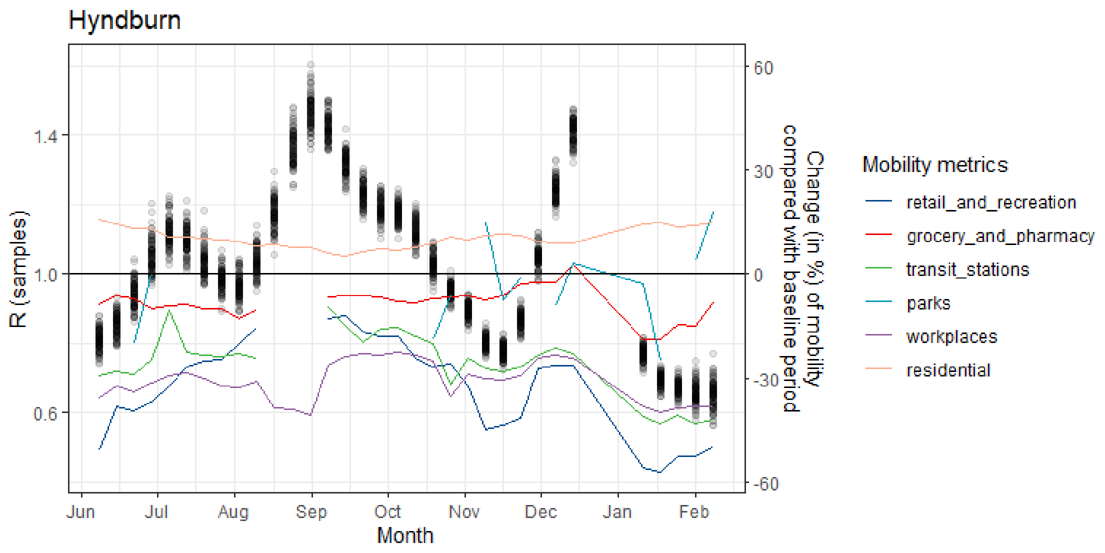
Hyndburn
R (samples)
1.4
1.0
0.6
60
30
0
-30
-60
Change (in %) of mobility compared with baseline period
Jun
Jul
Aug
Sep
Oct
Nov
Dec
Jan
Feb
Month
Mobility metrics
retail_and_recreation
grocery_and_pharmacy
transit_stations
parks
workplaces
residential

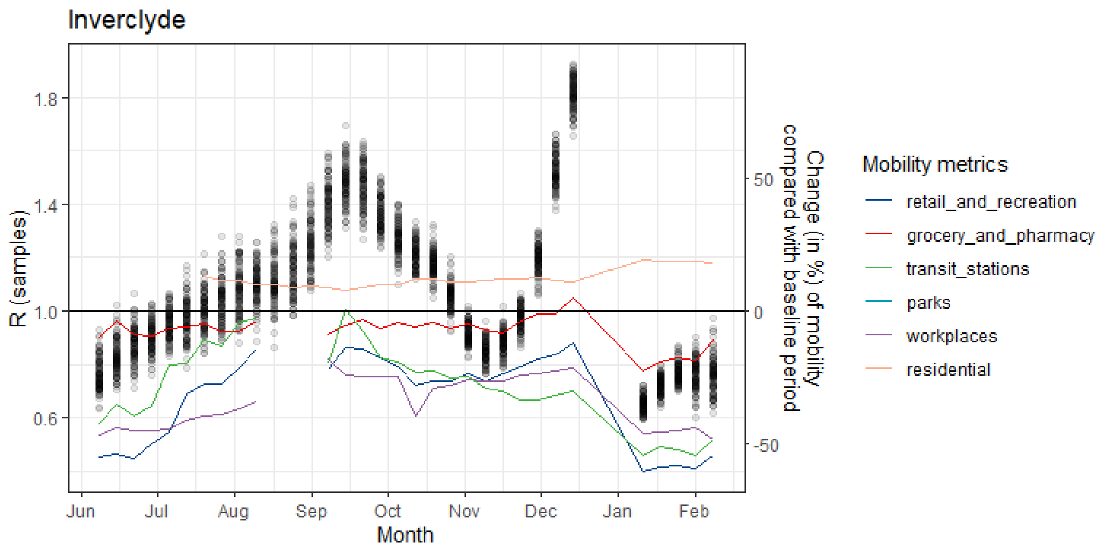
Inverclyde
R (samples)
1.8
1.4
1.0
0.6
Jun
Jul
Aug
Sep
Oct
Nov
Dec
Jan
Feb
Month
50
0
-50
Change (in %) of mobility compared with baseline period
Mobility metrics
retail_and_recreation
grocery_and_pharmacy
transit_stations
parks
workplaces
residential

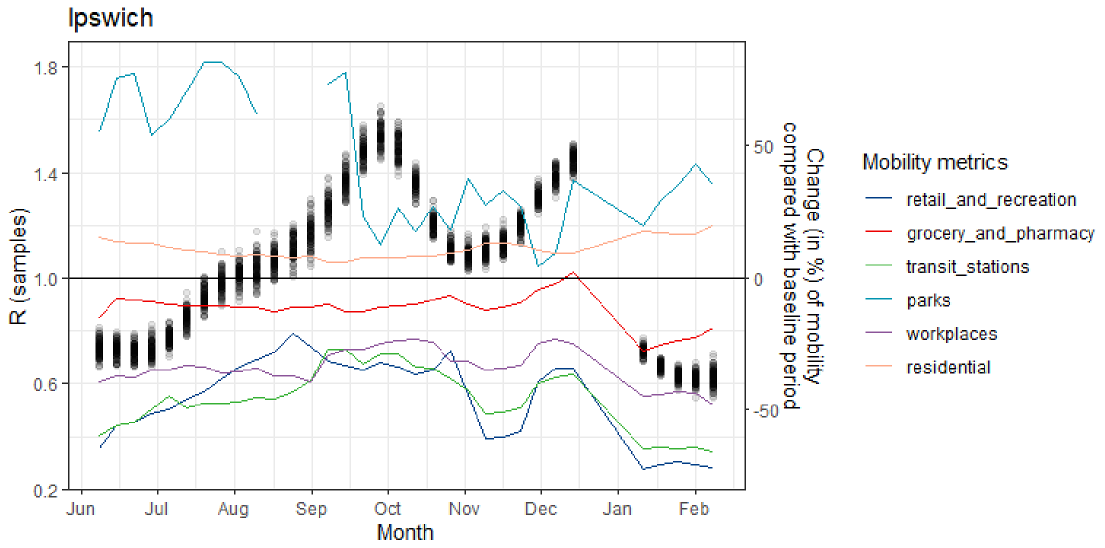
Ipswich
R (samples)
1.8
1.4
1.0
0.6
0.2
Jun
Jul
Aug
Sep
Oct
Nov
Dec
Jan
Feb
Month
50
0
-50
Change (in %) of mobility compared with baseline period
Mobility metrics
retail_and_recreation
grocery_and_pharmacy
transit_stations
parks
workplaces
residential

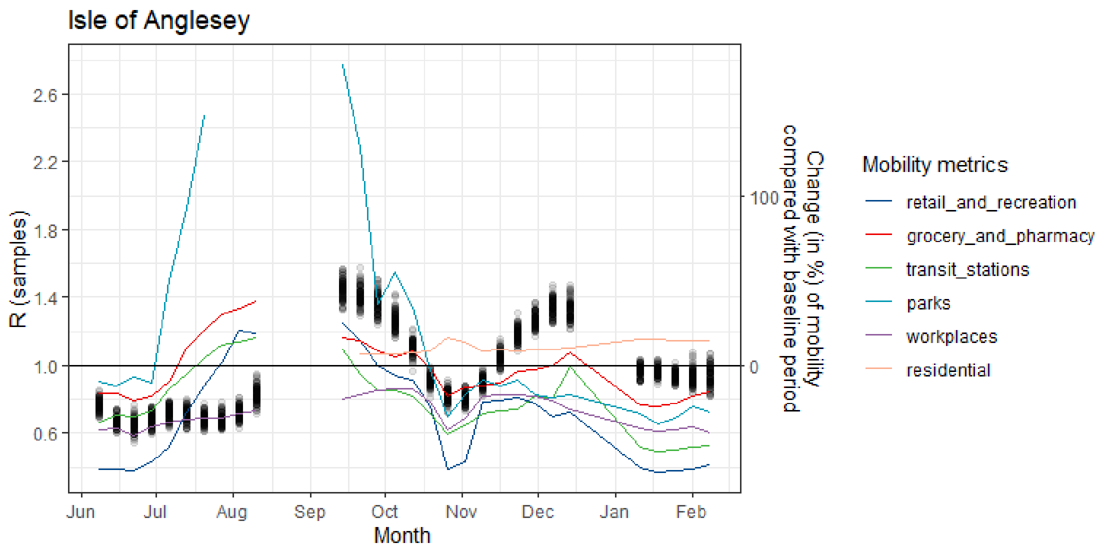
Isle of Anglesey
R (samples)
2.6
2.2
1.8
1.4
1.0
0.6
Jun
Jul
Aug
Sep
Oct
Nov
Dec
Jan
Feb
Month
100
0
Change (in %) of mobility compared with baseline period
Mobility metrics
retail_and_recreation
grocery_and_pharmacy
transit_stations
parks
workplaces
residential

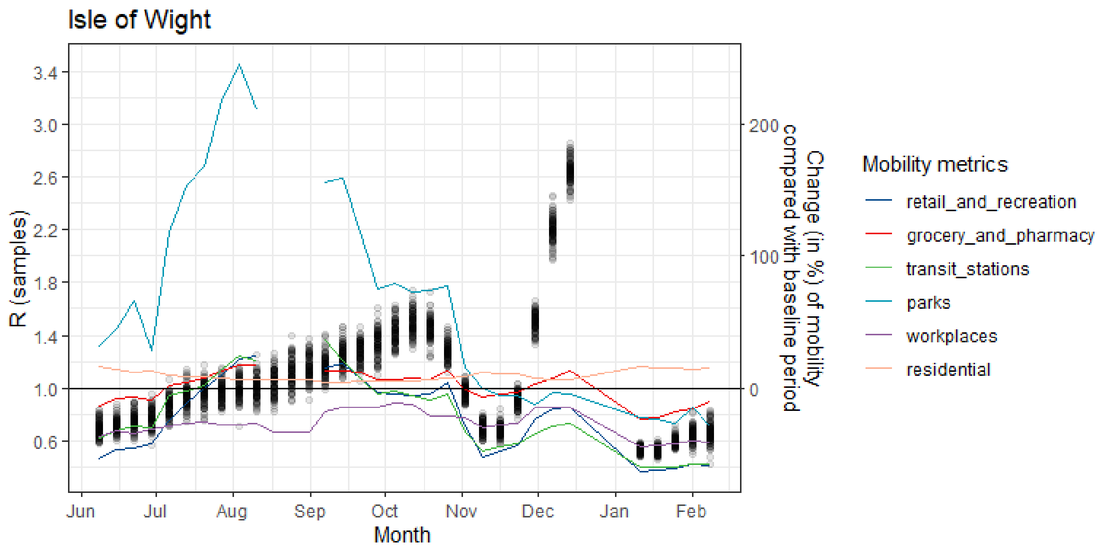
Isle of Wight
R (samples)
3.4
3.0
2.6
2.2
1.8
1.4
1.0
0.6
Jun Jul Aug Sep Oct Nov Dec Jan Feb
Month
200
100
0
Change (in %) of mobility compared with baseline period
Mobility metrics
retail_and_recreation
grocery_and_pharmacy
transit_stations
parks
workplaces
residential

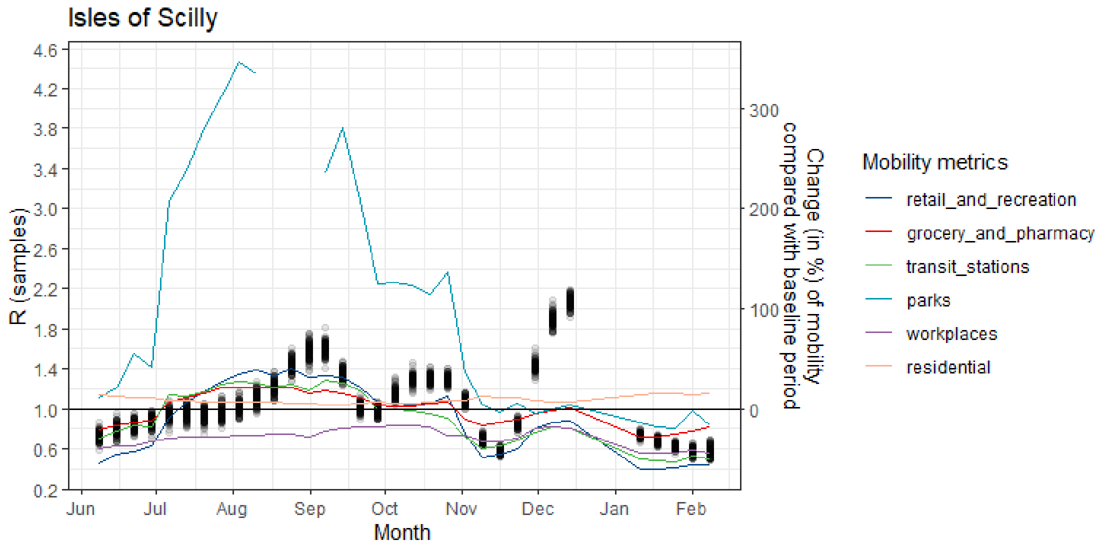
Isles of Scilly
R (samples)
4.6
4.2
3.8
3.4
3.0
2.6
2.2
1.8
1.4
1.0
0.6
0.2
Jun Jul Aug Sep Oct Nov Dec Jan Feb
Month
300
200
100
0
Change (in %) of mobility compared with baseline period
Mobility metrics
retail_and_recreation
grocery_and_pharmacy
transit_stations
parks
workplaces
residential

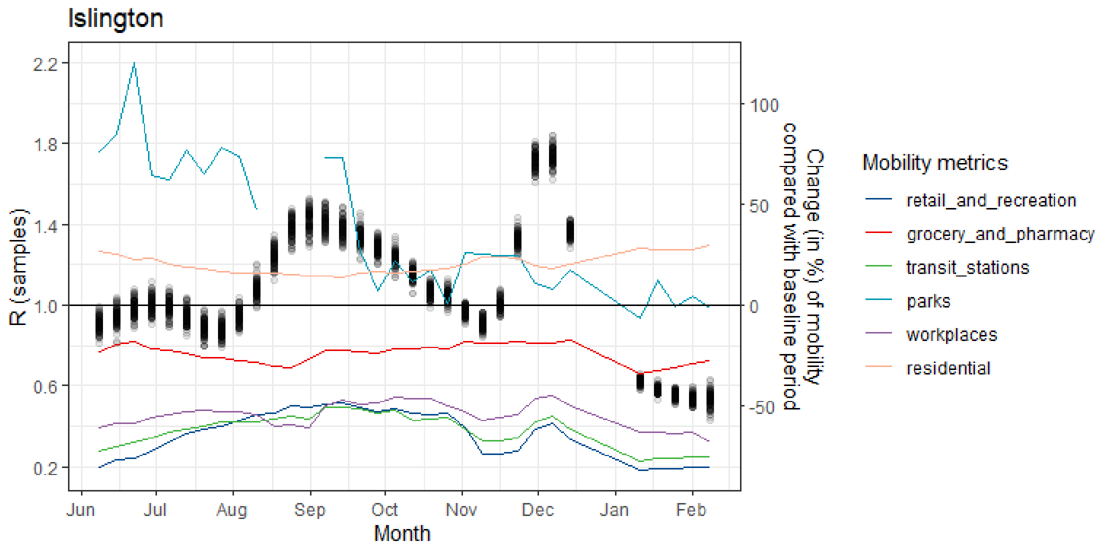
Islington
R (samples)
2.2
1.8
1.4
1.0
0.6
0.2
Jun Jul Aug Sep Oct Nov Dec Jan Feb
Month
100
50
0
-50
Change (in %) of mobility compared with baseline period
Mobility metrics
retail_and_recreation
grocery_and_pharmacy
transit_stations
parks
workplaces
residential

Kensington and Chelsea

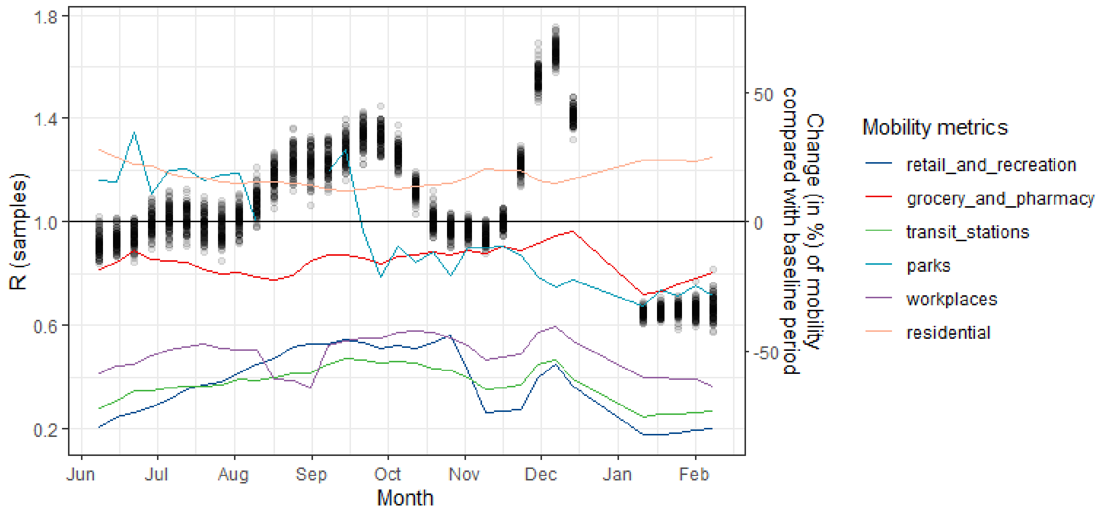

Kettering

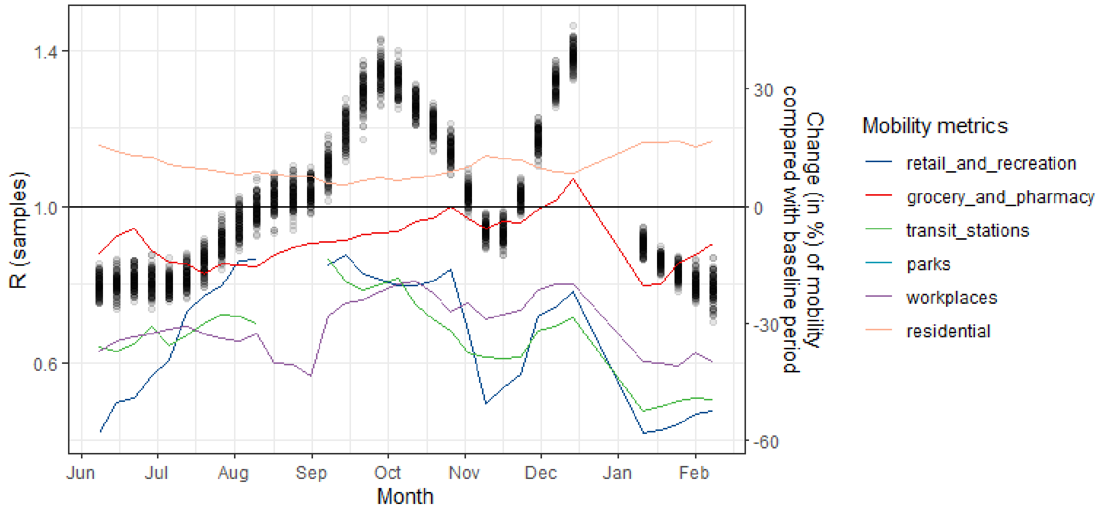

King's Lynn and West Norfolk

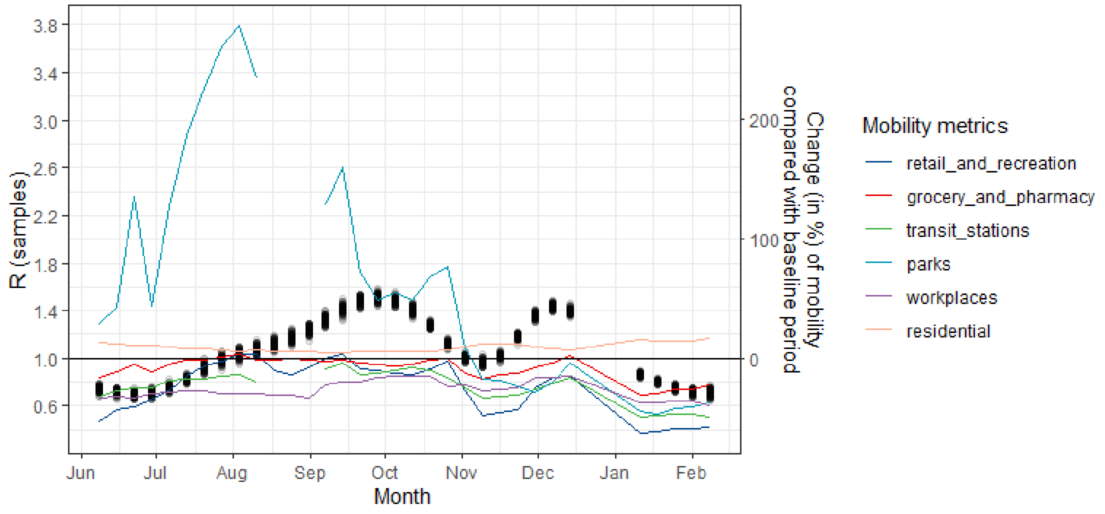

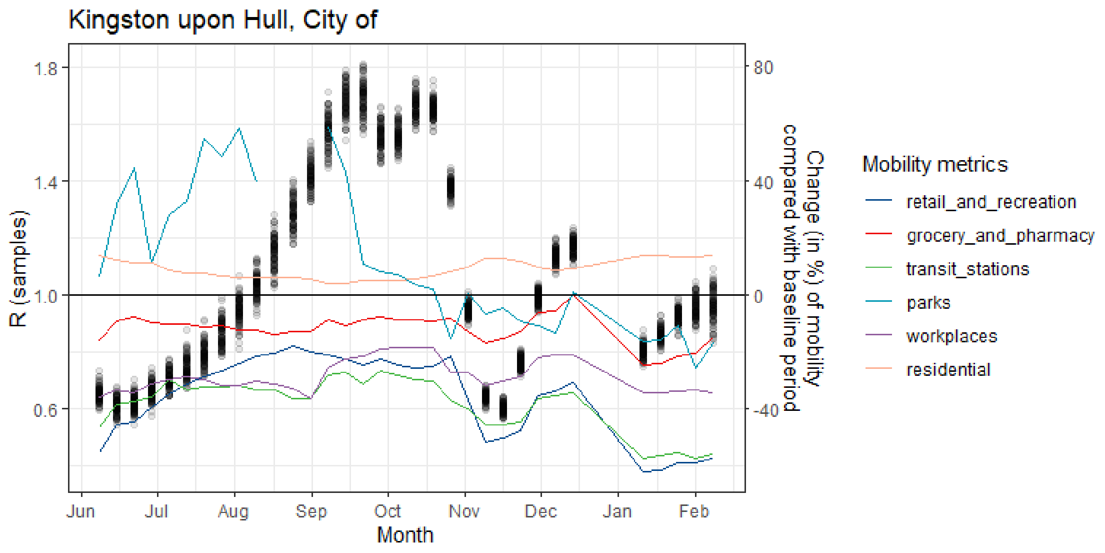
Kingston upon Hull, City of
R (samples)
1.8
1.4
1.0
0.6
80
40
0
-40
Change (in %) of mobility compared with baseline period
Jun Jul Aug Sep Oct Nov Dec Jan Feb
Month
Mobility metrics
retail_and_recreation
grocery_and_pharmacy
transit_stations
parks
workplaces
residential

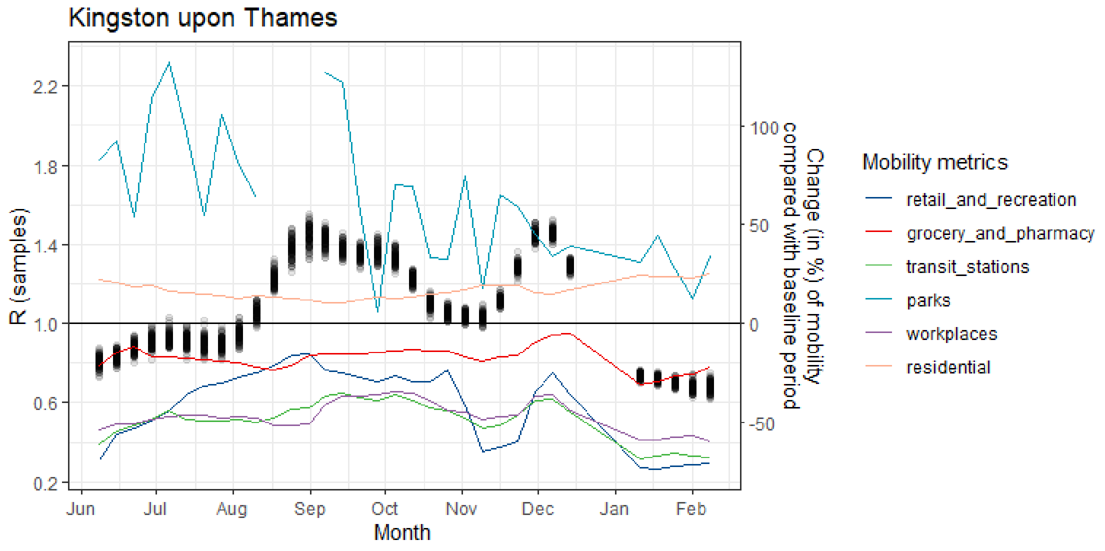
Kingston upon Thames
R (samples)
2.2
1.8
1.4
1.0
0.6
0.2
100
50
0
-50
Change (in %) of mobility compared with baseline period
Jun Jul Aug Sep Oct Nov Dec Jan Feb
Month
Mobility metrics
retail_and_recreation
grocery_and_pharmacy
transit_stations
parks
workplaces
residential

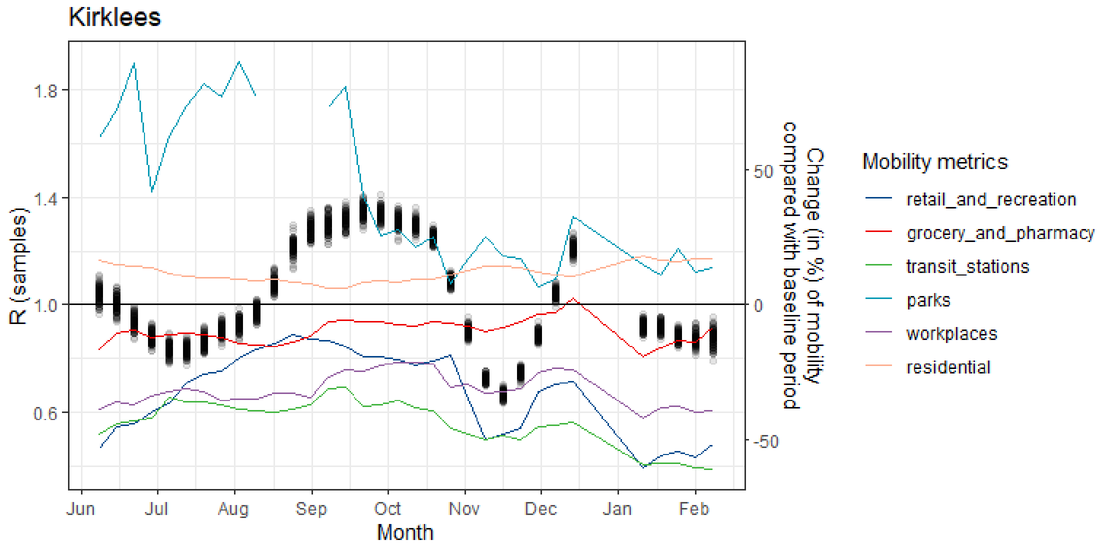
Kirklees
R (samples)
1.8
1.4
1.0
0.6
50
0
-50
Change (in %) of mobility compared with baseline period
Jun Jul Aug Sep Oct Nov Dec Jan Feb
Month
Mobility metrics
retail_and_recreation
grocery_and_pharmacy
transit_stations
parks
workplaces
residential

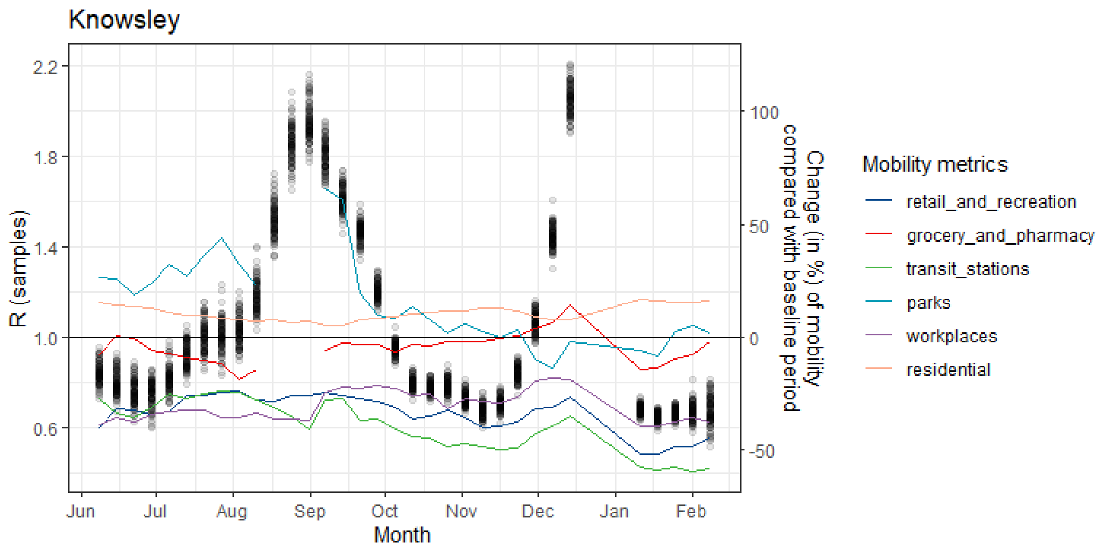
Knowsley
R (samples)
2.2
1.8
1.4
1.0
0.6
Jun
Jul
Aug
Sep
Oct
Nov
Dec
Jan
Feb
Month
100
50
0
-50
Change (in %) of mobility compared with baseline period
Mobility metrics
retail_and_recreation
grocery_and_pharmacy
transit_stations
parks
workplaces
residential

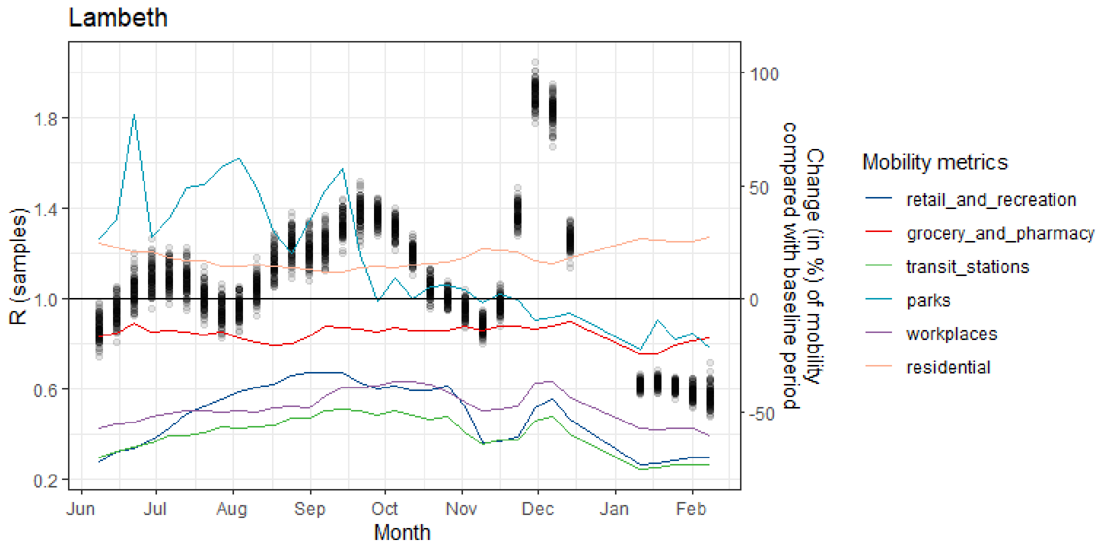
Lambeth
R (samples)
1.8
1.4
1.0
0.6
0.2
Jun
Jul
Aug
Sep
Oct
Nov
Dec
Jan
Feb
Month
100
50
0
-50
Change (in %) of mobility compared with baseline period
Mobility metrics
retail_and_recreation
grocery_and_pharmacy
transit_stations
parks
workplaces
residential

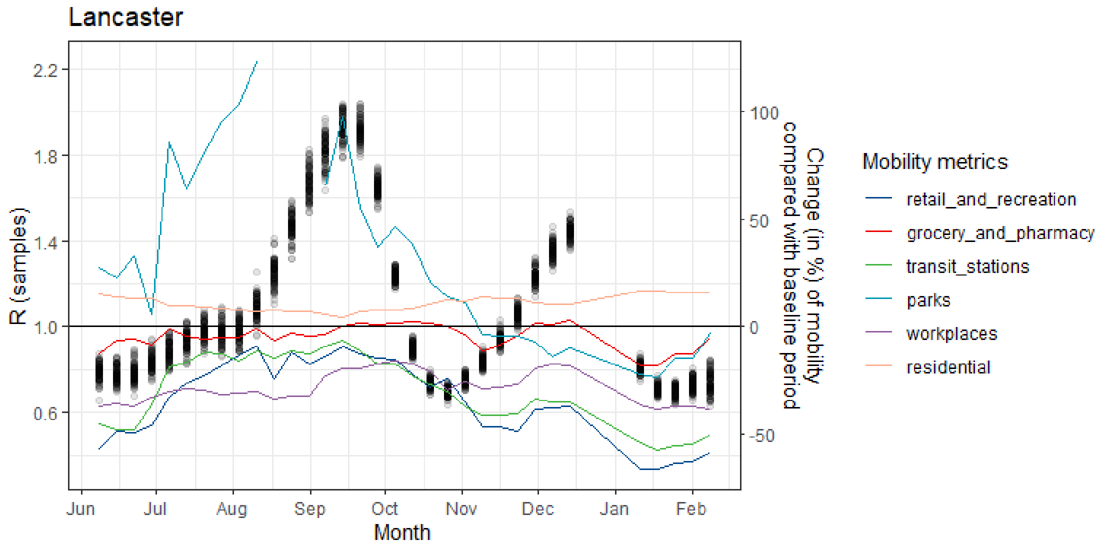
Lancaster
R (samples)
2.2
1.8
1.4
1.0
0.6
Jun
Jul
Aug
Sep
Oct
Nov
Dec
Jan
Feb
Month
100
50
0
-50
Change (in %) of mobility compared with baseline period
Mobility metrics
retail_and_recreation
grocery_and_pharmacy
transit_stations
parks
workplaces
residential

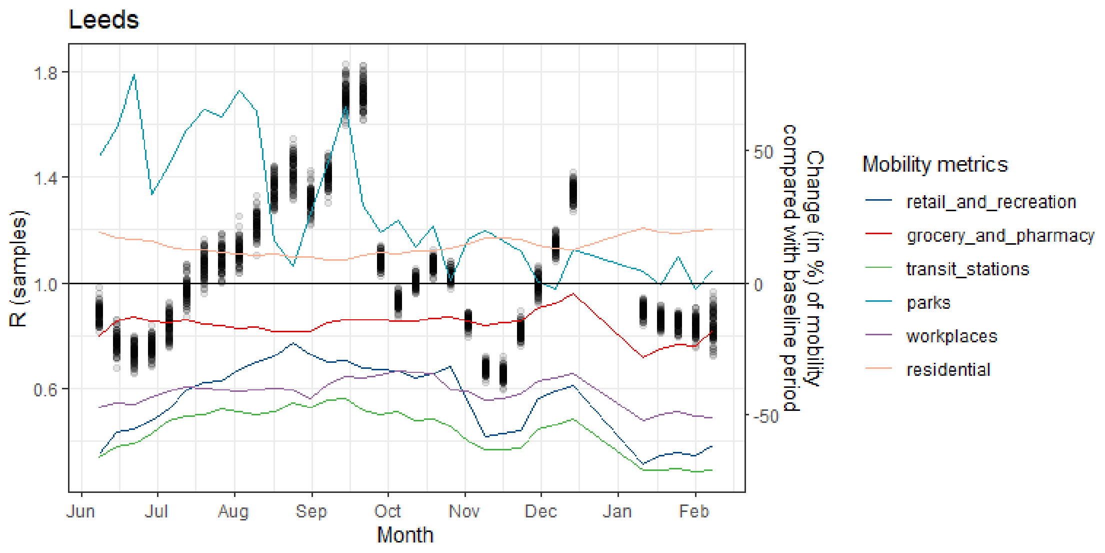

Leeds
R (samples)
1.8
1.4
1.0
0.6
Jun
Jul
Aug
Sep
Oct
Nov
Dec
Jan
Feb
Month
50
0
-50
Change (in %) of mobility compared with baseline period
Mobility metrics
retail_and_recreation
grocery_and_pharmacy
transit_stations
parks
workplaces
residential

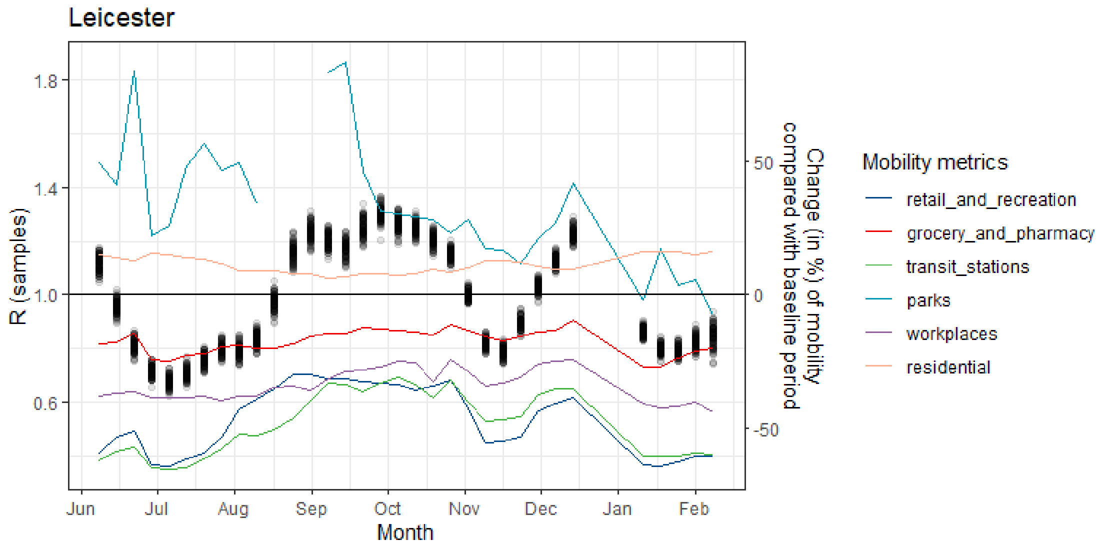

Leicester
R (samples)
1.8
1.4
1.0
0.6
Jun
Jul
Aug
Sep
Oct
Nov
Dec
Jan
Feb
Month
50
0
-50
Change (in %) of mobility compared with baseline period
Mobility metrics
retail_and_recreation
grocery_and_pharmacy
transit_stations
parks
workplaces
residential

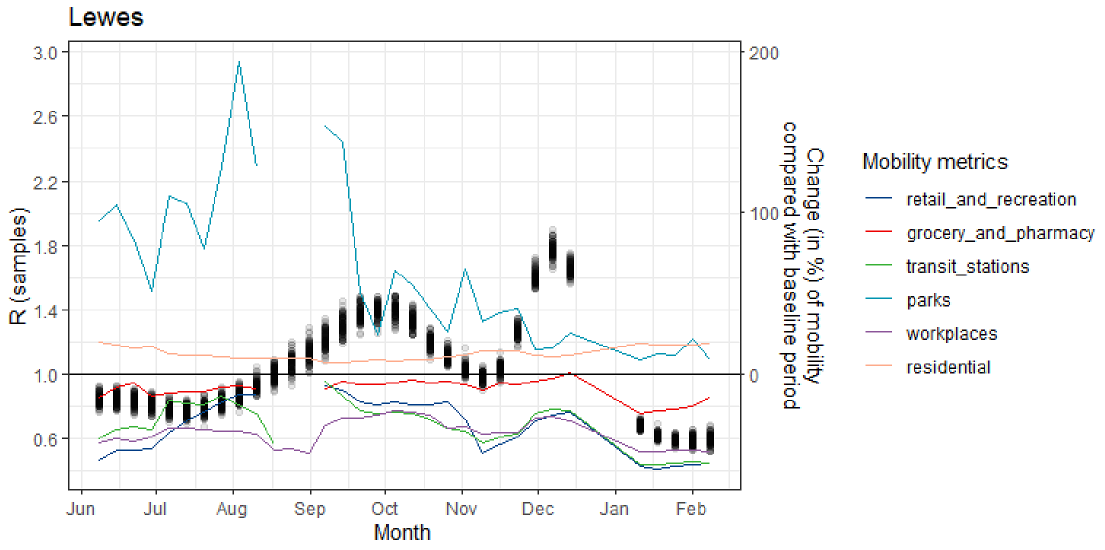

Lewes
R (samples)
3.0
2.6
2.2
1.8
1.4
1.0
0.6
Jun
Jul
Aug
Sep
Oct
Nov
Dec
Jan
Feb
Month
200
100
0
Change (in %) of mobility compared with baseline period
Mobility metrics
retail_and_recreation
grocery_and_pharmacy
transit_stations
parks
workplaces
residential

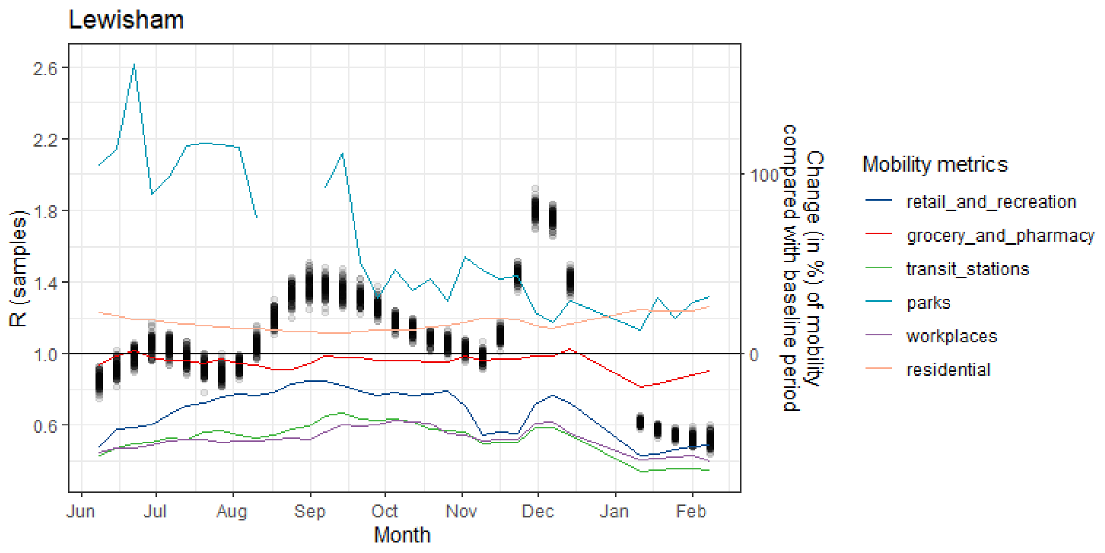
Lewisham
R (samples)
2.6
2.2
1.8
1.4
1.0
0.6
Jun
Jul
Aug
Sep
Oct
Nov
Dec
Jan
Feb
Month
100
0
Change (in %) of mobility compared with baseline period
Mobility metrics
retail_and_recreation
grocery_and_pharmacy
transit_stations
parks
workplaces
residential

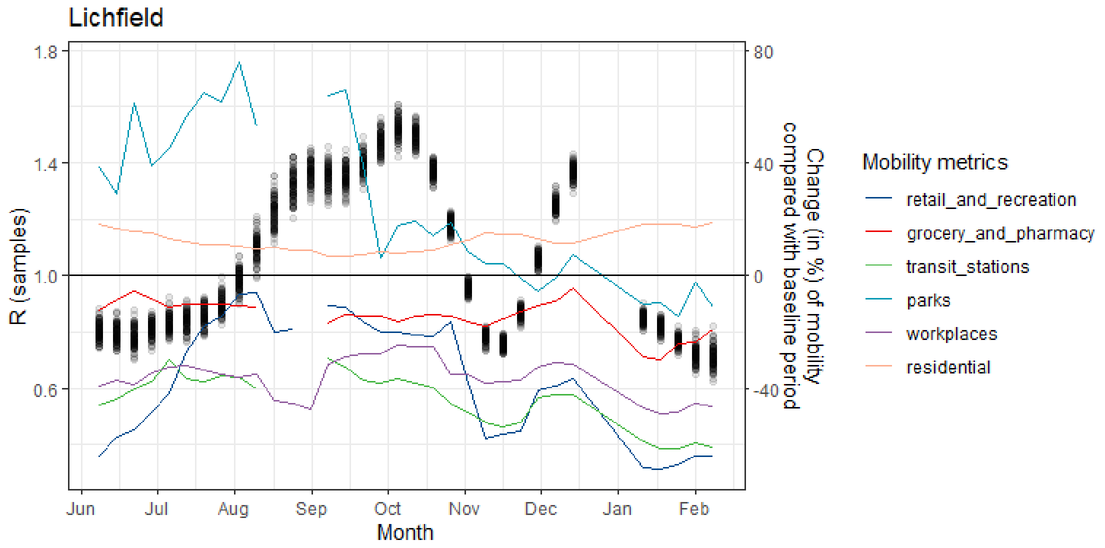
Lichfield
R (samples)
1.8
1.4
1.0
0.6
Jun
Jul
Aug
Sep
Oct
Nov
Dec
Jan
Feb
Month
80
40
0
-40
Change (in %) of mobility compared with baseline period
Mobility metrics
retail_and_recreation
grocery_and_pharmacy
transit_stations
parks
workplaces
residential

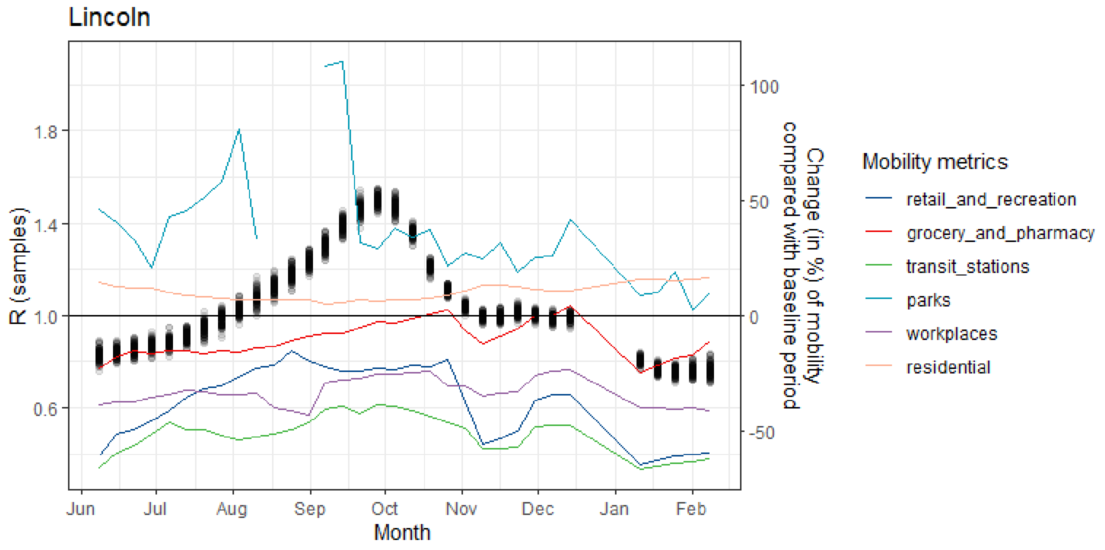
Lincoln
R (samples)
1.8
1.4
1.0
0.6
Jun
Jul
Aug
Sep
Oct
Nov
Dec
Jan
Feb
Month
100
50
0
-50
Change (in %) of mobility compared with baseline period
Mobility metrics
retail_and_recreation
grocery_and_pharmacy
transit_stations
parks
workplaces
residential

Lisburn and Castlereagh

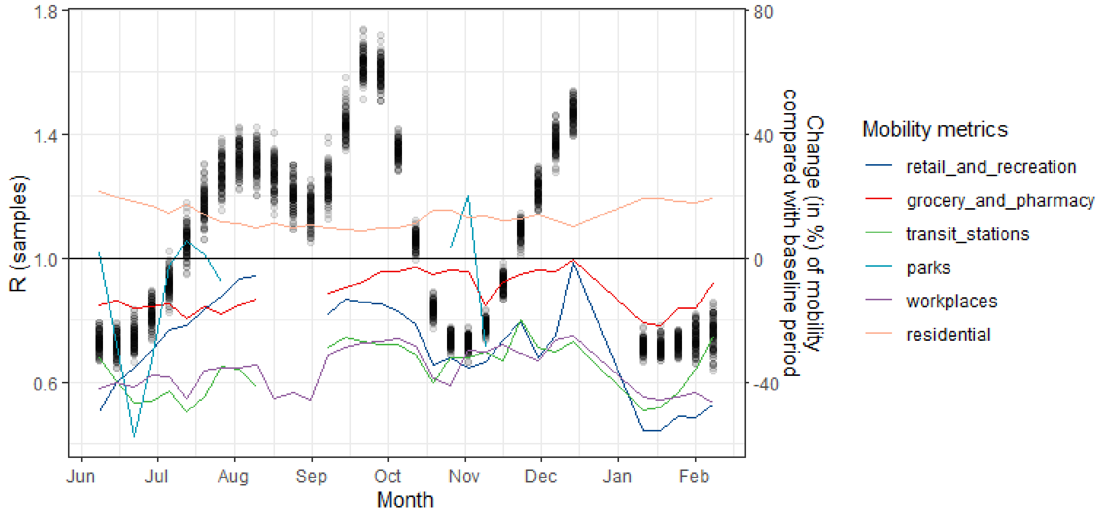

Liverpool

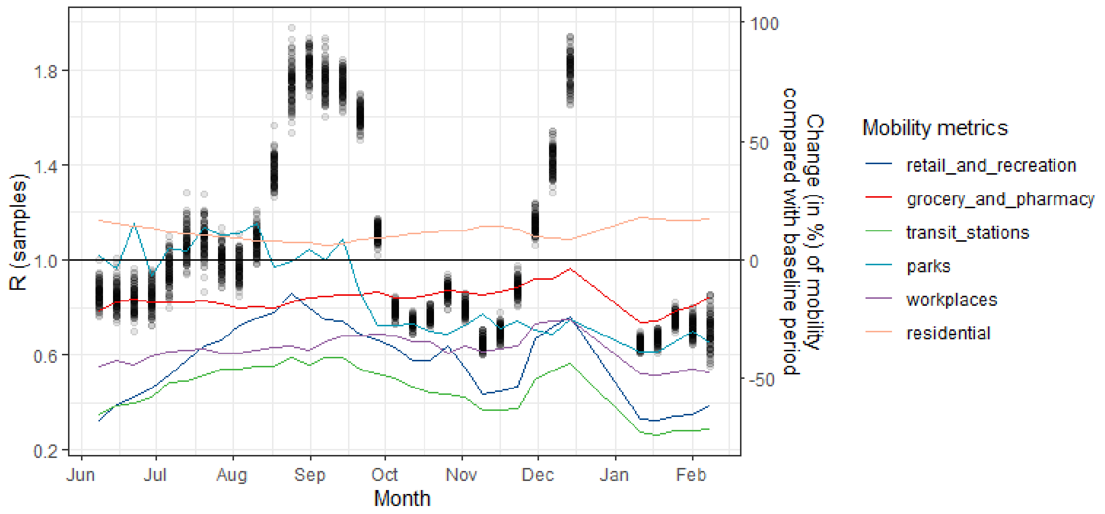

Luton

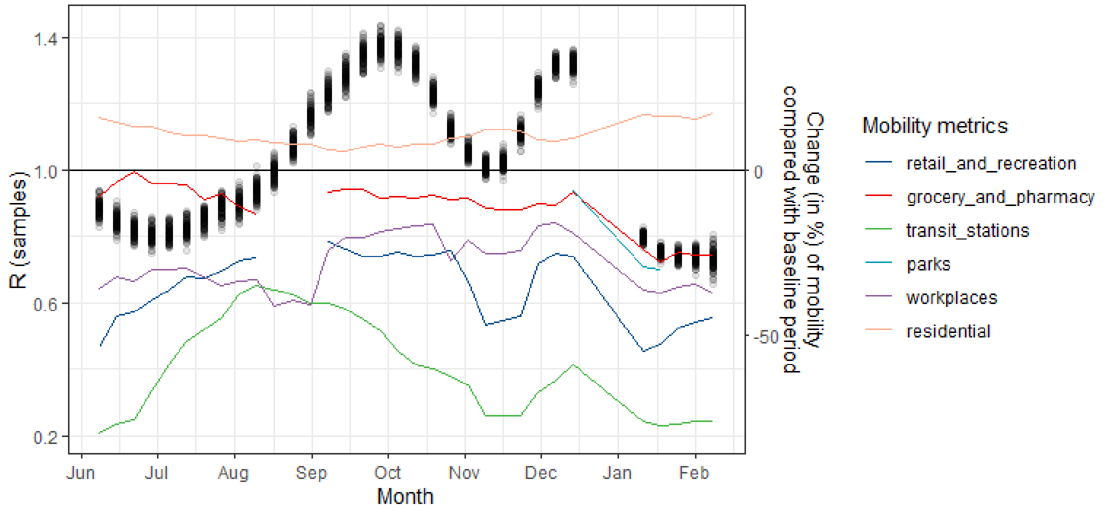

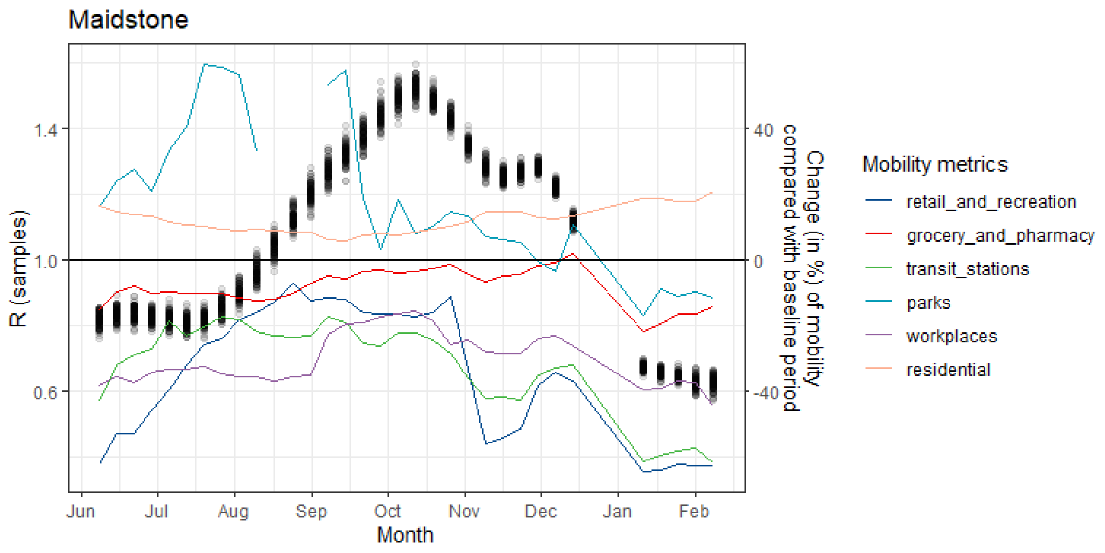
Maidstone
R (samples)
1.4
1.0
0.6
40
0
-40
Change (in %) of mobility compared with baseline period
Jun
Jul
Aug
Sep
Oct
Nov
Dec
Jan
Feb
Month
Mobility metrics
retail_and_recreation
grocery_and_pharmacy
transit_stations
parks
workplaces
residential

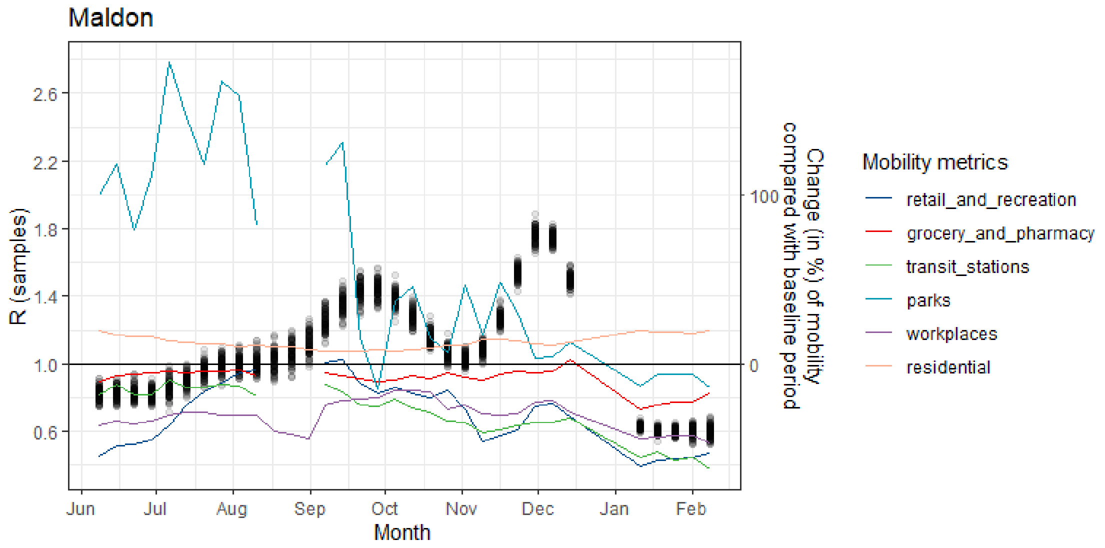
Maldon
R (samples)
2.6
2.2
1.8
1.4
1.0
0.6
100
0
Change (in %) of mobility compared with baseline period
Jun
Jul
Aug
Sep
Oct
Nov
Dec
Jan
Feb
Month
Mobility metrics
retail_and_recreation
grocery_and_pharmacy
transit_stations
parks
workplaces
residential

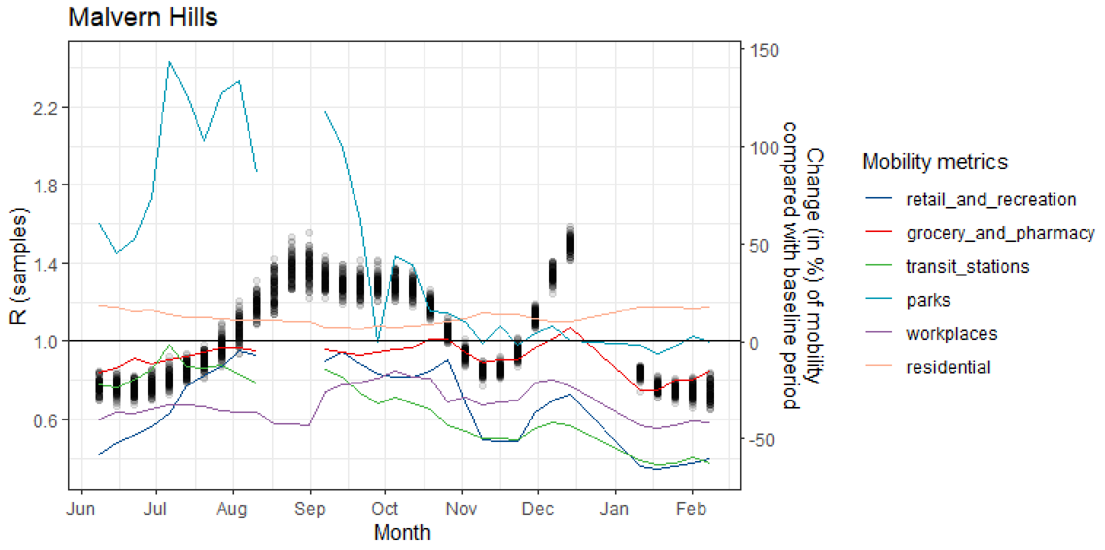
Malvern Hills
R (samples)
2.2
1.8
1.4
1.0
0.6
150
100
50
0
-50
Change (in %) of mobility compared with baseline period
Jun
Jul
Aug
Sep
Oct
Nov
Dec
Jan
Feb
Month
Mobility metrics
retail_and_recreation
grocery_and_pharmacy
transit_stations
parks
workplaces
residential

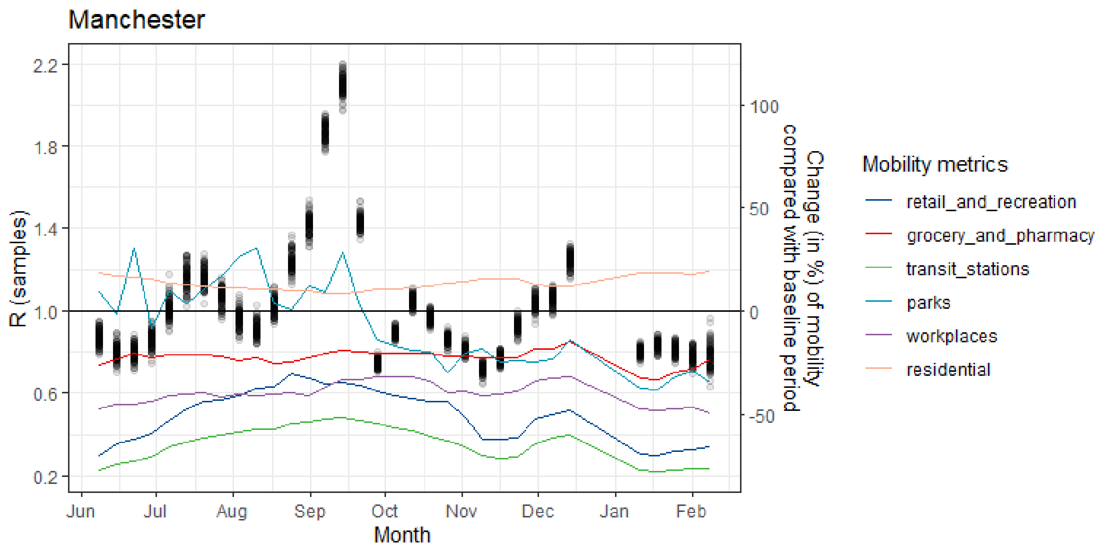
Manchester
R (samples)
2.2
1.8
1.4
1.0
0.6
0.2
100
50
0
-50
Change (in %) of mobility compared with baseline period
Jun
Jul
Aug
Sep
Oct
Nov
Dec
Jan
Feb
Month
Mobility metrics
retail_and_recreation
grocery_and_pharmacy
transit_stations
parks
workplaces
residential

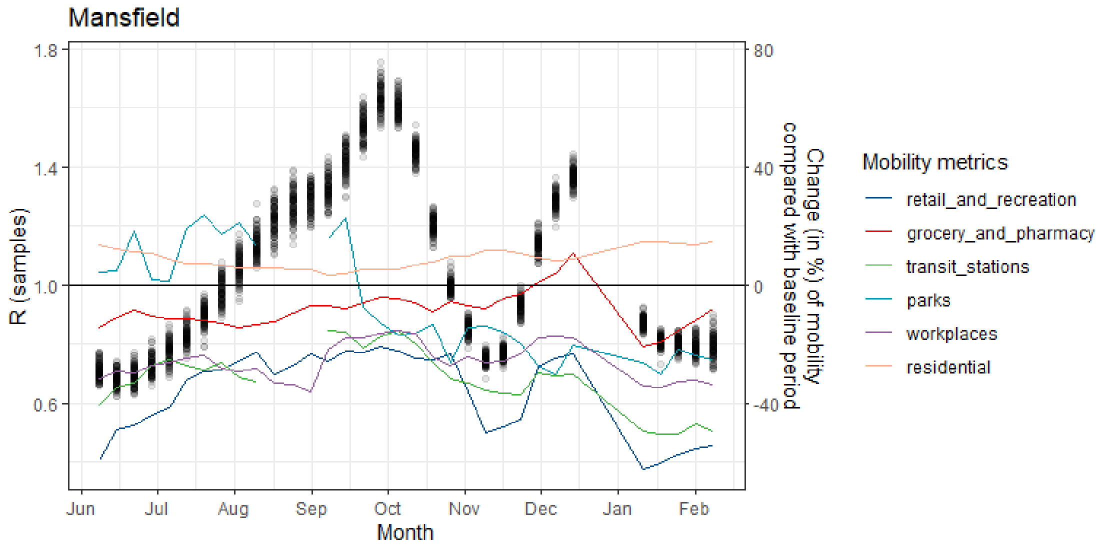
Mansfield
R (samples)
1.8
1.4
1.0
0.6
80
40
0
-40
Change (in %) of mobility compared with baseline period
Jun
Jul
Aug
Sep
Oct
Nov
Dec
Jan
Feb
Month
Mobility metrics
retail_and_recreation
grocery_and_pharmacy
transit_stations
parks
workplaces
residential

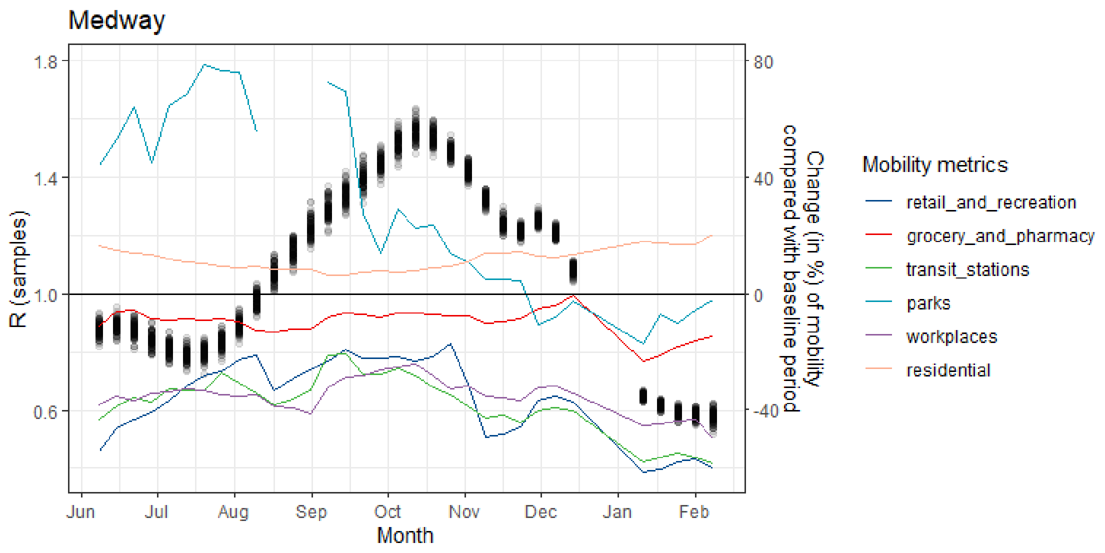
Medway
R (samples)
1.8
1.4
1.0
0.6
80
40
0
-40
Change (in %) of mobility compared with baseline period
Jun
Jul
Aug
Sep
Oct
Nov
Dec
Jan
Feb
Month
Mobility metrics
retail_and_recreation
grocery_and_pharmacy
transit_stations
parks
workplaces
residential

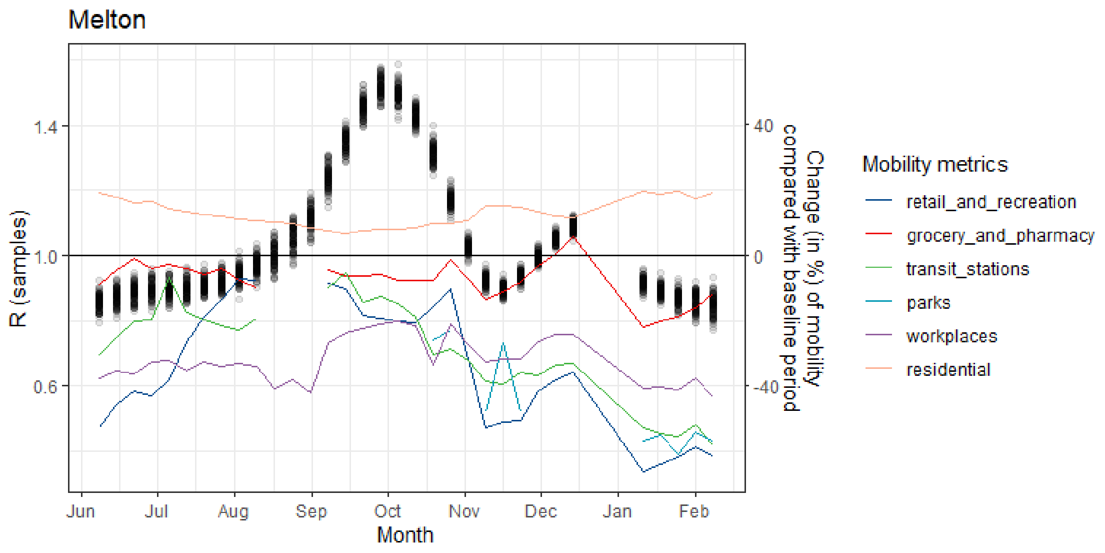
Melton
R (samples)
1.4
1.0
0.6
Jun
Jul
Aug
Sep
Oct
Nov
Dec
Jan
Feb
Month
40
0
-40
Change (in %) of mobility compared with baseline period
Mobility metrics
retail_and_recreation
grocery_and_pharmacy
transit_stations
parks
workplaces
residential

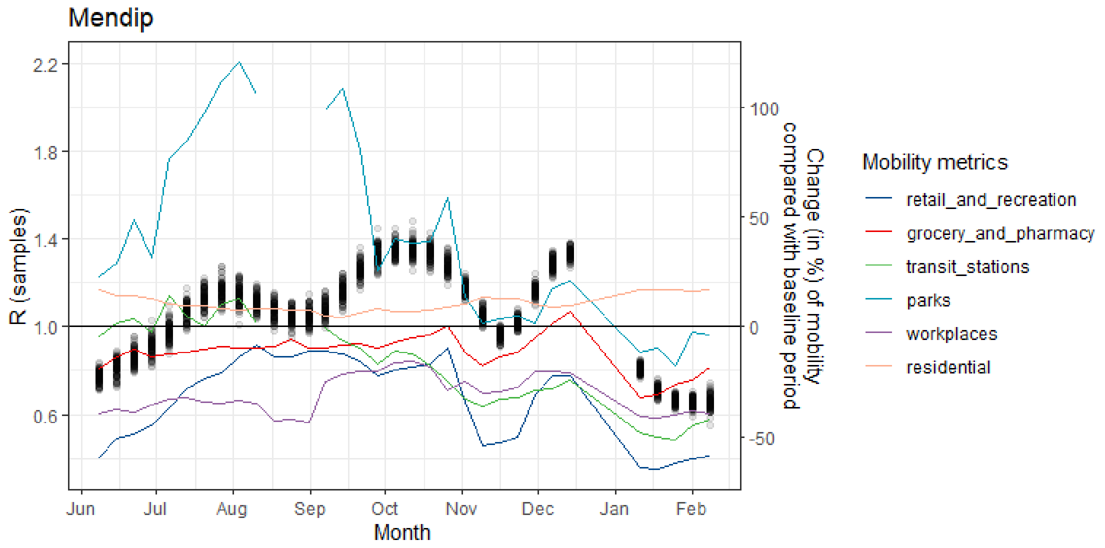
Mendip
R (samples)
2.2
1.8
1.4
1.0
0.6
Jun
Jul
Aug
Sep
Oct
Nov
Dec
Jan
Feb
Month
100
50
0
-50
Change (in %) of mobility compared with baseline period
Mobility metrics
retail_and_recreation
grocery_and_pharmacy
transit_stations
parks
workplaces
residential

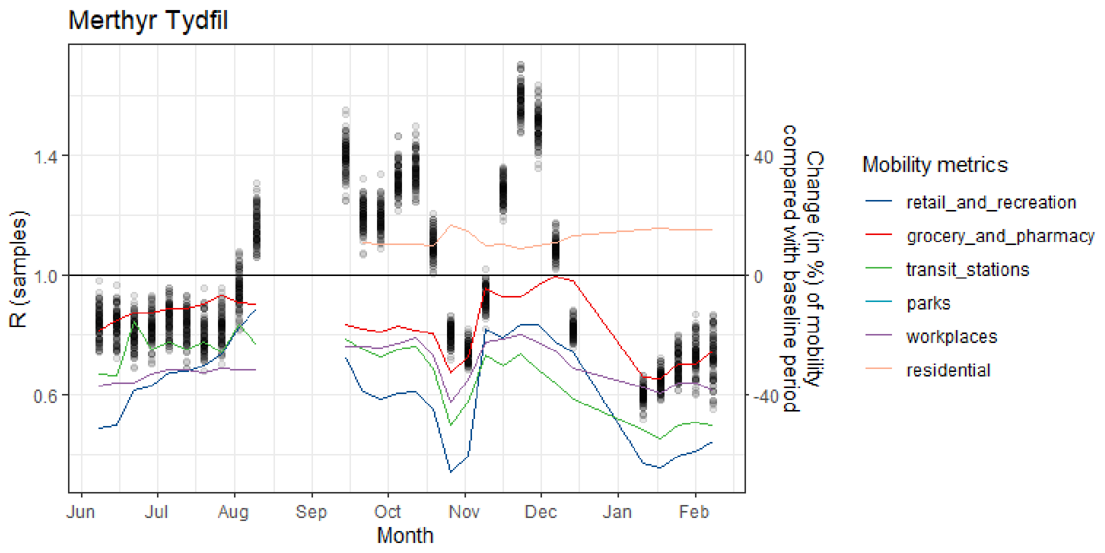
Merthyr Tydfil
R (samples)
1.4
1.0
0.6
Jun
Jul
Aug
Sep
Oct
Nov
Dec
Jan
Feb
Month
40
0
-40
Change (in %) of mobility compared with baseline period
Mobility metrics
retail_and_recreation
grocery_and_pharmacy
transit_stations
parks
workplaces
residential

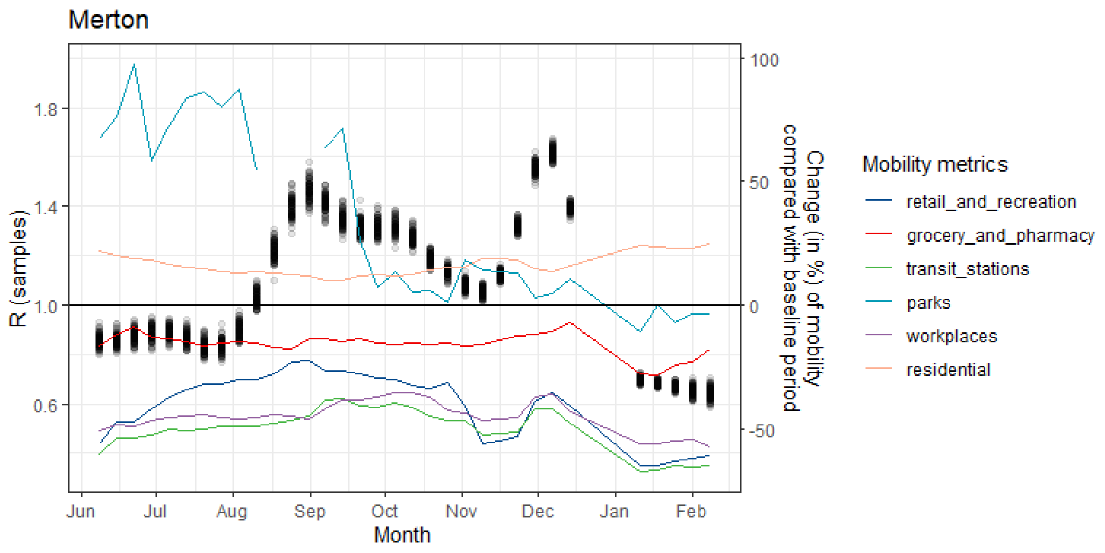
Merton
R (samples)
1.8
1.4
1.0
0.6
Jun
Jul
Aug
Sep
Oct
Nov
Dec
Jan
Feb
Month
100
50
0
-50
Change (in %) of mobility compared with baseline period
Mobility metrics
retail_and_recreation
grocery_and_pharmacy
transit_stations
parks
workplaces
residential

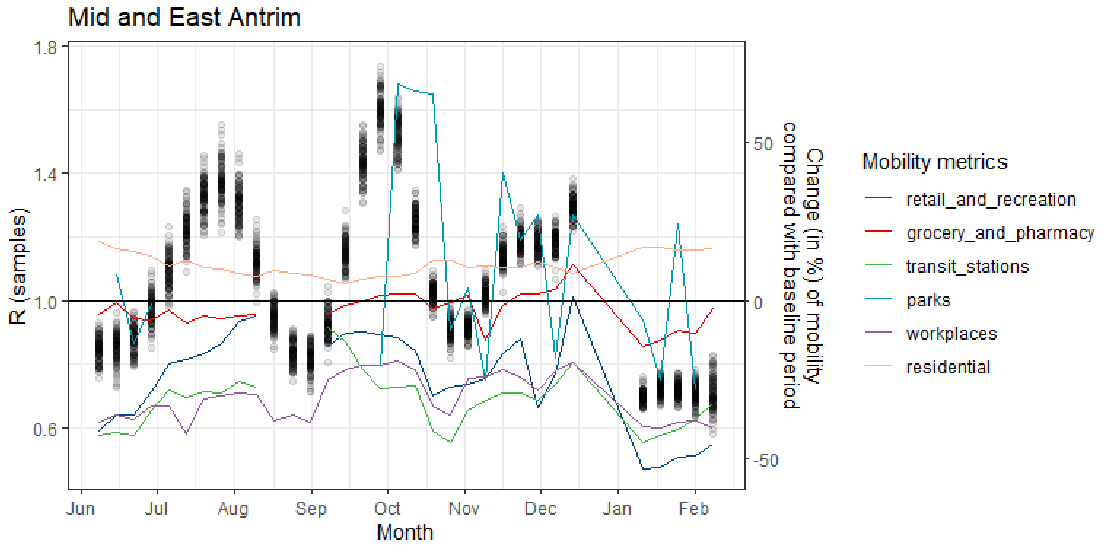
Mid and East Antrim
R (samples)
1.8
1.4
1.0
0.6
Jun
Jul
Aug
Sep
Oct
Nov
Dec
Jan
Feb
Month
50
0
-50
Change (in %) of mobility compared with baseline period
Mobility metrics
retail_and_recreation
grocery_and_pharmacy
transit_stations
parks
workplaces
residential

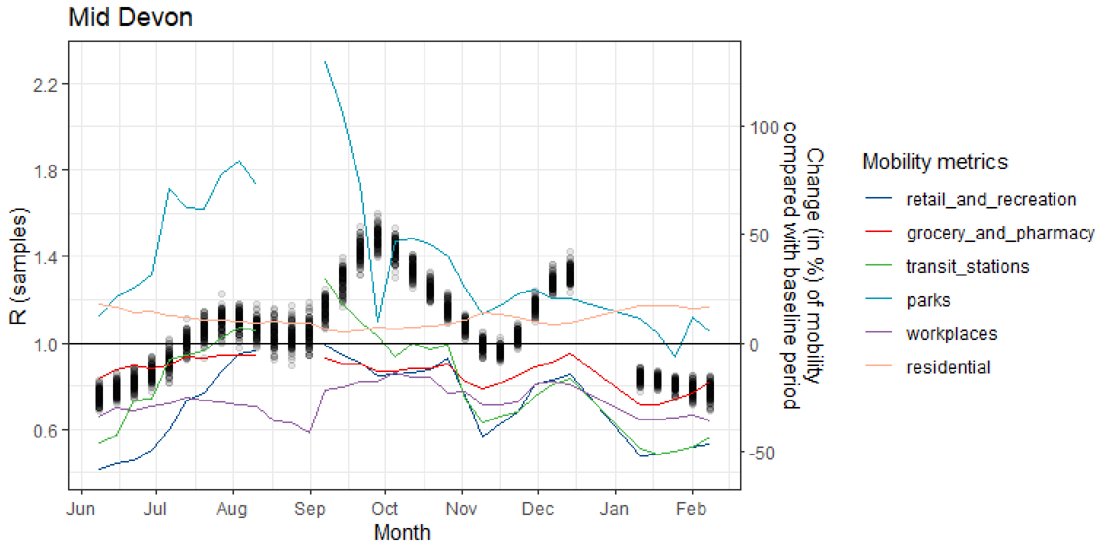
Mid Devon
R (samples)
2.2
1.8
1.4
1.0
0.6
Jun
Jul
Aug
Sep
Oct
Nov
Dec
Jan
Feb
Month
100
50
0
-50
Change (in %) of mobility compared with baseline period
Mobility metrics
retail_and_recreation
grocery_and_pharmacy
transit_stations
parks
workplaces
residential

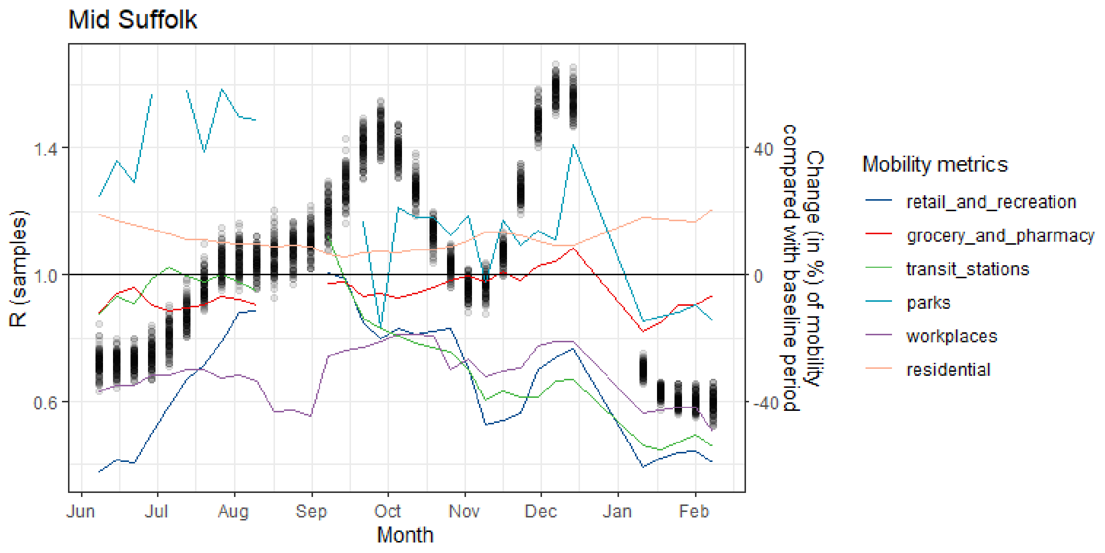
Mid Suffolk
R (samples)
1.4
1.0
0.6
40
0
-40
Change (in %) of mobility compared with baseline period
Jun
Jul
Aug
Sep
Oct
Nov
Dec
Jan
Feb
Month
Mobility metrics
retail_and_recreation
grocery_and_pharmacy
transit_stations
parks
workplaces
residential

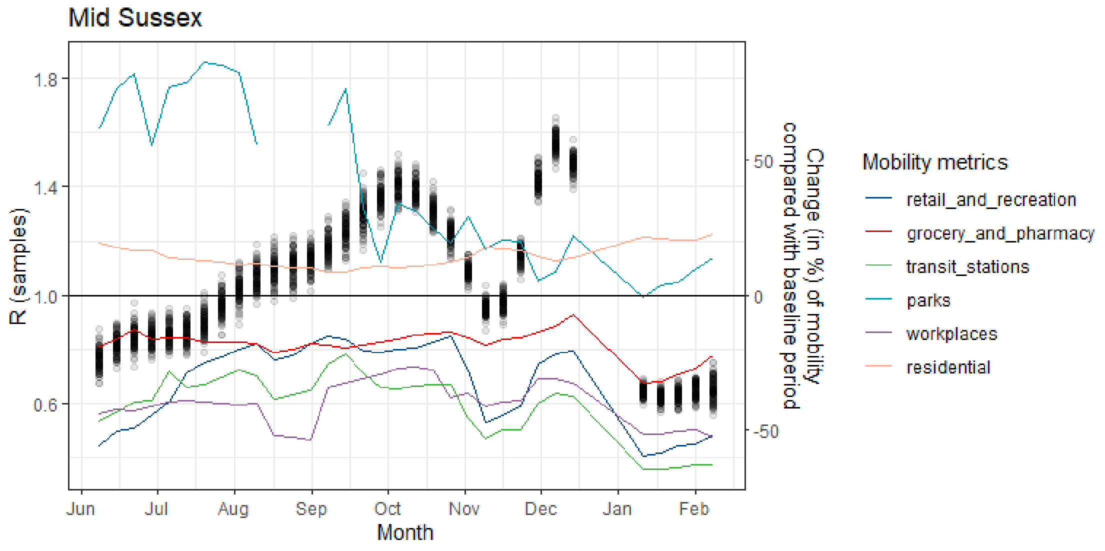
Mid Sussex
R (samples)
1.8
1.4
1.0
0.6
50
0
-50
Change (in %) of mobility compared with baseline period
Jun
Jul
Aug
Sep
Oct
Nov
Dec
Jan
Feb
Month
Mobility metrics
retail_and_recreation
grocery_and_pharmacy
transit_stations
parks
workplaces
residential

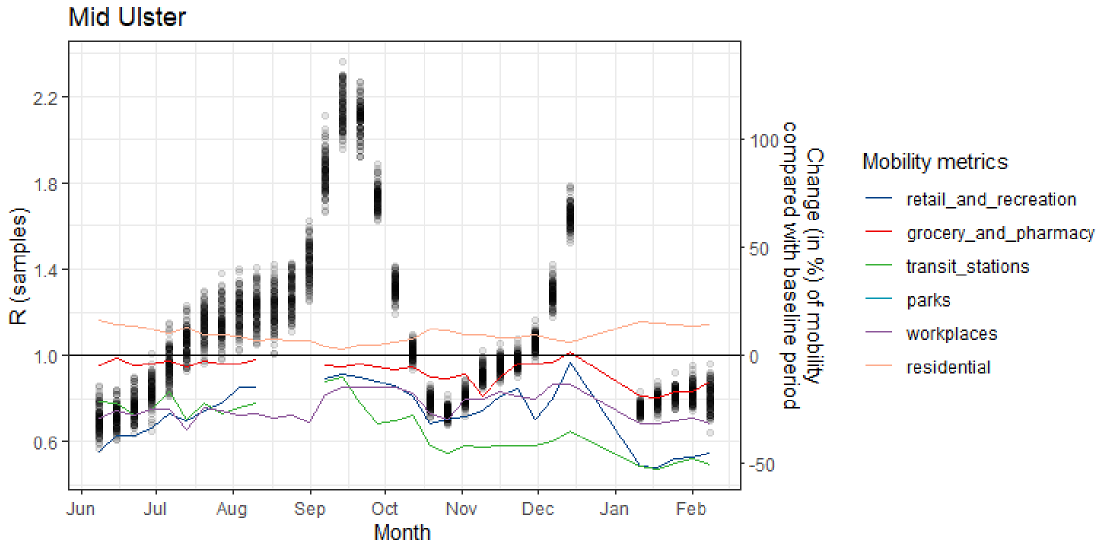
Mid Ulster
R (samples)
2.2
1.8
1.4
1.0
0.6
100
50
0
-50
Change (in %) of mobility compared with baseline period
Jun
Jul
Aug
Sep
Oct
Nov
Dec
Jan
Feb
Month
Mobility metrics
retail_and_recreation
grocery_and_pharmacy
transit_stations
parks
workplaces
residential

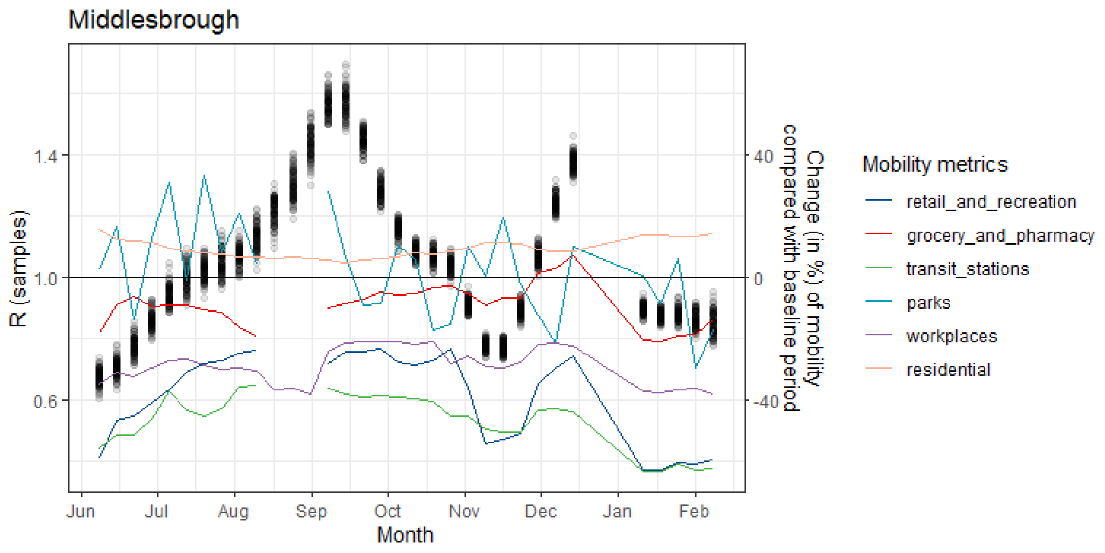
Middlesbrough
R (samples)
1.4
1.0
0.6
40
0
-40
Change (in %) of mobility compared with baseline period
Jun
Jul
Aug
Sep
Oct
Nov
Dec
Jan
Feb
Month
Mobility metrics
retail_and_recreation
grocery_and_pharmacy
transit_stations
parks
workplaces
residential

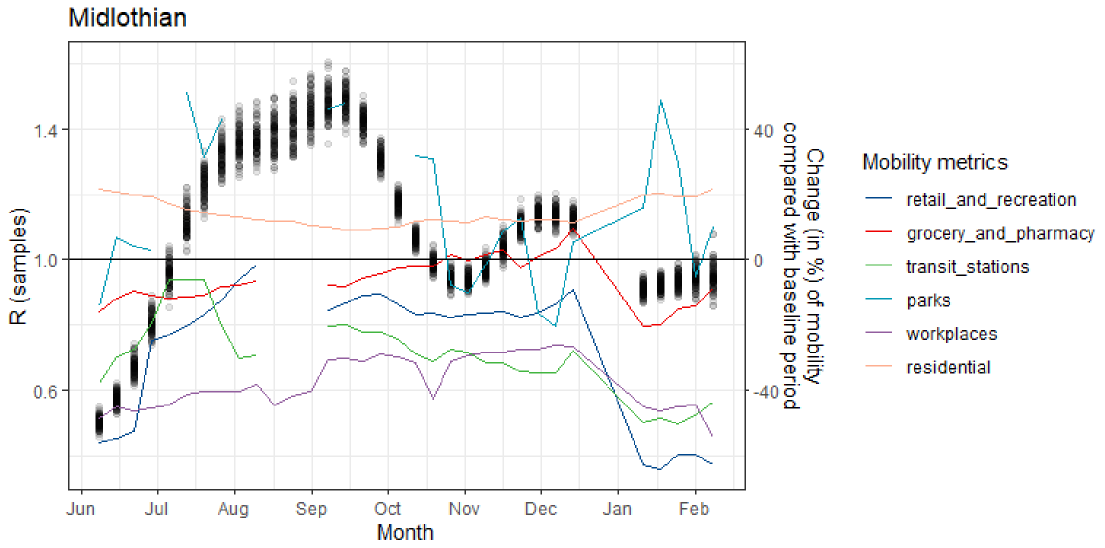
Midlothian
R (samples)
1.4
1.0
0.6
40
0
-40
Change (in %) of mobility compared with baseline period
Jun
Jul
Aug
Sep
Oct
Nov
Dec
Jan
Feb
Month
Mobility metrics
retail_and_recreation
grocery_and_pharmacy
transit_stations
parks
workplaces
residential

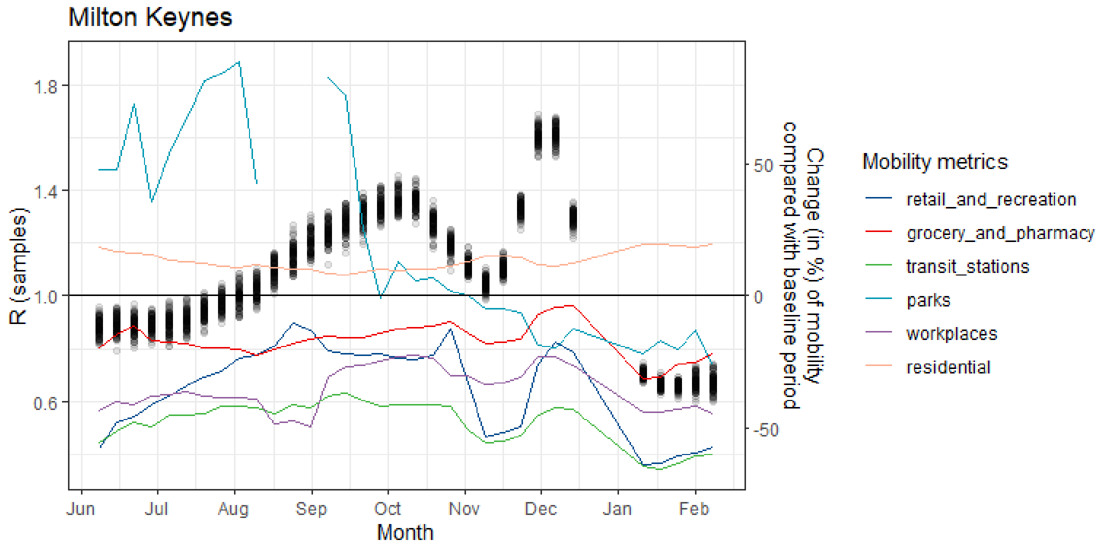
Milton Keynes
R (samples)
1.8
1.4
1.0
0.6
50
0
-50
Change (in %) of mobility compared with baseline period
Jun
Jul
Aug
Sep
Oct
Nov
Dec
Jan
Feb
Month
Mobility metrics
retail_and_recreation
grocery_and_pharmacy
transit_stations
parks
workplaces
residential

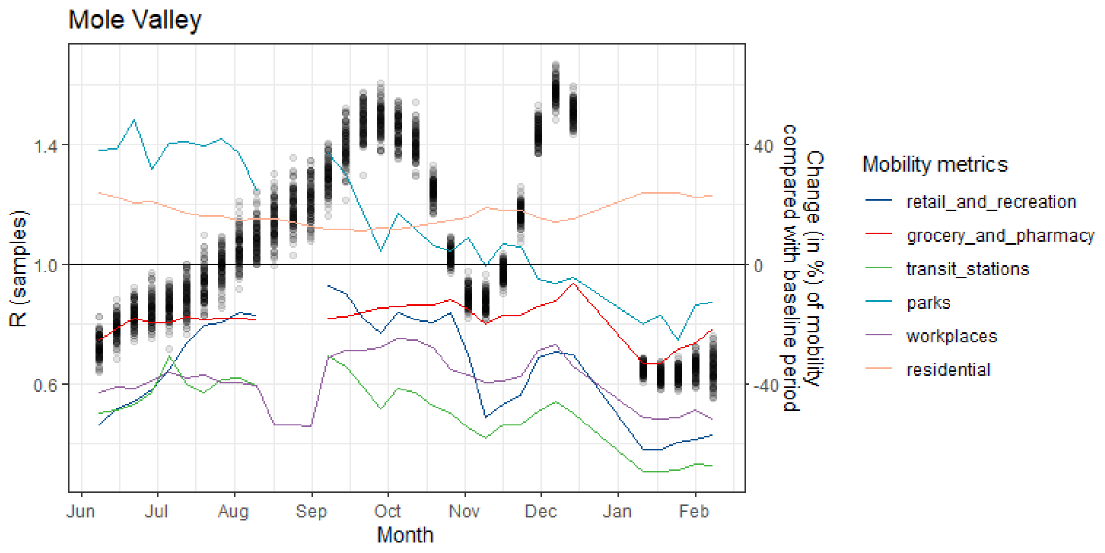
Mole Valley
R (samples)
1.4
1.0
0.6
Jun
Jul
Aug
Sep
Oct
Nov
Dec
Jan
Feb
Month
40
0
-40
Change (in %) of mobility
compared with baseline period
Mobility metrics
retail_and_recreation
grocery_and_pharmacy
transit_stations
parks
workplaces
residential

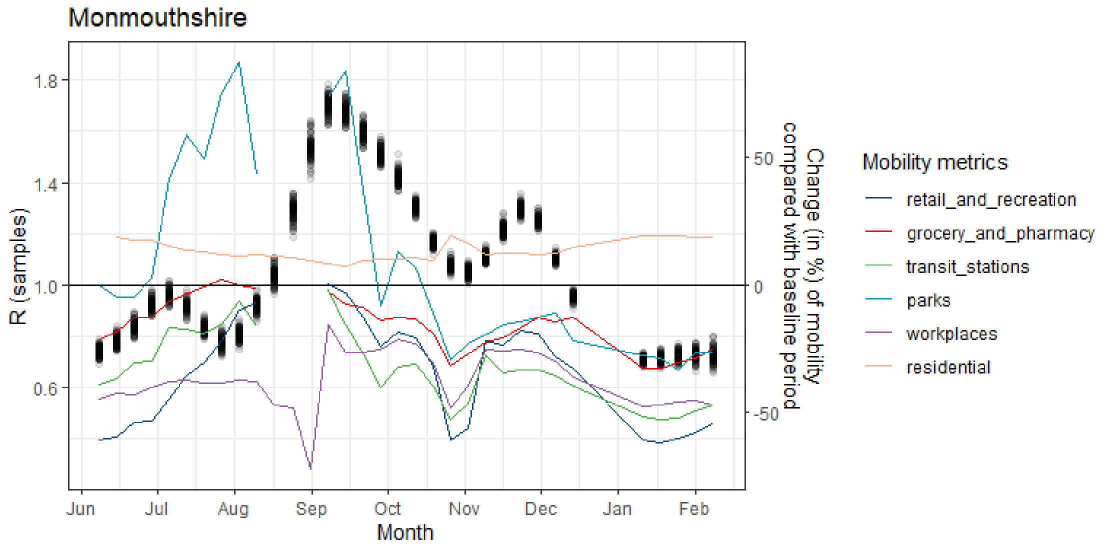
Monmouthshire
R (samples)
1.8
1.4
1.0
0.6
Jun
Jul
Aug
Sep
Oct
Nov
Dec
Jan
Feb
Month
50
0
-50
Change (in %) of mobility
compared with baseline period
Mobility metrics
retail_and_recreation
grocery_and_pharmacy
transit_stations
parks
workplaces
residential

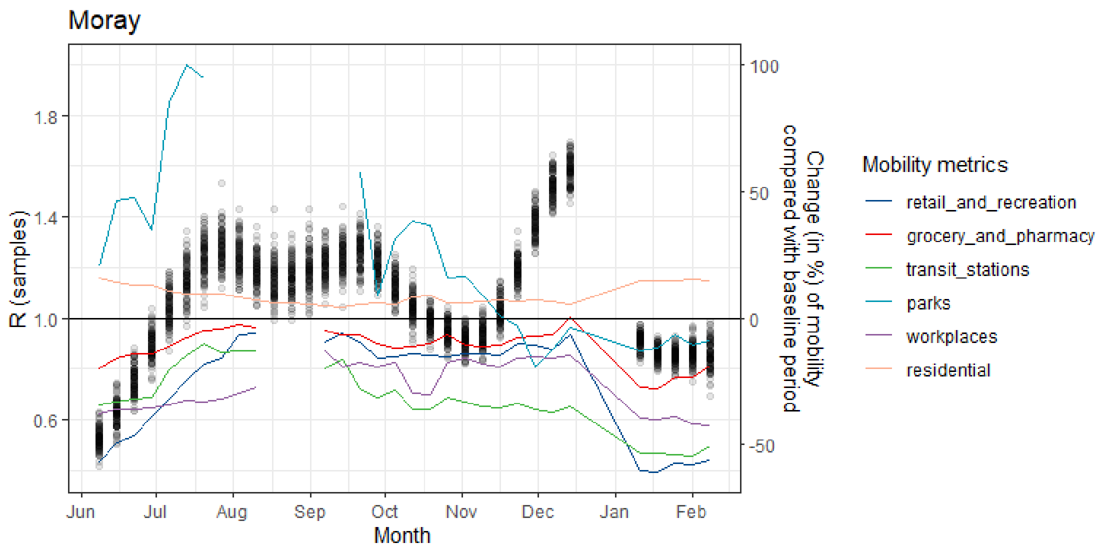
Moray
R (samples)
1.8
1.4
1.0
0.6
Jun
Jul
Aug
Sep
Oct
Nov
Dec
Jan
Feb
Month
100
50
0
-50
Change (in %) of mobility
compared with baseline period
Mobility metrics
retail_and_recreation
grocery_and_pharmacy
transit_stations
parks
workplaces
residential

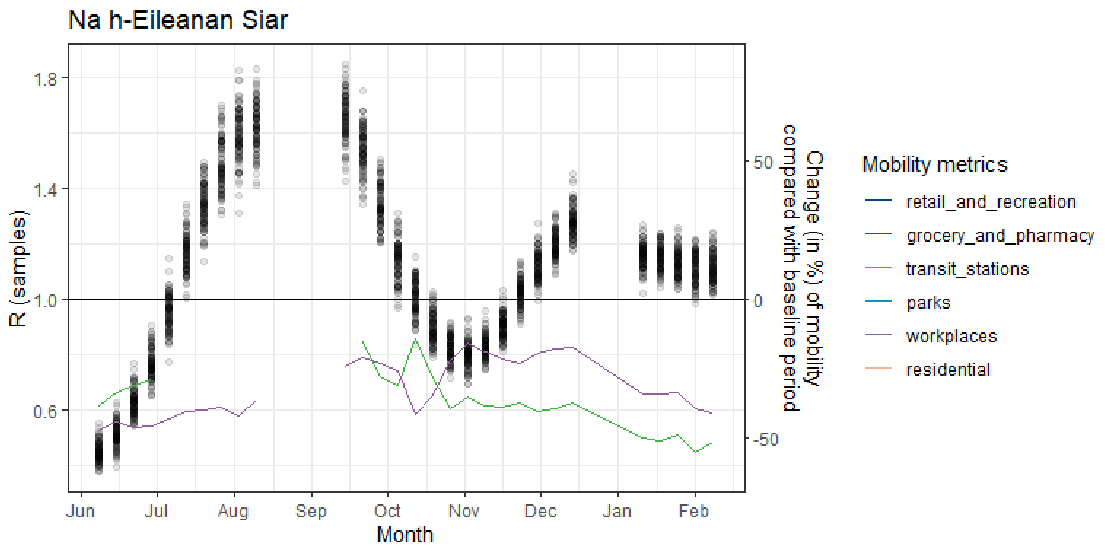
Na h-Eileanan Siar
R (samples)
Change (in %) of mobility compared with baseline period
Month
Jun
Jul
Aug
Sep
Oct
Nov
Dec
Jan
Feb
Mobility metrics
retail_and_recreation
grocery_and_pharmacy
transit_stations
parks
workplaces
residential

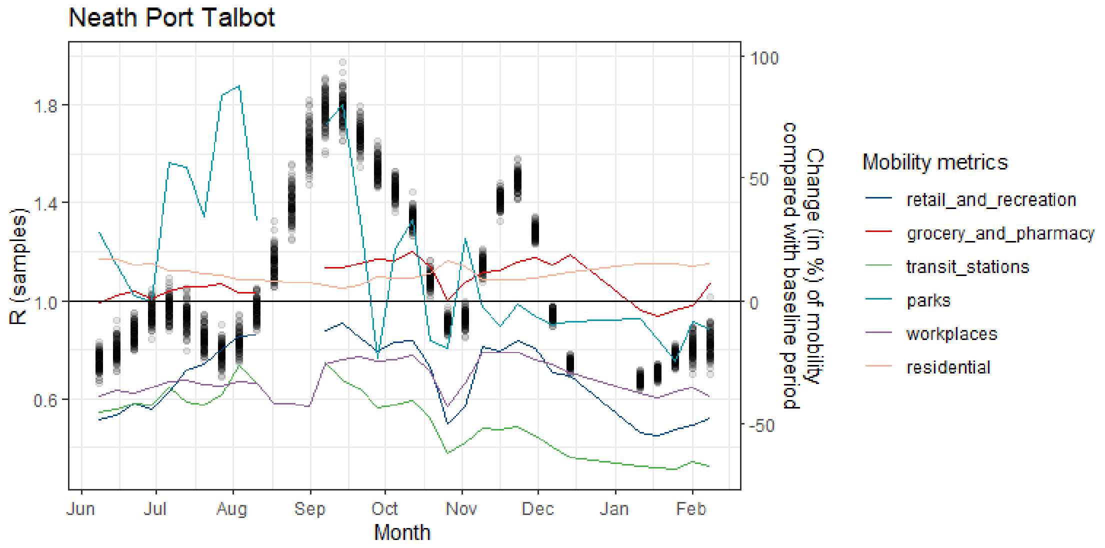
Neath Port Talbot
R (samples)
Change (in %) of mobility compared with baseline period
Month
Jun
Jul
Aug
Sep
Oct
Nov
Dec
Jan
Feb
Mobility metrics
retail_and_recreation
grocery_and_pharmacy
transit_stations
parks
workplaces
residential

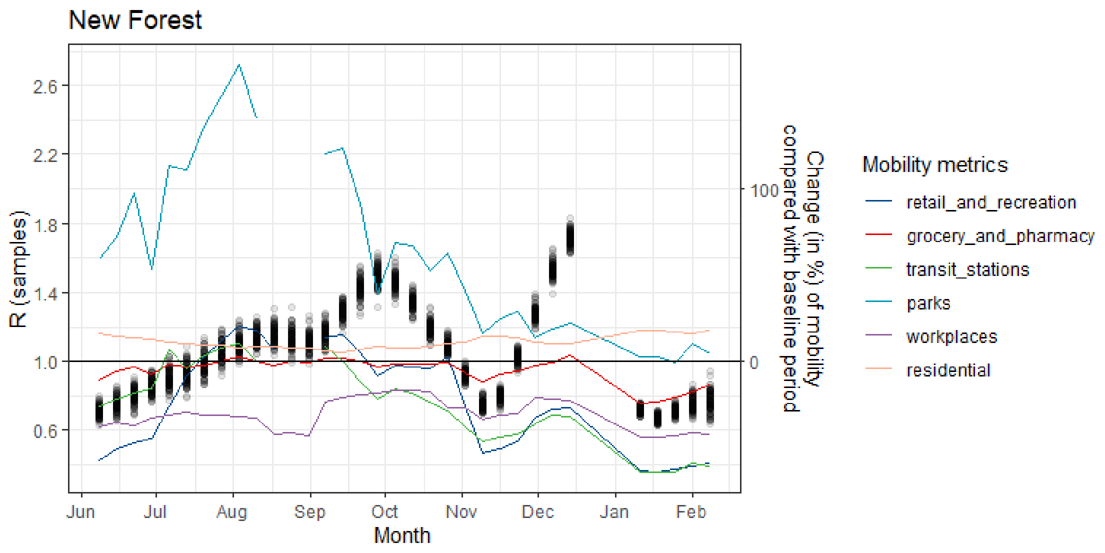
New Forest
R (samples)
Change (in %) of mobility compared with baseline period
Month
Jun
Jul
Aug
Sep
Oct
Nov
Dec
Jan
Feb
Mobility metrics
retail_and_recreation
grocery_and_pharmacy
transit_stations
parks
workplaces
residential

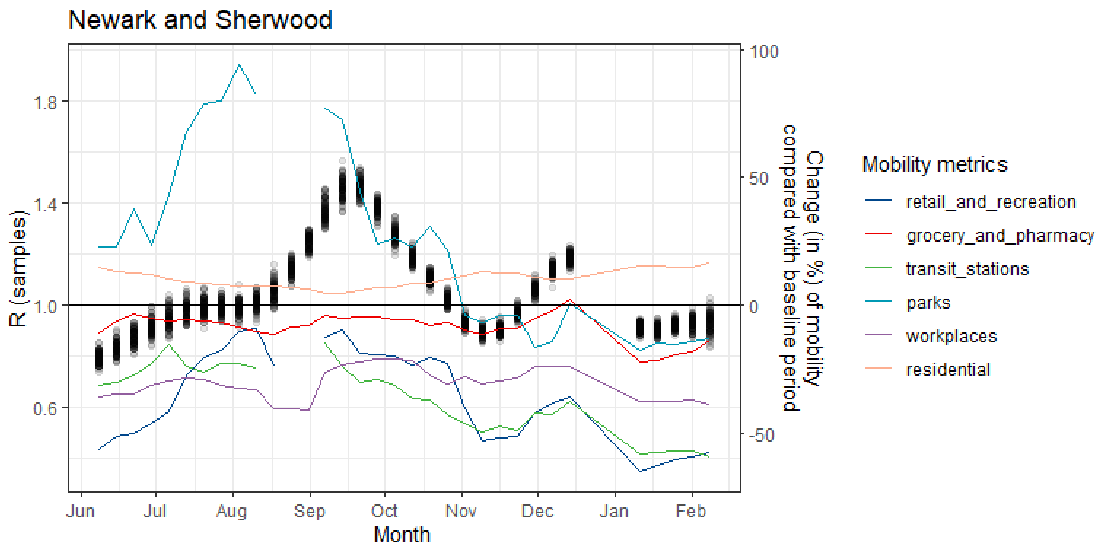

Newark and Sherwood
R (samples)
1.8
1.4
1.0
0.6
100
50
0
-50
Change (in %) of mobility compared with baseline period
Jun
Jul
Aug
Sep
Oct
Nov
Dec
Jan
Feb
Month
Mobility metrics
retail_and_recreation
grocery_and_pharmacy
transit_stations
parks
workplaces
residential

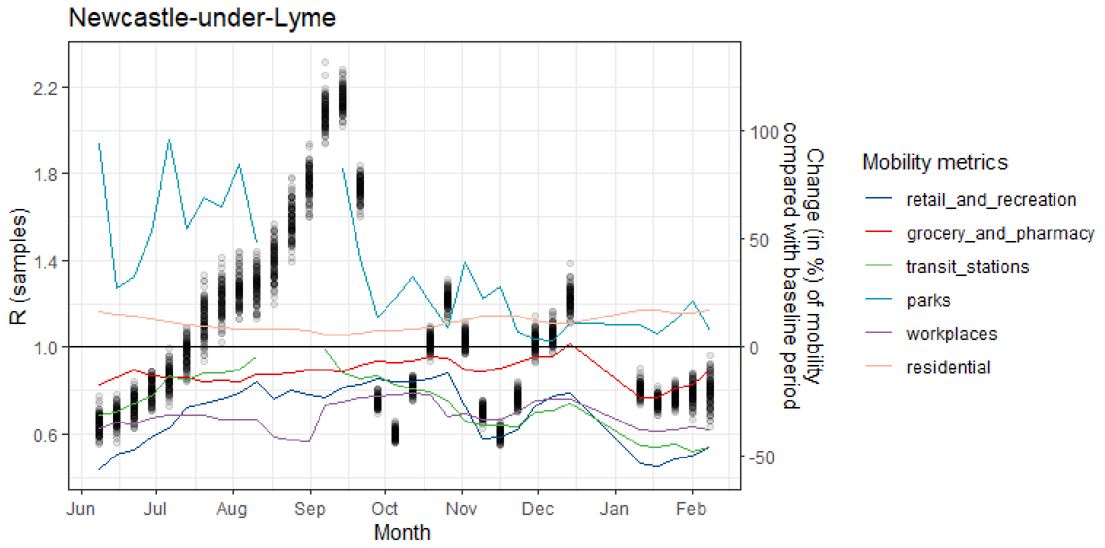

Newcastle-under-Lyme
R (samples)
2.2
1.8
1.4
1.0
0.6
100
50
0
-50
Change (in %) of mobility compared with baseline period
Jun
Jul
Aug
Sep
Oct
Nov
Dec
Jan
Feb
Month
Mobility metrics
retail_and_recreation
grocery_and_pharmacy
transit_stations
parks
workplaces
residential

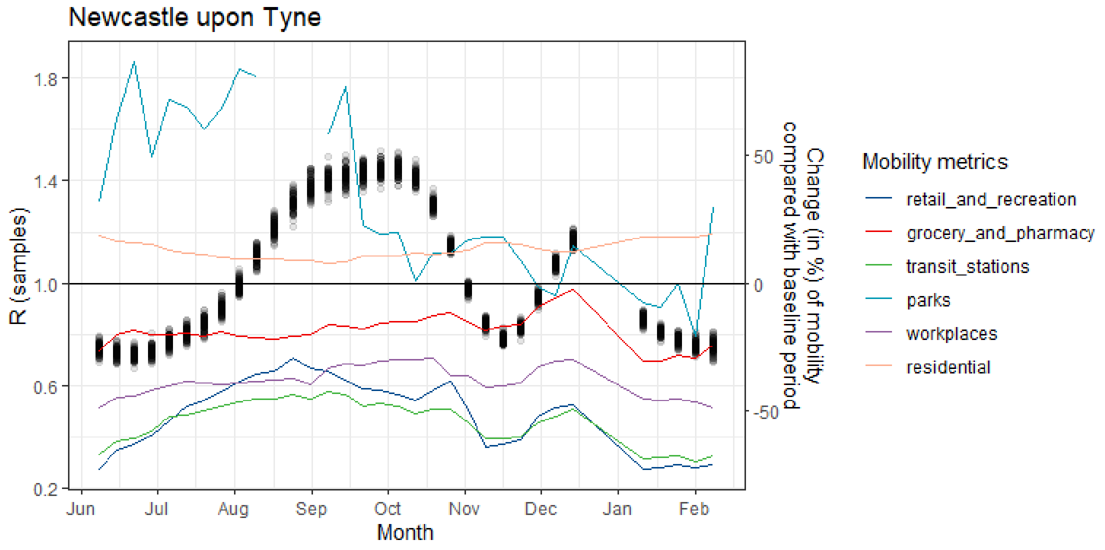

Newcastle upon Tyne
R (samples)
1.8
1.4
1.0
0.6
0.2
50
0
-50
Change (in %) of mobility compared with baseline period
Jun
Jul
Aug
Sep
Oct
Nov
Dec
Jan
Feb
Month
Mobility metrics
retail_and_recreation
grocery_and_pharmacy
transit_stations
parks
workplaces
residential

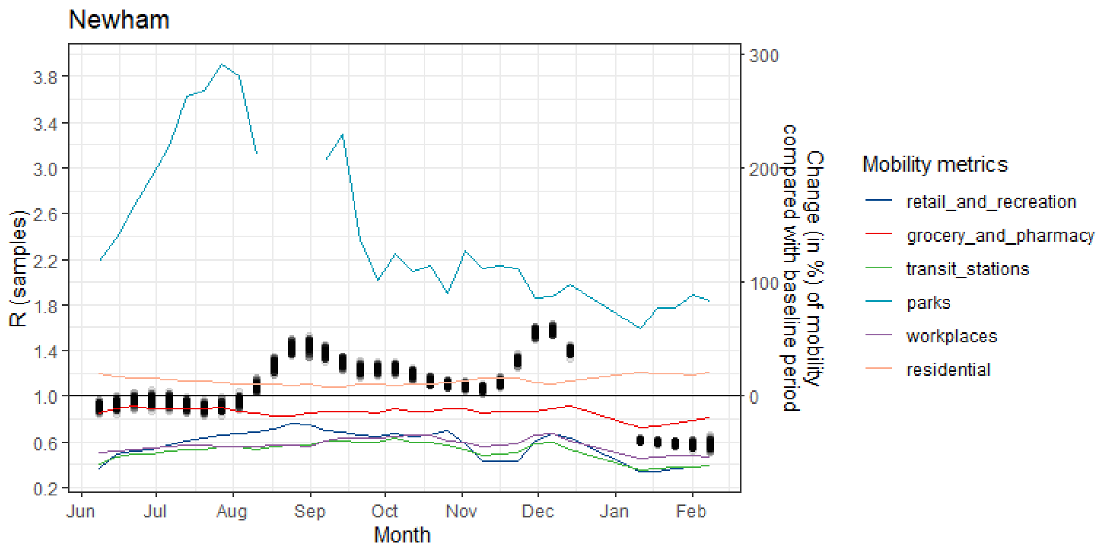
Newham
R (samples)
Change (in %) of mobility compared with baseline period
Month
Mobility metrics
retail_and_recreation
grocery_and_pharmacy
transit_stations
parks
workplaces
residential

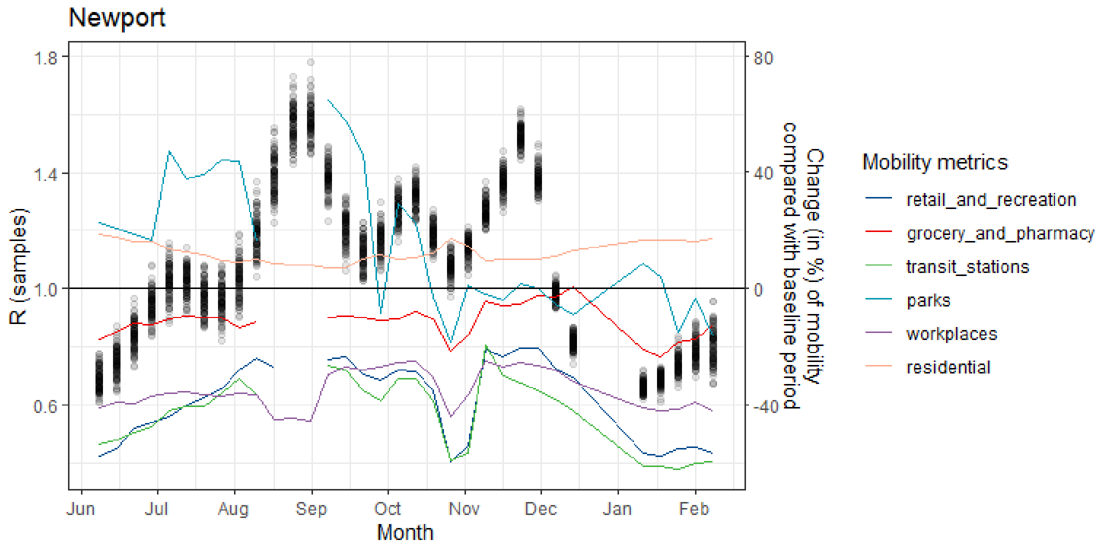
Newport
R (samples)
Change (in %) of mobility compared with baseline period
Month
Mobility metrics
retail_and_recreation
grocery_and_pharmacy
transit_stations
parks
workplaces
residential

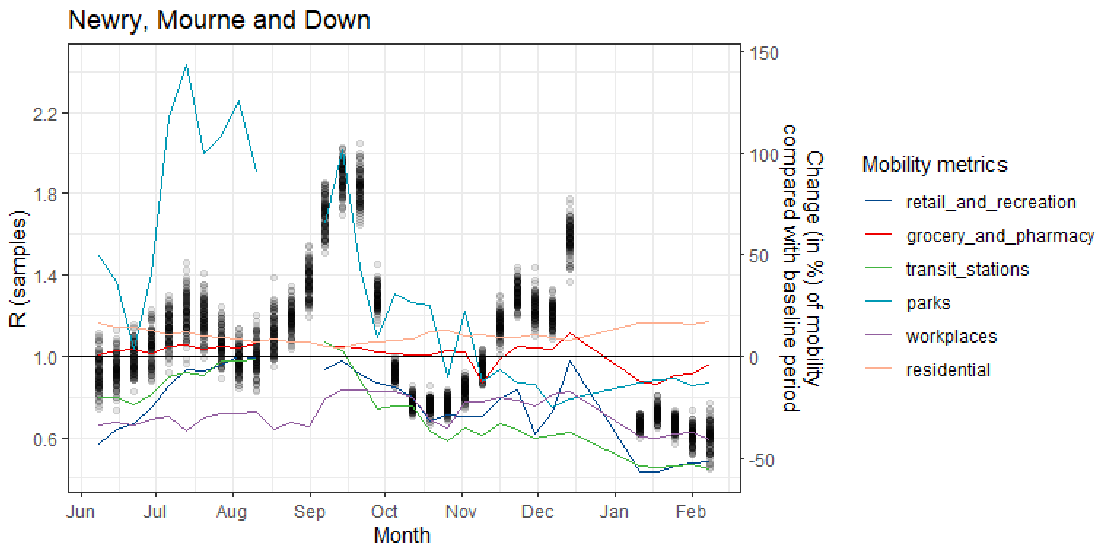
Newry, Mourne and Down
R (samples)
Change (in %) of mobility compared with baseline period
Month
Mobility metrics
retail_and_recreation
grocery_and_pharmacy
transit_stations
parks
workplaces
residential

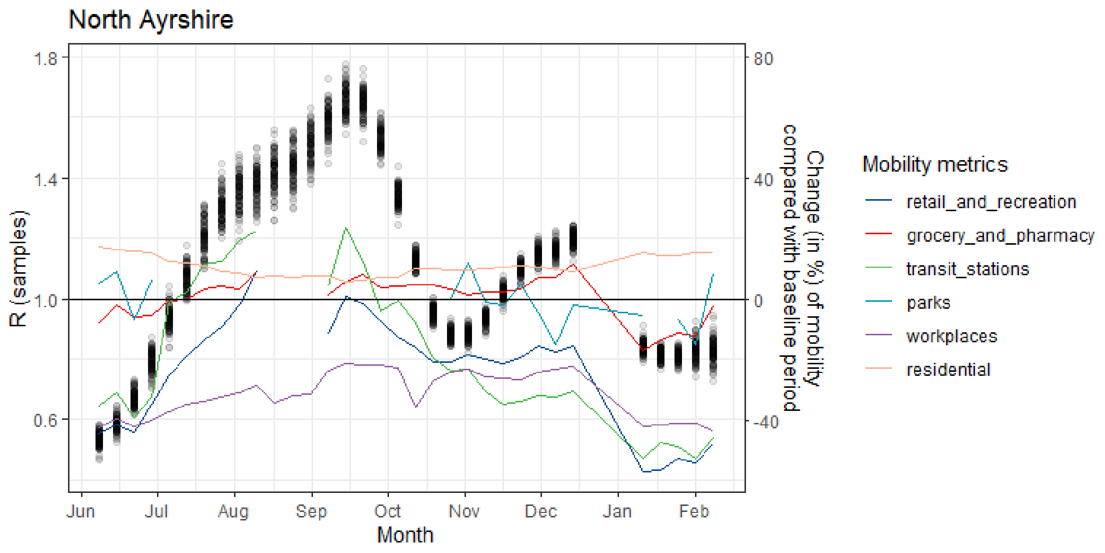
North Ayrshire
R (samples)
Change (in %) of mobility compared with baseline period
Month
Jun
Jul
Aug
Sep
Oct
Nov
Dec
Jan
Feb
Mobility metrics
retail_and_recreation
grocery_and_pharmacy
transit_stations
parks
workplaces
residential

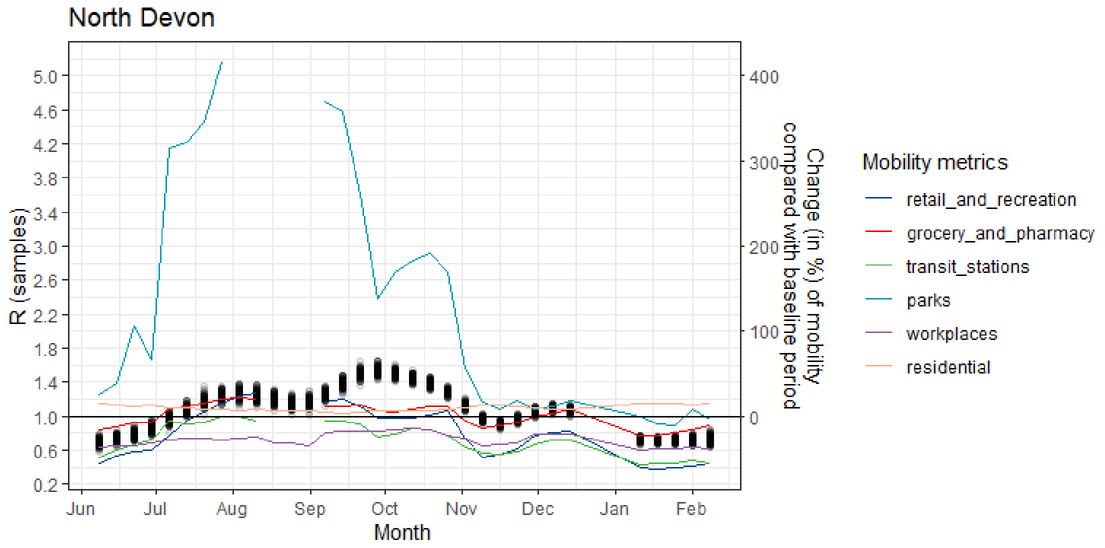
North Devon
R (samples)
Change (in %) of mobility compared with baseline period
Month
Jun
Jul
Aug
Sep
Oct
Nov
Dec
Jan
Feb
Mobility metrics
retail_and_recreation
grocery_and_pharmacy
transit_stations
parks
workplaces
residential

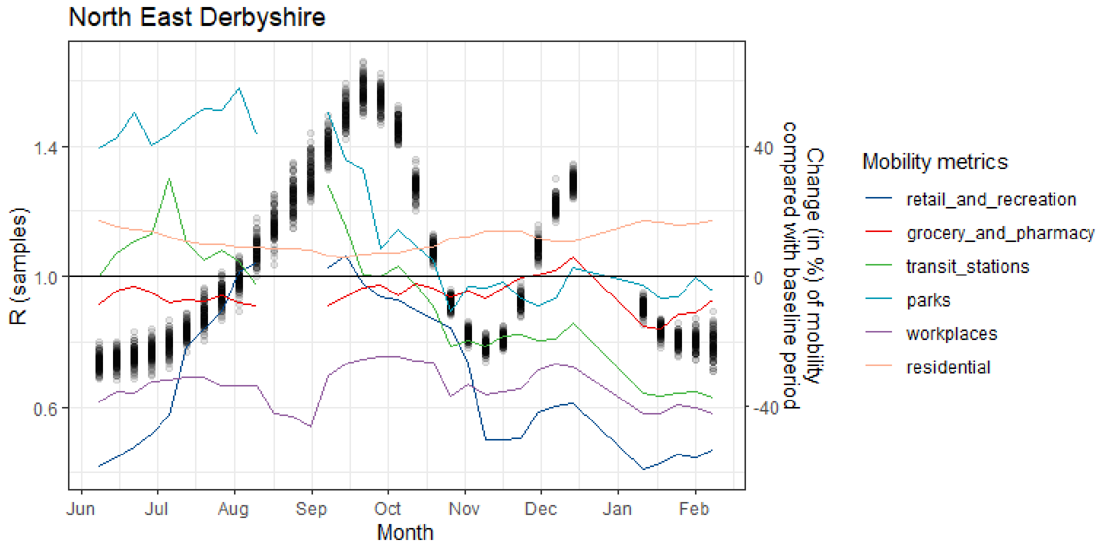
North East Derbyshire
R (samples)
Change (in %) of mobility compared with baseline period
Month
Jun
Jul
Aug
Sep
Oct
Nov
Dec
Jan
Feb
Mobility metrics
retail_and_recreation
grocery_and_pharmacy
transit_stations
parks
workplaces
residential

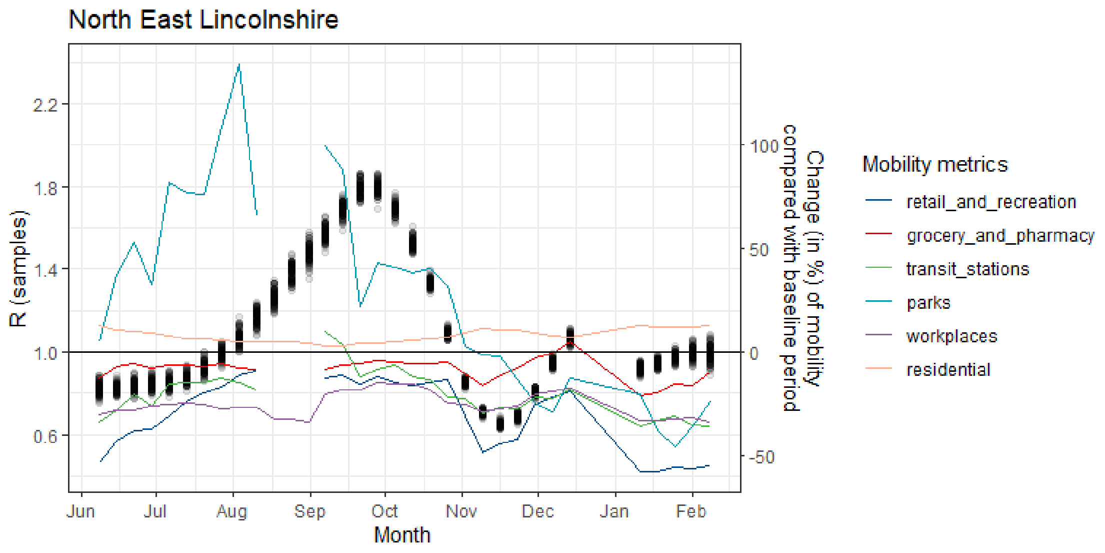
North East Lincolnshire
R (samples)
2.2
1.8
1.4
1.0
0.6
100
50
0
-50
Change (in %) of mobility compared with baseline period
Jun
Jul
Aug
Sep
Oct
Nov
Dec
Jan
Feb
Month
Mobility metrics
retail_and_recreation
grocery_and_pharmacy
transit_stations
parks
workplaces
residential

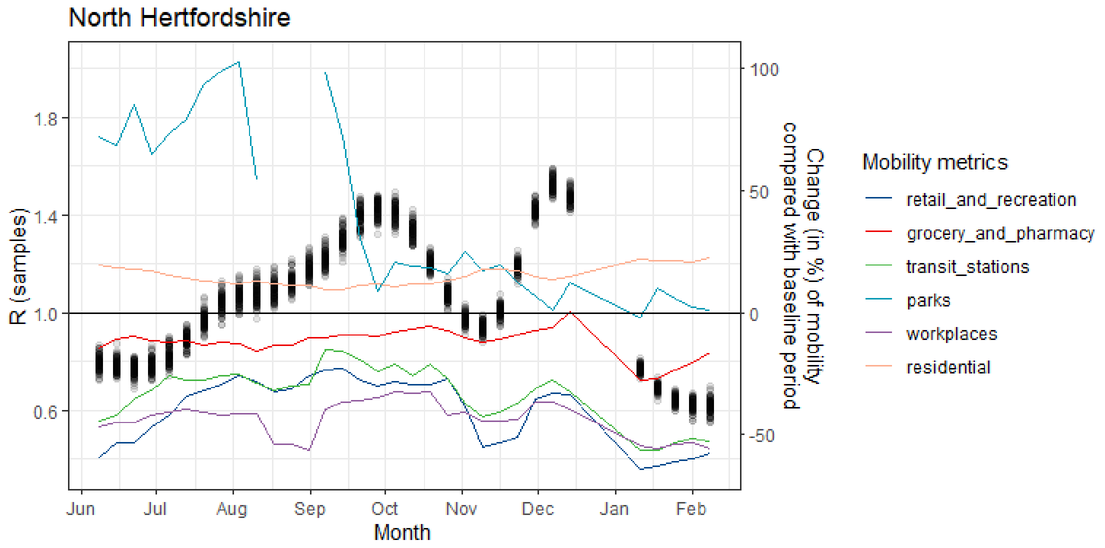
North Hertfordshire
R (samples)
1.8
1.4
1.0
0.6
100
50
0
-50
Change (in %) of mobility compared with baseline period
Jun
Jul
Aug
Sep
Oct
Nov
Dec
Jan
Feb
Month
Mobility metrics
retail_and_recreation
grocery_and_pharmacy
transit_stations
parks
workplaces
residential

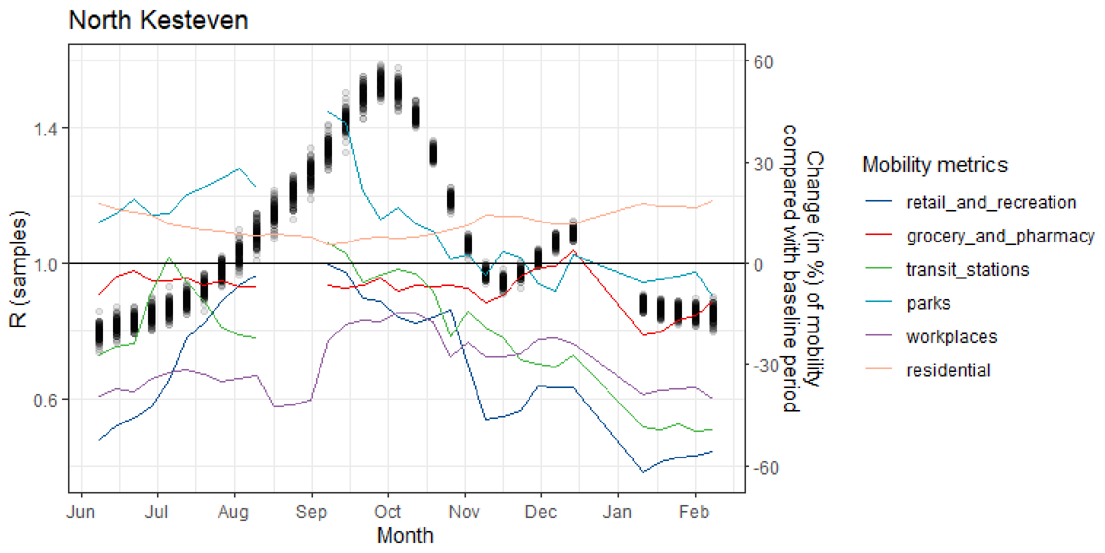
North Kesteven
R (samples)
1.4
1.0
0.6
60
30
0
-30
-60
Change (in %) of mobility compared with baseline period
Jun
Jul
Aug
Sep
Oct
Nov
Dec
Jan
Feb
Month
Mobility metrics
retail_and_recreation
grocery_and_pharmacy
transit_stations
parks
workplaces
residential

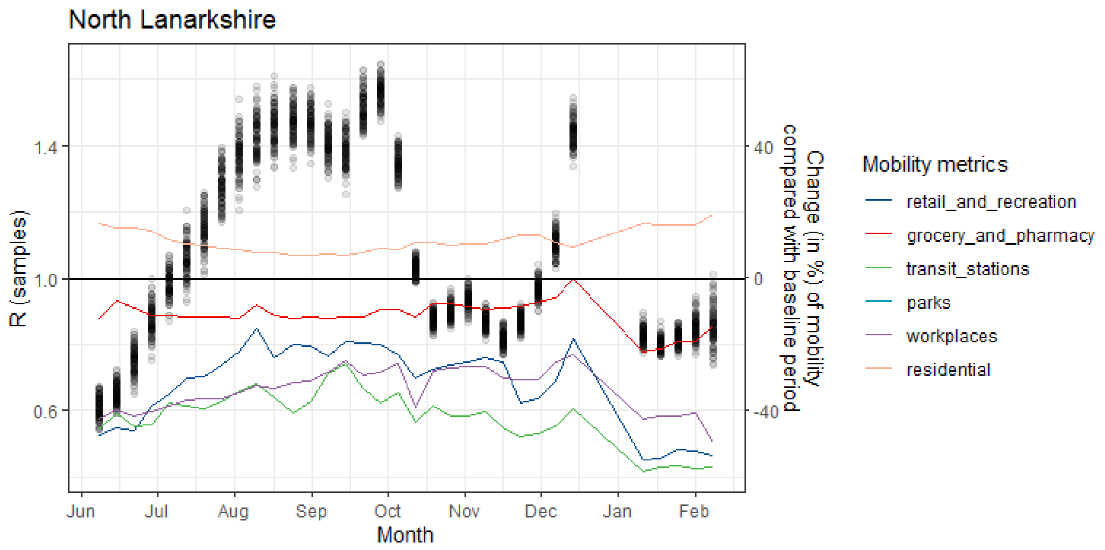
North Lanarkshire
R (samples)
1.4
1.0
0.6
Jun
Jul
Aug
Sep
Oct
Nov
Dec
Jan
Feb
Month
40
0
-40
Change (in %) of mobility compared with baseline period
Mobility metrics
retail_and_recreation
grocery_and_pharmacy
transit_stations
parks
workplaces
residential

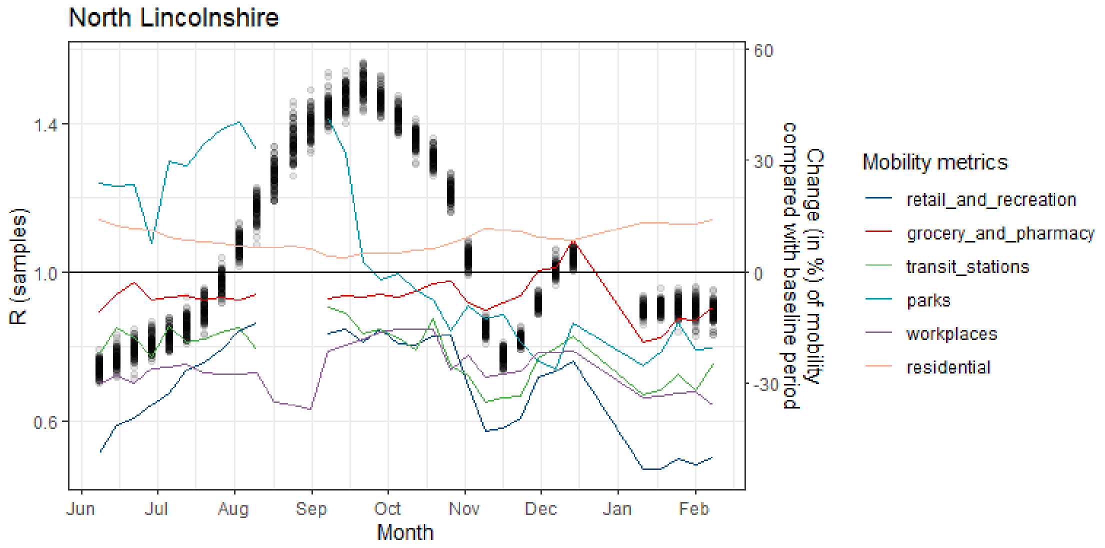
North Lincolnshire
R (samples)
1.4
1.0
0.6
Jun
Jul
Aug
Sep
Oct
Nov
Dec
Jan
Feb
Month
60
30
0
-30
Change (in %) of mobility compared with baseline period
Mobility metrics
retail_and_recreation
grocery_and_pharmacy
transit_stations
parks
workplaces
residential

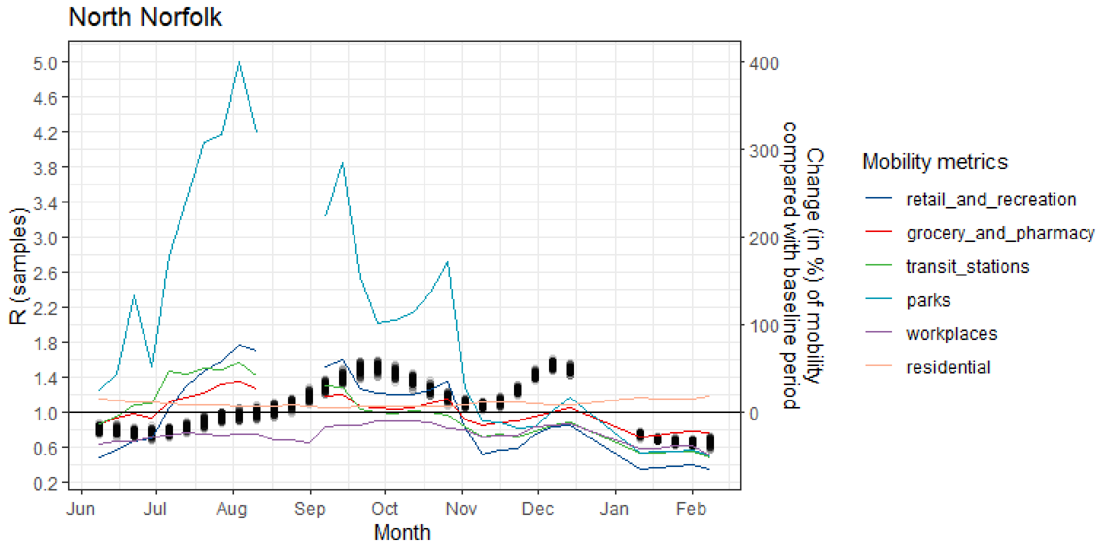
North Norfolk
R (samples)
5.0
4.6
4.2
3.8
3.4
3.0
2.6
2.2
1.8
1.4
1.0
0.6
0.2
Jun
Jul
Aug
Sep
Oct
Nov
Dec
Jan
Feb
Month
400
300
200
100
0
Change (in %) of mobility compared with baseline period
Mobility metrics
retail_and_recreation
grocery_and_pharmacy
transit_stations
parks
workplaces
residential

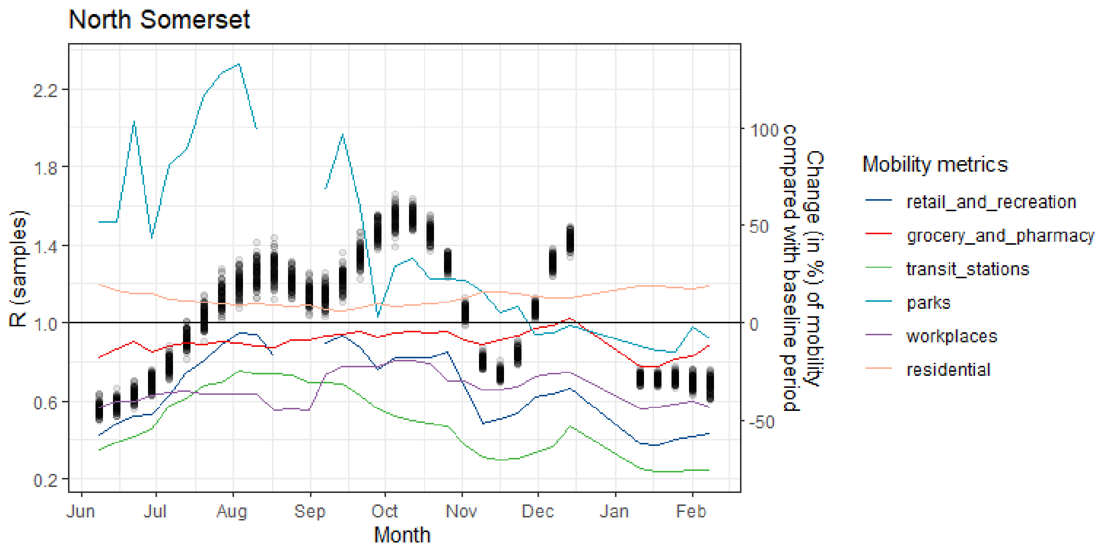
North Somerset
R (samples)
2.2
1.8
1.4
1.0
0.6
0.2
100
50
0
-50
Change (in %) of mobility compared with baseline period
Jun
Jul
Aug
Sep
Oct
Nov
Dec
Jan
Feb
Month
Mobility metrics
retail_and_recreation
grocery_and_pharmacy
transit_stations
parks
workplaces
residential

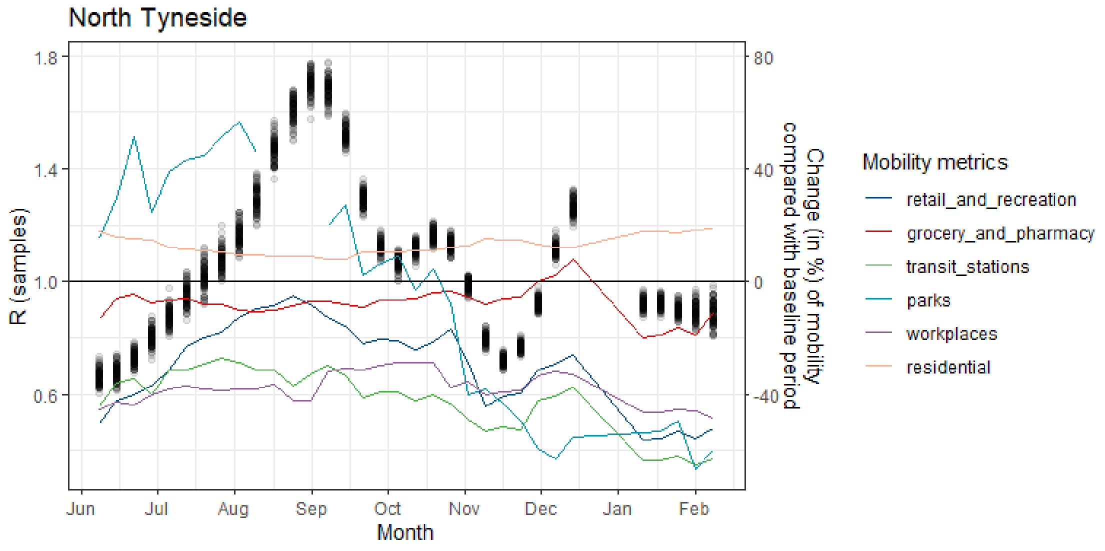
North Tyneside
R (samples)
1.8
1.4
1.0
0.6
80
40
0
-40
Change (in %) of mobility compared with baseline period
Jun
Jul
Aug
Sep
Oct
Nov
Dec
Jan
Feb
Month
Mobility metrics
retail_and_recreation
grocery_and_pharmacy
transit_stations
parks
workplaces
residential

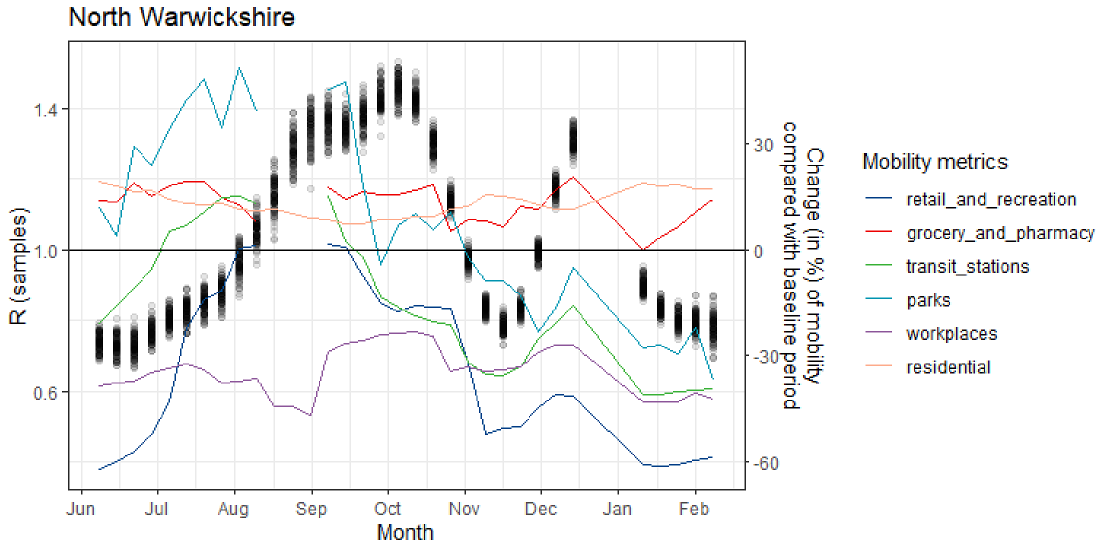
North Warwickshire
R (samples)
1.4
1.0
0.6
30
0
-30
-60
Change (in %) of mobility compared with baseline period
Jun
Jul
Aug
Sep
Oct
Nov
Dec
Jan
Feb
Month
Mobility metrics
retail_and_recreation
grocery_and_pharmacy
transit_stations
parks
workplaces
residential

North West Leicestershire

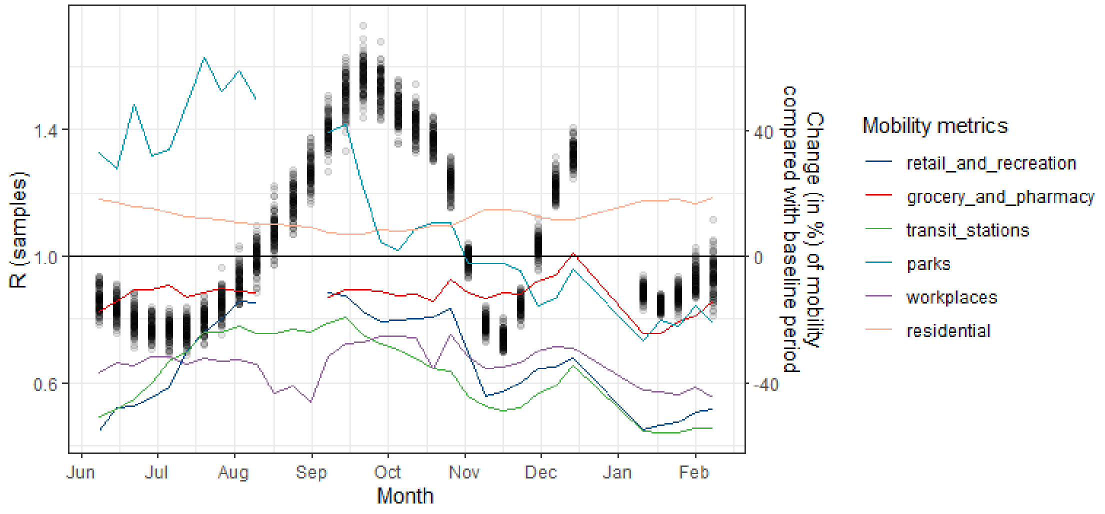

Northampton

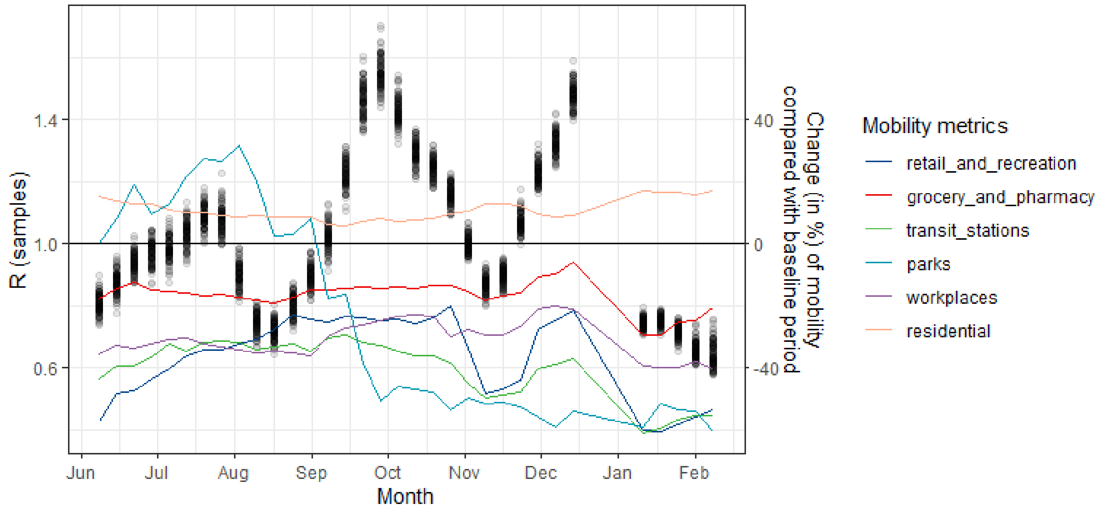

Northumberland

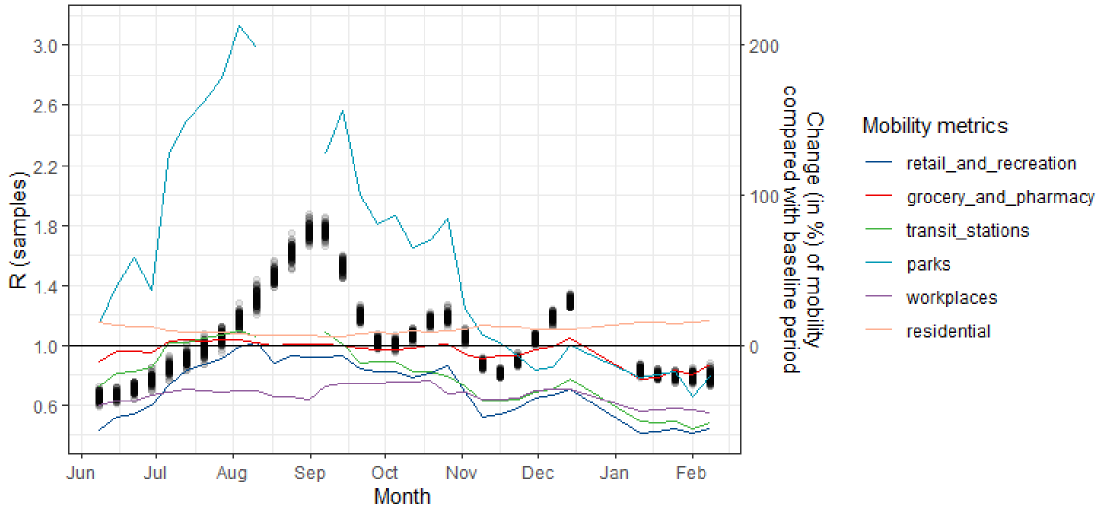

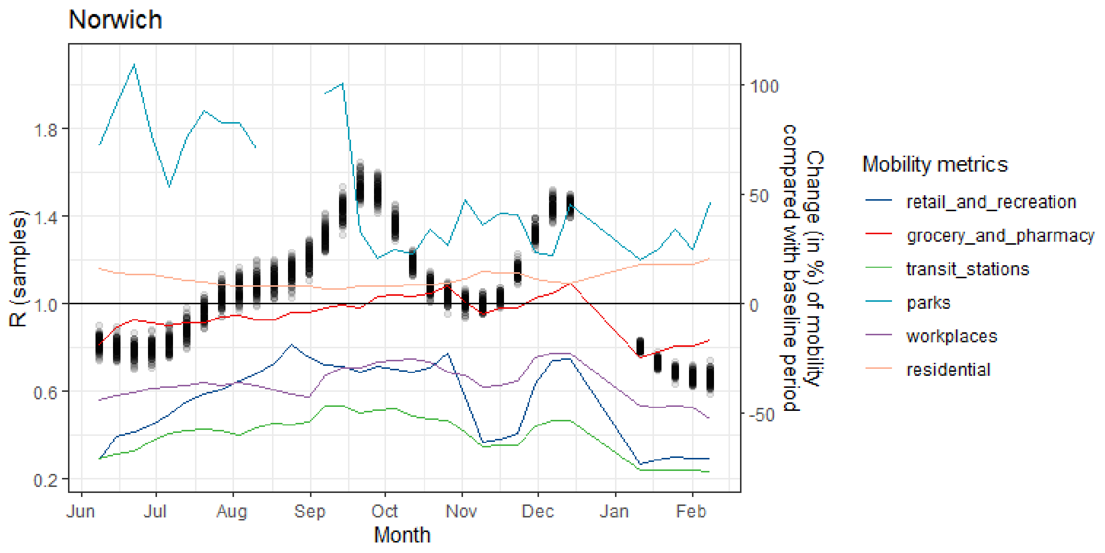
Norwich
R (samples)
1.8
1.4
1.0
0.6
0.2
Jun
Jul
Aug
Sep
Oct
Nov
Dec
Jan
Feb
Month
100
50
0
-50
Change (in %) of mobility compared with baseline period
Mobility metrics
retail_and_recreation
grocery_and_pharmacy
transit_stations
parks
workplaces
residential

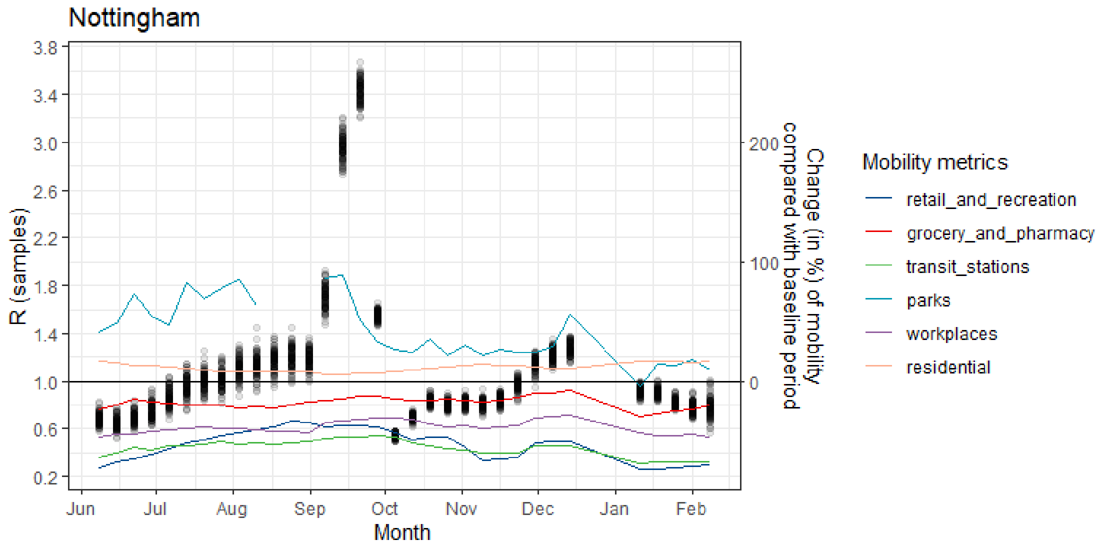
Nottingham
R (samples)
3.8
3.4
3.0
2.6
2.2
1.8
1.4
1.0
0.6
0.2
Jun
Jul
Aug
Sep
Oct
Nov
Dec
Jan
Feb
Month
200
100
0
Change (in %) of mobility compared with baseline period
Mobility metrics
retail_and_recreation
grocery_and_pharmacy
transit_stations
parks
workplaces
residential

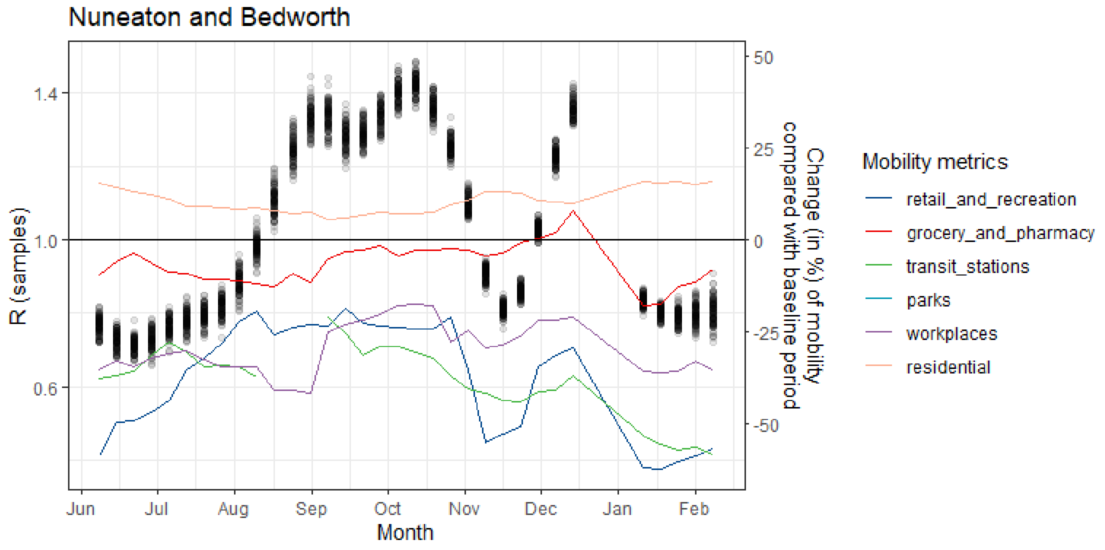
Nuneaton and Bedworth
R (samples)
1.4
1.0
0.6
Jun
Jul
Aug
Sep
Oct
Nov
Dec
Jan
Feb
Month
50
25
0
-25
-50
Change (in %) of mobility compared with baseline period
Mobility metrics
retail_and_recreation
grocery_and_pharmacy
transit_stations
parks
workplaces
residential

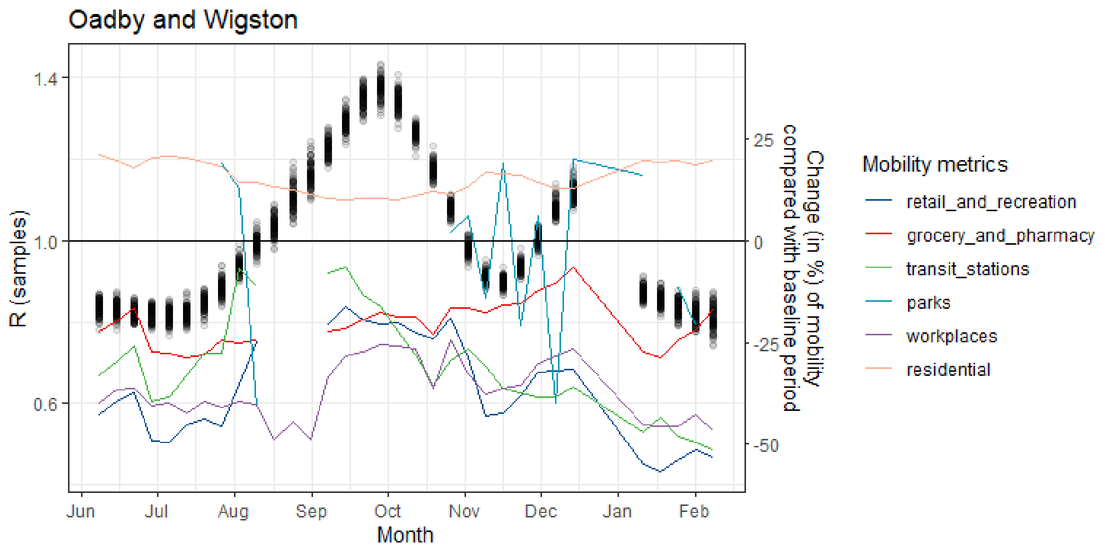
Oadby and Wigston
R (samples)
1.4
1.0
0.6
25
0
-25
-50
Change (in %) of mobility compared with baseline period
Jun
Jul
Aug
Sep
Oct
Nov
Dec
Jan
Feb
Month
Mobility metrics
retail_and_recreation
grocery_and_pharmacy
transit_stations
parks
workplaces
residential

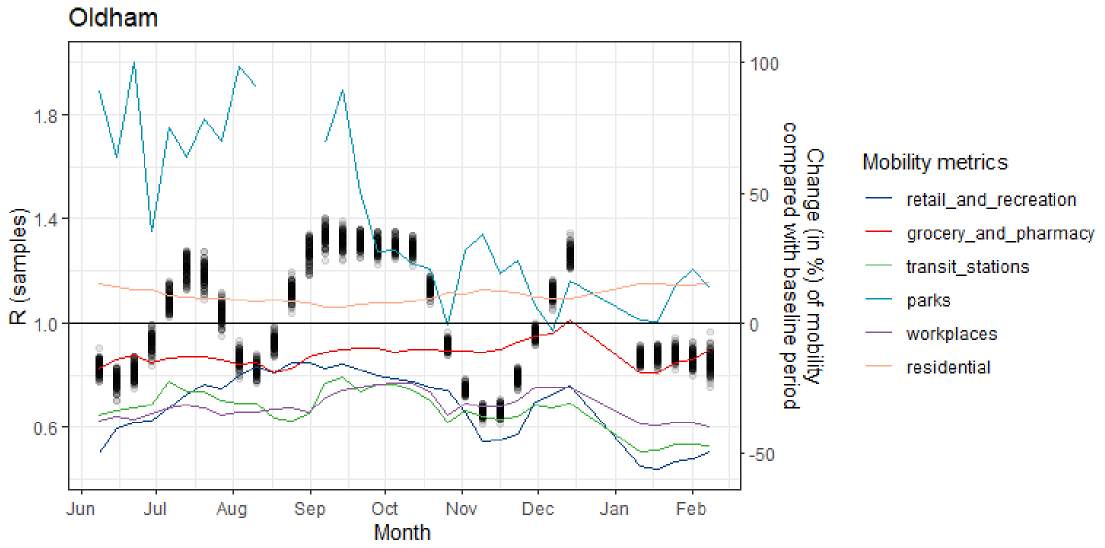
Oldham
R (samples)
1.8
1.4
1.0
0.6
100
50
0
-50
Change (in %) of mobility compared with baseline period
Jun
Jul
Aug
Sep
Oct
Nov
Dec
Jan
Feb
Month
Mobility metrics
retail_and_recreation
grocery_and_pharmacy
transit_stations
parks
workplaces
residential

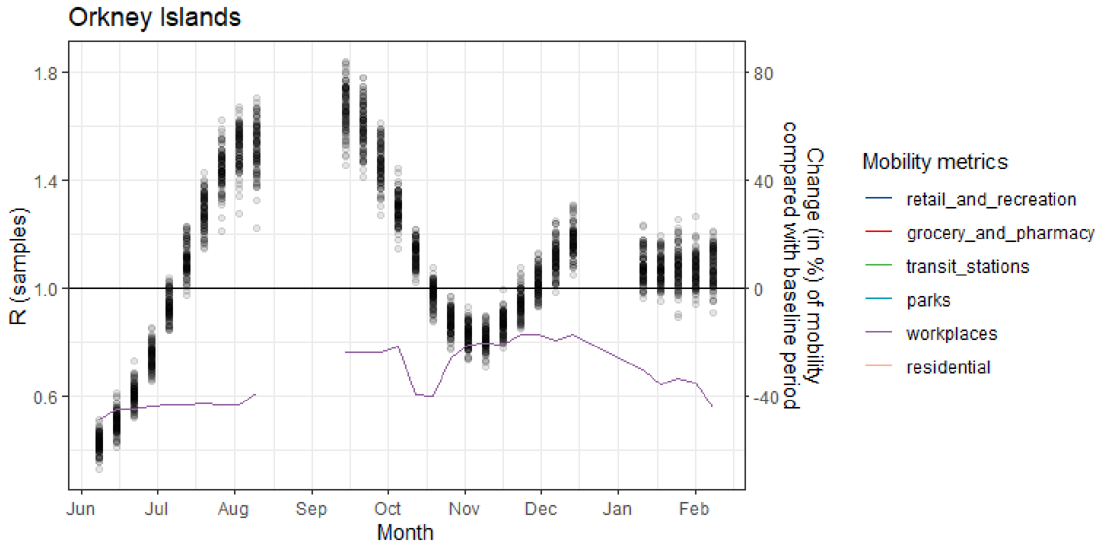
Orkney Islands
R (samples)
1.8
1.4
1.0
0.6
80
40
0
-40
Change (in %) of mobility compared with baseline period
Jun
Jul
Aug
Sep
Oct
Nov
Dec
Jan
Feb
Month
Mobility metrics
retail_and_recreation
grocery_and_pharmacy
transit_stations
parks
workplaces
residential

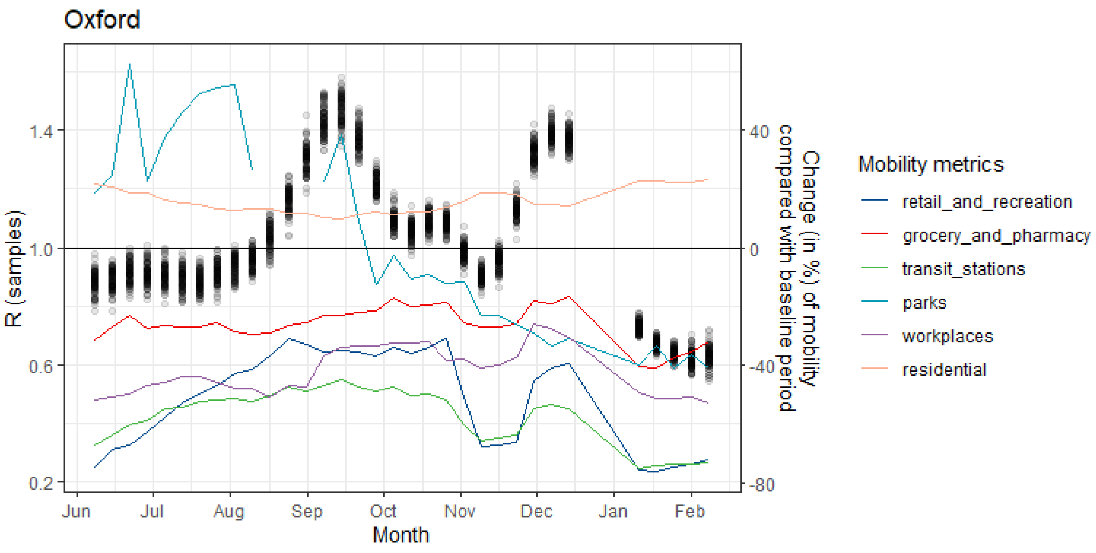
Oxford
R (samples)
1.4
1.0
0.6
0.2
Jun
Jul
Aug
Sep
Oct
Nov
Dec
Jan
Feb
Month
40
0
-40
-80
Change (in %) of mobility compared with baseline period
Mobility metrics
retail_and_recreation
grocery_and_pharmacy
transit_stations
parks
workplaces
residential

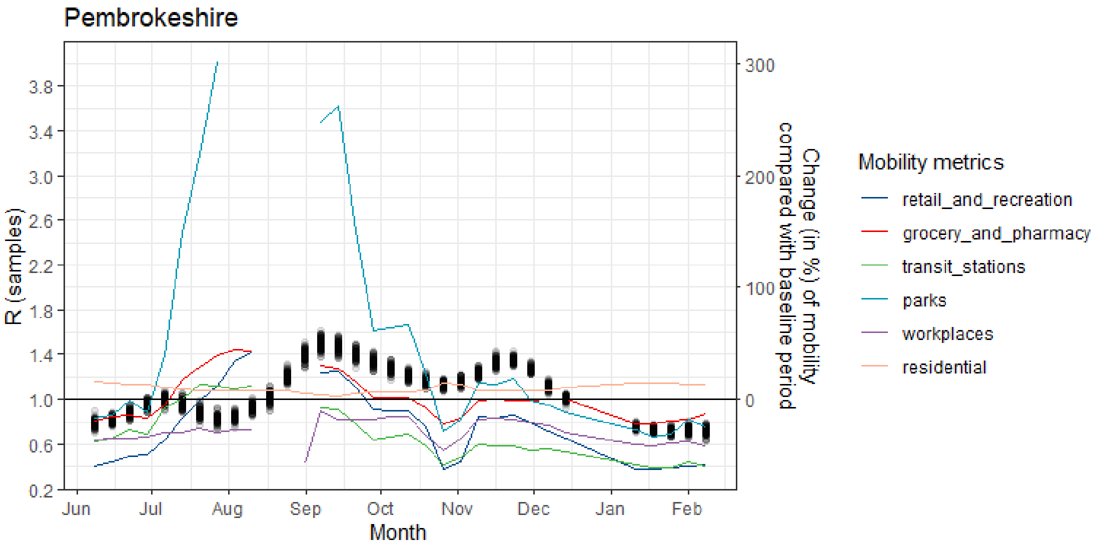
Pembrokeshire
R (samples)
3.8
3.4
3.0
2.6
2.2
1.8
1.4
1.0
0.6
0.2
Jun
Jul
Aug
Sep
Oct
Nov
Dec
Jan
Feb
Month
300
200
100
0
Change (in %) of mobility compared with baseline period
Mobility metrics
retail_and_recreation
grocery_and_pharmacy
transit_stations
parks
workplaces
residential

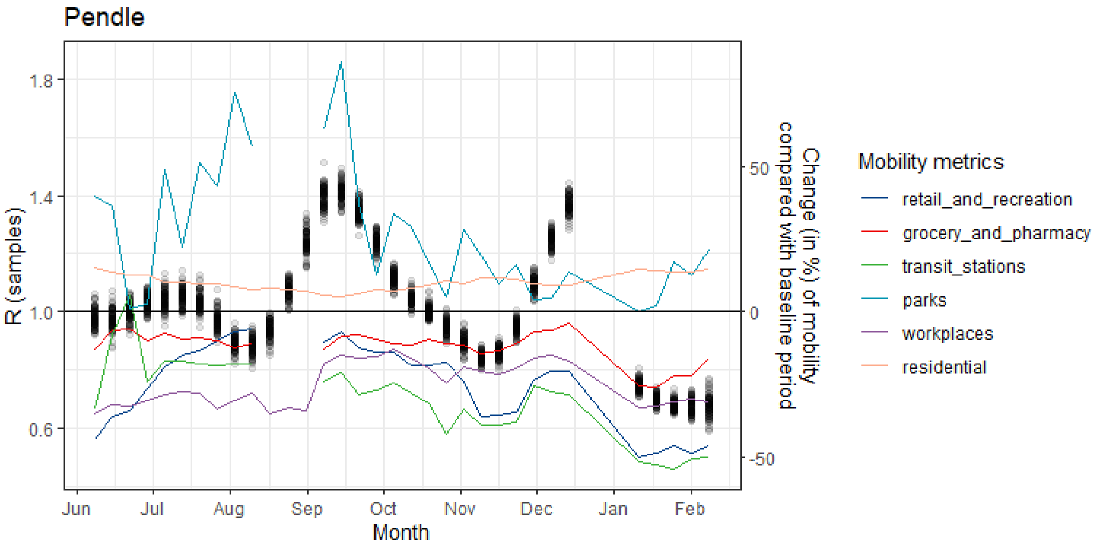
Pendle
R (samples)
1.8
1.4
1.0
0.6
Jun
Jul
Aug
Sep
Oct
Nov
Dec
Jan
Feb
Month
50
0
-50
Change (in %) of mobility compared with baseline period
Mobility metrics
retail_and_recreation
grocery_and_pharmacy
transit_stations
parks
workplaces
residential

Perth and Kinross

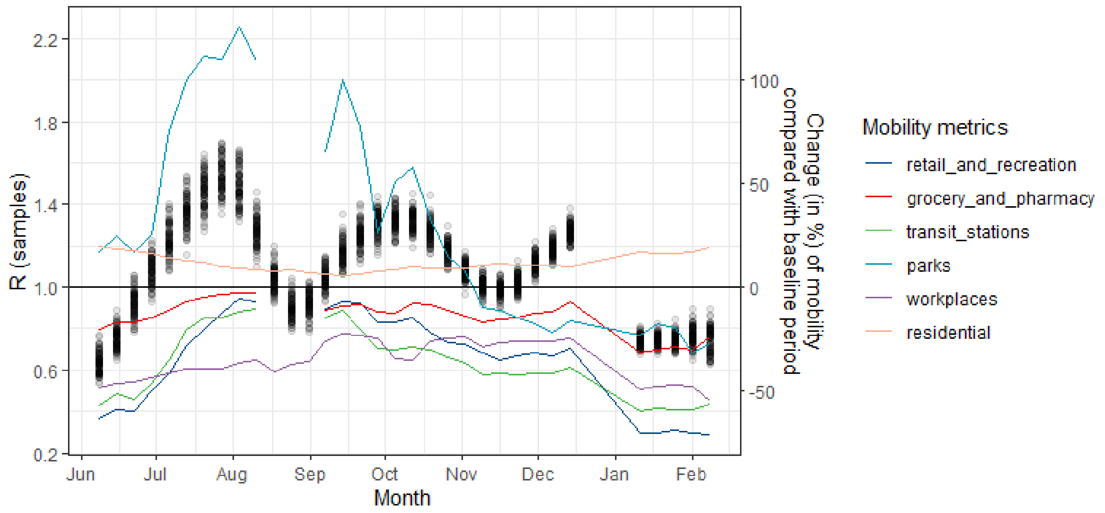

Peterborough

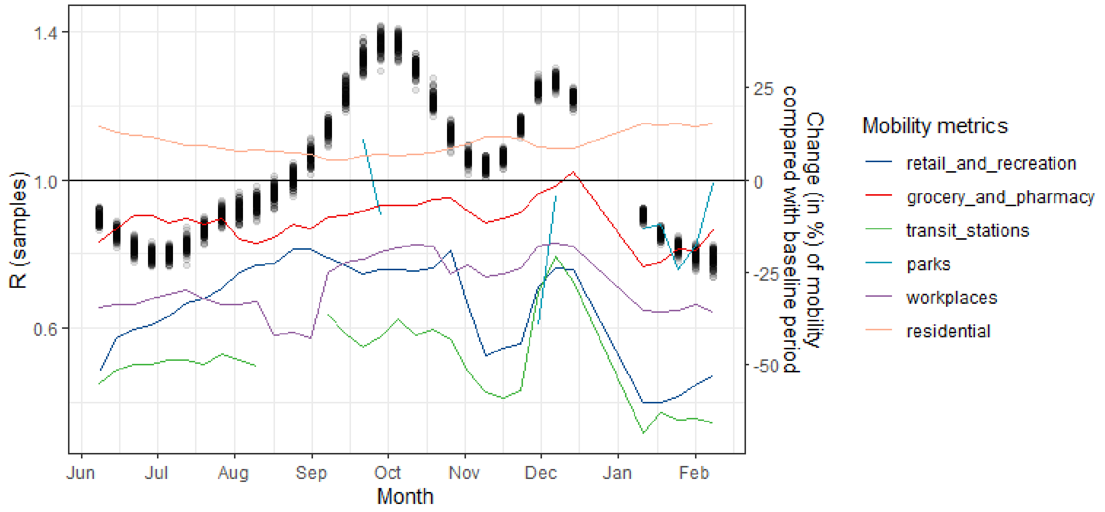

Plymouth

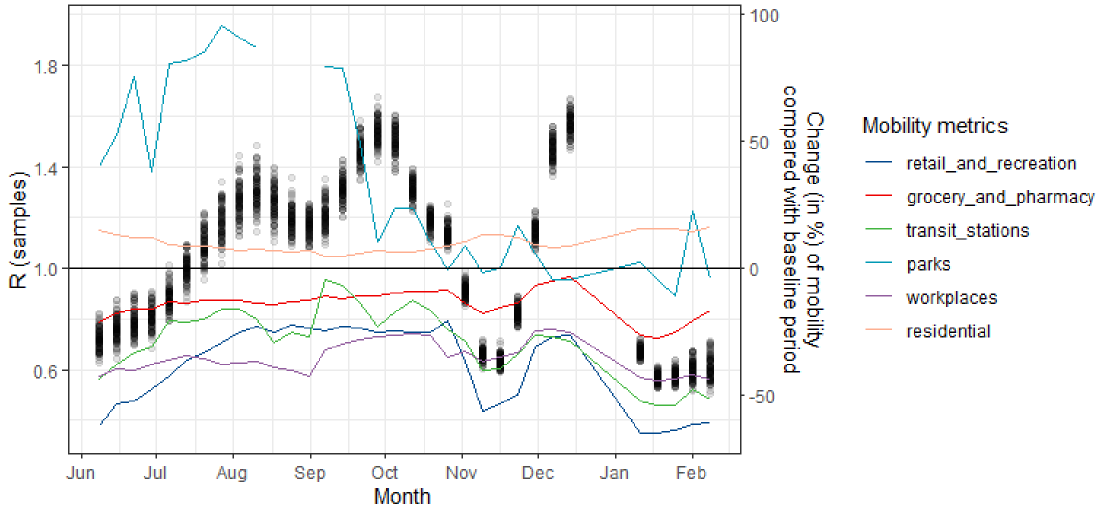

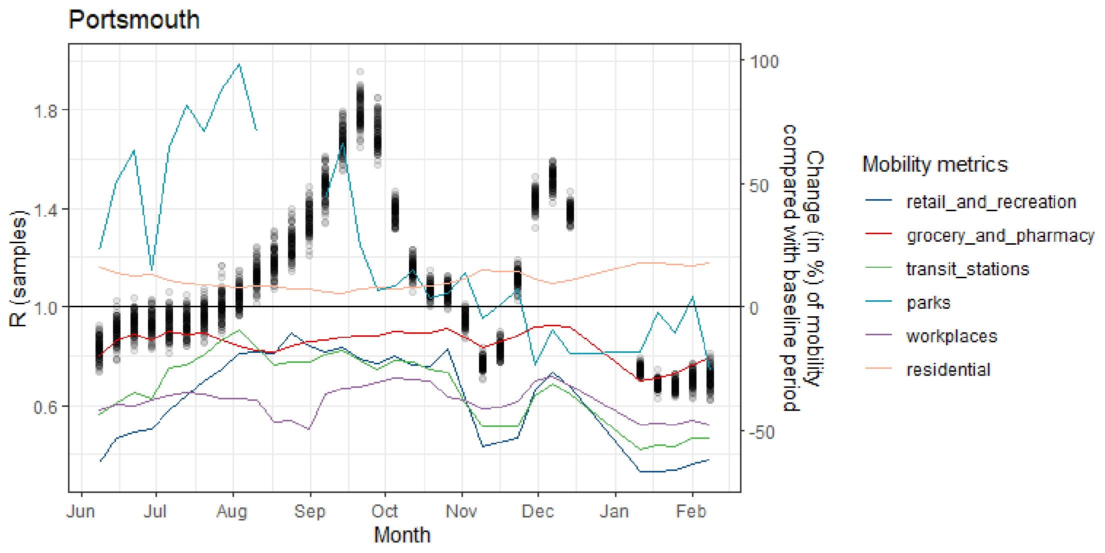
Portsmouth
R (samples)
Change (in %) of mobility compared with baseline period
Month
Jun
Jul
Aug
Sep
Oct
Nov
Dec
Jan
Feb
Mobility metrics
retail_and_recreation
grocery_and_pharmacy
transit_stations
parks
workplaces
residential

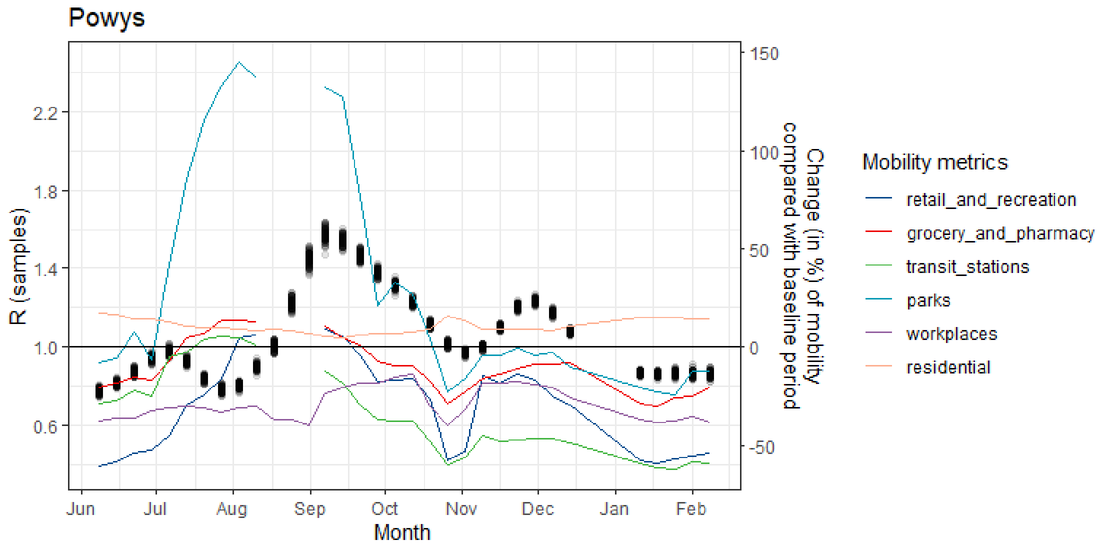
Powys
R (samples)
Change (in %) of mobility compared with baseline period
Month
Jun
Jul
Aug
Sep
Oct
Nov
Dec
Jan
Feb
Mobility metrics
retail_and_recreation
grocery_and_pharmacy
transit_stations
parks
workplaces
residential

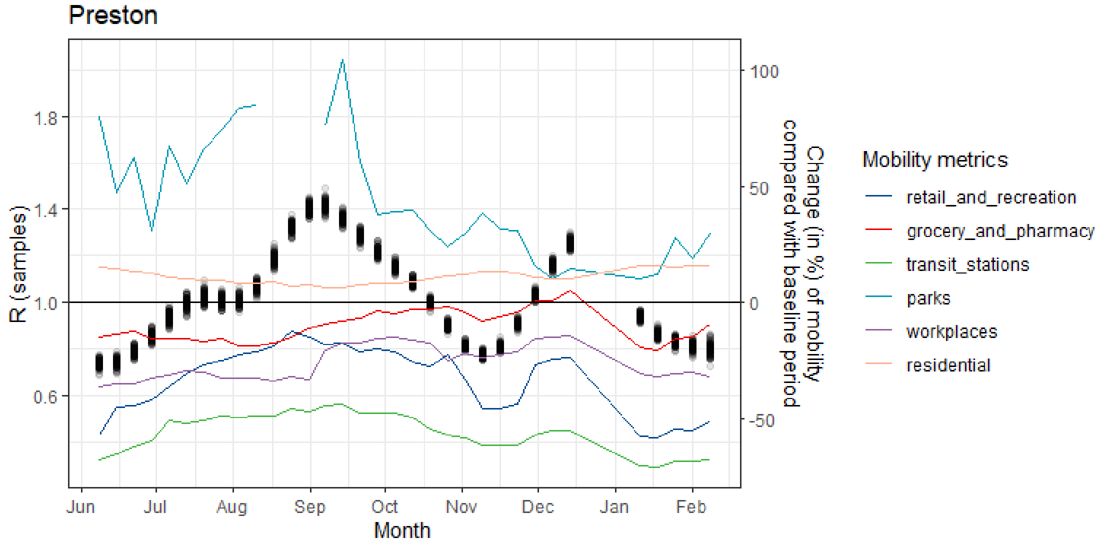
Preston
R (samples)
Change (in %) of mobility compared with baseline period
Month
Jun
Jul
Aug
Sep
Oct
Nov
Dec
Jan
Feb
Mobility metrics
retail_and_recreation
grocery_and_pharmacy
transit_stations
parks
workplaces
residential

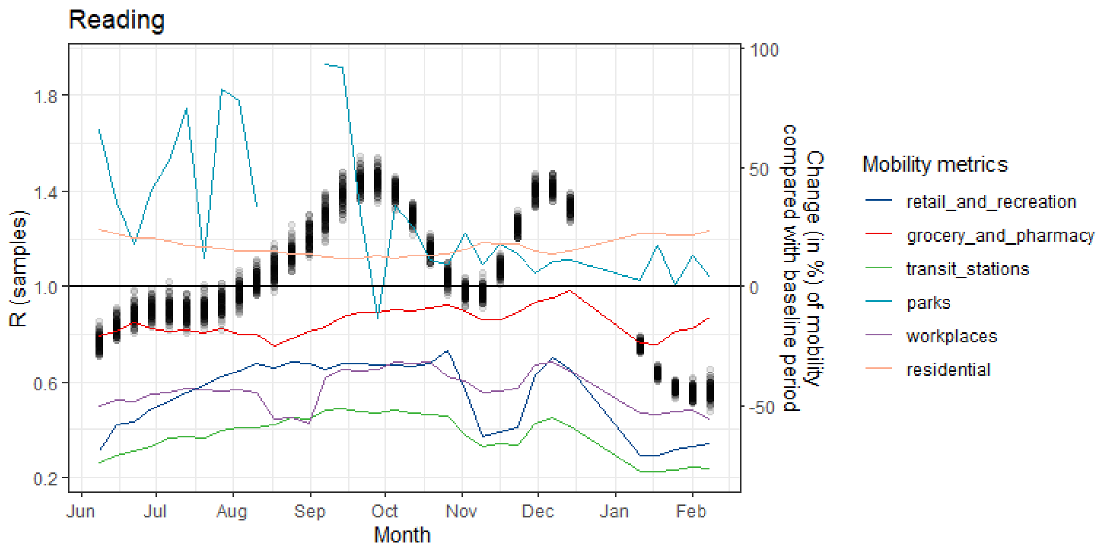
Reading
R (samples)
1.8
1.4
1.0
0.6
0.2
Jun
Jul
Aug
Sep
Oct
Nov
Dec
Jan
Feb
Month
100
50
0
-50
Change (in %) of mobility
compared with baseline period
Mobility metrics
retail_and_recreation
grocery_and_pharmacy
transit_stations
parks
workplaces
residential

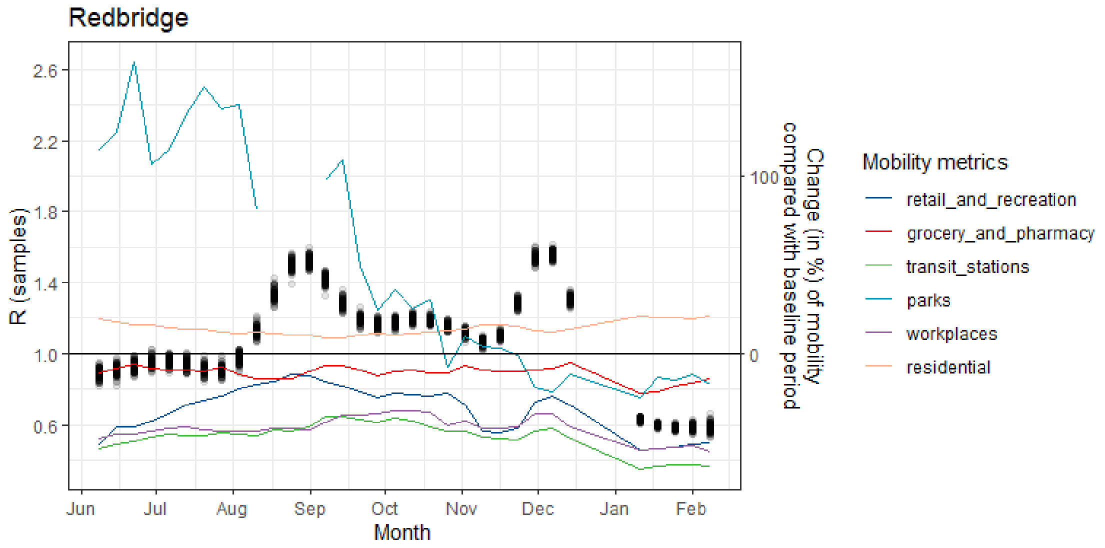
Redbridge
R (samples)
2.6
2.2
1.8
1.4
1.0
0.6
Jun
Jul
Aug
Sep
Oct
Nov
Dec
Jan
Feb
Month
100
0
Change (in %) of mobility
compared with baseline period
Mobility metrics
retail_and_recreation
grocery_and_pharmacy
transit_stations
parks
workplaces
residential

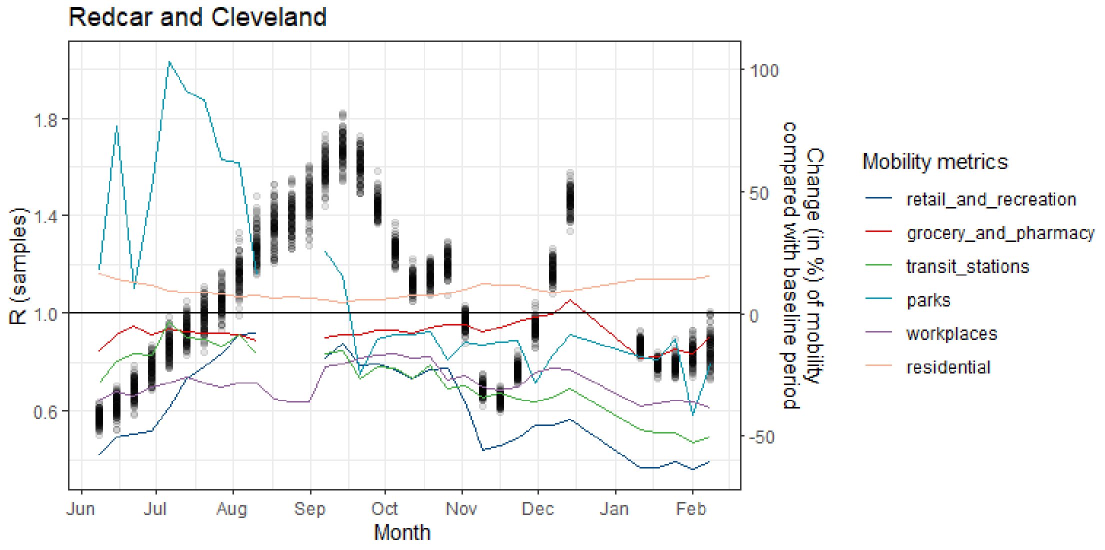
Redcar and Cleveland
R (samples)
1.8
1.4
1.0
0.6
Jun
Jul
Aug
Sep
Oct
Nov
Dec
Jan
Feb
Month
100
50
0
-50
Change (in %) of mobility
compared with baseline period
Mobility metrics
retail_and_recreation
grocery_and_pharmacy
transit_stations
parks
workplaces
residential

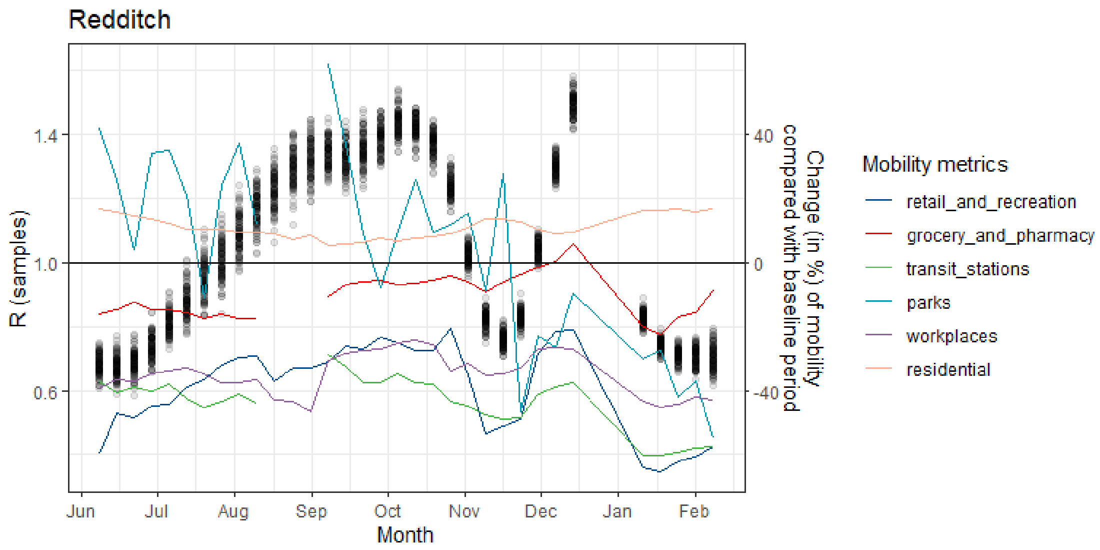
Redditch
R (samples)
1.4
1.0
0.6
40
0
-40
Change (in %) of mobility compared with baseline period
Jun
Jul
Aug
Sep
Oct
Nov
Dec
Jan
Feb
Month
Mobility metrics
retail_and_recreation
grocery_and_pharmacy
transit_stations
parks
workplaces
residential

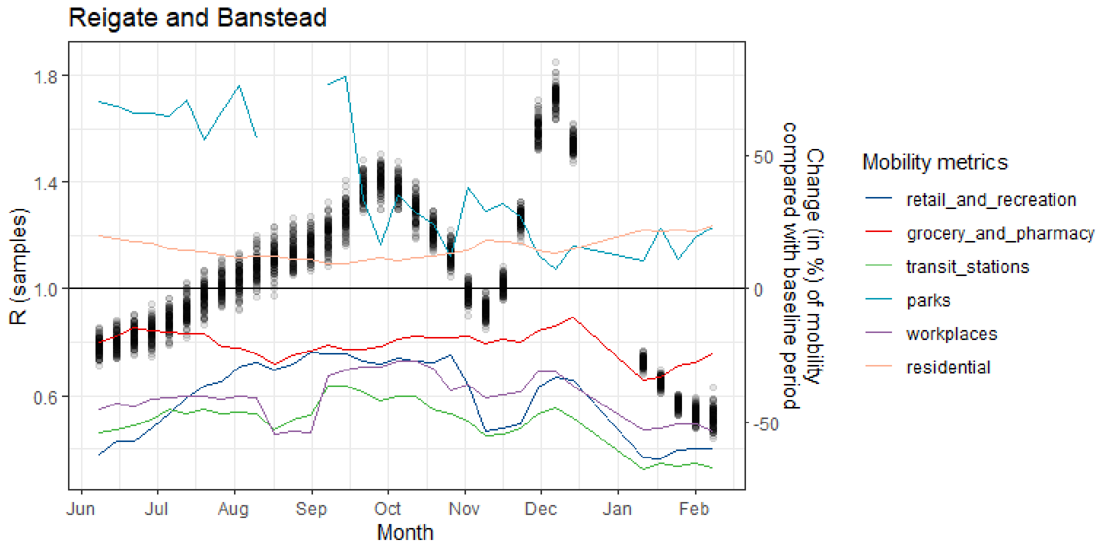
Reigate and Banstead
R (samples)
1.8
1.4
1.0
0.6
50
0
-50
Change (in %) of mobility compared with baseline period
Jun
Jul
Aug
Sep
Oct
Nov
Dec
Jan
Feb
Month
Mobility metrics
retail_and_recreation
grocery_and_pharmacy
transit_stations
parks
workplaces
residential

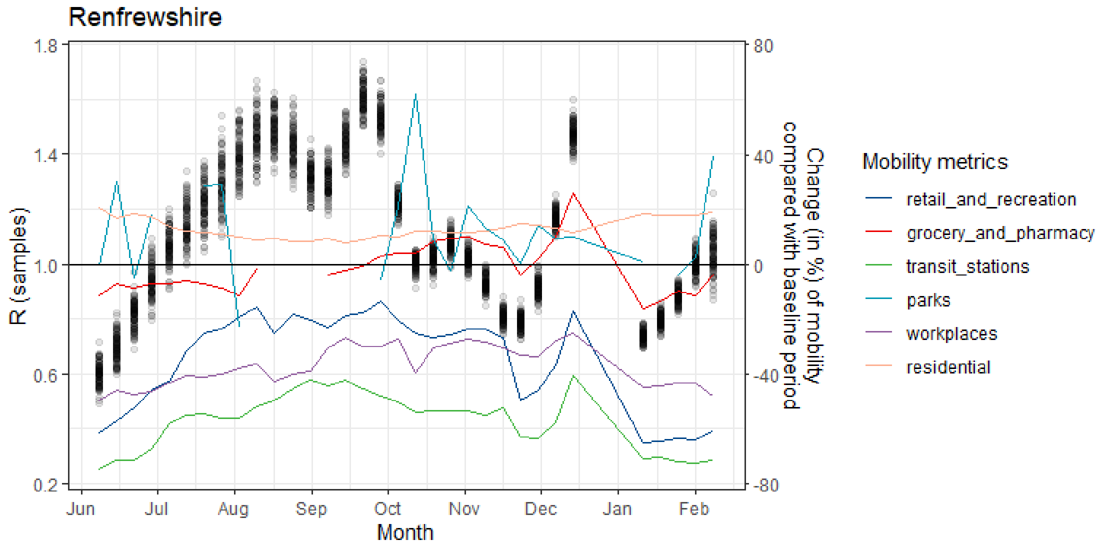
Renfrewshire
R (samples)
1.8
1.4
1.0
0.6
0.2
80
40
0
-40
-80
Change (in %) of mobility compared with baseline period
Jun
Jul
Aug
Sep
Oct
Nov
Dec
Jan
Feb
Month
Mobility metrics
retail_and_recreation
grocery_and_pharmacy
transit_stations
parks
workplaces
residential

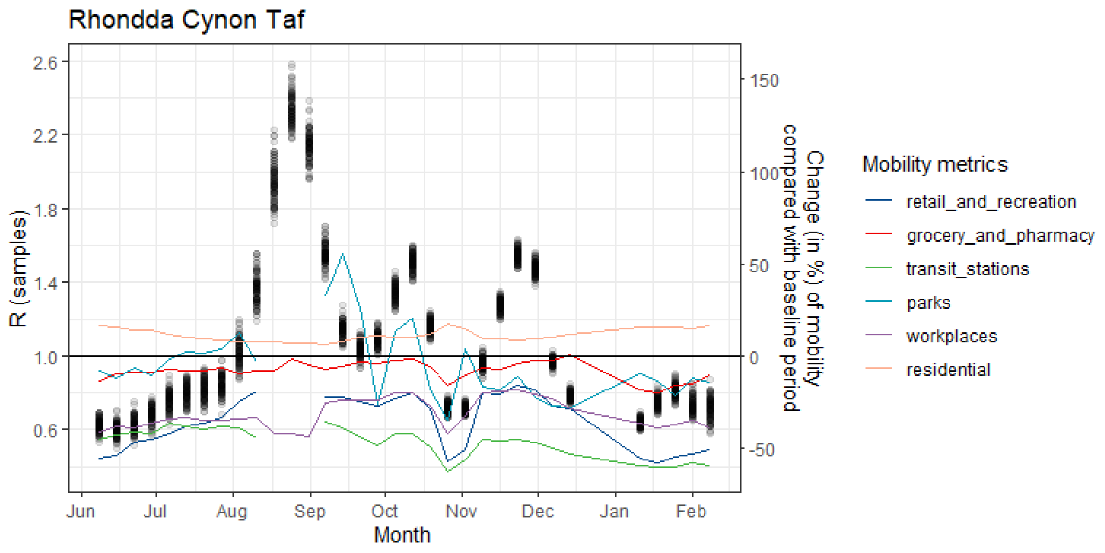

Rhondda Cynon Taf
R (samples)
2.6
2.2
1.8
1.4
1.0
0.6
150
100
50
0
-50
Change (in %) of mobility compared with baseline period
Jun
Jul
Aug
Sep
Oct
Nov
Dec
Jan
Feb
Month
Mobility metrics
retail_and_recreation
grocery_and_pharmacy
transit_stations
parks
workplaces
residential

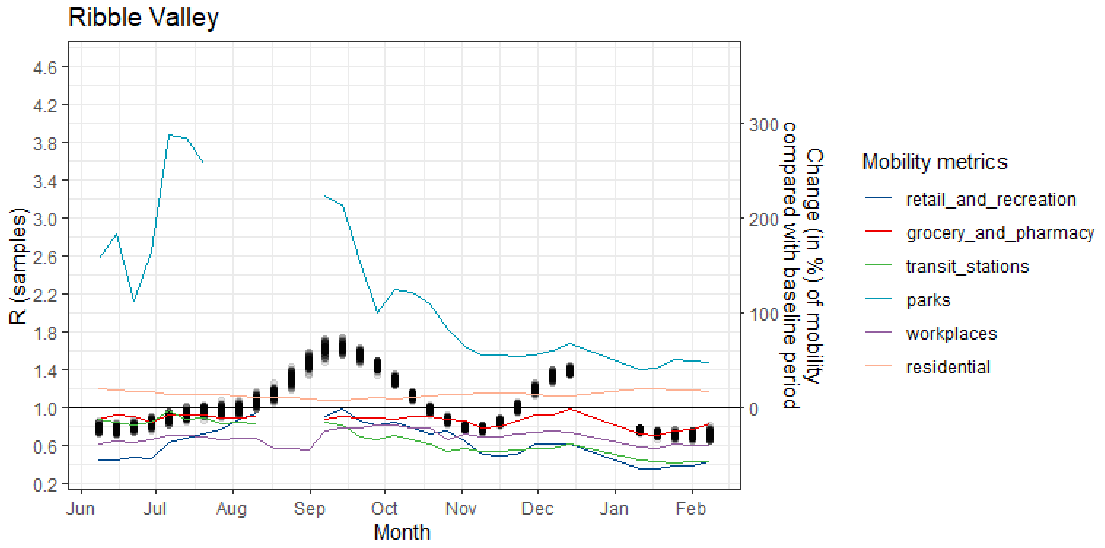

Ribble Valley
R (samples)
4.6
4.2
3.8
3.4
3.0
2.6
2.2
1.8
1.4
1.0
0.6
0.2
300
200
100
0
Change (in %) of mobility compared with baseline period
Jun
Jul
Aug
Sep
Oct
Nov
Dec
Jan
Feb
Month
Mobility metrics
retail_and_recreation
grocery_and_pharmacy
transit_stations
parks
workplaces
residential

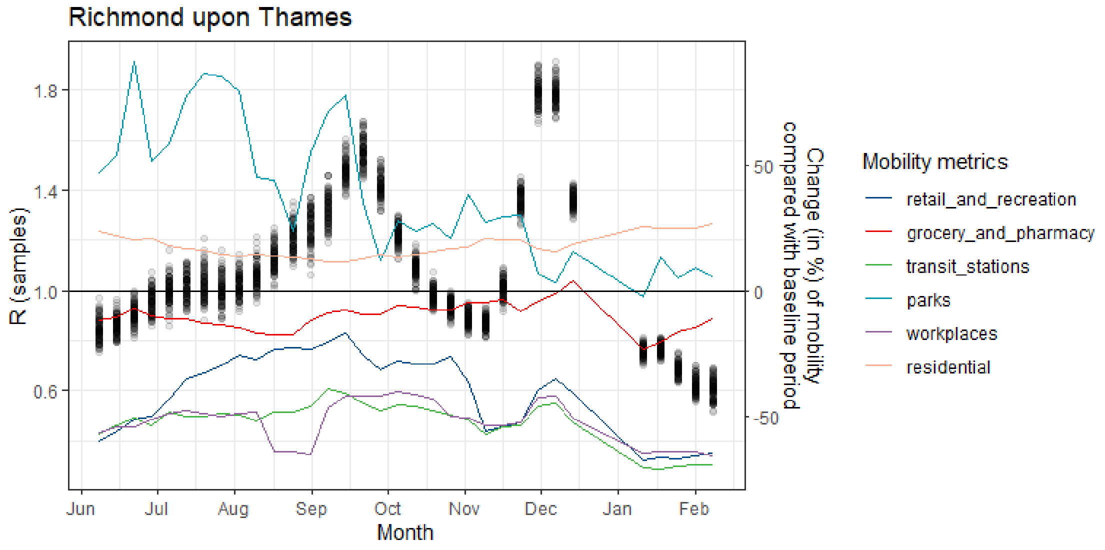

Richmond upon Thames
R (samples)
1.8
1.4
1.0
0.6
50
0
-50
Change (in %) of mobility compared with baseline period
Jun
Jul
Aug
Sep
Oct
Nov
Dec
Jan
Feb
Month
Mobility metrics
retail_and_recreation
grocery_and_pharmacy
transit_stations
parks
workplaces
residential

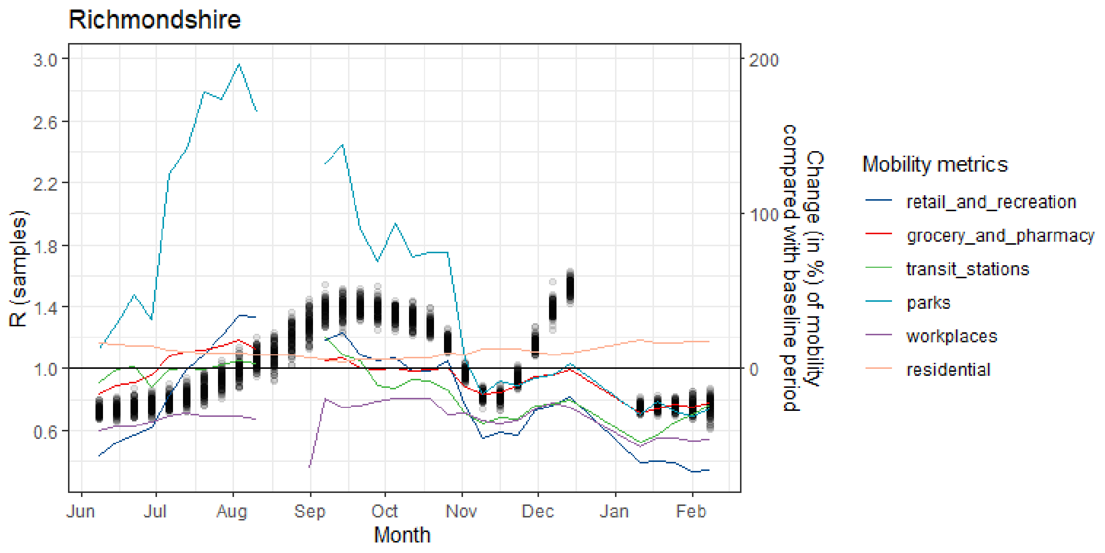
Richmondshire
R (samples)
Change (in %) of mobility compared with baseline period
Month
Jun
Jul
Aug
Sep
Oct
Nov
Dec
Jan
Feb
Mobility metrics
retail_and_recreation
grocery_and_pharmacy
transit_stations
parks
workplaces
residential

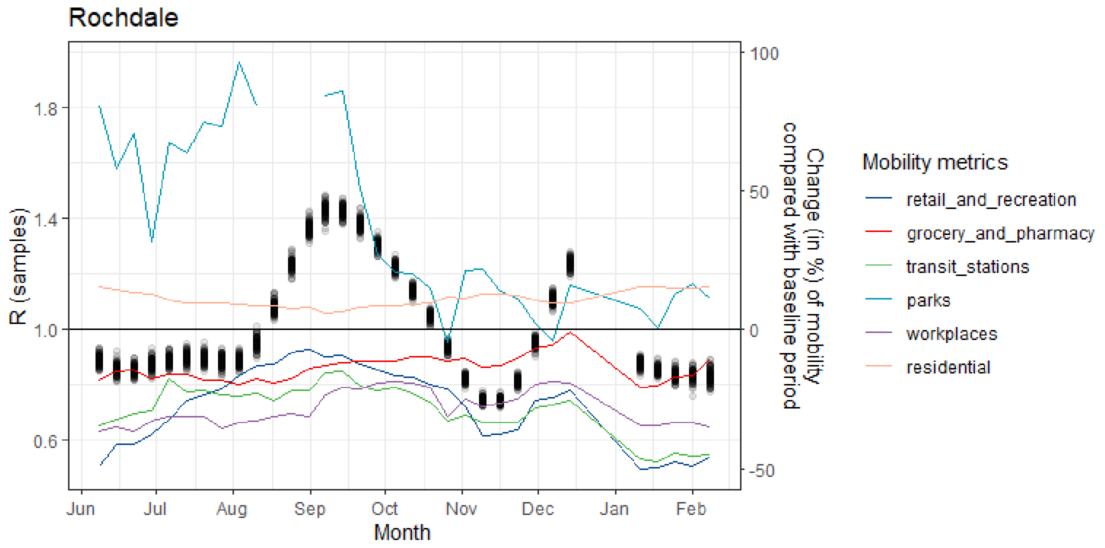
Rochdale
R (samples)
Change (in %) of mobility compared with baseline period
Month
Jun
Jul
Aug
Sep
Oct
Nov
Dec
Jan
Feb
Mobility metrics
retail_and_recreation
grocery_and_pharmacy
transit_stations
parks
workplaces
residential

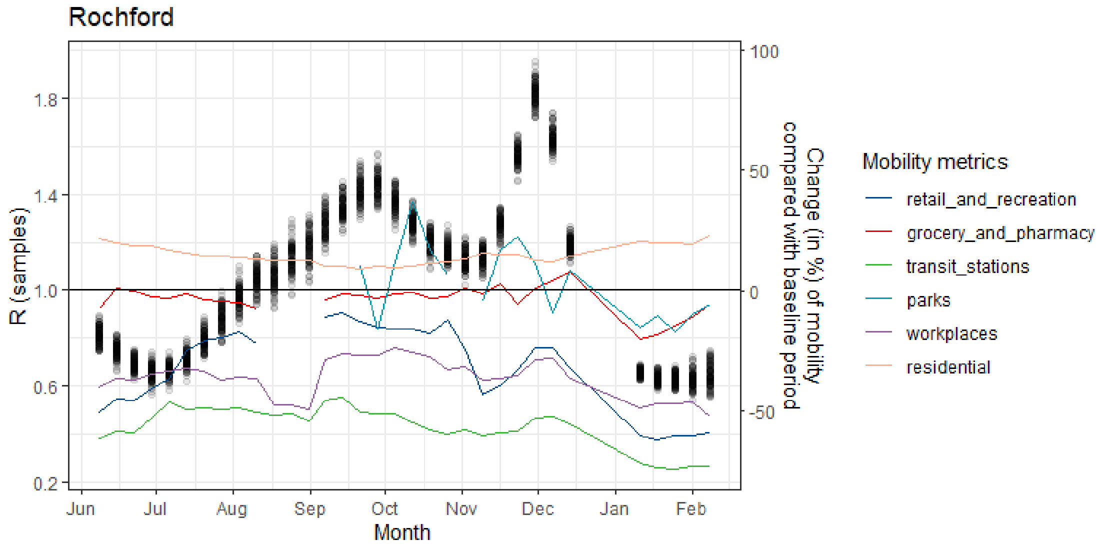
Rochford
R (samples)
Change (in %) of mobility compared with baseline period
Month
Jun
Jul
Aug
Sep
Oct
Nov
Dec
Jan
Feb
Mobility metrics
retail_and_recreation
grocery_and_pharmacy
transit_stations
parks
workplaces
residential

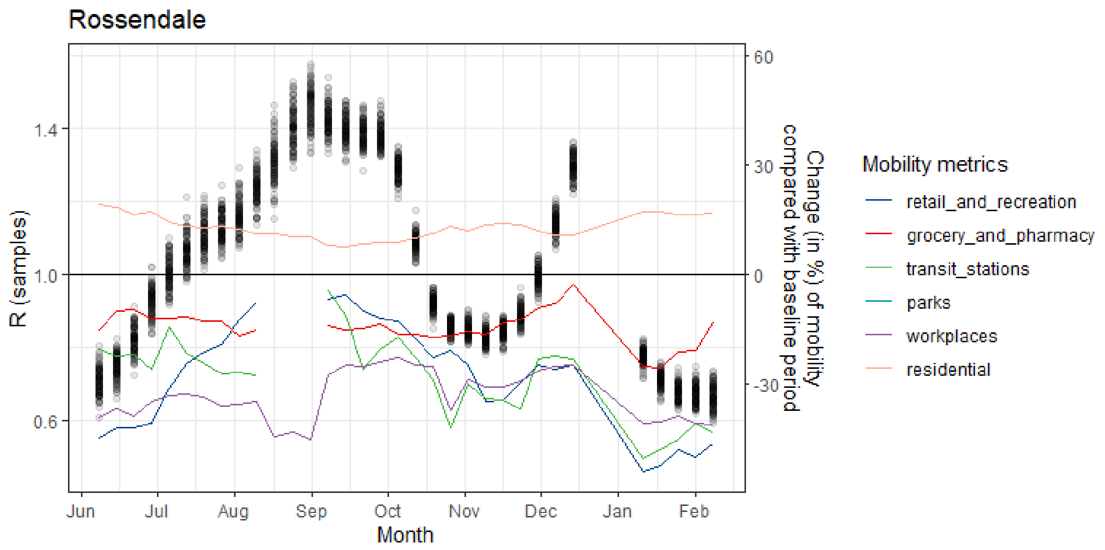
Rossendale
R (samples)
1.4
1.0
0.6
60
30
0
-30
Change (in %) of mobility compared with baseline period
Jun
Jul
Aug
Sep
Oct
Nov
Dec
Jan
Feb
Month
Mobility metrics
retail_and_recreation
grocery_and_pharmacy
transit_stations
parks
workplaces
residential

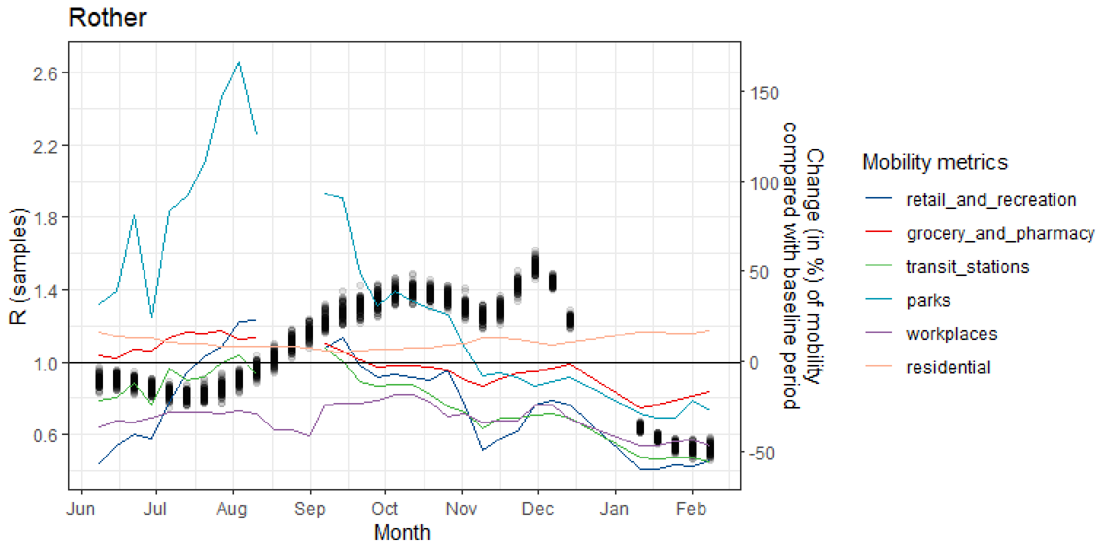
Rother
R (samples)
2.6
2.2
1.8
1.4
1.0
0.6
150
100
50
0
-50
Change (in %) of mobility compared with baseline period
Jun
Jul
Aug
Sep
Oct
Nov
Dec
Jan
Feb
Month
Mobility metrics
retail_and_recreation
grocery_and_pharmacy
transit_stations
parks
workplaces
residential

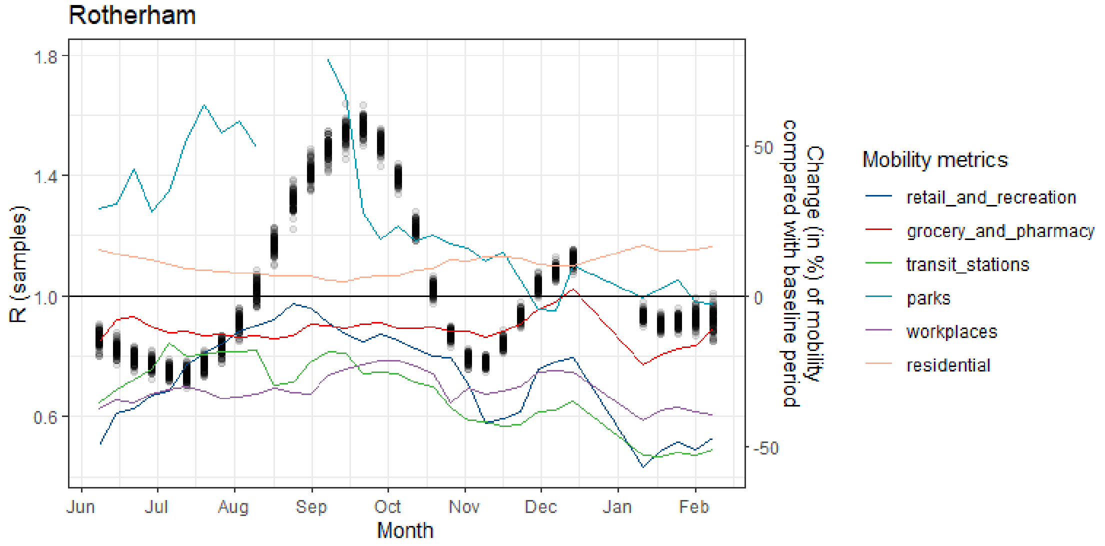
Rotherham
R (samples)
1.8
1.4
1.0
0.6
50
0
-50
Change (in %) of mobility compared with baseline period
Jun
Jul
Aug
Sep
Oct
Nov
Dec
Jan
Feb
Month
Mobility metrics
retail_and_recreation
grocery_and_pharmacy
transit_stations
parks
workplaces
residential

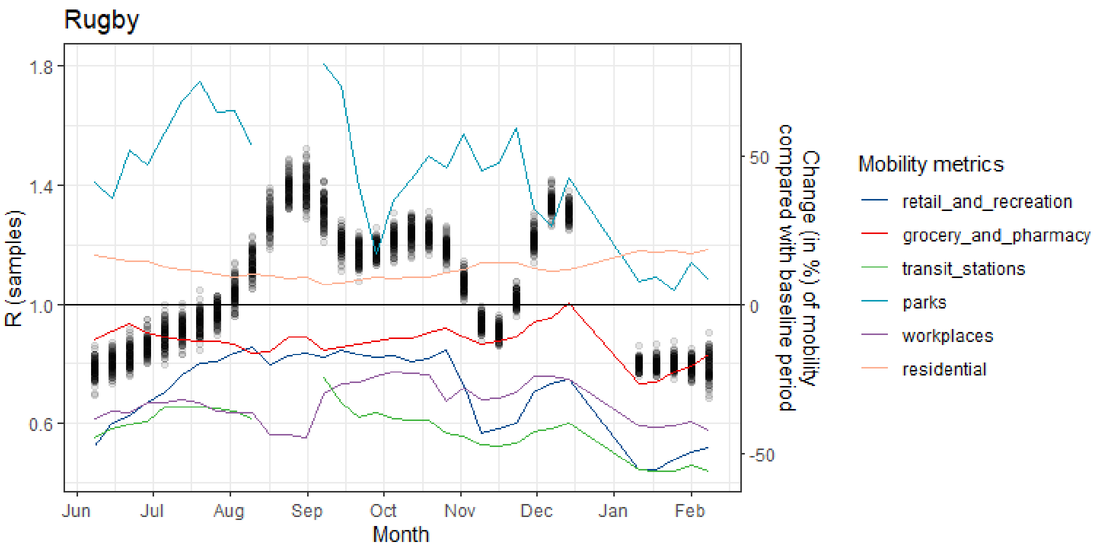
Rugby
R (samples)
1.8
1.4
1.0
0.6
Jun Jul Aug Sep Oct Nov Dec Jan Feb
Month
50
0
-50
Change (in %) of mobility compared with baseline period
Mobility metrics
retail_and_recreation
grocery_and_pharmacy
transit_stations
parks
workplaces
residential

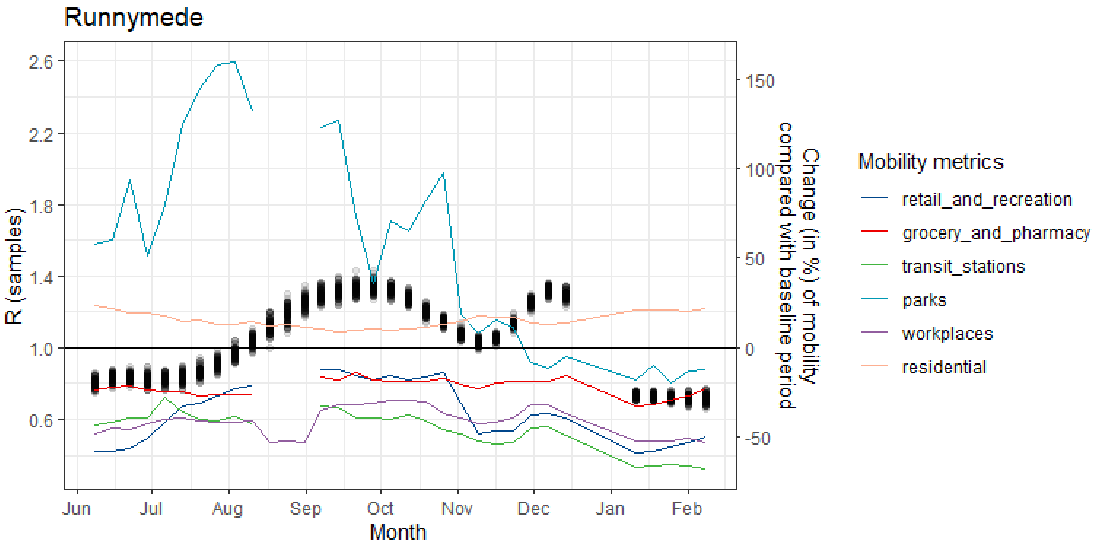
Runnymede
R (samples)
2.6
2.2
1.8
1.4
1.0
0.6
Jun Jul Aug Sep Oct Nov Dec Jan Feb
Month
150
100
50
0
-50
Change (in %) of mobility compared with baseline period
Mobility metrics
retail_and_recreation
grocery_and_pharmacy
transit_stations
parks
workplaces
residential

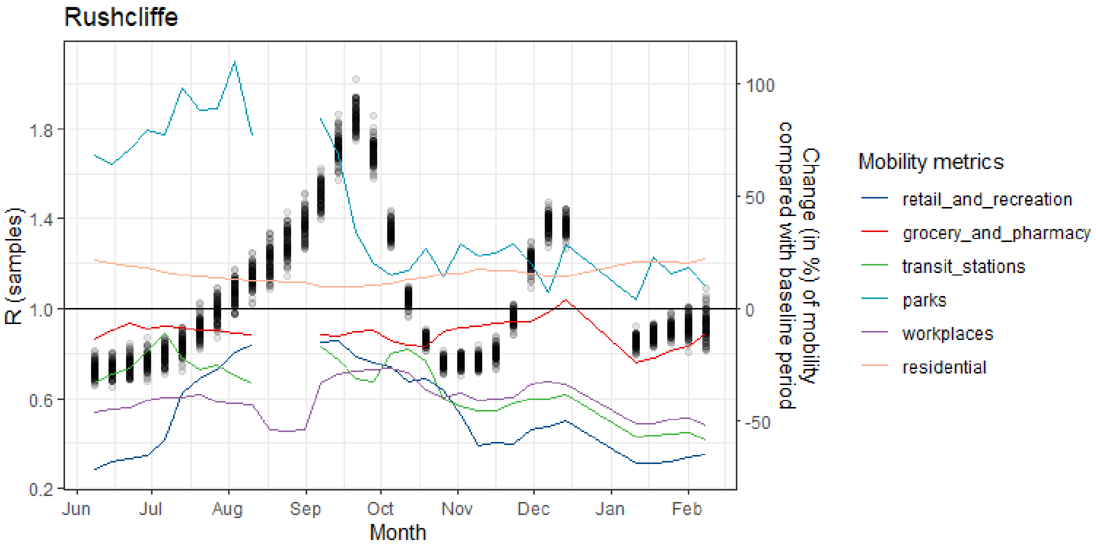
Rushcliffe
R (samples)
1.8
1.4
1.0
0.6
0.2
Jun Jul Aug Sep Oct Nov Dec Jan Feb
Month
100
50
0
-50
Change (in %) of mobility compared with baseline period
Mobility metrics
retail_and_recreation
grocery_and_pharmacy
transit_stations
parks
workplaces
residential

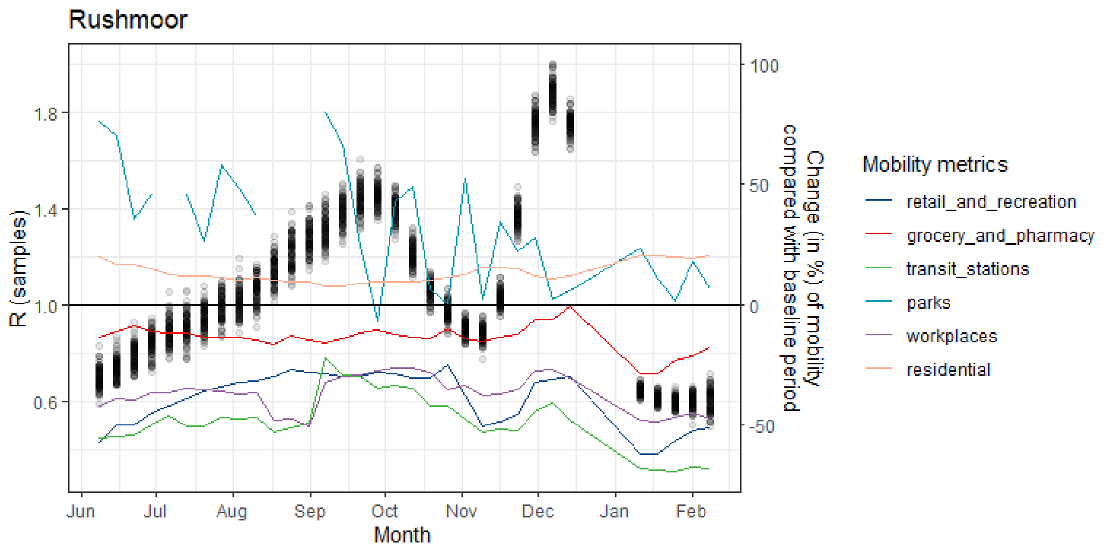
Rushmoor
R (samples)
1.8
1.4
1.0
0.6
Change (in %) of mobility compared with baseline period
100
50
0
-50
Jun
Jul
Aug
Sep
Oct
Nov
Dec
Jan
Feb
Month
Mobility metrics
retail_and_recreation
grocery_and_pharmacy
transit_stations
parks
workplaces
residential

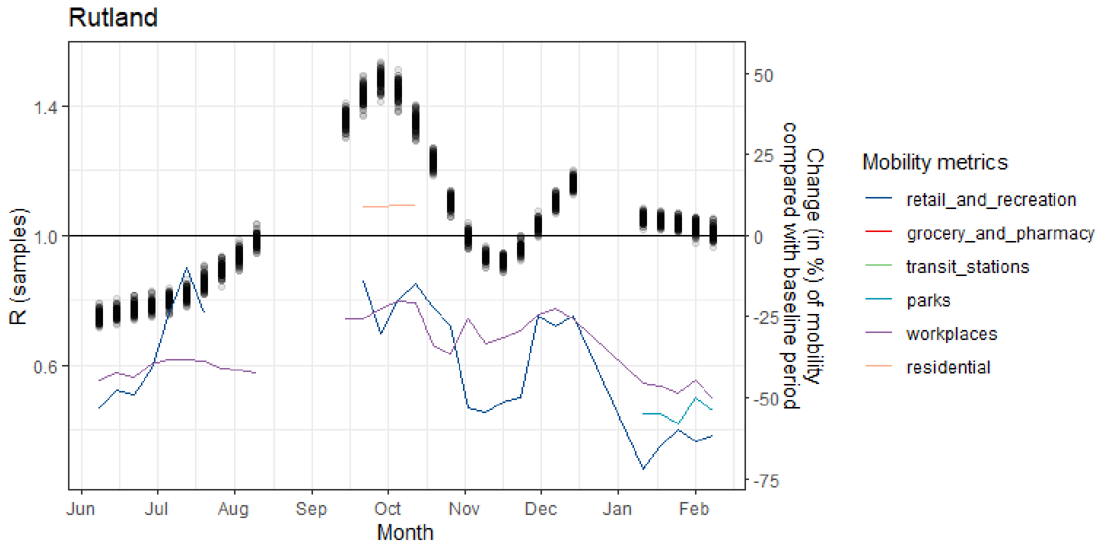
Rutland
R (samples)
1.4
1.0
0.6
Change (in %) of mobility compared with baseline period
50
25
0
-25
-50
-75
Jun
Jul
Aug
Sep
Oct
Nov
Dec
Jan
Feb
Month
Mobility metrics
retail_and_recreation
grocery_and_pharmacy
transit_stations
parks
workplaces
residential

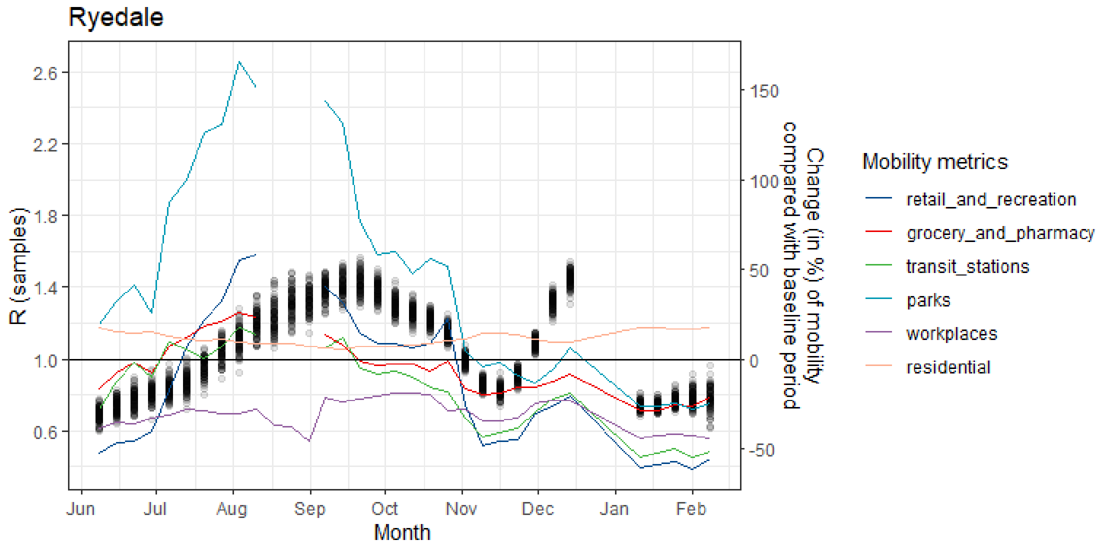
Ryedale
R (samples)
2.6
2.2
1.8
1.4
1.0
0.6
Change (in %) of mobility compared with baseline period
150
100
50
0
-50
Jun
Jul
Aug
Sep
Oct
Nov
Dec
Jan
Feb
Month
Mobility metrics
retail_and_recreation
grocery_and_pharmacy
transit_stations
parks
workplaces
residential

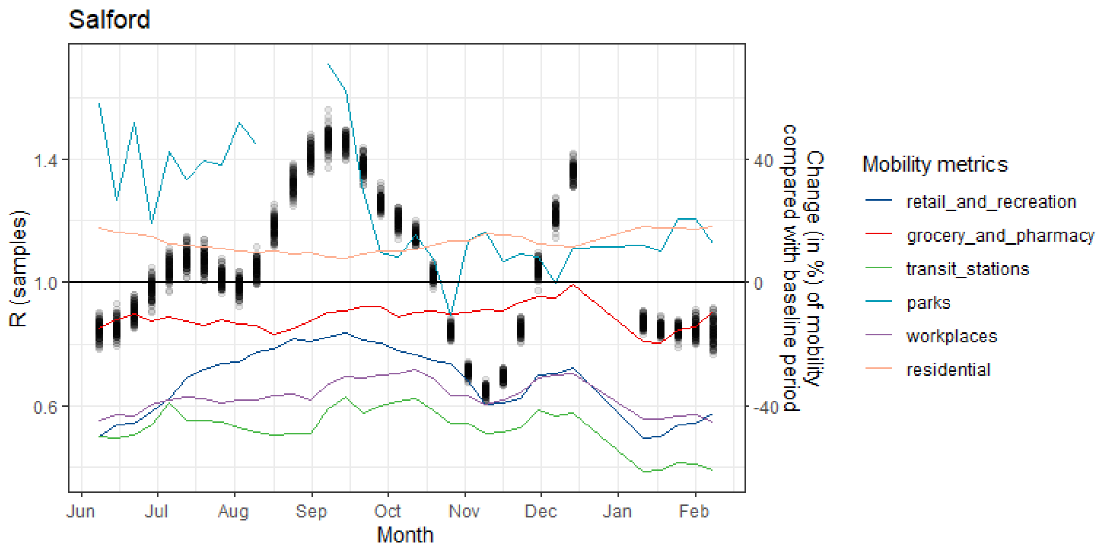
Salford
R (samples)
1.4
1.0
0.6
Jun
Jul
Aug
Sep
Oct
Nov
Dec
Jan
Feb
Month
40
0
-40
Change (in %) of mobility compared with baseline period
Mobility metrics
retail_and_recreation
grocery_and_pharmacy
transit_stations
parks
workplaces
residential

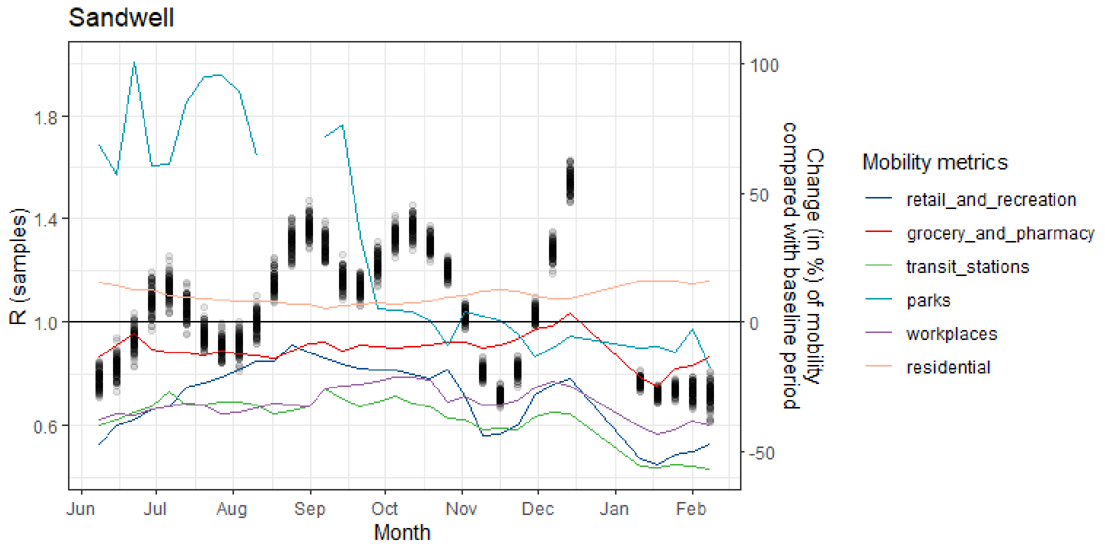
Sandwell
R (samples)
1.8
1.4
1.0
0.6
Jun
Jul
Aug
Sep
Oct
Nov
Dec
Jan
Feb
Month
100
50
0
-50
Change (in %) of mobility compared with baseline period
Mobility metrics
retail_and_recreation
grocery_and_pharmacy
transit_stations
parks
workplaces
residential

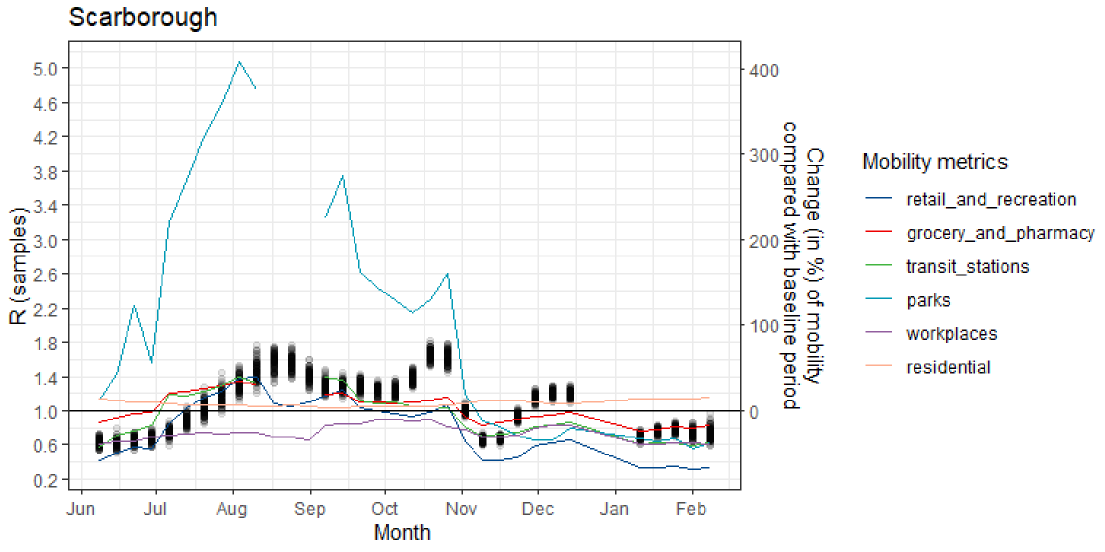
Scarborough
R (samples)
5.0
4.6
4.2
3.8
3.4
3.0
2.6
2.2
1.8
1.4
1.0
0.6
0.2
Jun
Jul
Aug
Sep
Oct
Nov
Dec
Jan
Feb
Month
400
300
200
100
0
Change (in %) of mobility compared with baseline period
Mobility metrics
retail_and_recreation
grocery_and_pharmacy
transit_stations
parks
workplaces
residential

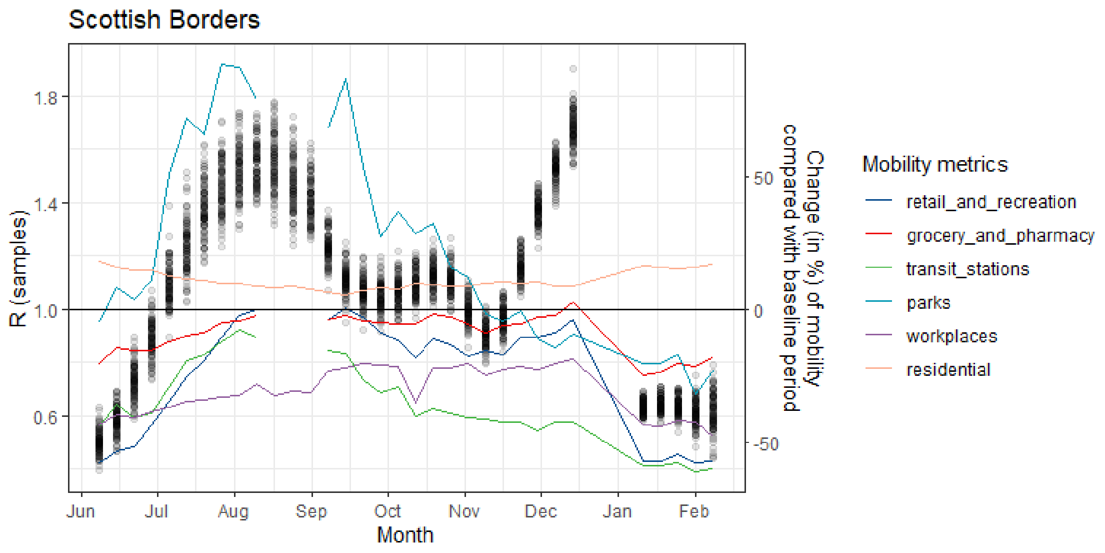
Scottish Borders
R (samples)
1.8
1.4
1.0
0.6
Jun
Jul
Aug
Sep
Oct
Nov
Dec
Jan
Feb
Month
50
0
-50
Change (in %) of mobility
compared with baseline period
Mobility metrics
retail_and_recreation
grocery_and_pharmacy
transit_stations
parks
workplaces
residential

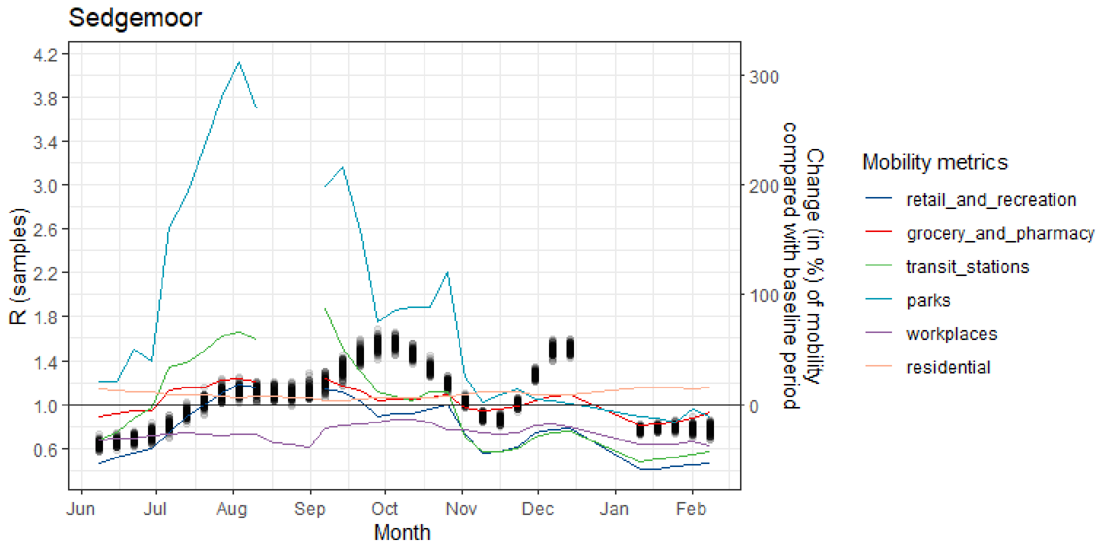
Sedgemoor
R (samples)
4.2
3.8
3.4
3.0
2.6
2.2
1.8
1.4
1.0
0.6
Jun
Jul
Aug
Sep
Oct
Nov
Dec
Jan
Feb
Month
300
200
100
0
Change (in %) of mobility
compared with baseline period
Mobility metrics
retail_and_recreation
grocery_and_pharmacy
transit_stations
parks
workplaces
residential

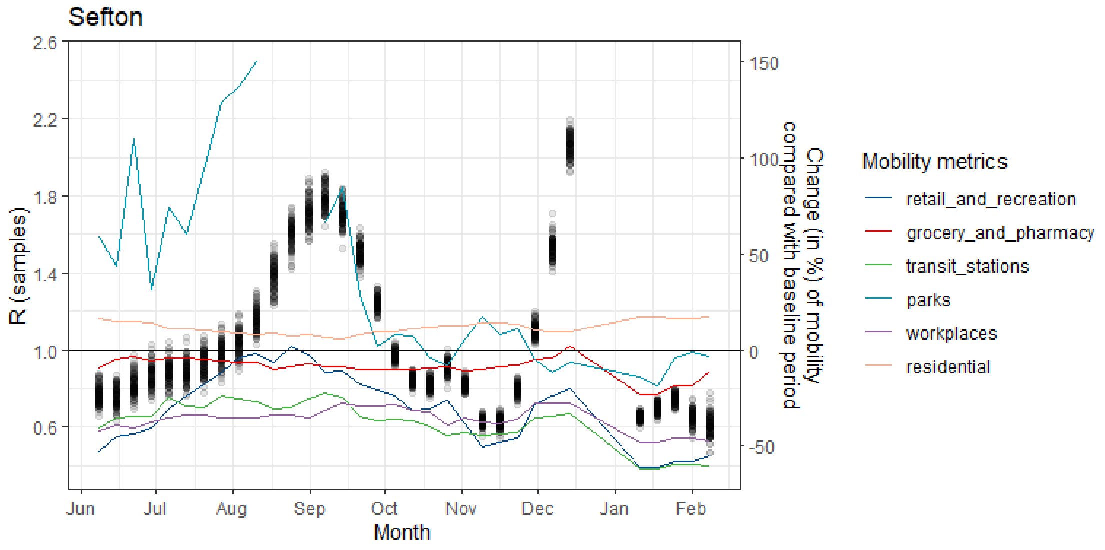
Sefton
R (samples)
2.6
2.2
1.8
1.4
1.0
0.6
Jun
Jul
Aug
Sep
Oct
Nov
Dec
Jan
Feb
Month
150
100
50
0
-50
Change (in %) of mobility
compared with baseline period
Mobility metrics
retail_and_recreation
grocery_and_pharmacy
transit_stations
parks
workplaces
residential

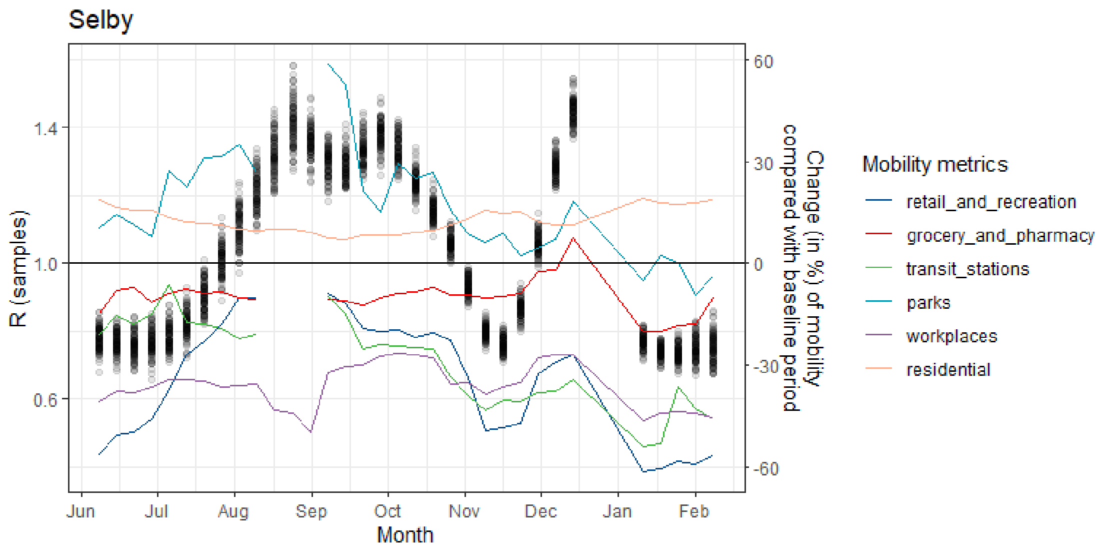
Selby
R (samples)
1.4
1.0
0.6
Jun
Jul
Aug
Sep
Oct
Nov
Dec
Jan
Feb
Month
60
30
0
-30
-60
Change (in %) of mobility
compared with baseline period
Mobility metrics
retail_and_recreation
grocery_and_pharmacy
transit_stations
parks
workplaces
residential

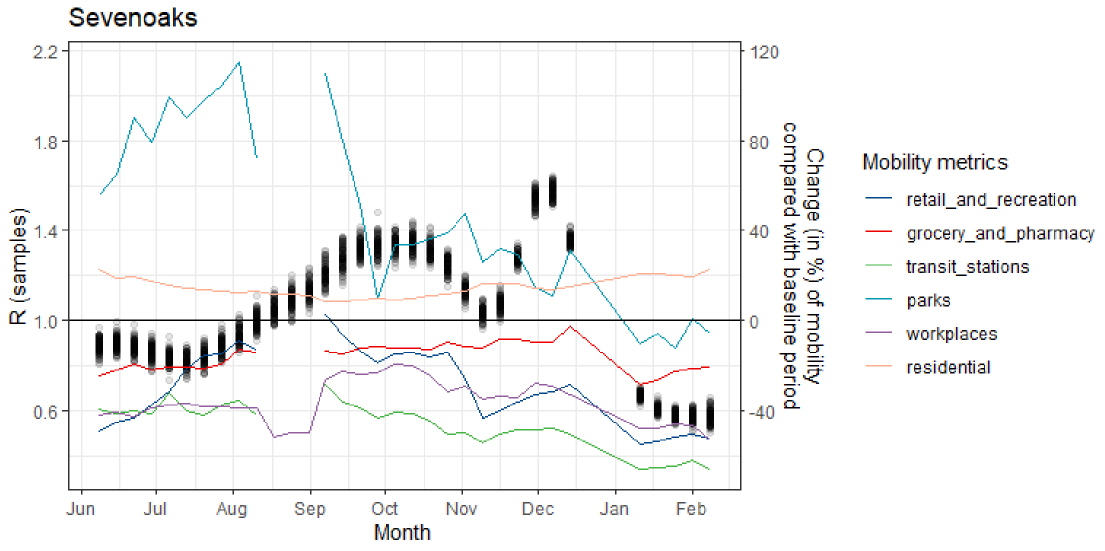
Sevenoaks
R (samples)
2.2
1.8
1.4
1.0
0.6
Jun
Jul
Aug
Sep
Oct
Nov
Dec
Jan
Feb
Month
120
80
40
0
-40
Change (in %) of mobility
compared with baseline period
Mobility metrics
retail_and_recreation
grocery_and_pharmacy
transit_stations
parks
workplaces
residential

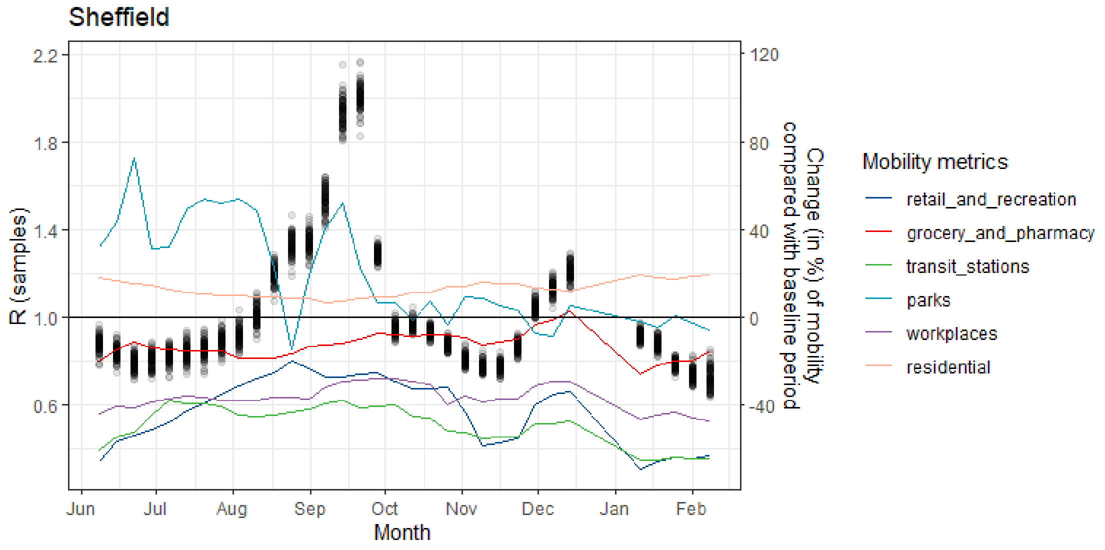
Sheffield
R (samples)
2.2
1.8
1.4
1.0
0.6
Jun
Jul
Aug
Sep
Oct
Nov
Dec
Jan
Feb
Month
120
80
40
0
-40
Change (in %) of mobility
compared with baseline period
Mobility metrics
retail_and_recreation
grocery_and_pharmacy
transit_stations
parks
workplaces
residential

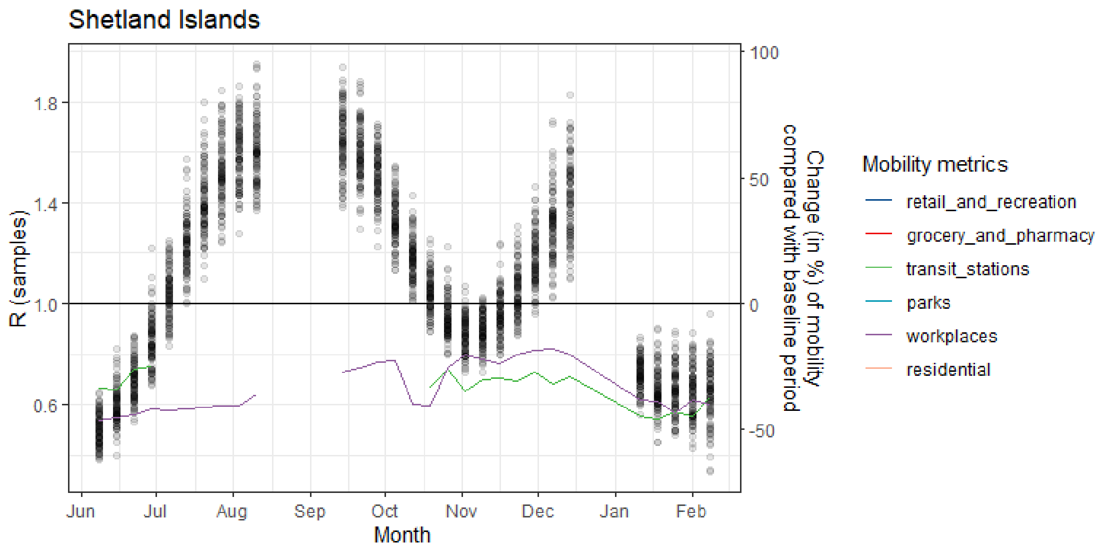
Shetland Islands
R (samples)
Month
Change (in %) of mobility compared with baseline period
Mobility metrics
retail_and_recreation
grocery_and_pharmacy
transit_stations
parks
workplaces
residential

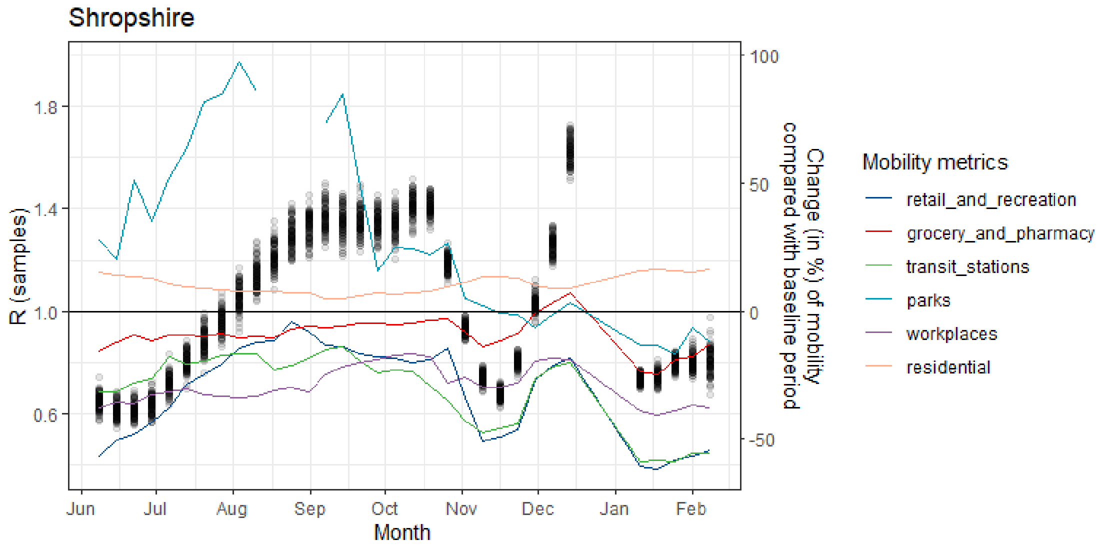
Shropshire
R (samples)
Month
Change (in %) of mobility compared with baseline period
Mobility metrics
retail_and_recreation
grocery_and_pharmacy
transit_stations
parks
workplaces
residential

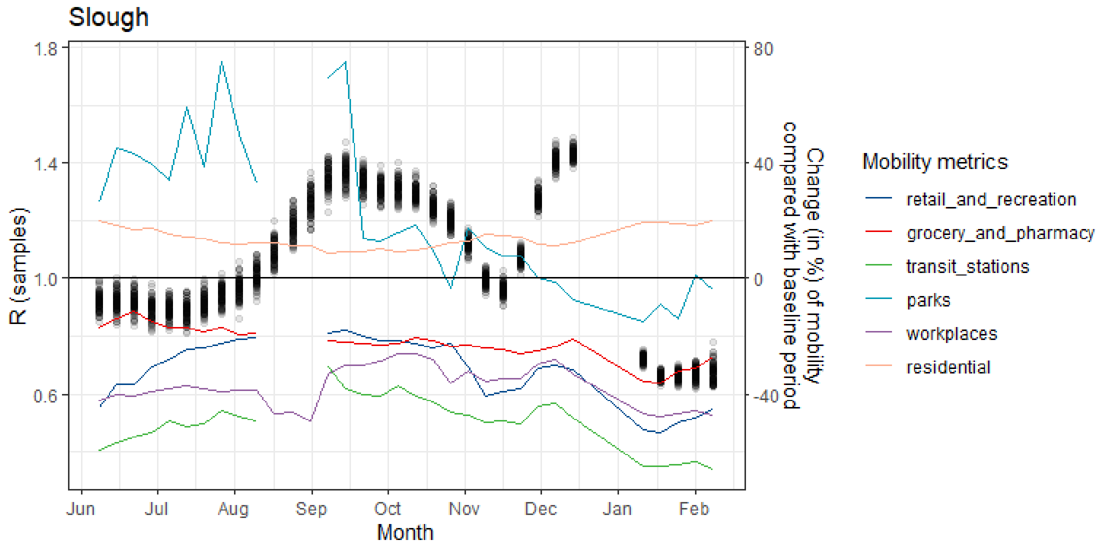
Slough
R (samples)
Month
Change (in %) of mobility compared with baseline period
Mobility metrics
retail_and_recreation
grocery_and_pharmacy
transit_stations
parks
workplaces
residential

Solihull

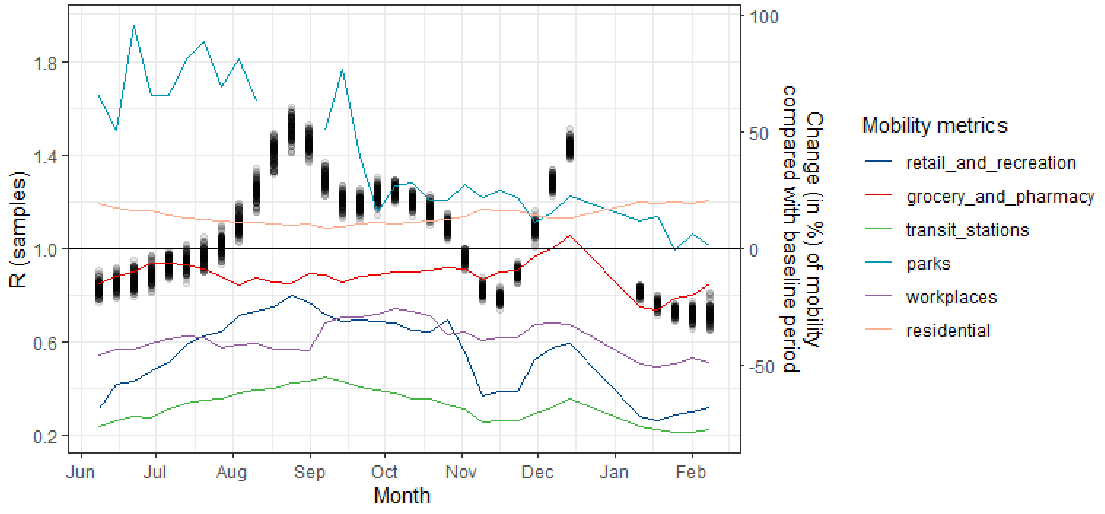

Somerset West and Taunton

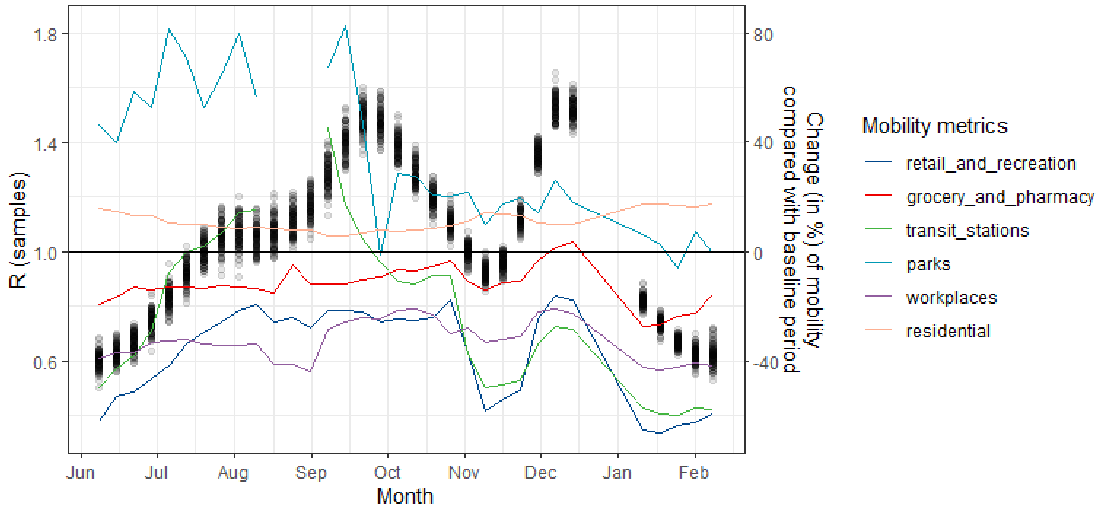

South Ayrshire

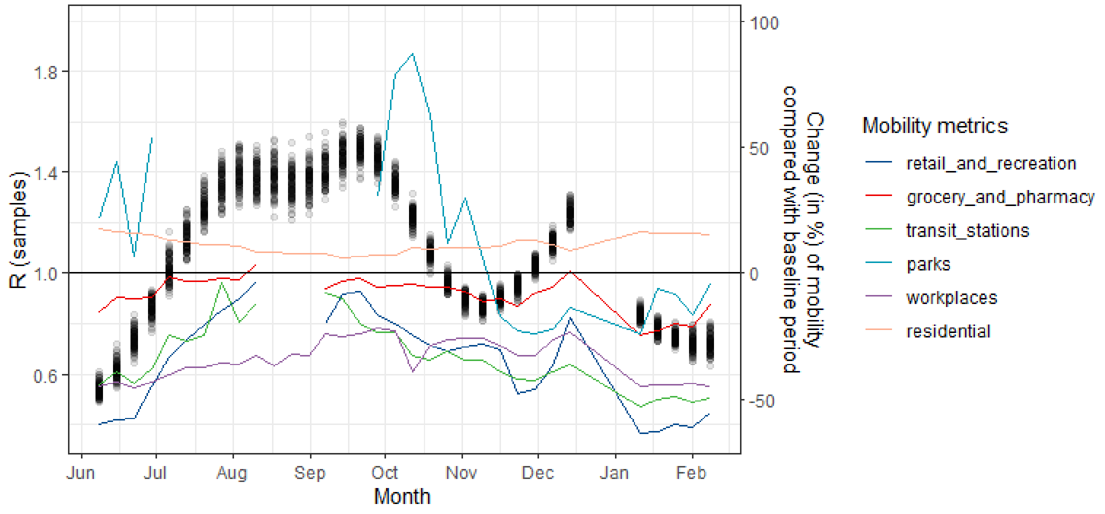

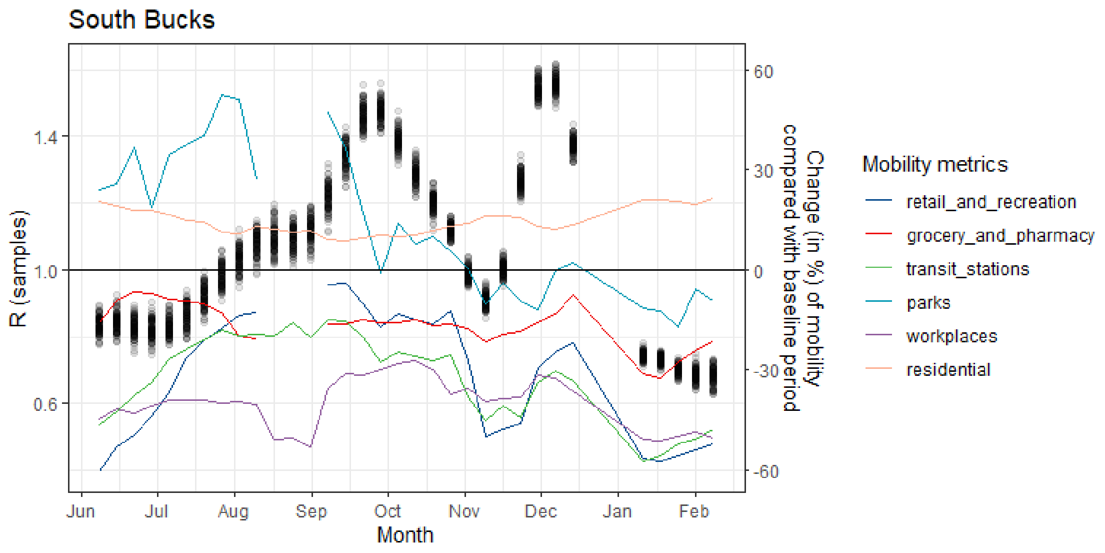
South Bucks
R (samples)
1.4
1.0
0.6
60
30
0
-30
-60
Change (in %) of mobility compared with baseline period
Jun
Jul
Aug
Sep
Oct
Nov
Dec
Jan
Feb
Month
Mobility metrics
retail_and_recreation
grocery_and_pharmacy
transit_stations
parks
workplaces
residential

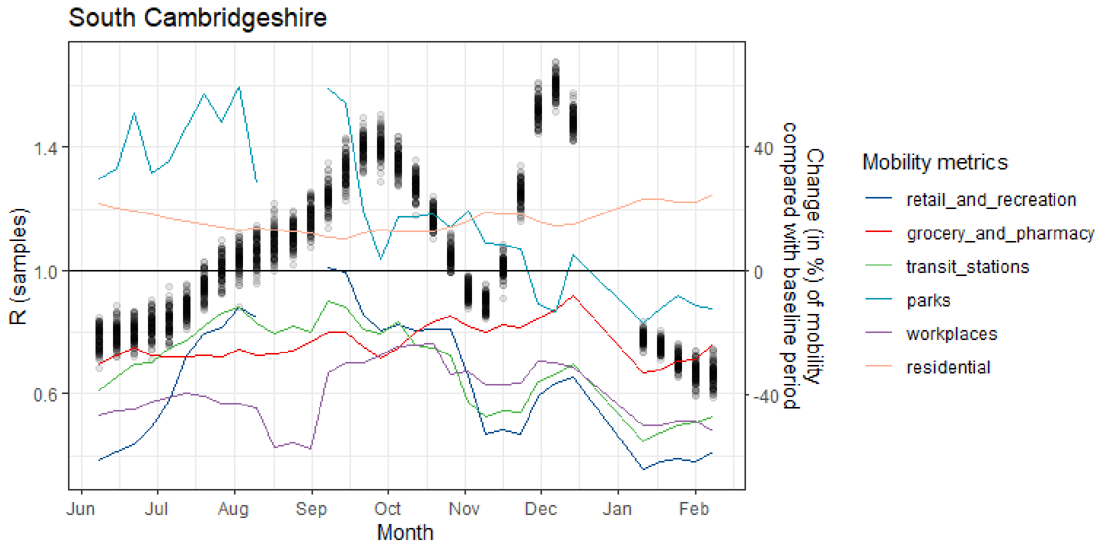
South Cambridgeshire
R (samples)
1.4
1.0
0.6
40
0
-40
Change (in %) of mobility compared with baseline period
Jun
Jul
Aug
Sep
Oct
Nov
Dec
Jan
Feb
Month
Mobility metrics
retail_and_recreation
grocery_and_pharmacy
transit_stations
parks
workplaces
residential

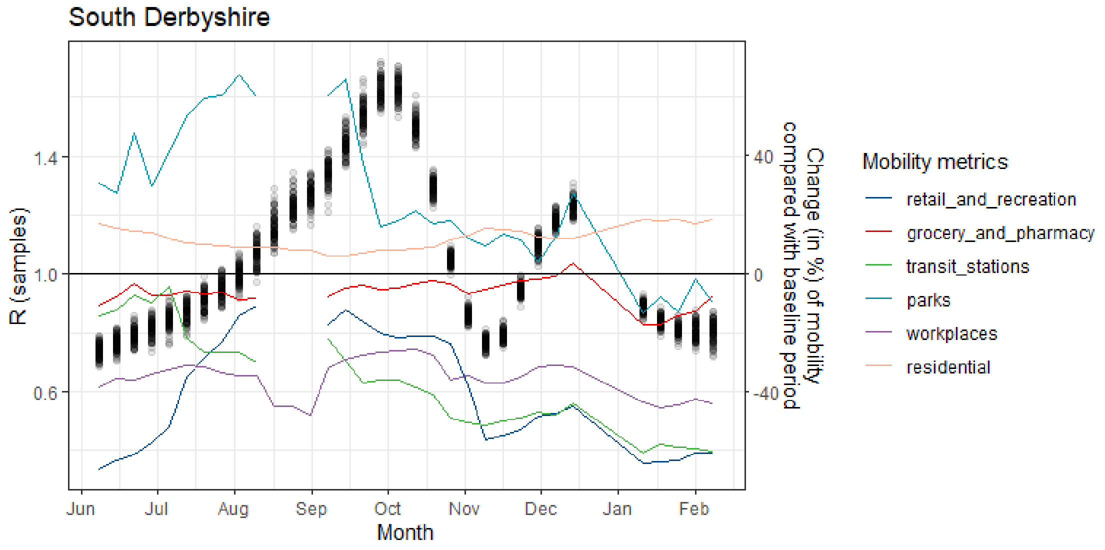
South Derbyshire
R (samples)
1.4
1.0
0.6
40
0
-40
Change (in %) of mobility compared with baseline period
Jun
Jul
Aug
Sep
Oct
Nov
Dec
Jan
Feb
Month
Mobility metrics
retail_and_recreation
grocery_and_pharmacy
transit_stations
parks
workplaces
residential

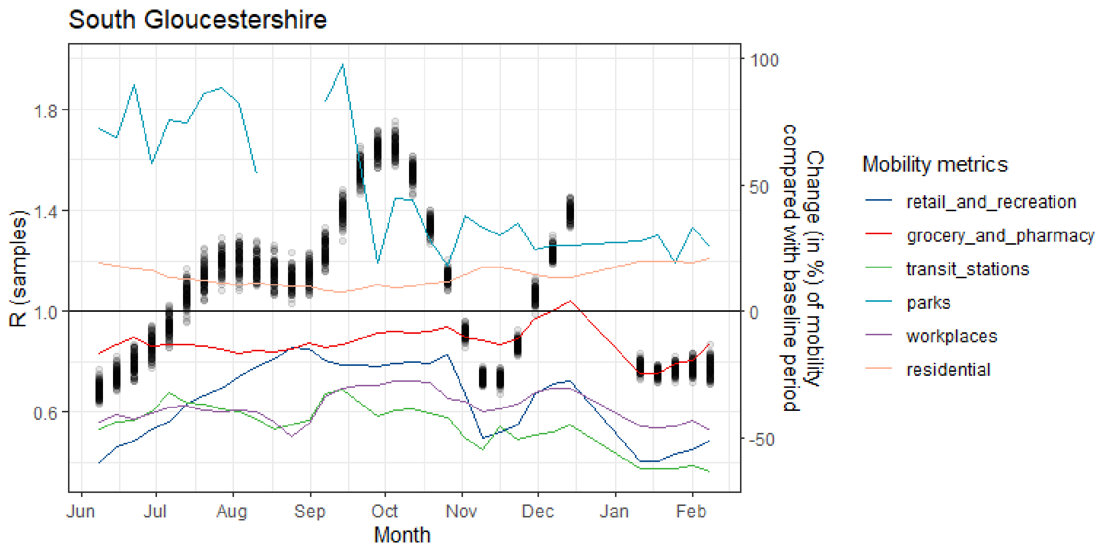

South Gloucestershire
R (samples)
1.8
1.4
1.0
0.6
Jun
Jul
Aug
Sep
Oct
Nov
Dec
Jan
Feb
Month
100
50
0
-50
Change (in %) of mobility compared with baseline period
Mobility metrics
retail_and_recreation
grocery_and_pharmacy
transit_stations
parks
workplaces
residential

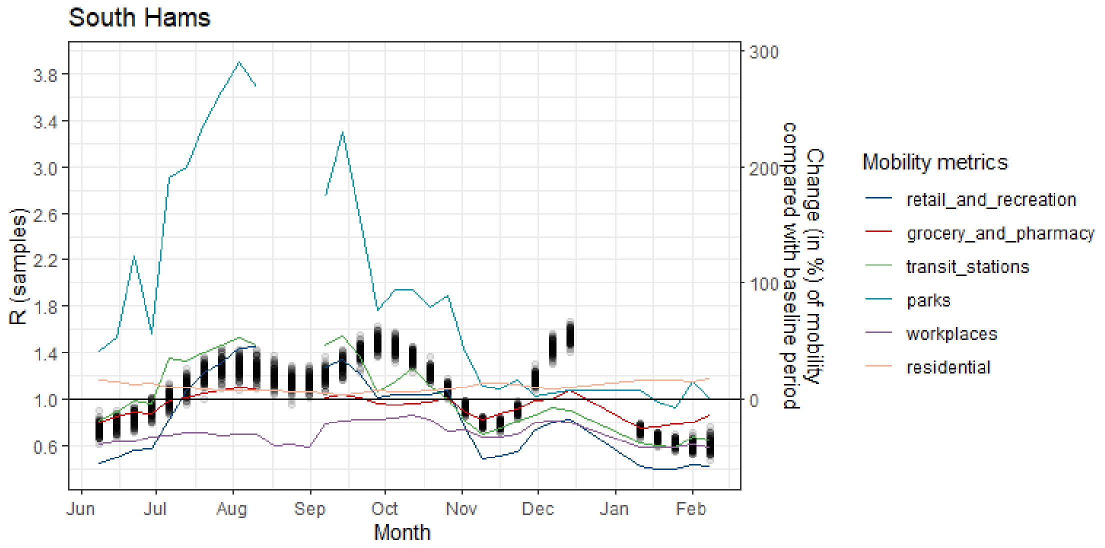

South Hams
R (samples)
3.8
3.4
3.0
2.6
2.2
1.8
1.4
1.0
0.6
Jun
Jul
Aug
Sep
Oct
Nov
Dec
Jan
Feb
Month
300
200
100
0
Change (in %) of mobility compared with baseline period
Mobility metrics
retail_and_recreation
grocery_and_pharmacy
transit_stations
parks
workplaces
residential

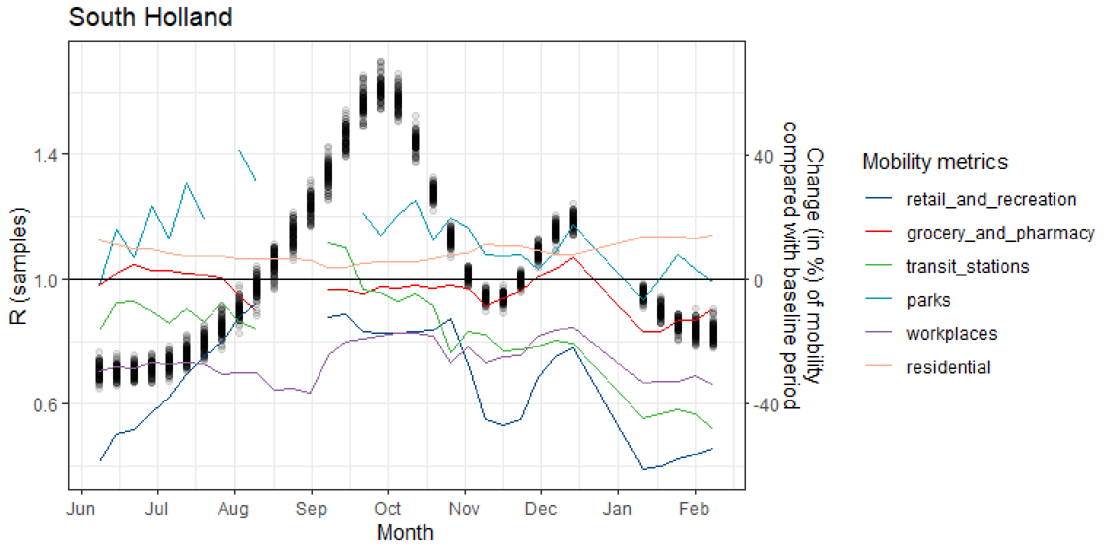

South Holland
R (samples)
1.4
1.0
0.6
Jun
Jul
Aug
Sep
Oct
Nov
Dec
Jan
Feb
Month
40
0
-40
Change (in %) of mobility compared with baseline period
Mobility metrics
retail_and_recreation
grocery_and_pharmacy
transit_stations
parks
workplaces
residential

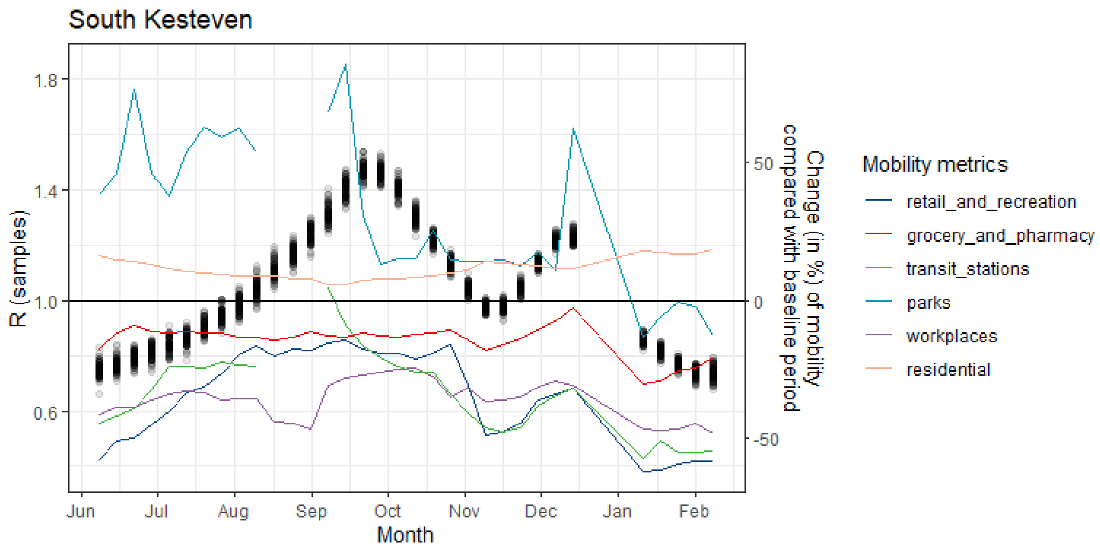
South Kesteven
R (samples)
1.8
1.4
1.0
0.6
Jun
Jul
Aug
Sep
Oct
Nov
Dec
Jan
Feb
Month
Change (in %) of mobility compared with baseline period
50
0
-50
Mobility metrics
retail_and_recreation
grocery_and_pharmacy
transit_stations
parks
workplaces
residential

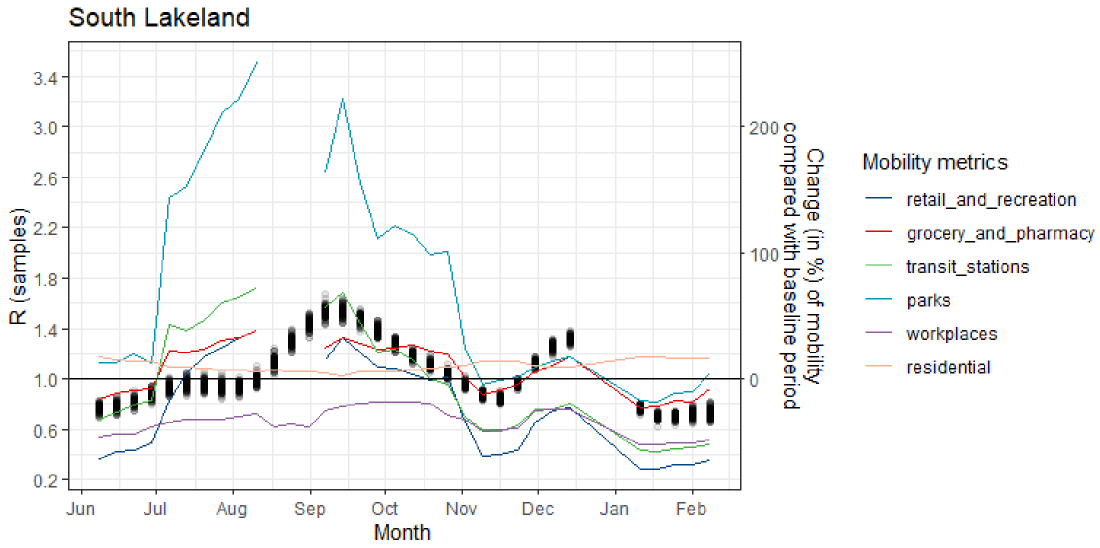
South Lakeland
R (samples)
3.4
3.0
2.6
2.2
1.8
1.4
1.0
0.6
0.2
Jun
Jul
Aug
Sep
Oct
Nov
Dec
Jan
Feb
Month
Change (in %) of mobility compared with baseline period
200
100
0
Mobility metrics
retail_and_recreation
grocery_and_pharmacy
transit_stations
parks
workplaces
residential

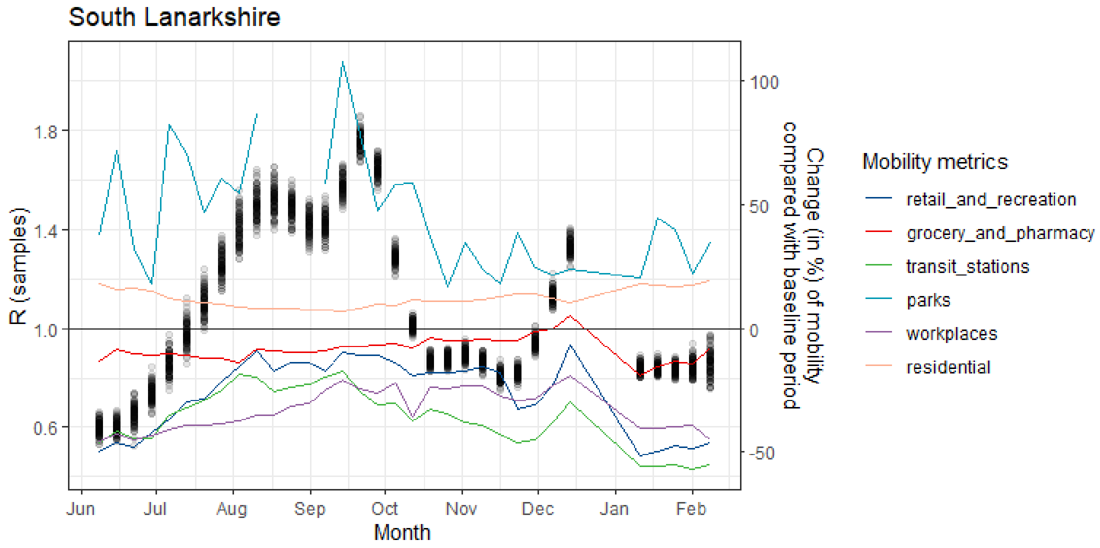
South Lanarkshire
R (samples)
1.8
1.4
1.0
0.6
Jun
Jul
Aug
Sep
Oct
Nov
Dec
Jan
Feb
Month
Change (in %) of mobility compared with baseline period
100
50
0
-50
Mobility metrics
retail_and_recreation
grocery_and_pharmacy
transit_stations
parks
workplaces
residential

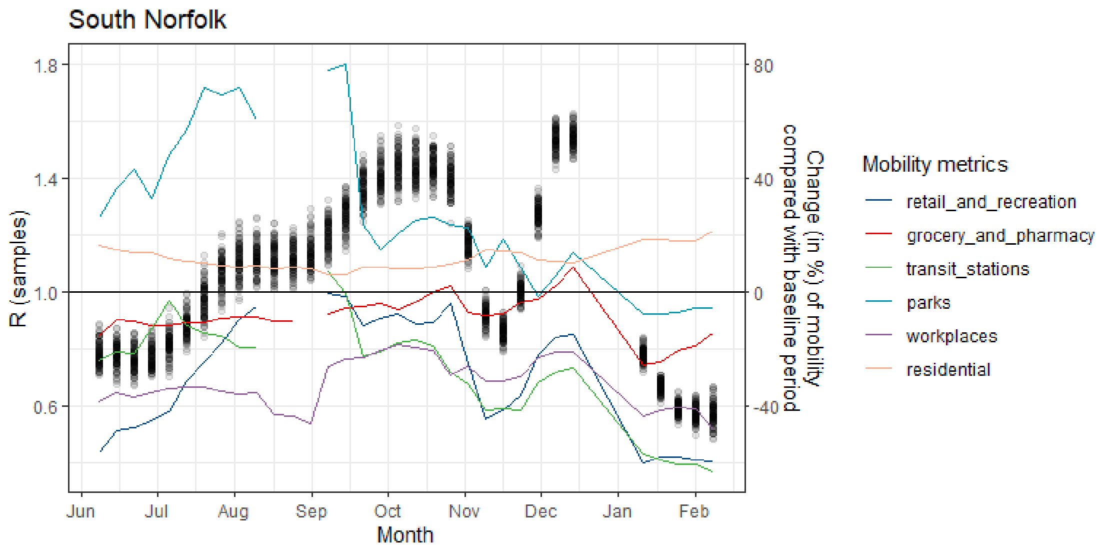
South Norfolk
R (samples)
1.8
1.4
1.0
0.6
Jun
Jul
Aug
Sep
Oct
Nov
Dec
Jan
Feb
Month
80
40
0
-40
Change (in %) of mobility compared with baseline period
Mobility metrics
retail_and_recreation
grocery_and_pharmacy
transit_stations
parks
workplaces
residential

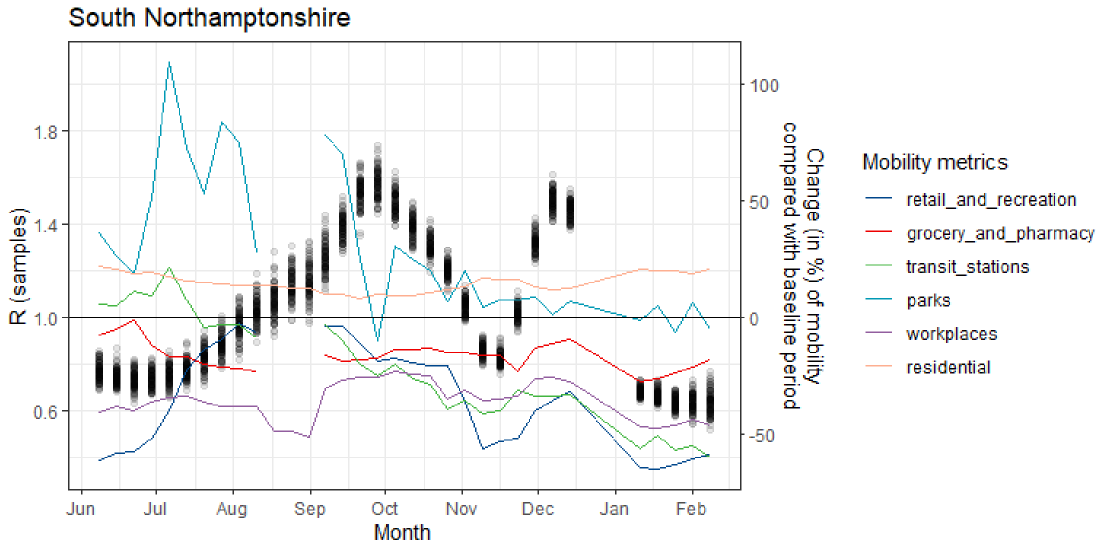
South Northamptonshire
R (samples)
1.8
1.4
1.0
0.6
Jun
Jul
Aug
Sep
Oct
Nov
Dec
Jan
Feb
Month
100
50
0
-50
Change (in %) of mobility compared with baseline period
Mobility metrics
retail_and_recreation
grocery_and_pharmacy
transit_stations
parks
workplaces
residential

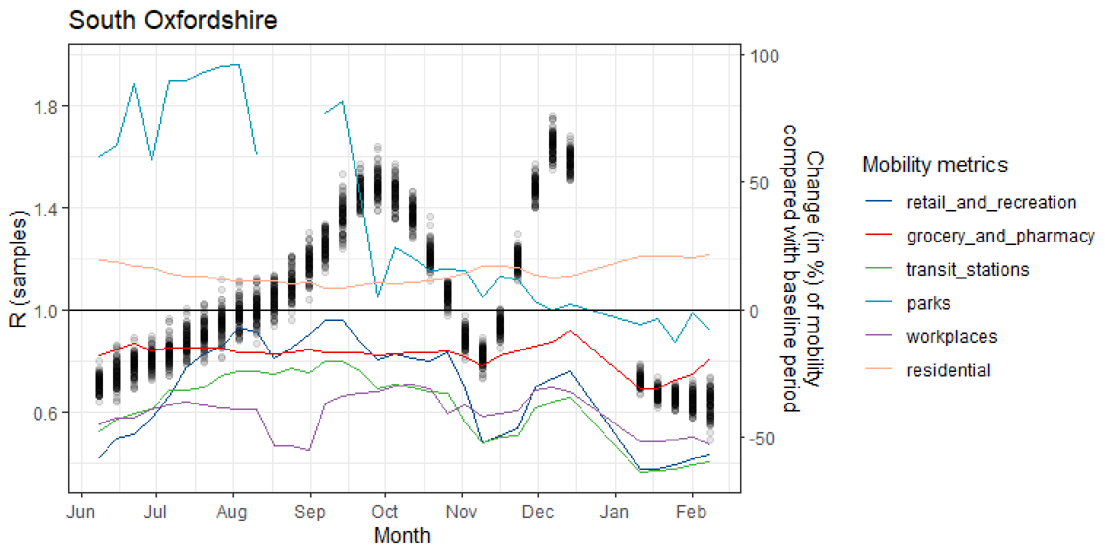
South Oxfordshire
R (samples)
1.8
1.4
1.0
0.6
Jun
Jul
Aug
Sep
Oct
Nov
Dec
Jan
Feb
Month
100
50
0
-50
Change (in %) of mobility compared with baseline period
Mobility metrics
retail_and_recreation
grocery_and_pharmacy
transit_stations
parks
workplaces
residential

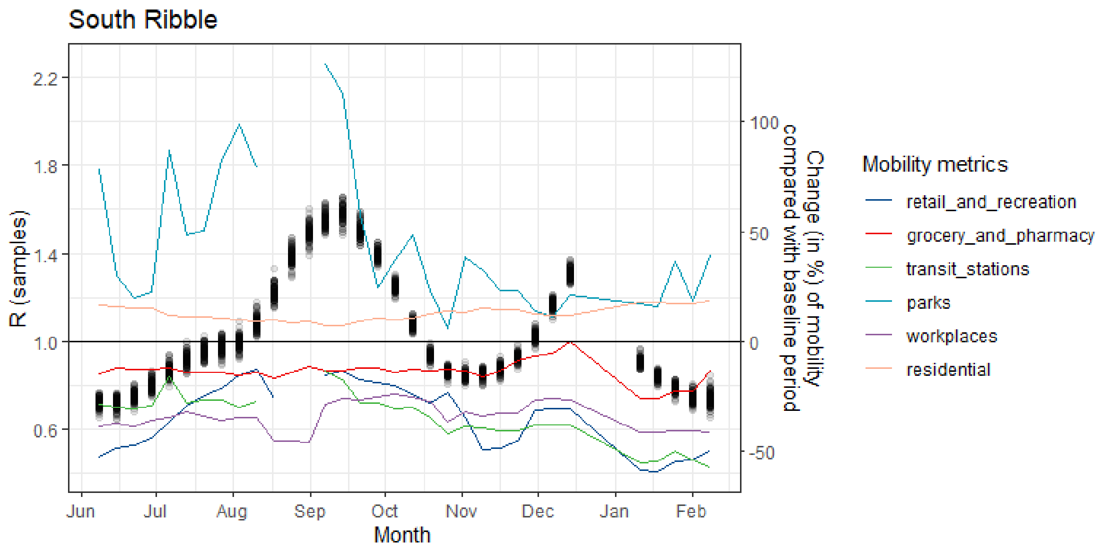
South Ribble
R (samples)
2.2
1.8
1.4
1.0
0.6
100
50
0
-50
Change (in %) of mobility compared with baseline period
Jun
Jul
Aug
Sep
Oct
Nov
Dec
Jan
Feb
Month
Mobility metrics
retail_and_recreation
grocery_and_pharmacy
transit_stations
parks
workplaces
residential

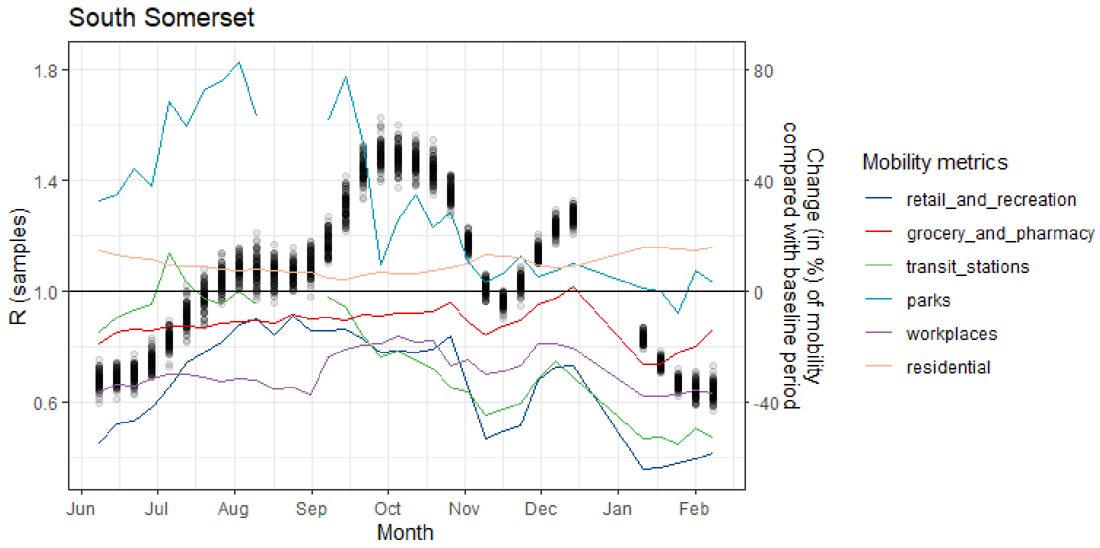
South Somerset
R (samples)
1.8
1.4
1.0
0.6
80
40
0
-40
Change (in %) of mobility compared with baseline period
Jun
Jul
Aug
Sep
Oct
Nov
Dec
Jan
Feb
Month
Mobility metrics
retail_and_recreation
grocery_and_pharmacy
transit_stations
parks
workplaces
residential

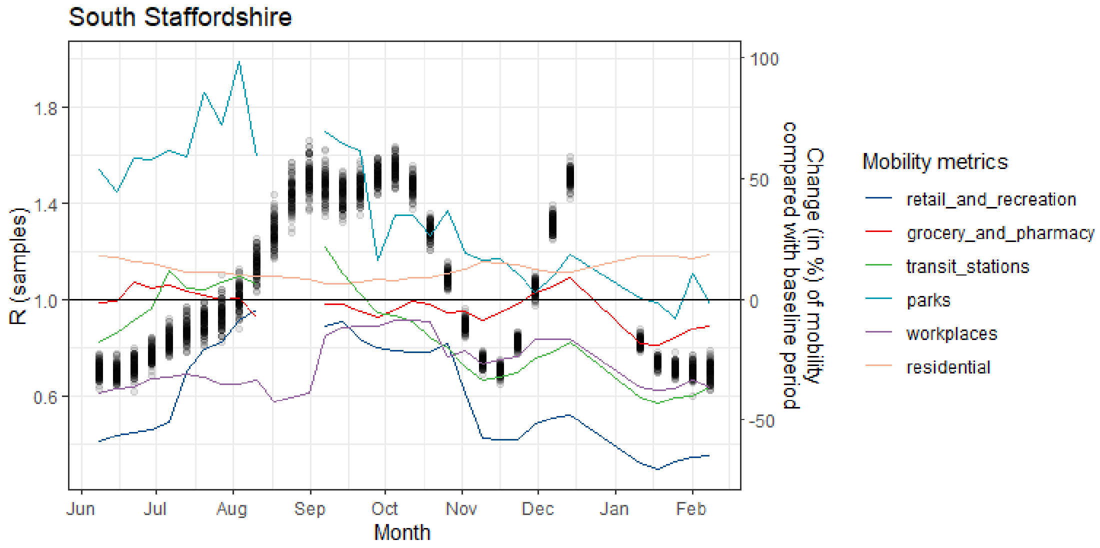
South Staffordshire
R (samples)
1.8
1.4
1.0
0.6
100
50
0
-50
Change (in %) of mobility compared with baseline period
Jun
Jul
Aug
Sep
Oct
Nov
Dec
Jan
Feb
Month
Mobility metrics
retail_and_recreation
grocery_and_pharmacy
transit_stations
parks
workplaces
residential

South Tyneside

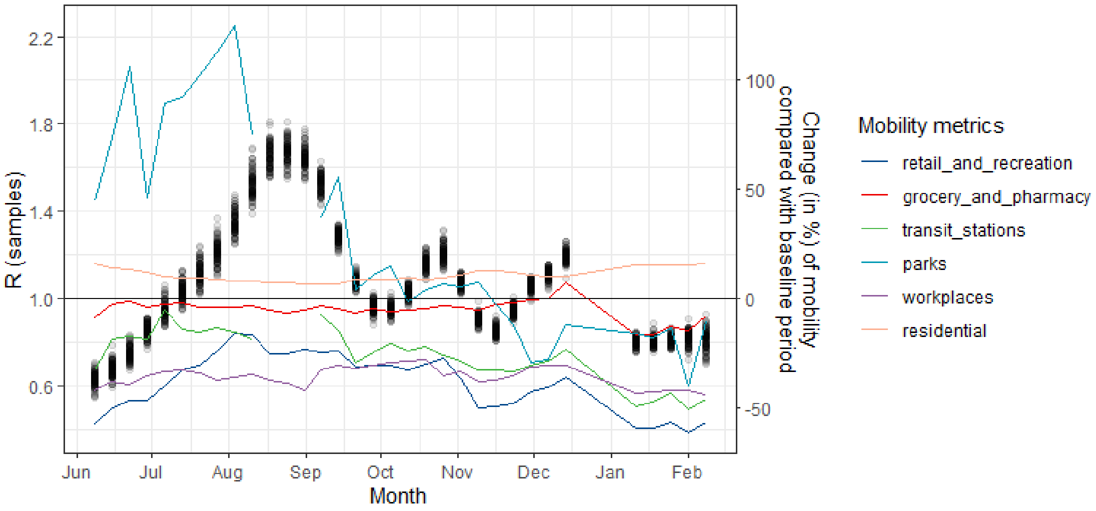

Southampton

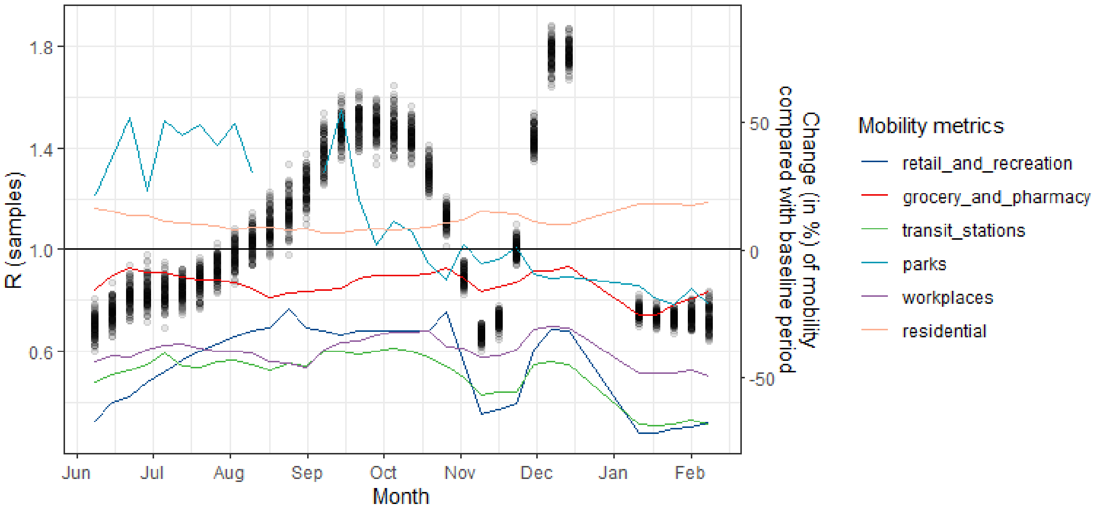

Southend-on-Sea

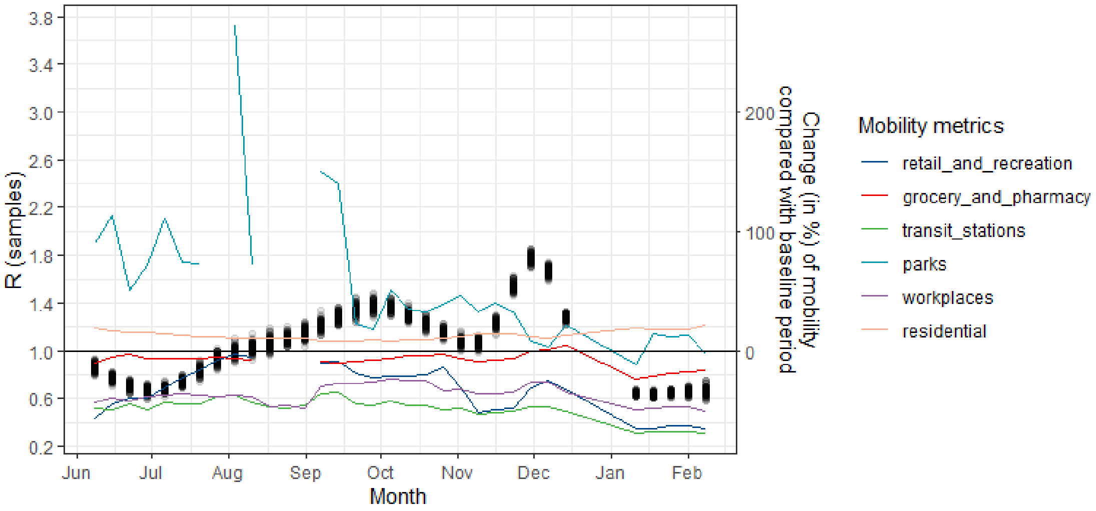

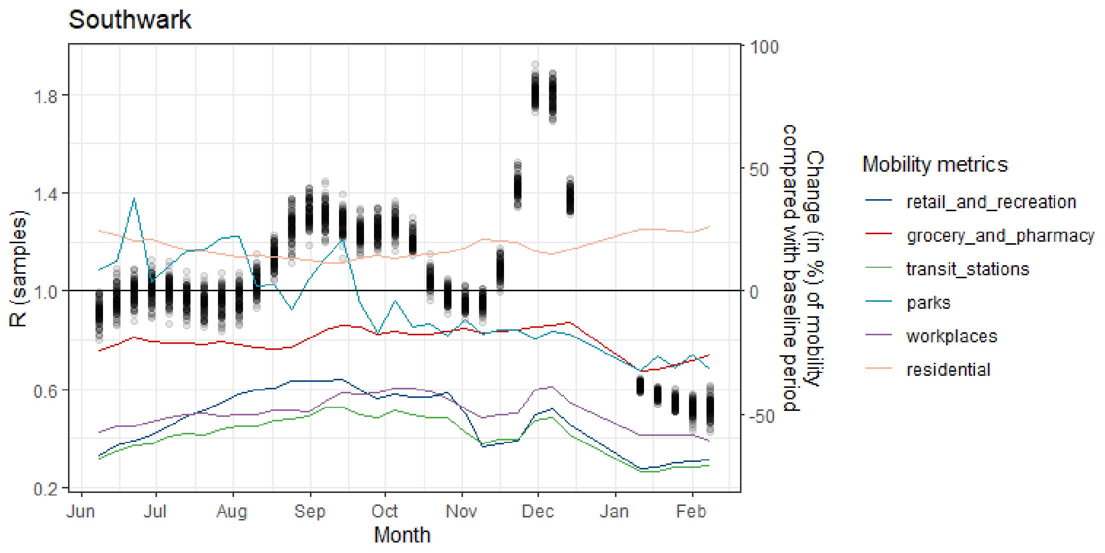
Southwark
R (samples)
1.8
1.4
1.0
0.6
0.2
Jun
Jul
Aug
Sep
Oct
Nov
Dec
Jan
Feb
Month
100
50
0
-50
Change (in %) of mobility compared with baseline period
Mobility metrics
retail_and_recreation
grocery_and_pharmacy
transit_stations
parks
workplaces
residential

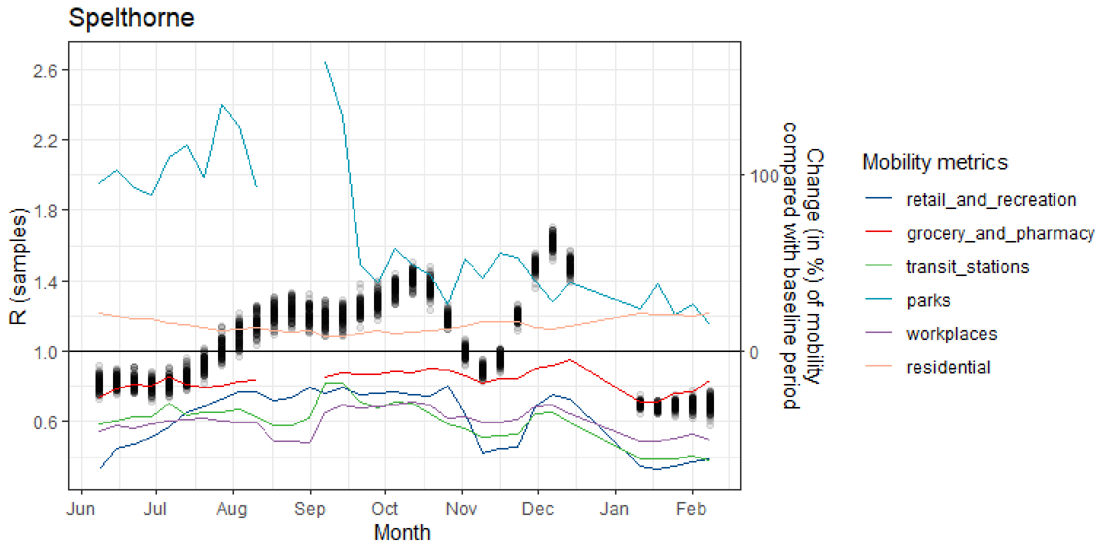
Spelthorne
R (samples)
2.6
2.2
1.8
1.4
1.0
0.6
Jun
Jul
Aug
Sep
Oct
Nov
Dec
Jan
Feb
Month
100
0
Change (in %) of mobility compared with baseline period
Mobility metrics
retail_and_recreation
grocery_and_pharmacy
transit_stations
parks
workplaces
residential

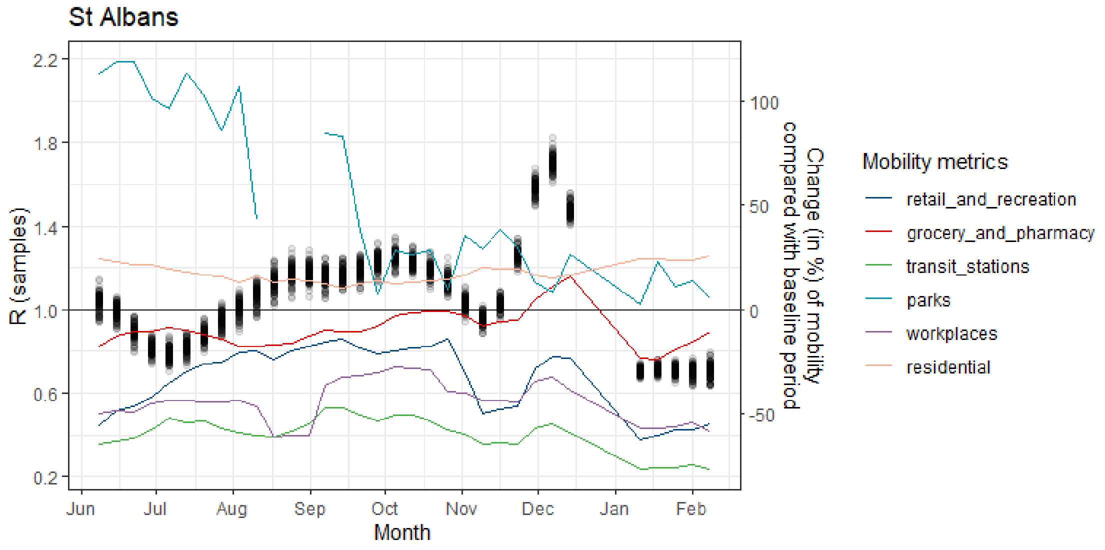
St Albans
R (samples)
2.2
1.8
1.4
1.0
0.6
0.2
Jun
Jul
Aug
Sep
Oct
Nov
Dec
Jan
Feb
Month
100
50
0
-50
Change (in %) of mobility compared with baseline period
Mobility metrics
retail_and_recreation
grocery_and_pharmacy
transit_stations
parks
workplaces
residential

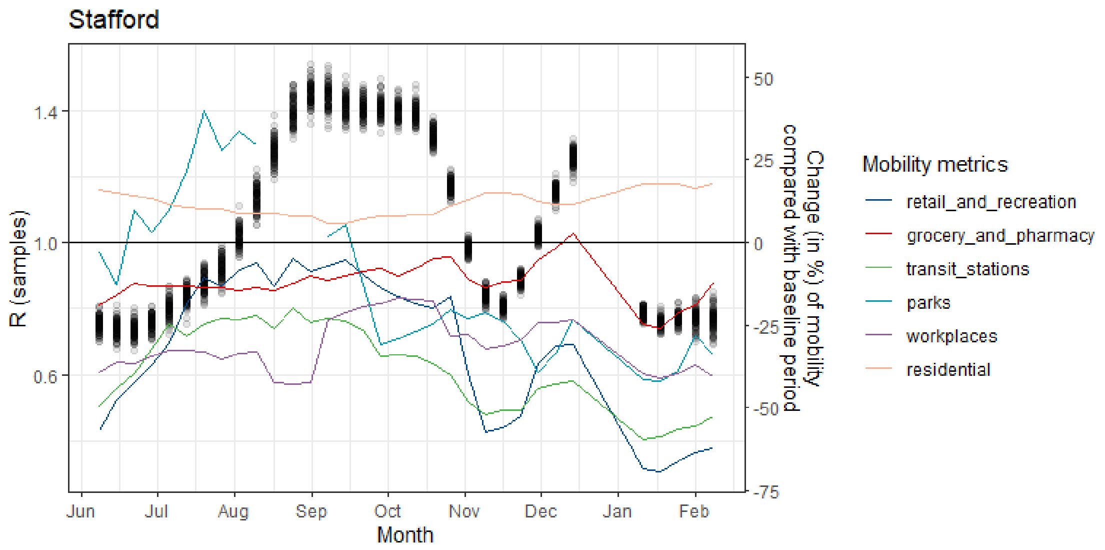
Stafford
R (samples)
1.4
1.0
0.6
50
25
0
-25
-50
-75
Change (in %) of mobility compared with baseline period
Jun
Jul
Aug
Sep
Oct
Nov
Dec
Jan
Feb
Month
Mobility metrics
retail_and_recreation
grocery_and_pharmacy
transit_stations
parks
workplaces
residential

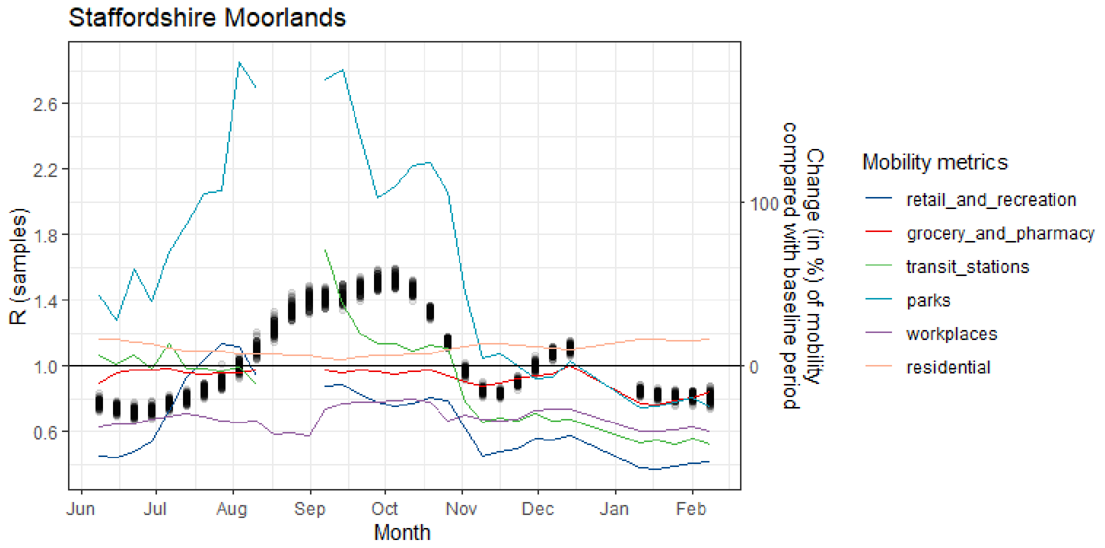
Staffordshire Moorlands
R (samples)
2.6
2.2
1.8
1.4
1.0
0.6
100
0
Change (in %) of mobility compared with baseline period
Jun
Jul
Aug
Sep
Oct
Nov
Dec
Jan
Feb
Month
Mobility metrics
retail_and_recreation
grocery_and_pharmacy
transit_stations
parks
workplaces
residential

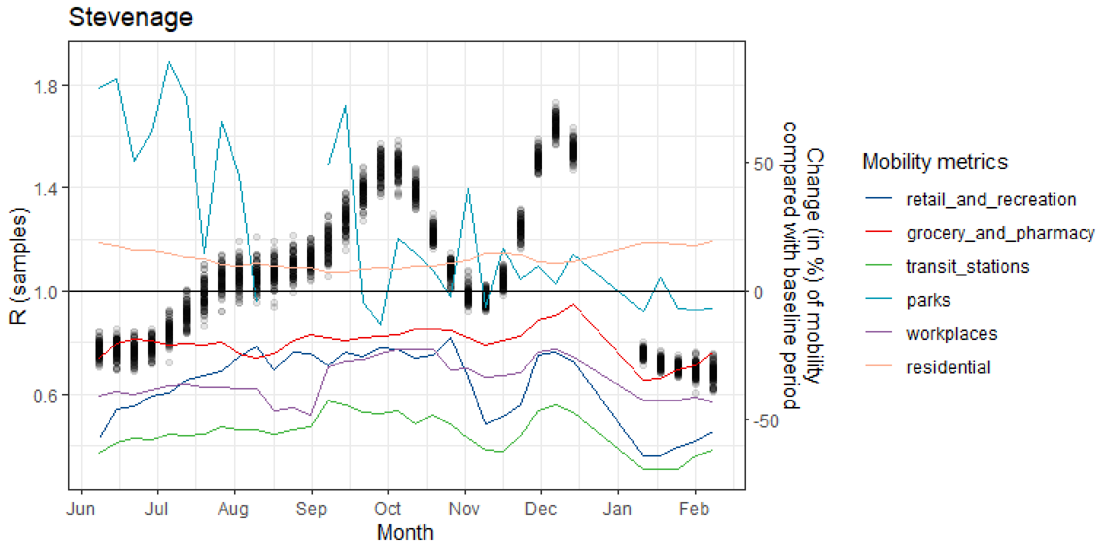
Stevenage
R (samples)
1.8
1.4
1.0
0.6
50
0
-50
Change (in %) of mobility compared with baseline period
Jun
Jul
Aug
Sep
Oct
Nov
Dec
Jan
Feb
Month
Mobility metrics
retail_and_recreation
grocery_and_pharmacy
transit_stations
parks
workplaces
residential

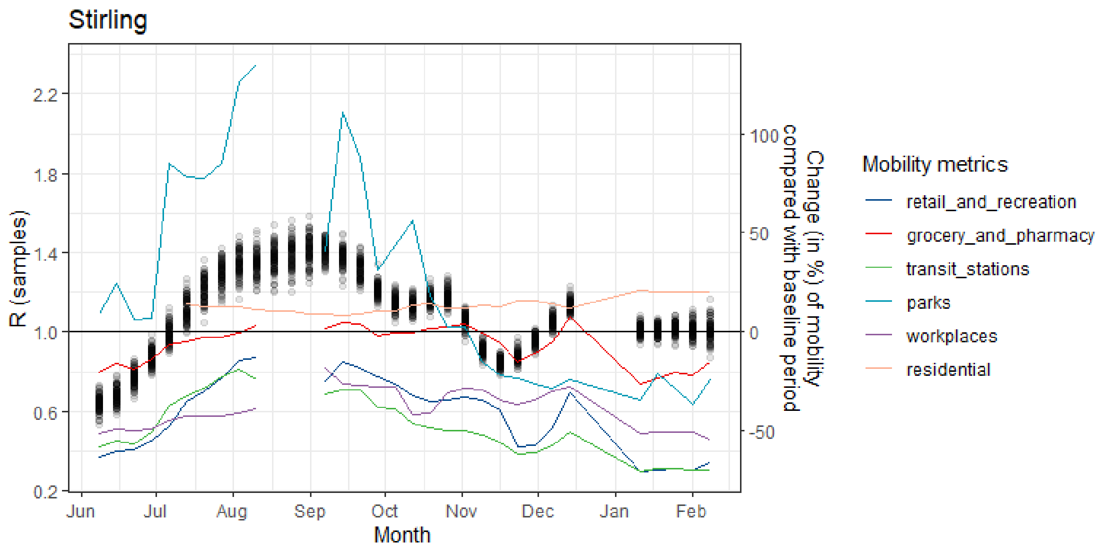
Stirling
R (samples)
2.2
1.8
1.4
1.0
0.6
0.2
Jun
Jul
Aug
Sep
Oct
Nov
Dec
Jan
Feb
Month
100
50
0
-50
Change (in %) of mobility
compared with baseline period
Mobility metrics
retail_and_recreation
grocery_and_pharmacy
transit_stations
parks
workplaces
residential

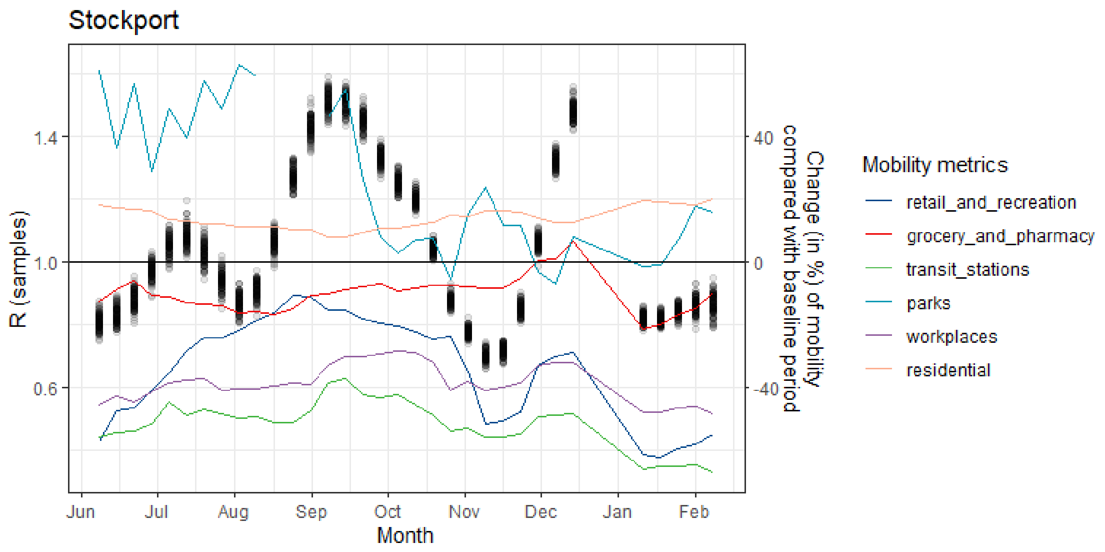
Stockport
R (samples)
1.4
1.0
0.6
Jun
Jul
Aug
Sep
Oct
Nov
Dec
Jan
Feb
Month
40
0
-40
Change (in %) of mobility
compared with baseline period
Mobility metrics
retail_and_recreation
grocery_and_pharmacy
transit_stations
parks
workplaces
residential

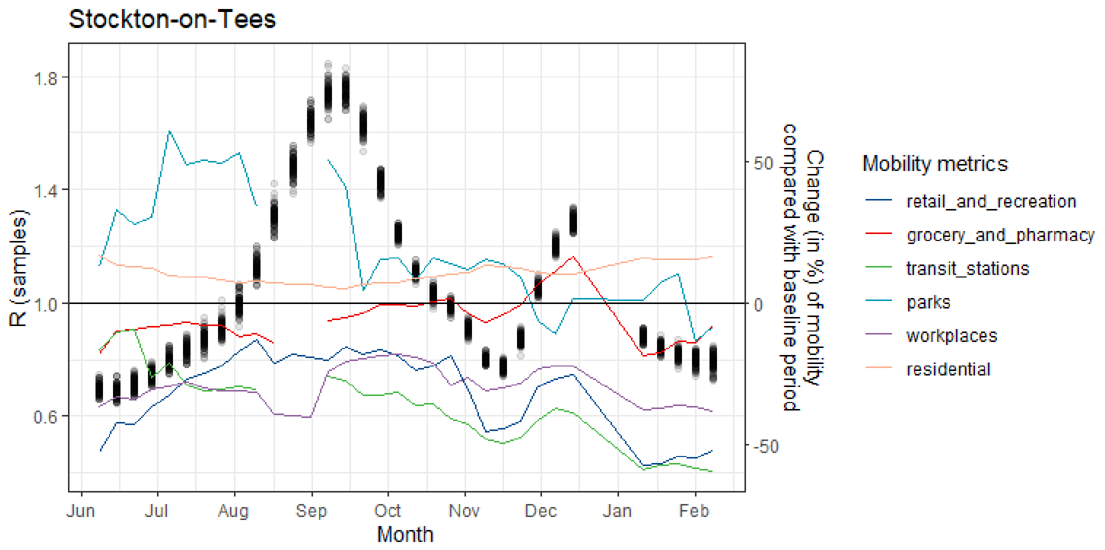
Stockton-on-Tees
R (samples)
1.8
1.4
1.0
0.6
Jun
Jul
Aug
Sep
Oct
Nov
Dec
Jan
Feb
Month
50
0
-50
Change (in %) of mobility
compared with baseline period
Mobility metrics
retail_and_recreation
grocery_and_pharmacy
transit_stations
parks
workplaces
residential

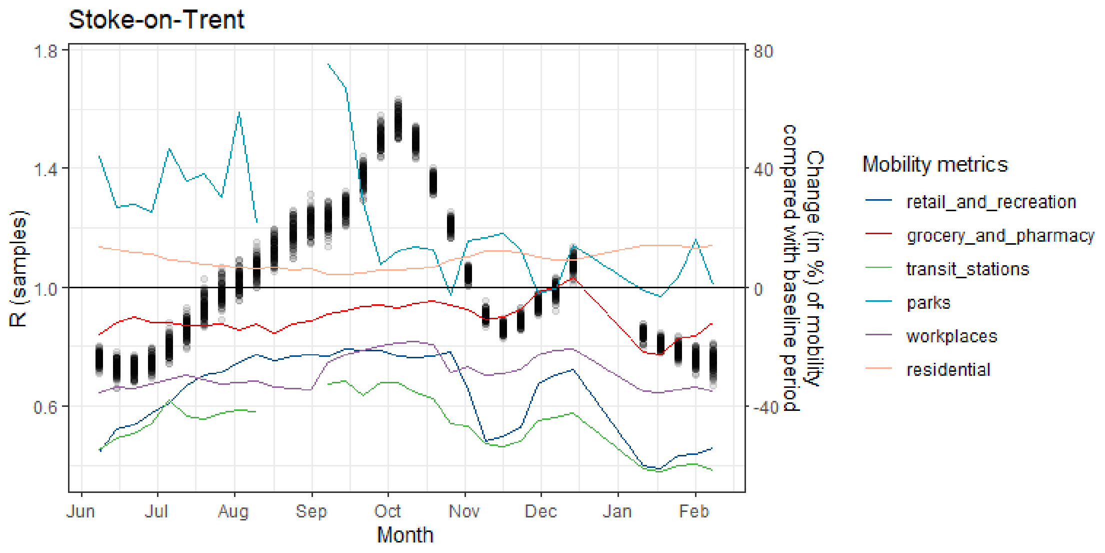

Stoke-on-Trent
R (samples)
1.8
1.4
1.0
0.6
80
40
0
-40
Change (in %) of mobility compared with baseline period
Jun
Jul
Aug
Sep
Oct
Nov
Dec
Jan
Feb
Month
Mobility metrics
retail_and_recreation
grocery_and_pharmacy
transit_stations
parks
workplaces
residential

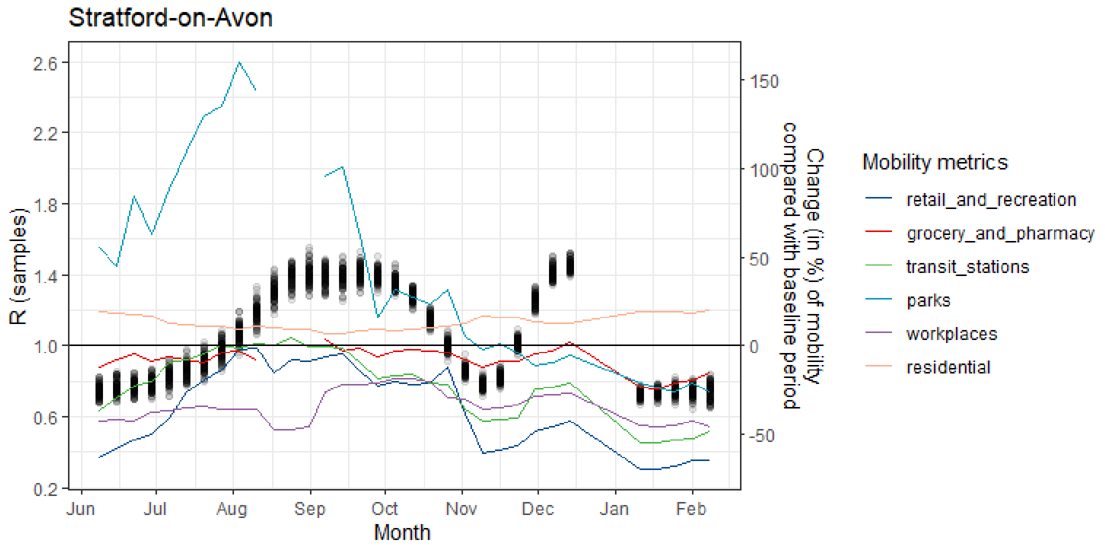

Stratford-on-Avon
R (samples)
2.6
2.2
1.8
1.4
1.0
0.6
0.2
150
100
50
0
-50
Change (in %) of mobility compared with baseline period
Jun
Jul
Aug
Sep
Oct
Nov
Dec
Jan
Feb
Month
Mobility metrics
retail_and_recreation
grocery_and_pharmacy
transit_stations
parks
workplaces
residential

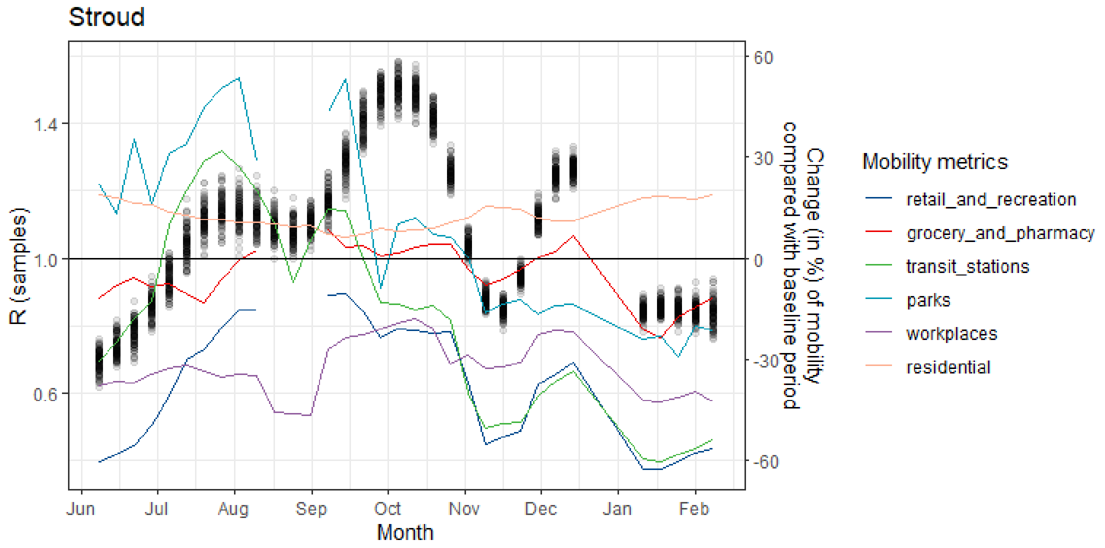

Stroud
R (samples)
1.4
1.0
0.6
60
30
0
-30
-60
Change (in %) of mobility compared with baseline period
Jun
Jul
Aug
Sep
Oct
Nov
Dec
Jan
Feb
Month
Mobility metrics
retail_and_recreation
grocery_and_pharmacy
transit_stations
parks
workplaces
residential

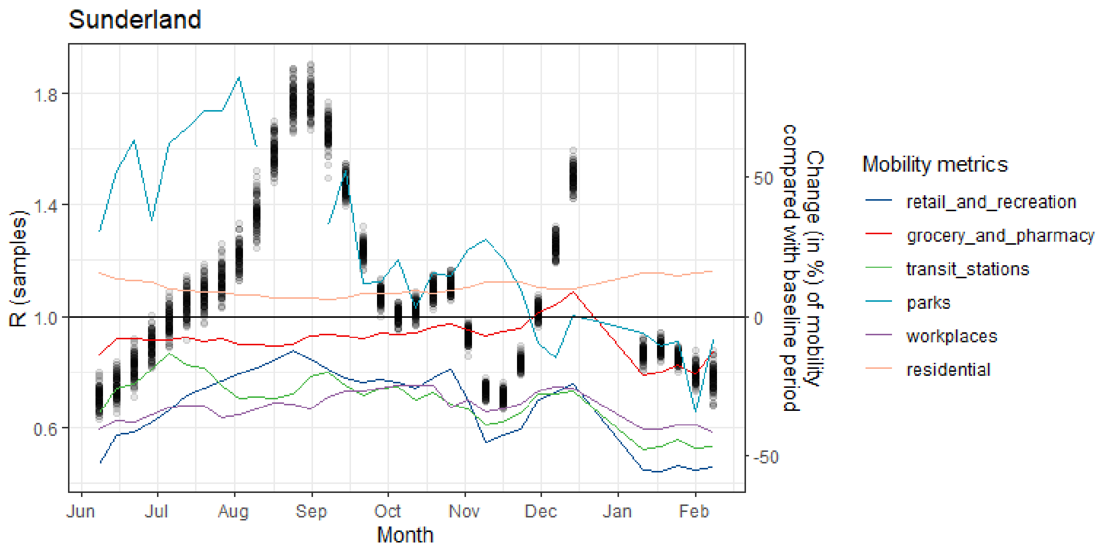

Sunderland
R (samples)
1.8
1.4
1.0
0.6
Jun
Jul
Aug
Sep
Oct
Nov
Dec
Jan
Feb
Month
50
0
-50
Change (in %) of mobility compared with baseline period
Mobility metrics
retail_and_recreation
grocery_and_pharmacy
transit_stations
parks
workplaces
residential

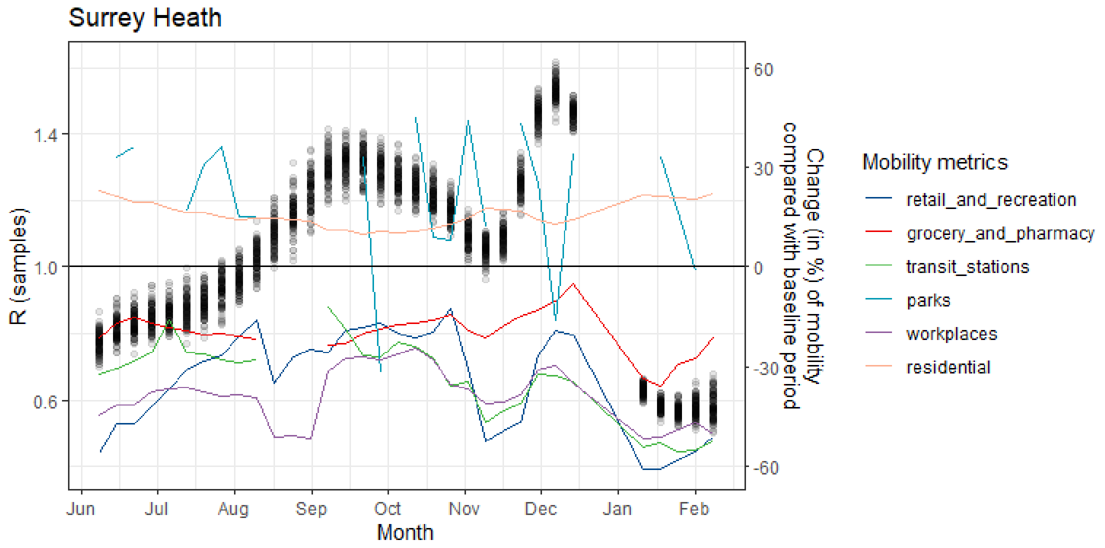

Surrey Heath
R (samples)
1.4
1.0
0.6
Jun
Jul
Aug
Sep
Oct
Nov
Dec
Jan
Feb
Month
60
30
0
-30
-60
Change (in %) of mobility compared with baseline period
Mobility metrics
retail_and_recreation
grocery_and_pharmacy
transit_stations
parks
workplaces
residential

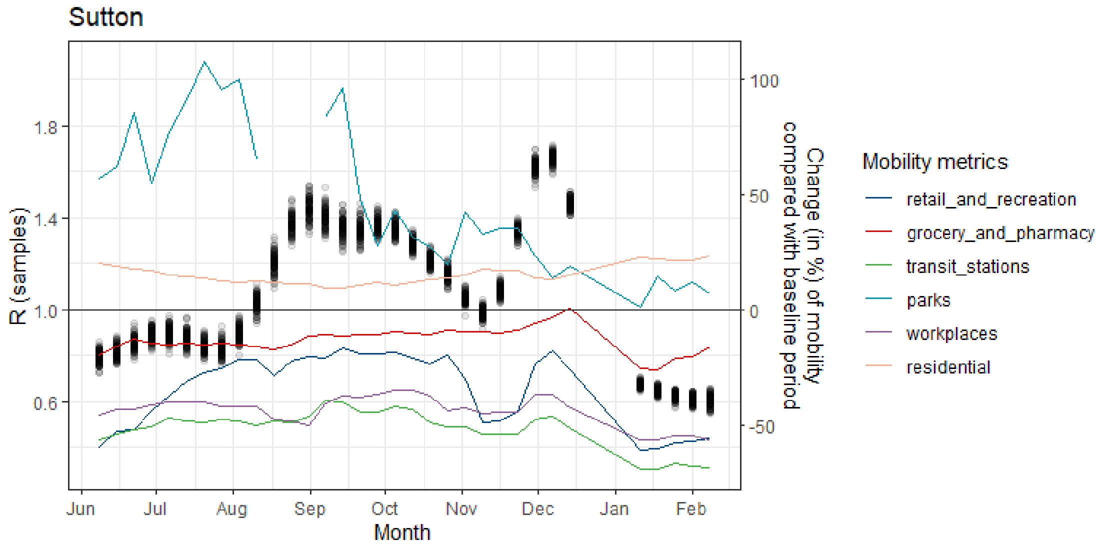

Sutton
R (samples)
1.8
1.4
1.0
0.6
Jun
Jul
Aug
Sep
Oct
Nov
Dec
Jan
Feb
Month
100
50
0
-50
Change (in %) of mobility compared with baseline period
Mobility metrics
retail_and_recreation
grocery_and_pharmacy
transit_stations
parks
workplaces
residential

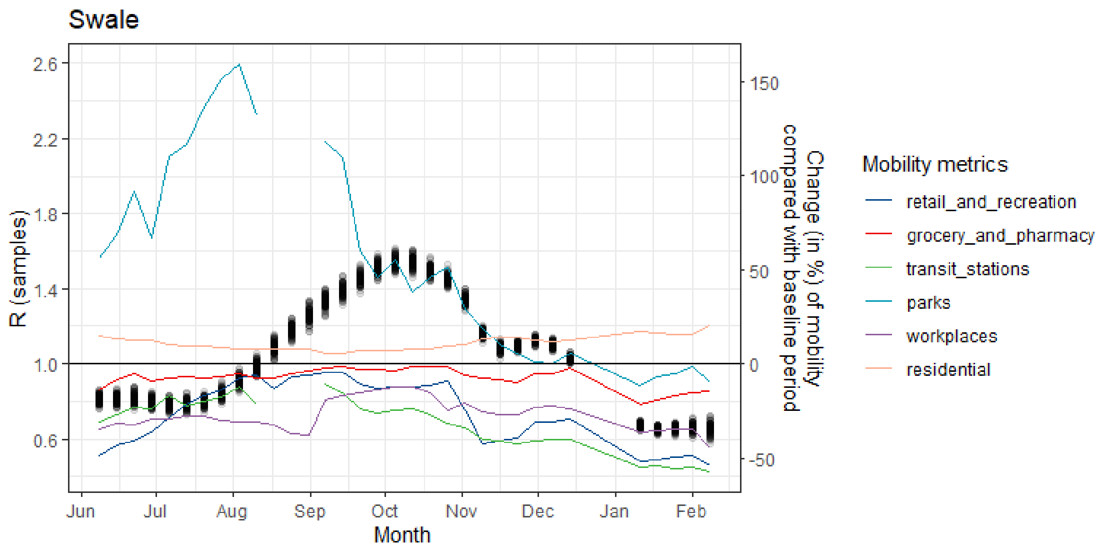
Swale
R (samples)
2.6
2.2
1.8
1.4
1.0
0.6
150
100
50
0
-50
Change (in %) of mobility compared with baseline period
Jun
Jul
Aug
Sep
Oct
Nov
Dec
Jan
Feb
Month
Mobility metrics
retail_and_recreation
grocery_and_pharmacy
transit_stations
parks
workplaces
residential

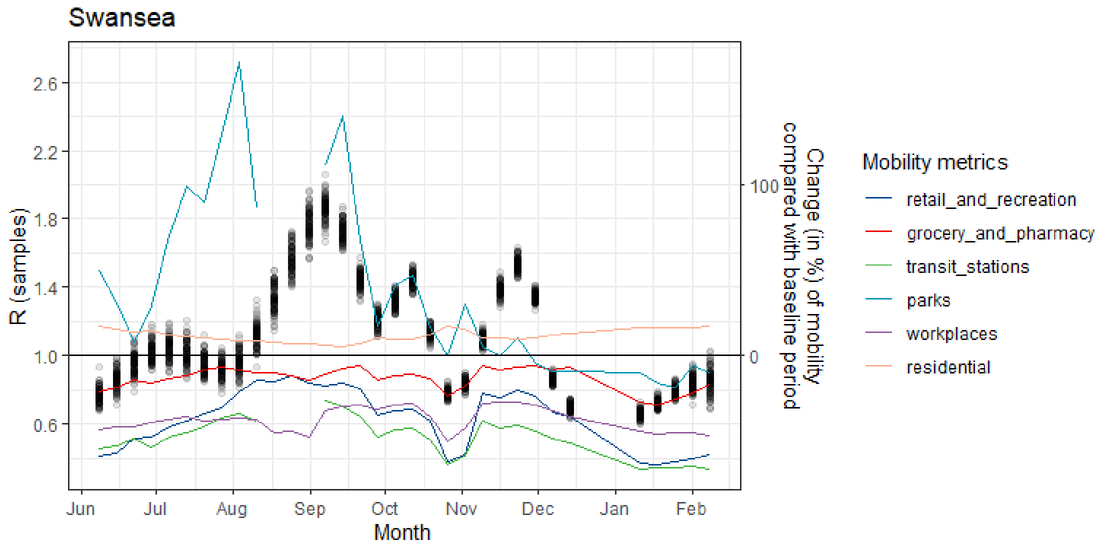
Swansea
R (samples)
2.6
2.2
1.8
1.4
1.0
0.6
100
0
Change (in %) of mobility compared with baseline period
Jun
Jul
Aug
Sep
Oct
Nov
Dec
Jan
Feb
Month
Mobility metrics
retail_and_recreation
grocery_and_pharmacy
transit_stations
parks
workplaces
residential

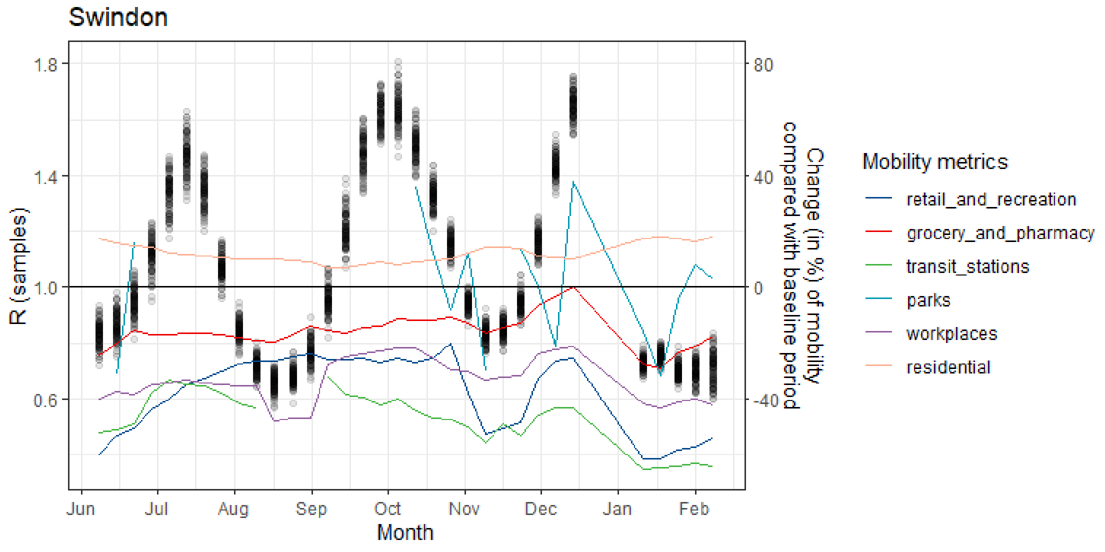
Swindon
R (samples)
1.8
1.4
1.0
0.6
80
40
0
-40
Change (in %) of mobility compared with baseline period
Jun
Jul
Aug
Sep
Oct
Nov
Dec
Jan
Feb
Month
Mobility metrics
retail_and_recreation
grocery_and_pharmacy
transit_stations
parks
workplaces
residential

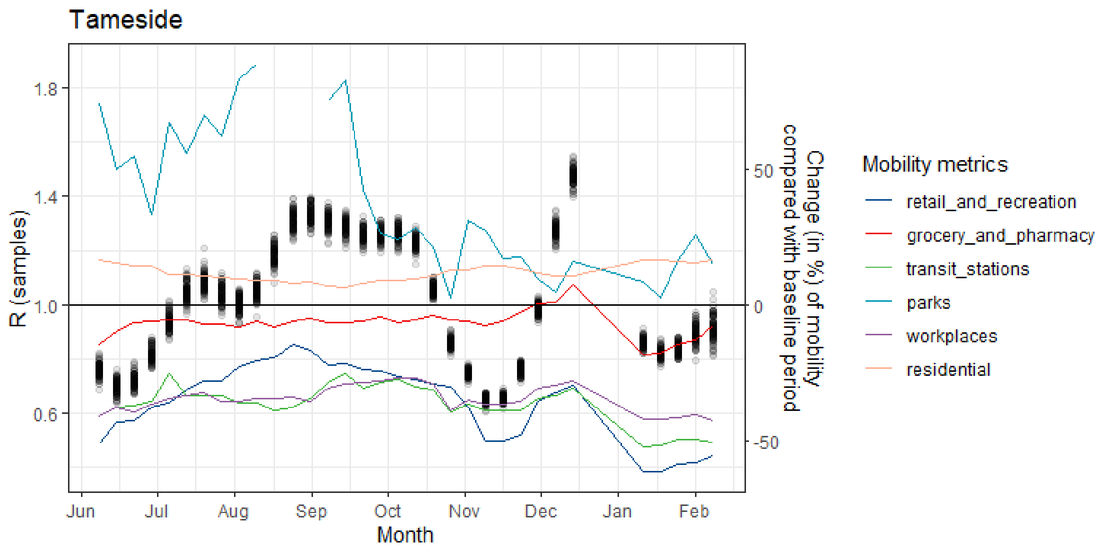
Tameside
R (samples)
1.8
1.4
1.0
0.6
Jun
Jul
Aug
Sep
Oct
Nov
Dec
Jan
Feb
Month
50
0
-50
Change (in %) of mobility
compared with baseline period
Mobility metrics
retail_and_recreation
grocery_and_pharmacy
transit_stations
parks
workplaces
residential

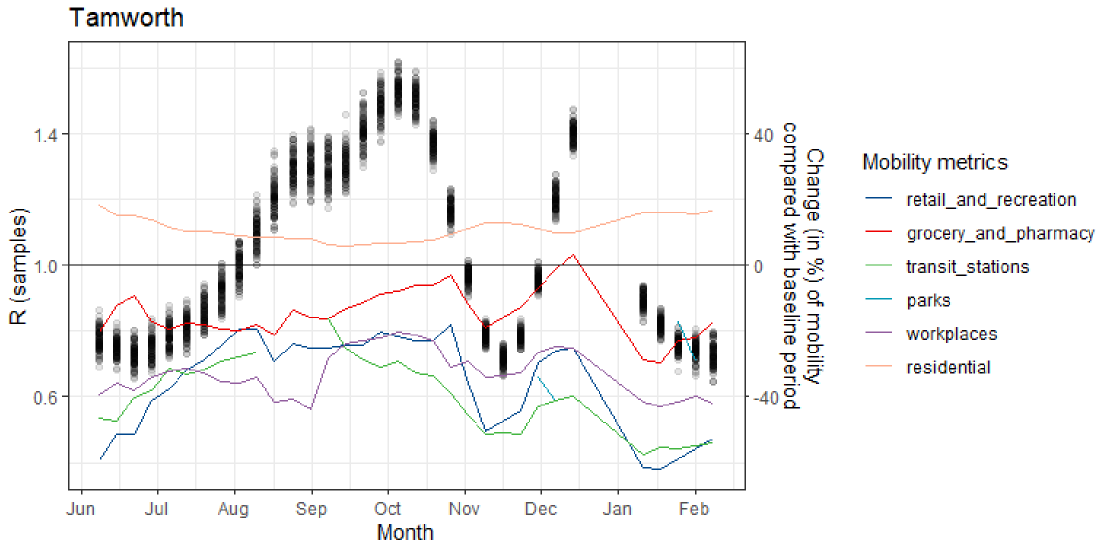
Tamworth
R (samples)
1.4
1.0
0.6
Jun
Jul
Aug
Sep
Oct
Nov
Dec
Jan
Feb
Month
40
0
-40
Change (in %) of mobility
compared with baseline period
Mobility metrics
retail_and_recreation
grocery_and_pharmacy
transit_stations
parks
workplaces
residential

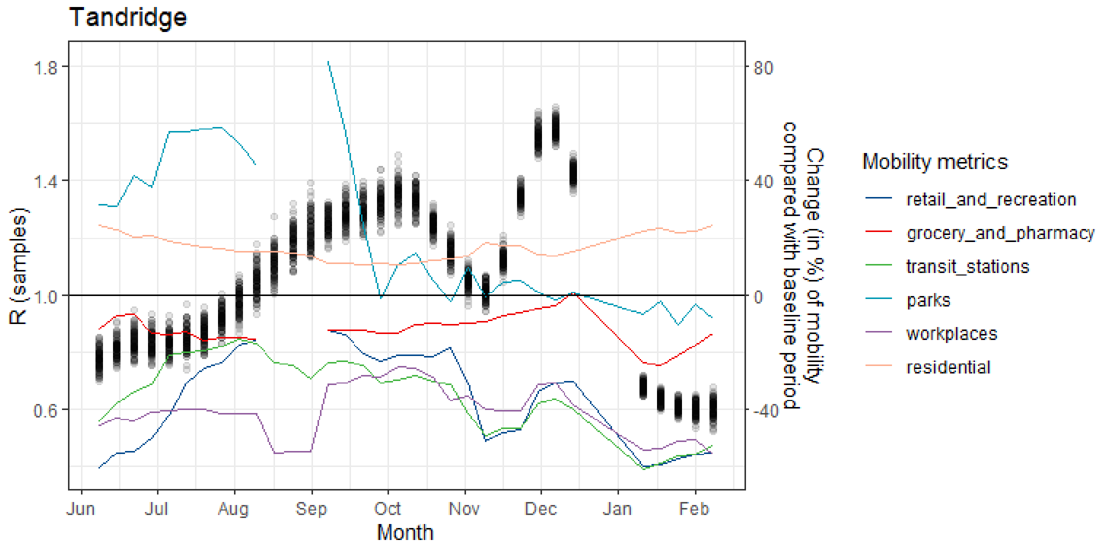
Tandridge
R (samples)
1.8
1.4
1.0
0.6
Jun
Jul
Aug
Sep
Oct
Nov
Dec
Jan
Feb
Month
80
40
0
-40
Change (in %) of mobility
compared with baseline period
Mobility metrics
retail_and_recreation
grocery_and_pharmacy
transit_stations
parks
workplaces
residential

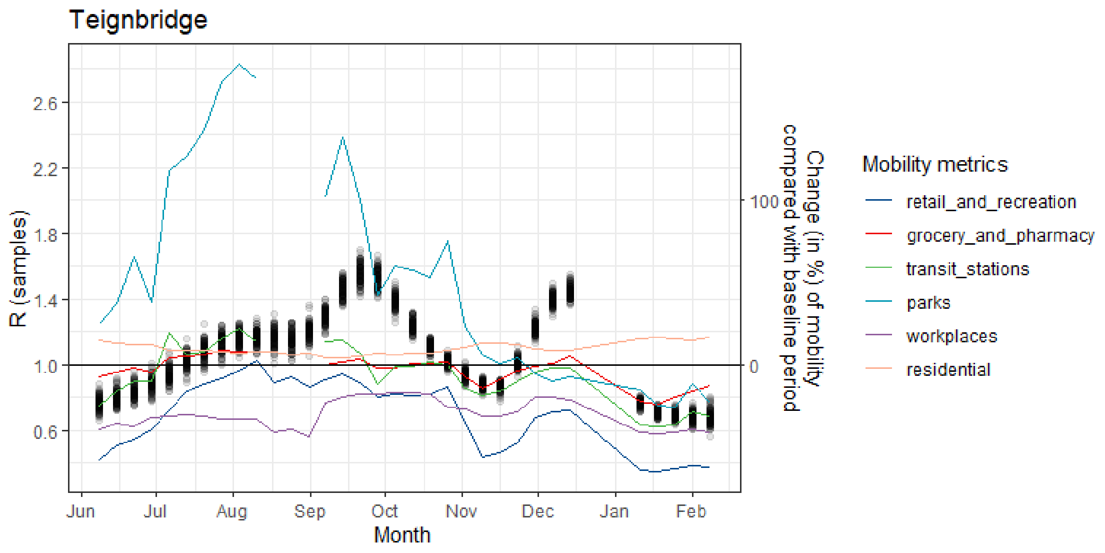
Teignbridge
R (samples)
2.6
2.2
1.8
1.4
1.0
0.6
Jun
Jul
Aug
Sep
Oct
Nov
Dec
Jan
Feb
Month
100
0
Change (in %) of mobility compared with baseline period
Mobility metrics
retail_and_recreation
grocery_and_pharmacy
transit_stations
parks
workplaces
residential

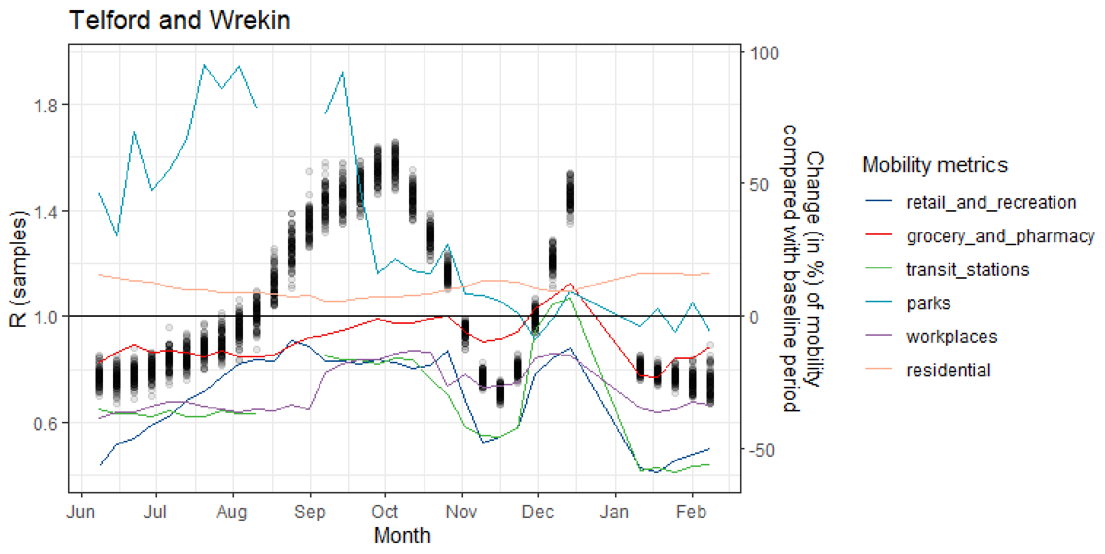
Telford and Wrekin
R (samples)
1.8
1.4
1.0
0.6
Jun
Jul
Aug
Sep
Oct
Nov
Dec
Jan
Feb
Month
100
50
0
-50
Change (in %) of mobility compared with baseline period
Mobility metrics
retail_and_recreation
grocery_and_pharmacy
transit_stations
parks
workplaces
residential

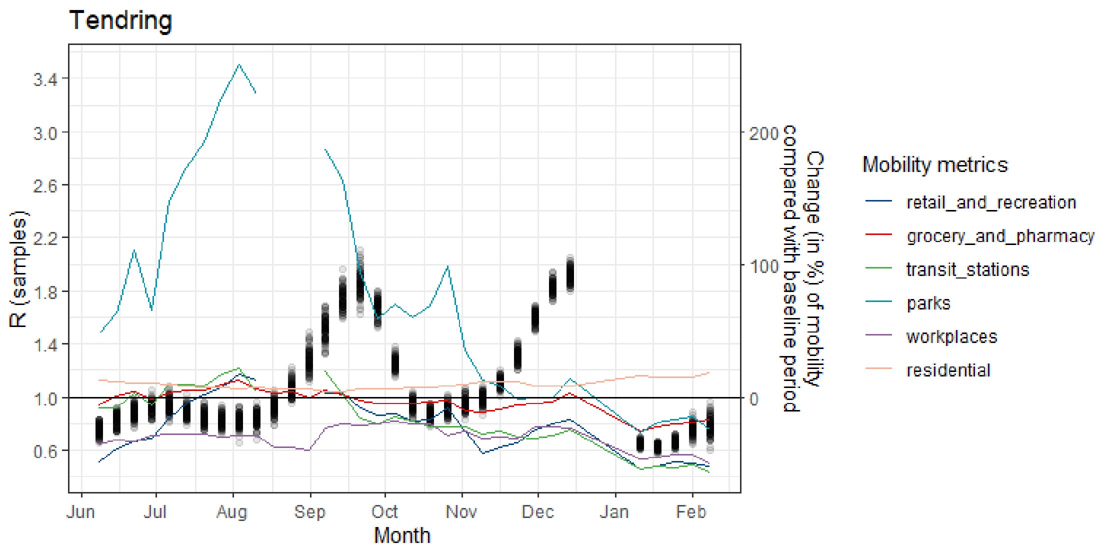
Tendring
R (samples)
3.4
3.0
2.6
2.2
1.8
1.4
1.0
0.6
Jun
Jul
Aug
Sep
Oct
Nov
Dec
Jan
Feb
Month
200
100
0
Change (in %) of mobility compared with baseline period
Mobility metrics
retail_and_recreation
grocery_and_pharmacy
transit_stations
parks
workplaces
residential

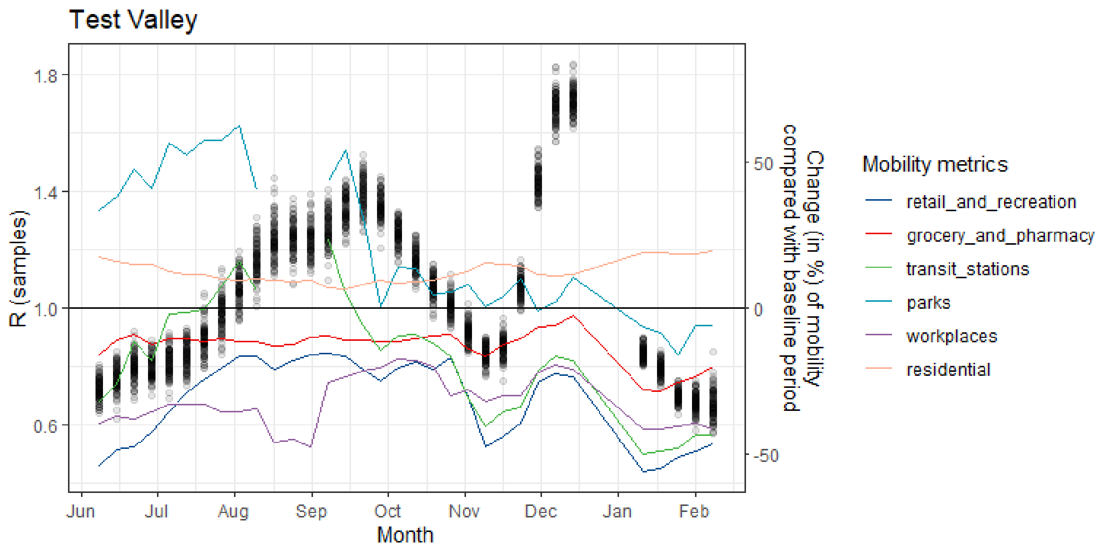
Test Valley
R (samples)
1.8
1.4
1.0
0.6
Jun
Jul
Aug
Sep
Oct
Nov
Dec
Jan
Feb
Month
50
0
-50
Change (in %) of mobility
compared with baseline period
Mobility metrics
retail_and_recreation
grocery_and_pharmacy
transit_stations
parks
workplaces
residential

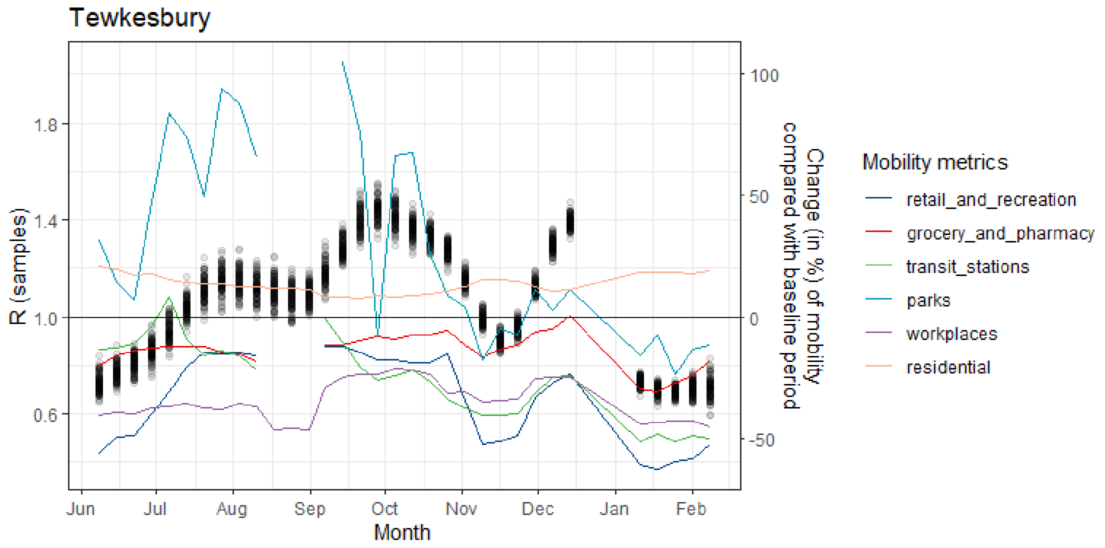
Tewkesbury
R (samples)
1.8
1.4
1.0
0.6
Jun
Jul
Aug
Sep
Oct
Nov
Dec
Jan
Feb
Month
100
50
0
-50
Change (in %) of mobility
compared with baseline period
Mobility metrics
retail_and_recreation
grocery_and_pharmacy
transit_stations
parks
workplaces
residential

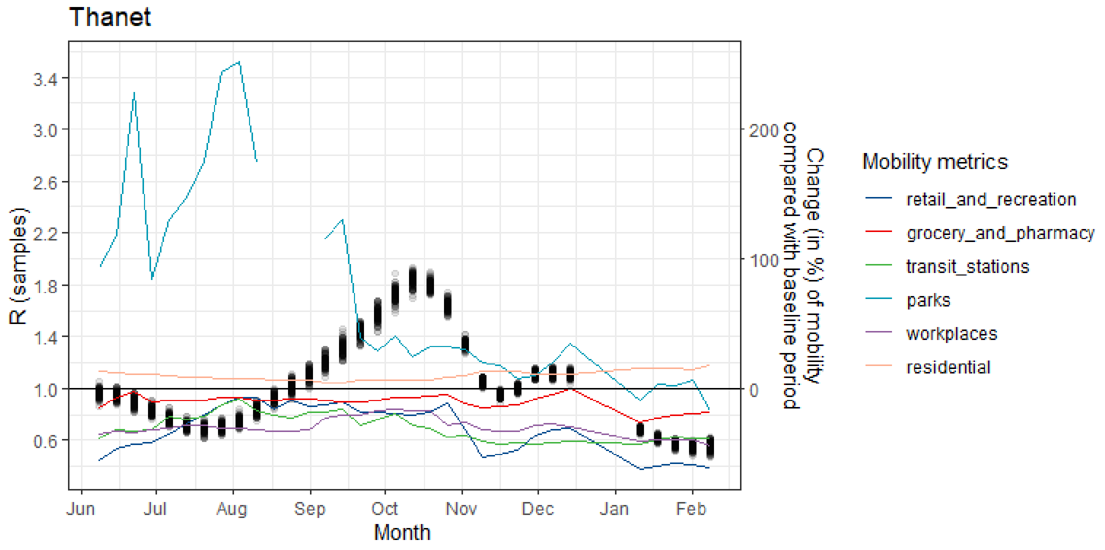
Thanet
R (samples)
3.4
3.0
2.6
2.2
1.8
1.4
1.0
0.6
Jun
Jul
Aug
Sep
Oct
Nov
Dec
Jan
Feb
Month
200
100
0
Change (in %) of mobility
compared with baseline period
Mobility metrics
retail_and_recreation
grocery_and_pharmacy
transit_stations
parks
workplaces
residential

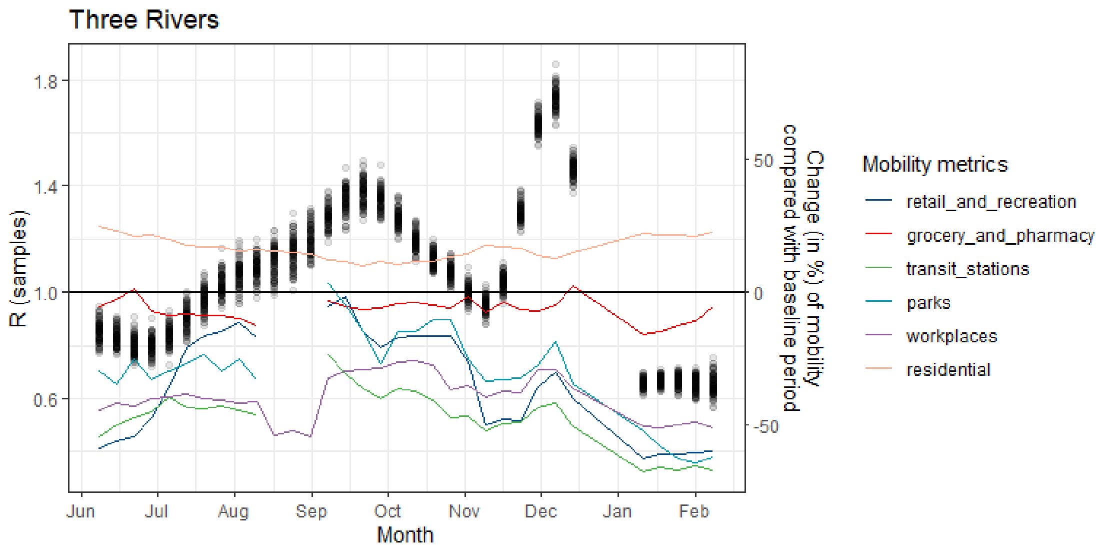

Three Rivers
R (samples)
1.8
1.4
1.0
0.6
Change (in %) of mobility
compared with baseline period
50
0
-50
Jun
Jul
Aug
Sep
Oct
Nov
Dec
Jan
Feb
Month
Mobility metrics
retail_and_recreation
grocery_and_pharmacy
transit_stations
parks
workplaces
residential

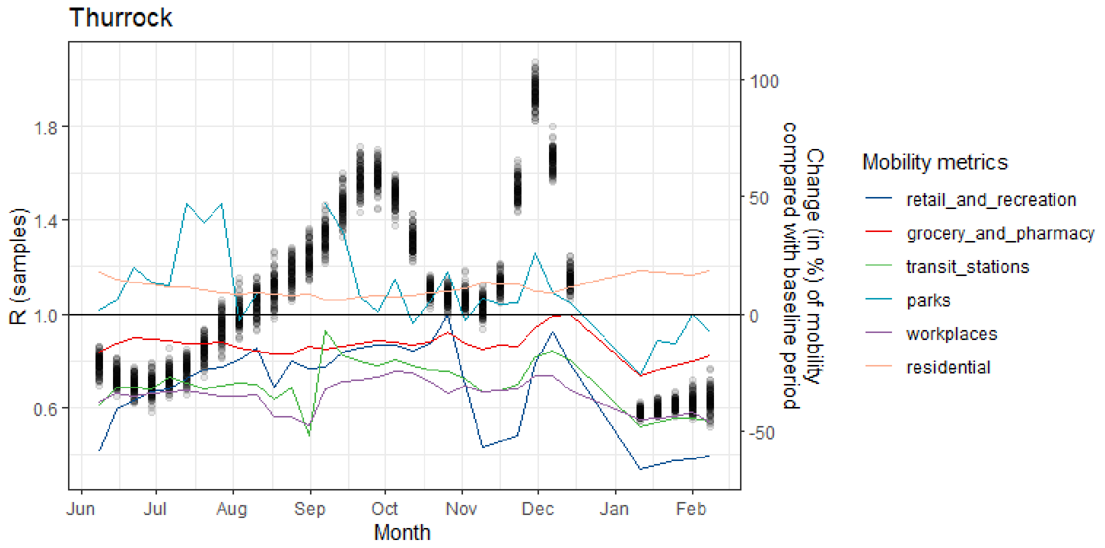

Thurrock
R (samples)
1.8
1.4
1.0
0.6
Change (in %) of mobility
compared with baseline period
100
50
0
-50
Jun
Jul
Aug
Sep
Oct
Nov
Dec
Jan
Feb
Month
Mobility metrics
retail_and_recreation
grocery_and_pharmacy
transit_stations
parks
workplaces
residential

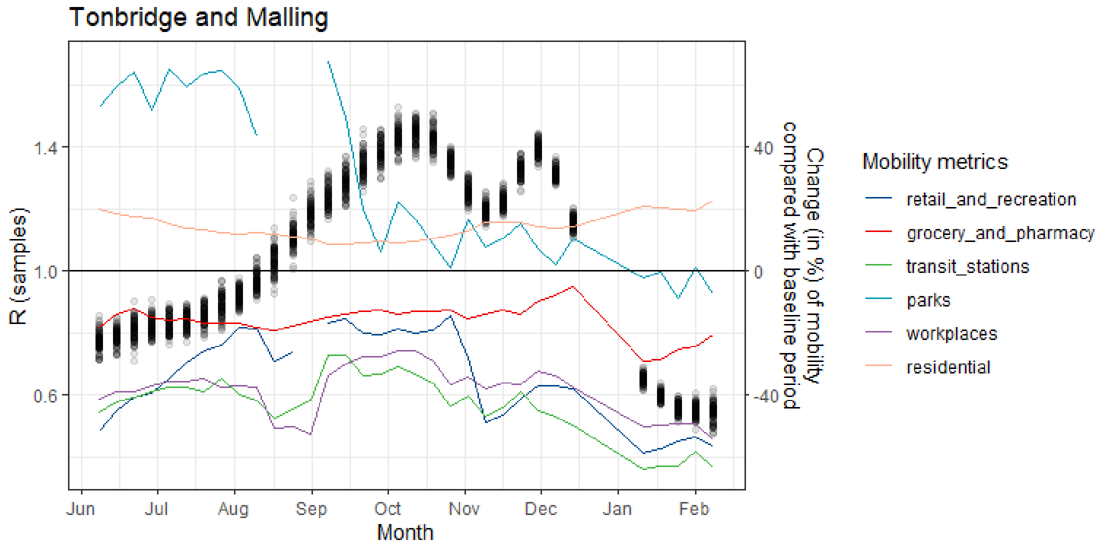

Tonbridge and Malling
R (samples)
1.4
1.0
0.6
Change (in %) of mobility
compared with baseline period
40
0
-40
Jun
Jul
Aug
Sep
Oct
Nov
Dec
Jan
Feb
Month
Mobility metrics
retail_and_recreation
grocery_and_pharmacy
transit_stations
parks
workplaces
residential

Torbay

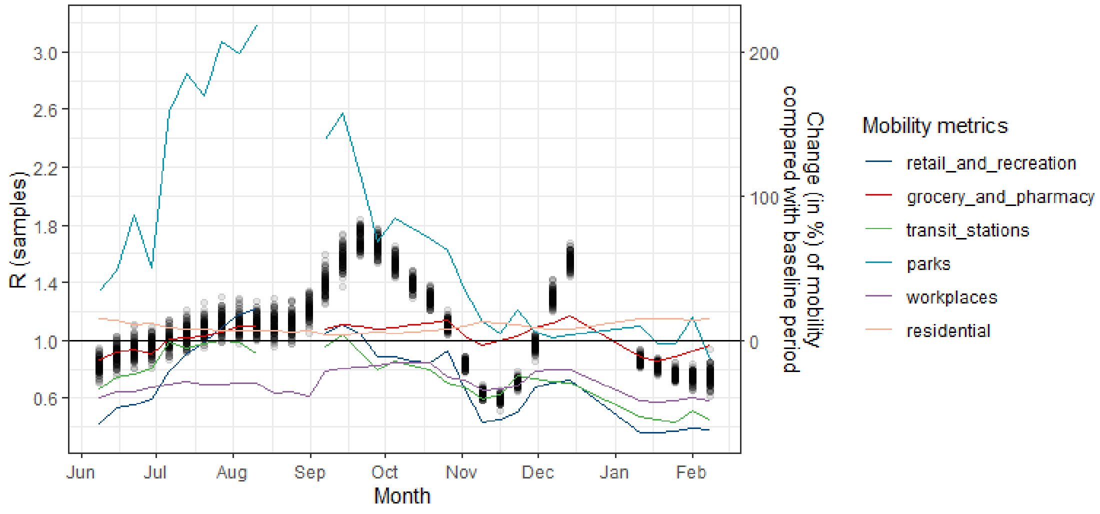

Torfaen

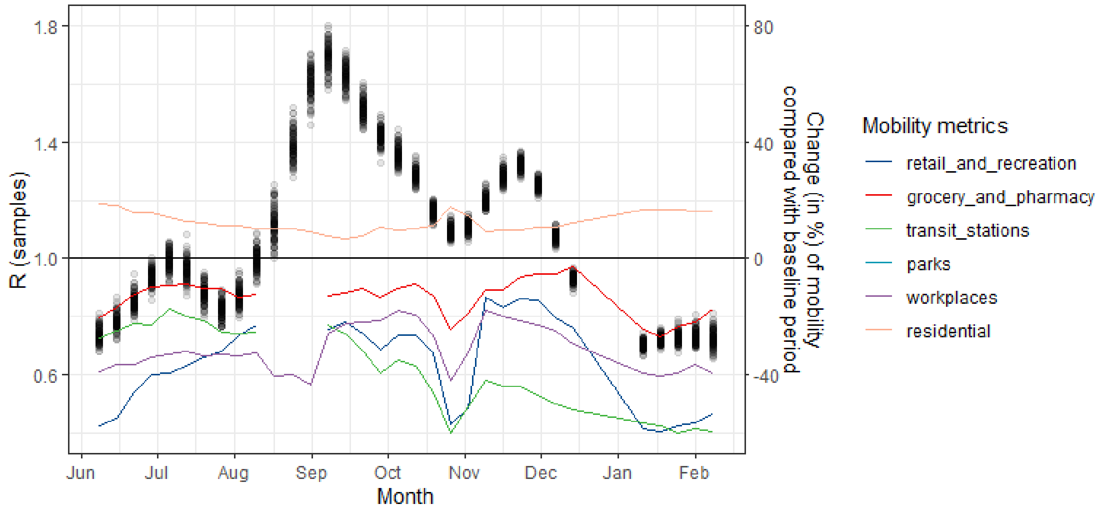

Torridge

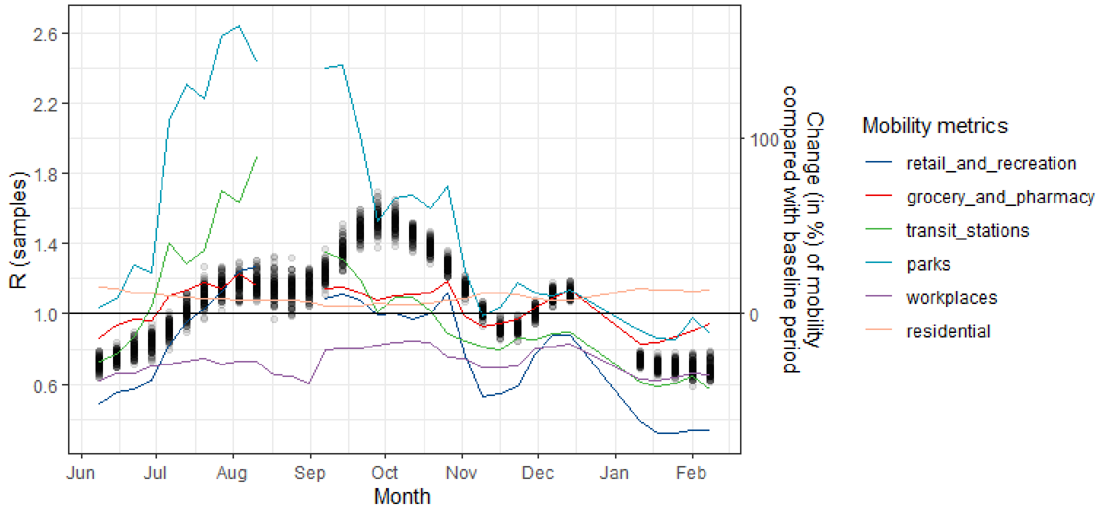

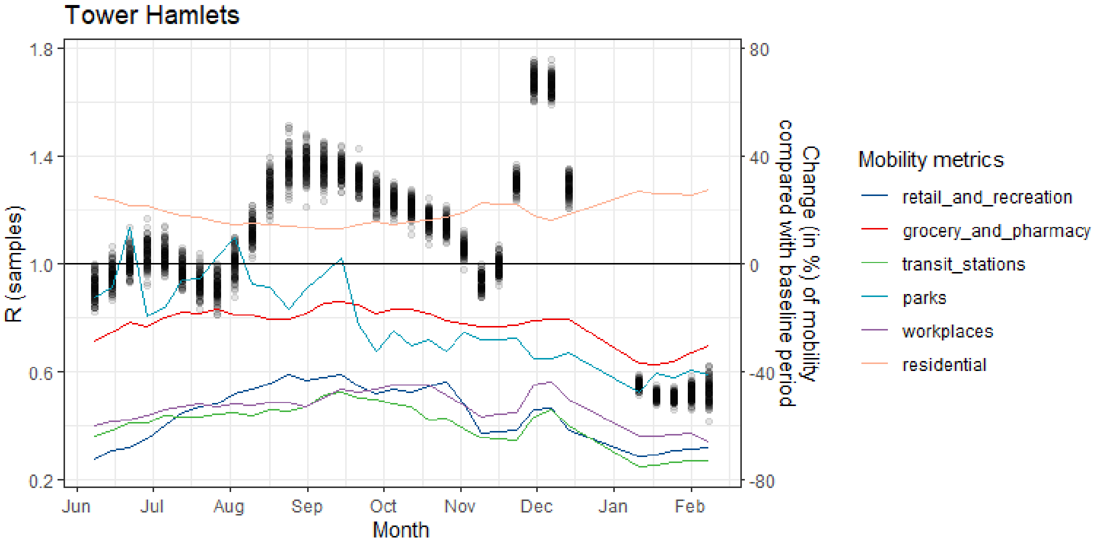
Tower Hamlets
R (samples)
1.8
1.4
1.0
0.6
0.2
Change (in %) of mobility compared with baseline period
80
40
0
-40
-80
Jun
Jul
Aug
Sep
Oct
Nov
Dec
Jan
Feb
Month
Mobility metrics
retail_and_recreation
grocery_and_pharmacy
transit_stations
parks
workplaces
residential

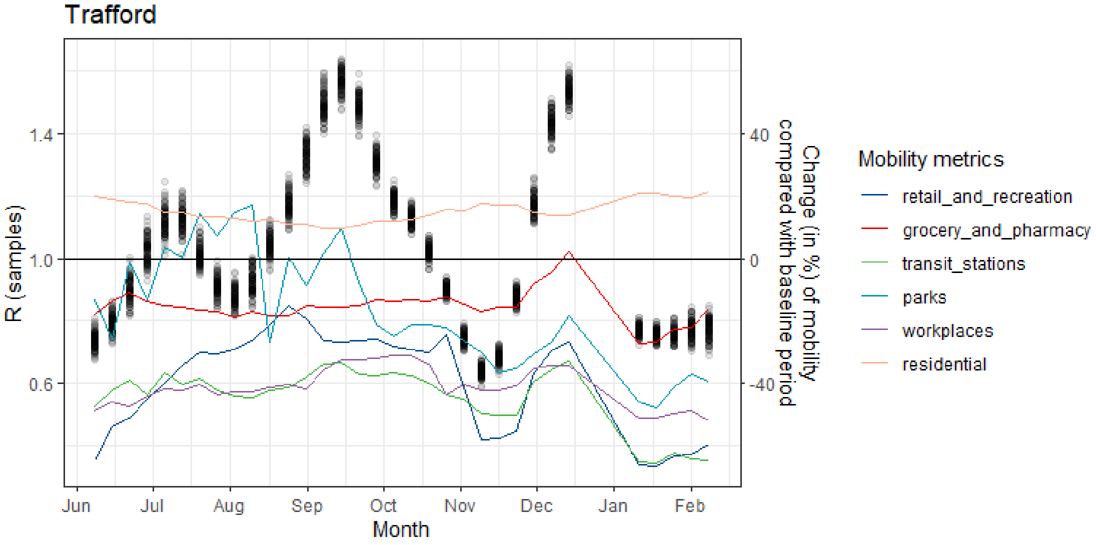
Trafford
R (samples)
1.4
1.0
0.6
Change (in %) of mobility compared with baseline period
40
0
-40
Jun
Jul
Aug
Sep
Oct
Nov
Dec
Jan
Feb
Month
Mobility metrics
retail_and_recreation
grocery_and_pharmacy
transit_stations
parks
workplaces
residential

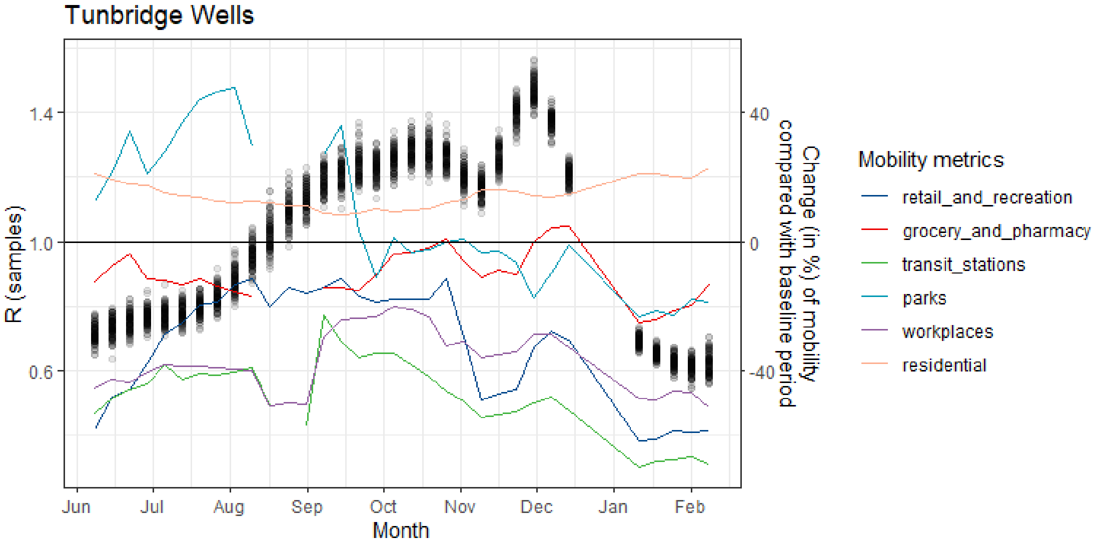
Tunbridge Wells
R (samples)
1.4
1.0
0.6
Change (in %) of mobility compared with baseline period
40
0
-40
Jun
Jul
Aug
Sep
Oct
Nov
Dec
Jan
Feb
Month
Mobility metrics
retail_and_recreation
grocery_and_pharmacy
transit_stations
parks
workplaces
residential

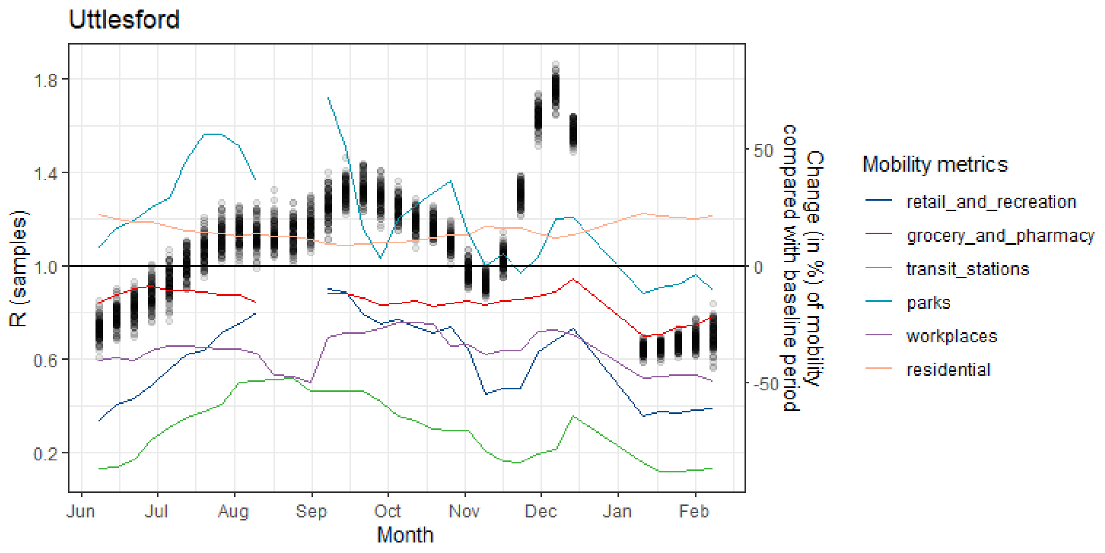
Uttlesford
1.8
1.4
1.0
0.6
0.2
R (samples)
50
0
-50
Change (in %) of mobility
compared with baseline period
Jun
Jul
Aug
Sep
Oct
Nov
Dec
Jan
Feb
Month
Mobility metrics
retail_and_recreation
grocery_and_pharmacy
transit_stations
parks
workplaces
residential

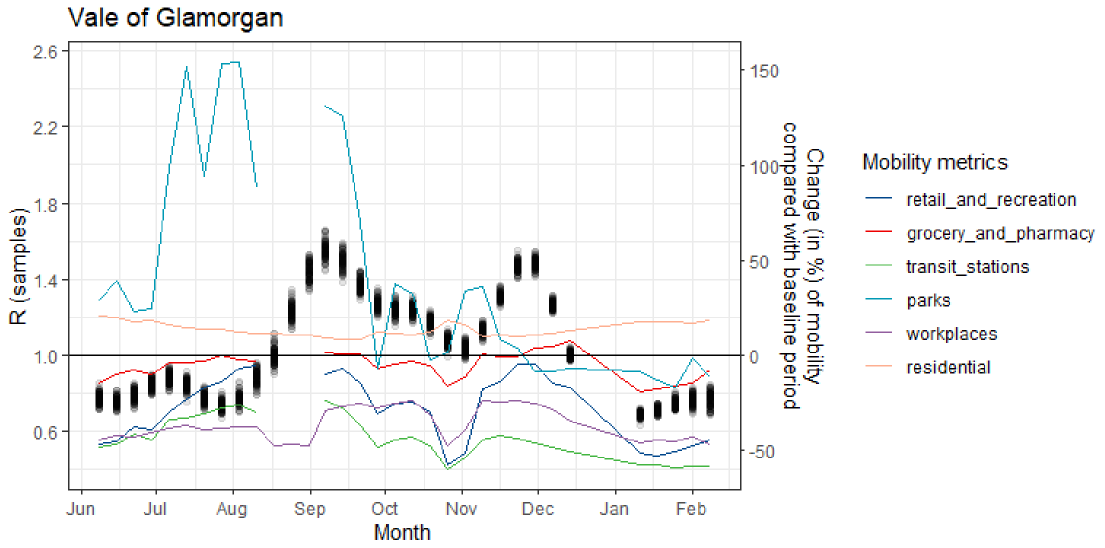
Vale of Glamorgan
2.6
2.2
1.8
1.4
1.0
0.6
R (samples)
150
100
50
0
-50
Change (in %) of mobility
compared with baseline period
Jun
Jul
Aug
Sep
Oct
Nov
Dec
Jan
Feb
Month
Mobility metrics
retail_and_recreation
grocery_and_pharmacy
transit_stations
parks
workplaces
residential

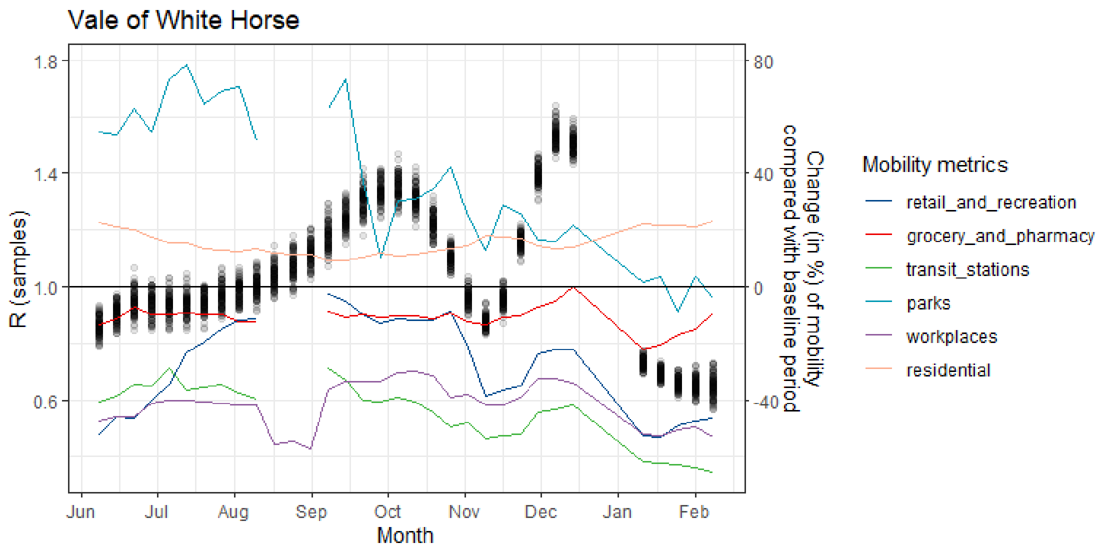
Vale of White Horse
1.8
1.4
1.0
0.6
R (samples)
80
40
0
-40
Change (in %) of mobility
compared with baseline period
Jun
Jul
Aug
Sep
Oct
Nov
Dec
Jan
Feb
Month
Mobility metrics
retail_and_recreation
grocery_and_pharmacy
transit_stations
parks
workplaces
residential

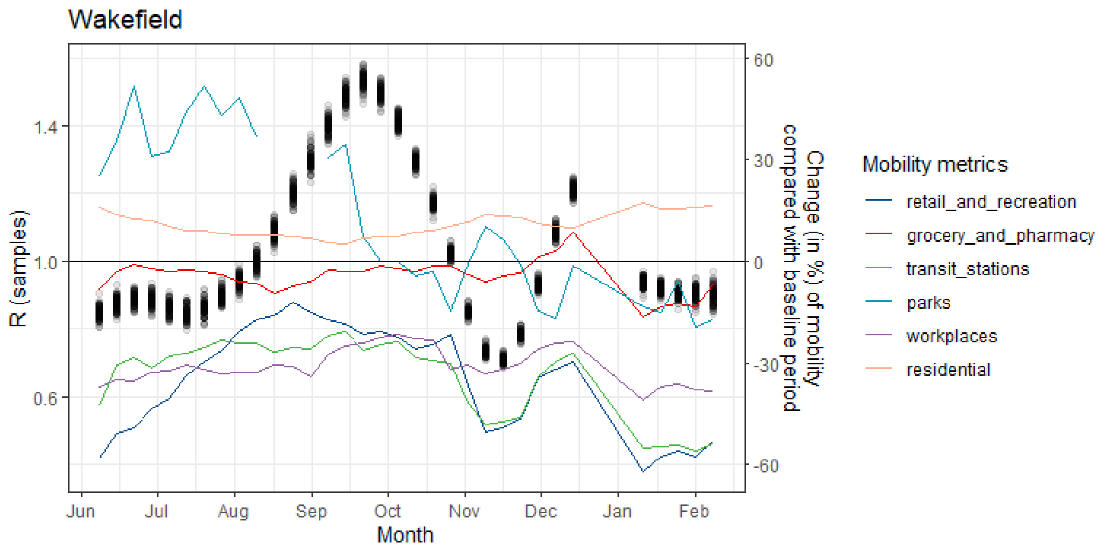
Wakefield
R (samples)
1.4
1.0
0.6
60
30
0
-30
-60
Change (in %) of mobility compared with baseline period
Jun
Jul
Aug
Sep
Oct
Nov
Dec
Jan
Feb
Month
Mobility metrics
retail_and_recreation
grocery_and_pharmacy
transit_stations
parks
workplaces
residential

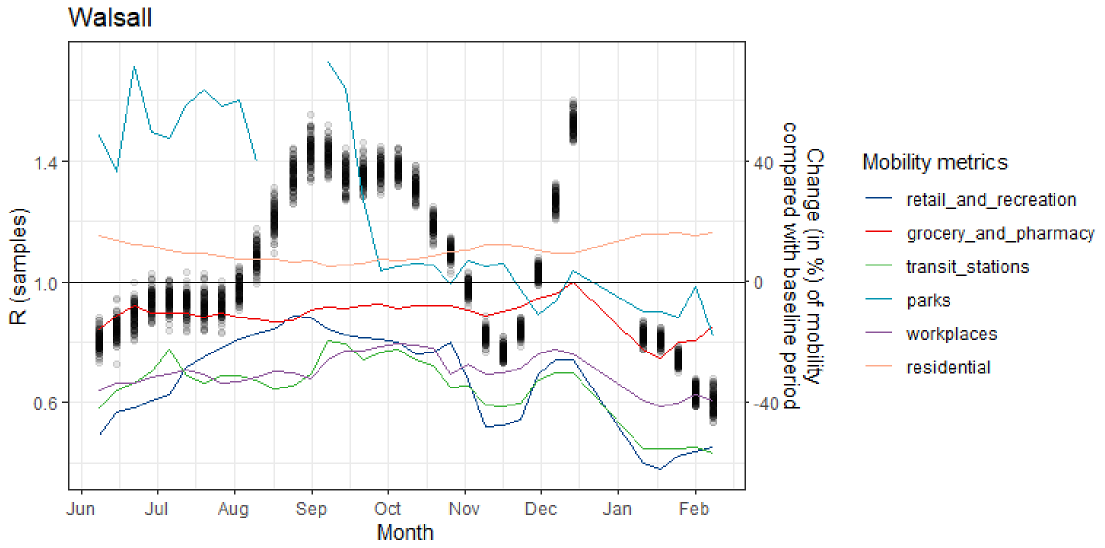
Walsall
R (samples)
1.4
1.0
0.6
40
0
-40
Change (in %) of mobility compared with baseline period
Jun
Jul
Aug
Sep
Oct
Nov
Dec
Jan
Feb
Month
Mobility metrics
retail_and_recreation
grocery_and_pharmacy
transit_stations
parks
workplaces
residential

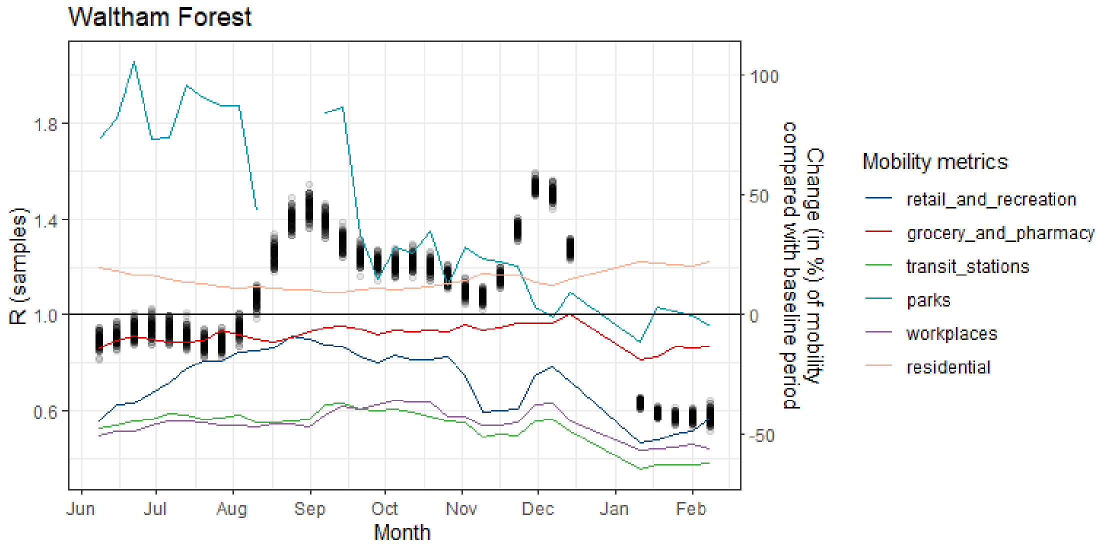
Waltham Forest
R (samples)
1.8
1.4
1.0
0.6
100
50
0
-50
Change (in %) of mobility compared with baseline period
Jun
Jul
Aug
Sep
Oct
Nov
Dec
Jan
Feb
Month
Mobility metrics
retail_and_recreation
grocery_and_pharmacy
transit_stations
parks
workplaces
residential

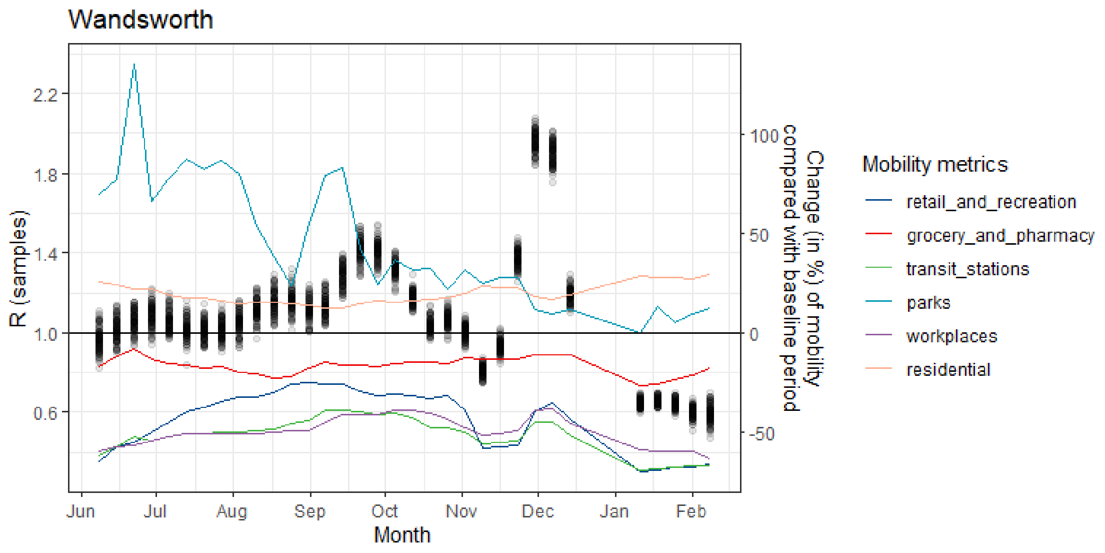
Wandsworth
R (samples)
Change (in %) of mobility compared with baseline period
Month
Jun Jul Aug Sep Oct Nov Dec Jan Feb
Mobility metrics
retail_and_recreation
grocery_and_pharmacy
transit_stations
parks
workplaces
residential

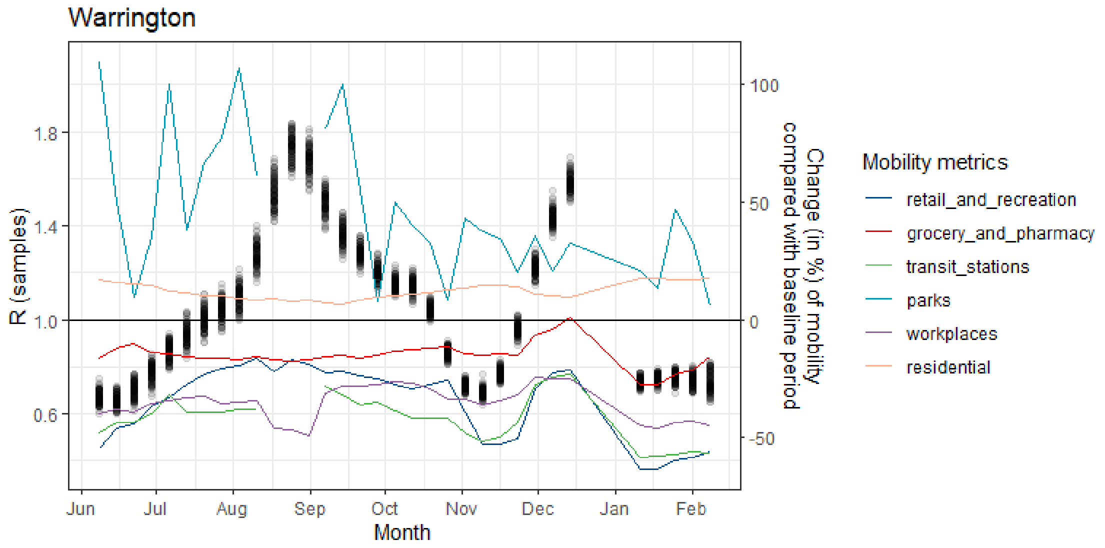
Warrington
R (samples)
Change (in %) of mobility compared with baseline period
Month
Jun Jul Aug Sep Oct Nov Dec Jan Feb
Mobility metrics
retail_and_recreation
grocery_and_pharmacy
transit_stations
parks
workplaces
residential

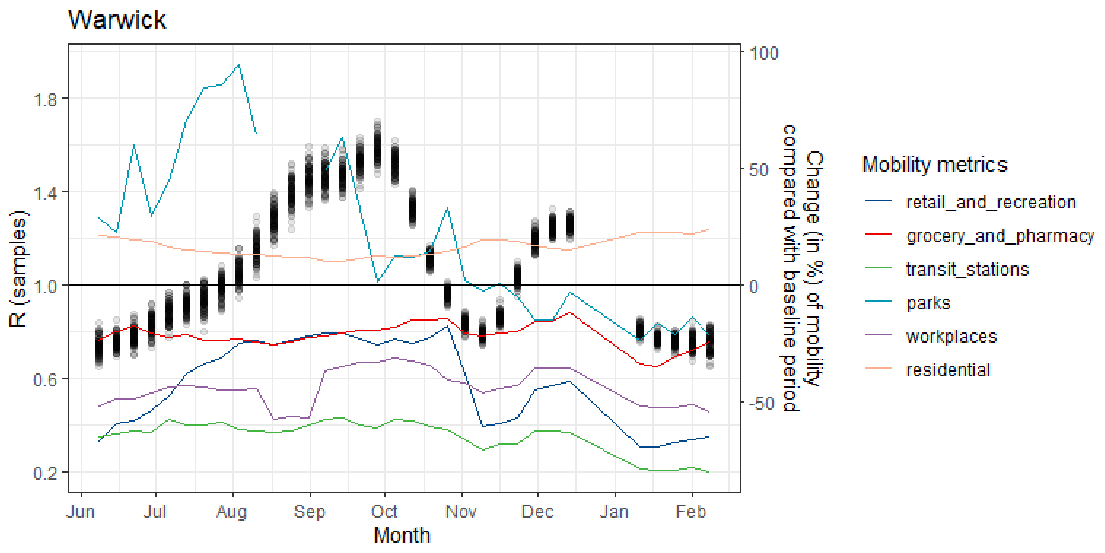
Warwick
R (samples)
Change (in %) of mobility compared with baseline period
Month
Jun Jul Aug Sep Oct Nov Dec Jan Feb
Mobility metrics
retail_and_recreation
grocery_and_pharmacy
transit_stations
parks
workplaces
residential

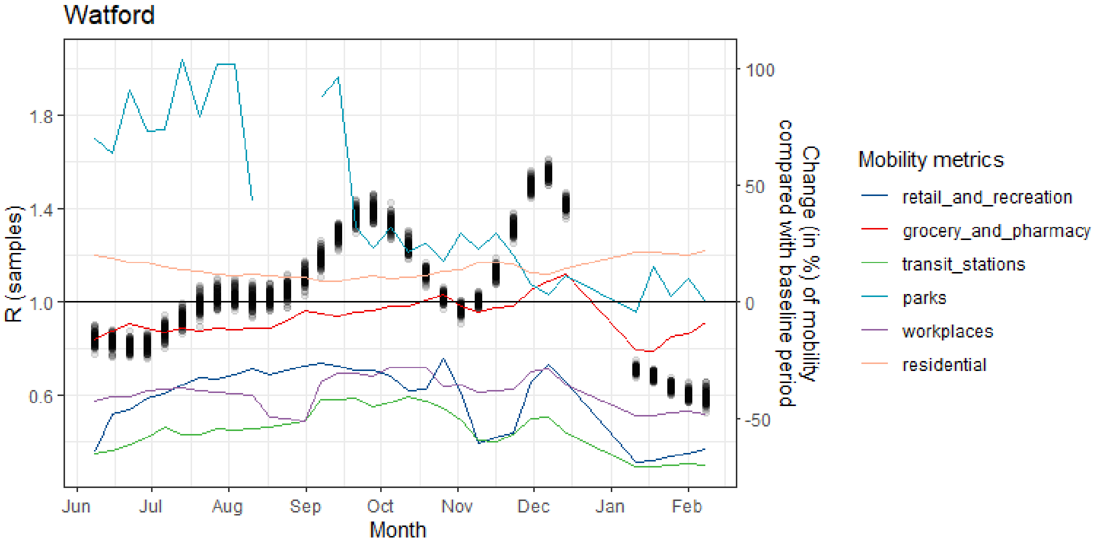
Watford
R (samples)
1.8
1.4
1.0
0.6
100
50
0
-50
Change (in %) of mobility compared with baseline period
Jun
Jul
Aug
Sep
Oct
Nov
Dec
Jan
Feb
Month
Mobility metrics
retail_and_recreation
grocery_and_pharmacy
transit_stations
parks
workplaces
residential

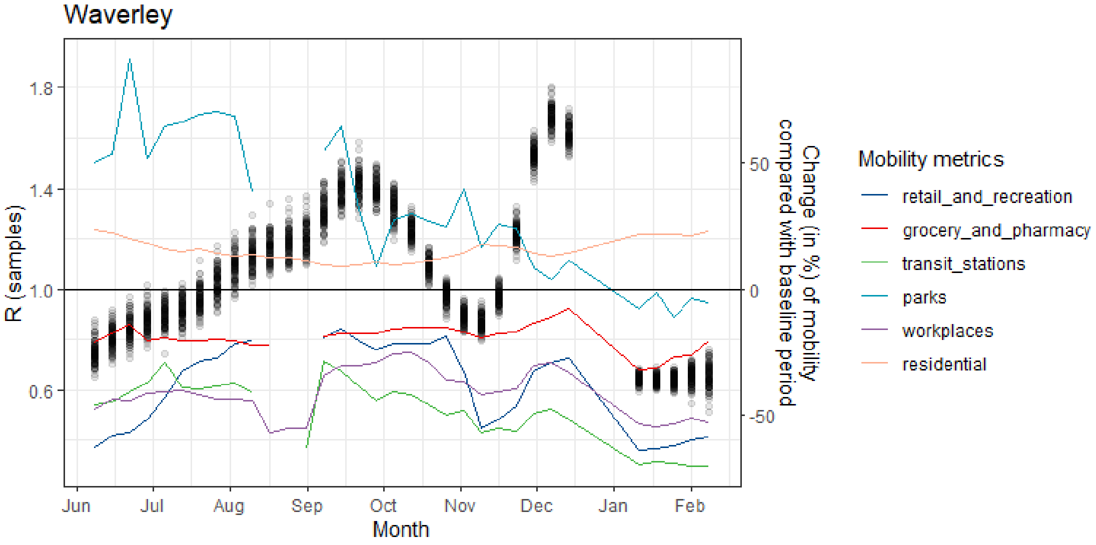
Waverley
R (samples)
1.8
1.4
1.0
0.6
50
0
-50
Change (in %) of mobility compared with baseline period
Jun
Jul
Aug
Sep
Oct
Nov
Dec
Jan
Feb
Month
Mobility metrics
retail_and_recreation
grocery_and_pharmacy
transit_stations
parks
workplaces
residential

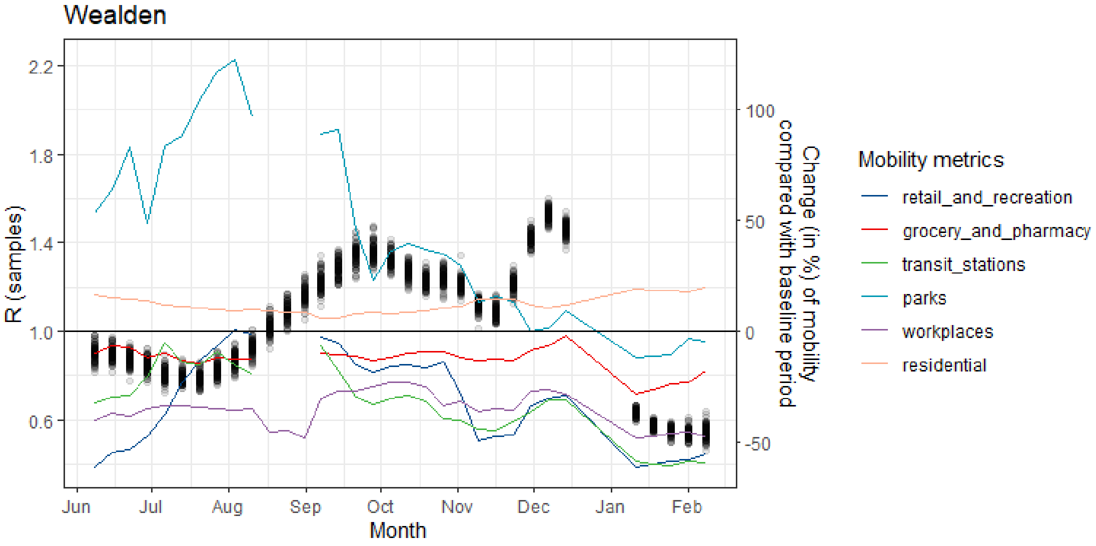
Wealden
R (samples)
2.2
1.8
1.4
1.0
0.6
100
50
0
-50
Change (in %) of mobility compared with baseline period
Jun
Jul
Aug
Sep
Oct
Nov
Dec
Jan
Feb
Month
Mobility metrics
retail_and_recreation
grocery_and_pharmacy
transit_stations
parks
workplaces
residential

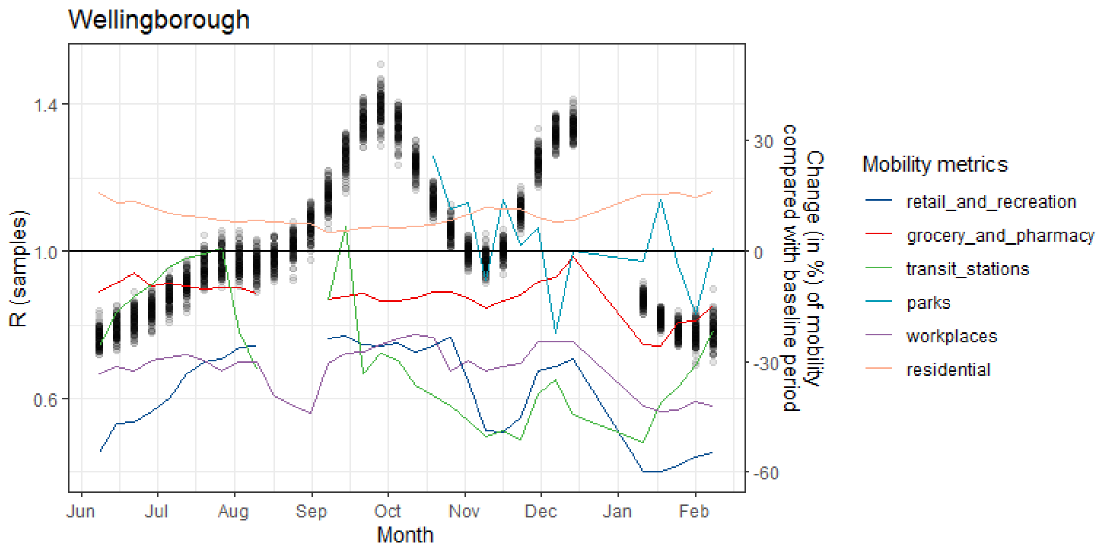
Wellingborough
R (samples)
1.4
1.0
0.6
30
0
-30
-60
Change (in %) of mobility compared with baseline period
Jun
Jul
Aug
Sep
Oct
Nov
Dec
Jan
Feb
Month
Mobility metrics
retail_and_recreation
grocery_and_pharmacy
transit_stations
parks
workplaces
residential

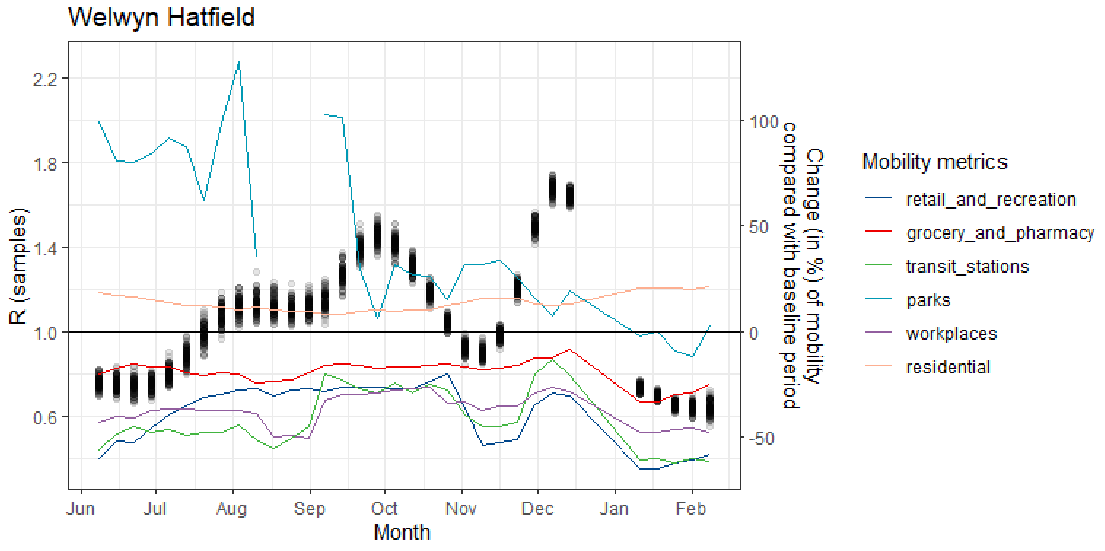
Welwyn Hatfield
R (samples)
2.2
1.8
1.4
1.0
0.6
100
50
0
-50
Change (in %) of mobility compared with baseline period
Jun
Jul
Aug
Sep
Oct
Nov
Dec
Jan
Feb
Month
Mobility metrics
retail_and_recreation
grocery_and_pharmacy
transit_stations
parks
workplaces
residential

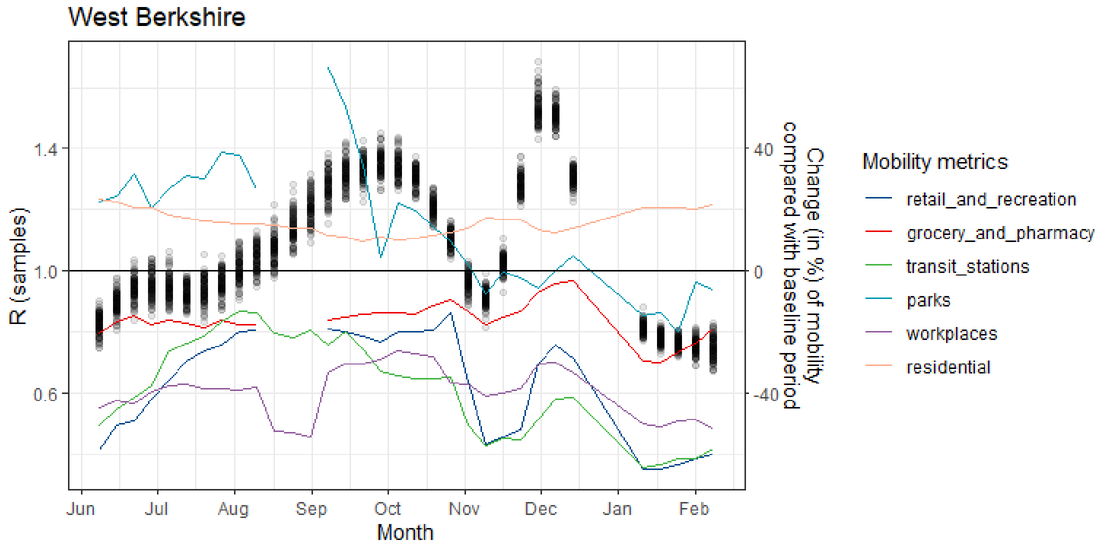
West Berkshire
R (samples)
1.4
1.0
0.6
40
0
-40
Change (in %) of mobility compared with baseline period
Jun
Jul
Aug
Sep
Oct
Nov
Dec
Jan
Feb
Month
Mobility metrics
retail_and_recreation
grocery_and_pharmacy
transit_stations
parks
workplaces
residential

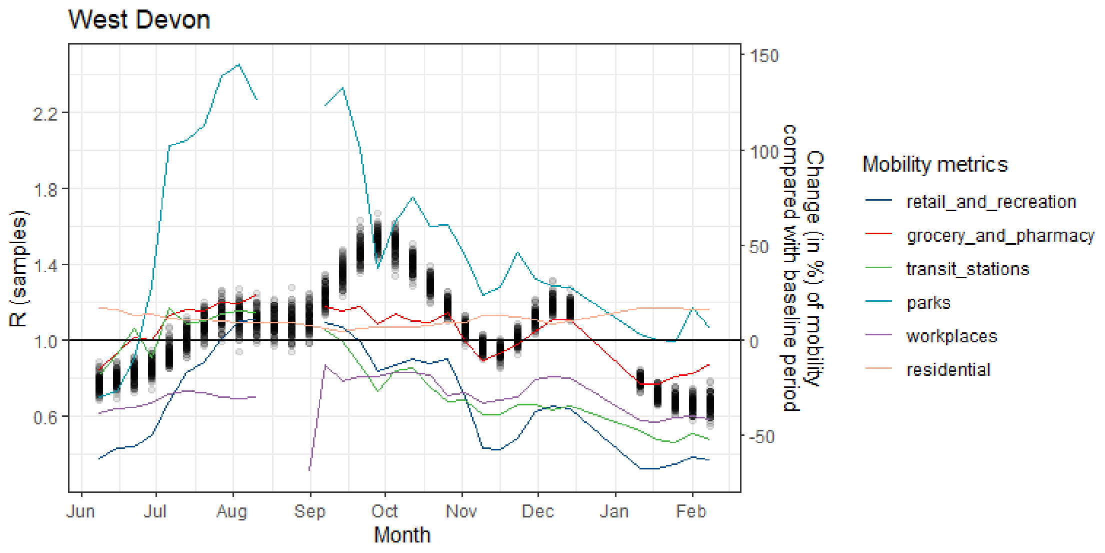
West Devon
R (samples)
2.2
1.8
1.4
1.0
0.6
150
100
50
0
-50
Change (in %) of mobility compared with baseline period
Jun
Jul
Aug
Sep
Oct
Nov
Dec
Jan
Feb
Month
Mobility metrics
retail_and_recreation
grocery_and_pharmacy
transit_stations
parks
workplaces
residential

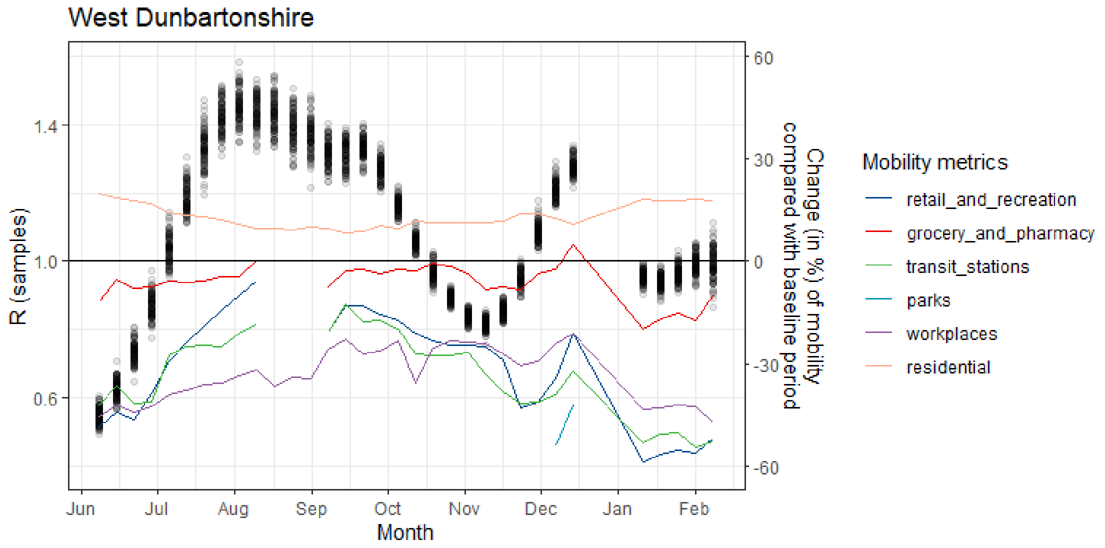
West Dunbartonshire
R (samples)
1.4
1.0
0.6
60
30
0
-30
-60
Change (in %) of mobility compared with baseline period
Jun
Jul
Aug
Sep
Oct
Nov
Dec
Jan
Feb
Month
Mobility metrics
retail_and_recreation
grocery_and_pharmacy
transit_stations
parks
workplaces
residential

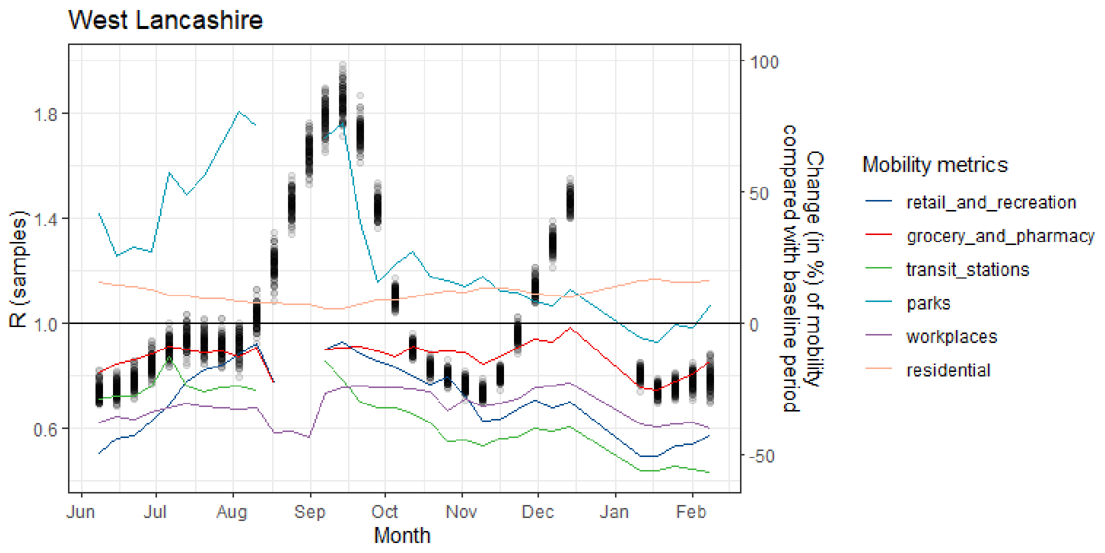
West Lancashire
R (samples)
1.8
1.4
1.0
0.6
100
50
0
-50
Change (in %) of mobility compared with baseline period
Jun
Jul
Aug
Sep
Oct
Nov
Dec
Jan
Feb
Month
Mobility metrics
retail_and_recreation
grocery_and_pharmacy
transit_stations
parks
workplaces
residential

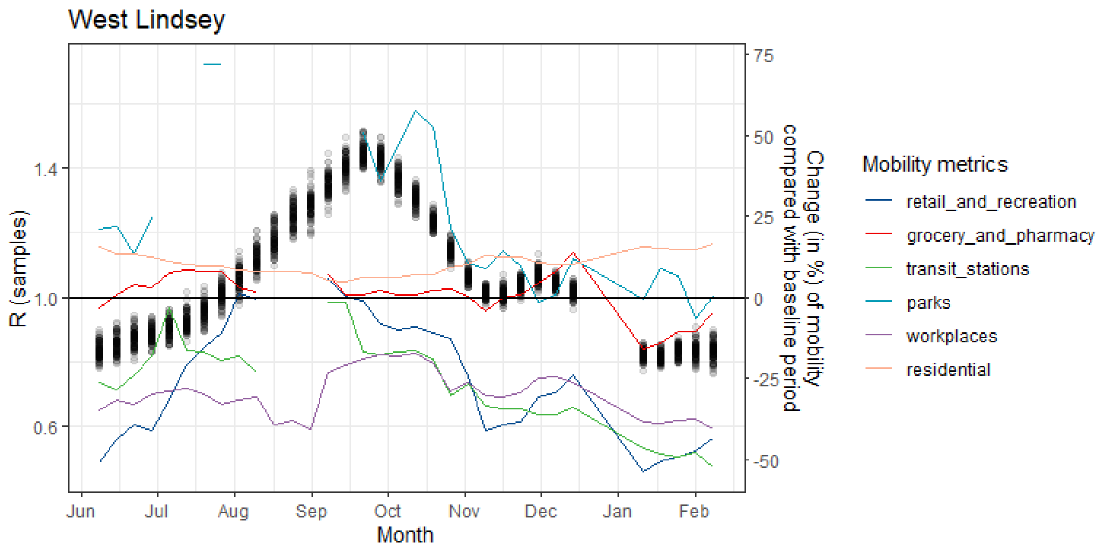
West Lindsey
R (samples)
1.4
1.0
0.6
75
50
25
0
-25
-50
Change (in %) of mobility compared with baseline period
Jun
Jul
Aug
Sep
Oct
Nov
Dec
Jan
Feb
Month
Mobility metrics
retail_and_recreation
grocery_and_pharmacy
transit_stations
parks
workplaces
residential

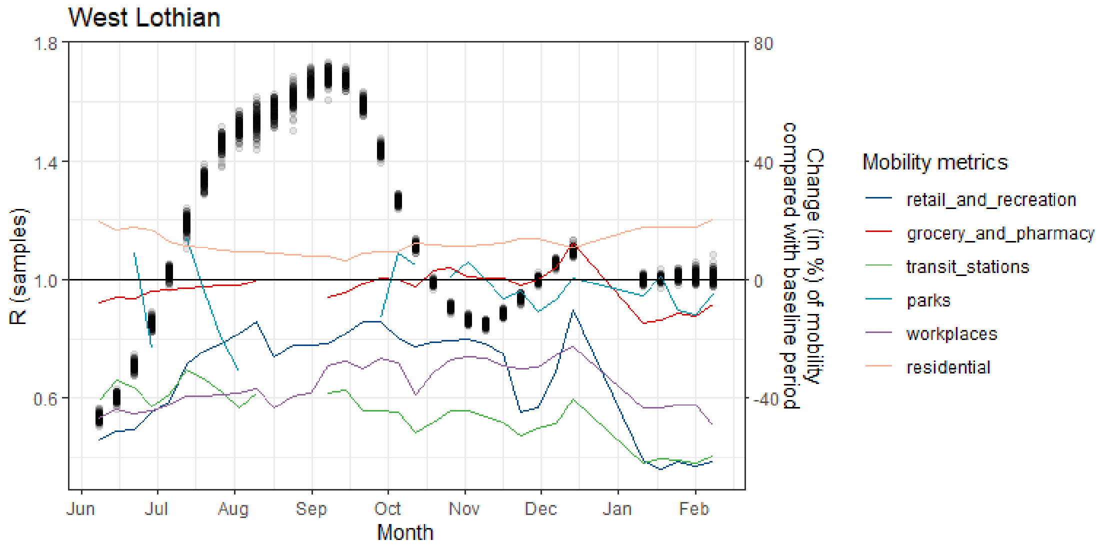
West Lothian
R (samples)
1.8
1.4
1.0
0.6
80
40
0
-40
Change (in %) of mobility compared with baseline period
Jun
Jul
Aug
Sep
Oct
Nov
Dec
Jan
Feb
Month
Mobility metrics
retail_and_recreation
grocery_and_pharmacy
transit_stations
parks
workplaces
residential

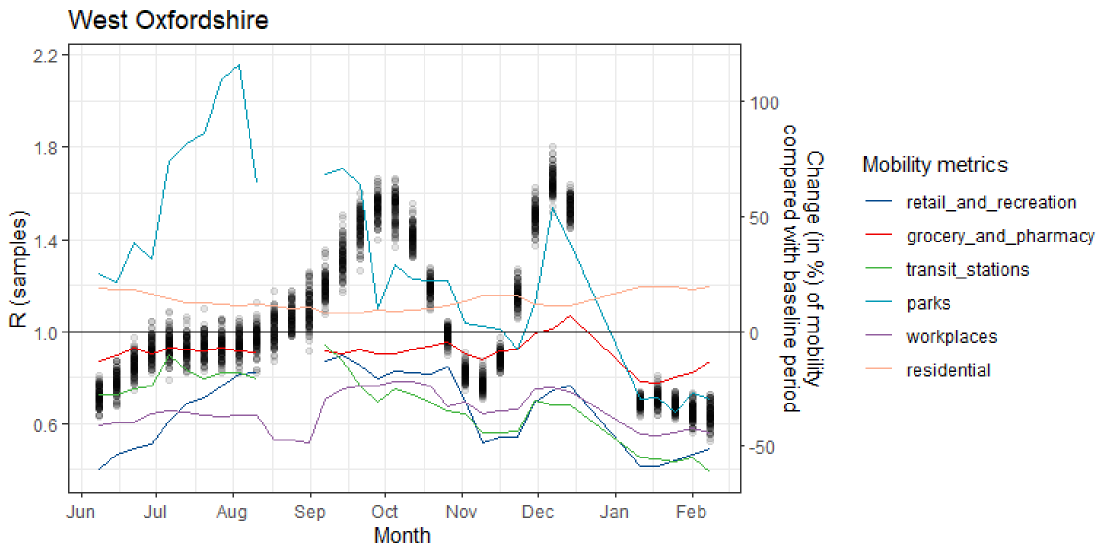
West Oxfordshire
R (samples)
2.2
1.8
1.4
1.0
0.6
100
50
0
-50
Change (in %) of mobility compared with baseline period
Jun
Jul
Aug
Sep
Oct
Nov
Dec
Jan
Feb
Month
Mobility metrics
retail_and_recreation
grocery_and_pharmacy
transit_stations
parks
workplaces
residential

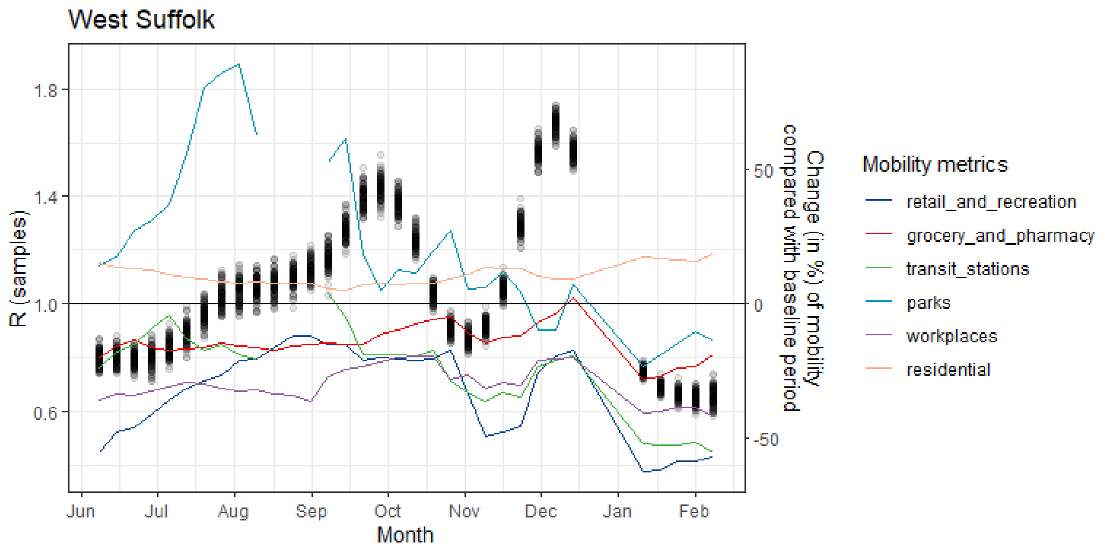
West Suffolk
R (samples)
1.8
1.4
1.0
0.6
Jun
Jul
Aug
Sep
Oct
Nov
Dec
Jan
Feb
Month
50
0
-50
Change (in %) of mobility compared with baseline period
Mobility metrics
retail_and_recreation
grocery_and_pharmacy
transit_stations
parks
workplaces
residential

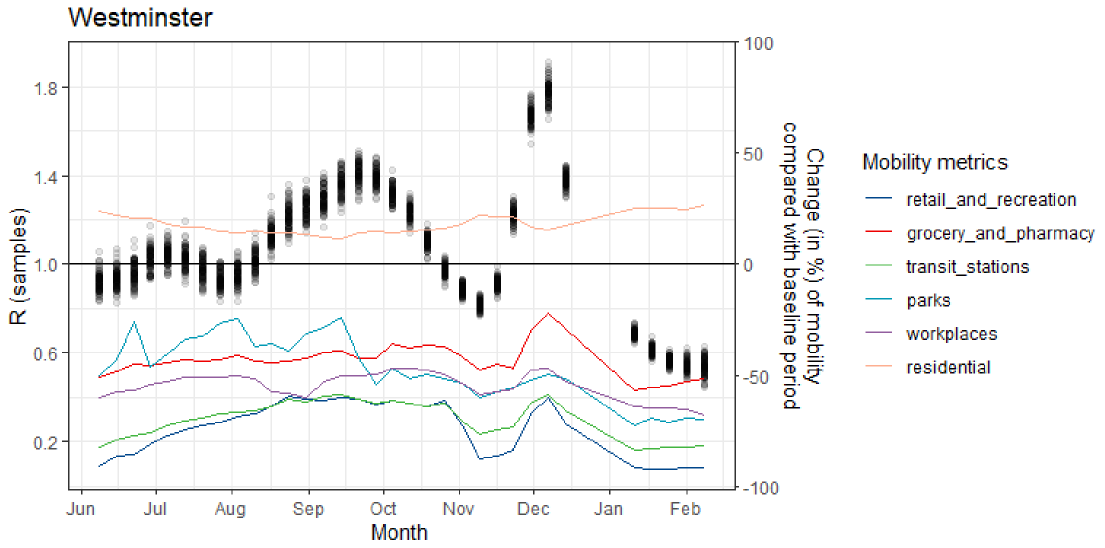
Westminster
R (samples)
1.8
1.4
1.0
0.6
0.2
Jun
Jul
Aug
Sep
Oct
Nov
Dec
Jan
Feb
Month
100
50
0
-50
-100
Change (in %) of mobility compared with baseline period
Mobility metrics
retail_and_recreation
grocery_and_pharmacy
transit_stations
parks
workplaces
residential

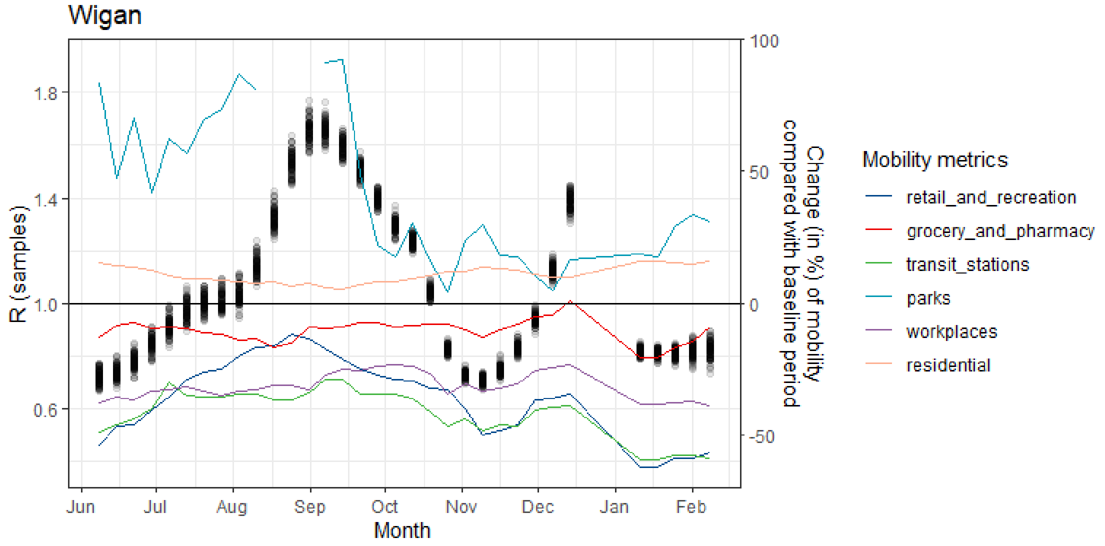
Wigan
R (samples)
1.8
1.4
1.0
0.6
Jun
Jul
Aug
Sep
Oct
Nov
Dec
Jan
Feb
Month
100
50
0
-50
Change (in %) of mobility compared with baseline period
Mobility metrics
retail_and_recreation
grocery_and_pharmacy
transit_stations
parks
workplaces
residential

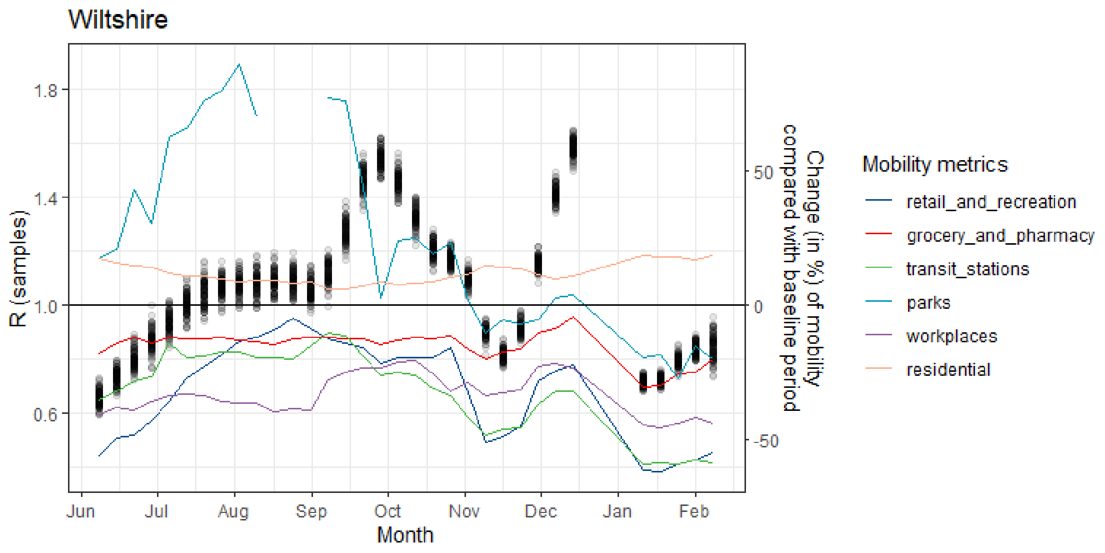
Wiltshire
R (samples)
1.8
1.4
1.0
0.6
Jun
Jul
Aug
Sep
Oct
Nov
Dec
Jan
Feb
Month
50
0
-50
Change (in %) of mobility compared with baseline period
Mobility metrics
retail_and_recreation
grocery_and_pharmacy
transit_stations
parks
workplaces
residential

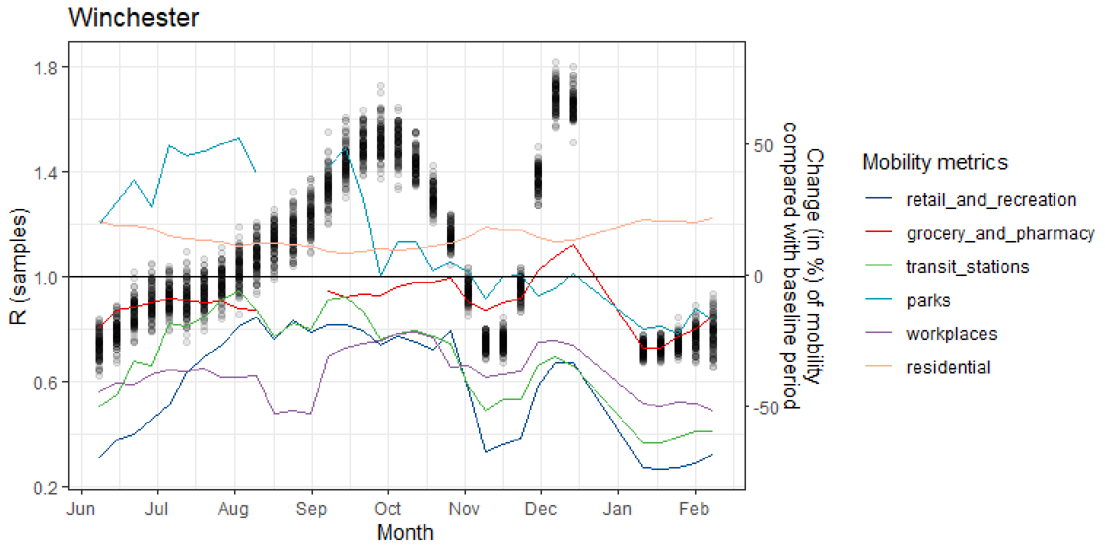
Winchester
R (samples)
1.8
1.4
1.0
0.6
0.2
Jun
Jul
Aug
Sep
Oct
Nov
Dec
Jan
Feb
Month
50
0
-50
Change (in %) of mobility compared with baseline period
Mobility metrics
retail_and_recreation
grocery_and_pharmacy
transit_stations
parks
workplaces
residential

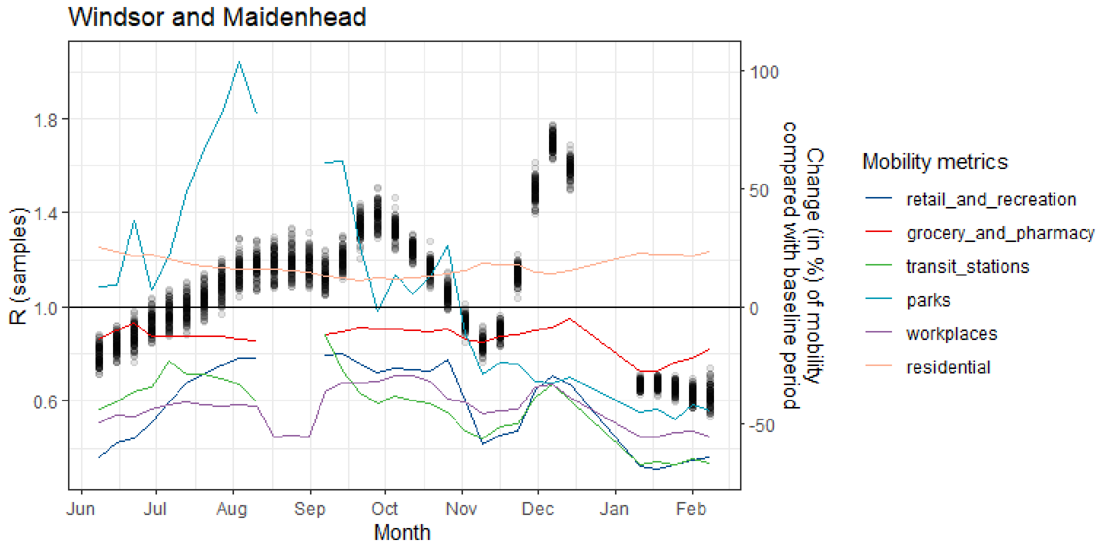
Windsor and Maidenhead
R (samples)
1.8
1.4
1.0
0.6
Jun
Jul
Aug
Sep
Oct
Nov
Dec
Jan
Feb
Month
100
50
0
-50
Change (in %) of mobility compared with baseline period
Mobility metrics
retail_and_recreation
grocery_and_pharmacy
transit_stations
parks
workplaces
residential

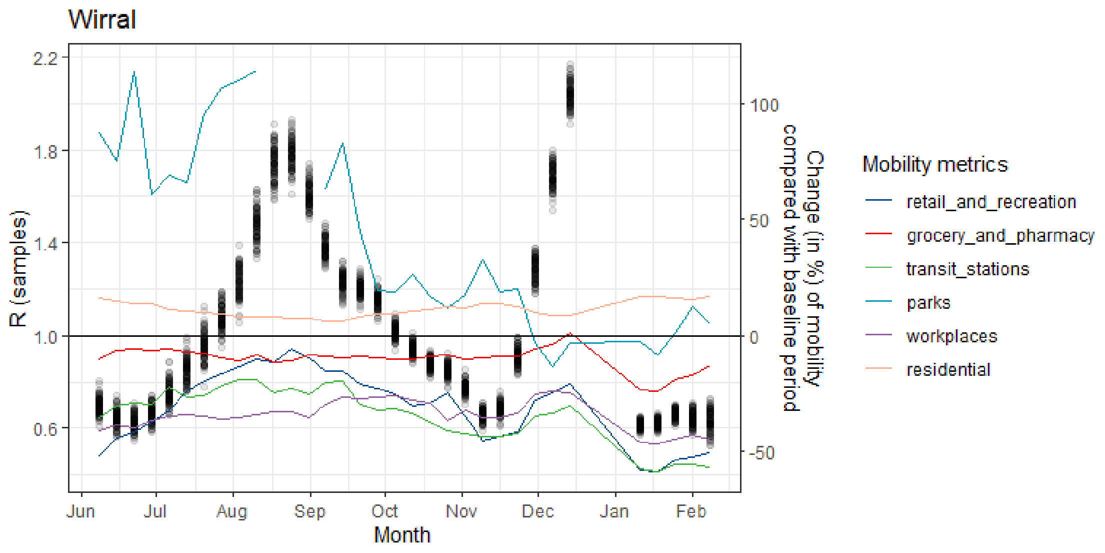
Wirral
R (samples)
2.2
1.8
1.4
1.0
0.6
Jun
Jul
Aug
Sep
Oct
Nov
Dec
Jan
Feb
Month
100
50
0
-50
Change (in %) of mobility compared with baseline period
Mobility metrics
retail_and_recreation
grocery_and_pharmacy
transit_stations
parks
workplaces
residential

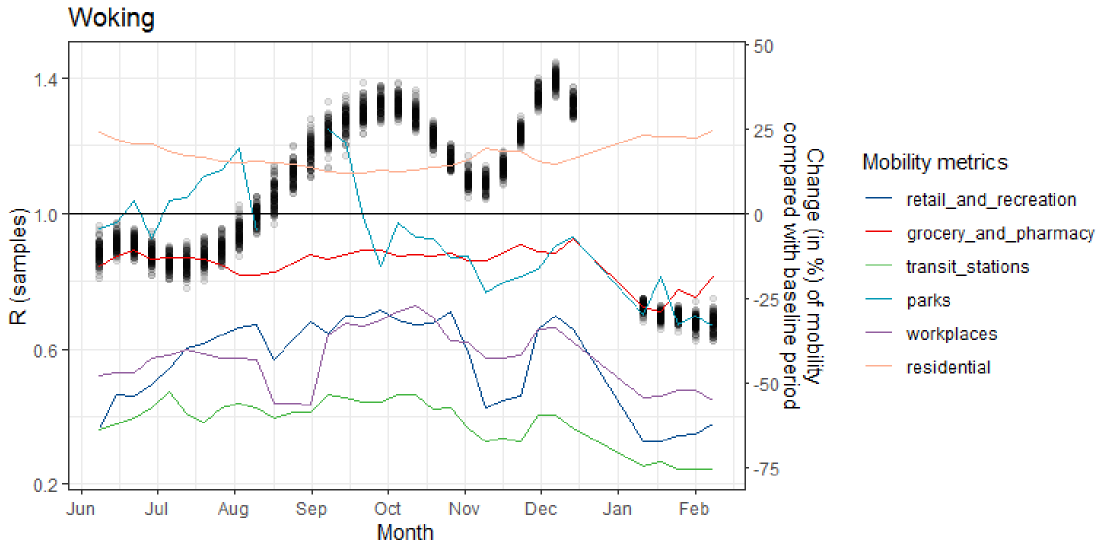
Woking
R (samples)
1.4
1.0
0.6
0.2
Jun
Jul
Aug
Sep
Oct
Nov
Dec
Jan
Feb
Month
50
25
0
-25
-50
-75
Change (in %) of mobility compared with baseline period
Mobility metrics
retail_and_recreation
grocery_and_pharmacy
transit_stations
parks
workplaces
residential

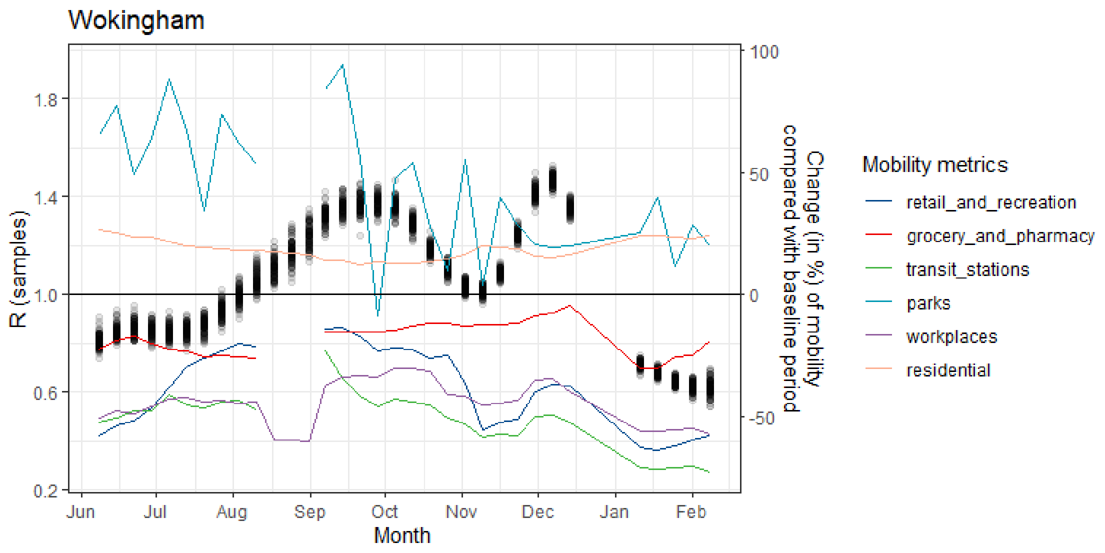
Wokingham
R (samples)
1.8
1.4
1.0
0.6
0.2
Jun
Jul
Aug
Sep
Oct
Nov
Dec
Jan
Feb
Month
100
50
0
-50
Change (in %) of mobility compared with baseline period
Mobility metrics
retail_and_recreation
grocery_and_pharmacy
transit_stations
parks
workplaces
residential

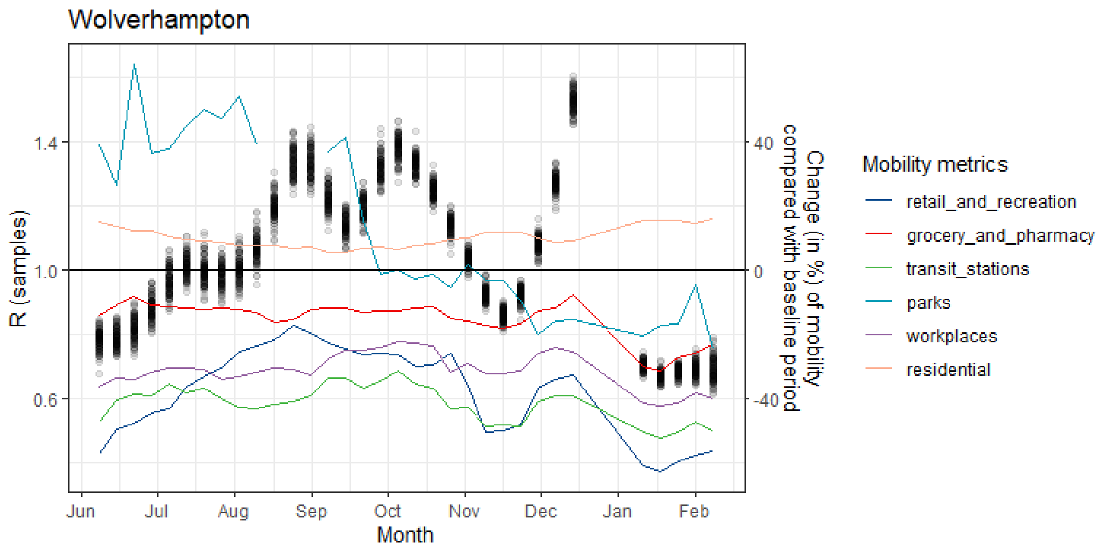
Wolverhampton
R (samples)
1.4
1.0
0.6
40
0
-40
Change (in %) of mobility compared with baseline period
Jun
Jul
Aug
Sep
Oct
Nov
Dec
Jan
Feb
Month
Mobility metrics
retail_and_recreation
grocery_and_pharmacy
transit_stations
parks
workplaces
residential

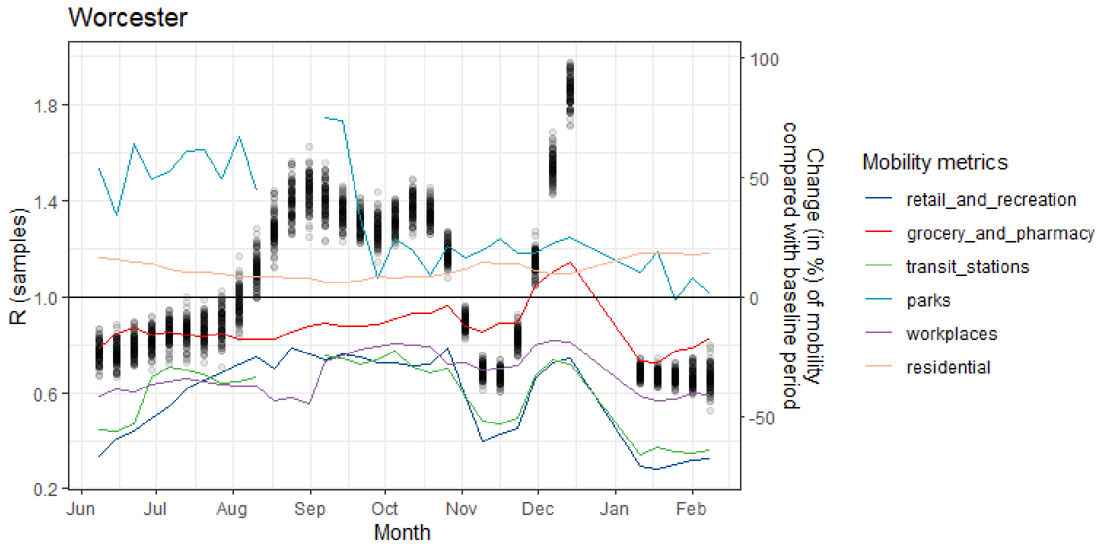
Worcester
R (samples)
1.8
1.4
1.0
0.6
0.2
100
50
0
-50
Change (in %) of mobility compared with baseline period
Jun
Jul
Aug
Sep
Oct
Nov
Dec
Jan
Feb
Month
Mobility metrics
retail_and_recreation
grocery_and_pharmacy
transit_stations
parks
workplaces
residential

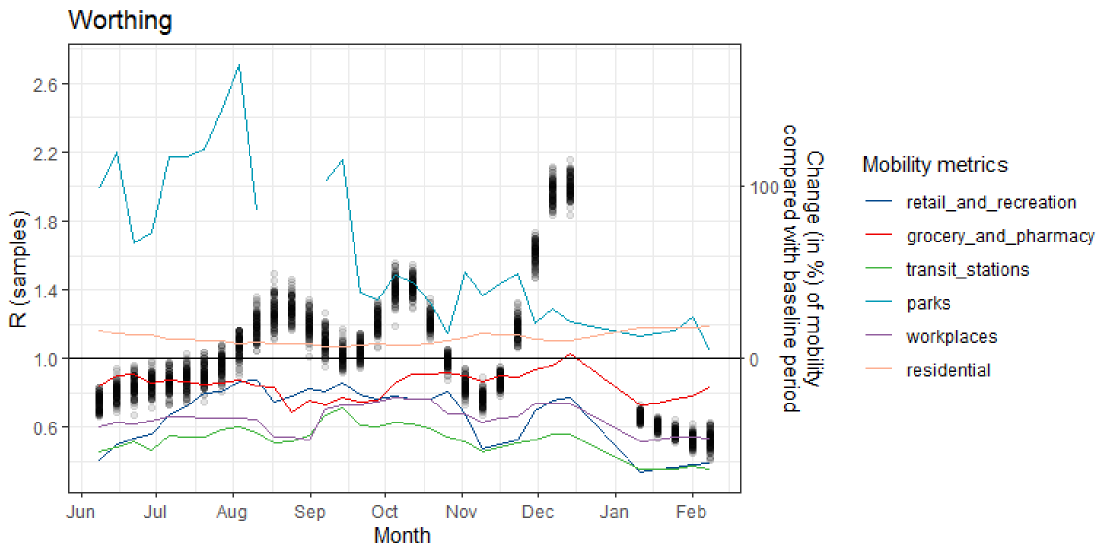
Worthing
R (samples)
2.6
2.2
1.8
1.4
1.0
0.6
100
0
Change (in %) of mobility compared with baseline period
Jun
Jul
Aug
Sep
Oct
Nov
Dec
Jan
Feb
Month
Mobility metrics
retail_and_recreation
grocery_and_pharmacy
transit_stations
parks
workplaces
residential

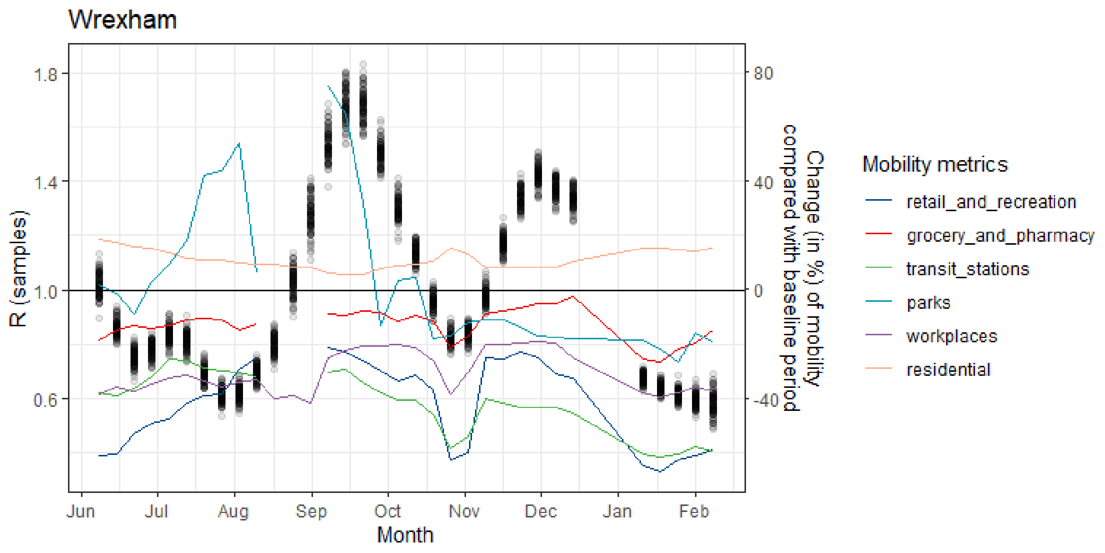
Wrexham
R (samples)
1.8
1.4
1.0
0.6
80
40
0
-40
Change (in %) of mobility compared with baseline period
Jun
Jul
Aug
Sep
Oct
Nov
Dec
Jan
Feb
Month
Mobility metrics
retail_and_recreation
grocery_and_pharmacy
transit_stations
parks
workplaces
residential

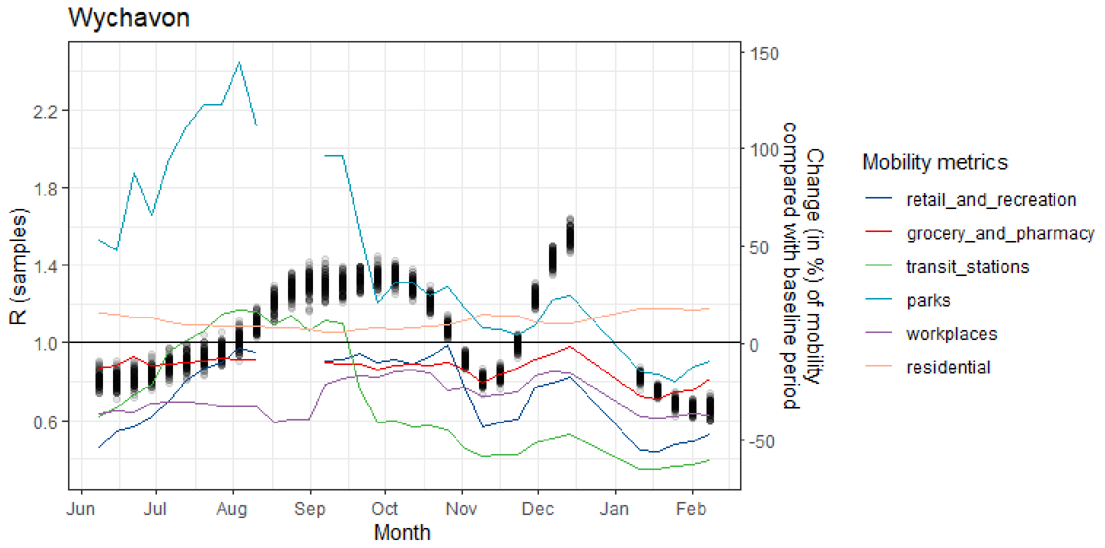
Wychavon
R (samples)
2.2
1.8
1.4
1.0
0.6
150
100
50
0
-50
Change (in %) of mobility compared with baseline period
Jun
Jul
Aug
Sep
Oct
Nov
Dec
Jan
Feb
Month
Mobility metrics
retail_and_recreation
grocery_and_pharmacy
transit_stations
parks
workplaces
residential

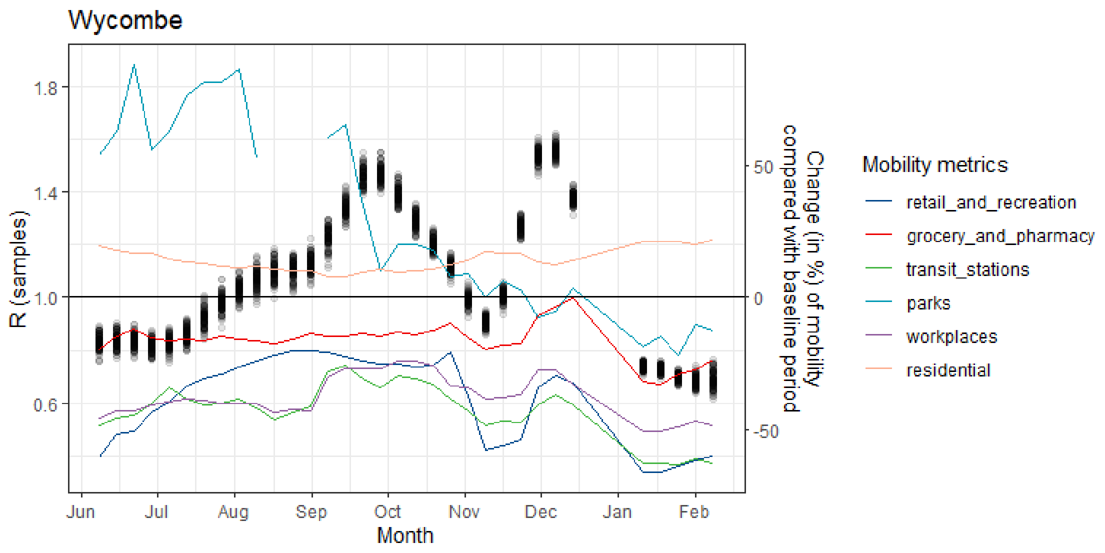
Wycombe
R (samples)
1.8
1.4
1.0
0.6
50
0
-50
Change (in %) of mobility compared with baseline period
Jun
Jul
Aug
Sep
Oct
Nov
Dec
Jan
Feb
Month
Mobility metrics
retail_and_recreation
grocery_and_pharmacy
transit_stations
parks
workplaces
residential

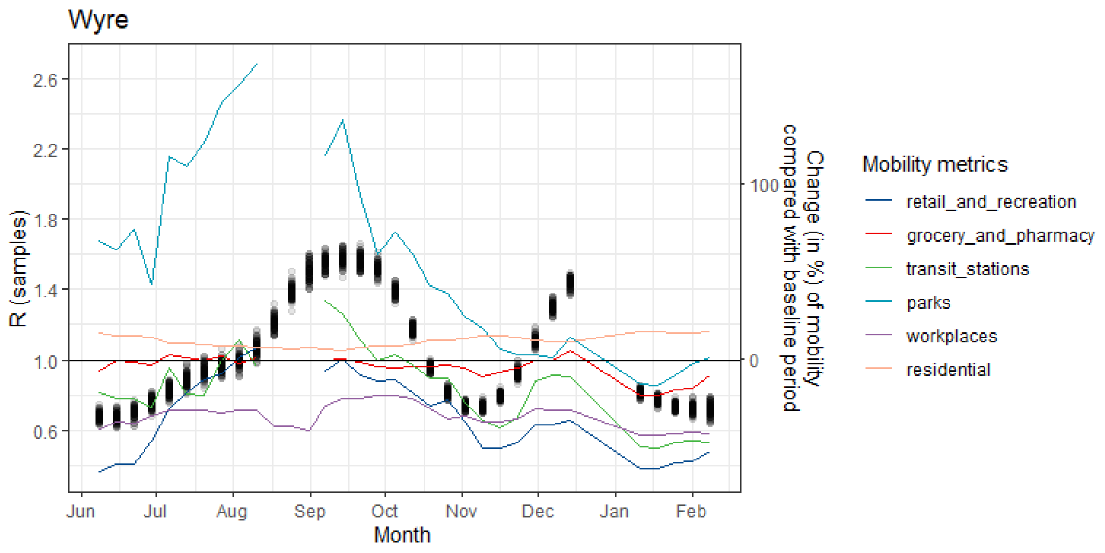

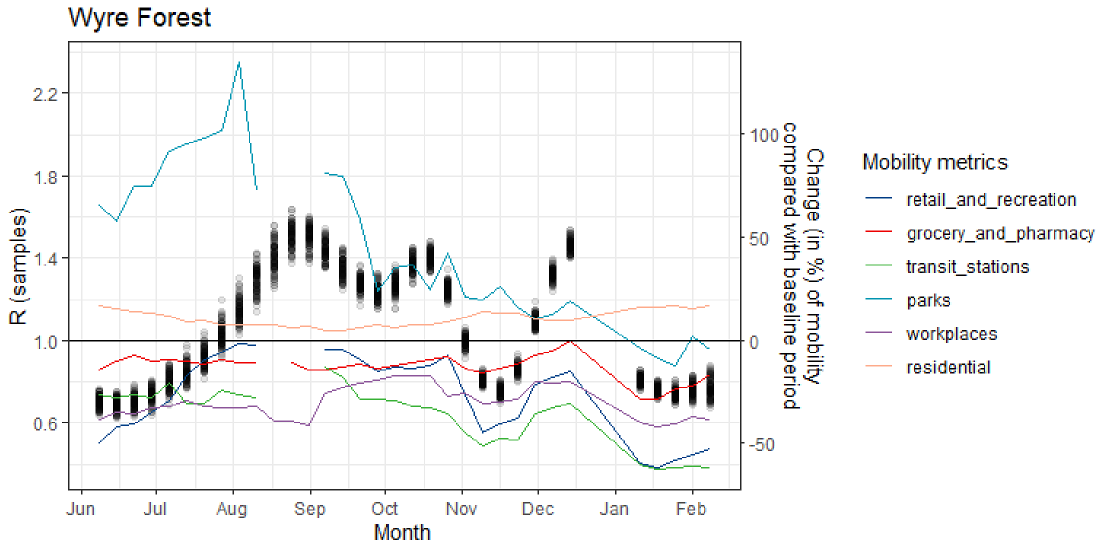

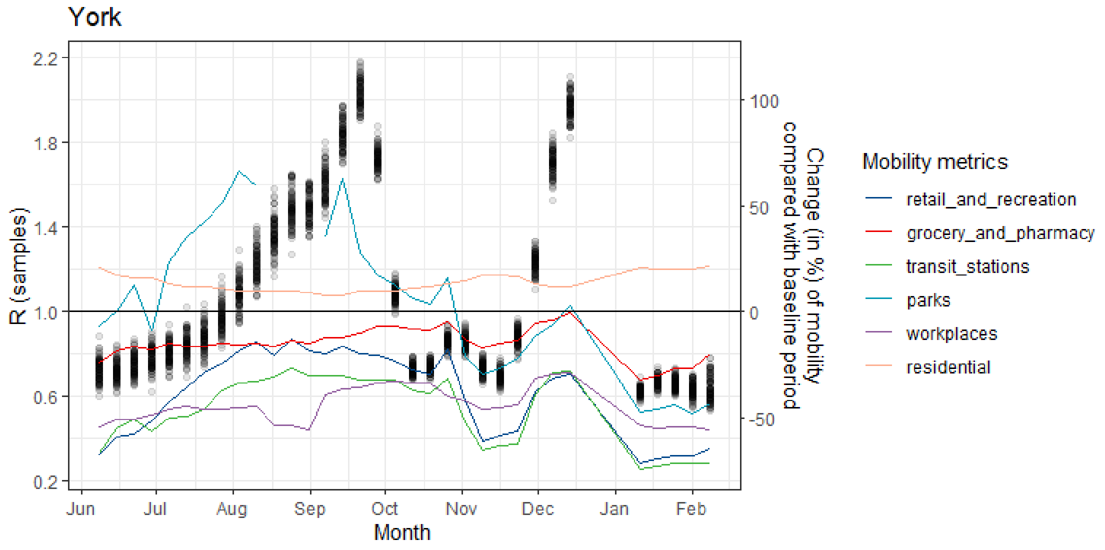

Black dots denote sampled R; R was sampled 100 times based on the 30%, 60% and 90% credible intervals of the model posteriors as detailed in **Text S1**.

Figure S2. Comparisons of findings from the main and sensitivity analyses

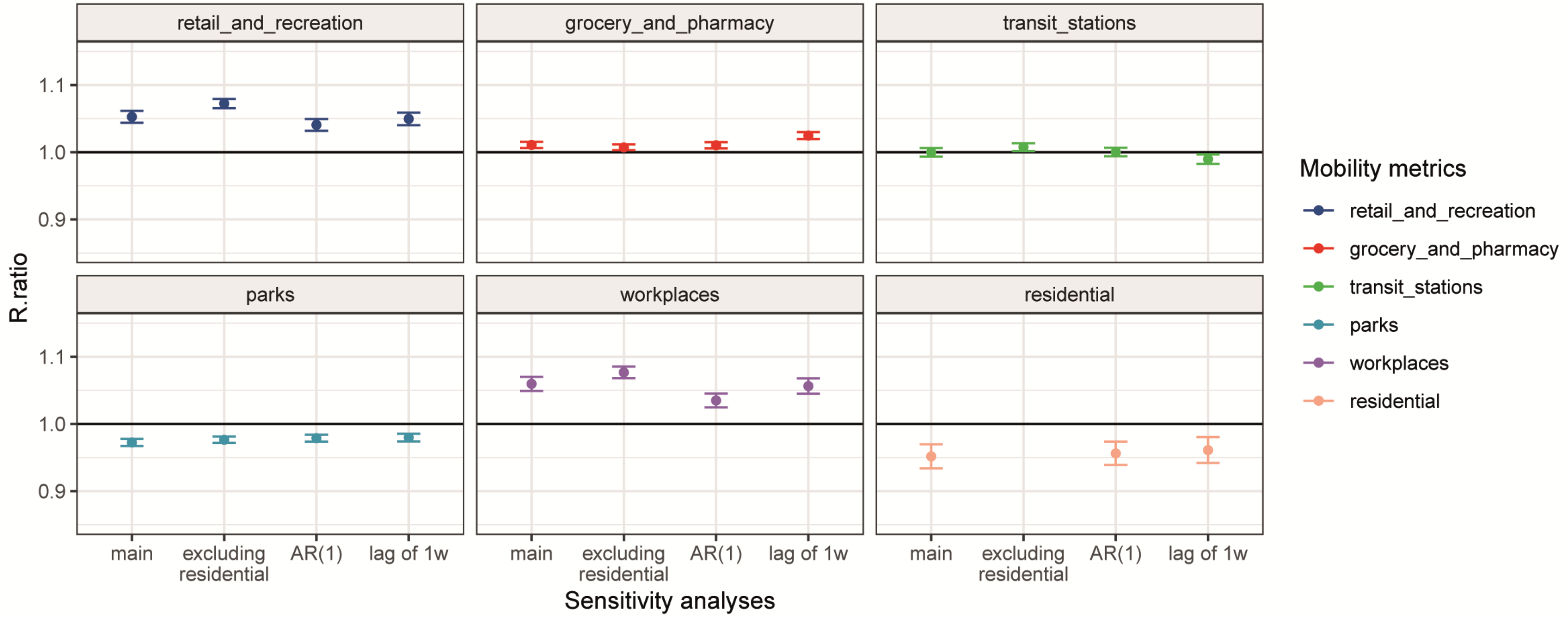

Main = the main model; excluding residential = the sensitivity analysis that excluded the mobility metric of residential stay from the main model; AR(1) = the sensitivity analysis that applied an autoregressive(1) error term; lag of 1w = the sensitivity analysis that applied one week of lag to the effect of changes in mobility metrics on changes in R.

Figure S3. Change over time in the association between visits to retail and recreation places and R by region

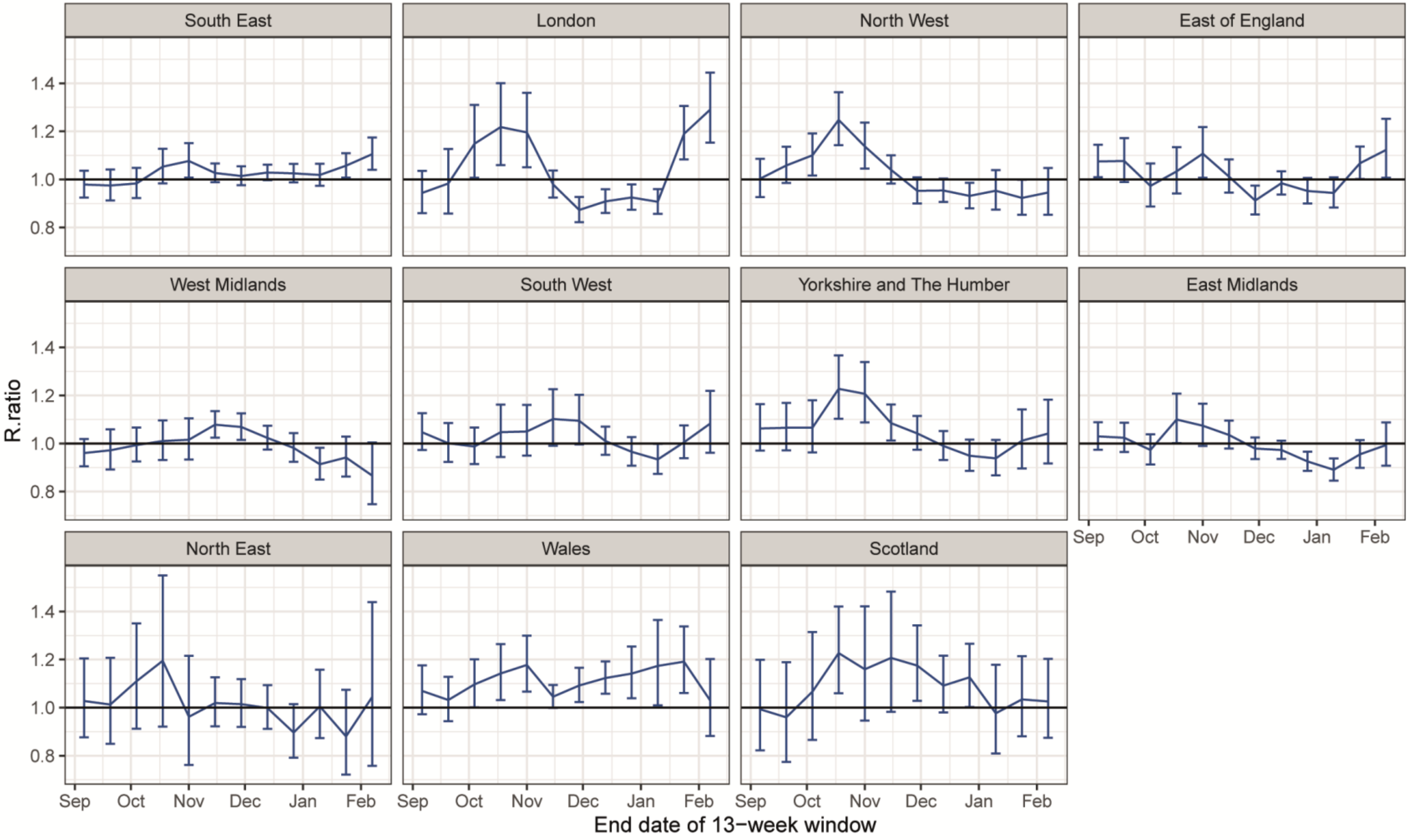

The number by x-axis denotes the number of local authorities included in the analysis. The three weeks of the Christmas and New Year festive period (i.e. between 21st Dec 2020 and 10th Jan 2021) were excluded from the analysis. Northern Ireland was not included in the analysis due to insufficient data.

Figure S4. Change over time in the association between visits to workplaces and R by region

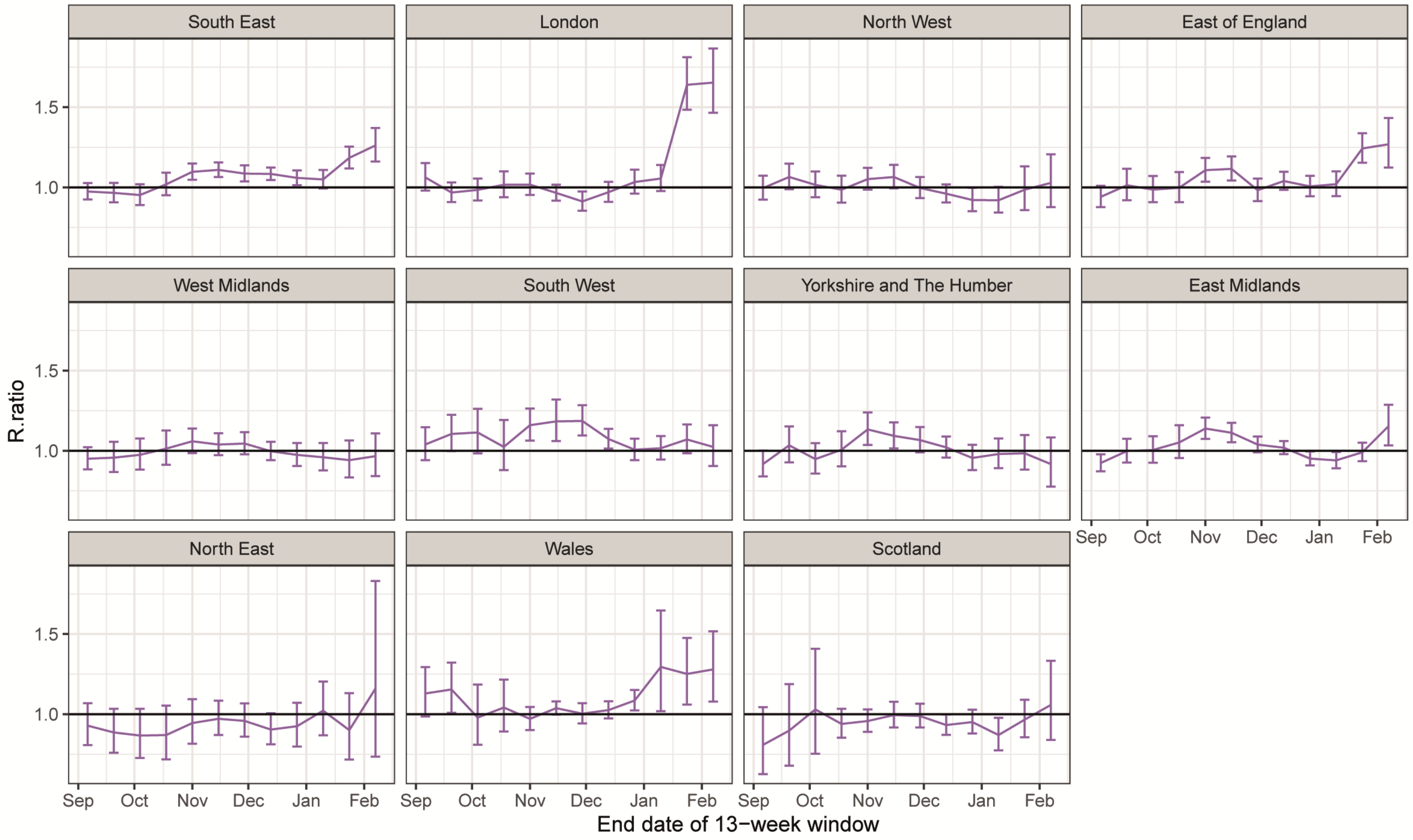

The number by x-axis denotes the number of local authorities included in the analysis. The three weeks of the Christmas and New Year festive period (i.e. between 21st Dec 2020 and 10th Jan 2021) were excluded from the analysis. Northern Ireland was not included in the analysis due to insufficient data.

Figure S5. Change over time in the association between time length spent at residential areas and R by region

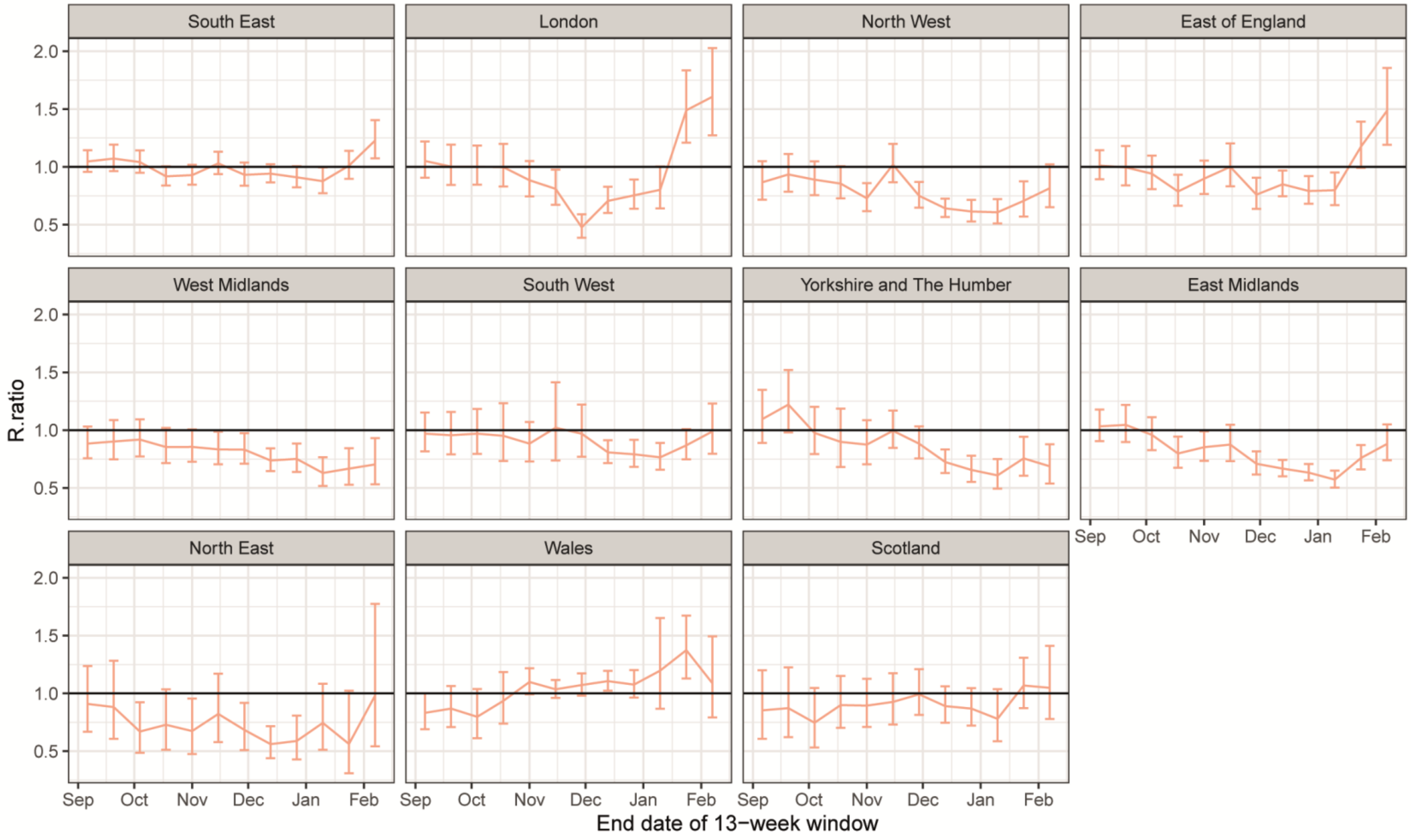

The number by x-axis denotes the number of local authorities included in the analysis. The three weeks of the Christmas and New Year festive period (i.e. between 21st Dec 2020 and 10th Jan 2021) were excluded from the analysis. Northern Ireland was not included in the analysis due to insufficient data.

STROBE checklist

STROBE Statement—checklist of items that should be included in reports of observational studies

	Item No	Recommendation	Page No
**Title and abstract**	1	(*a*) Indicate the study's design with a commonly used term in the title or the abstract	1
		(*b*) Provide in the abstract an informative and balanced summary of what was done and what was found	
**Introduction**			
Background/rationale	2	Explain the scientific background and rationale for the investigation being reported	3
Objectives	3	State specific objectives, including any prespecified hypotheses	3
**Methods**			
Study design	4	Present key elements of study design early in the paper	3
Setting	5	Describe the setting, locations, and relevant dates, including periods of recruitment, exposure, follow-up, and data collection	3-4
Participants	6	(*a*) *Cohort study*—Give the eligibility criteria, and the sources and methods of selection of participants. Describe methods of follow-up<br>*Case-control study*—Give the eligibility criteria, and the sources and methods of case ascertainment and control selection. Give the rationale for the choice of cases and controls<br>*Cross-sectional study*—Give the eligibility criteria, and the sources and methods of selection of participants	4
		(*b*) *Cohort study*—For matched studies, give matching criteria and number of exposed and unexposed<br>*Case-control study*—For matched studies, give matching criteria and the number of controls per case	
Variables	7	Clearly define all outcomes, exposures, predictors, potential confounders, and effect modifiers. Give diagnostic criteria, if applicable	4-5
Data sources/ measurement	8*	For each variable of interest, give sources of data and details of methods of assessment (measurement). Describe comparability of assessment methods if there is more than one group	3-4
Bias	9	Describe any efforts to address potential sources of bias	5
Study size	10	Explain how the study size was arrived at	NA
Quantitative variables	11	Explain how quantitative variables were handled in the analyses. If applicable, describe which groupings were chosen and why	4-5
Statistical methods	12	(*a*) Describe all statistical methods, including those used to control for confounding	4-5
		(*b*) Describe any methods used to examine subgroups and interactions	
		(*c*) Explain how missing data were addressed	
		(*d*) *Cohort study*—If applicable, explain how loss to follow-up was addressed<br>*Case-control study*—If applicable, explain how matching of cases and controls was addressed<br>*Cross-sectional study*—If applicable, describe analytical methods taking account of sampling strategy	
		(*e*) Describe any sensitivity analyses	

Continued on next page

**Results**			
Participants	13*	(a) Report numbers of individuals at each stage of study—eg numbers potentially eligible, examined for eligibility, confirmed eligible, included in the study, completing follow-up, and analysed	5
		(b) Give reasons for non-participation at each stage	
		(c) Consider use of a flow diagram	
Descriptive data	14*	(a) Give characteristics of study participants (eg demographic, clinical, social) and information on exposures and potential confounders	Figure S1
		(b) Indicate number of participants with missing data for each variable of interest	
		(c) *Cohort study*—Summarise follow-up time (eg, average and total amount)	
Outcome data	15*	*Cohort study*—Report numbers of outcome events or summary measures over time	Figure S1
		Case-control study—Report numbers in each exposure category, or summary measures of exposure	
		Cross-sectional study—Report numbers of outcome events or summary measures	
Main results	16	(*a*) Give unadjusted estimates and, if applicable, confounder-adjusted estimates and their precision (eg, 95% confidence interval). Make clear which confounders were adjusted for and why they were included	5-6
		(*b*) Report category boundaries when continuous variables were categorized	
		(*c*) If relevant, consider translating estimates of relative risk into absolute risk for a meaningful time period	
Other analyses	17	Report other analyses done—eg analyses of subgroups and interactions, and sensitivity analyses	6
**Discussion**			
Key results	18	Summarise key results with reference to study objectives	6
Limitations	19	Discuss limitations of the study, taking into account sources of potential bias or imprecision. Discuss both direction and magnitude of any potential bias	7-8
Interpretation	20	Give a cautious overall interpretation of results considering objectives, limitations, multiplicity of analyses, results from similar studies, and other relevant evidence	7
Generalisability	21	Discuss the generalisability (external validity) of the study results	8
**Other information**			
Funding	22	Give the source of funding and the role of the funders for the present study and, if applicable, for the original study on which the present article is based	5

*Give information separately for cases and controls in case-control studies and, if applicable, for exposed and unexposed groups in cohort and cross-sectional studies.

Note: An Explanation and Elaboration article discusses each checklist item and gives methodological background and published examples of transparent reporting. The STROBE checklist is best used in conjunction with this article (freely available on the Web sites of PLoS Medicine at http://www.plosmedicine.org/, Annals of Internal Medicine at http://www.annals.org/, and Epidemiology at http://www.epidem.com/). Information on the STROBE Initiative is available at www.strobe-statement.org.

References

1. Badr HS, Du H, Marshall M, Dong E, Squire MM, Gardner LM. Association between mobility patterns and COVID-19 transmission in the USA: a mathematical modelling study. *Lancet Infect Dis* 2020; **20**(11): 1247-54.
2. Brown KA, Soucy JR, Buchan SA, et al. The mobility gap: estimating mobility thresholds required to control SARS-CoV-2 in Canada. *Cmaj* 2021; **193**(17): E592-e600.
3. Carlitz RD, Makhura MN. Life under lockdown: Illustrating tradeoffs in South Africa's response to COVID-19. *World Dev* 2021; **137**: 105168.
4. Cazelles B, Comiskey C, Nguyen-Van-Yen B, Champagne C, Roche B. Parallel trends in the transmission of SARS-CoV-2 and retail/recreation and public transport mobility during non-lockdown periods. *Int J Infect Dis* 2021; **104**: 693-5.
5. Chen Y, Chen M, Huang B, Wu C, Shi W. Modeling the Spatiotemporal Association Between COVID-19 Transmission and Population Mobility Using Geographically and Temporally Weighted Regression. *Geohealth* 2021; **5**(5): e2021GH000402.
6. da Silva TT, Francisquini R, Nascimento MCV. Meteorological and human mobility data on predicting COVID-19 cases by a novel hybrid decomposition method with anomaly detection analysis: A case study in the capitals of Brazil. *Expert Syst Appl* 2021; **182**: 115190.
7. Díaz-Castro L, Cabello-Rangel H, Hoffman K. The Impact of Health Policies and Sociodemographic Factors on Doubling Time of the COVID-19 Pandemic in Mexico. *Int J Environ Res Public Health* 2021; **18**(5).
8. Fan C, Lee S, Yang Y, Oztekin B, Li Q, Mostafavi A. Effects of population co-location reduction on cross-county transmission risk of COVID-19 in the United States. *Appl Netw Sci* 2021; **6**(1): 14.
9. Gao S, Rao J, Kang Y, et al. Association of Mobile Phone Location Data Indications of Travel and Stay-at-Home Mandates With COVID-19 Infection Rates in the US. *JAMA Netw Open* 2020; **3**(9): e2020485.
10. Gatalo O, Tseng K, Hamilton A, Lin G, Klein E. Associations between phone mobility data and COVID-19 cases. *Lancet Infect Dis* 2020.
11. Glaeser EL, Gorback C, Redding SJ. JUE Insight: How Much does COVID-19 Increase with Mobility? Evidence from New York and Four Other U.S. Cities. *J Urban Econ* 2020: 103292.
12. Iacus SM, Santamaria C, Sermi F, Spyratos S, Tarchi D, Vespe M. Human mobility and COVID-19 initial dynamics. *Nonlinear Dyn* 2020: 1-19.
13. Jamshidi S, Baniasad M, Niyogi D. Global to USA County Scale Analysis of Weather, Urban Density, Mobility, Homestay, and Mask Use on COVID-19. *Int J Environ Res Public Health* 2020; **17**(21).
14. Kajitani Y, Hatayama M. Explaining the effective reproduction number of COVID-19 through mobility and enterprise statistics: Evidence from the first wave in Japan. *PLoS One* 2021; **16**(3): e0247186.
15. Kissler SM, Kishore N, Prabhu M, et al. Reductions in commuting mobility correlate with geographic differences in SARS-CoV-2 prevalence in New York City. *Nat Commun* 2020; **11**(1): 4674.
16. Kraemer MUG, Yang CH, Gutierrez B, et al. The effect of human mobility and control measures on the COVID-19 epidemic in China. *Science* 2020; **368**(6490): 493-7.
17. Lamb MR, Kandula S, Shaman J. Differential COVID-19 case positivity in New York City neighborhoods: Socioeconomic factors and mobility. *Influenza Other Respir Viruses* 2020.
18. Leung K, Wu JT, Leung GM. Real-time tracking and prediction of COVID-19 infection using digital proxies of population mobility and mixing. *Nat Commun* 2021; **12**(1): 1501.
19. Li X, Rudolph AE, Mennis J. Association Between Population Mobility Reductions and New COVID-19 Diagnoses in the United States Along the Urban-Rural Gradient, February-April, 2020. *Prev Chronic Dis* 2020; **17**: E118.
20. Monod M, Blenkinsop A, Xi X, et al. Age groups that sustain resurging COVID-19 epidemics in the United States. *Science* 2021; **371**(6536).
21. Nakanishi M, Shibasaki R, Yamasaki S, et al. On-site Dining in Tokyo During the COVID-19 Pandemic: Time Series Analysis Using Mobile Phone Location Data. *JMIR Mhealth Uhealth* 2021; **9**(5): e27342.
22. Sartorius B, Lawson AB, Pullan RL. Modelling and predicting the spatio-temporal spread of COVID-19, associated deaths and impact of key risk factors in England. *Sci Rep* 2021; **11**(1): 5378.
23. Sehra ST, Kishfy LJ, Brodski A, George MD, Wiebe DJ, Baker JF. Association of cell phone location data and trends in COVID-19 infections during loosening of stay-at-home restrictions. *J Travel Med* 2020; **27**(8).
24. Steiger E, Mussgnug T, Kroll LE. Causal graph analysis of COVID-19 observational data in German districts reveals effects of determining factors on reported case numbers. *PLoS One* 2021; **16**(5): e0237277.
25. Unwin HJT, Mishra S, Bradley VC, et al. State-level tracking of COVID-19 in the United States. *Nat Commun* 2020; **11**(1): 6189.
26. Wang B, Liu J, Li Y, et al. Airborne particulate matter, population mobility and COVID-19: a multi-city study in China. *BMC Public Health* 2020; **20**(1): 1585.

27. Wang S, Liu Y, Hu T. Examining the Change of Human Mobility Adherent to Social Restriction Policies and Its Effect on COVID-19 Cases in Australia. *Int J Environ Res Public Health* 2020; **17**(21).
28. Xiong C, Hu S, Yang M, Luo W, Zhang L. Mobile device data reveal the dynamics in a positive relationship between human mobility and COVID-19 infections. *Proc Natl Acad Sci U S A* 2020; **117**(44): 27087-9.
29. Yang H, Chen D, Jiang Q, Yuan Z. High intensities of population movement were associated with high incidence of COVID-19 during the pandemic. *Epidemiol Infect* 2020; **148**: e177.
30. Younis J, Freitag H, Ruthberg JS, Romanes JP, Nielsen C, Mehta N. Social Media as an Early Proxy for Social Distancing Indicated by the COVID-19 Reproduction Number: Observational Study. *JMIR Public Health Surveill* 2020; **6**(4): e21340.
31. Zheng Z, Xie Z, Qin Y, Wang K, Yu Y, Fu P. Exploring the influence of human mobility factors and spread prediction on early COVID-19 in the USA. *BMC Public Health* 2021; **21**(1): 615.
32. Zhou Y, Xu R, Hu D, Yue Y, Li Q, Xia J. Effects of human mobility restrictions on the spread of COVID-19 in Shenzhen, China: a modelling study using mobile phone data. *Lancet Digit Health* 2020; **2**(8): e417-e24.
33. Zhu Y, Xie J, Huang F, Cao L. The mediating effect of air quality on the association between human mobility and COVID-19 infection in China. *Environ Res* 2020; **189**: 109911.
34. Google LLC. Google COVID-19 Community Mobility Reports. 2021. https://www.google.com/covid19/mobility/ (accessed 17-Feb 2021).
35. Aktay A, Bavadekar S, Cossoul G, et al. Google COVID-19 community mobility reports: Anonymization process description (version 1.0). *arXiv preprint arXiv:200404145* 2020.
36. Apple. Mobility Trends Reports. 2021. https://covid19.apple.com/mobility (accessed 15 Feb 2021).
37. Gandy A, Mishra S. ImperialCollegeLondon/covid19local. 2021. https://zenodo.org/record/4506699 (accessed 17-Feb 2021).
38. Mishra S, Scott J, Zhu H, et al. A COVID-19 Model for Local Authorities of the United Kingdom. *medRxiv* 2020: 2020.11.24.20236661.
39. Northern Ireland Statistics and Research Agency. 2019 Mid Year Population Estimates for Northern Ireland. 2020. https://www.nisra.gov.uk/publications/2019-mid-year-population-estimates-northern-ireland (accessed 27-Jan 2021).
40. Office for National Statistics. Population estimates for the UK, England and Wales, Scotland and Northern Ireland, provisional: mid-2019. 2020. https://www.ons.gov.uk/peoplepopulationandcommunity/populationandmigration/populationestimates/bulletins/annualmidyearpopulationestimates/mid2019#population-age-structure-and-density-for-local-authority-areas (accessed 27-Jan 2021).
41. Legatum Institute. The UK Prospertity Index. 2016. https://uk.prosperity.com/docs/2016/2016UKProsperityDataset.xls (accessed 18-Nov 2020).
42. Institute L. A guide to UK prosperity index. 2016. https://uk.prosperity.com/docs/2016/2016UKProsperityIndexMethodology.pdf (accessed 18-Nov 2020).
43. Statistics OfN. 2011 Census Data On Nomis. 2011. https://www.nomisweb.co.uk/census/2011 (accessed 18-Nov 2020).
